# Simultaneous Ultra-Sensitive Detection of Structural and Single Nucleotide Variants Using Multiplex Droplet Digital PCR in Liquid Biopsies from Children with Medulloblastoma

**DOI:** 10.3390/cancers15071972

**Published:** 2023-03-25

**Authors:** Cecilia Arthur, Cecilia Jylhä, Teresita Díaz de Ståhl, Alia Shamikh, Johanna Sandgren, Richard Rosenquist, Magnus Nordenskjöld, Arja Harila, Gisela Barbany, Ulrika Sandvik, Emma Tham

**Affiliations:** 1 Clinical Genetics, Karolinska University Hospital, 171 76 Stockholm, Sweden; 2 Department of Molecular Medicine and Surgery, Karolinska Institutet, 171 76 Stockholm, Sweden; 3 Department of Oncology-Pathology, Karolinska Institutet, 171 77 Stockholm, Sweden; 4 Clinical Pathology and Cancer Diagnostics, Karolinska University Hospital, 171 76 Stockholm, Sweden; 5 Department of Women’s and Children’s Health, Uppsala University, 751 85 Uppsala, Sweden; 6 Department of Clinical Neuroscience, Division of Neurosurgery, Karolinska Institutet, 171 77 Stockholm, Sweden

**Keywords:** medulloblastoma, liquid biopsy, cell free DNA, cerebrospinal fluid, plasma, MRD, biomarker, ddPCR

## Abstract

**Simple Summary:**

Medulloblastoma is one of the most common types of brain tumors in children. During and after treatment with surgery, chemotherapy, and/or radiotherapy, children with this disease are monitored with imaging and cerebrospinal fluid analysis for the detection of tumor cells. These methods are not always sensitive or specific enough to confirm or rule out residual disease. Here, we develop a laboratory test based on the genetic makeup of medulloblastomas in 12 children. We analyze liquid biopsies (cerebrospinal fluid and blood plasma) for specific genetic fragments leaking from the individual tumors and find molecular traces of disease in 75% (9/12) of children overall. None of the children had malignant cells in the cerebrospinal fluid. We propose that this test could open up new technical possibilities to track measurable residual disease in children with medulloblastoma in order to further risk-adapt treatment, but first, larger studies of the approach at standardized time points are warranted.

**Abstract:**

Medulloblastoma is a malignant embryonal tumor of the central nervous system (CNS) that mainly affects infants and children. Prognosis is highly variable, and molecular biomarkers for measurable residual disease (MRD) detection are lacking. Analysis of cell-free DNA (cfDNA) in cerebrospinal fluid (CSF) using broad genomic approaches, such as low-coverage whole-genome sequencing, has shown promising prognostic value. However, more sensitive methods are needed for MRD analysis. Here, we show the technical feasibility of capturing medulloblastoma-associated structural variants and point mutations simultaneously in cfDNA using multiplexed droplet digital PCR (ddPCR). Assay sensitivity was assessed with a dilution series of tumor in normal genomic DNA, and the limit of detection was below 100 pg of input DNA for all assays. False positive rates were zero for structural variant assays. Liquid biopsies (CSF and plasma, n = 47) were analyzed from 12 children with medulloblastoma, all with negative CSF cytology. MRD was detected in 75% (9/12) of patients overall. In CSF samples taken before or within 21 days of surgery, MRD was detected in 88% (7/8) of patients with localized disease and in one patient with the metastasized disease. Our results suggest that this approach could expand the utility of ddPCR and complement broader analyses of cfDNA for MRD detection.

## 1. Introduction

Medulloblastoma (MB) is one of the most common malignant central nervous system (CNS) tumors affecting infants and children [1,2]. It is a biologically heterogeneous disease of embryonal origin that is prone to spread to the spinal cord and meninges. Diagnosis is based on clinical evaluation, neuroimaging, and histopathological and molecular features of tissue biopsies. MB is divided into four principal molecular groups; WNT-activated, SHH-activated, group 3, and 4 [3,4]. The SHH group is divided into four subgroups and groups 3 and 4 into eight subgroups, all with their own clinicopathological and genetic features [5].

Treatment of MB includes surgery, adjuvant chemotherapy, and in most cases, cranial or craniospinal radiotherapy (RT), depending on the age of the child, the histopathological and molecular features of the tumor, the radicality of surgery and the metastatic status of the disease. Infants and young children (<3 years of age) receive multiagent chemotherapy, often with autologous hematopoietic stem cell rescue, to delay or avoid irradiating the developing brain and spinal cord. Standard-risk patients (children ≥ 3 years of age with total or near-total resection of the tumor, no evidence of disseminated disease, and classic or nodular desmoplastic histology) typically receive craniospinal RT followed by adjuvant multiagent chemotherapy. Treatment of high-risk MB is challenging and includes intensive chemotherapy and higher RT doses than in the standard risk protocols [6,7]. Prognosis is widely diverse. While the five-year overall survival is ~80% for non-infant patients with non-metastatic MB and successful total tumor resection, it is ~60% for children with metastatic disease or non-radical surgery. The WNT group has the highest five-year overall survival (>95%), while group 3 has the lowest (<60%) [8]. Although a majority of children with MB survive their disease [9,10,11], many survivors have severe long-term toxicity effects of treatment, impacting their quality of life [12,13].

Recent studies using whole-genome sequencing (WGS) and other high-throughput technologies have transformed cancer research by greatly expanding our knowledge of the genetic events that drive malignant transformation in pediatric cancers in general [14,15,16] and in MBs specifically [17,18]. These techniques are increasingly being implemented in the clinical setting and have the advantage of providing a comprehensive map of the clonal genetic aberrations that can potentially be used as biomarkers [19,20].

Molecular biomarkers for measurable residual disease (MRD) detection are lacking in MB. Patients are thus monitored by clinical assessment, imaging (magnetic resonance imaging, MRI), and cerebrospinal fluid (CSF) cytology. In short, the residual disease is assessed by visualization of malignant cells. Disadvantages of MRI include the frequent need for general anesthesia and the occasional inability to discriminate between residual tumor tissue and post-therapeutic changes (fluids, blood, or contrast-enhanced reactive tissue in the surgical cavity or irradiation artifacts). CSF sampling requires less anesthesia, but cytology has low sensitivity [21,22]. These methodological shortcomings sometimes leave clinicians in the dark and may lead to the under or over-treatment of disease, both scenarios associated with their own detrimental consequences. Molecular biomarkers could help resolve some of these issues.

MRD is paramount for the precision treatment of several hematological cancers. Acute lymphoblastic leukemia (ALL), the most common cancer in children, is a prime example. MRD has played a central role in the dramatic improvement in the outcome of childhood ALL over the last decades and is a pivotal parameter in risk-adapted treatment protocols [23,24]. An MRD marker in childhood MB should ideally reflect some intrinsic feature specific to the main malignant clone and be accessible by minimally invasive procedures.

Circulating tumor DNA (ctDNA) has emerged as a potential biomarker for different cancers, and an increasing number of studies explore ctDNA for MRD detection in solid tumors [25,26,27]. These short-lived fragments of free-floating DNA wrapped around nucleosomes that leak from dying tumor cells, reflect the genetic aberrations present in the tumor cells and are found in many body fluids accessible through minimally invasive procedures [28,29,30]. Early studies on liquid biopsies for CNS tumors mainly focused on the detection of ctDNA in different body fluids (plasma, serum, CSF) with targeted methods or small gene panels. These studies showed that CSF is superior to blood for ctDNA detection, but few children with MB were included in the studies [25,31,32,33,34,35].

In more recent years, a few smaller studies have shown the possibility of characterizing, risk stratifying, and/or monitoring MBs based on ctDNA in CSF, plasma, and/or urine, using broader genomic methods (whole-exome sequencing, low-coverage WGS; lcWGS, large gene panels or whole-genome bisulfite sequencing), sometimes combined with targeted approaches to validate findings (droplet digital PCR, ddPCR, for single plex single nucleotide variants, SNVs, or targeted next-generation sequencing, NGS) [36,37,38,39,40]. One larger study applying lcWGS for the detection of copy number aberrations (CNAs) has shown the prognostic value of ctDNA in childhood MB at the end of therapy [41], but this method does not capture copy-number-neutral aberrations and/or point mutations. These alterations may be particularly interesting if affecting a pathway that can be targeted with precision treatment, such as PTCH1 mutations and the Hedgehog pathway.

ddPCR is an ultrasensitive technology that offers detection and absolute quantification of low abundance targets. Here, we test the technical feasibility of ddPCR to provide an MRD method that targets different kinds of genetic alterations in MB. We show that using a tumor-informed approach based on WGS data enables the development of highly sensitive assays that simultaneously capture SNVs and structural variants (SVs). ctDNA positivity in CSF and/or plasma was observed in 75% (9/12) of patients, all of whom had negative CSF cytology throughout the course of the disease. This method could be complementary to broader methods, such as lcWGS, for MRD detection in MB and should be explored in future studies.

## 2. Materials and Methods

### 2.1. Patient Material

In this study, 12 children with MB were included, all diagnosed between 2017 and 2021 and treated surgically at Karolinska University Hospital, Stockholm, Sweden, and 11/12 patients received adjuvant treatment at the same site. Clinical data were collected from medical records. The study was reviewed and approved by the Swedish Ethical Review Authority (2016/2-31/1, 2017/599-32/1, 2018/1472-32/1, 2019-01222, 2016/429-31/2, 2018/1484-32/2, 2019-01221). Written informed consent to participate in this study was provided by the patients’ legal guardian/next of kin.

Routine investigations of diagnostic tumor tissue samples from patients diagnosed in 2017 to 2019 included fluorescence in situ hybridization (FISH) for amplifications of *MYC* (alias *c-MYC*), *MYCN* (alias *NMYC*) and monosomy of chromosome 6, as well as Sanger sequencing for SNVs in *CTNNB1* (NM_001904.4, exons 3 and 5) and *TP53* (NM_000546.6, exons 4, 5, 6, 7 and 8). Diagnostic tumor tissue samples from patients diagnosed in 2020 and onwards were analyzed with amplicon sequencing using the Oncomine™ Childhood Cancer Research Assay, Chef-Ready on Ion Torrent™ S5 (both Thermo Fisher Scientific Waltham, MA, USA).

CSF samples for cytology were collected in sterile tubes and processed within 60 min after arrival at the pathology department. Cell count and morphologic examination of CSF were performed on cytospin preparations (1000 rpm for 5 min). In samples with a normal cell count (<5 × 10^3^ cells/mL), morphological assessment was performed after staining with May-Grünwald Giemsa (MGG). In samples with an increased cell count (>5 × 10^3^ cells/mL), additional immunocytochemical staining for synaptophysin (major synaptic vesicle protein p38) was performed according to standard procedures. If no malignant cells were identified, the result was termed “benign” [42].

Liquid biopsies (CSF and blood) were taken in connection with clinical sampling at any time point that material could be spared. CSF samples were taken intraoperatively, prior to tumor resection, and/or post-operatively through a lumbar puncture in accordance with the patients’ treatment protocols. Whenever possible, a paired blood sample was taken at the same time point. Samples were collected in Cell-Free DNA (cfDNA) BCT tubes (10 mL) (STRECK, La Vista, NE, USA) and kept at RT until further processed within 5 days. Volumes were 1–5 mL for plasma and 0.8–8.8 mL for CSF. After cell removal by double centrifugation (10 min at 4 °C 1600× *g* and 16,000× *g*), supernatants were frozen at −80 °C. Cell-free supernatants were thawed and cfDNA isolated using the QiAamp Circulating Nucleic Acid Kit on the QIAvac24 Plus vacuum manifold and the Qiacube (Qiagen, Manchester, UK). cfDNA was eluted in 40 μL of AVE buffer and was stored at −20 °C for a maximum of 3 months.

Plasma from blood donors was processed in the same manner and used as a negative control (NC) for liquid biopsies. NC genomic DNA (gDNA) was extracted from peripheral blood (PB) collected in EDTA, BD Vacutainer^®^ tubes (Becton Dickinson, Franklin Lakes, NJ, USA) from non-cancer patients using QIAamp DNA Blood Maxi kit (Qiagen, Hilden, Germany) and was anonymized and pooled prior to use for ddPCR assay testing.

### 2.2. Whole-Genome Sequencing and DNA Methylation Profiling

Fresh frozen tumors and patient-matched blood samples for WGS were available from all patients at The Swedish Childhood Tumor Biobank/Karolinska University Hospital biobank. gDNA from tumor samples (positive control, PC) was isolated using AllPrep DNA/RNA/protein Mini kit (Qiagen, Hilden, Germany). Matched genomic DNA for each patient was extracted from PB (EDTA, BD Vacutainer^®^) using QIAamp^®^ DNA Blood Midi/Maxi kit, vacuum protocol (Qiagen, Hilden, Germany). The quantification and qualification of extracted nucleic acids were performed by spectrophotometric (ND-1000 spectrophotometer, NanoDrop Technologies, Wilmington, DE, USA) and fluorescence-based methods (Qubit^®^ fluorimeter using Qubit™ dsDNA BR Assay Kit, Invitrogen™, Thermo Fisher Scientific, Waltham, MA, USA). gDNA was stored at −80 °C until use.

Libraries for WGS were prepared with TruSeq PCR-free kit (Illumina, SanDiego, CA, USA) with 350 bp insert size, and WGS samples were sequenced on either the NovaSeq 6000 or the HiSeqX v2.5 instrument, 2 × 150 bp paired-end (Illumina, SanDiego, CA, USA). gDNA-input was 1µg, and coverage was 45/30× for tumor/normal, respectively. Library preparation and sequencing were performed at the Genomic Production Center, ScilifeLab, Stockholm, Sweden. Sequence data was delivered in FASTQ format, using Illumina 1.8 quality scores.

DNA sequence data were processed via Sarek v2.7.1, an open-source workflow for short read alignment and processing, following the GATK best-practice recommendations [43]. Briefly, the steps run were: quality control of FASTQ files using FASTQC (https://www.bioinformatics.babraham.ac.uk/projects/fastqc/ (accessed on 7 July 2021)), alignment of short reads to the human reference genome sequence (GRCh38/hg38) using bwa-mem, with the ALT-aware option turned on (VN:0.7.17-r1188, https://arxiv.org/abs/1303.3997 (accessed on 14 February 2022)), sorting of reads and marking of PCR duplicates with GATK MarkDuplicates and base quality scores recalibration and jointly realignment of reads around insertions and deletions (indels), using GATK tools (v4.1.7.0, https://github.com/broadinstitute (accessed on 22 March 2022)).

Somatic SNV and indel calls were generated by GATK MuTect2 using the resource bundle 1000g_pon.hg38.vcf.gz panel of normals file (v4.1.7.0, https://www.biorxiv.org/content/10.1101/861054v1 (accessed on 22 March 2022)). Somatic structural variants (SVs) were called by Manta (v1.6.0) [44]. Variant annotation was done within Sarek using snpEff (v4.3t) [45] and VEP (v99.2) [46]. Copy-number profiles, ploidy, and tumor cell ratios were generated by Control-FREEC (v11.6) [47] and ASCAT (v2.5.2) [48]. Quality control metrics and coverage were collated by MultiQC [49].

The annotated lists of variants obtained after running Sarek were further processed accordingly: only somatic mutations called by Mutect2 with alternate allele count ≥ 5 and flagged as PASS were retained. Selected Mutect2 variants were further filtered using filter_vep ((MAX_AF ≤ 0.001 or not MAX_AF) and (CADD_PHRED ≥ 20 or not CADD_PHRED) and (IMPACT is HIGH or IMPACT is MODERATE)). Only somatic variants called by Manta, supported by both spanning paired (PR) and split reads (SR) with alternate allele count ≥ 5 (PR ≥ 5 and SR ≥ 5), with no more than one supportive read in the matched normal sample, and flagged as PASS, were considered. Further, only Manta somatic SVs with a somatic variant quality score (SOMATICSCORE) ≥ 50 were retained. Additionally, predicted somatic fusion genes were identified from somatic Manta SVs by the SnpEff tool [45].

All the somatic variants (SNV, INDELs, CNAs, SVs, and fusion genes) were further clinically prioritized by selecting mutations in genes reported to be involved in cancer, including cancer census genes (Cosmic v90), and more specifically, in genes recurrently altered in pediatric tumors including MB as identified in previous studies [14,15,16,18]. Read alignments were inspected using the Integrative Genomics Viewer [50].

Tumor DNA methylation profiling was performed at the SNP&SEQ Technology Platform, Uppsala, Sweden (https://www.scilifelab.se/units/ngiuppsala/ (accessed on 4 April 2021)) using Infinium Human Methylation 450 or EPIC Bead Chip arrays (both Illumina), and 500 ng of gDNA derived from fresh-frozen tumors was used as input material. IDAT files were processed at the German Cancer Research Center website (DKFZ, https://www.molecularneuropathology.org/mnp (accessed on 10 October 2022)) and assigned to methylation classes using the brain tumor methylation classifier v11b4 [51]. Methylation profiling was performed to refine subgroups based on clinical data.

### 2.3. Droplet Digital PCR

For droplet digital PCR, we aimed at developing assays targeting ≥ 2 tumor-specific genetic aberrations per patient, ideally both one SNV and one SV. The following criteria were applied to select target sequences from WGS data:

(i) events typical for MB according to the WHO Classification of Central Nervous System Tumors, 5th edition [5];
(ii) recurrent genomic events described in the MB literature [17,18];
(iii) novel, tumor-specific junction sequences resulting from SVs.

We prioritized events based on the supporting number of reads. Non-recurrent SNVs were checked for impact in Cosmic (v95, https://cancer.sanger.ac.uk/cosmic (accessed on 29 March 2022)), ClinVar (https://www.ncbi.nlm.nih.gov/clinvar/ (accessed on 29 March 2022)), and cBioPortal (v4.0.0–4.1.4, Curated set of non-redundant studies, https://www.cbioportal.org/ (accessed on 5 April 2022)) in March/April of 2022 [52,53,54,55]. For relapsed cases, targets should be present in both the primary tumor and secondary lesion.

SNV assays were ordered as Wet-Lab Validated ddPCR Assays for Mutation Detection, if available (Bulletin 7144, BioRad, Hercules, CA, USA) otherwise generated using the BioRad Assay Design Engine and purchased from BioRad (USA) [56,57]. Patient-specific SV assays covering breakpoint sequences were designed using Primer3Plus software according to the Rare Mutation Detection Best Practices Guidelines (BioRad, Hercules, CA, USA) [58,59]. In Silico PCR and Human BLAT Search were performed to check specificity [60]. SV assay primers and probes were purchased from Integrated DNA Technologies™ (IDT, USA). Amplicon sizes were <120 bp for all assays. Target probes were labeled with 5′ 6-FAM™ fluorophores and 3′ Iowa Black^®^FQ quenchers. Probes for reference sequences, either a reference gene (*ABCC9*, dHsaCP2506567, BioRad, Hercules, CA, USA) or an SNV wildtype (wt) sequence, were labeled with 5′ HEX fluorophores and 3′ BHQ-1/Iowa Black^®^FQ quenchers. The *ABCC9* gene was chosen as a reference for the SV targets based on personal experience with this assay. All reference sequences (*ABCC9* and SNV wt assays) were located in copy-number-neutral regions. Sequence details are available upon request.

ddPCR reactions were run on the QX200 AutoDG Droplet Digital PCR System/QX200 Droplet Reader (BioRad, Hercules, CA, USA) according to the manufacturer’s instructions, applying both Poisson and total error with 95% CI.

First, optimal annealing temperatures for single plex assays were determined. Gradient-ddPCRs (55–65 °C) were run on PC gDNA and NC gDNA in parallel. All reactions contained a reference sequence assay to control DNA concentration and amplifiability. Reactions contained: 5.5 μL of ddPCR Multiplex Supermix 4X (BioRad, Hercules, CA, USA), 1 μL of 20× assay mix for target, 1 μL of 20× assay mix for reference sequence (all assays with 900/450 nM of primers/probes, respectively), 11 μL (10 ng) of gDNA and nuclease-free water up to a total volume of 22 μL per well. Thermal cycling conditions were: 1 cycle at 95 °C for 10 min (enzyme activation), 40 cycles at 94 °C for 30 s (denaturation) and individualized annealing temperature for 1 min (extension), 1 cycle at 98 °C for 10 min (enzyme deactivation), and 1 cycle at 4 °C ∞ at ramp rates of 2 °C/s. Results were visually reviewed in the QX Manager Software (BioRad, Hercules, CA, USA).

Second, the limit of detection (LoD) was assessed by ddPCR on a dilution series of undigested PC gDNA in NC gDNA at a constant total input of 10 ng, ranging from 10^−1^ to 10^−4^.

Last, amplitude multiplexing was performed according to the Applications guide to enable simultaneous detection and discrimination of all targets for each patient in one reaction [61].

For liquid biopsy analysis, 11 μL of cfDNA eluate was loaded in triplicate ddPCR reactions with the multiplexed assay mix using the same cycling conditions as above. Control samples (no template control, NC gDNA, and PC gDNA) in triplicates and 12 wells of NC cfDNA extracted from a total of 20 mL of donor plasma were run on all plates.

Output data were manually reviewed, and thresholds were set for negative, single, and double positive droplets based on the control samples included in each run according to the provider’s instructions. To call a sample positive, three or more droplets with FAM-fluorescence were required, with no overlapping error bars between the sample and the NC cfDNA. Borderline samples were called trace positive if ≥3 mutant droplets were observed, and the assay had a false-positive rate (FPR) of zero. FPR (events/well) for each assay was calculated from the total number of positive mutant events detected in 12 wells of NC cfDNA.

Copies of target/reference molecules per ml of the liquid biopsy were calculated by dividing the sum of all FAM or HEX signals (merged data from triplicate ddPCR reactions) by the initial liquid biopsy volume after correction for input volume (33 of 40 μL). Total cfDNA was calculated from *ABCC9* HEX signals or SNV HEX + FAM signals from wt and mutant molecules of an SNV target, respectively. Conversion of raw data (copies/μL) to haploid genomic equivalents (hGEs) and/or mass was performed as outlined in Appendix B of the Rare Mutation Detection Best Practices Guidelines for Droplet Digital™ PCR (BioRad, Hercules, CA, USA) [59].

## 3. Results

### 3.1. Participants’ Characteristics

Twelve children ages 1–15 years old were included in the study: three patients had MBs in the WNT group; four in the SHH group (all *TP53* wt); and five in the non-WNT, non-SHH group according to clinical data. Eleven patients had localized disease and were included at primary diagnosis; one of these patients developed a local cerebellar relapse during the study (patient 19). One patient (patient 15) was included at relapse with metastatic disease (intracranial and spinal). Median follow-up time was three years and four months for the patients with localized disease, all of whom were progression-free at the last clinical evaluation. The patient with metastatic disease passed away one year and two months after the relapse diagnosis.

CSF and plasma were sampled in conjunction with clinical procedures. WGS data were generated for all patients. Methylation array data were available for all patients except one (patient 43) and provided subtypes for all non-WNT cases (Table 1). CNAs detected by methylation were concordant with those detected by WGS. Further details on WGS and methylation data are available in Supplementary Figure S1 and Table S1.

### 3.2. Target Identification in WGS Tumor Data

Suitable target sequences were found in WGS tumor data for all patients. Four target sequences had been previously detected in tumor DNA by standard-of-care (SNVs in either the *CTNNB1*, *SMO*, or *KDM6A* genes). We detected these and another 28 potential targets. Among the 28 sequences, we identified 12 additional driver events, or events recurrent in MB, and 11/12 patients (all except patient 21) had at least one such event targeted with a ddPCR assay.

### 3.3. ddPCR Assay Performance; Sensitivity and False-Positive Rates

In total, 32 assays were designed and tested, and 8/32 assays were discarded: four SNV-based assays due to lack of specificity (background in NC samples) and four SV assays due to redundancy and overlapping FAM fluorescence with other SV assays. The remaining 24 assays, 15 for SNVs and nine for SVs, were tested for the limit of detection (LoD) by ten-fold dilutions of PC in NC gDNA, with a total gDNA input of 10 ng in each reaction. The final sequences used as targets are listed in Table 2. Details on all sequences tested are available in Supplementary Table S2.

All assays could detect 100 pg of genomic tumor DNA, and six assays could detect 10 pg, corresponding to 30 and 3 hGEs, respectively. Most assays (14/24) had FPRs of 0 (all SV assays and five SNV assays), while the remaining 10 SNV assays had FPRs of 0.08–1.58 events/well (corresponding to 0.11–2.17 events/mL of NC plasma). Dilution series data are available in Supplementary File S1.

### 3.4. ctDNA Detection in CSF Samples

**Figure 1:**
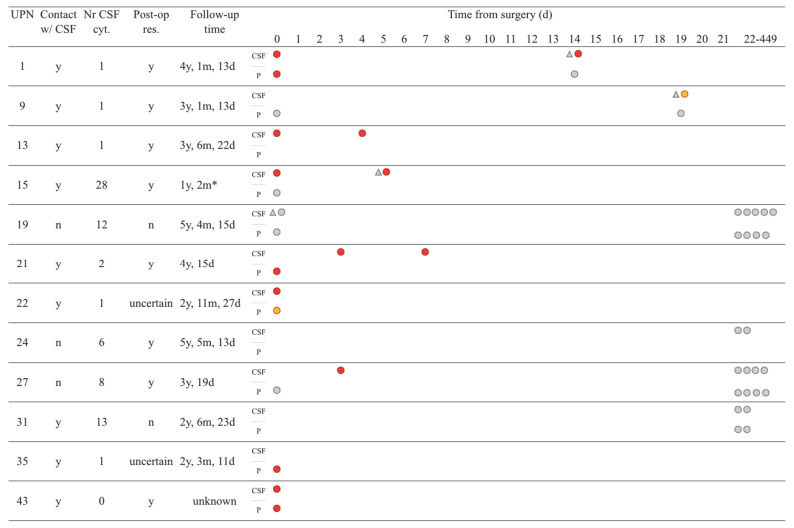
Liquid biopsy and CSF cytology results from surgery until three weeks post-operatively. Tumor location in relation to CSF reservoir as denoted on diagnostic MRI (contact with CSF; yes/no). Residual tumor tissue as denoted on post-operative MRI (post-op res; yes/no). Follow-up time based on the date of last MRI (y, year; m, month; d, day, asterisk denotes diseased). CSF, cerebrospinal fluid; P, plasma. CSF cytology (triangles) and ctDNA results (circles). ctDNA positive samples in red, trace positive samples in orange, and negative samples in grey.

CSF samples (n = 26) were available for 11/12 patients (all except patient 35) (Figure 1). Samples taken on the day of surgery prior to resection (d0, n = 6) were ctDNA positive for five patients: four patients undergoing primary surgery for localized disease and one patient (patient 15) who had surgery at relapse for one of two spinal metastases. The negative d0 sample came from patient 19, who had a localized, well-defined parenchymal tumor in the left cerebellar hemisphere, while the other tumors were all in contact with CSF.

Post-operative CSF samples taken within 21 days of surgery (n = 7) were all ctDNA positive or trace positive: 6/7 samples were from patients with localized disease, and one sample was from patient 15 with the metastasized disease. On d19, minute amounts of ctDNA were seen in CSF in patient 9 (localized disease), with 2 and 4 clearly positive FAM fluorescent droplets for two assays (duplication of 1p21.1 and *CSNK2B*, respectively). Both assays had an FPR of zero; hence, the sample was called trace positive for *CSNK2B* despite the very low levels of cfDNA (18 hGEs/mL corresponding to 50 pg of DNA/mL). No CSF sample prior to d19 was available from this patient.

Total cfDNA levels in ctDNA positive CSF samples were highly variable; 18–62,992 hGEs/mL (median 8573 hGEs/mL corresponding to 28.3 ng DNA/mL). Unfortunately, most of the d15 CSF samples were sent for cytology only, and there was no CSF left at the clinical lab for additional analyses.

Samples taken more than three weeks after surgery (d22–d449, n = 13) were all ctDNA negative, including all sequential samples from patient 19. Total cfDNA levels in ctDNA negative CSF samples were 3–467 hGEs/mL (median 20 hGEs/mL corresponding to 66 pg DNA/mL). Thus, ctDNA positive CSF samples had considerably higher median cfDNA levels than ctDNA negative samples, with a small overlap in concentration range. CSF sample volumes were comparable between ctDNA positive and negative samples (mean volume 2.3 and 1.9 mL, respectively).

In summary, ctDNA positivity was seen in CSF samples taken within three weeks of surgery in 89% (8/9) of patients: in 88% (7/8) of patients with localized disease, including the trace positive sample, and in the only patient with metastasized disease (patient 15). ctDNA positivity was seen in 73% (8/11) of patients with CSF samples taken at any timepoint; in 70% (7/10) of patients with localized disease, including the trace positive sample, and in the only patient with metastasized disease (patient 15).

### 3.5. ctDNA Detection in Plasma Samples

Plasma samples (n = 21) were received from 10/12 patients (all except patients 13 and 24). d0 samples were available from eight patients with localized disease and one patient with metastasized disease (patient 15). Four samples were ctDNA positive and one trace positive (patient 22); all from patients with localized disease. Both SNV and SV assays could detect ctDNA. The total cfDNA amount in positive plasma samples was 924–14,632 hGEs/mL (median 1526 hGEs/mL corresponding to 5.0 ng DNA/mL). The ctDNA positive sample with the lowest cfDNA amount (patient 35) had 924 hGE/mL, corresponding to 3.0 ng DNA/mL. Four out of five positive plasma samples had a paired ctDNA positive CSF sample taken d0–d3.

All post-op plasma samples (n = 12) were ctDNA negative. The total cfDNA amount in negative plasma samples (n = 16) was 489–4866 hGEs/mL (median 1556 hGEs/mL corresponding to 5.1 ng DNA/mL). Plasma sample volumes were comparable between ctDNA positive and negative samples (mean volume 2.5 and 3.1 mL, respectively), as was the median total cfDNA.

In summary, ctDNA positivity in plasma was observed on d0 in 63% (5/8) of patients with localized disease, including the trace positive case, while the patient with metastatic disease was ctDNA negative.

Plasma and CSF samples taken at the same time point (n = 15) were compared regarding ctDNA levels. Five samples were taken on d0 and ten post-operatively; 12/15 pairs were concordant, two ctDNA positive and ten ctDNA negative. ctDNA positive pairs had very low levels of targets in plasma (3–10 hGE/mL) compared to CSF (4132–8554 hGE/mL). Three pairs were discordant; all were ctDNA positive in CSF and ctDNA negative in plasma. A summary of all ctDNA results is available in Supplementary File S2 and Table S3.

### 3.6. CSF Cytology

CSF cytology results from post-op samples taken around d15 were available for 11/12 patients (all except patient 43). For 6/11 patients, additional follow-up samples were available, including from both patients with relapses (15 and 19). All samples for all patients (n = 74) were negative for malignant cells, and 20% (15/74) of samples had increased cell counts, some with morphological inflammation.

**Figure 2:**
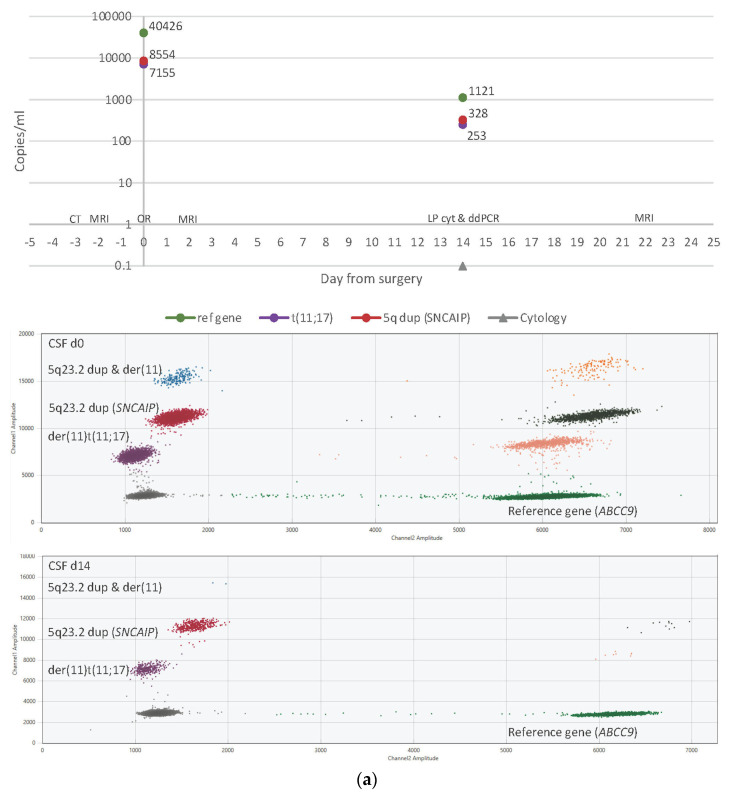

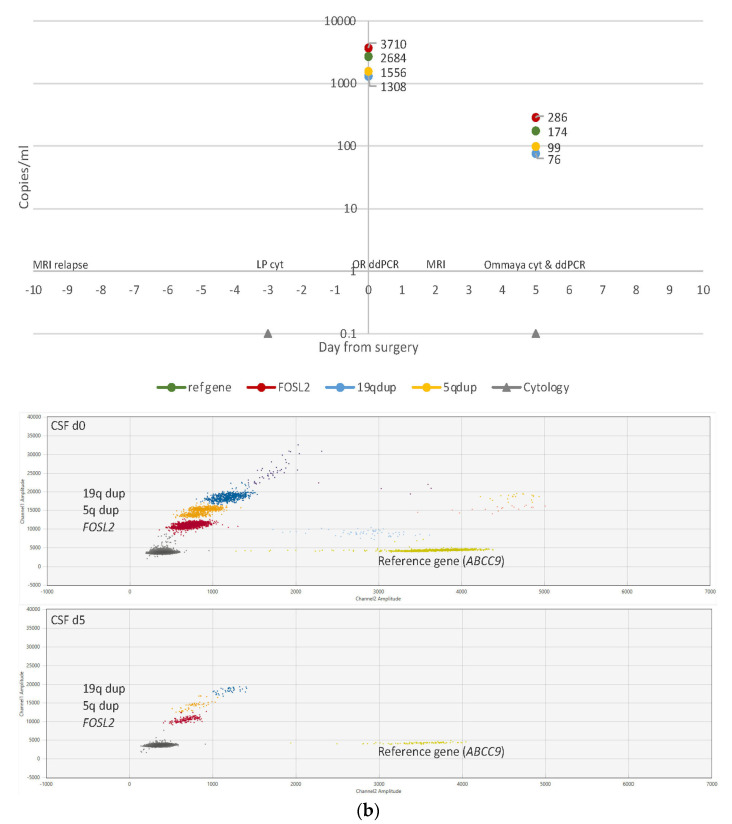
Timelines (top panels) with sampling time points and results from CSF ctDNA ddPCR and CSF cytology. 2D fluorescence amplitude plots (bottom panels) showing output data generated by the QX Manager™ Software (BioRad, Hercules, CA, USA). *Y*-axis, end-point FAM-fluorescence from targets. *X*-axis, end-point HEX-fluorescence from reference gene (*ABCC9*). (**a**) Results for patient 1 on d0, prior to surgery, and d14 post-operatively. Targets: der(11)t(11;17) in purple, 5q dup/*SNCAIP* in red, and both targets in blue. Empty droplets in grey, reference positive droplets in green. Double positives (target and reference) in multiple colors on the right-hand side of the plot: der(11) and reference in pink, 5q dup and reference in dark green, both targets and reference in orange (**b**) Results for patient 15 on d0, prior to surgery, and d5 post-operatively. Targets: *FOSL2* c.383G>A in red, 5q13.3 tandem duplication in orange, and 19q12 tandem duplication in blue, and all targets in purple.. Empty droplets in grey, reference positive droplets in mustard. Double positives (target and reference) in multiple colors on the right-hand side of the plot: *FOSL2* and reference in light blue, 5q13.3 tandem dup and reference in pink, 19q12 tandem dup and reference in yellow, all targets and reference in wine.

From the CSF samples for cytology, 8/74 were also analyzed for ctDNA, and 3/8 were ctDNA positive or trace positive. Two ctDNA positive/trace positive samples were taken by lumbar puncture (from patient 1 on d14 and patient 9 on d19) and were comparable in volumes between cytology and ctDNA samples (1.2–2 mL). Both samples had increased cell counts (>5000 cells/mL), why complementary synaptophysin analysis was performed with negative results. ctDNA results over time for patient 1 is shown in Figure 2a.

The third ctDNA positive sample was taken from an Ommaya reservoir (from patient 15 with metastatic relapse) on d5, and 7 mL of CSF was sent for cytology, showing normal cell counts and benign morphology, while 0.8 mL was analyzed for ctDNA and showed 76–286 hGE/mL of the three tumor targets (illustrated in Figure 2b).

### 3.7. Imaging Results

Imaging results were available for all patients at diagnosis and at first post-operative control and for 11/12 patients during follow-up. Imaging (CT or MRI) was performed in the cohort on 184 occasions.

In all patients where tumors grew into a CSF reservoir, and imaging showed a post-op residual tumor, or where such residual could not be ruled out, ctDNA was positive or trace positive on at least one occasion (n = 9). In 2/3 patients where the tumors did not grow into a CSF reservoir, ctDNA was negative (patients 19 and 24). ctDNA was also undetectable in patient 31 whose post-operative imaging showed radical extirpation of the tumor several weeks before the liquid biopsy was taken (patient 31).

## 4. Discussion

Pediatric oncology is increasingly moving towards genomic profiling of tumor/normal samples from children with cancer in order to identify underlying germline conditions, somatic drivers, predictive and prognostic markers, as well as therapeutic targets [62]. A collateral benefit of this kind of genome-wide profiling is the identification of unique tumor sequences that can be used as biomarkers in ctDNA analysis. These sequences ideally reflect truncal driving events, on which the tumor depends for its survival and clonal expansion, and thus are preserved during the course of the disease. Sometimes, however, singular-driving genetic events are not identified. Instead, a few or multiple complex chromosomal rearrangements are seen as the result of a genomic catastrophic event leading to oncogenic transformation. The novel junction sequences in the genome after such an event may also serve as highly specific tumor biomarker sequences.

WGS analysis, although costly, enables the detection of all types of genetic aberrations (SNVs, indels, SVs, and CNAs), making comprehensive tumor characterization and identification of MRD targets possible in one seamless workflow. In addition, WGS data will be the key to the implementation of new and more precise therapeutic strategies that are currently being developed. WGS is not globally available, but in some countries, WGS profiling of tumors is becoming standard procedure for diagnostics, at least for certain cancer types such as pediatric cancer (e.g., via Genomic Medicine Sweden), and in these countries, it is reasonable to make use of the data for MRD assessment [20,63]. Rapidly decreasing sequencing costs will likely also stimulate the gradual implementation of WGS in cancer diagnostics in the near future.

In this study, we used WGS data to develop multiplexed ddPCR assays against tumor-specific alterations. The multi-target approach was applied to minimize false negative results that may derive from the unavoidable subsampling error associated with low-concentration samples or from the clonal evolution of tumor cells and subsequent loss of a target. The tumor targets identified and selected for ddPCR design are discussed for each molecular group separately in greater detail below.

All three patients in the WNT group (9, 13, 35) showed a somatic hotspot SNV in *CTNNB1* exon 3. Patients 9 and 13 had another SNV in a gene encoding a subunit of the SWI/SNF nucleosome-remodeling complex (*CSNK2B, PIK3CA*), which are typical aberrations within this group [18]. Patients 9 and 35 also displayed SVs causing unique sequences in the tumor cells not previously described in the literature (duplications of 1p21.1 and 13q14.2, respectively), which were also used as ddPCR targets. This was not unexpected as WNT-activated MB genomes have no known recurrent focal somatic CNAs [17], with monosomy 6 being the only characteristic cytogenetic feature (present in patients 9 and 13) [64].

All four patients in the SHH group (19, 24, 27, and 31) carried at least one SNV in an SHH signaling pathway gene (*PTCH1, SMO*) or a gene recurrently mutated in SHH-activated MB (*KMT2D, MAX*) [18]. Patient 24 had a *PTCH1* driver mutation; however, the SNV assay failed (two designs; one due to poor cluster separation of wt and mutant droplets and one due to cross-hybridization of the mutant probe to the wt sequence). This patient showed an additional SNV (non-synonymous missense) annotated as likely pathogenic in the ClinVar database in *PRMT7*, a gene that modulates SHH signaling through methylation and suppression of GLI2 binding to SUFU [65]. The *PRMT7* SNV was, therefore, the only target in this patient.

Patient 27 displayed a truncating germline *SUFU* variant discovered through clinical testing (c.1309_1310insT, p.Glu437Valfs*30, ACMG class 4, paternally inherited), causing Gorlin syndrome (also known as Basal cell nevus syndrome, autosomal dominant, MIM #109400). The *SUFU* second hit was a copy number neutral loss of heterozygosity of the wt allele (10q24.32) and not a suitable target. We, therefore, selected an additional SNV target (splice donor variant annotated as pathogenic in the ClinVar database) in *OFD1,* a gene located on chromosome X (Xp22.2) encoding a centriole and centriolar satellite protein [66]. Other splice variants in the same gene have been described in several cancer types, including glioblastoma in cBioPortal.

None of the patients in group 2 had an SV suitable for assay design due to the presence of highly repetitive regions, few reads supporting variants, low coverage, and/or artifacts.

The five patients in group 3/4 (non-WNT, non-SHH; 1, 15, 21, 22, 43) did not share any common aberration. Patient 15 carried an SNV (non-synonymous missense) annotated as pathogenic in the Cosmic database in the *FOSL2* gene on 2p23.2, seen in both the primary tumor and the relapse (spinal metastasis). The same gene was also affected by a duplication. *FOSL2* (previously *FRA2*) encodes a leucine zipper protein and a subunit of the transcription factor complex AP-1 [66]. The same variant has been reported in two types of carcinomas in cBioPortal, and several studies point towards an oncogenic potential of FOSL2 in tumor cell growth and metastasis [67,68,69].

Patient 21 showed an SNV (frameshift) in the *B4GALT1* gene. Mutations in this gene have been described in almost 40 types of cancer in cBioPortal, including glioblastoma and glioblastoma multiforme. A few studies have proposed *B4GALT1* as a regulator of the SHH pathway [70,71,72].

Patient 22 (male) had a missense variant in the Jumonji C domain of the *KDM5D* gene (previously *JARID1D*) encoding lysine demethylase 5D [66]. Mutations in several other KDM gene family members have been described exclusively in group 3/4 MBs, implicating disruption of lysine demethylation in these tumors [73,74,75]. *KDM5D* is located on Yq11.223.

Only patient 43 had an SNV in a gene that is frequently mutated in group 3/4 MBs; *KDM6A* [18]. This was a truncating driver SNV with a second hit in the form of monosomy X (female patient).

SVs were identified and targeted in all patients. Three SVs were previously described drivers or potential drivers in MB: a *SNCAIP* tandem duplication and duplications/amplifications of chromosomes 5q and 7q in patients 1, 15, and 22, respectively. The *SNCAIP* variant is present in 17% of group 4 MBs making it the most frequent SV in this group [17,18]. The tandem duplication is a suggested disruptor of the chromatin environment, creating new interactions between the *SNCAIP* super-enhancer and other gene promoters in adjacent topologically associating domains leading to aberrant gene induction, a phenomenon also known as enhancer hijacking. Different kinds of duplications/amplifications of chromosomes 5q and 7q are seen in 31% and 54% of group 4 MBs, respectively [18].

Another four SVs detected in group 3/4 were not previously described in the literature. Patient 1 had a rearrangement between 11p and 17q, an associated loss of 11p, and several gains affecting 17q. Chromosome 11p aberrations, mainly deletions, and chromosome 17q aberrations, mainly duplications/amplifications, are reported in 32% and 58–86% of groups 3 and 4 MB, respectively [18].

Patient 15 showed a 19q12 tandem duplication involving the *ZNF536* gene, which is highly expressed in the brain and a proposed negative regulator of neuron differentiation [76]. Experimental siRNA silencing of *ZNF536* in breast cancer cell lines harboring 19q12 amplification has shown reduced cell viability compared to breast cancer cell lines lacking the amplification [77].

Patient 21 displayed an amplification of 10q22.2 encompassing exon 1 of the MANE transcript of *SAMD8* (alias *SMSr*) encoding a protein involved in sphingolipid metabolism that is highly expressed in brain tissue [78,79]. It has been reported to act as a suppressor of ceramide-mediated apoptosis [80], and it is possible that an amplification could enhance this anti-apoptotic effect.

Patient 43 had an Xp22.11 tandem duplication affecting the *KLHL15* gene, causing a frameshift after the first methionine residue. The same patient (female) had a CNA profile with monosomy X, and the tandem duplication was thus chosen as a marker for the remaining rearranged X chromosome in the tumor cells.

In summary, WGS identified a driving or recurrent event in MB in all except patient 21 and assays targeting ≥2 tumor sequences could be developed for all except patient 24. However, not all patients had both SNVs and SVs that could be targeted with ddPCR; some had few aberrations of either type (i.e., no SVs in the SHH group), and a few targets were located in complex regions making design difficult or impossible.

Liquid biopsy analysis showed that the approach of multiplexing SNVs and SVs is technically feasible and that our assays were highly sensitive, detecting as little as 3–5 hGEs in CSF and plasma, respectively. On d0, 83% (5/6) of CSF samples and 56% (5/9) of plasma samples were ctDNA positive. The only ctDNA negative CSF sample on d0 came from patient 19, whose tumor grew isolated from the CSF, and anatomical location is known to impact ctDNA detectability [31,33]. Within three weeks of surgery, 88% (7/8) of patients with localized disease, as well as the patient with the metastasized disease, were ctDNA positive in CSF. Overall, MRD was detected in 75% (9/12) of patients in the study.

MRD near d0 does not correlate with clinical outcome and is primarily of technical rather than clinical interest [41]. However, if pre/peri-operative CSF is positive, this indicates that the ctDNA marker may be used for the detection of MRD and/or relapse. If the pre/peri-operative sample is negative, the follow-up samples will also likely be negative. In the latter scenario, if metastases/relapse develop in contact with CSF, the marker may or may not be useful. In the study by Liu et al. 2021 MRD was investigated in CSF at baseline (d0) [41]. We sought to do the same in our study to explore ddPCR-based detectability of MB and to relate the results to the anatomical location (contact w CSF y/n) and stage (local/metastasized disease). cfDNA detection in plasma is generally of less interest than detection in CSF. However, it was not always possible to obtain CSF at diagnosis. In these cases, if the plasma sample was positive, this indicates that the ctDNA marker may be used for detection of MRD.

Sampling time points varied between patients as research samples could only be taken if a child underwent a clinical procedure and enough sample volume could be spared. Despite this limitation, our study supports the use of ctDNA in CSF as an MB MRD biomarker superior to cytology which was negative in all (n = 74) samples from all patients. For example, highly discordant results were seen in the d14 CSF sample from patient 1, with 253–328 hGEs/mL of each SV target, radiology supporting post-operative residual tumor tissue, but with negative cytology including synaptophysin staining. The elevated cell count and morphological inflammation in the sample did not impact our assays. Similarly, patient 15, with metastatic disease, had negative CSF cytology results both pre- and post-operatively (on d-3 and d5, respectively, and on another 26 occasions until he passed away, including two samples analyzed with both cytology and flow cytometry with negative results). In contrast, the patient was clearly ctDNA positive in CSF; the pre-operative sample taken on d0 for ctDNA analysis had >1000 hGEs of all three targets/mL CSF and the post-operative sample taken on d5 had 76–286 hGEs of targets/mL CSF. Unfortunately, no more than these two CSF samples were sent for ctDNA analysis.

The use of plasma negative controls for all liquid biopsies (plasma and CSF) can be considered a technical limitation; ideally, CSF negative control samples from age-matched patients without oncological disease would have been used. However, such samples were not available at the time of the experiments. This would have been an issue of greater importance if we had used a tumor agnostic approach.

An advantage of our approach is its applicability to a multitude of genetic aberrations: from point mutations to large genomic events. To our knowledge, this is the first study on multiplex ddPCR for both SNVs and SVs in CSF from patients with CNS tumors. A previous study using ddPCR for ctDNA MRD in MB in children with single plex SNV assays has shown the detectability of ctDNA at baseline in CSF but not in plasma [37]. It also showed feasibility for longitudinal analysis in CSF, although, in the follow-up setting, a multi-target approach is preferable. Multiplexing ddPCR for several SNVs is a possibility but might be a technically challenging one, as background signal from the wt assay complicates the interpretation of results. Multiplexing several SV assays, on the other hand, overrides this issue as they have little or no background.

SV targets may expand the utility of ddPCR analysis to track tumors that have no or few SNVs, harbor SNVs that do not allow for ddPCR assay design, or have SVs that are copy-number-neutral. While upfront design work is necessary, a turn-around-time (TAT) of approx. 3–4 weeks from delivery of WGS data to a ready-to-use assay is possible. Thus, analysis during and at the end of adjuvant therapy is feasible. ddPCR is a robust, easy-to-use, quantitative method that requires picogram levels of input DNA, no standard curves, little optimization, minimal bioinformatic processing of results, and has a technical TAT of one day once assays are in place. lcWGS, on the other hand, has the advantage of a streamlined experimental workflow and the ability to detect new clonal aberrations. However, it does not capture copy-number neutral aberrations or SNVs and may not have the resolution to detect ctDNA in CSF samples with increased cell counts or inflammation, causing increased levels of background cfDNA [41]. A combined approach of lcWGS and multiplex ddPCR could be complementary and should be explored in future studies on longitudinal samples taken at standardized time points.

## 5. Conclusions

WGS enabled the identification of driving or recurrent events in MB and informed the design of multiplexed ddPCR assays targeting SNVs and SVs simultaneously. Assays were highly sensitive, regardless of target aberration type, and SV assays had excellent specificity making multiplexing easy and interpretation straightforward. Using ddPCR, ctDNA could be detected in CSF in a majority of patients, outperforming the sensitivity of CSF cytology in this patient cohort. Detection was possible in low-volume samples (<1 mL) as well as in samples with elevated cell counts. In addition, ctDNA was detected in more than half of all pre-operative plasma samples. Although based on a limited number of patients, the results indicate the technical feasibility of multiplex ddPCR as an ultra-sensitive method to measure ctDNA-based MRD in MB. The approach should be evaluated at standardized time points in larger clinical series.

## Figures and Tables

**Table 1:** Participants’ characteristics.

UPN	Demographics		Clinical Features			Molecular Features			
	Age (Y)	Gender	Tumor Location	Size (cm)	Histology	Treatment Protocol	Mutations Somatic | Germline	CNAs	Subgroup | Methylation Subtype
1	14	M	4th ventricle	4	C	PNET5	—	—	¾ | VIII
9	10	F	4th ventricle	3.5 × 3 × 4	C	PNET5	*CTNNB1* | —	6-	WNT
13	7	F	4th ventricle	4.5 × 4.5 × 4	C	PNET5	*CTNNB1* | —	6-	WNT
15p	5	M	4th ventricle	4.5 × 4 × 4	C	PNET5	—	—	¾ | V
15 r/m	6	M	4th ventricle, base of scull, spinal cord	NA/0.4–0.6/0.7	C	MEMMAT	—	—	¾ | III
19p	4	F	LCH	6 × 5 × 3.8	C	PNET5	—	—	SHH | A
19r	5	F	LCH	0.4/0.6/0.2	C	TOTEM	—	—	SHH | A
21	4	M	4th ventricle	4.5 × 4 × 3.7	C	HIT Medguidance	—	—	¾ | VI
22	5	M	4th ventricle	4.5 × 5 × 4	C	PNET5	—	—	¾ | VI *
24	1	F	RCH	2.5	D/N	HIT-SKK	—	—	SHH | B
27	1	M	Vermis	4.5 × 3.5 × 4	D/N	HIT-SKK	— | *SUFU*	—	SHH | B
31	2	F	4th ventricle	3.5 × 3 × 3.5	N	HIT Medguidance	*SMO, KMT2D* | —	—	SHH | B
35	5	M	4th ventricle	2.4	C	PNET5	*CTNNB1* | —	—	WNT
43	15	F	4th ventricle	4 × 3.5 × 2.5	C	NA	*KDM6A* | —	—	¾ | NA

UPN, unique patient number; p, primary tumor; r, local relapse; m, metastasis; LCH, left cerebellar hemisphere; RCH, right cerebellar hemisphere; C, classical; D, desmoplastic; N, nodular; NA, data not available. Tumor location and size as denoted on diagnostic MRI. Clinical subgroup assigned according to the 2016 WHO Classification of Tumors of the CNS. Methylation subtype in capital letters/roman numerals based on methylation array data processed through DKFZ brain tumor methylation classifier with scores ≥ 0.9 (asterisk denotes a score 0.5–0.9).

**Table 2:** ddPCR assays and limit of detection on genomic tumor DNA.

UPN	Target	Target Position GRCh38/hg38	Amplicon Size	LoD	FPR
			(bp)	(pg)	Events/Well
1	der(11)t(11;17)(p11.2;q23.1)	chr11:46,349,051	98	100	0
	5q23.2 tandem duplication (*SNCAIP*)	chr5:122,518,910	100	100	0
9	*CTNNB1* c.100G>A, p.Gly34Arg *	chr3:41,224,612	62	100	0.17
	*CSNK2B* c.419G>T, p.Cys140Phe	chr6:31,669,370	64	100	0
	1p21.1 intergenic tandem duplication	chr1:105,150,201	89	100	0
13	*CTNNB1* c.101G>A, p.Gly34Glu *	chr3:41,224,613	64	100	0.58
	*PIK3CA* c.3140A>G, p.His1047Arg	chr3:179,234,297	80	100	0
15	*FOSL2* c.383G>A, p.Arg128His	chr2:28,408,787	62	10	0
	19q12 tandem duplication (*ZNF536*)	chr19:30,403,430	116	100	0
	5q13.3 tandem duplication (*HEXB*)	chr5:74,152,786	91	100	0
19	*MAX* c.179G>A, p.Arg60Gln	chr14:65,078,029	69	100	0.25
	*PTCH1* c.2287dup, p.Val763GlyfsTer27	chr9:95,467,388	63	100	0.25
21	*B4GALT1* c.421_428dup p.Glu144CysfsTer2	chr9:33,135,408	64	10	0
	10q22.2 amp (*SAMD8*)	chr10:73,926,074	87	10	0
22	*KDM5D* c.1642G>C, p.Asp548His	chrY:19,720,946	64	100	0
	7q21.2 amp (*CDK6*)	chr7:92,501,482	92	10	0
24	*PRMT7* c.224C>T, p.Thr75Met	chr16:68,324,774	62	10	0.5
27	*KMT2D* c.13825C>T, p.Gln4609Ter	chr12:49,030,615	98	100	0.6
	*OFD1* c.1411+1G>A	chrX:13,756,768	65	10	0.25
31	*KMT2D* c.7933C>T, p.Arg2645Ter	chr12:49,039,837	68	100	0.75
	*SMO* c.1247_1248delinsAA, p.Gly416Glu *	chr7:129,206,570	61	100	1.58
35	*CTNNB1*, c.101G>A, p.Gly34Glu *	chr3:41,224,613	64	100	0.17
	13q14.2 tandem duplication (*CAB39L*)	chr13:49,304,384	112	100	0
43	*KDM6A* c.4129C>T, p.Gln1377Ter *	chrX:45,110,202	68	100	0.08
	Xp22.11 tandem duplication (*KLHL15*)	chrX:24,005,865	90	100	0

Target sequences used for final ddPCR assays. Point mutations detected with clinical Sanger sequencing marked with an asterisk. UNP, unique patient number; GRCh38, Genome Reference Consortium Human Build 38; LoD, limit of detection; FPR, false positive rate; amp, amplification.

## Data Availability

The experimental results from the study are included in the manuscript or in the supplementary information. The raw data generated by WGS/methylation array analysis are not publicly available due to patients’ privacy. The analyzed datasets that support the findings presented in this study are available on request from the corresponding author.

## Supplementary Materials

The following supporting information can be downloaded at: https://www.mdpi.com/article/10.3390/cancers15071972/s1, Figure S1: Copy-number profiles, ploidy and tumor cell ratios generated by Control-FREEC, ASCAT and DKFZ brain tumor methylation classifier; File S1: QX Manager Software output data on dilution series; File S2: QX Manager Software output data on liquid biopsies; Table S1: Summary of results from DKFZ brain tumor methylation classifier; Table S2: Table of all tested ddPCR assays; Table S3: Summary of ctDNA results.
