# Supplementary material for: Simultaneous Ultra-Sensitive Detection of Structural and Single Nucleotide Variants Using Multiplex Droplet Digital PCR in Liquid Biopsies from Children with Medulloblastoma
Source: Cancers (Basel). 2023 Mar 25;15(7):1972. doi: 10.3390/cancers15071972 (PMC10092983; doi:10.3390/cancers15071972)
Supplement: Supplementary file 1 [file cancers-15-01972-s001.zip › Figure S1 Copy-number profiles, ploidy and tumor cell ratios generated by Control-FREEC, ASCAT and DKFZ brain tumor methylation classifier.pdf]

MB1

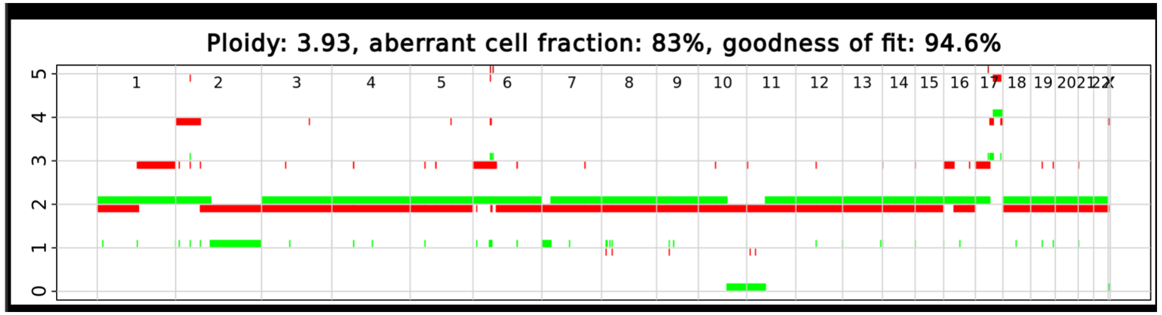
Ploidy: 3.93, aberrant cell fraction: 83%, goodness of fit: 94.6%

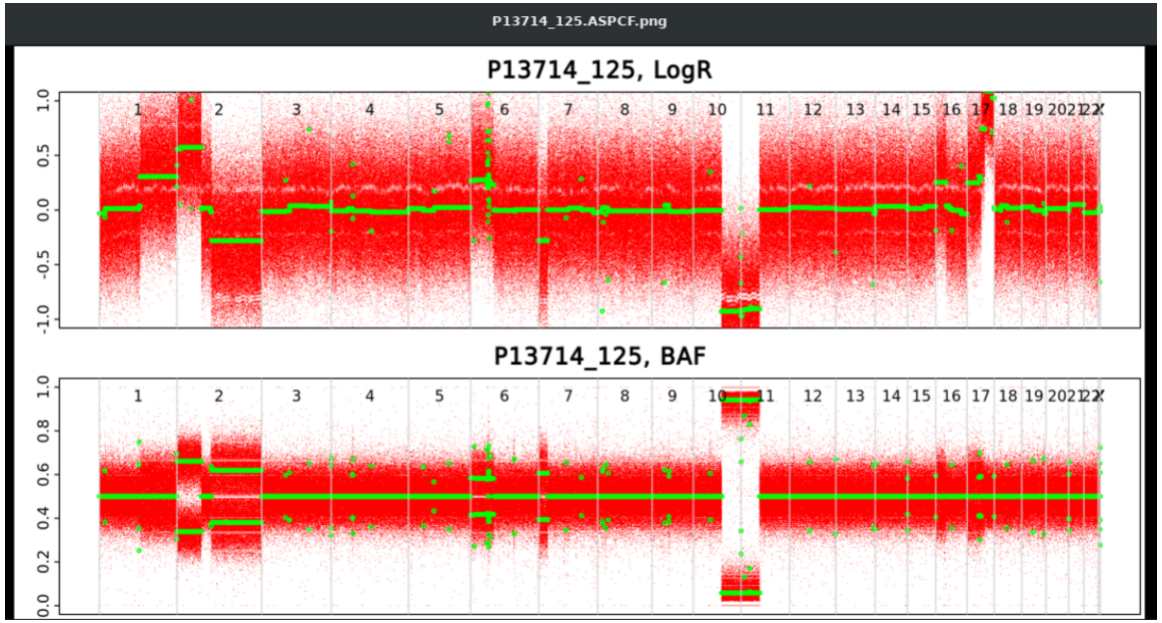
P13714_125.ASPCF.png
P13714_125, LogR
P13714_125, BAF

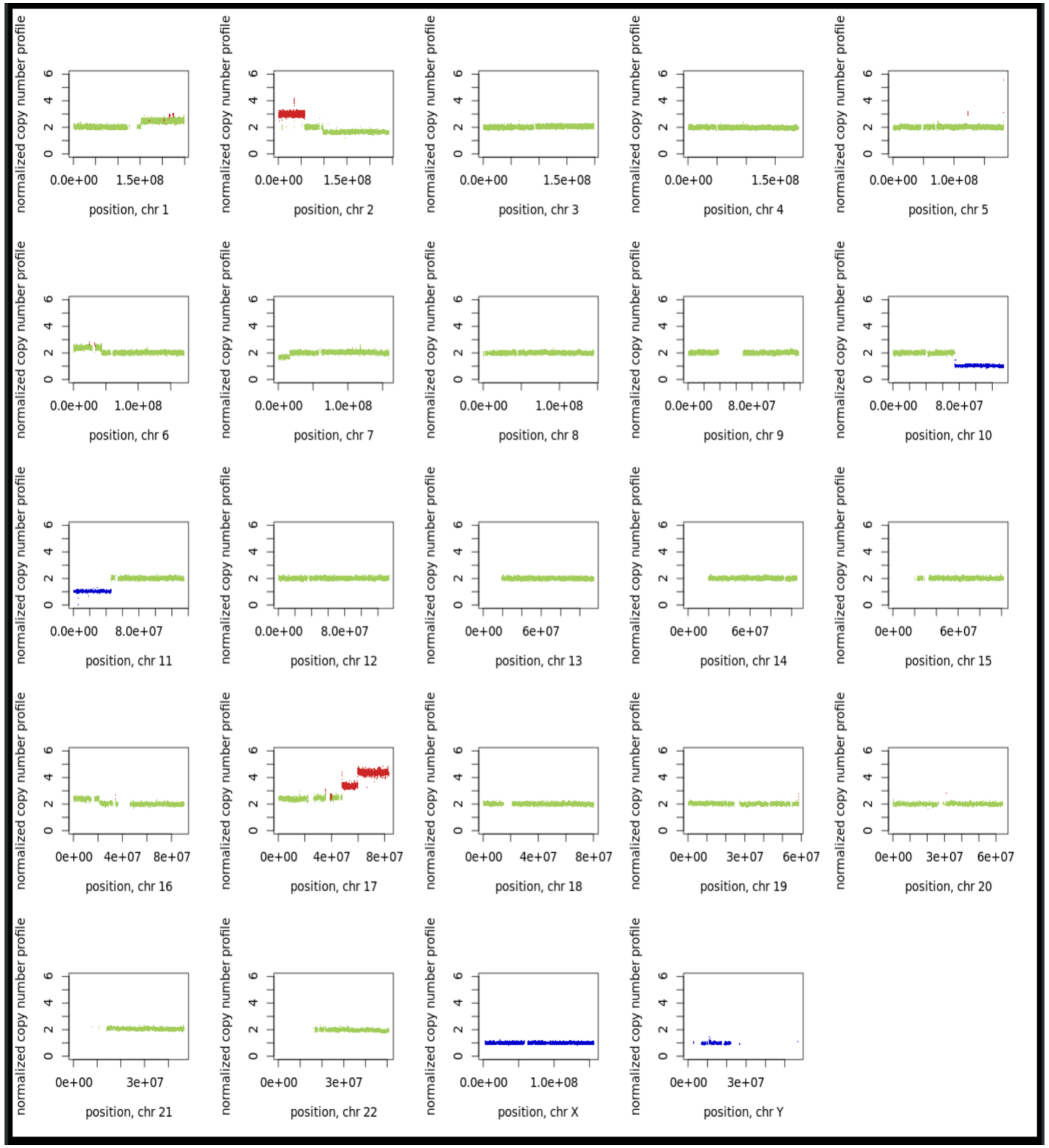
normalized copy number profile
position, chr 1
position, chr 2
position, chr 3
position, chr 4
position, chr 5
position, chr 6
position, chr 7
position, chr 8
position, chr 9
position, chr 10
position, chr 11
position, chr 12
position, chr 13
position, chr 14
position, chr 15
position, chr 16
position, chr 17
position, chr 18
position, chr 19
position, chr 20
position, chr 21
position, chr 22
position, chr X
position, chr Y

MB1

Copy number variation profile

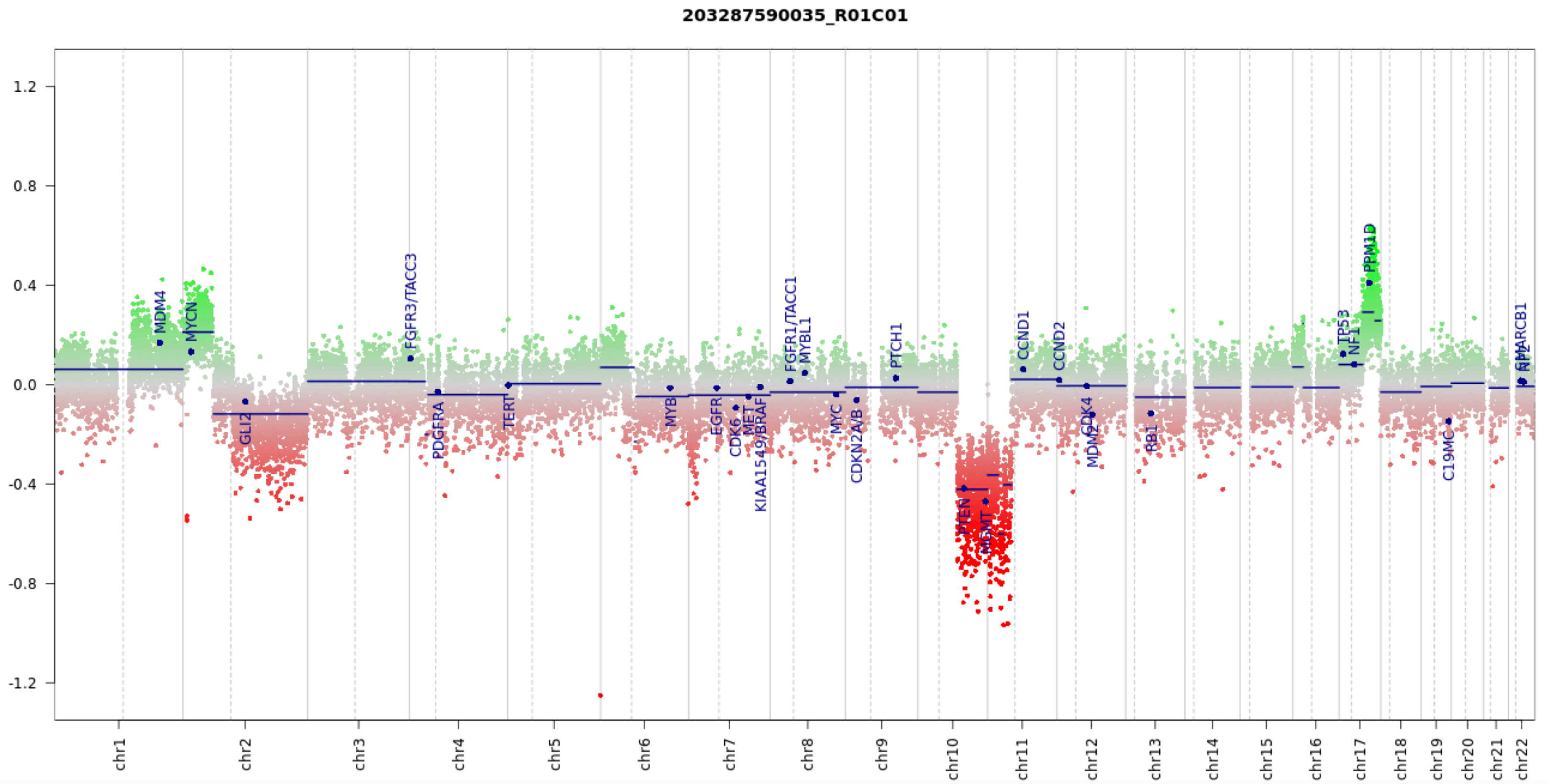

MB9

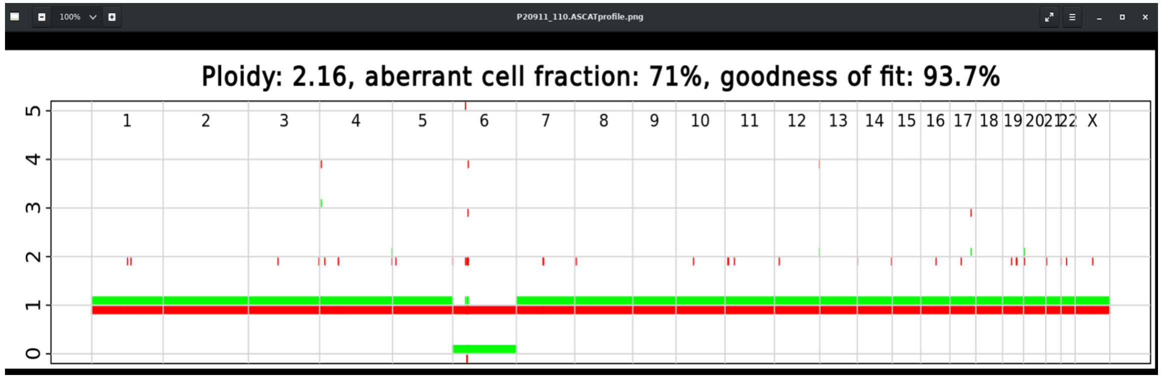
P20911_110.ASCATprofile.png
Ploidy: 2.16, aberrant cell fraction: 71%, goodness of fit: 93.7%

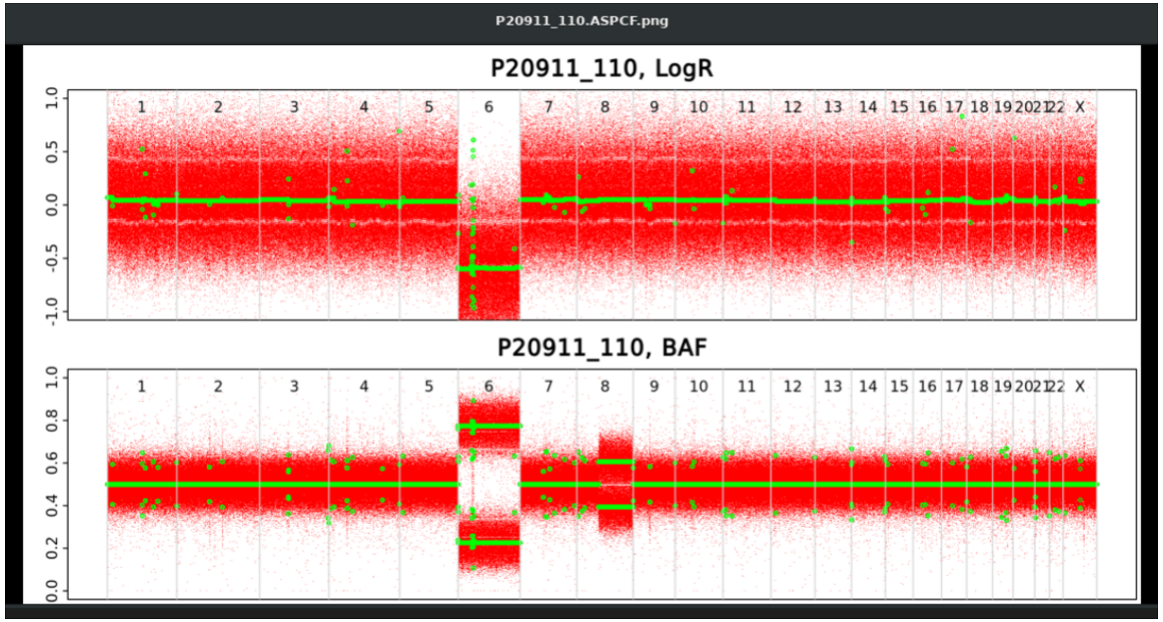
P20911_110.ASPCF.png
P20911_110, LogR
P20911_110, BAF

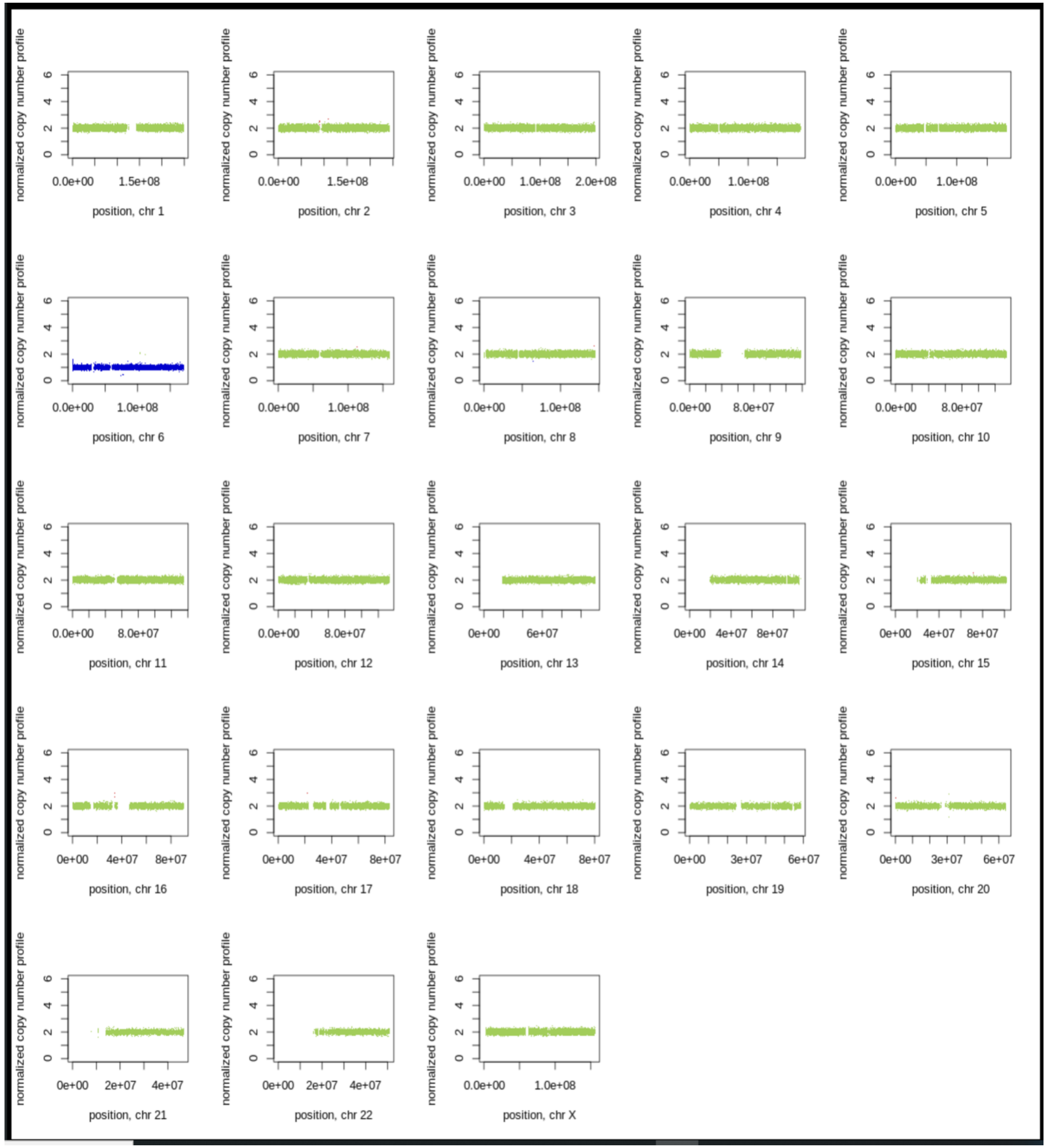
normalized copy number profile
position, chr 1
position, chr 2
position, chr 3
position, chr 4
position, chr 5
position, chr 6
position, chr 7
position, chr 8
position, chr 9
position, chr 10
position, chr 11
position, chr 12
position, chr 13
position, chr 14
position, chr 15
position, chr 16
position, chr 17
position, chr 18
position, chr 19
position, chr 20
position, chr 21
position, chr 22
position, chr X

MB9

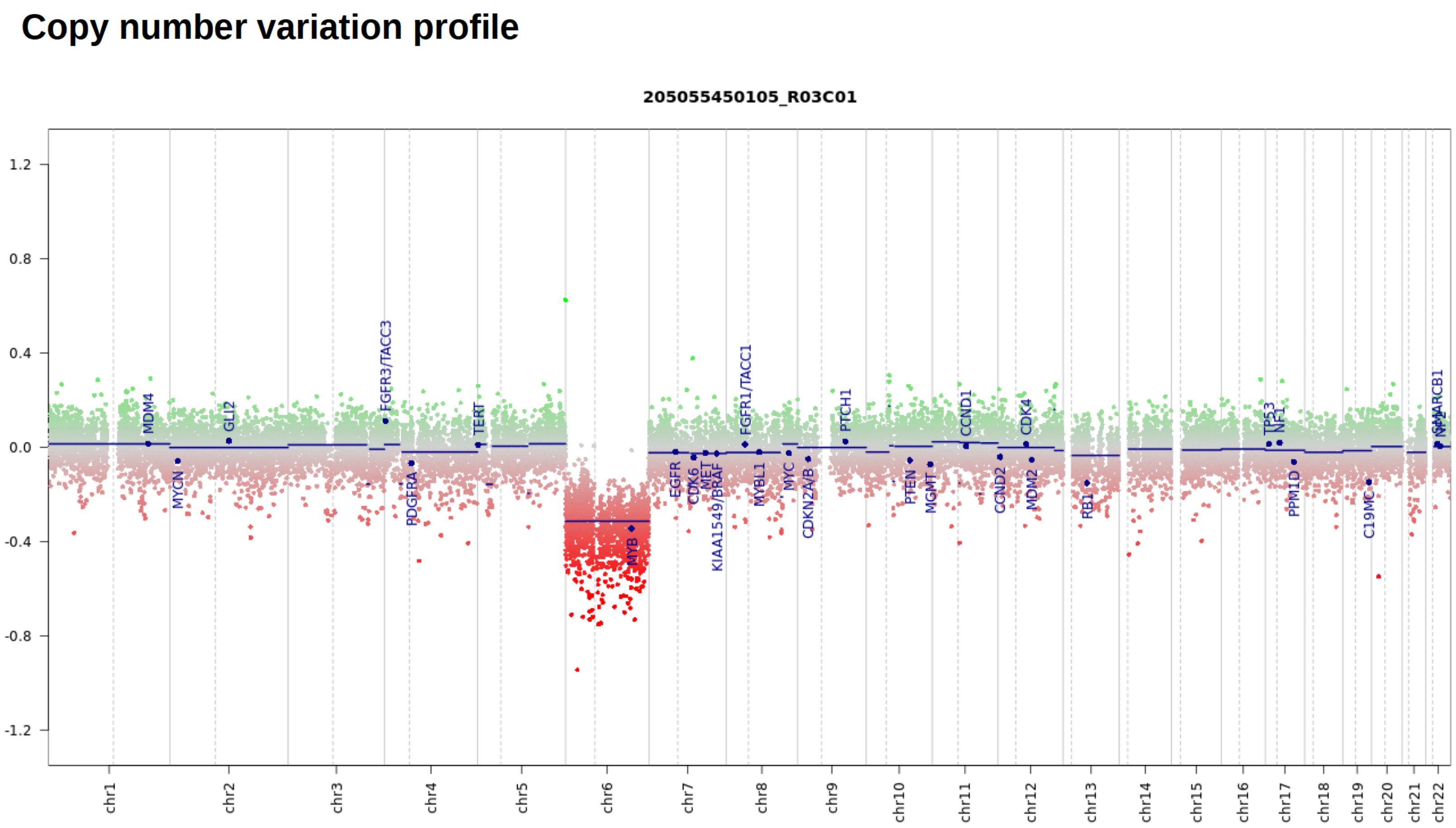
Copy number variation profile
205055450105_R03C01
1.2
0.8
0.4
0.0
-0.4
-0.8
-1.2
MDM4
MYCN
GLI2
FGFR3/TACC3
PDGFRA
TERT
MYB
EGFR
CDK6
MET
KIAA1549/BRAF
FGFR1/TACC1
MYBL1
MYC
CDKN2A/B
PTCH1
PTEN
MGMT
CCND1
CCND2
CDK4
MDM2
RB1
TP53
NF1
PPM1D
C19MC
SMARCB1
chr1
chr2
chr3
chr4
chr5
chr6
chr7
chr8
chr9
chr10
chr11
chr12
chr13
chr14
chr15
chr16
chr17
chr18
chr19
chr20
chr21
chr22

MB13

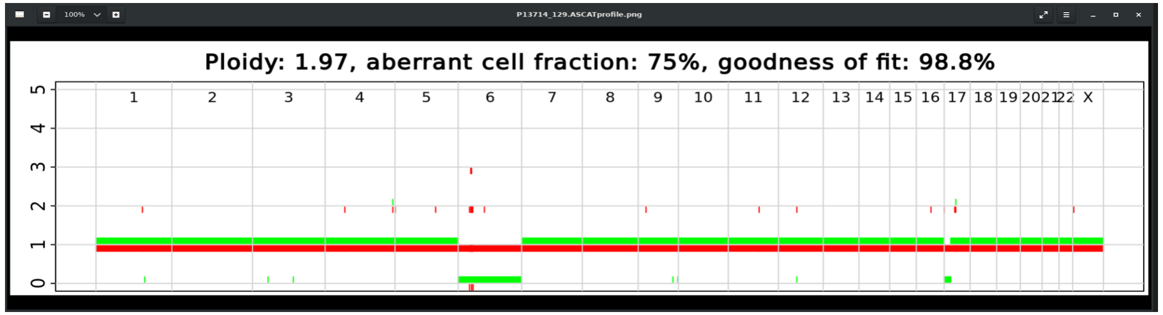
P13714_129.ASCATprofile.png
Ploidy: 1.97, aberrant cell fraction: 75%, goodness of fit: 98.8%
1 2 3 4 5 6 7 8 9 10 11 12 13 14 15 16 17 18 19 20 21 22 X

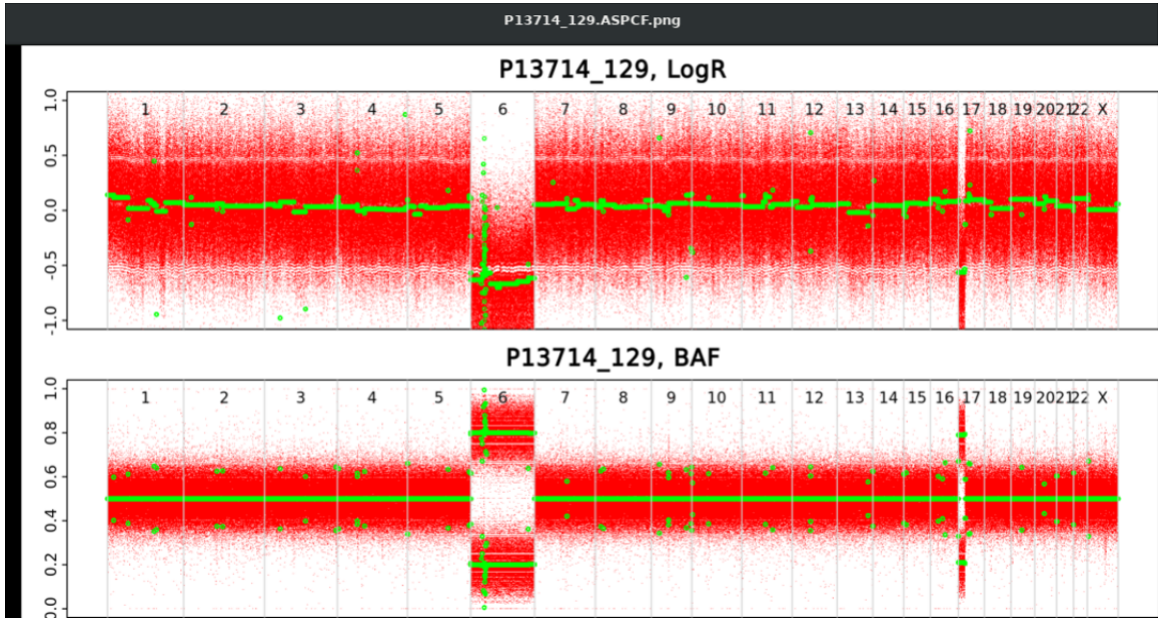
P13714_129.ASPCF.png
P13714_129, LogR
P13714_129, BAF
1 2 3 4 5 6 7 8 9 10 11 12 13 14 15 16 17 18 19 20 21 22 X

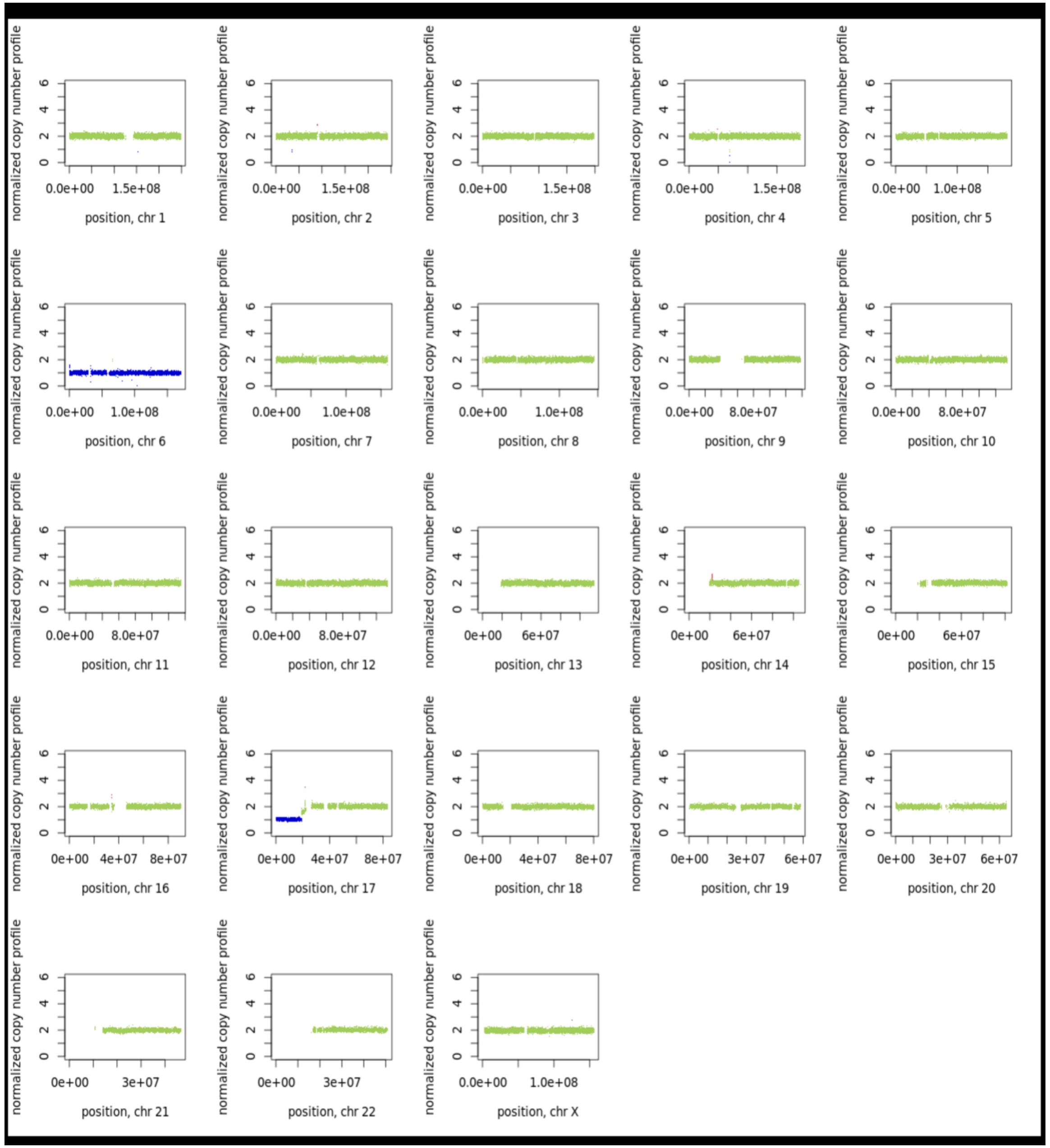
normalized copy number profile
position, chr 1
position, chr 2
position, chr 3
position, chr 4
position, chr 5
position, chr 6
position, chr 7
position, chr 8
position, chr 9
position, chr 10
position, chr 11
position, chr 12
position, chr 13
position, chr 14
position, chr 15
position, chr 16
position, chr 17
position, chr 18
position, chr 19
position, chr 20
position, chr 21
position, chr 22
position, chr X

MB13

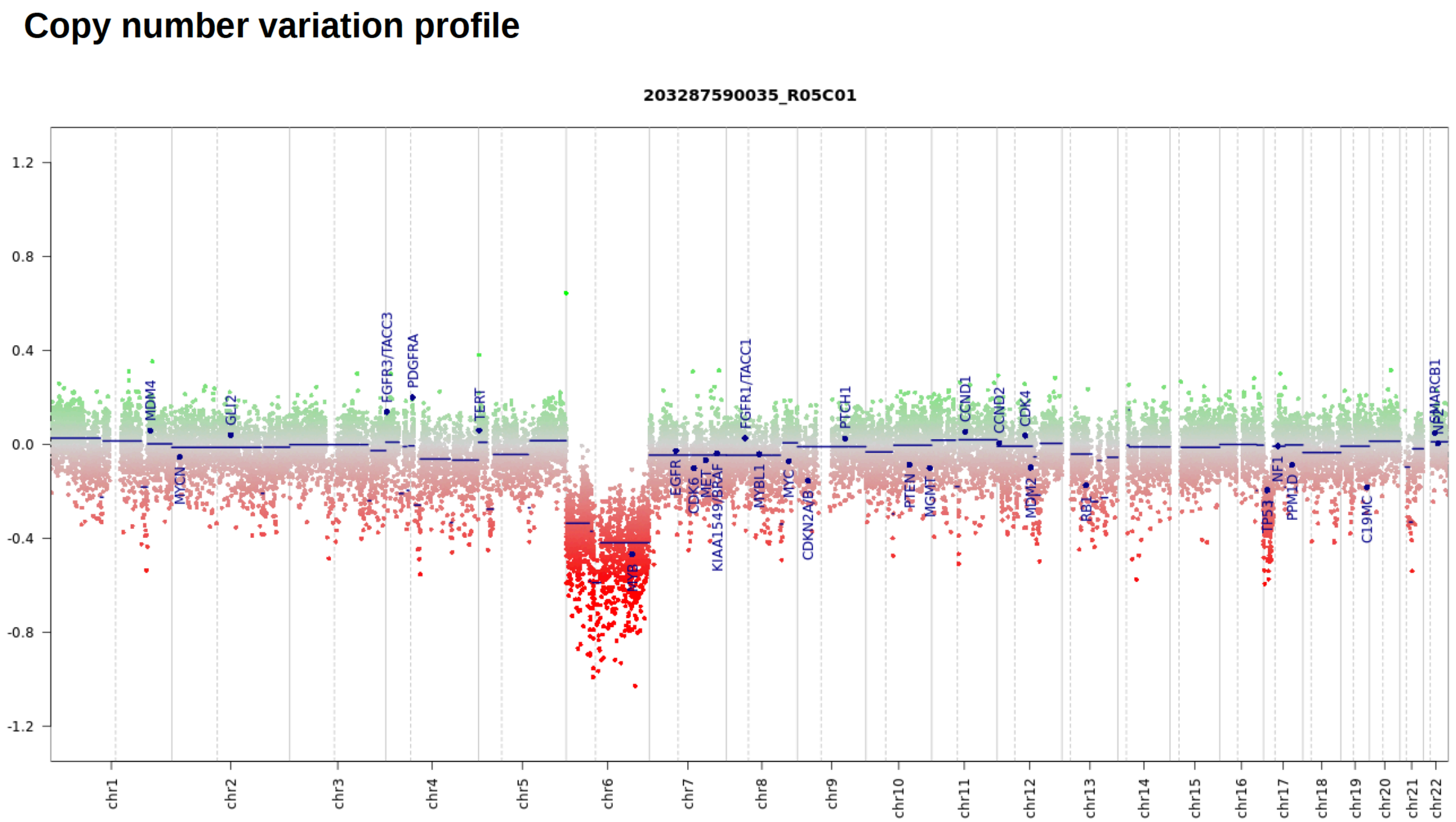

Copy number variation profile
203287590035_R05C01
1.2
0.8
0.4
0.0
-0.4
-0.8
-1.2
MDM4
MYCN
GLI2
FGFR3/TACC3
PDGFRA
TERT
EGFR
CDK6
MET
KIAA1549/BRAF
FGFR1/TACC1
MYBL1
MYC
CDKN2A/B
PTCH1
PTEN
MGMT
CCND1
CCND2
CDK4
MDM2
RB1
TP53
NF1
PPM1D
C19MC
SMARCB1
NF2
chr1
chr2
chr3
chr4
chr5
chr6
chr7
chr8
chr9
chr10
chr11
chr12
chr13
chr14
chr15
chr16
chr17
chr18
chr19
chr20
chr21
chr22

MB15p

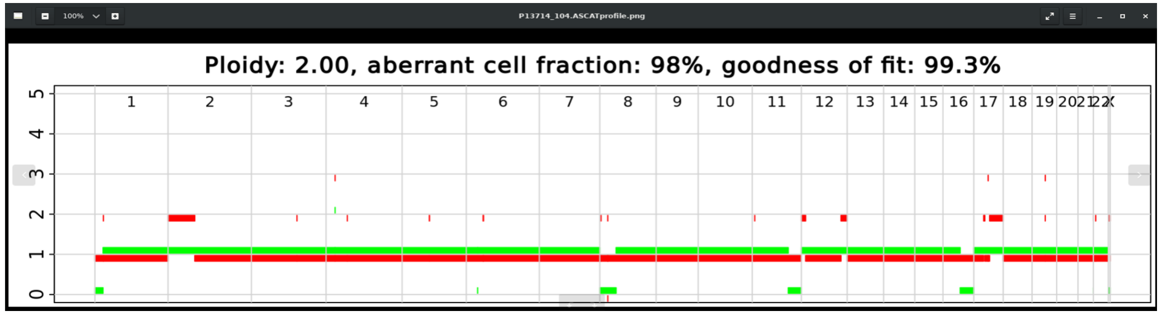
P13714_104.ASCATprofile.png
Ploidy: 2.00, aberrant cell fraction: 98%, goodness of fit: 99.3%
1 2 3 4 5 6 7 8 9 10 11 12 13 14 15 16 17 18 19 20 21 22 X

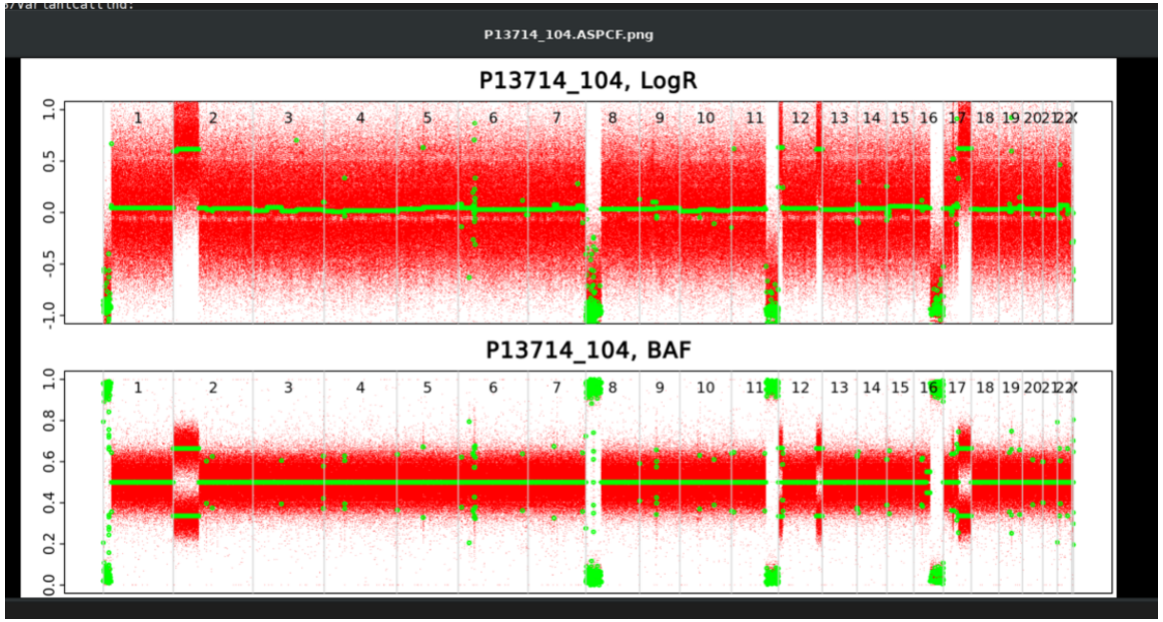
P13714_104.ASPCF.png
P13714_104, LogR
P13714_104, BAF

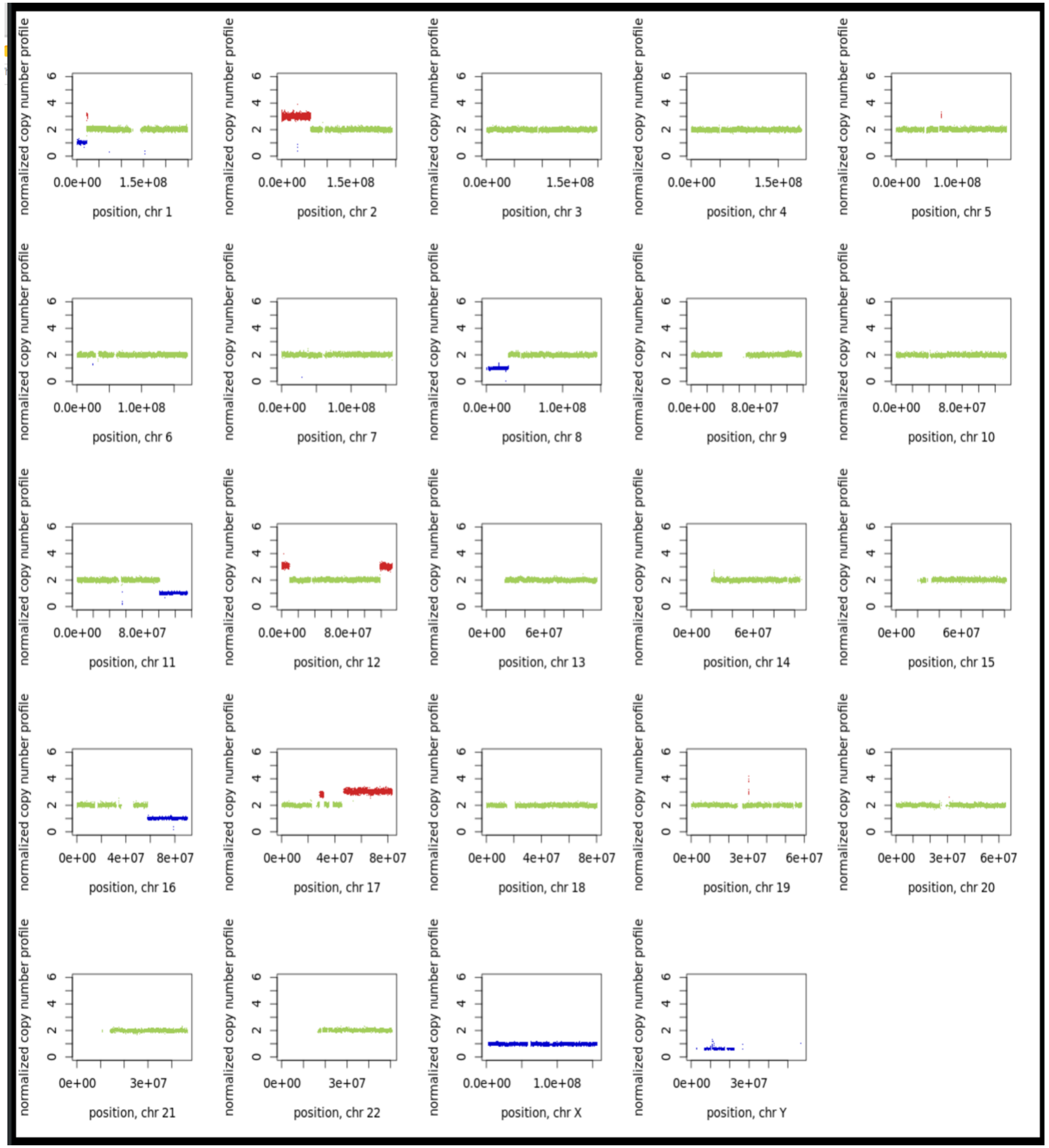
normalized copy number profile
position, chr 1
position, chr 2
position, chr 3
position, chr 4
position, chr 5
position, chr 6
position, chr 7
position, chr 8
position, chr 9
position, chr 10
position, chr 11
position, chr 12
position, chr 13
position, chr 14
position, chr 15
position, chr 16
position, chr 17
position, chr 18
position, chr 19
position, chr 20
position, chr 21
position, chr 22
position, chr X
position, chr Y

MB15p

Copy number variation profile

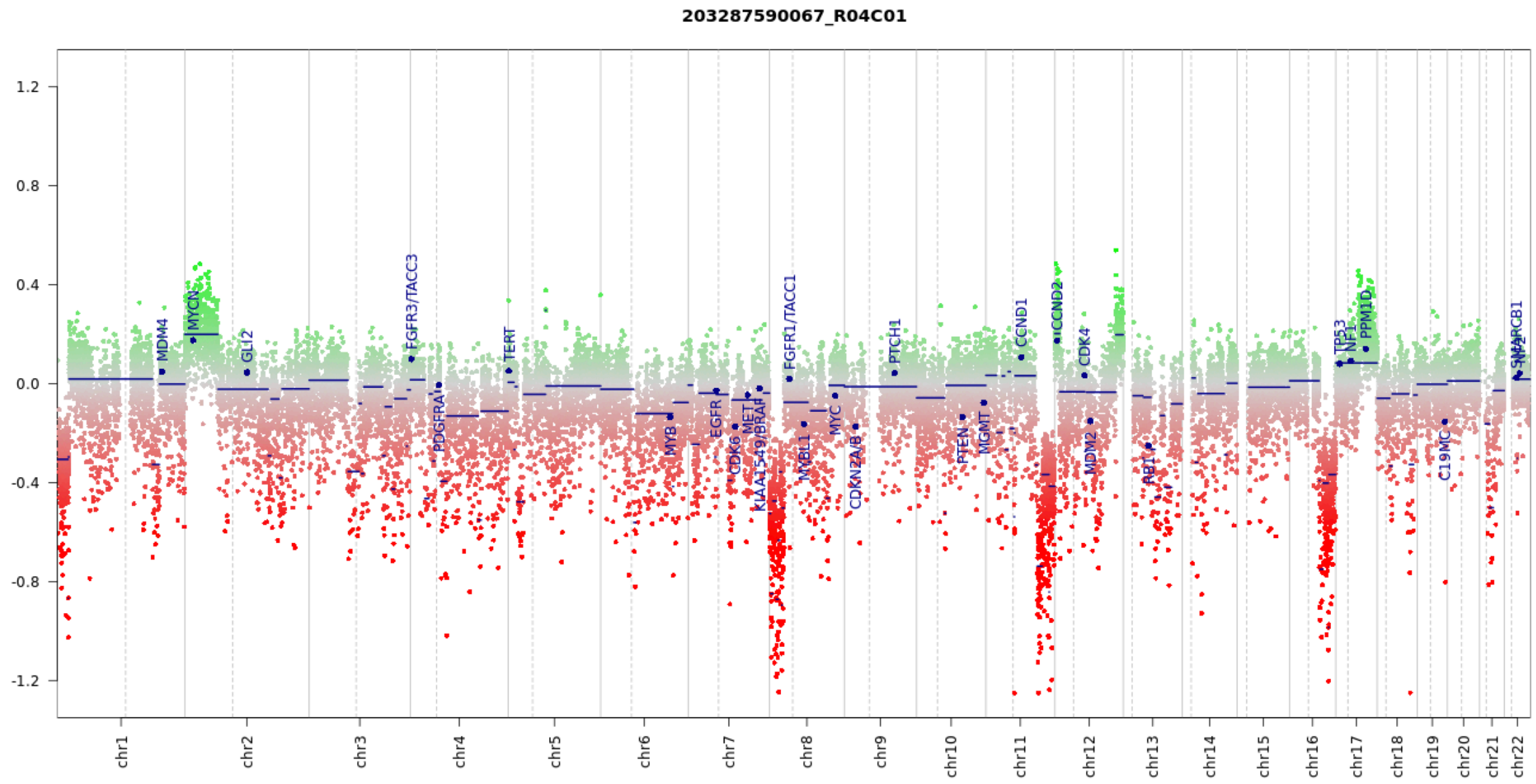

MB15r/m

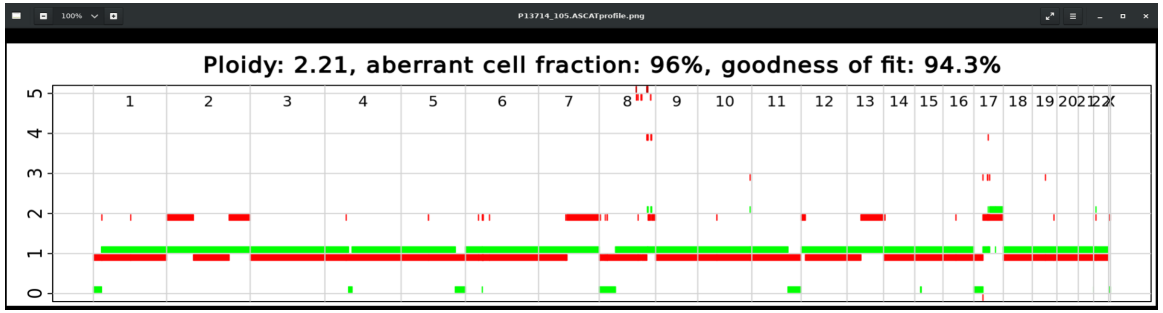
P13714_105.ASCATprofile.png
Ploidy: 2.21, aberrant cell fraction: 96%, goodness of fit: 94.3%

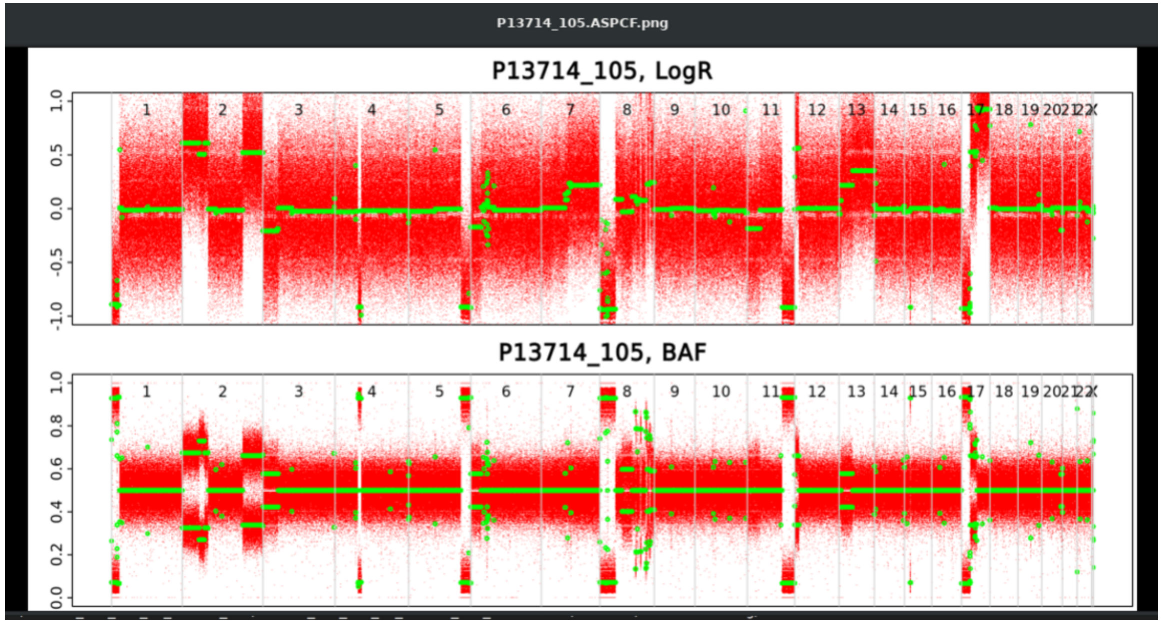
P13714_105.ASPCF.png
P13714_105, LogR
P13714_105, BAF

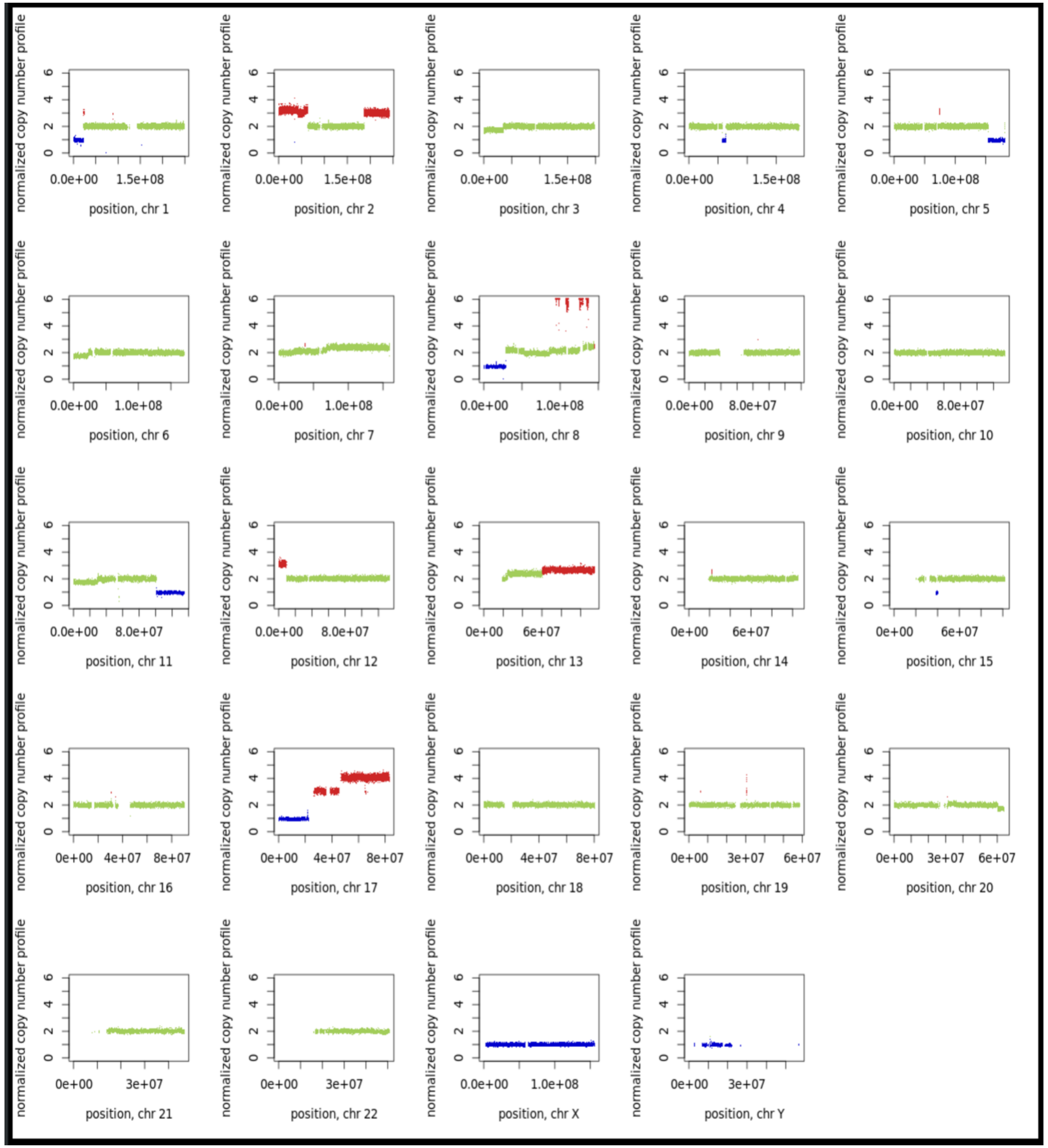
normalized copy number profile
position, chr 1
position, chr 2
position, chr 3
position, chr 4
position, chr 5
position, chr 6
position, chr 7
position, chr 8
position, chr 9
position, chr 10
position, chr 11
position, chr 12
position, chr 13
position, chr 14
position, chr 15
position, chr 16
position, chr 17
position, chr 18
position, chr 19
position, chr 20
position, chr 21
position, chr 22
position, chr X
position, chr Y

15r/m

Copy number variation profile

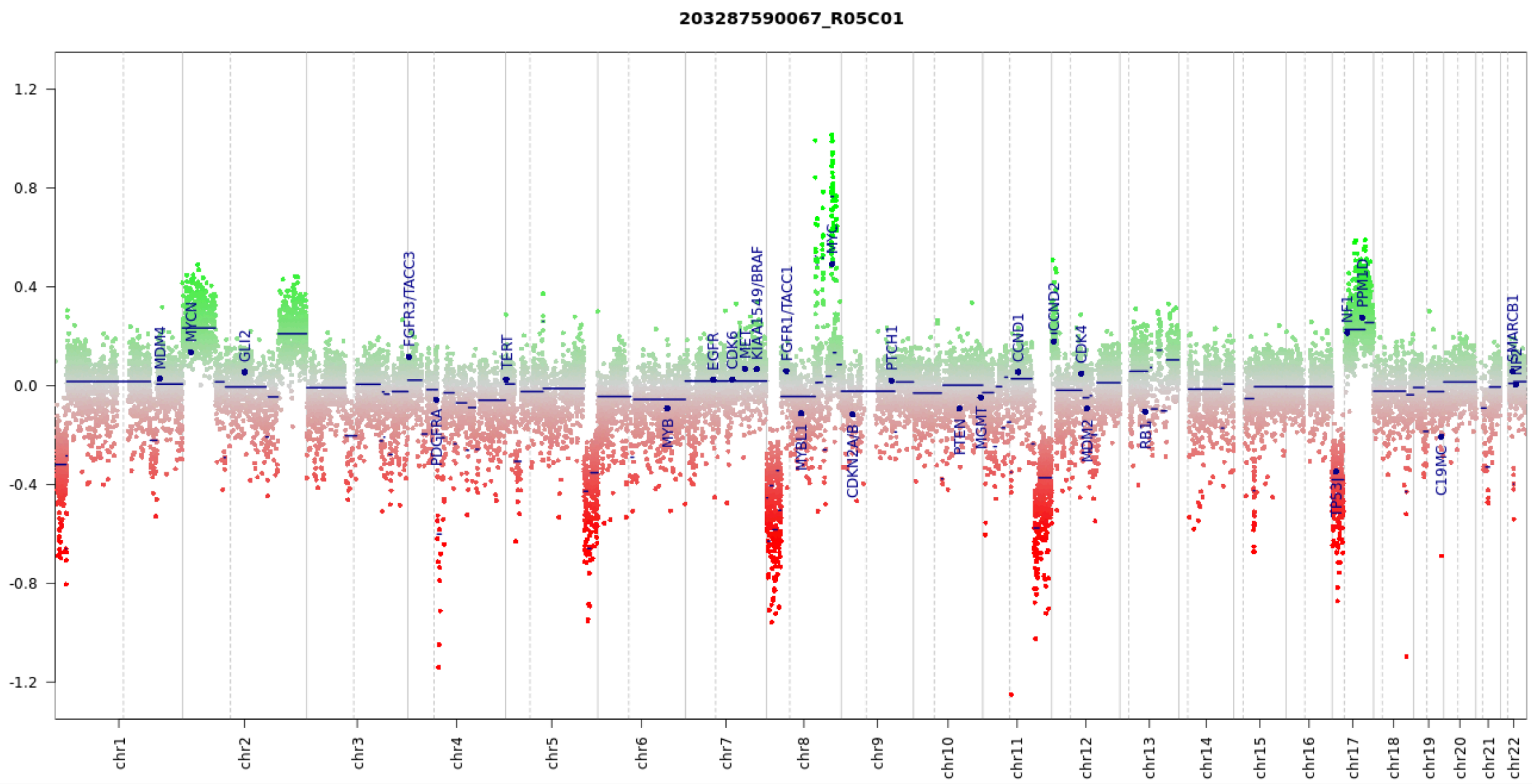

MB19p

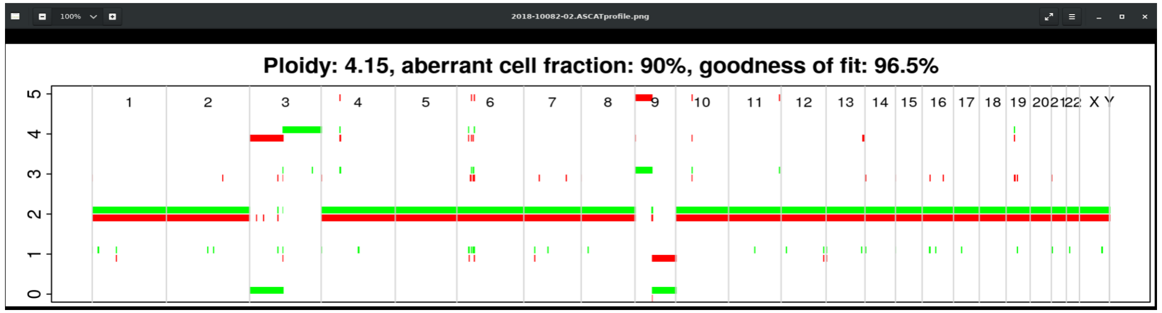
2018-10082-02.ASCATprofile.png
Ploidy: 4.15, aberrant cell fraction: 90%, goodness of fit: 96.5%

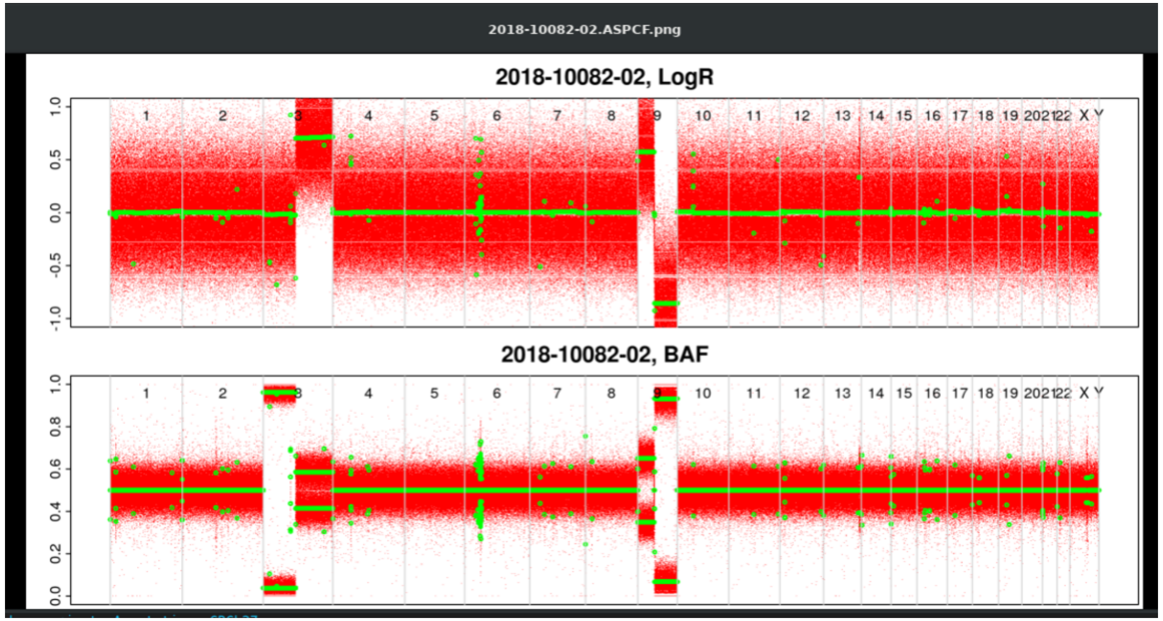
2018-10082-02.ASPCF.png
2018-10082-02, LogR
2018-10082-02, BAF

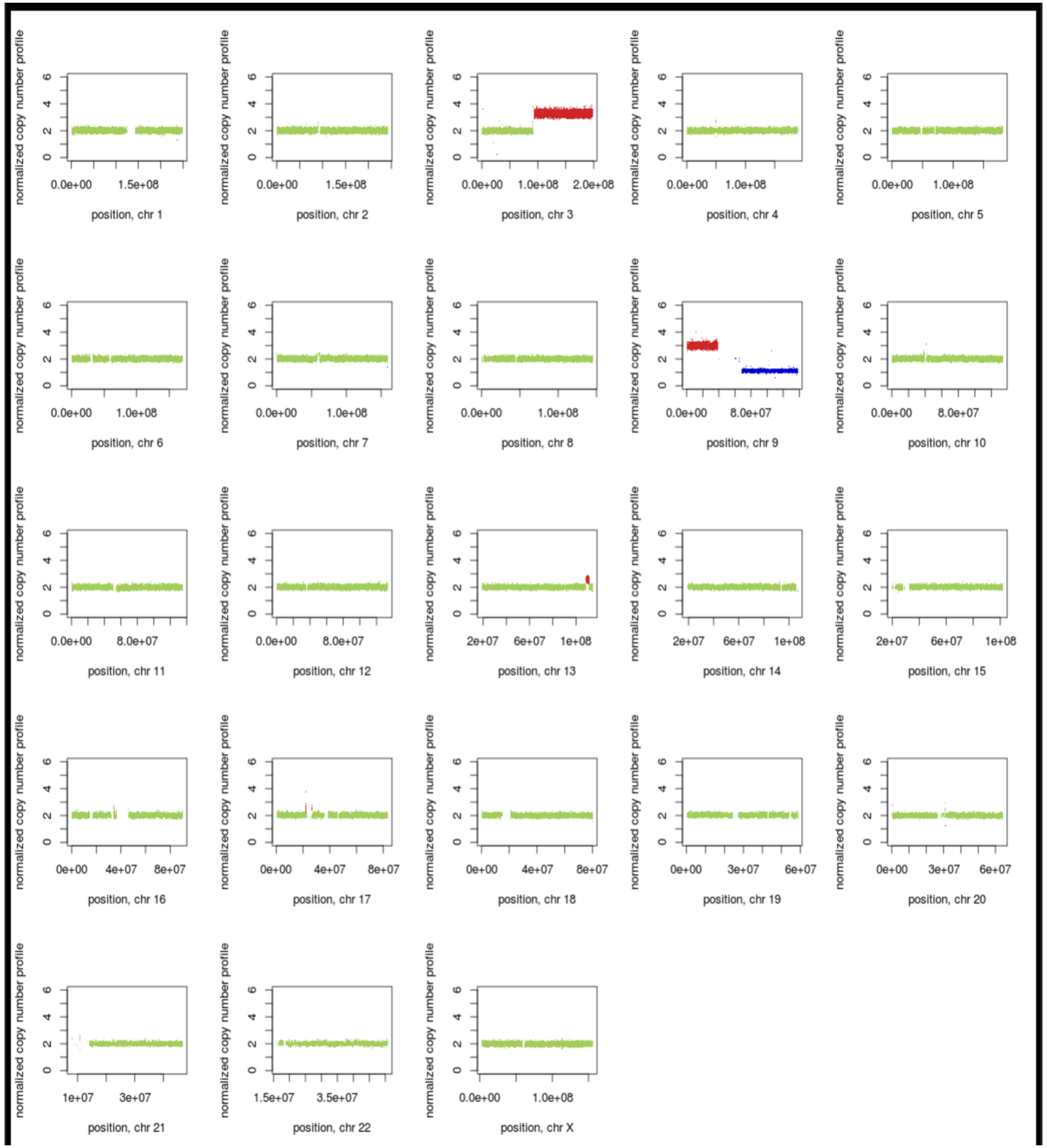
normalized copy number profile
position, chr 1
position, chr 2
position, chr 3
position, chr 4
position, chr 5
position, chr 6
position, chr 7
position, chr 8
position, chr 9
position, chr 10
position, chr 11
position, chr 12
position, chr 13
position, chr 14
position, chr 15
position, chr 16
position, chr 17
position, chr 18
position, chr 19
position, chr 20
position, chr 21
position, chr 22
position, chr X

MB19p

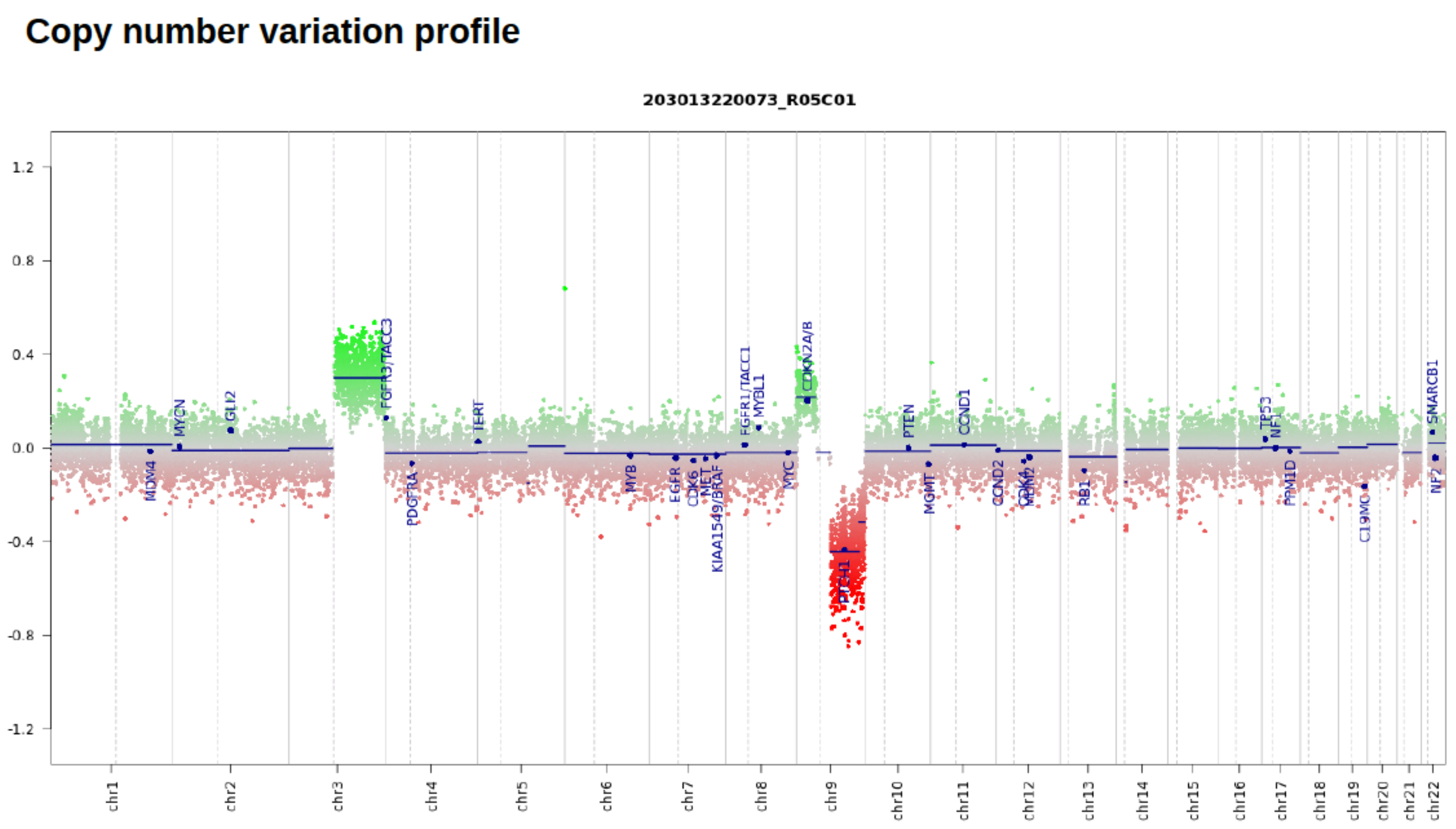
Copy number variation profile
203013220073_R05C01
1.2
0.8
0.4
0.0
-0.4
-0.8
-1.2
MDM4
MYCN
GLI2
FGFR3/TACC3
PDGFRA
TERT
MYB
EGFR
CDK6
MET
KIAA1549/BRAF
FGFR1/TACC1
MYBL1
MYC
CDKN2A/B
PTCH1
PTEN
MGMT
CCND1
CCND2
CDK4
MDM2
RB1
TP53
NF1
PPM1D
C19MC
SMARCB1
NF2
chr1
chr2
chr3
chr4
chr5
chr6
chr7
chr8
chr9
chr10
chr11
chr12
chr13
chr14
chr15
chr16
chr17
chr18
chr19
chr20
chr21
chr22

MB19r

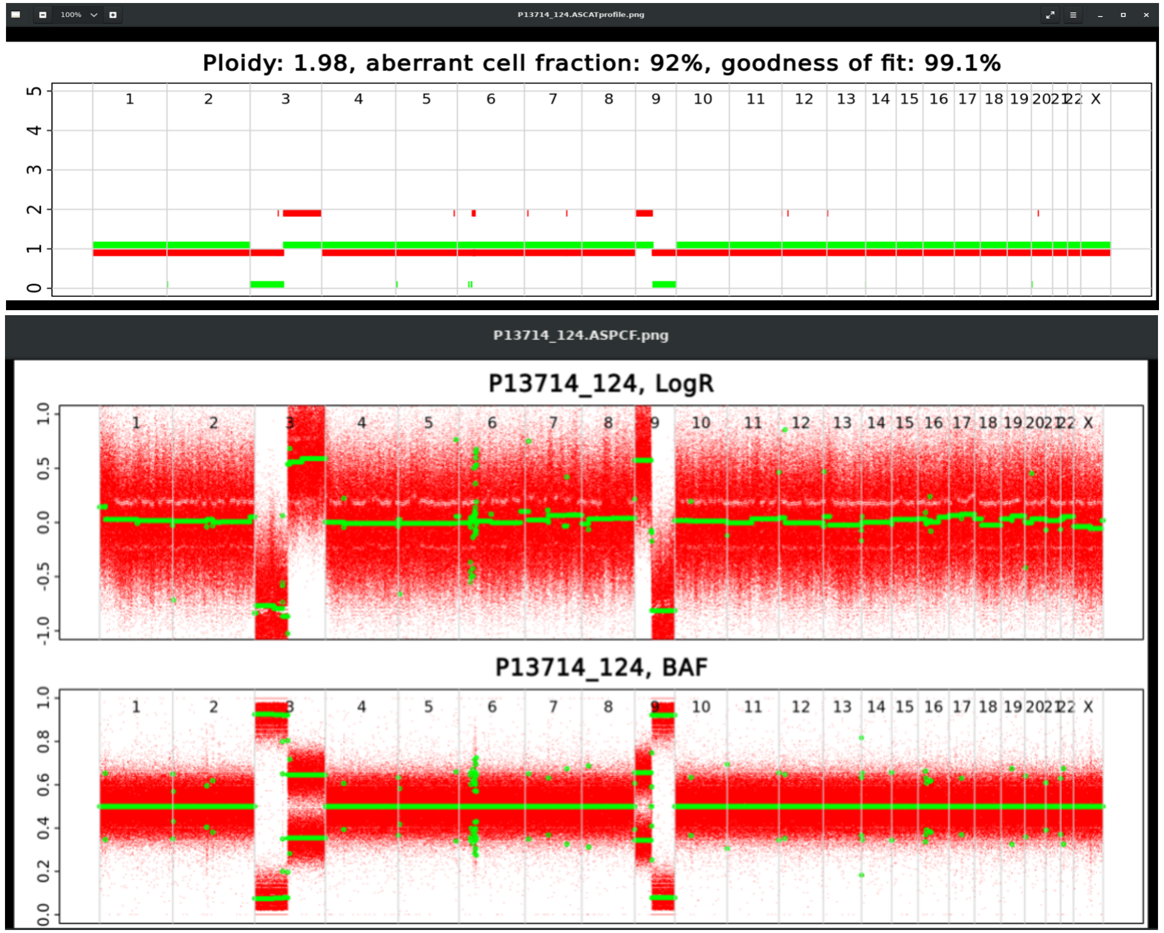
P13714_124.ASCATprofile.png
Ploidy: 1.98, aberrant cell fraction: 92%, goodness of fit: 99.1%
P13714_124.ASPCF.png
P13714_124, LogR
P13714_124, BAF

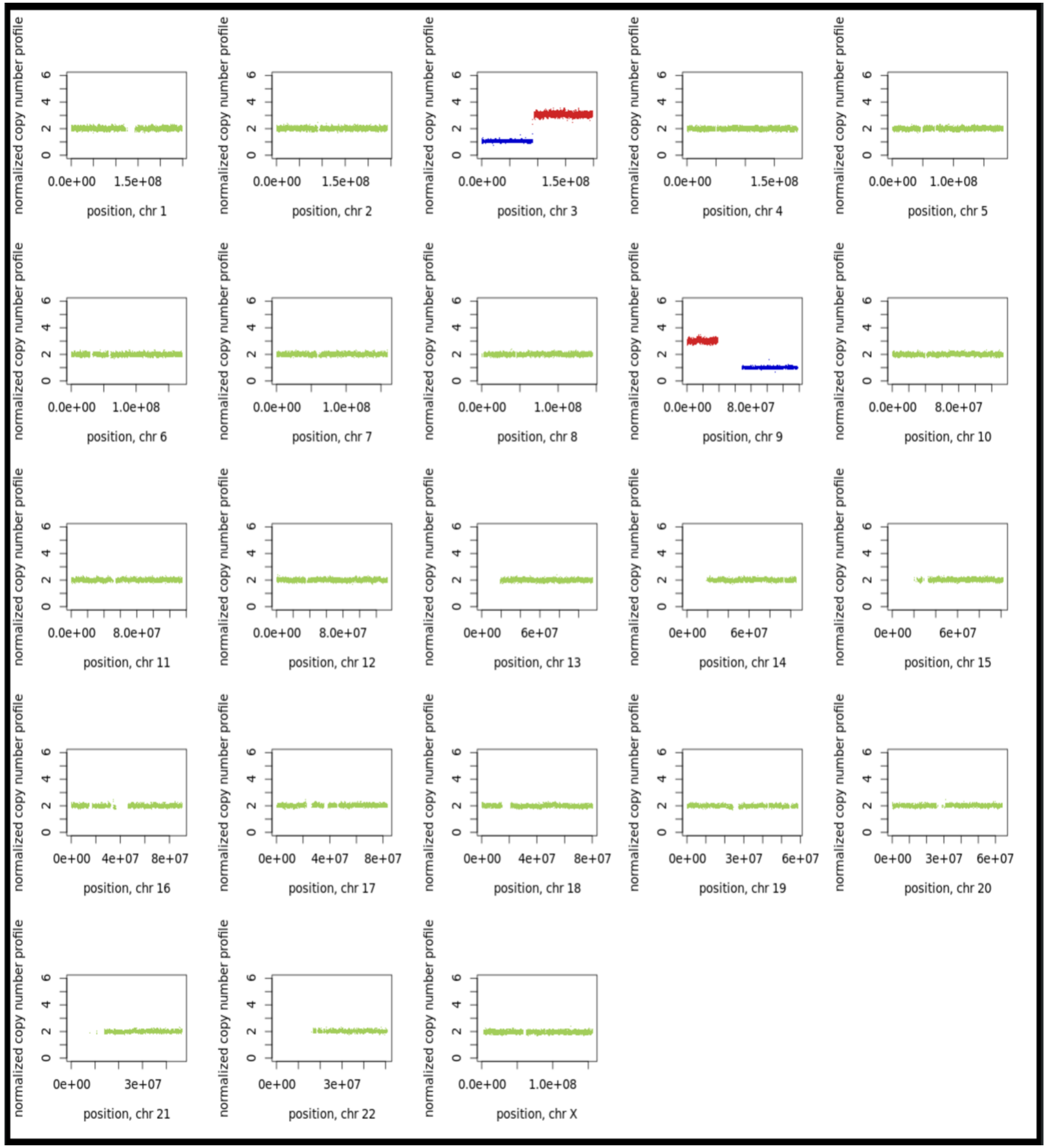
normalized copy number profile
position, chr 1
position, chr 2
position, chr 3
position, chr 4
position, chr 5
position, chr 6
position, chr 7
position, chr 8
position, chr 9
position, chr 10
position, chr 11
position, chr 12
position, chr 13
position, chr 14
position, chr 15
position, chr 16
position, chr 17
position, chr 18
position, chr 19
position, chr 20
position, chr 21
position, chr 22
position, chr X

MB19r

Copy number variation profile

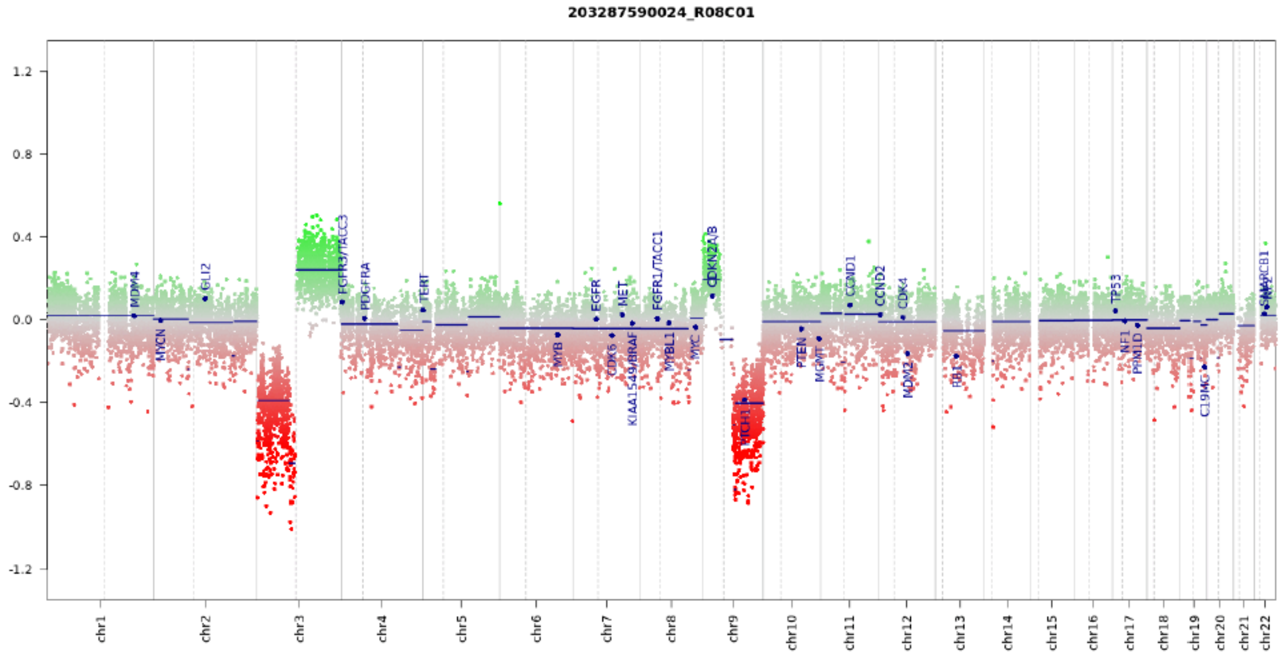

MB21

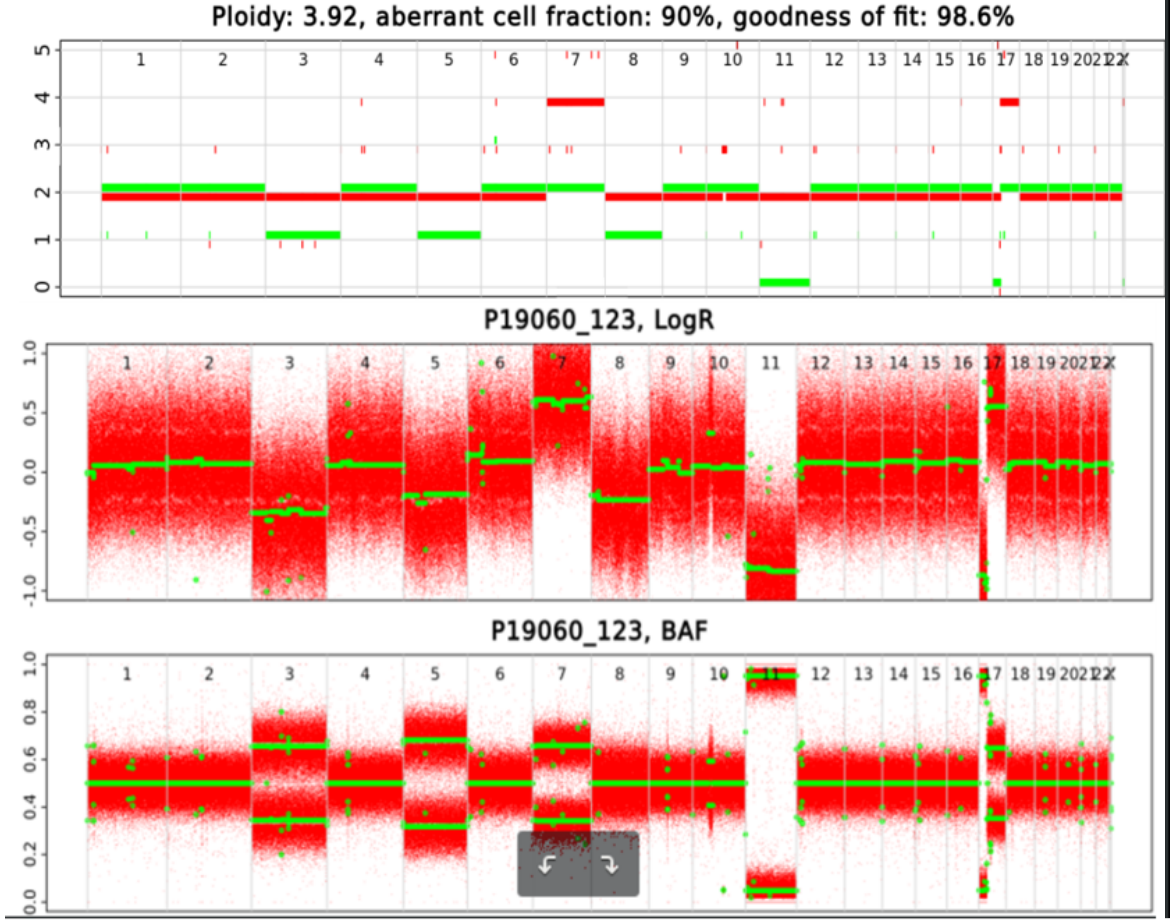
Ploidy: 3.92, aberrant cell fraction: 90%, goodness of fit: 98.6%
P19060_123, LogR
P19060_123, BAF

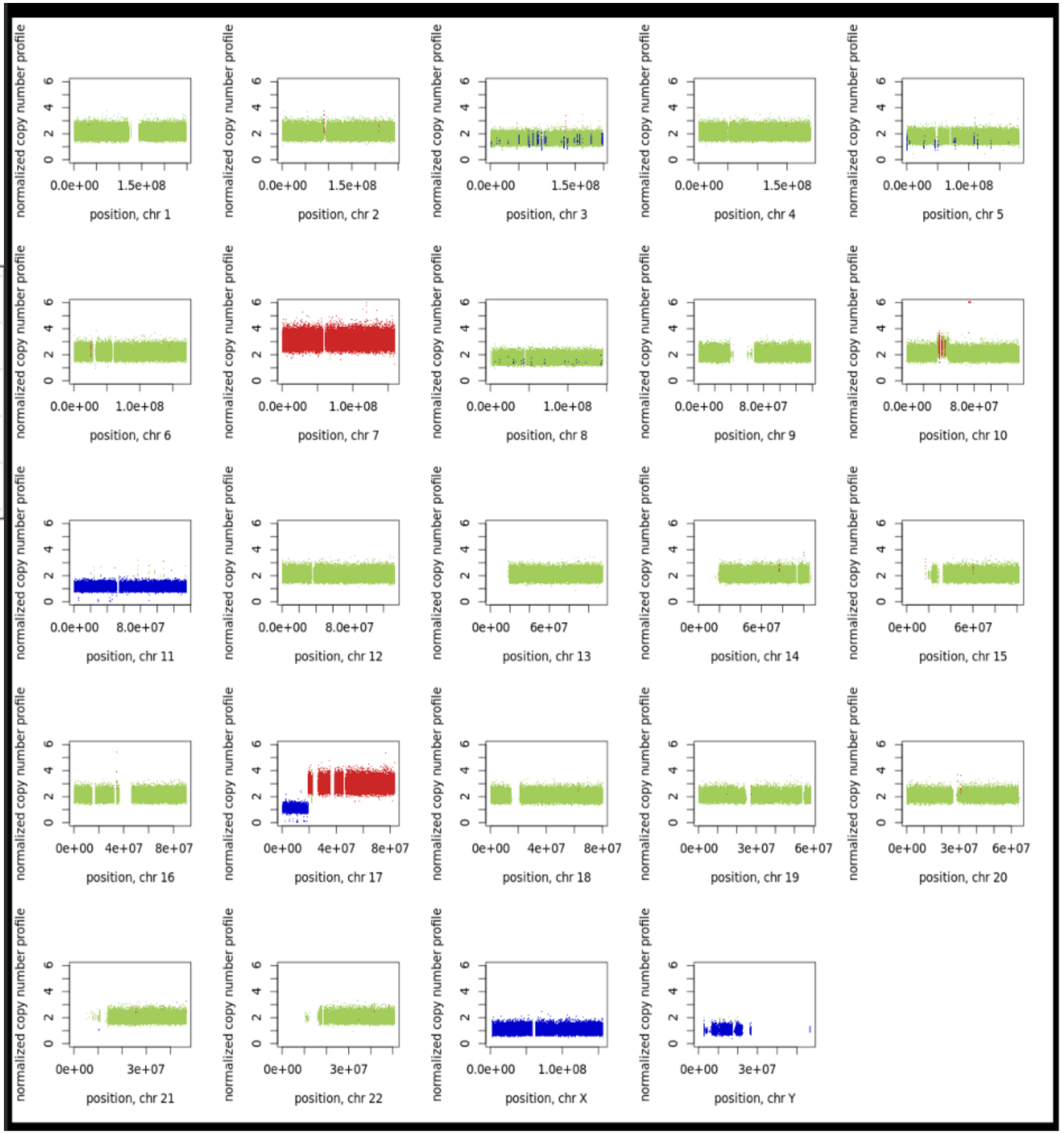
normalized copy number profile
position, chr 1
position, chr 2
position, chr 3
position, chr 4
position, chr 5
position, chr 6
position, chr 7
position, chr 8
position, chr 9
position, chr 10
position, chr 11
position, chr 12
position, chr 13
position, chr 14
position, chr 15
position, chr 16
position, chr 17
position, chr 18
position, chr 19
position, chr 20
position, chr 21
position, chr 22
position, chr X
position, chr Y

MB21 BAF

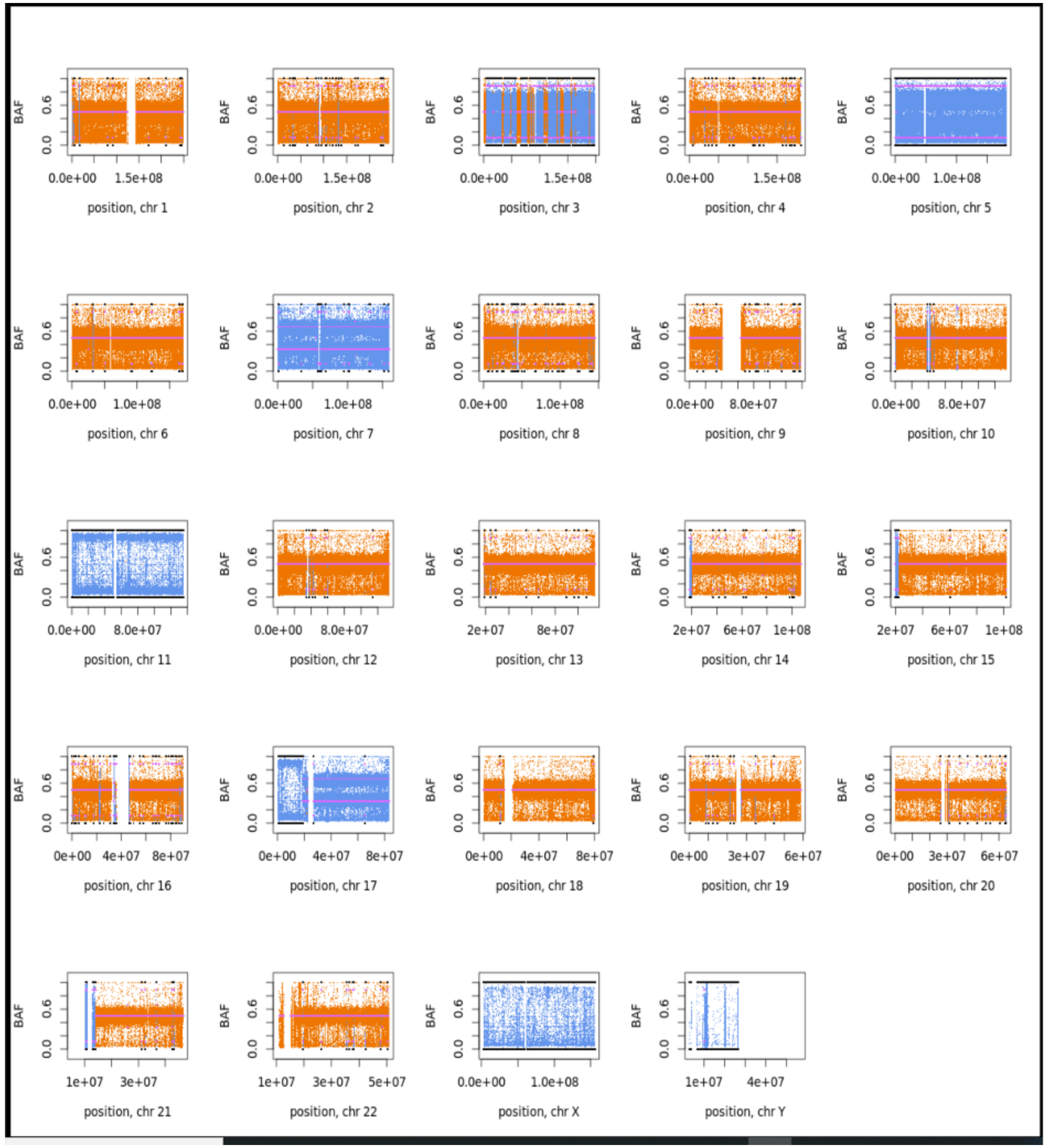
BAF
0.0
0.6
position, chr 1
position, chr 2
position, chr 3
position, chr 4
position, chr 5
position, chr 6
position, chr 7
position, chr 8
position, chr 9
position, chr 10
position, chr 11
position, chr 12
position, chr 13
position, chr 14
position, chr 15
position, chr 16
position, chr 17
position, chr 18
position, chr 19
position, chr 20
position, chr 21
position, chr 22
position, chr X
position, chr Y

MB21

Copy number variation profile

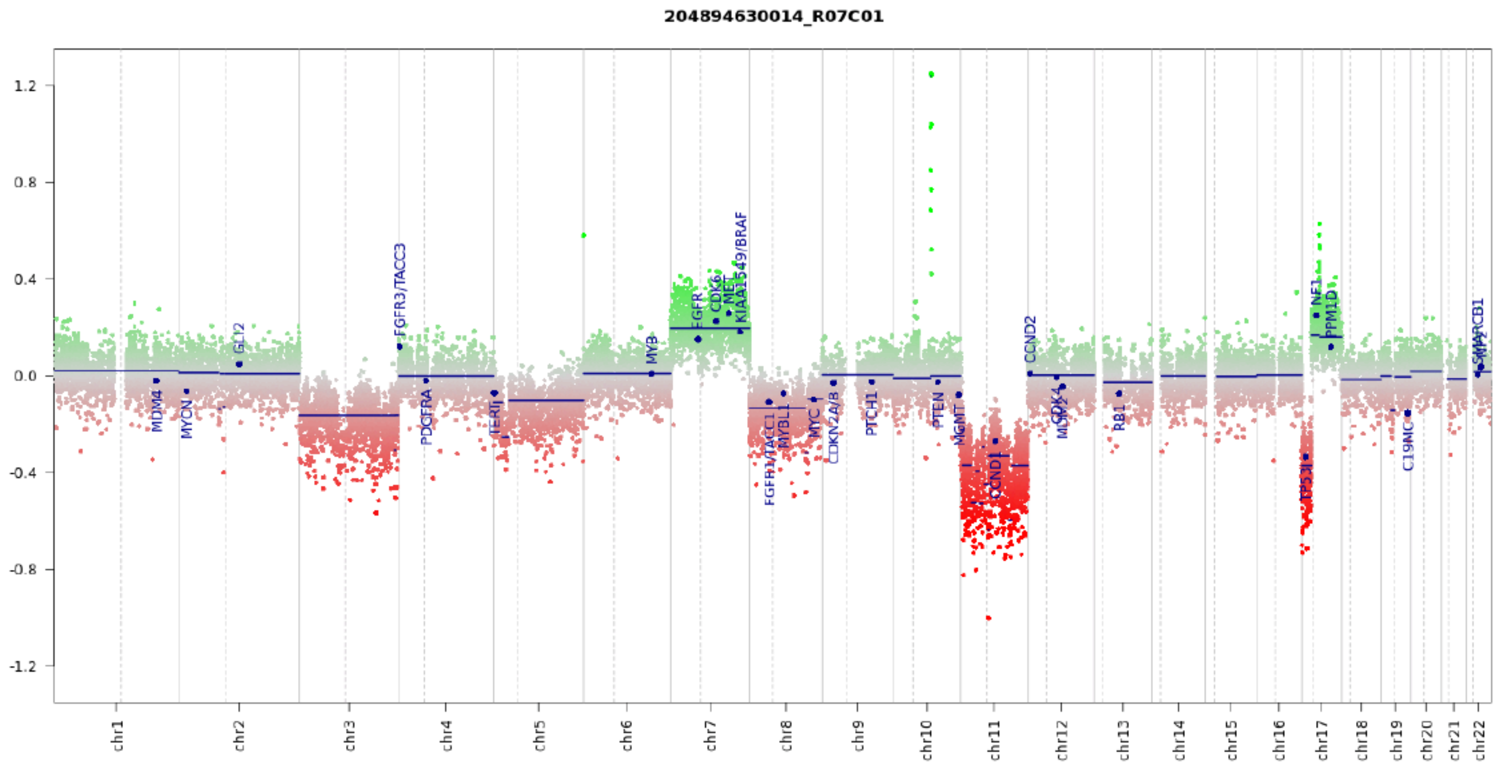

MB22

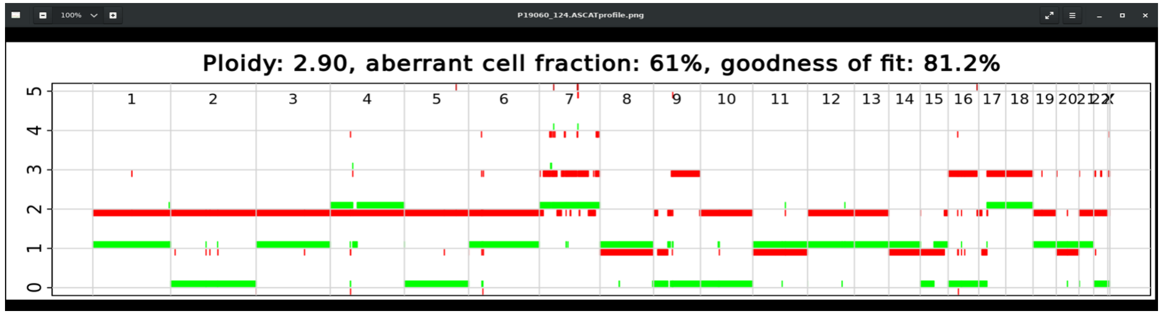
P19060_124.ASCATprofile.png
Ploidy: 2.90, aberrant cell fraction: 61%, goodness of fit: 81.2%

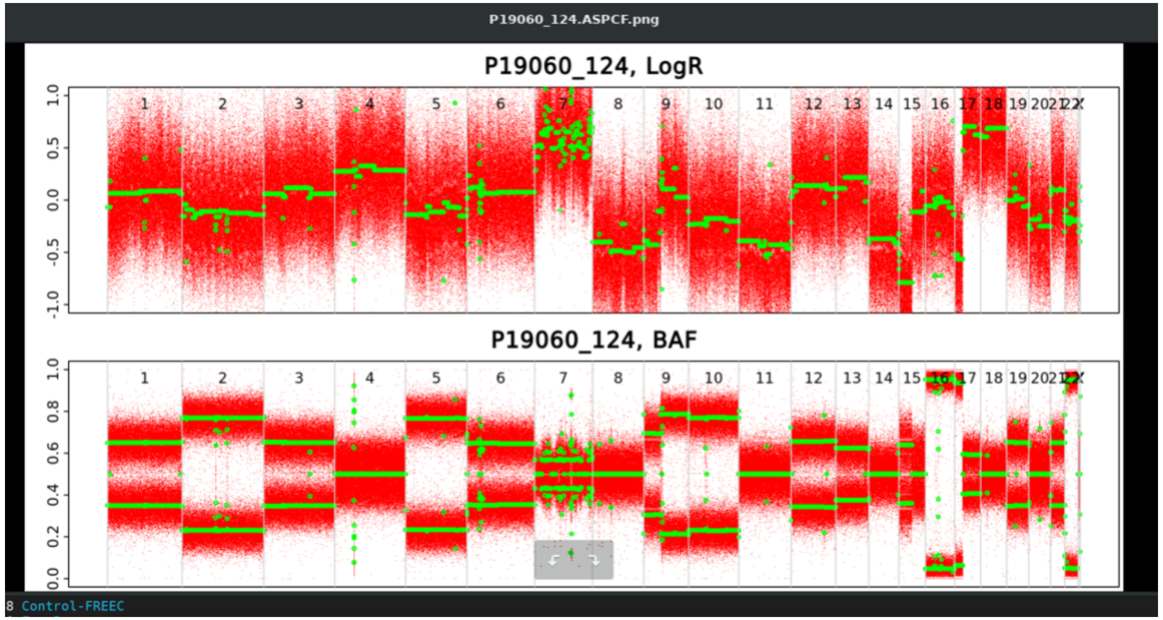
P19060_124.ASPCF.png
P19060_124, LogR
P19060_124, BAF
Control-FREEC

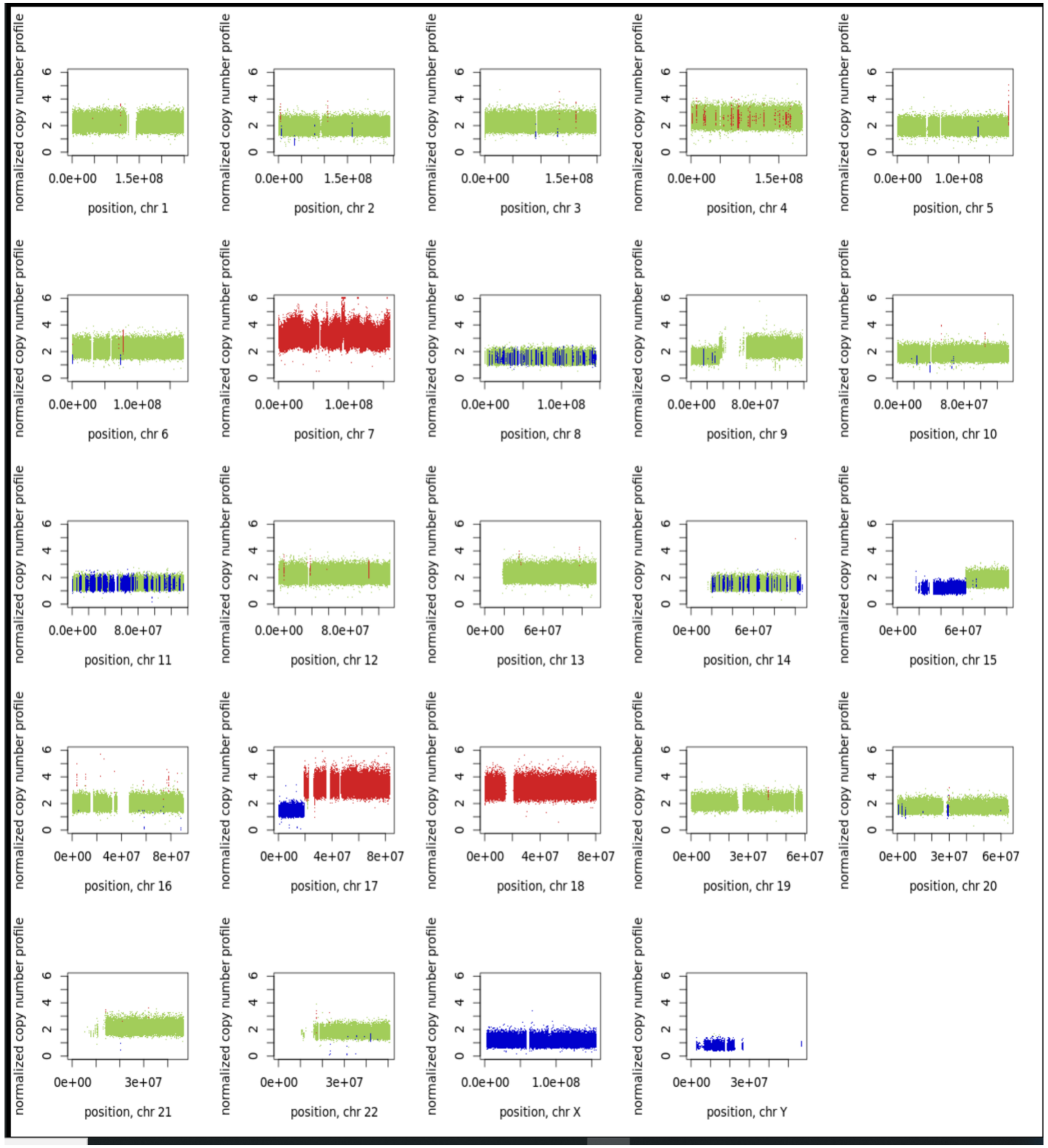
normalized copy number profile
position, chr 1
position, chr 2
position, chr 3
position, chr 4
position, chr 5
position, chr 6
position, chr 7
position, chr 8
position, chr 9
position, chr 10
position, chr 11
position, chr 12
position, chr 13
position, chr 14
position, chr 15
position, chr 16
position, chr 17
position, chr 18
position, chr 19
position, chr 20
position, chr 21
position, chr 22
position, chr X
position, chr Y

MB22 BAF

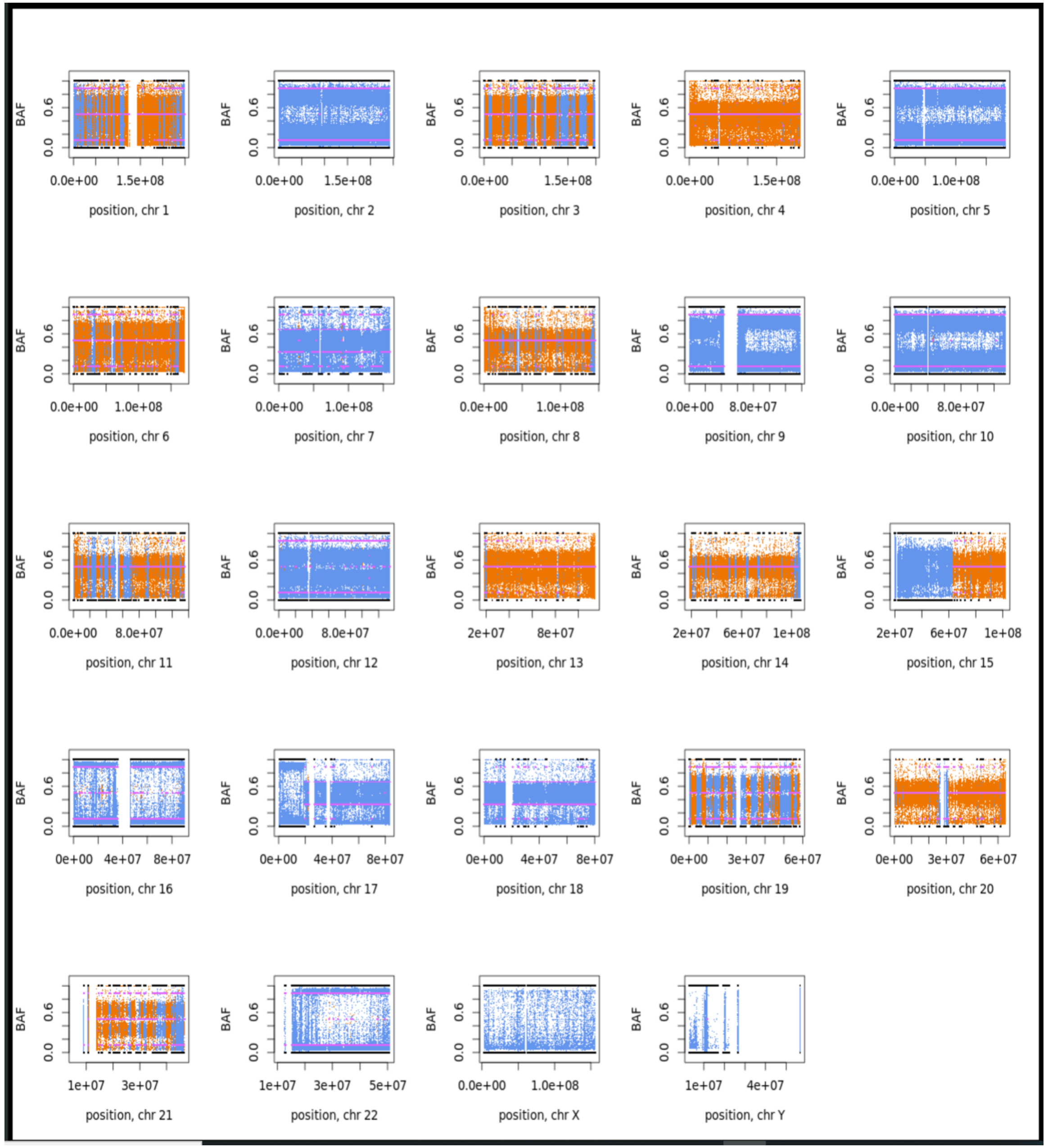

BAF
0.0
0.6
0.0e+00
1.5e+08
position, chr 1
position, chr 2
position, chr 3
position, chr 4
position, chr 5
position, chr 6
position, chr 7
position, chr 8
position, chr 9
position, chr 10
position, chr 11
position, chr 12
position, chr 13
position, chr 14
position, chr 15
position, chr 16
position, chr 17
position, chr 18
position, chr 19
position, chr 20
position, chr 21
position, chr 22
position, chr X
position, chr Y

MB22

Copy number variation profile

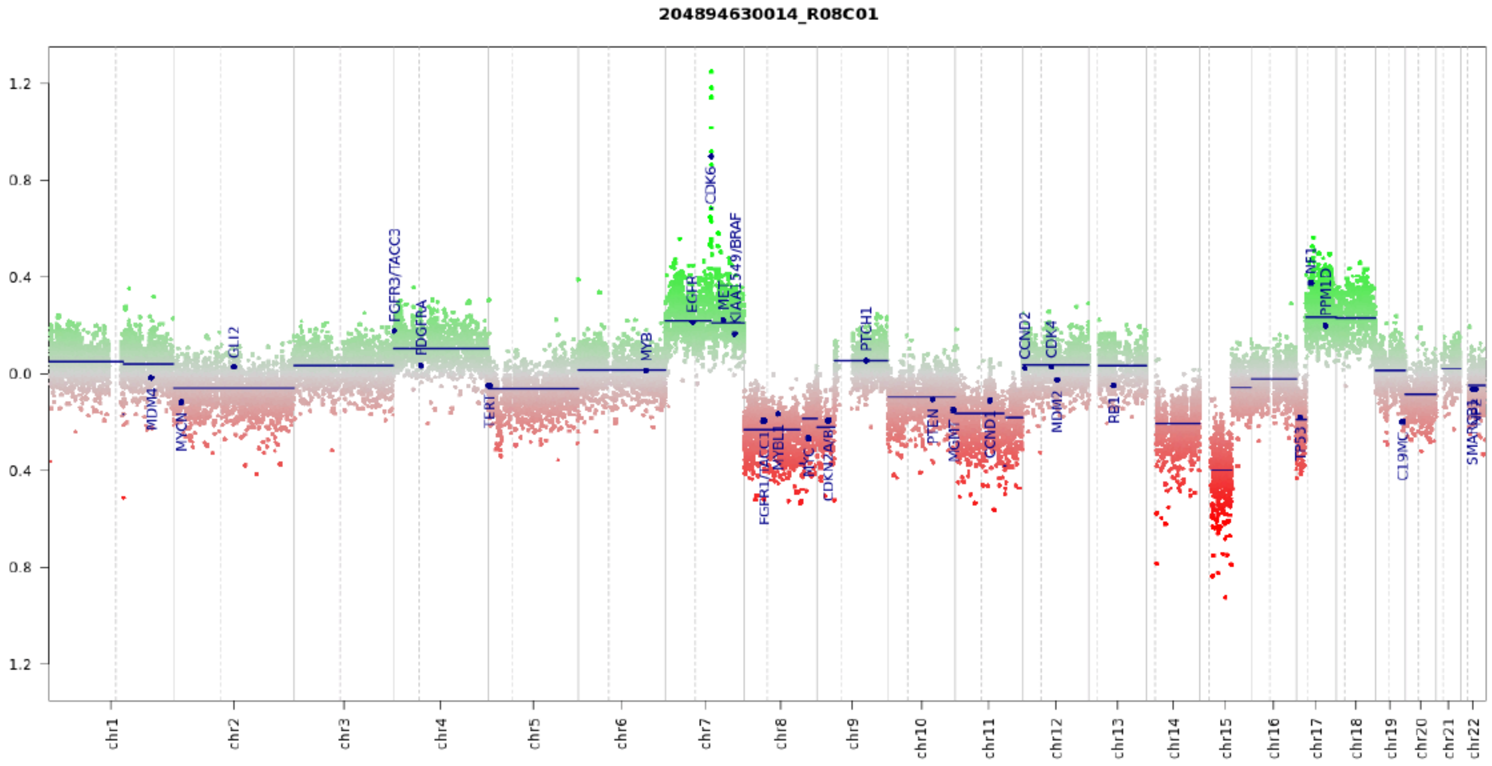

MB24

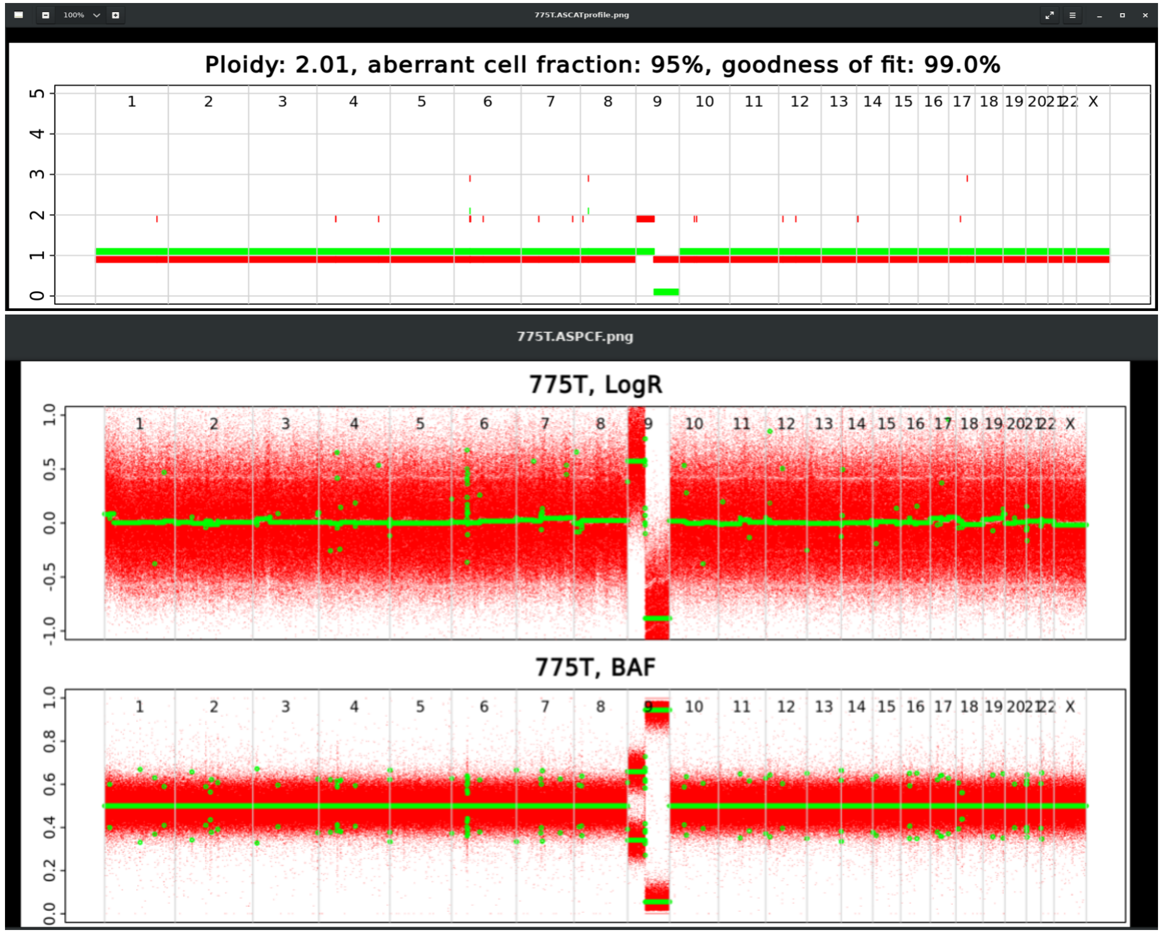
775T.ASCATprofile.png
Ploidy: 2.01, aberrant cell fraction: 95%, goodness of fit: 99.0%
775T.ASPCF.png
775T, LogR
775T, BAF

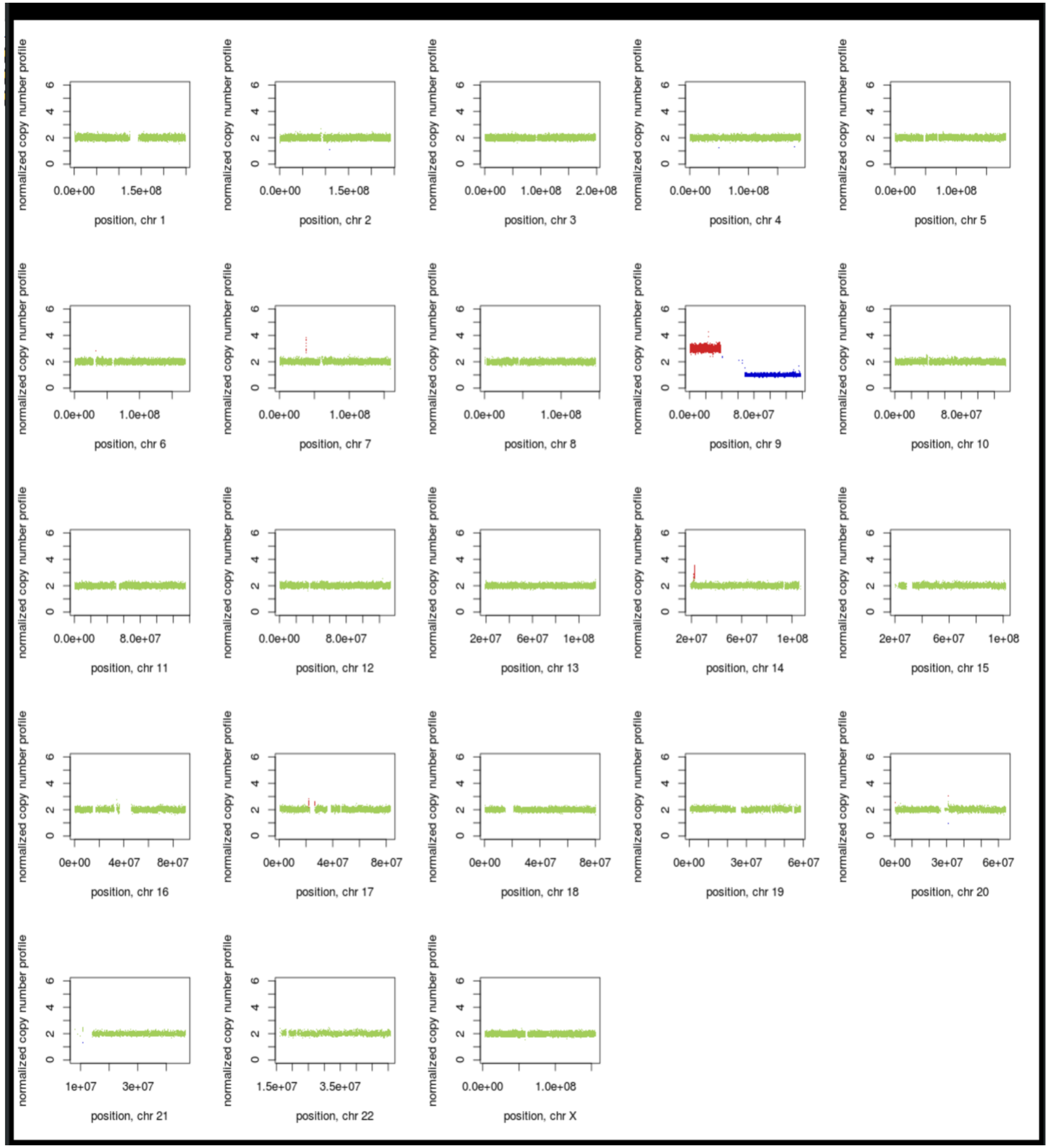
normalized copy number profile
position, chr 1
position, chr 2
position, chr 3
position, chr 4
position, chr 5
position, chr 6
position, chr 7
position, chr 8
position, chr 9
position, chr 10
position, chr 11
position, chr 12
position, chr 13
position, chr 14
position, chr 15
position, chr 16
position, chr 17
position, chr 18
position, chr 19
position, chr 20
position, chr 21
position, chr 22
position, chr X

MB24

Copy number variation profile

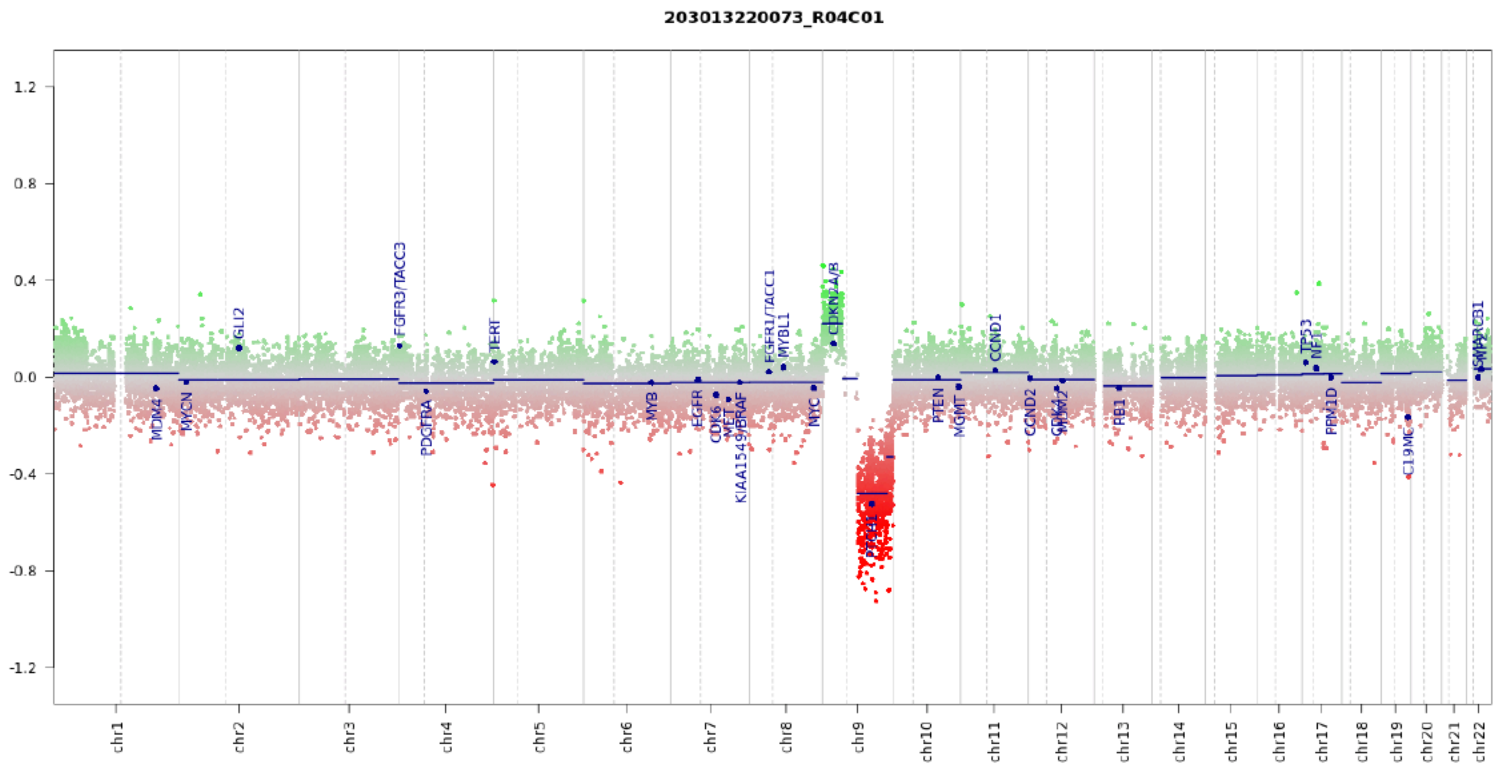

MB27

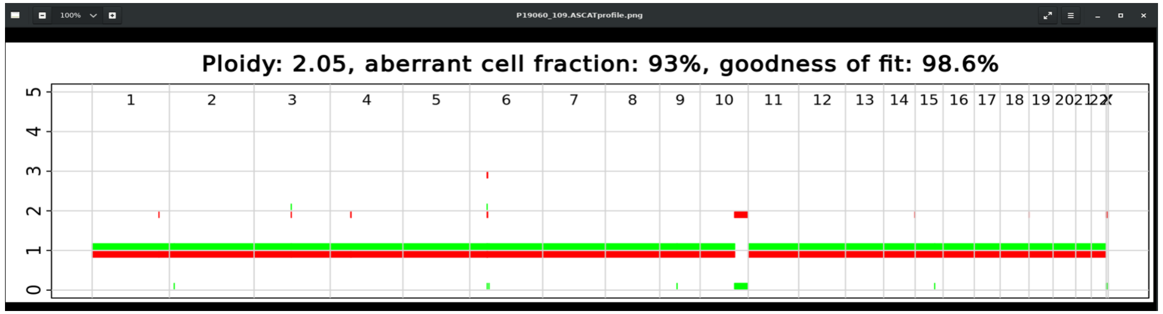
P19060_109.ASCATprofile.png
Ploidy: 2.05, aberrant cell fraction: 93%, goodness of fit: 98.6%

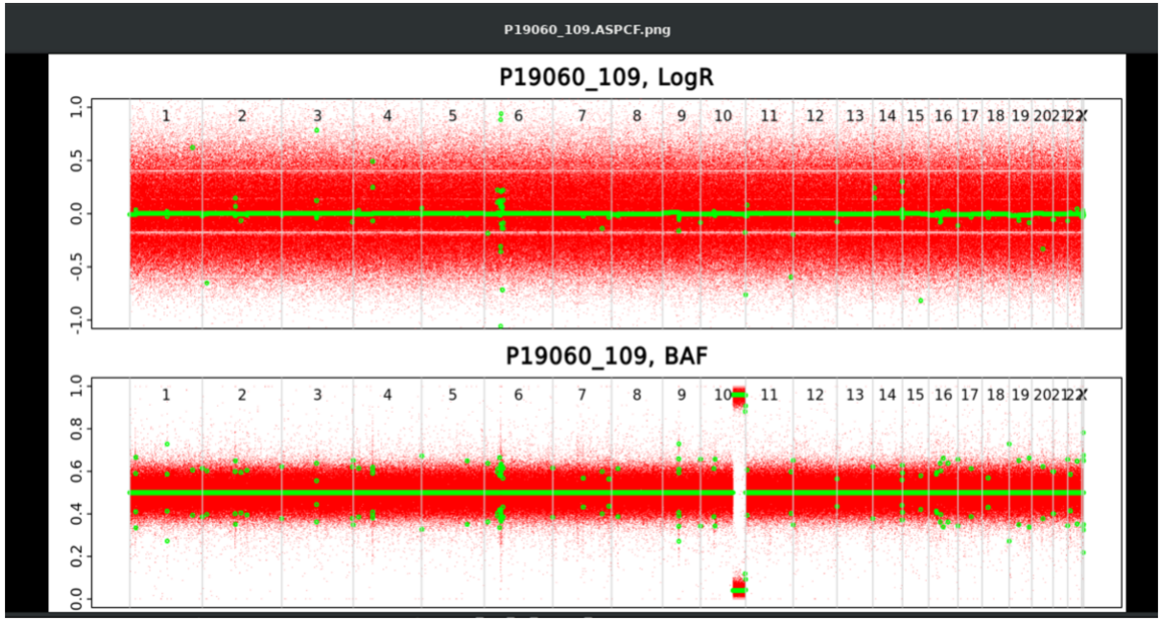
P19060_109.ASPCF.png
P19060_109, LogR
P19060_109, BAF

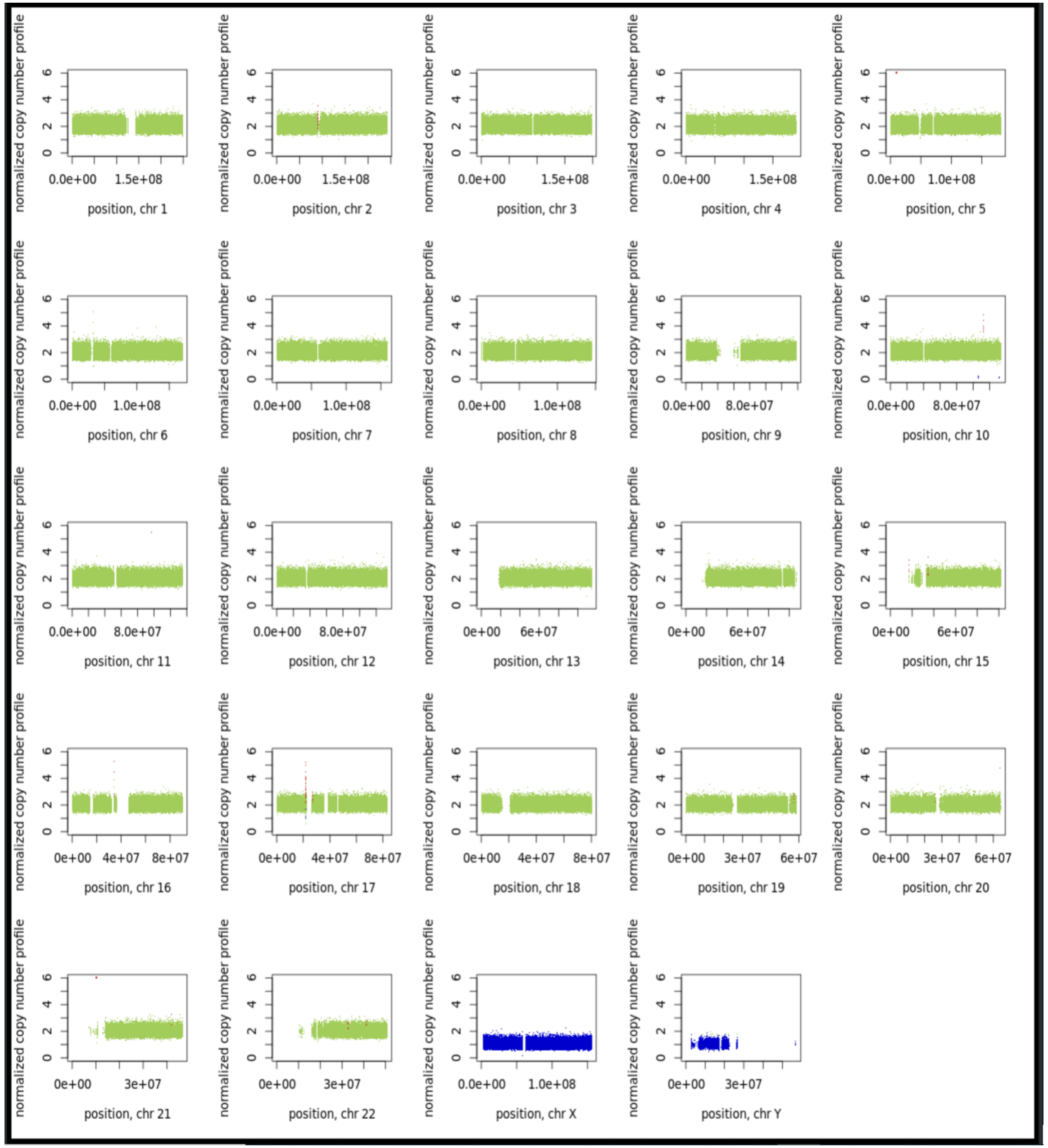
normalized copy number profile
position, chr 1
position, chr 2
position, chr 3
position, chr 4
position, chr 5
position, chr 6
position, chr 7
position, chr 8
position, chr 9
position, chr 10
position, chr 11
position, chr 12
position, chr 13
position, chr 14
position, chr 15
position, chr 16
position, chr 17
position, chr 18
position, chr 19
position, chr 20
position, chr 21
position, chr 22
position, chr X
position, chr Y

MB27 BAF

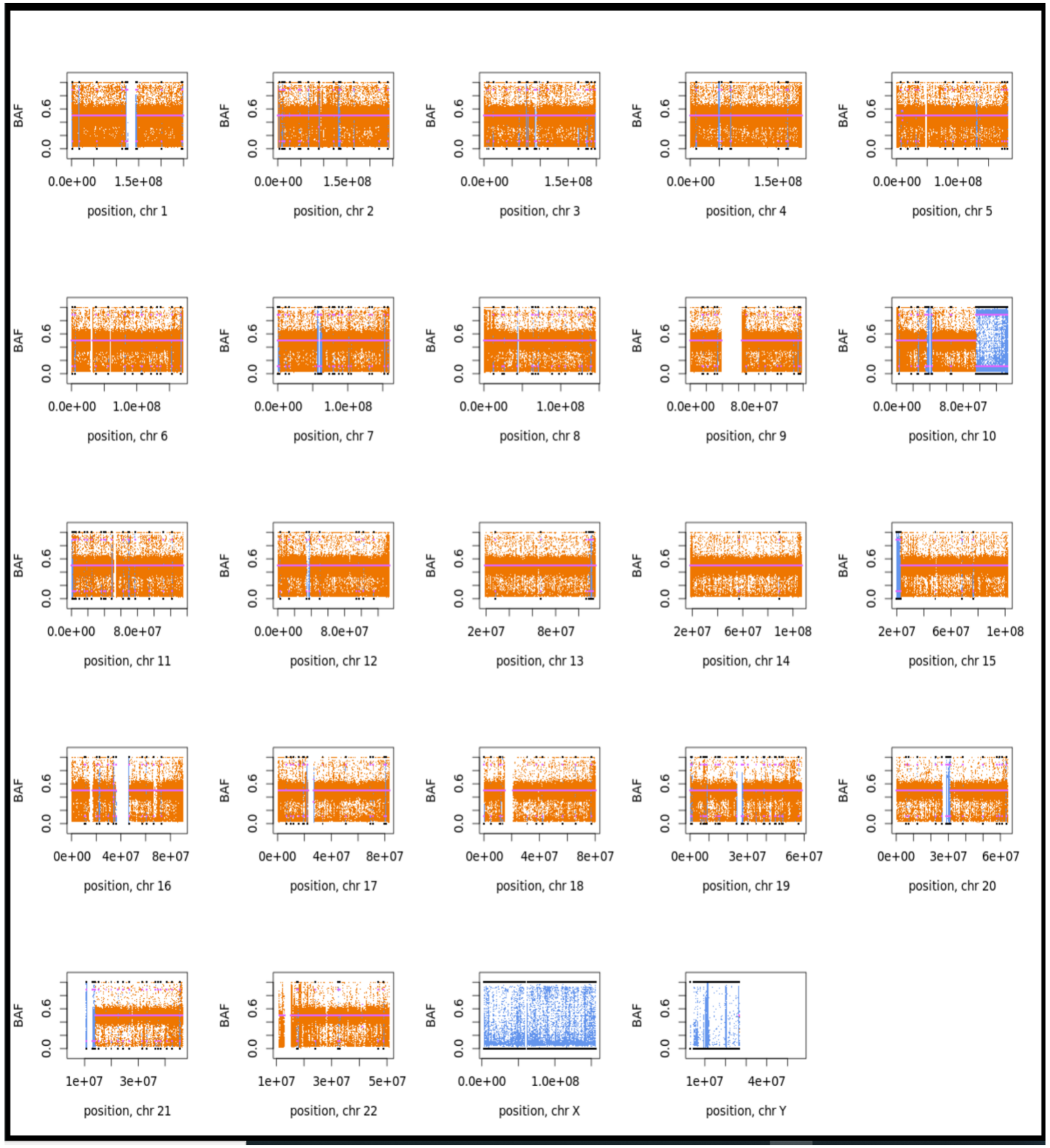

position, chr 1
position, chr 2
position, chr 3
position, chr 4
position, chr 5
position, chr 6
position, chr 7
position, chr 8
position, chr 9
position, chr 10
position, chr 11
position, chr 12
position, chr 13
position, chr 14
position, chr 15
position, chr 16
position, chr 17
position, chr 18
position, chr 19
position, chr 20
position, chr 21
position, chr 22
position, chr X
position, chr Y
BAF

MB27

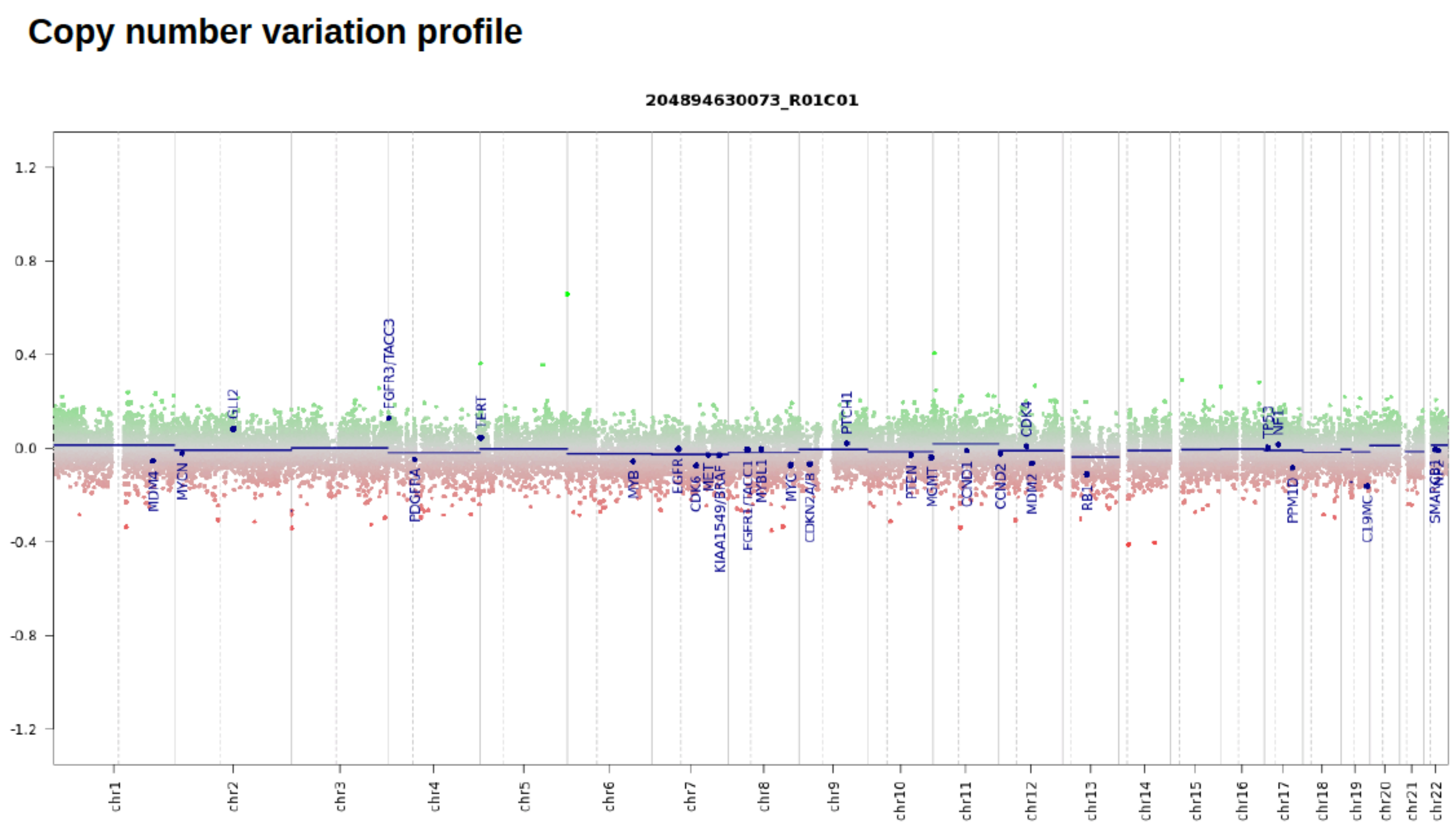
Copy number variation profile
204894630073_R01C01
1.2
0.8
0.4
0.0
-0.4
-0.8
-1.2
chr1
chr2
chr3
chr4
chr5
chr6
chr7
chr8
chr9
chr10
chr11
chr12
chr13
chr14
chr15
chr16
chr17
chr18
chr19
chr20
chr21
chr22
MDM4
MYCN
GLI2
FGFR3/TACC3
PDGFRA
TERT
MYB
EGFR
CDK6
MET
KIAA1549/BRAF
FGFR1/TACC1
MYBL1
MYC
CDKN2A/B
PTCH1
PTEN
MGMT
CCND1
CCND2
CDK4
MDM2
RB1
TP53
NF1
PPM1D
C19MC
SMARCB1

MB31

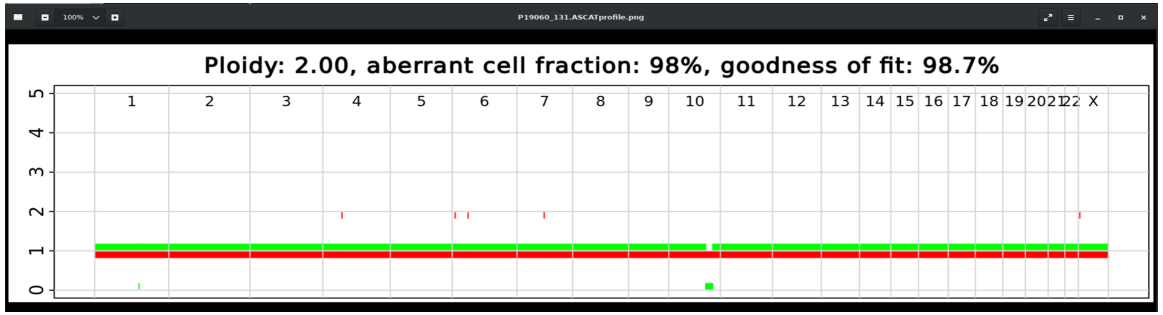
P19060_131.ASCATprofile.png
Ploidy: 2.00, aberrant cell fraction: 98%, goodness of fit: 98.7%
1 2 3 4 5 6 7 8 9 10 11 12 13 14 15 16 17 18 19 20 21 22 X
0 1 2 3 4 5

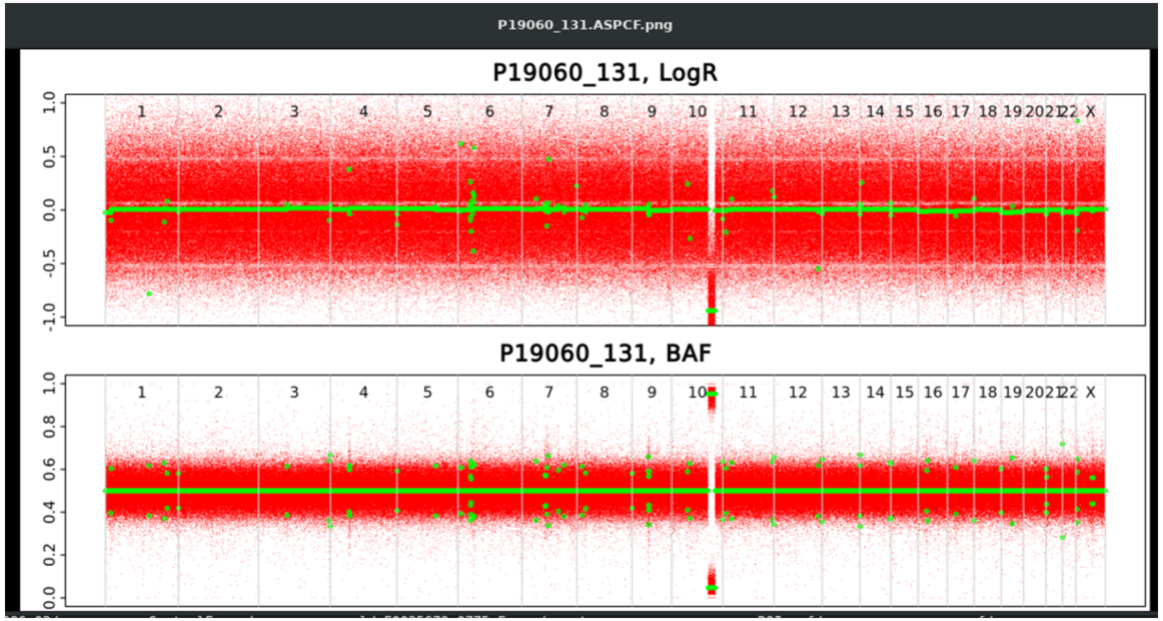
P19060_131.ASPCF.png
P19060_131, LogR
P19060_131, BAF
1 2 3 4 5 6 7 8 9 10 11 12 13 14 15 16 17 18 19 20 21 22 X

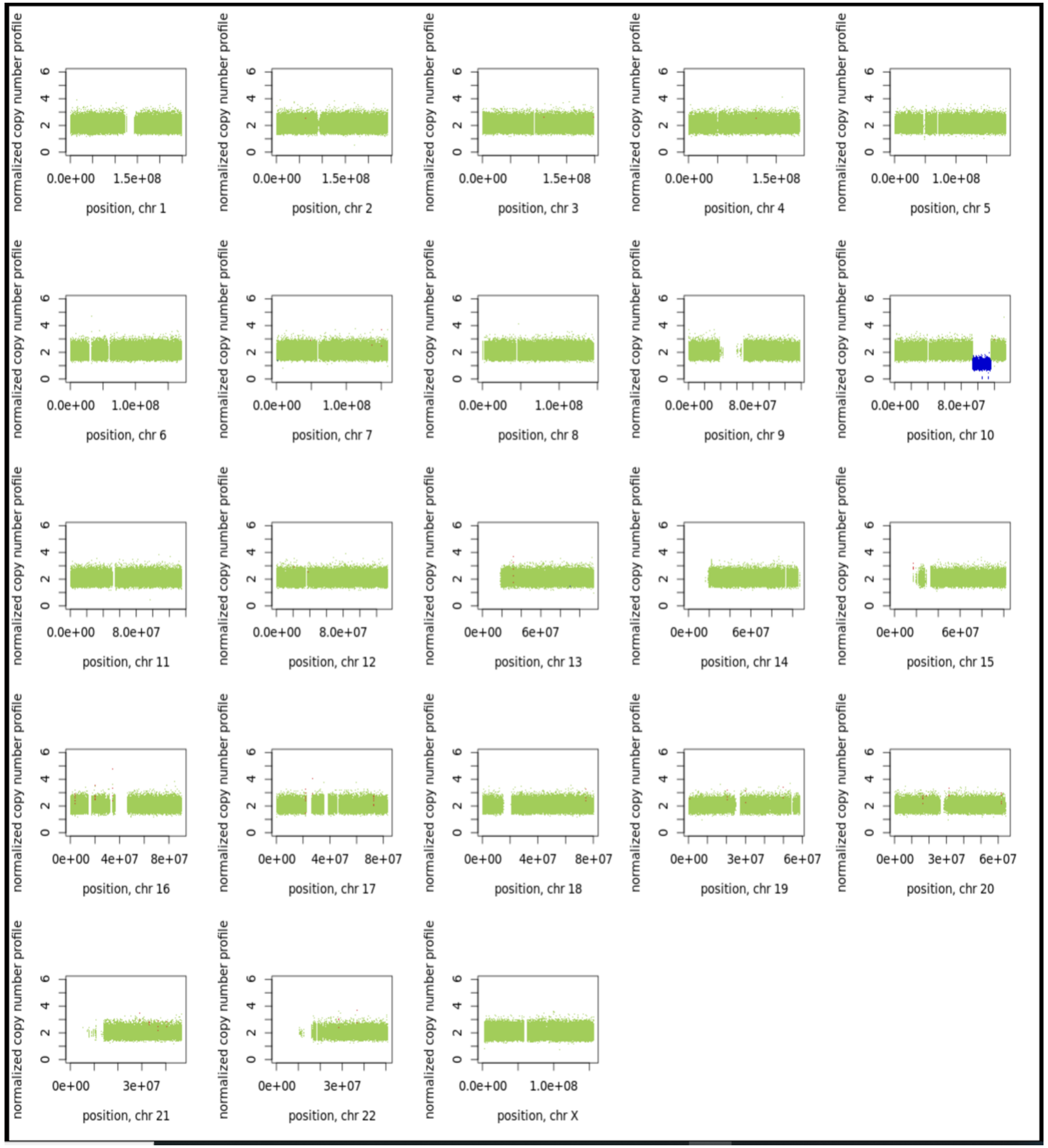
normalized copy number profile
position, chr 1
position, chr 2
position, chr 3
position, chr 4
position, chr 5
position, chr 6
position, chr 7
position, chr 8
position, chr 9
position, chr 10
position, chr 11
position, chr 12
position, chr 13
position, chr 14
position, chr 15
position, chr 16
position, chr 17
position, chr 18
position, chr 19
position, chr 20
position, chr 21
position, chr 22
position, chr X

MB31

Copy number variation profile

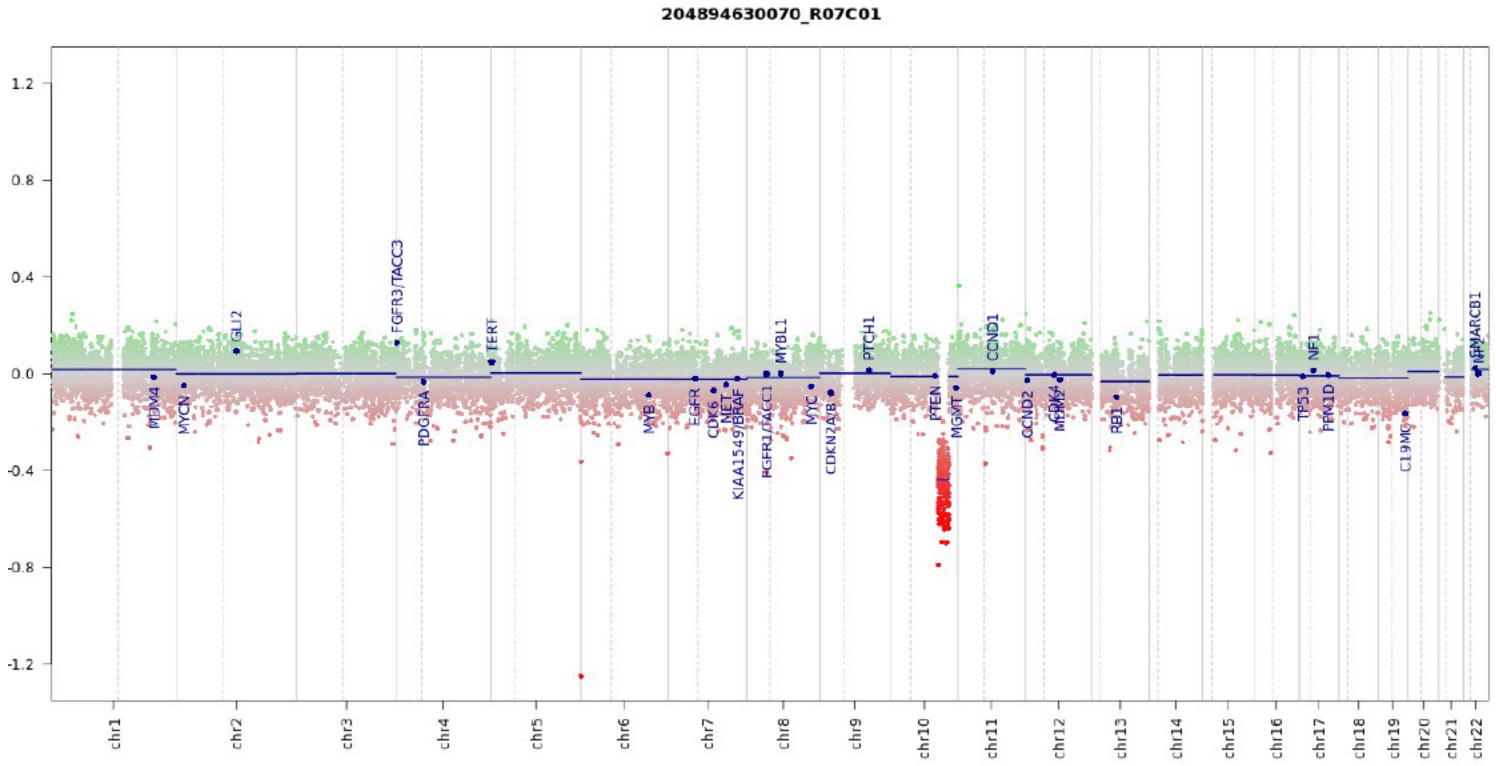

MB35

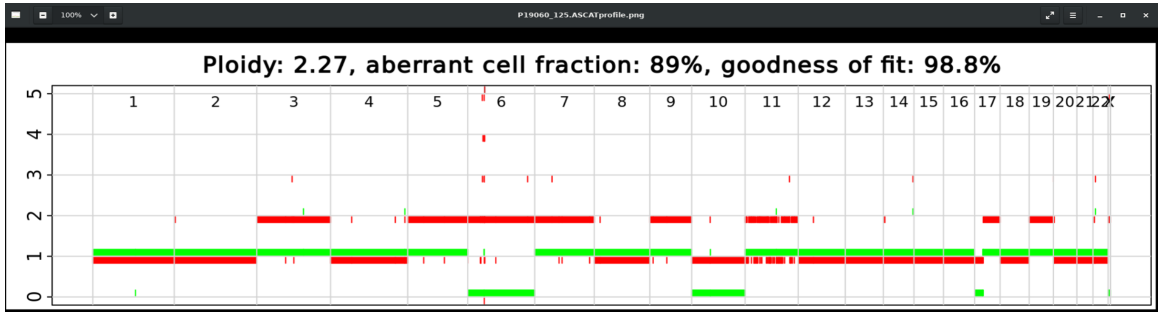
P19060_125.ASCATprofile.png
Ploidy: 2.27, aberrant cell fraction: 89%, goodness of fit: 98.8%

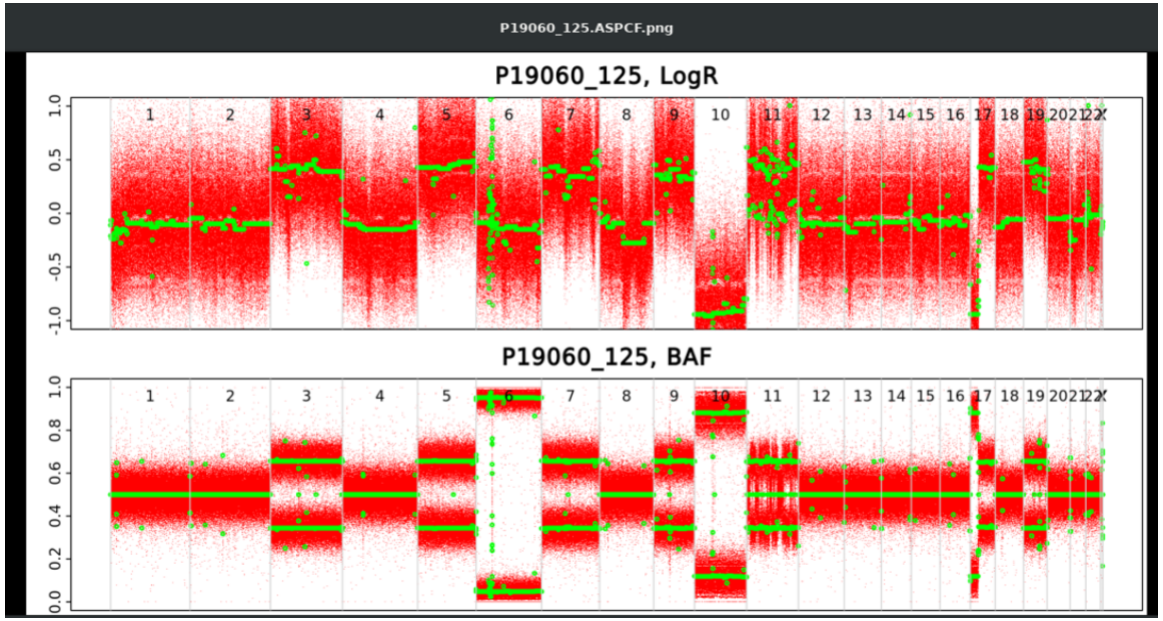
P19060_125.ASPCF.png
P19060_125, LogR
P19060_125, BAF

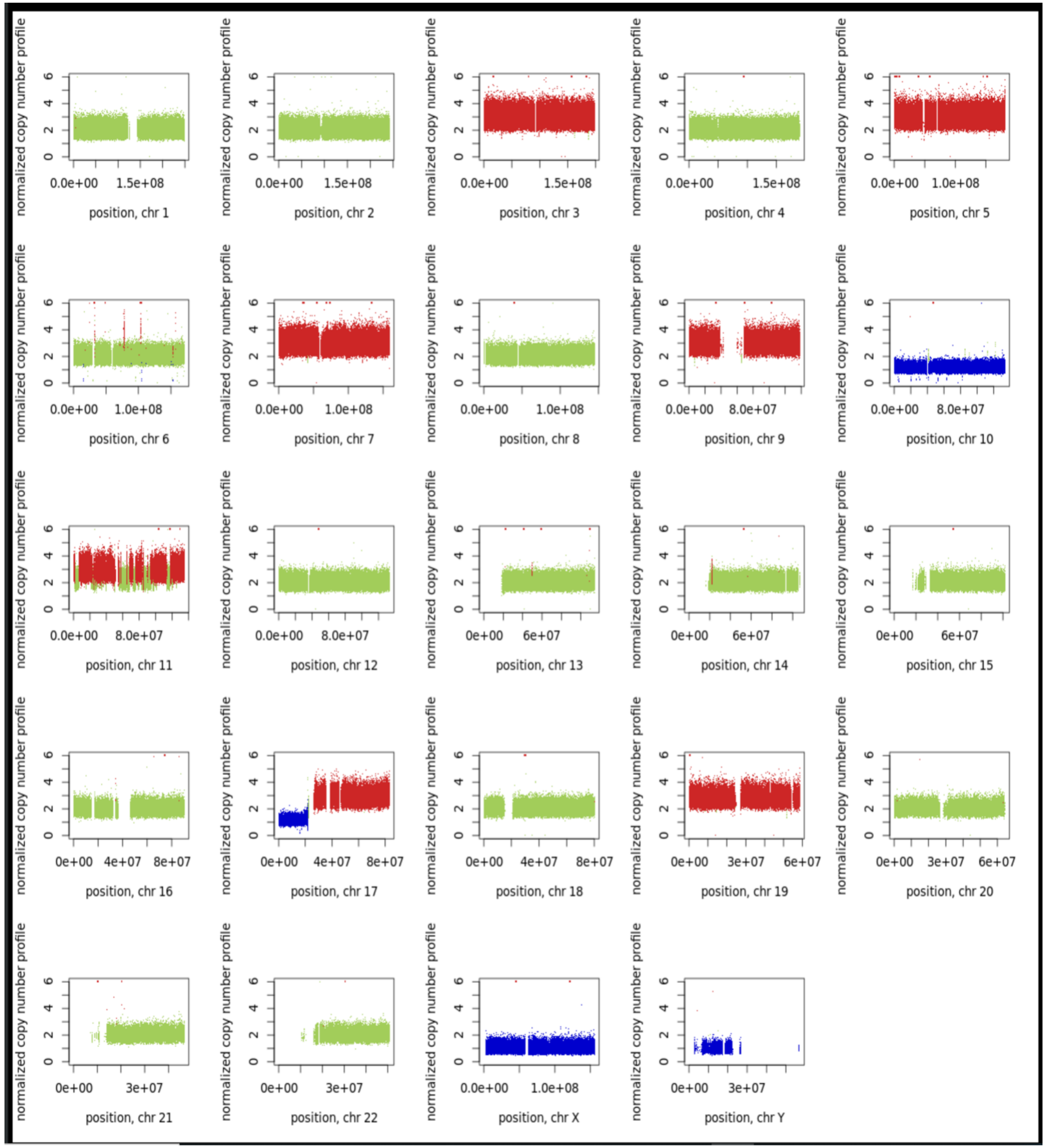
normalized copy number profile
position, chr 1
position, chr 2
position, chr 3
position, chr 4
position, chr 5
position, chr 6
position, chr 7
position, chr 8
position, chr 9
position, chr 10
position, chr 11
position, chr 12
position, chr 13
position, chr 14
position, chr 15
position, chr 16
position, chr 17
position, chr 18
position, chr 19
position, chr 20
position, chr 21
position, chr 22
position, chr X
position, chr Y

MB35 BAF

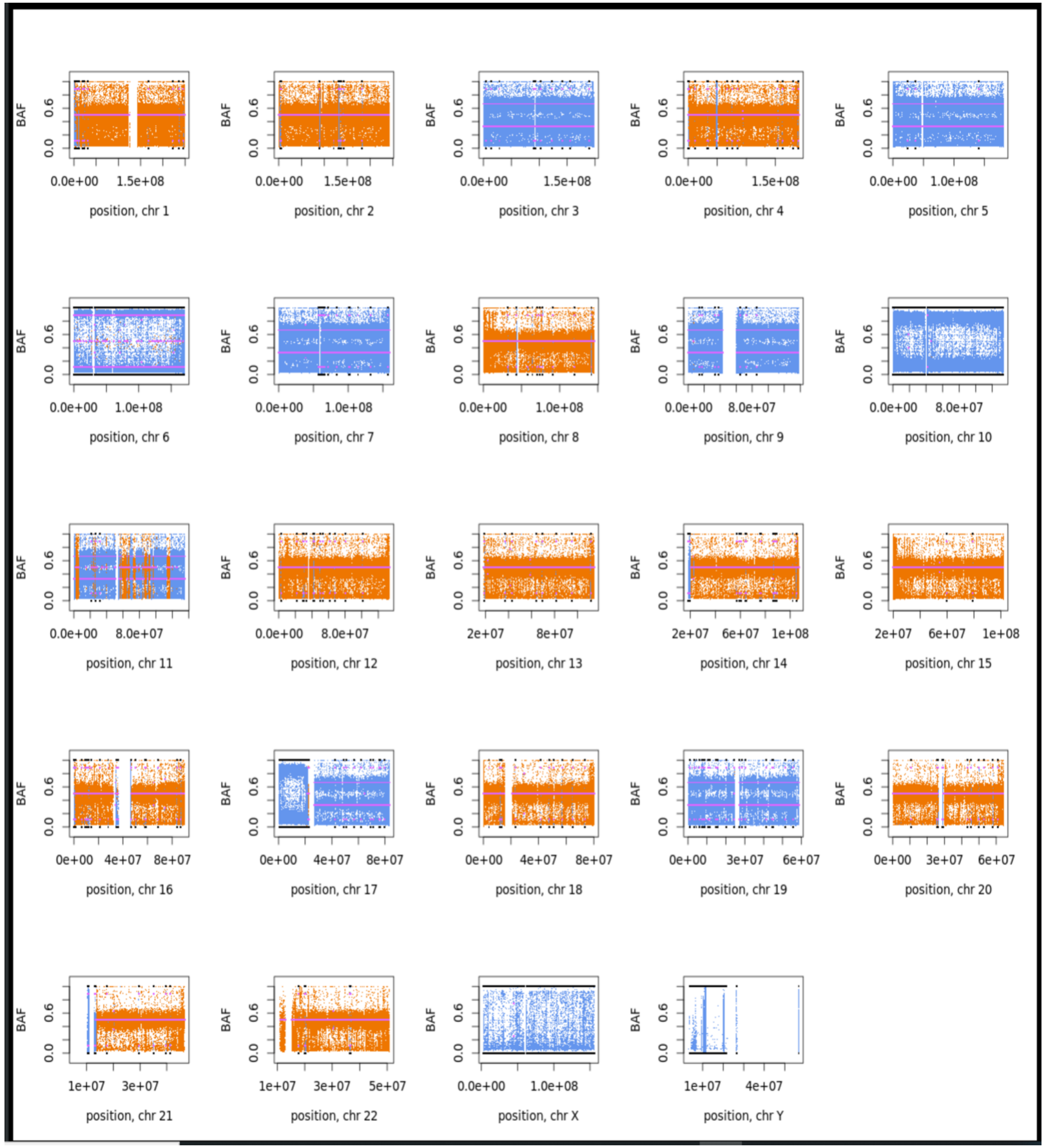

position, chr 1
position, chr 2
position, chr 3
position, chr 4
position, chr 5
position, chr 6
position, chr 7
position, chr 8
position, chr 9
position, chr 10
position, chr 11
position, chr 12
position, chr 13
position, chr 14
position, chr 15
position, chr 16
position, chr 17
position, chr 18
position, chr 19
position, chr 20
position, chr 21
position, chr 22
position, chr X
position, chr Y
BAF

MB35

Copy number variation profile

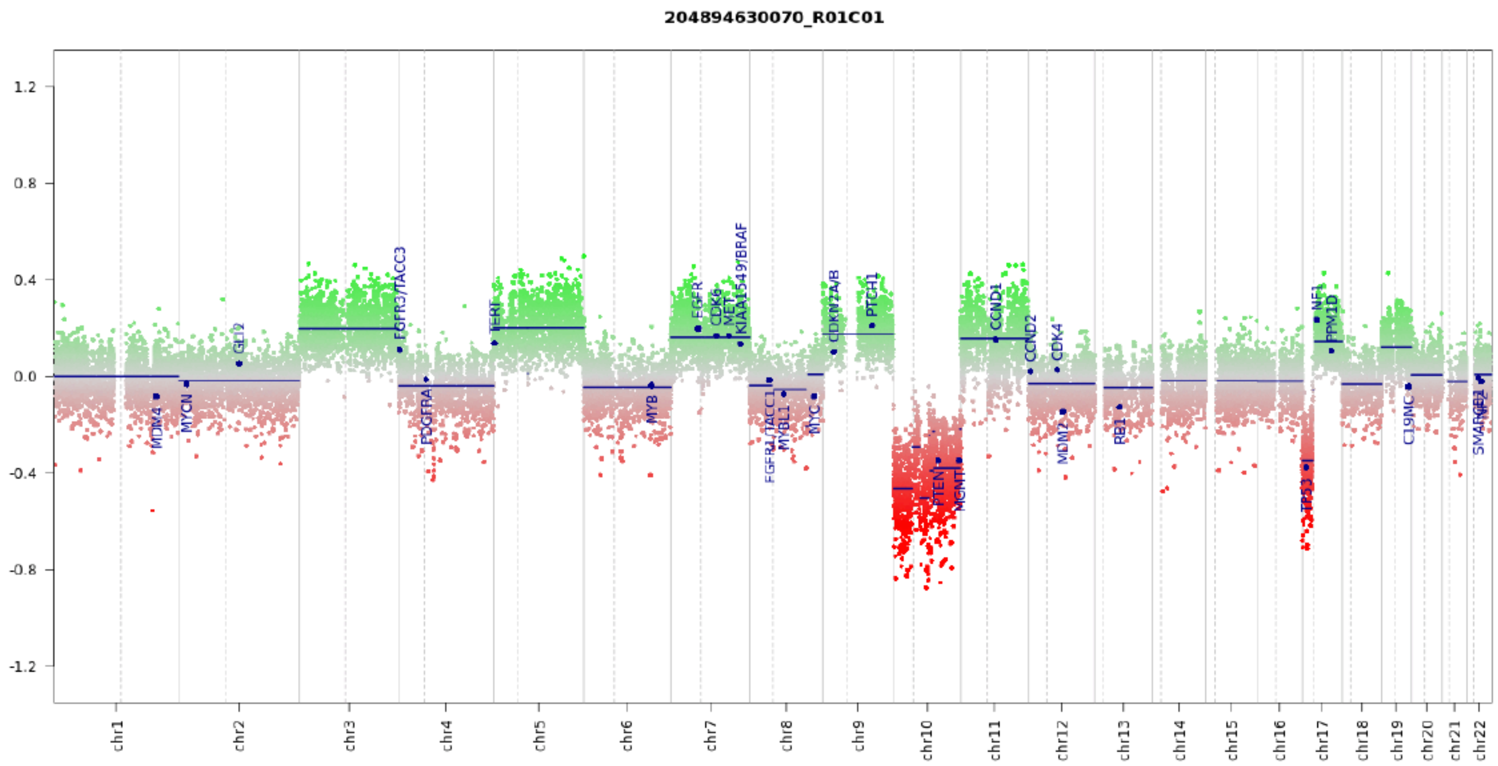

MB43

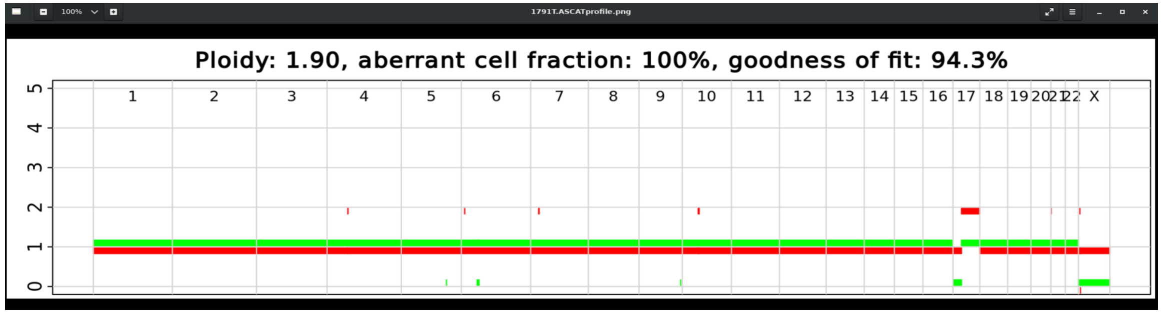
1791T.ASCATprofile.png
Ploidy: 1.90, aberrant cell fraction: 100%, goodness of fit: 94.3%

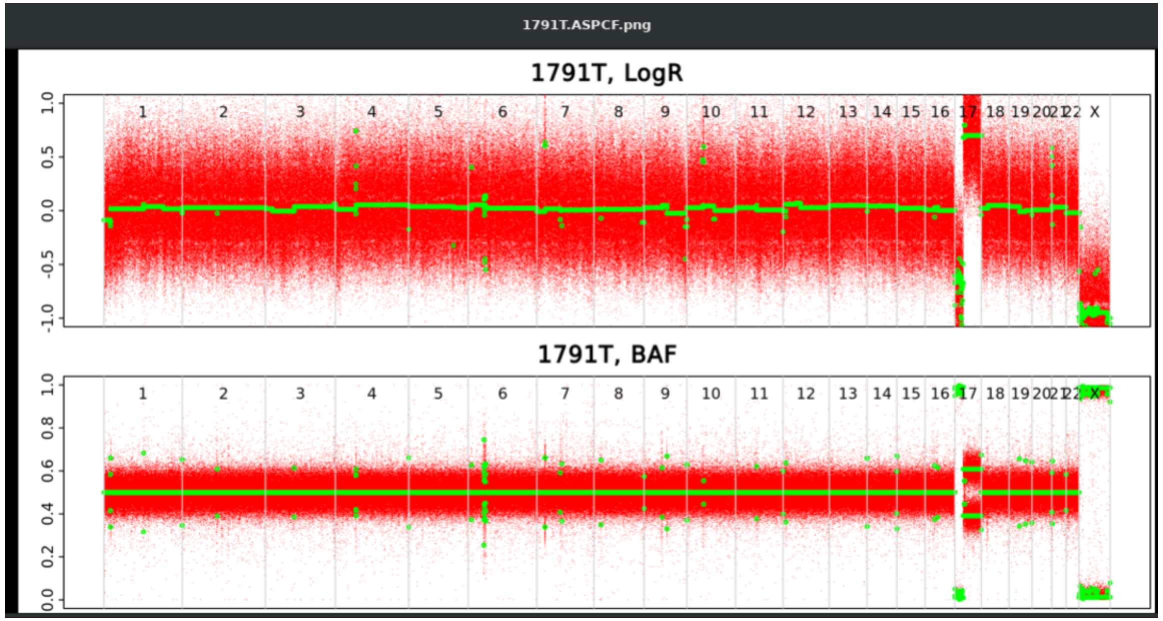
1791T.ASPCF.png
1791T, LogR
1791T, BAF

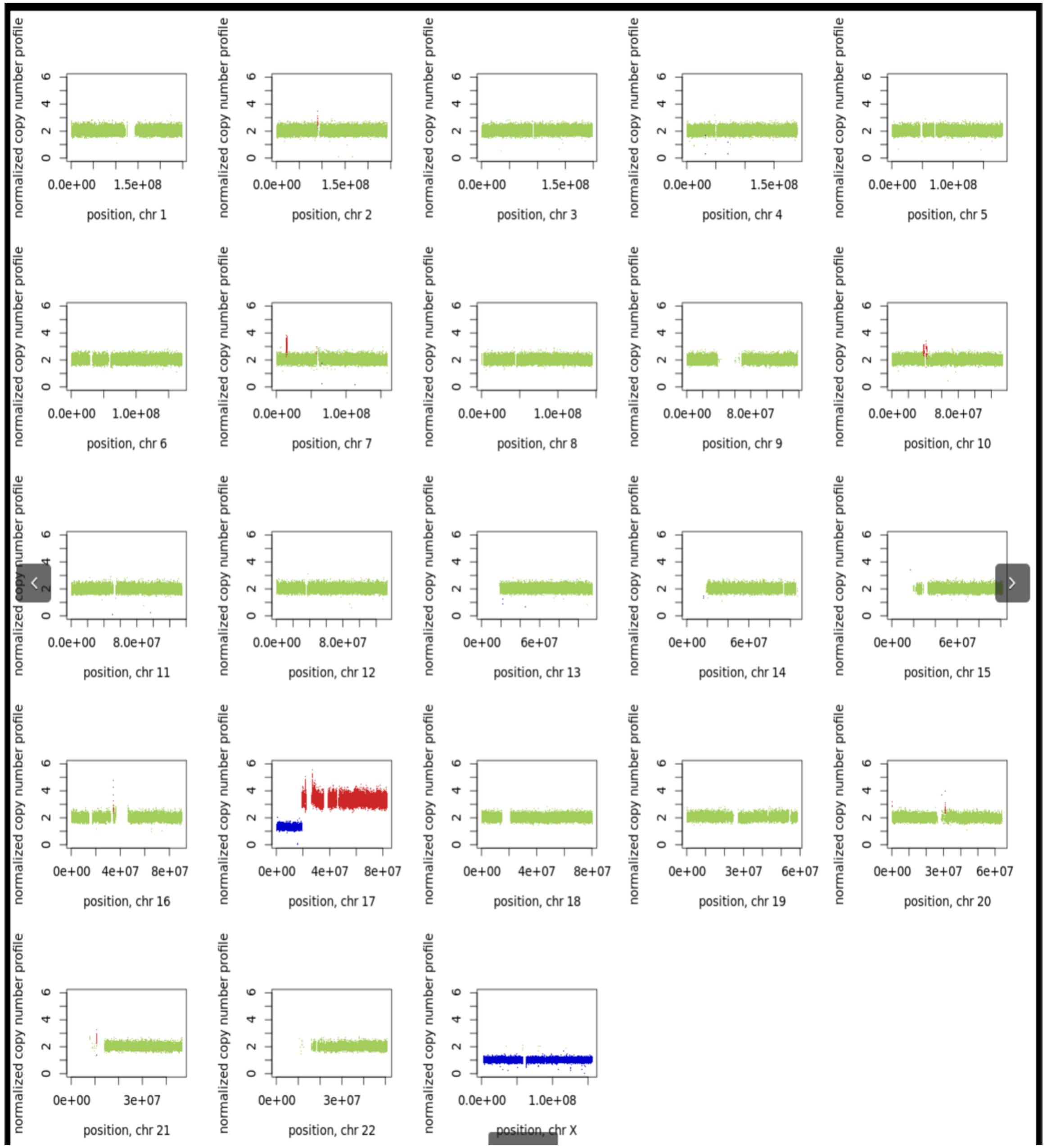
normalized copy number profile
position, chr 1
position, chr 2
position, chr 3
position, chr 4
position, chr 5
position, chr 6
position, chr 7
position, chr 8
position, chr 9
position, chr 10
position, chr 11
position, chr 12
position, chr 13
position, chr 14
position, chr 15
position, chr 16
position, chr 17
position, chr 18
position, chr 19
position, chr 20
position, chr 21
position, chr 22
position, chr X
