# Supplementary material for: Simultaneous Ultra-Sensitive Detection of Structural and Single Nucleotide Variants Using Multiplex Droplet Digital PCR in Liquid Biopsies from Children with Medulloblastoma
Source: Cancers (Basel). 2023 Mar 25;15(7):1972. doi: 10.3390/cancers15071972 (PMC10092983; doi:10.3390/cancers15071972)
Supplement: Supplementary file 1 [file cancers-15-01972-s001.zip › File S2 QX Manager Software output data on liquid biopsies/Liquid biopsies MB01.pdf]

MB01

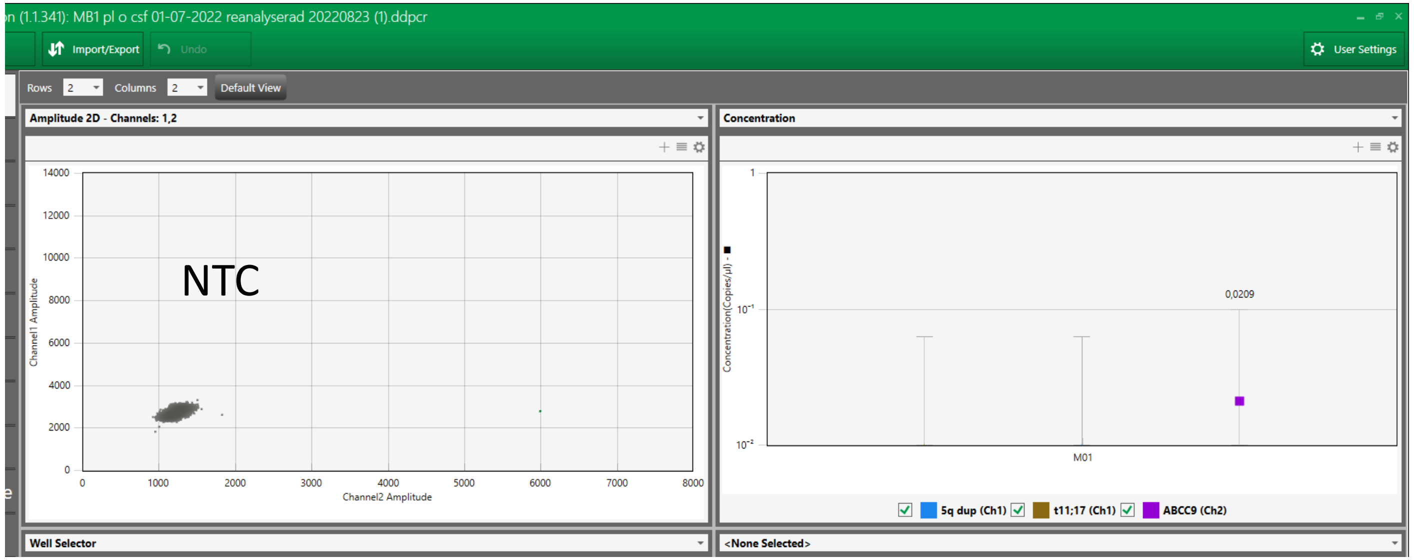
n (1.1.341): MB1 pl o csf 01-07-2022 reanalyserad 20220823 (1).ddpcr
Import/Export
Undo
User Settings
Rows 2 Columns 2 Default View
Amplitude 2D - Channels: 1,2
Concentration
NTC
Channel1 Amplitude
Channel2 Amplitude
Concentration(Copies/µl)
0,0209
M01
5q dup (Ch1)
t11;17 (Ch1)
ABCC9 (Ch2)
Well Selector
<None Selected>

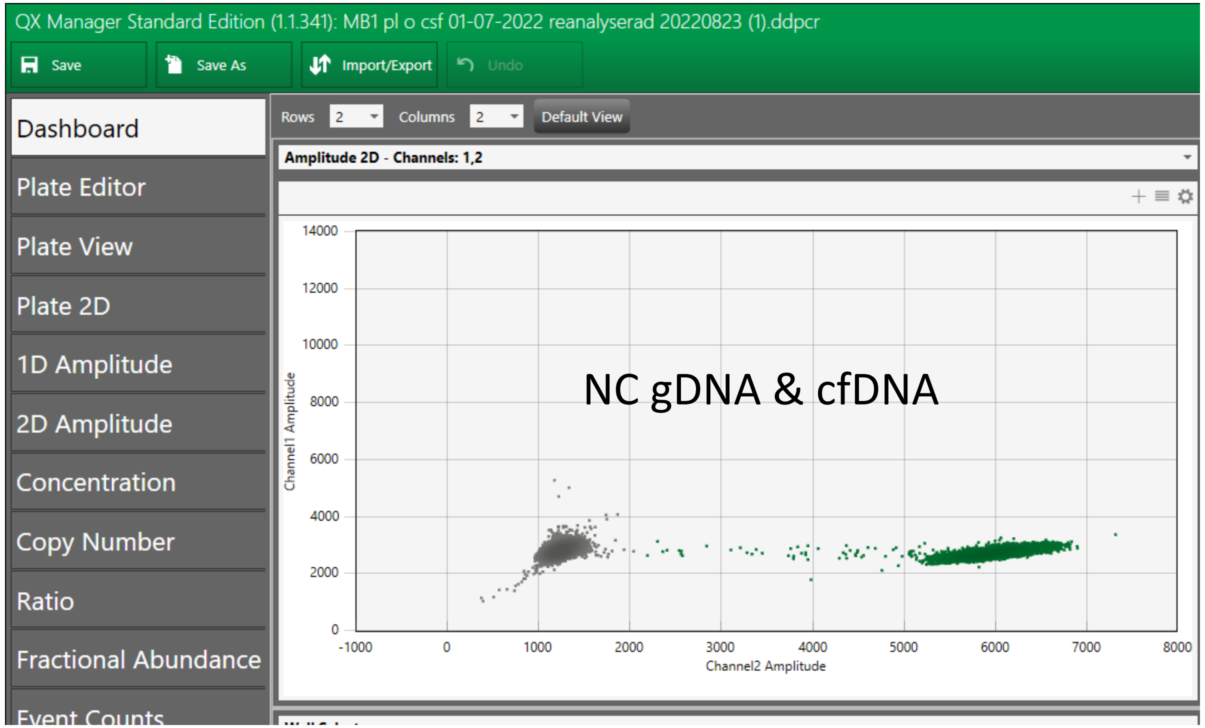
QX Manager Standard Edition (1.1.341): MB1 pl o csf 01-07-2022 reanalyserad 20220823 (1).ddpcr
Save
Save As
Import/Export
Undo
Dashboard
Plate Editor
Plate View
Plate 2D
1D Amplitude
2D Amplitude
Concentration
Copy Number
Ratio
Fractional Abundance
Event Counts
Rows 2 Columns 2 Default View
Amplitude 2D - Channels: 1,2
NC gDNA & cfDNA
Channel1 Amplitude
Channel2 Amplitude

MB01 Dilution series: LoD & LoQ 1:100 both targets

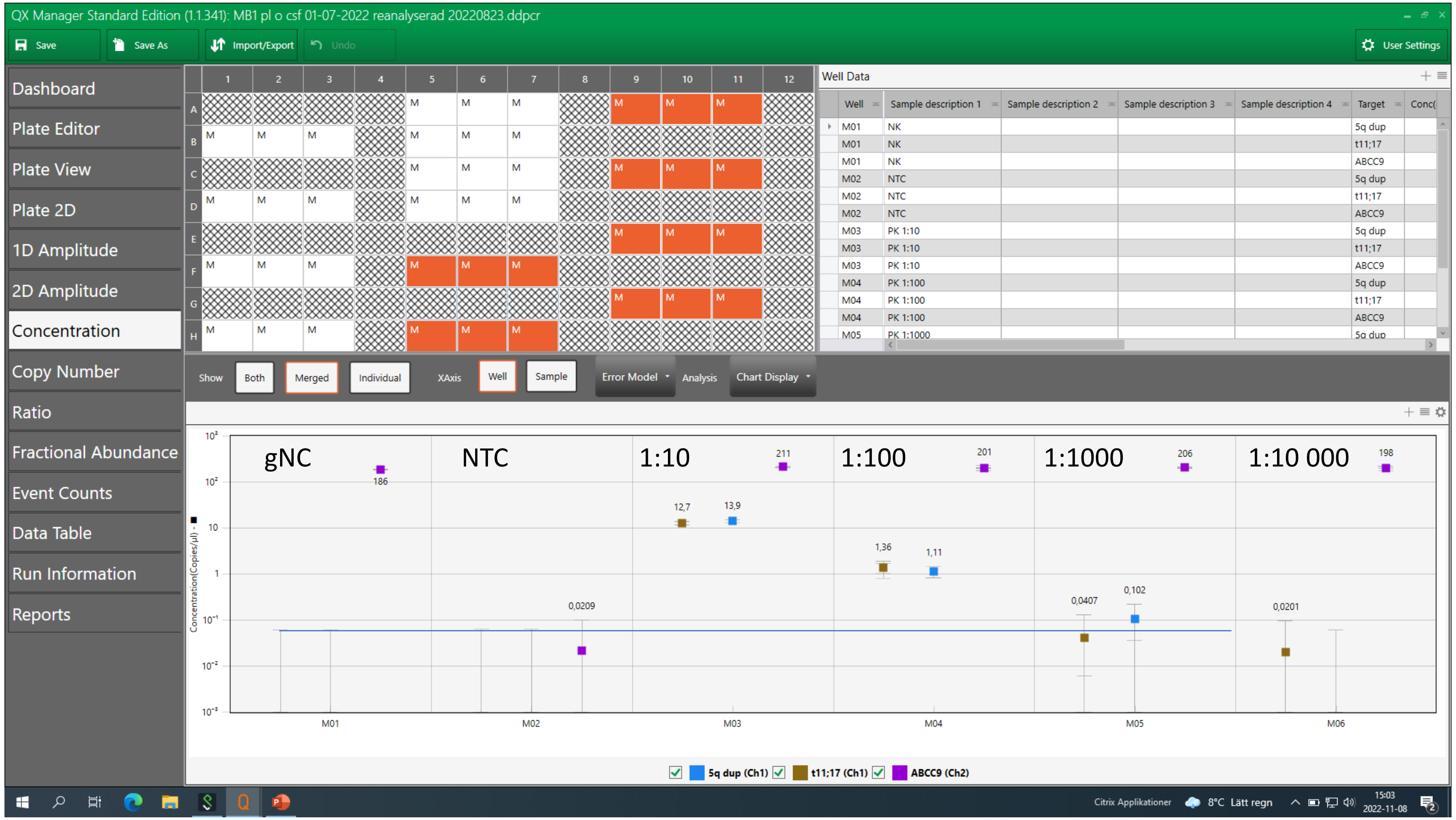

MB01 Concentration all liquid biopsies

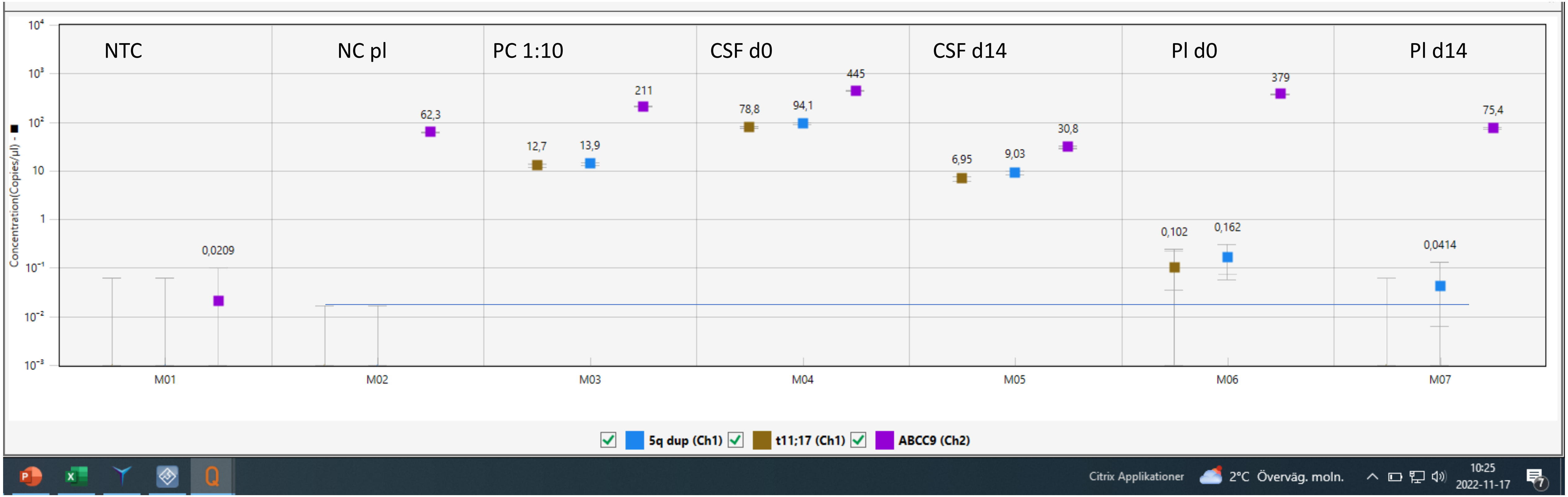

MB01 CSF d0

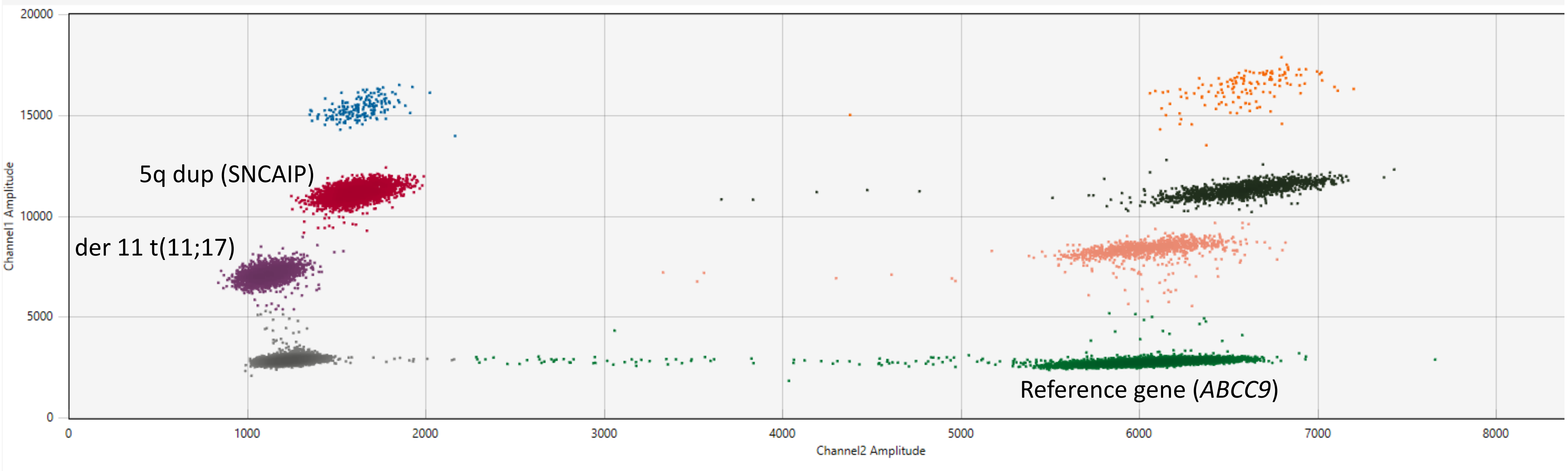

20000
15000
10000
5000
0
Channel1 Amplitude
5q dup (SNCAIP)
der 11 t(11;17)
Reference gene (*ABCC9*)
0
1000
2000
3000
4000
5000
6000
7000
8000
Channel2 Amplitude

MB01 PL d0

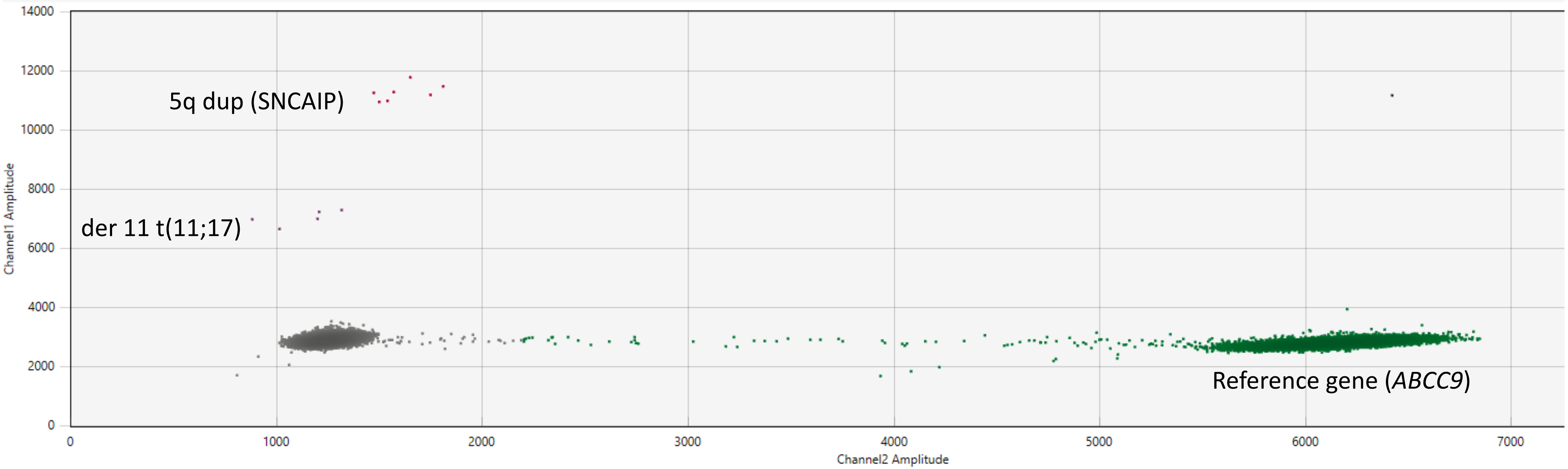
5q dup (SNCAIP)
der 11 t(11;17)
Reference gene (*ABCC9*)
Channel1 Amplitude
Channel2 Amplitude
14000
12000
10000
8000
6000
4000
2000
0
0
1000
2000
3000
4000
5000
6000
7000

MB01 CSF d14

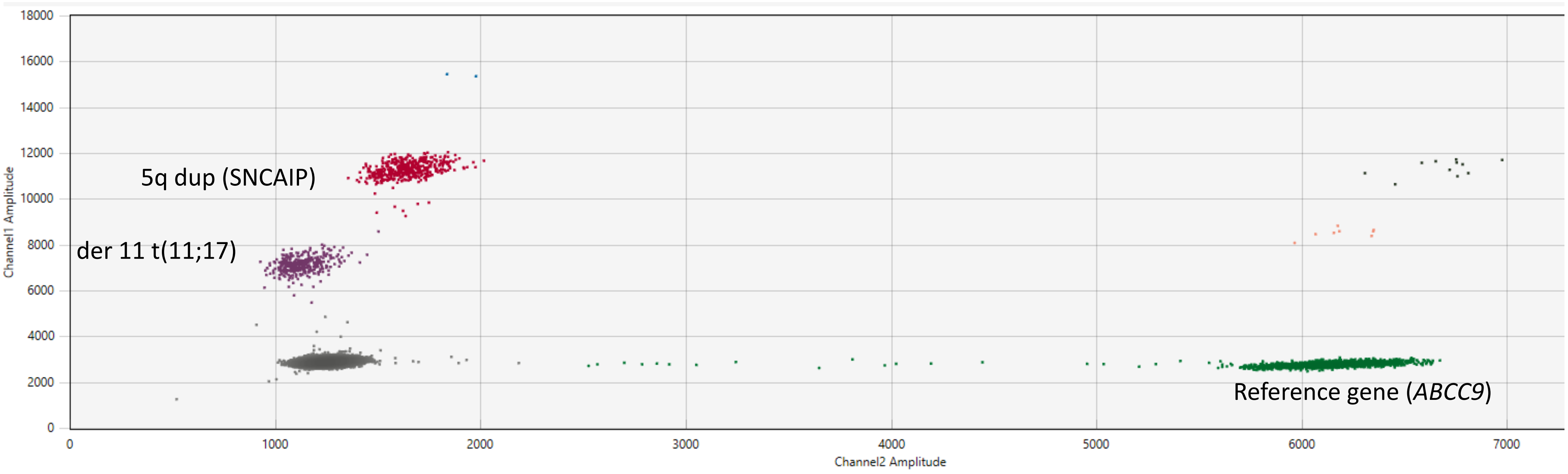

5q dup (SNCAIP)
der 11 t(11;17)
Reference gene (ABCC9)
Channel1 Amplitude
Channel2 Amplitude
0
2000
4000
6000
8000
10000
12000
14000
16000
18000
1000
2000
3000
4000
5000
6000
7000

MB01 PL d14

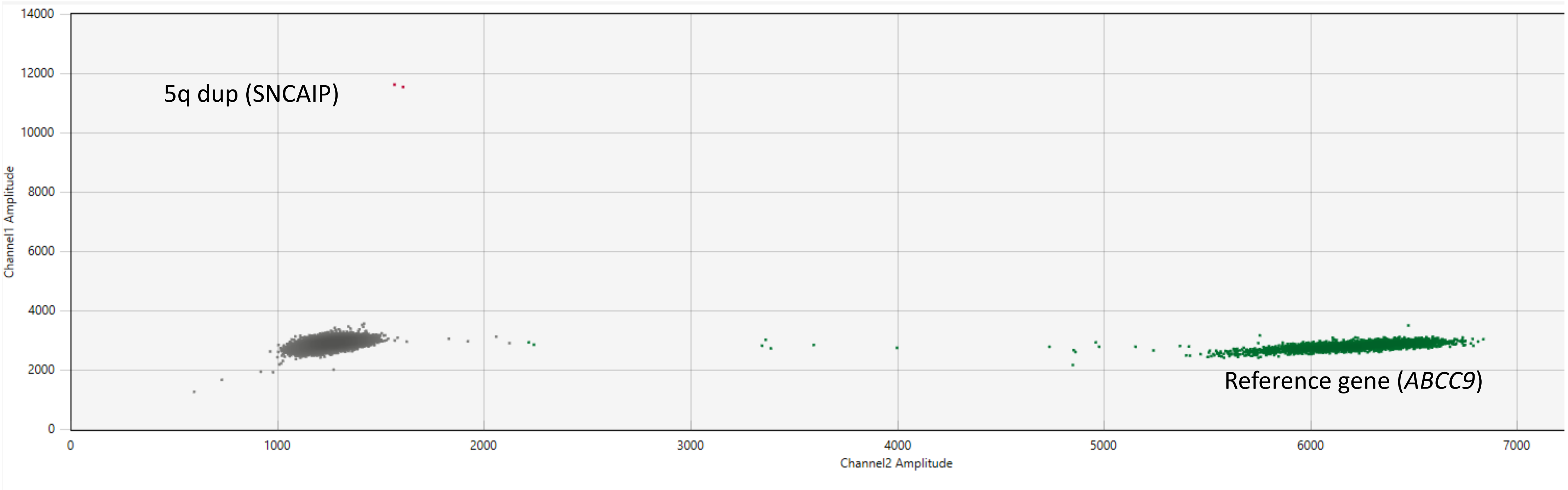
5q dup (SNCAIP)
Reference gene (ABCC9)
Channel1 Amplitude
Channel2 Amplitude
14000
12000
10000
8000
6000
4000
2000
0
1000
2000
3000
4000
5000
6000
7000
