# Supplementary material for: Simultaneous Ultra-Sensitive Detection of Structural and Single Nucleotide Variants Using Multiplex Droplet Digital PCR in Liquid Biopsies from Children with Medulloblastoma
Source: Cancers (Basel). 2023 Mar 25;15(7):1972. doi: 10.3390/cancers15071972 (PMC10092983; doi:10.3390/cancers15071972)
Supplement: Supplementary file 1 [file cancers-15-01972-s001.zip › File S2 QX Manager Software output data on liquid biopsies/Liquid biopsies MB09.pdf]

MB09 NTC

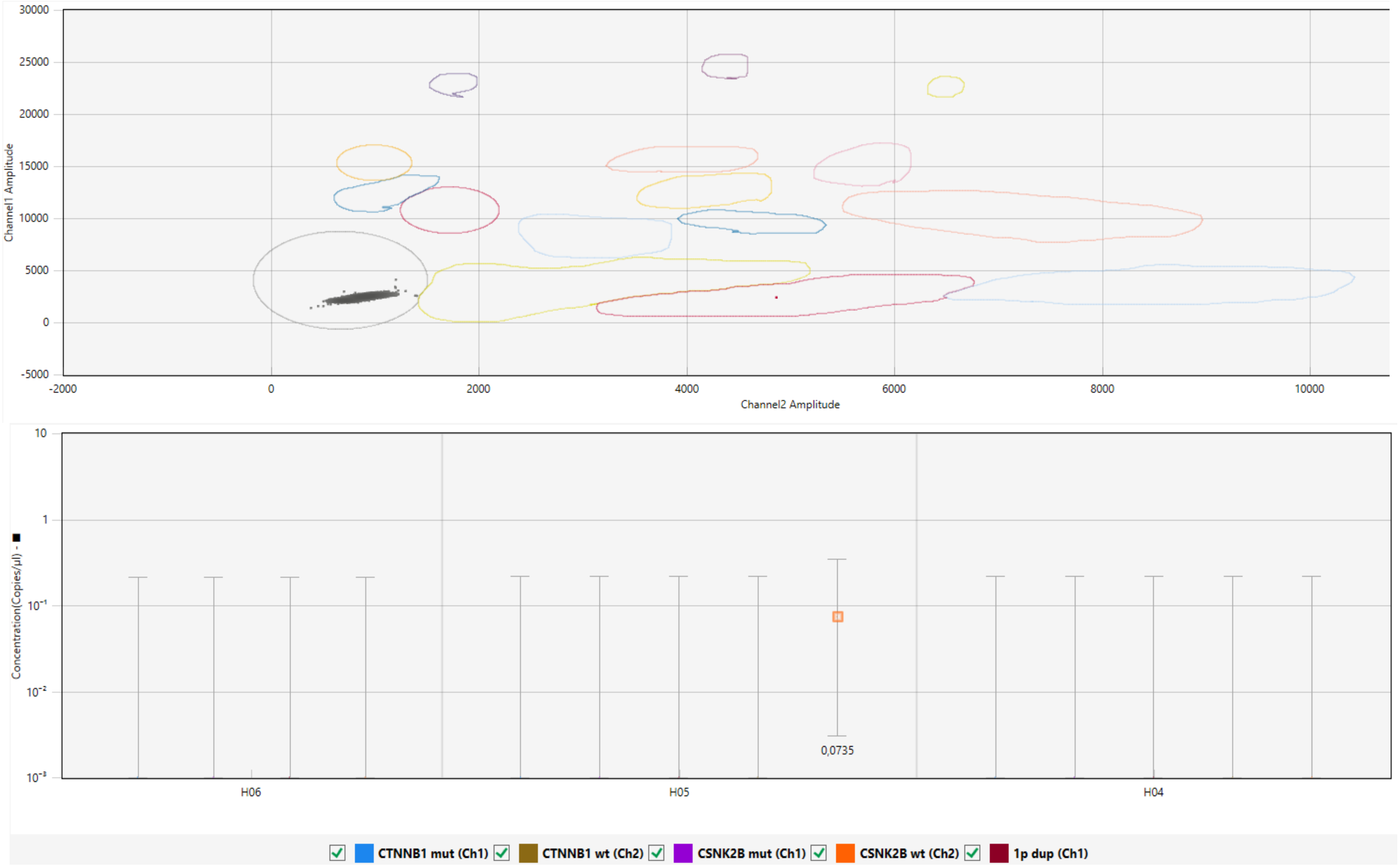

Channel1 Amplitude
Channel2 Amplitude
30000
25000
20000
15000
10000
5000
0
-5000
-2000
0
2000
4000
6000
8000
10000
Concentration(Copies/µl) -
10
1
10^{-1}
10^{-2}
10^{-3}
0,0735
H06
H05
H04
CTNNB1 mut (Ch1)
CTNNB1 wt (Ch2)
CSNK2B mut (Ch1)
CSNK2B wt (Ch2)
1p dup (Ch1)

MB09 NC pl & gNC

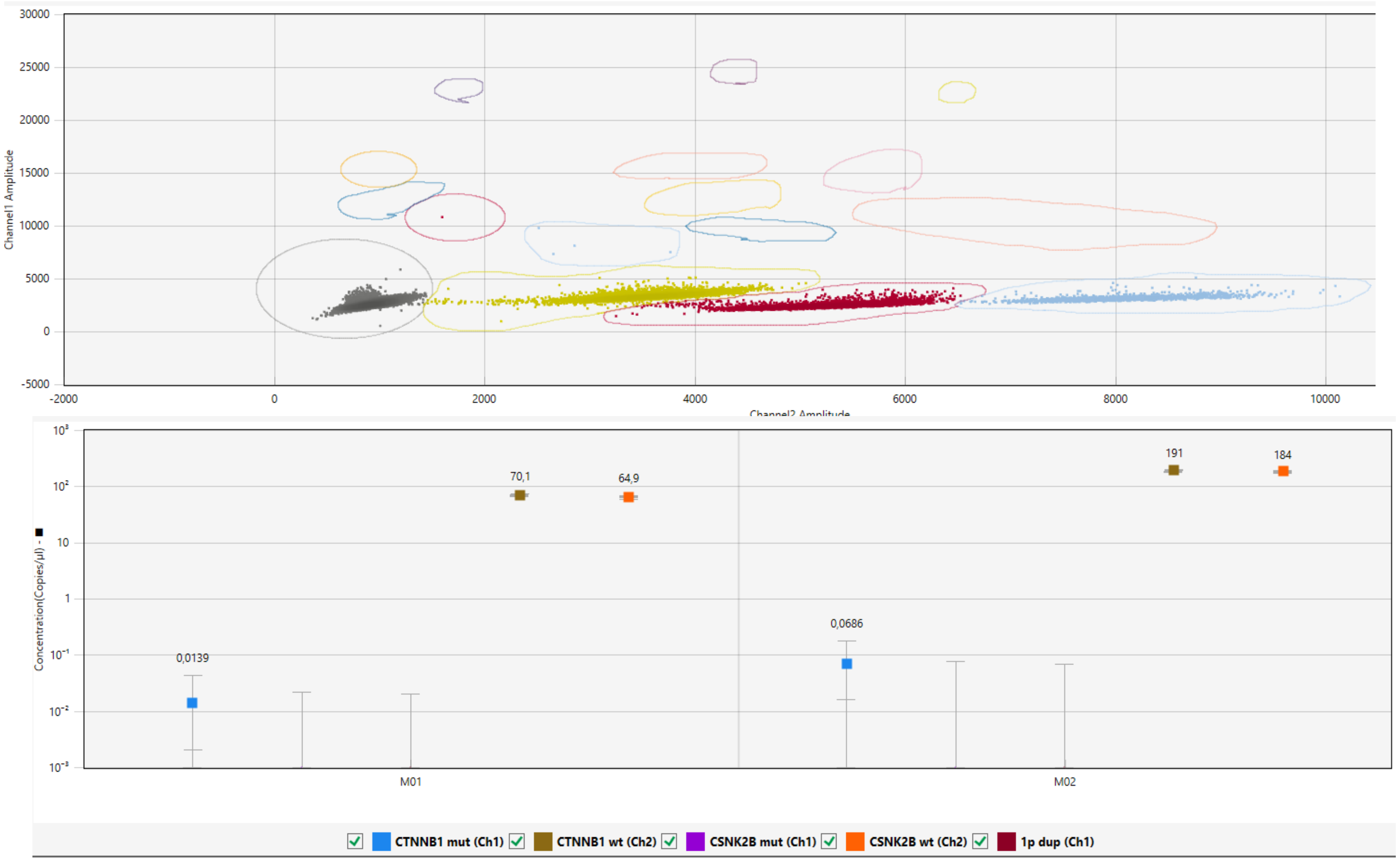

MB09 Example of clusters (PC 1:10)

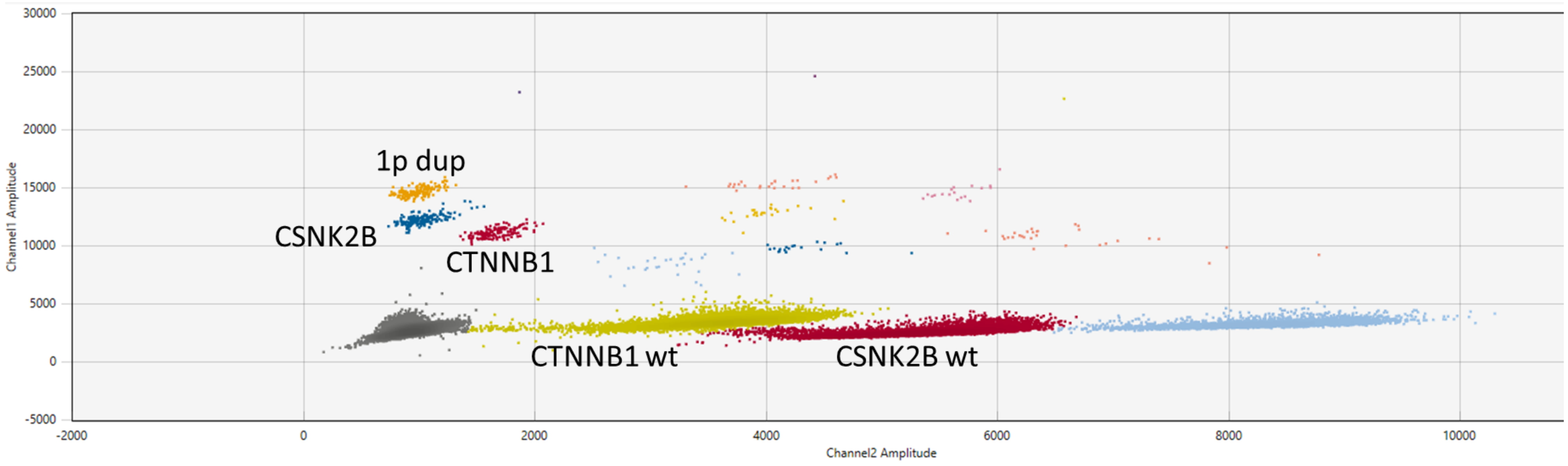

MB09 Dilution series: LoD & LoQ 1:100 both targets

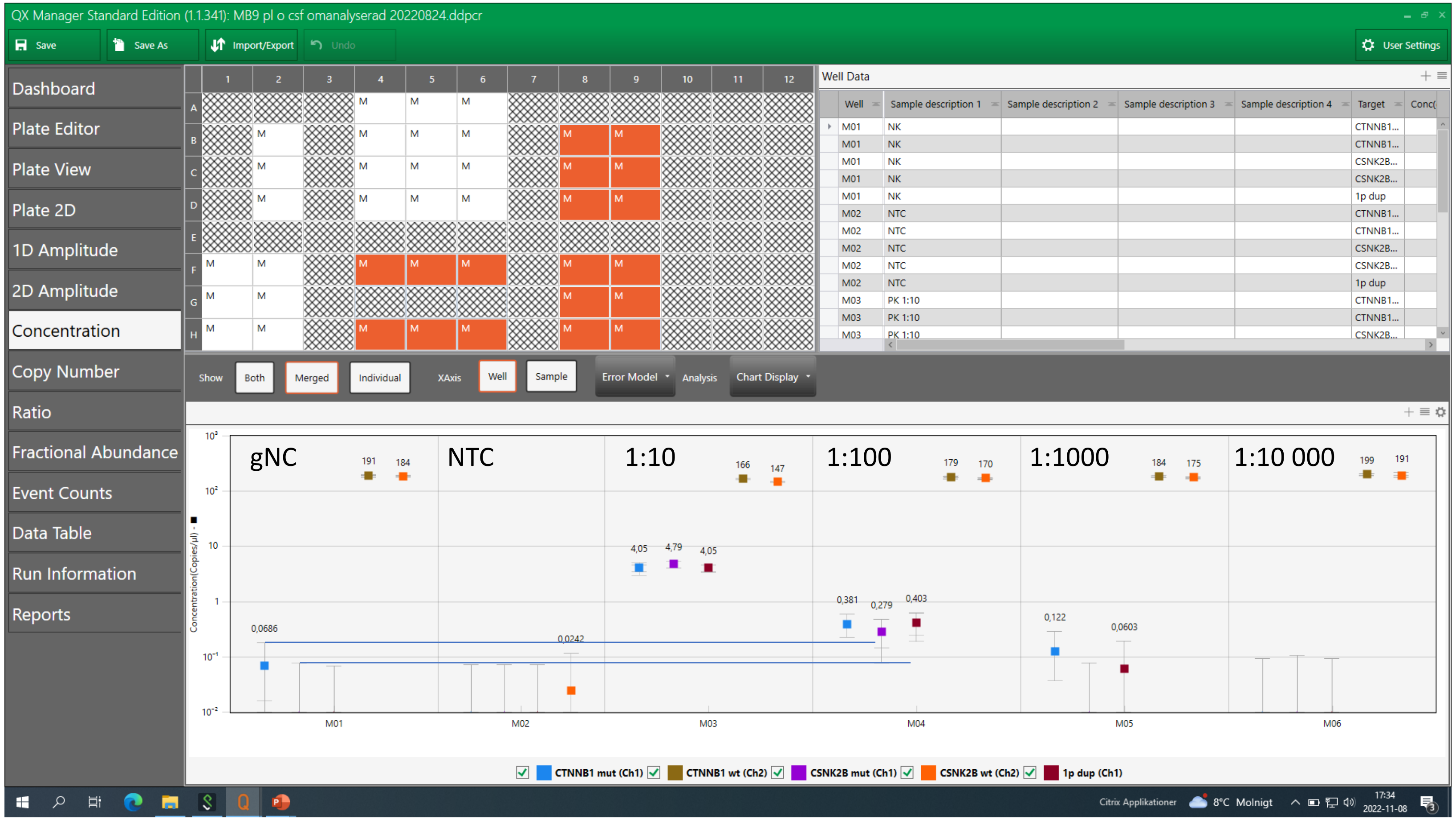

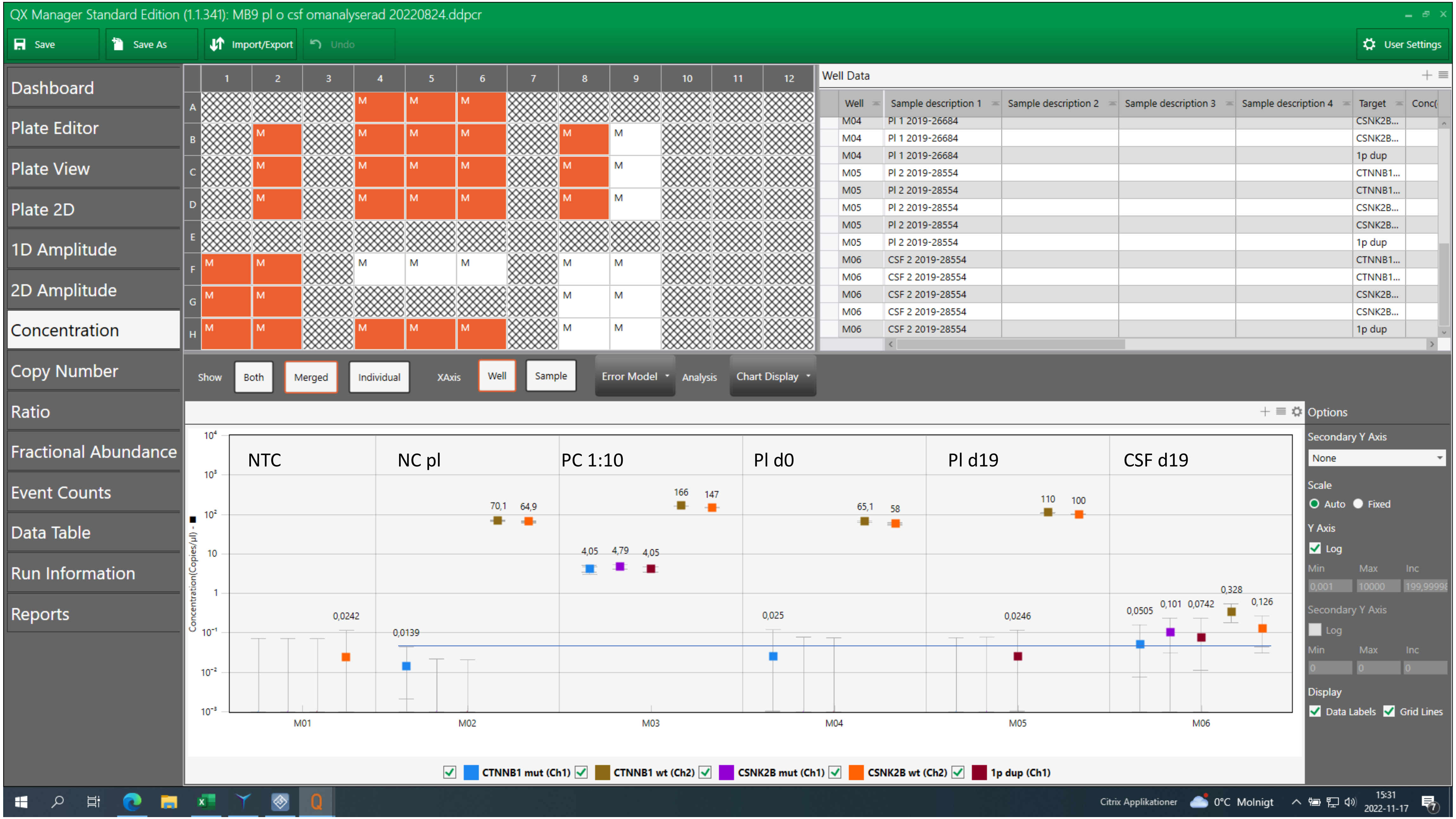
QX Manager Standard Edition (1.1.341): MB9 pl o csf omanalyserad 20220824.ddpcr
Save
Save As
Import/Export
Undo
User Settings
Dashboard
Plate Editor
Plate View
Plate 2D
1D Amplitude
2D Amplitude
Concentration
Copy Number
Ratio
Fractional Abundance
Event Counts
Data Table
Run Information
Reports
Well Data
Well | Sample description 1 | Sample description 2 | Sample description 3 | Sample description 4 | Target | Conc(
M04 | Pl 1 2019-26684 | CSNK2B...
M04 | Pl 1 2019-26684 | CSNK2B...
M04 | Pl 1 2019-26684 | 1p dup
M05 | Pl 2 2019-28554 | CTNNB1...
M05 | Pl 2 2019-28554 | CTNNB1...
M05 | Pl 2 2019-28554 | CSNK2B...
M05 | Pl 2 2019-28554 | CSNK2B...
M05 | Pl 2 2019-28554 | 1p dup
M06 | CSF 2 2019-28554 | CTNNB1...
M06 | CSF 2 2019-28554 | CTNNB1...
M06 | CSF 2 2019-28554 | CSNK2B...
M06 | CSF 2 2019-28554 | CSNK2B...
M06 | CSF 2 2019-28554 | 1p dup
Show
Both
Merged
Individual
XAxis
Well
Sample
Error Model
Analysis
Chart Display
NTC
NC pl
PC 1:10
Pl d0
Pl d19
CSF d19
70,1
64,9
166
147
4,05
4,79
4,05
65,1
58
110
100
0,0242
0,0139
0,025
0,0246
0,0505
0,101
0,0742
0,328
0,126
Concentration(Copies/µl)
M01
M02
M03
M04
M05
M06
CTNNB1 mut (Ch1)
CTNNB1 wt (Ch2)
CSNK2B mut (Ch1)
CSNK2B wt (Ch2)
1p dup (Ch1)
Options
Secondary Y Axis
None
Scale
Auto
Fixed
Y Axis
Log
Min
Max
Inc
0,001
10000
Secondary Y Axis
Log
Display
Data Labels
Grid Lines
Citrix Applikationer
0°C Molnigt
15:31
2022-11-17

MB09 PL d0

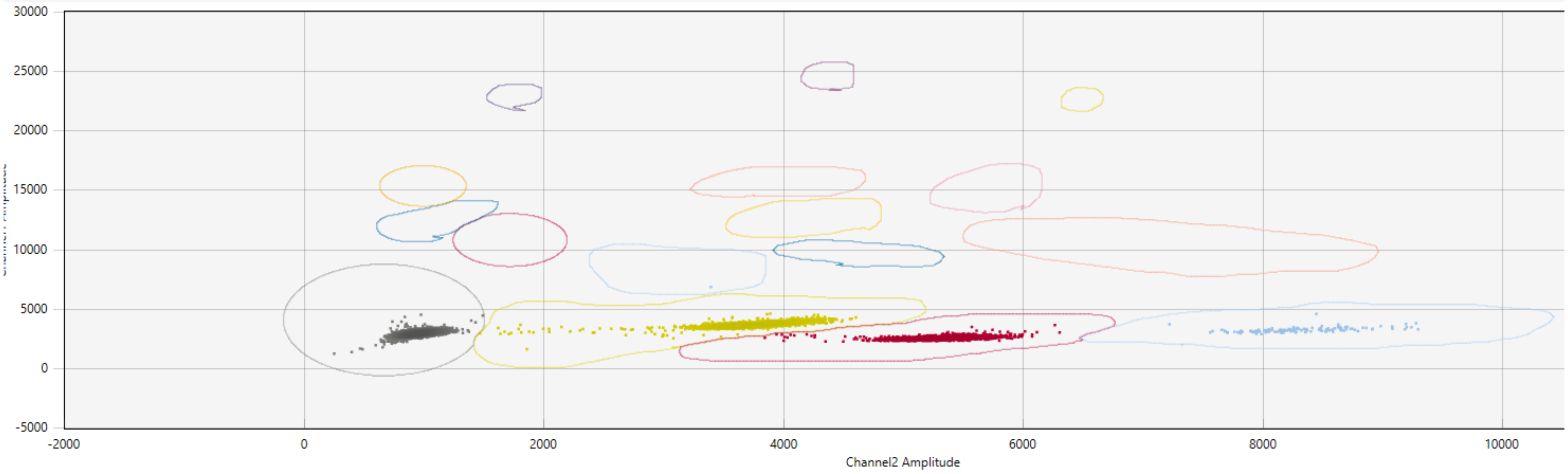

30000
25000
20000
15000
10000
5000
0
-5000
-2000
0
2000
4000
6000
8000
10000
Channel2 Amplitude

MB09 PL d19

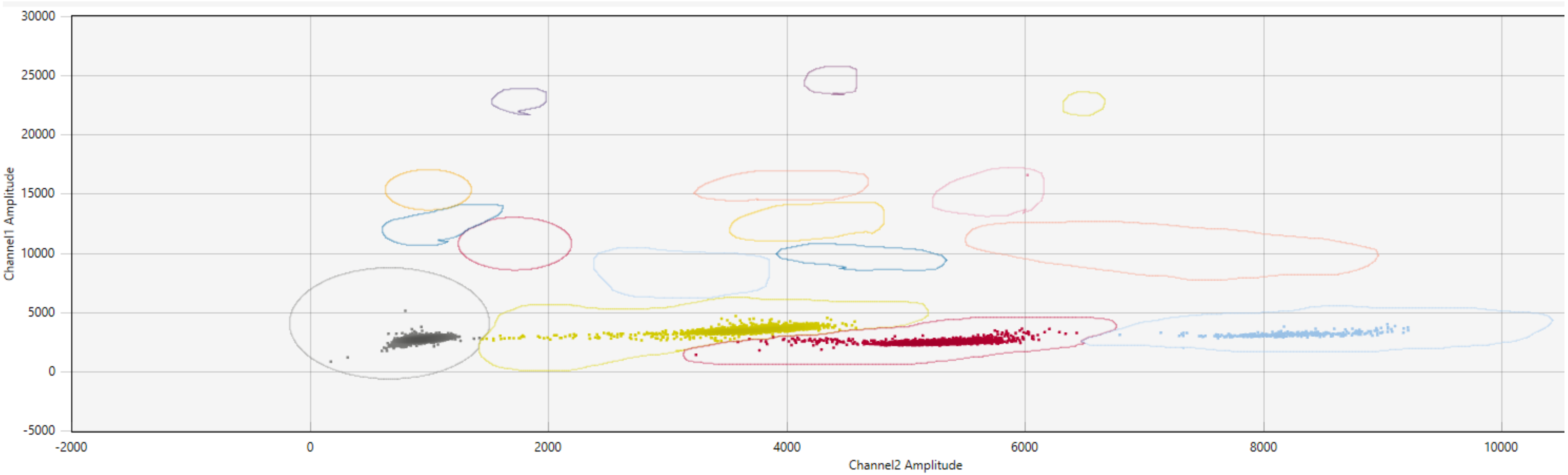

30000
25000
20000
15000
10000
5000
0
-5000
Channel1 Amplitude
-2000
0
2000
4000
6000
8000
10000
Channel2 Amplitude

MB09 CSF d19

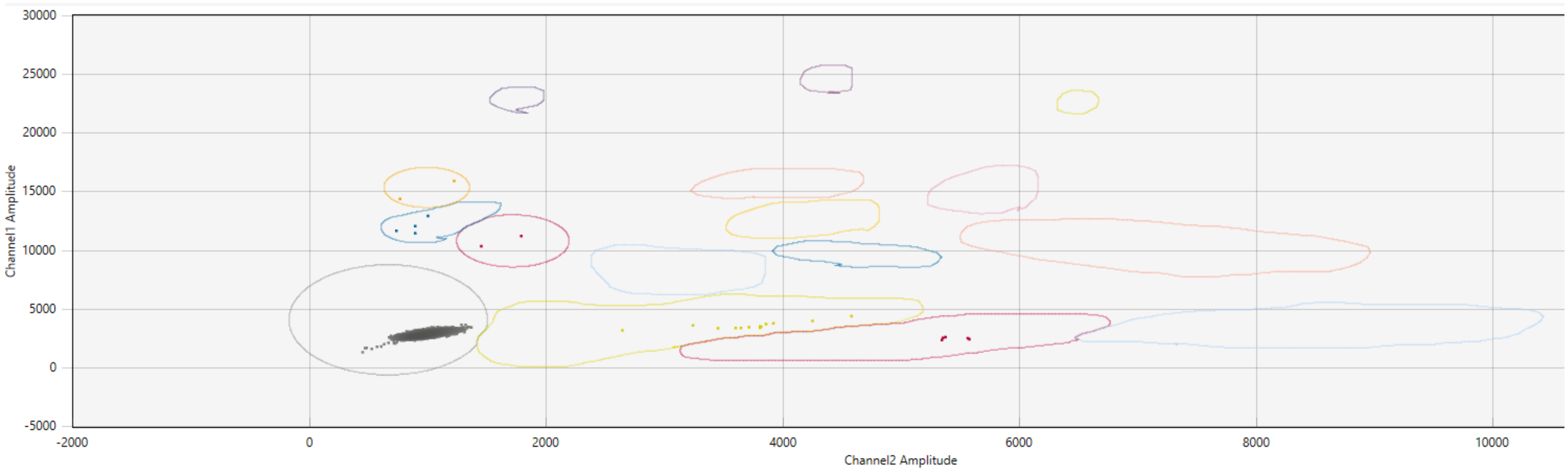

30000
25000
20000
15000
10000
5000
0
-5000
Channel1 Amplitude
-2000
0
2000
4000
6000
8000
10000
Channel2 Amplitude
