# Supplementary material for: Simultaneous Ultra-Sensitive Detection of Structural and Single Nucleotide Variants Using Multiplex Droplet Digital PCR in Liquid Biopsies from Children with Medulloblastoma
Source: Cancers (Basel). 2023 Mar 25;15(7):1972. doi: 10.3390/cancers15071972 (PMC10092983; doi:10.3390/cancers15071972)
Supplement: Supplementary file 1 [file cancers-15-01972-s001.zip › File S2 QX Manager Software output data on liquid biopsies/LIquid biopsies MB13.pdf]

MB13 NTC

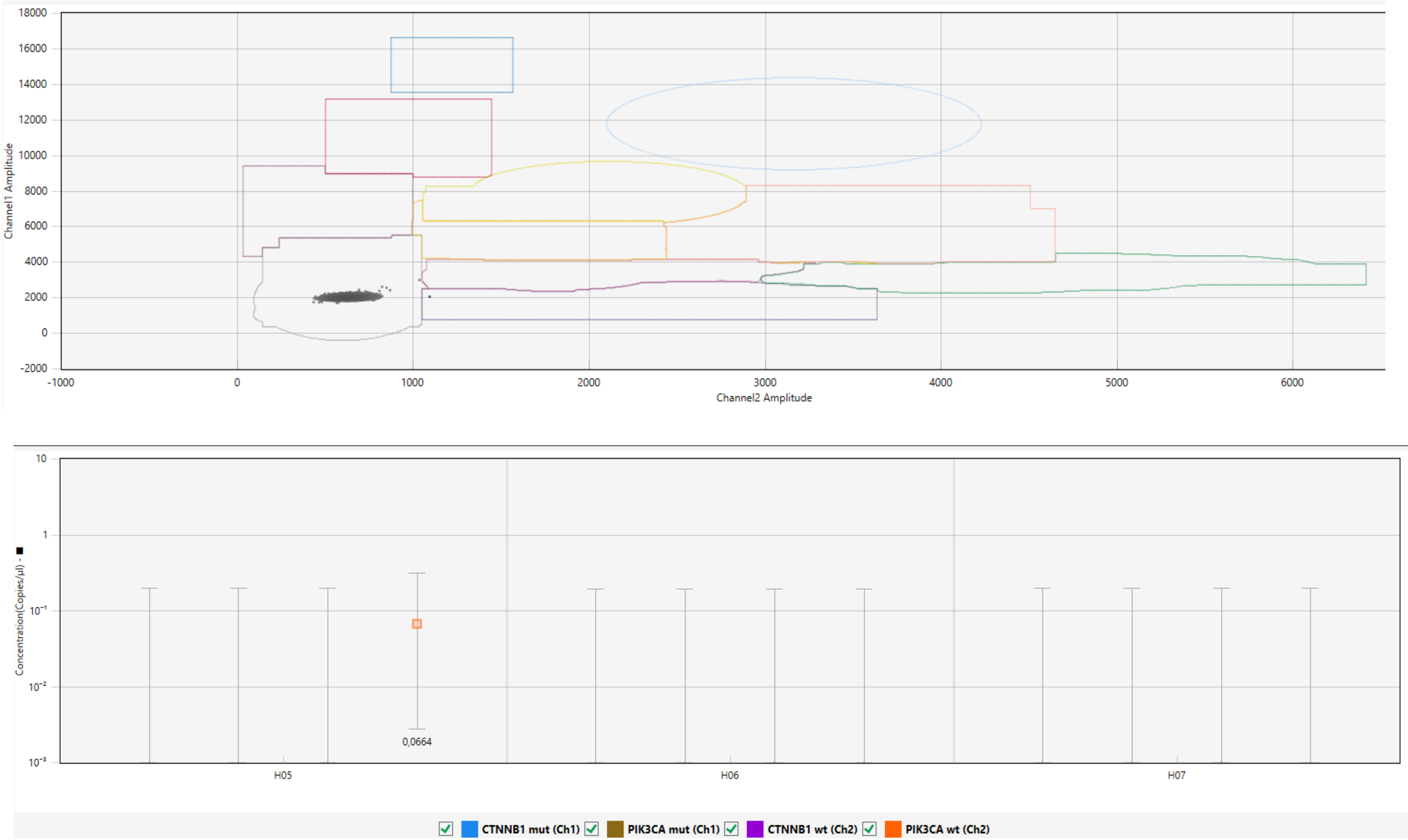
Channel1 Amplitude
Channel2 Amplitude
Concentration(Copies/µl)
0,0664
H05
H06
H07
CTNNB1 mut (Ch1)
PIK3CA mut (Ch1)
CTNNB1 wt (Ch2)
PIK3CA wt (Ch2)

MB13 NC pl & gNC

- Background in CTNNB1

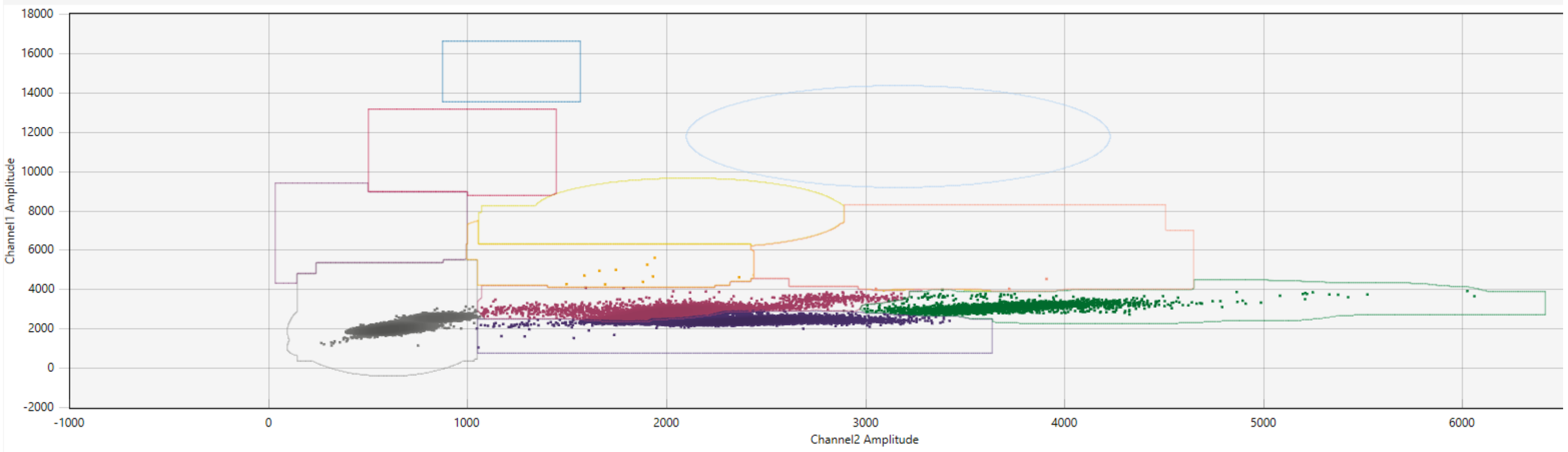

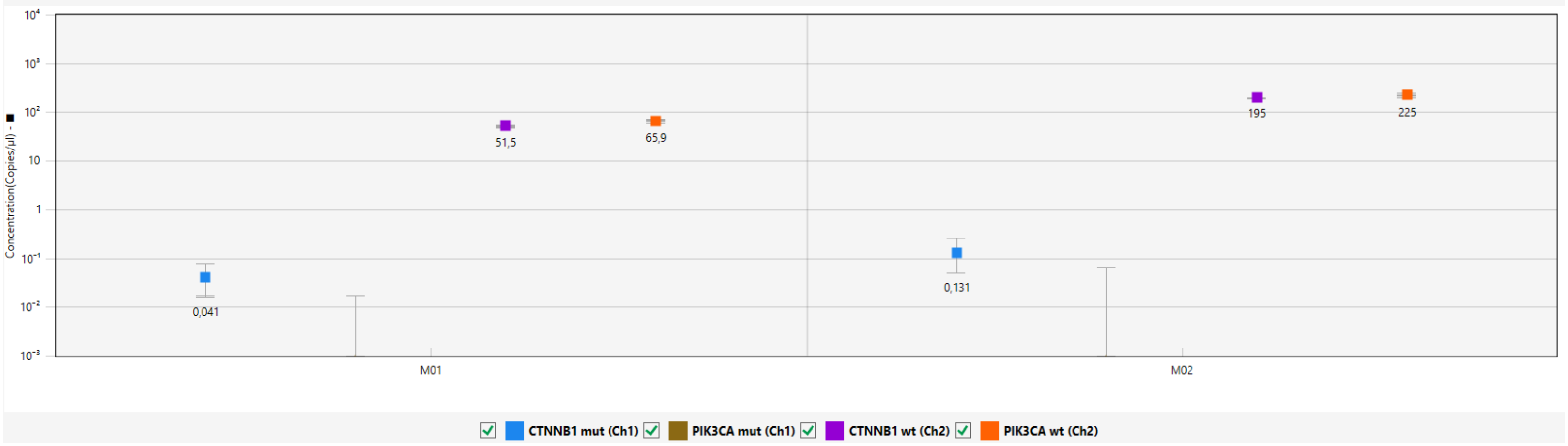

MB13 Example of clusters (PC 1:10)

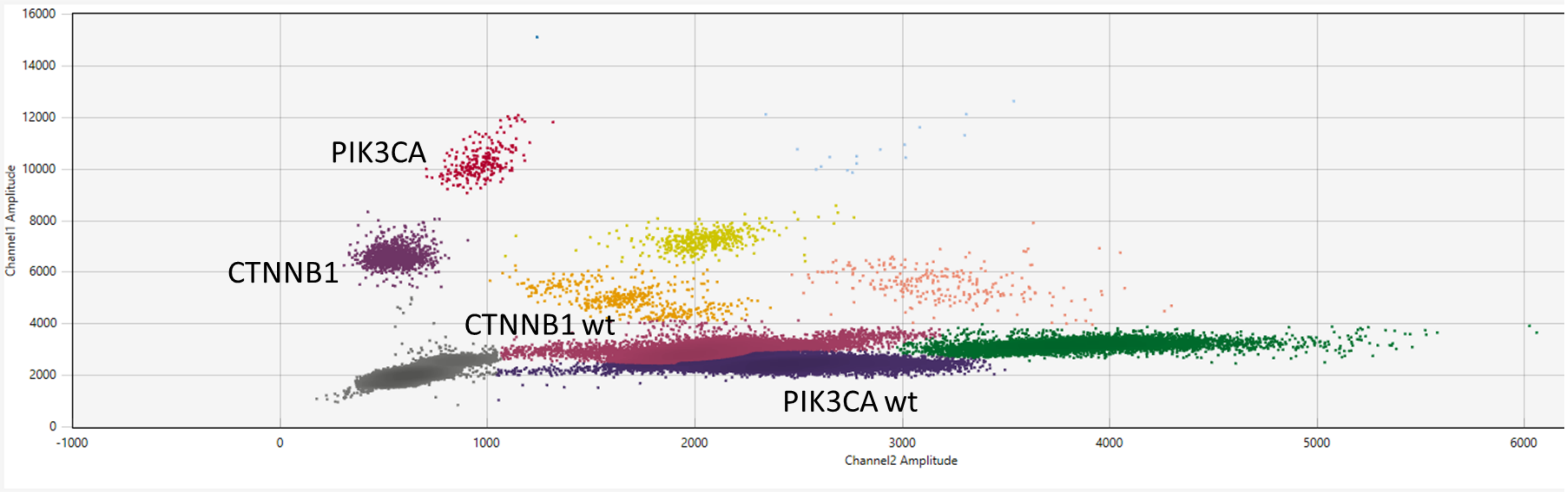

MB13 Dilution series: LoD & LoQ 1:100

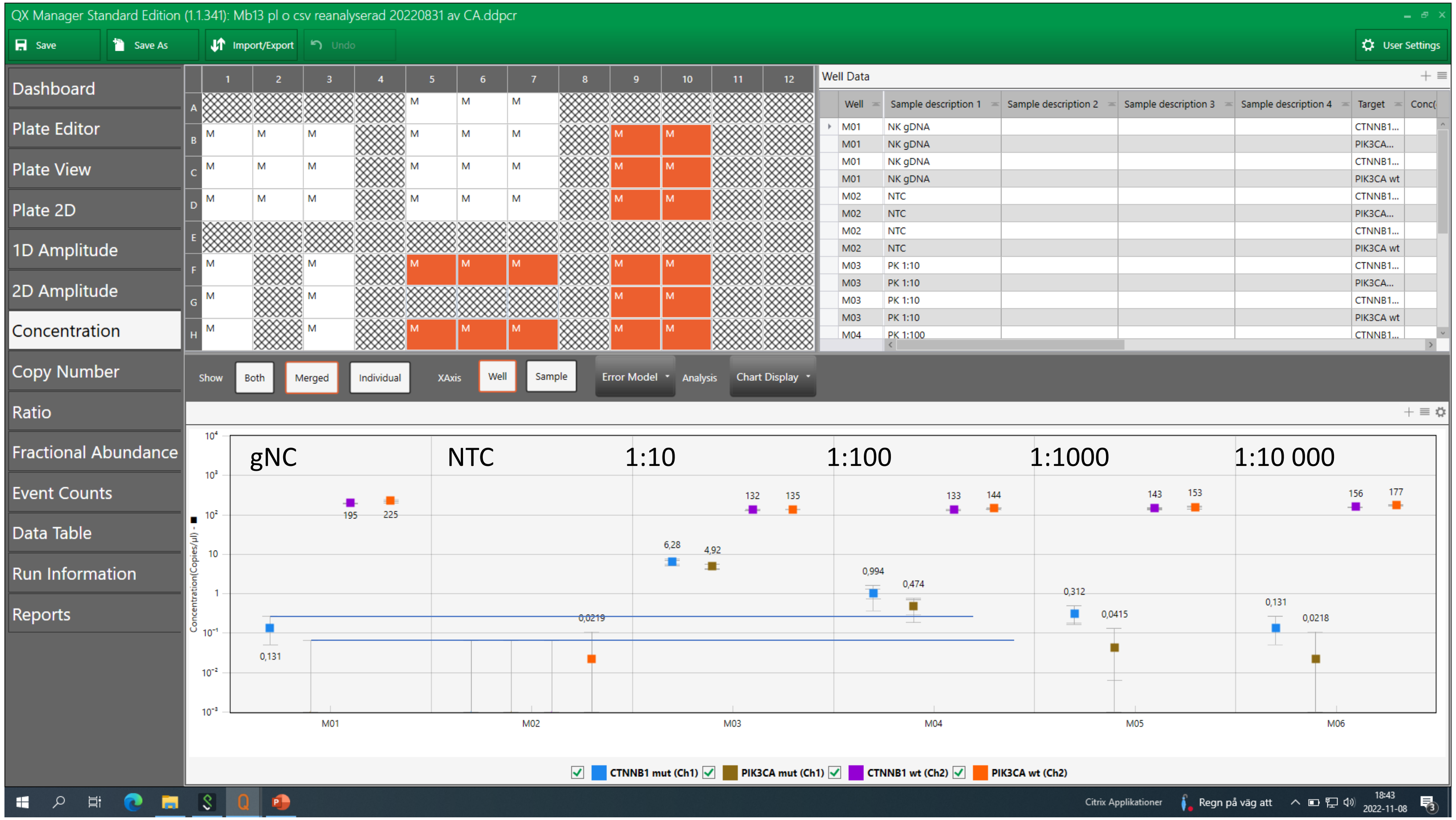

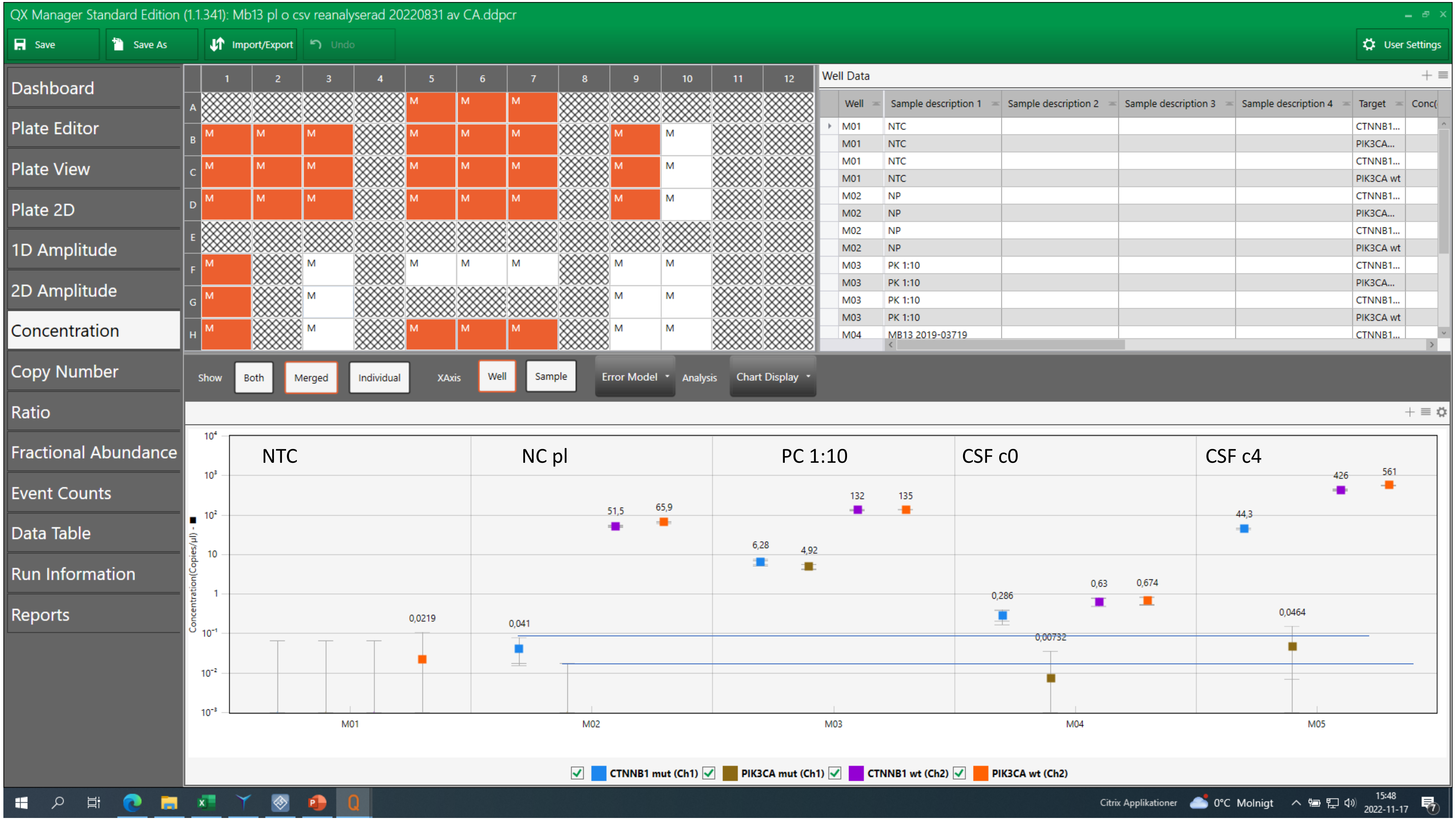
QX Manager Standard Edition (1.1.341): Mb13 pl o csv reanalyserad 20220831 av CA.ddpcr
Save
Save As
Import/Export
Undo
User Settings
Dashboard
Plate Editor
Plate View
Plate 2D
1D Amplitude
2D Amplitude
Concentration
Copy Number
Ratio
Fractional Abundance
Event Counts
Data Table
Run Information
Reports
Well Data
Well
Sample description 1
Sample description 2
Sample description 3
Sample description 4
Target
M01 NTC CTNNB1...
M01 NTC PIK3CA...
M01 NTC CTNNB1...
M01 NTC PIK3CA wt
M02 NP CTNNB1...
M02 NP PIK3CA...
M02 NP CTNNB1...
M02 NP PIK3CA wt
M03 PK 1:10 CTNNB1...
M03 PK 1:10 PIK3CA...
M03 PK 1:10 CTNNB1...
M03 PK 1:10 PIK3CA wt
M04 MB13 2019-03719 CTNNB1...
Show
Both
Merged
Individual
XAxis
Well
Sample
Error Model
Analysis
Chart Display
NTC
NC pl
PC 1:10
CSF c0
CSF c4
Concentration(Copies/µl)
0,0219
0,041
51,5
65,9
6,28
4,92
132
135
0,286
0,00732
0,63
0,674
44,3
426
561
0,0464
M01
M02
M03
M04
M05
CTNNB1 mut (Ch1)
PIK3CA mut (Ch1)
CTNNB1 wt (Ch2)
PIK3CA wt (Ch2)
Citrix Applikationer
0°C Molnigt
15:48
2022-11-17

MB13 CSF d0

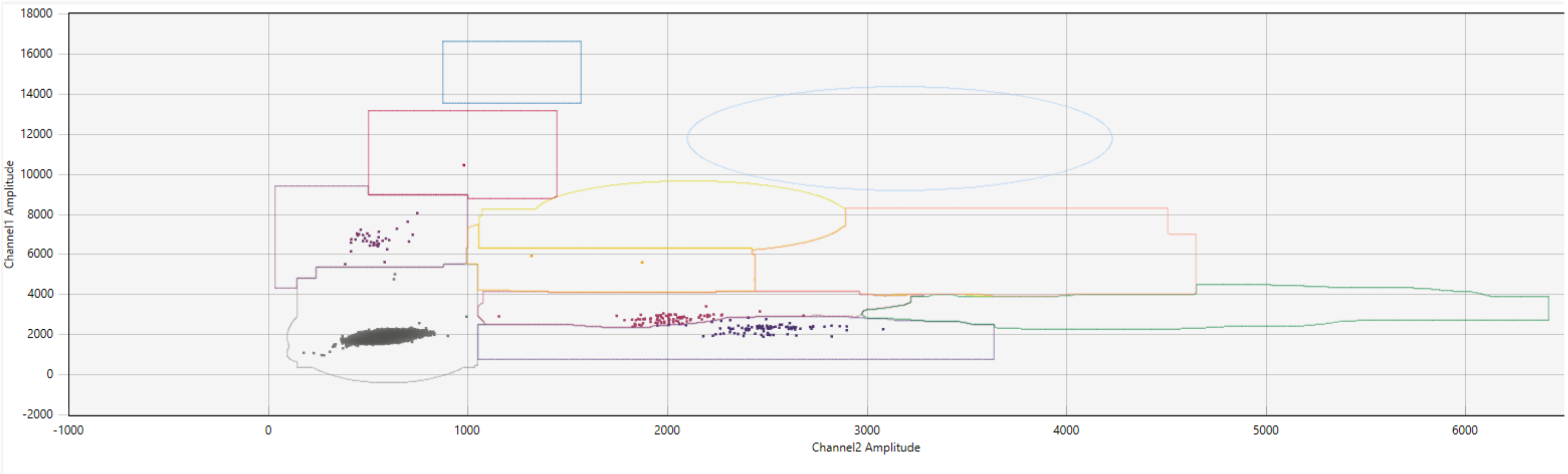

18000
16000
14000
12000
10000
8000
6000
4000
2000
0
-2000
Channel1 Amplitude
-1000
0
1000
2000
3000
4000
5000
6000
Channel2 Amplitude

MB13 CSF d4

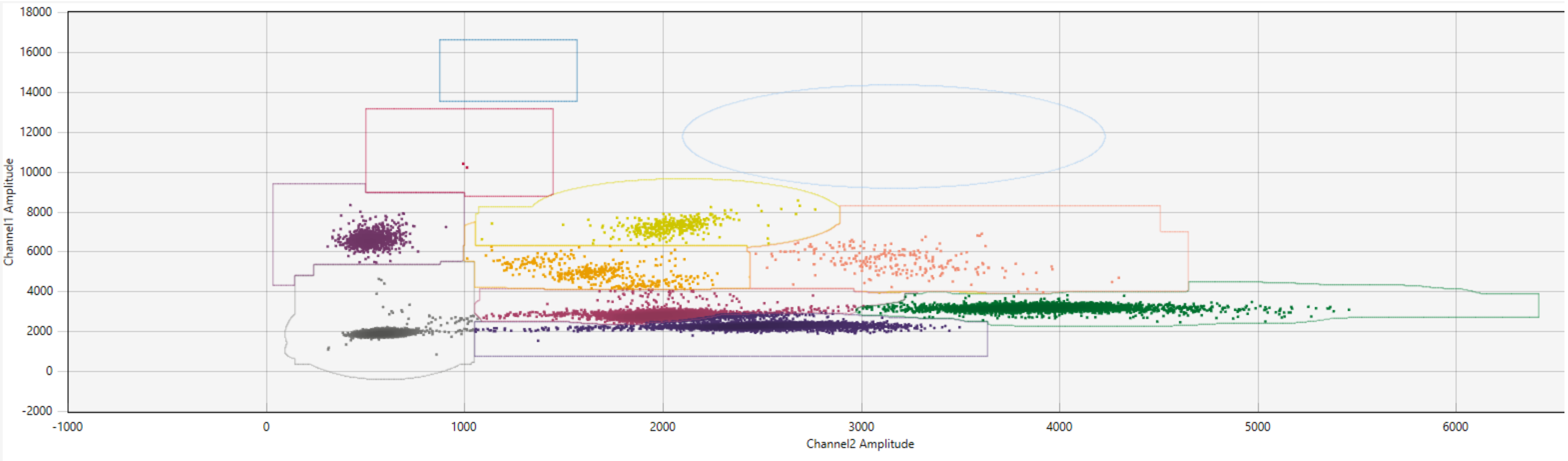

18000
16000
14000
12000
10000
8000
6000
4000
2000
0
-2000
Channel1 Amplitude
-1000
0
1000
2000
3000
4000
5000
6000
Channel2 Amplitude
