# Supplementary material for: Simultaneous Ultra-Sensitive Detection of Structural and Single Nucleotide Variants Using Multiplex Droplet Digital PCR in Liquid Biopsies from Children with Medulloblastoma
Source: Cancers (Basel). 2023 Mar 25;15(7):1972. doi: 10.3390/cancers15071972 (PMC10092983; doi:10.3390/cancers15071972)
Supplement: Supplementary file 1 [file cancers-15-01972-s001.zip › File S2 QX Manager Software output data on liquid biopsies/Liquid biopsies MB15.pdf]

MB15 NTC

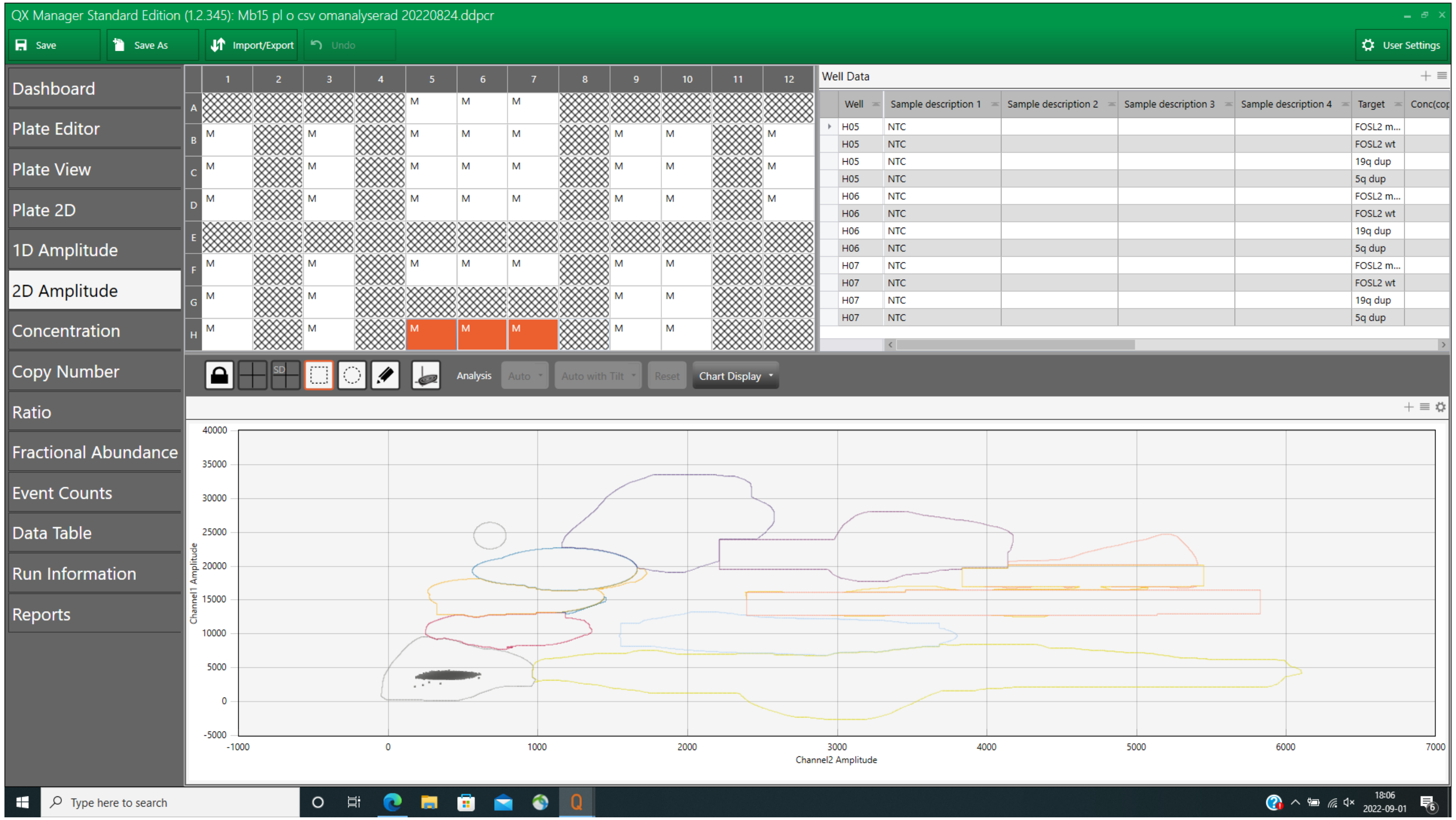
QX Manager Standard Edition (1.2.345): Mb15 pl o csv omanalyserad 20220824.ddpcr
Save
Save As
Import/Export
Undo
User Settings
Dashboard
Plate Editor
Plate View
Plate 2D
1D Amplitude
2D Amplitude
Concentration
Copy Number
Ratio
Fractional Abundance
Event Counts
Data Table
Run Information
Reports
Well Data
Well
Sample description 1
Sample description 2
Sample description 3
Sample description 4
Target
H05 NTC FOSL2 m...
H05 NTC FOSL2 wt
H05 NTC 19q dup
H05 NTC 5q dup
H06 NTC FOSL2 m...
H06 NTC FOSL2 wt
H06 NTC 19q dup
H06 NTC 5q dup
H07 NTC FOSL2 m...
H07 NTC FOSL2 wt
H07 NTC 19q dup
H07 NTC 5q dup
Analysis
Auto
Auto with Tilt
Reset
Chart Display
Channel1 Amplitude
Channel2 Amplitude
Type here to search
18:06
2022-09-01

MB15 NC

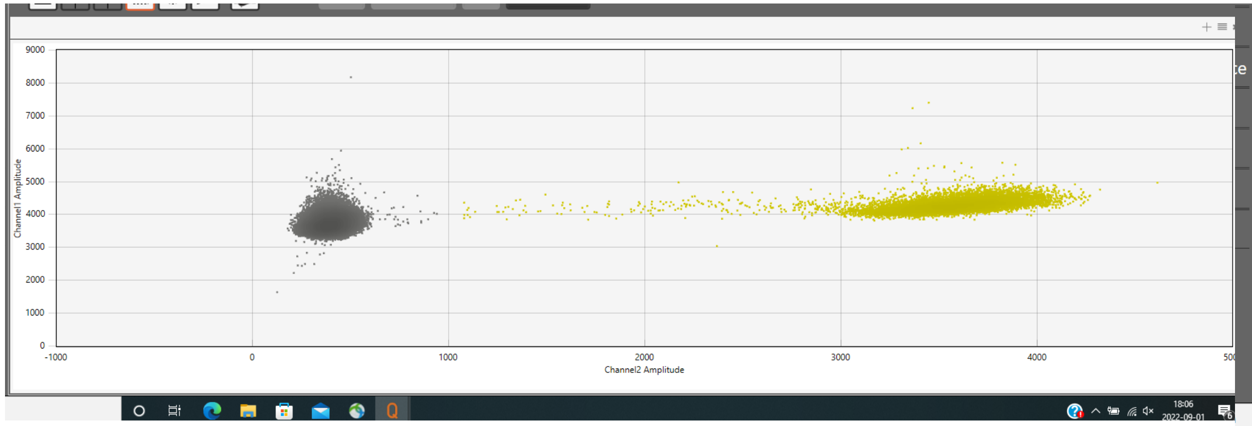
Channel1 Amplitude
Channel2 Amplitude
9000
8000
7000
6000
5000
4000
3000
2000
1000
0
-1000
0
1000
2000
3000
4000

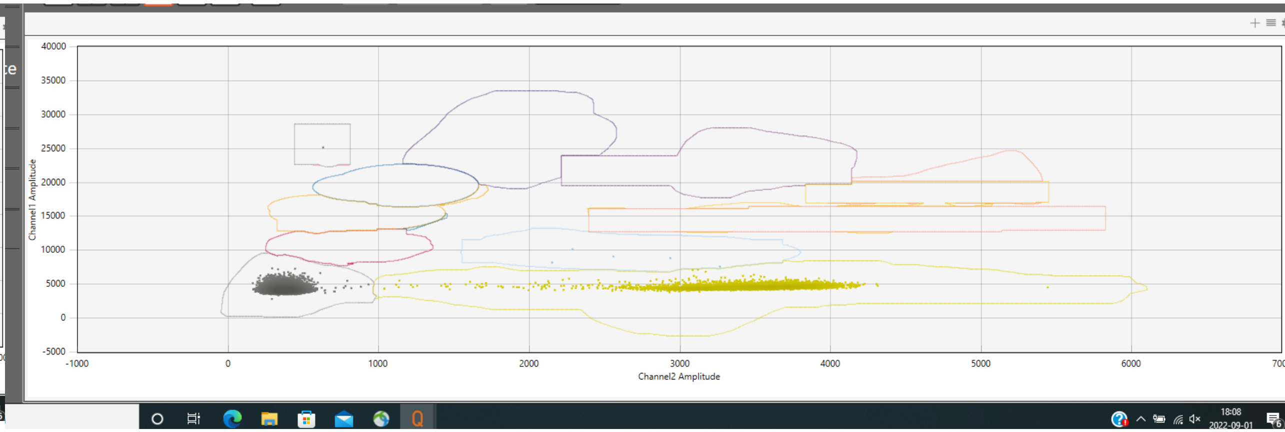
Channel1 Amplitude
Channel2 Amplitude
40000
35000
30000
25000
20000
15000
10000
5000
0
-5000
-1000
0
1000
2000
3000
4000
5000
6000

MB15 Example of clusters (PC 1:10)

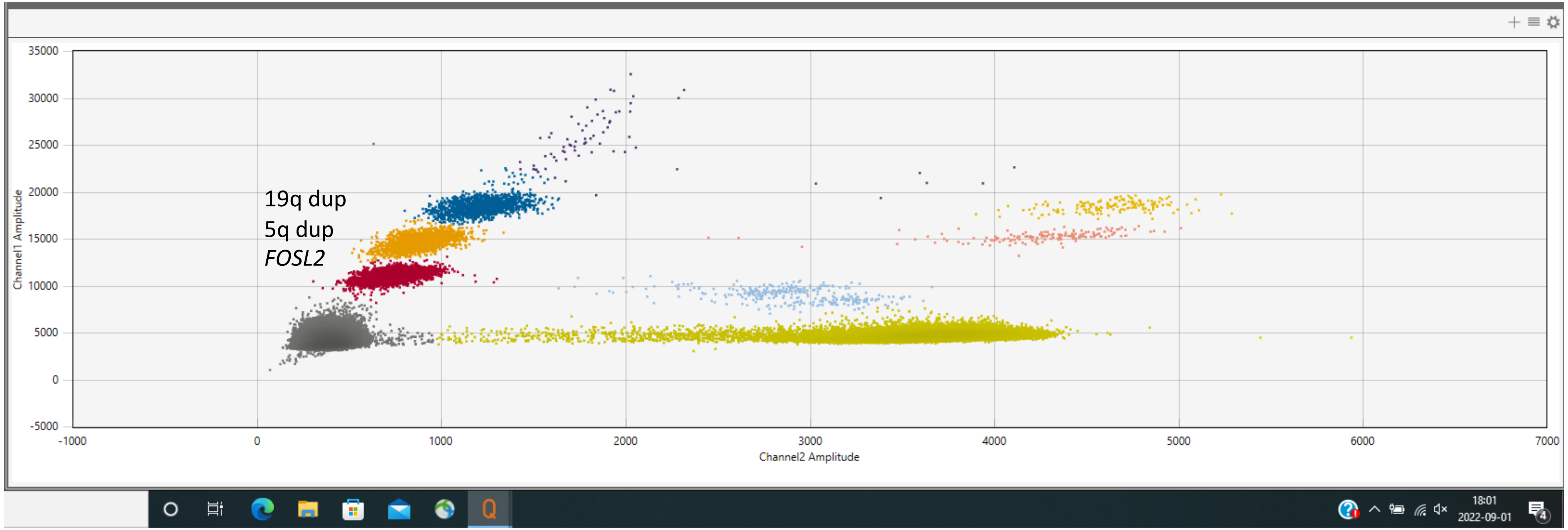

MB15 Dilution series: LoD 1:1000 for FOSL2, 1:100 for 5q dup and 19q dup. LoQ all 1:100.

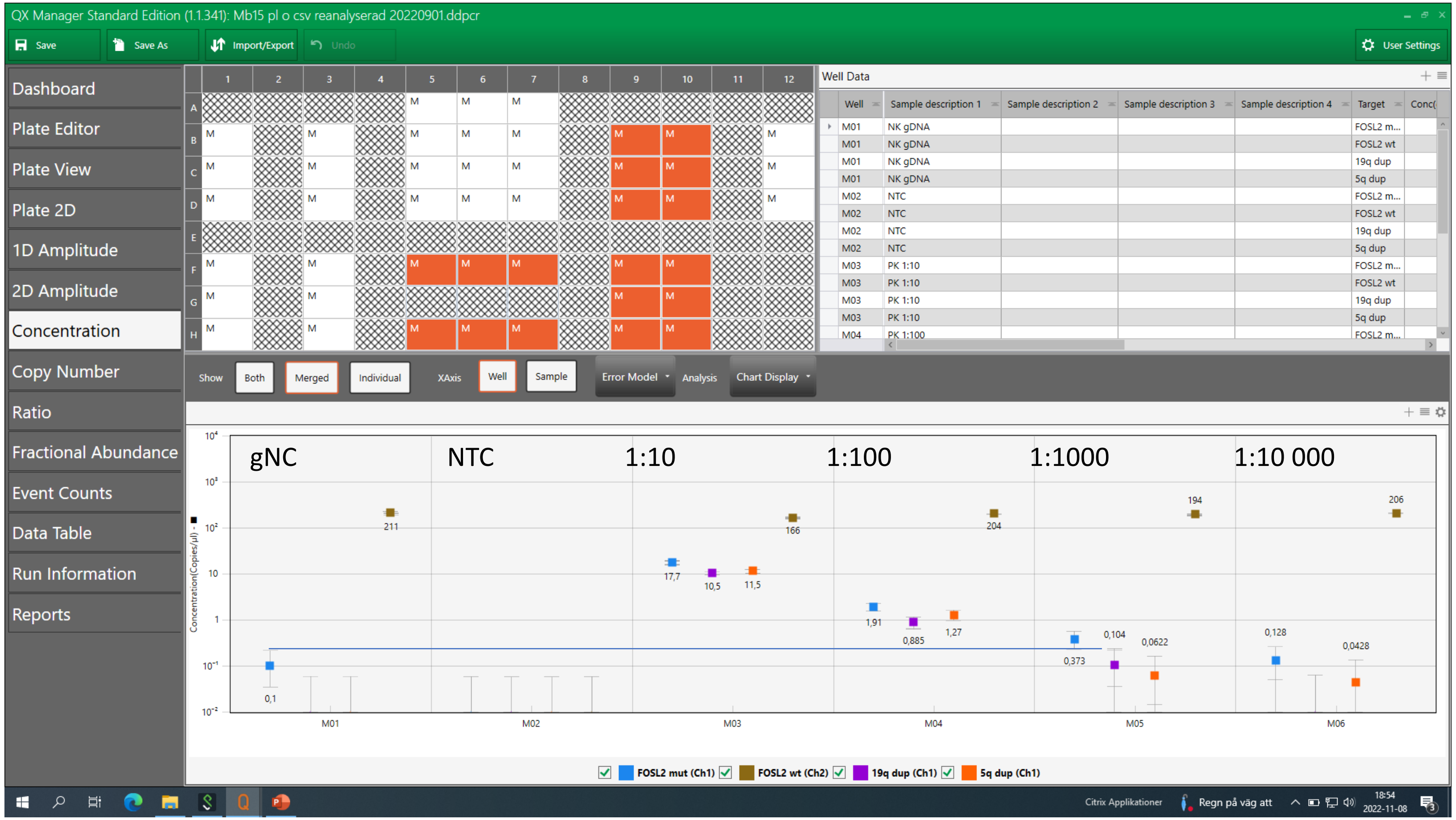

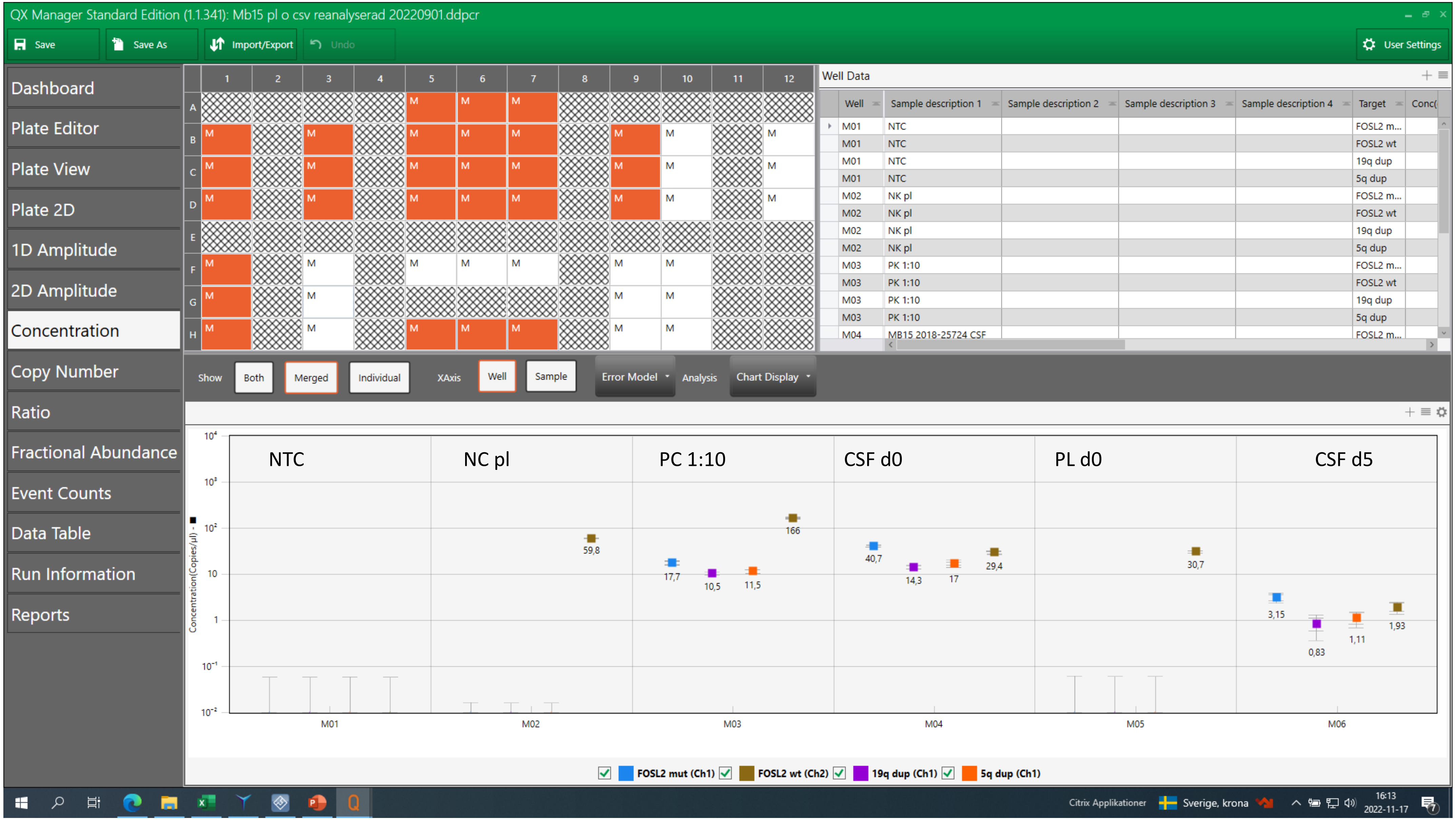
QX Manager Standard Edition (1.1.341): Mb15 pl o csv reanalyserad 20220901.ddpcr
Save
Save As
Import/Export
Undo
User Settings
Dashboard
Plate Editor
Plate View
Plate 2D
1D Amplitude
2D Amplitude
Concentration
Copy Number
Ratio
Fractional Abundance
Event Counts
Data Table
Run Information
Reports
Well Data
Well
Sample description 1
Sample description 2
Sample description 3
Sample description 4
Target
M01 NTC FOSL2 m...
M01 NTC FOSL2 wt
M01 NTC 19q dup
M01 NTC 5q dup
M02 NK pl FOSL2 m...
M02 NK pl FOSL2 wt
M02 NK pl 19q dup
M02 NK pl 5q dup
M03 PK 1:10 FOSL2 m...
M03 PK 1:10 FOSL2 wt
M03 PK 1:10 19q dup
M03 PK 1:10 5q dup
M04 MB15 2018-25724 CSF FOSL2 m...
Show
Both
Merged
Individual
XAxis
Well
Sample
Error Model
Analysis
Chart Display
NTC
NC pl
PC 1:10
CSF d0
PL d0
CSF d5
Concentration(Copies/µl)
59,8
17,7
10,5
11,5
166
40,7
14,3
17
29,4
30,7
3,15
0,83
1,11
1,93
M01
M02
M03
M04
M05
M06
FOSL2 mut (Ch1)
FOSL2 wt (Ch2)
19q dup (Ch1)
5q dup (Ch1)
Citrix Applikationer
Sverige, krona
16:13
2022-11-17

MB15 CSF d0

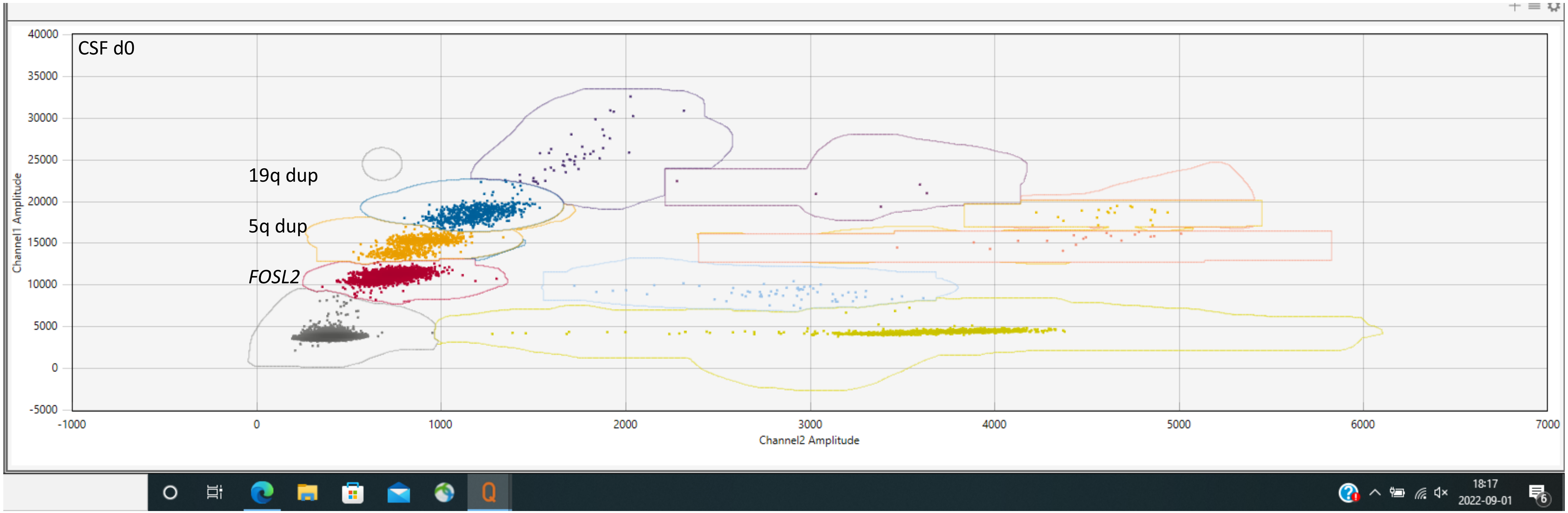

CSF d0
19q dup
5q dup
FOSL2
Channel1 Amplitude
Channel2 Amplitude
40000
35000
30000
25000
20000
15000
10000
5000
0
-5000
-1000
0
1000
2000
3000
4000
5000
6000
7000
18:17
2022-09-01

MB15 PL d0

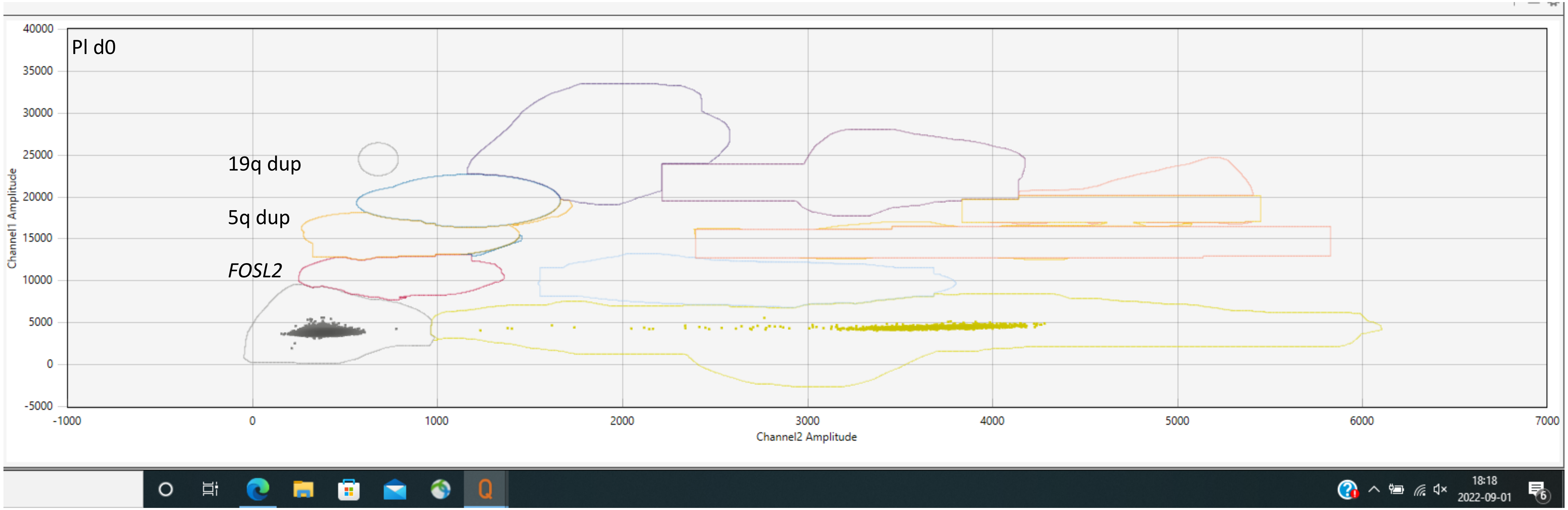
Pl d0
19q dup
5q dup
FOSL2
Channel1 Amplitude
Channel2 Amplitude
40000
35000
30000
25000
20000
15000
10000
5000
0
-5000
-1000
0
1000
2000
3000
4000
5000
6000
7000
18:18
2022-09-01

MB15 CSF d5

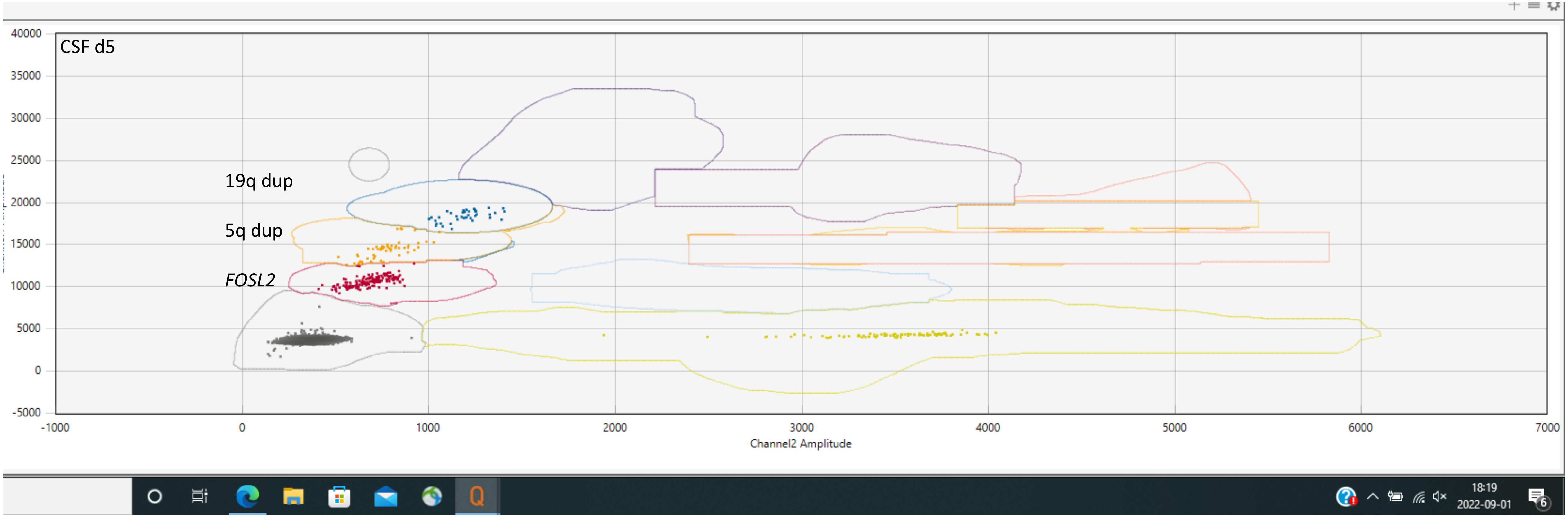

CSF d5
19q dup
5q dup
FOSL2
40000
35000
30000
25000
20000
15000
10000
5000
0
-5000
-1000
0
1000
2000
3000
4000
5000
6000
7000
Channel2 Amplitude
18:19
2022-09-01
