# Supplementary material for: Simultaneous Ultra-Sensitive Detection of Structural and Single Nucleotide Variants Using Multiplex Droplet Digital PCR in Liquid Biopsies from Children with Medulloblastoma
Source: Cancers (Basel). 2023 Mar 25;15(7):1972. doi: 10.3390/cancers15071972 (PMC10092983; doi:10.3390/cancers15071972)

MB19 NTC

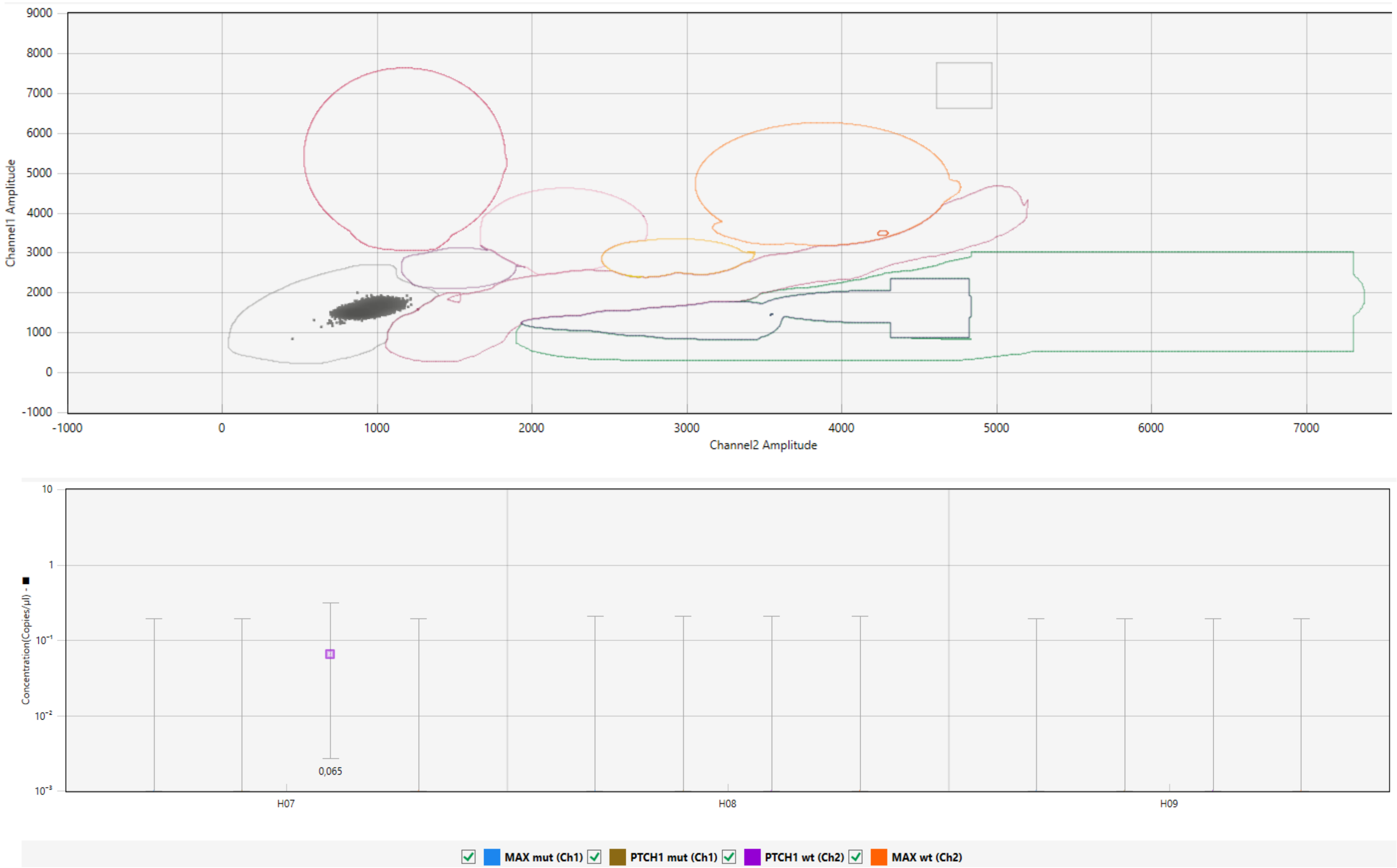
9000
8000
7000
6000
5000
4000
3000
2000
1000
0
-1000
Channel1 Amplitude
-1000
0
1000
2000
3000
4000
5000
6000
7000
Channel2 Amplitude
10
1
10^{-1}
10^{-2}
10^{-3}
Concentration(Copies/µl) -
0,065
H07
H08
H09
MAX mut (Ch1)
PTCH1 mut (Ch1)
PTCH1 wt (Ch2)
MAX wt (Ch2)

MB19 NC pl & gNC

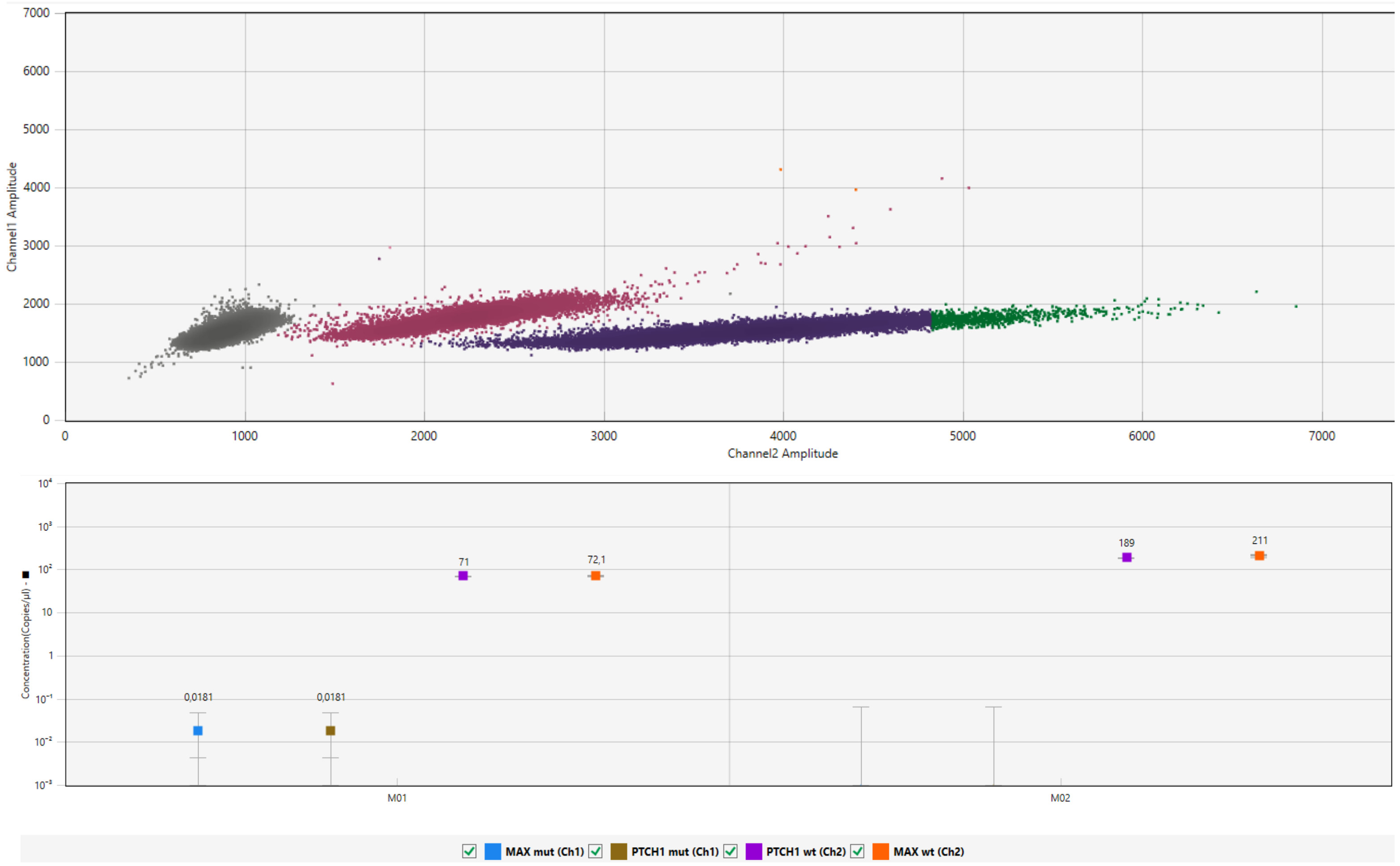

MB19 Example of clusters (PC 1:10)

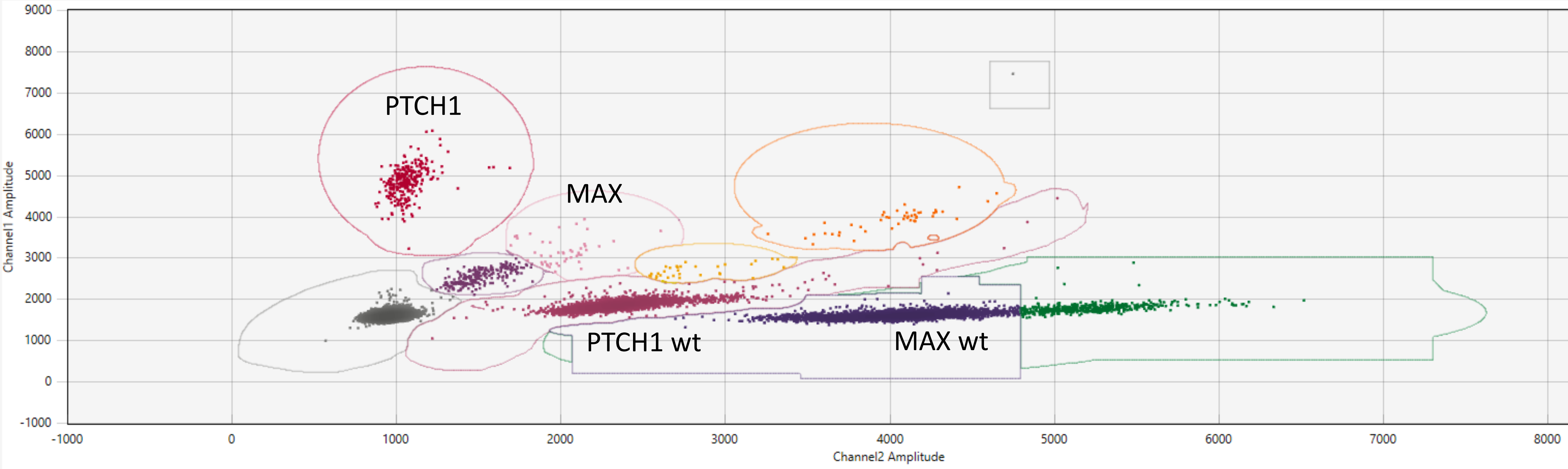

MB19 Dilution series: LoD & LoQ 1:100 both targets.

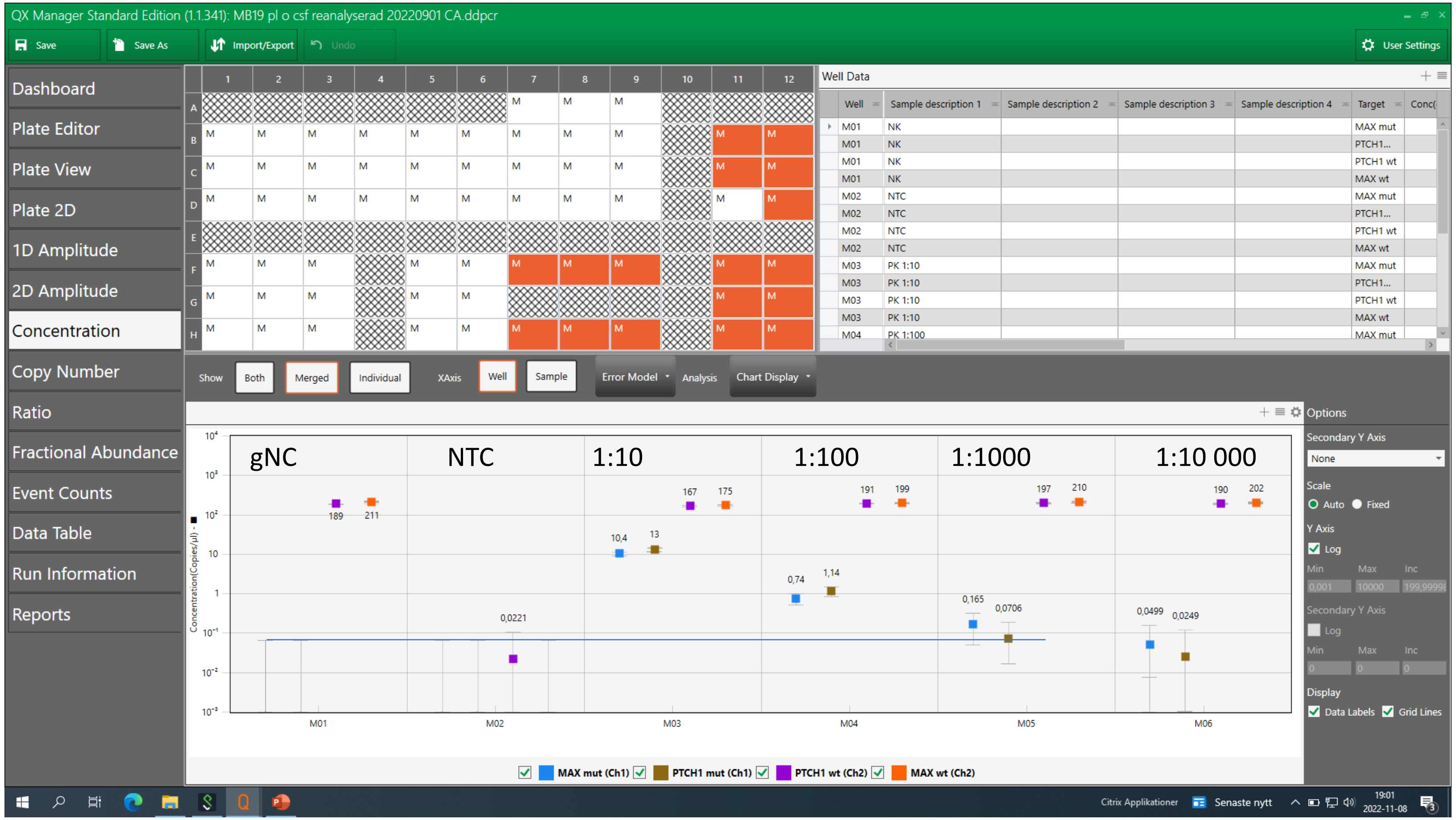

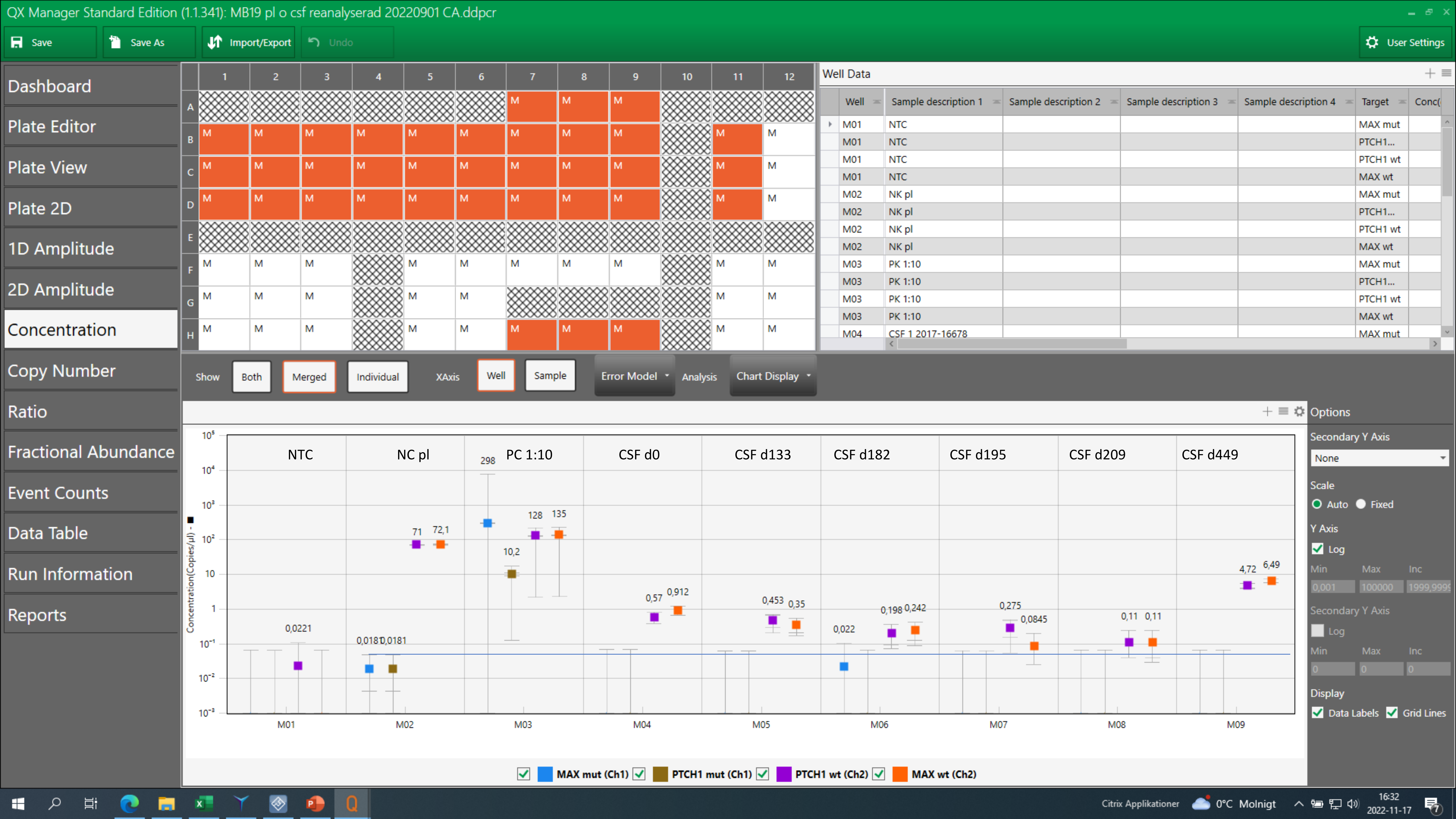
QX Manager Standard Edition (1.1.341): MB19 pl o csf reanalyserad 20220901 CA.ddpcr
Save
Save As
Import/Export
Undo
User Settings
Dashboard
Plate Editor
Plate View
Plate 2D
1D Amplitude
2D Amplitude
Concentration
Copy Number
Ratio
Fractional Abundance
Event Counts
Data Table
Run Information
Reports
Well Data
Well | Sample description 1 | Sample description 2 | Sample description 3 | Sample description 4 | Target | Conc(
M01 NTC MAX mut
M01 NTC PTCH1...
M01 NTC PTCH1 wt
M01 NTC MAX wt
M02 NK pl MAX mut
M02 NK pl PTCH1...
M02 NK pl PTCH1 wt
M02 NK pl MAX wt
M03 PK 1:10 MAX mut
M03 PK 1:10 PTCH1...
M03 PK 1:10 PTCH1 wt
M03 PK 1:10 MAX wt
M04 CSF 1 2017-16678 MAX mut
Show
Both
Merged
Individual
XAxis
Well
Sample
Error Model
Analysis
Chart Display
Options
NTC
NC pl
PC 1:10
CSF d0
CSF d133
CSF d182
CSF d195
CSF d209
CSF d449
Concentration(Copies/µl)
M01
M02
M03
M04
M05
M06
M07
M08
M09
MAX mut (Ch1)
PTCH1 mut (Ch1)
PTCH1 wt (Ch2)
MAX wt (Ch2)
Secondary Y Axis
None
Scale
Auto
Fixed
Y Axis
Log
Min
Max
Inc
0,001
100000
1999,999
Display
Data Labels
Grid Lines
Citrix Applikationer
0°C Molnigt
16:32
2022-11-17

MB19 CSF1 d0

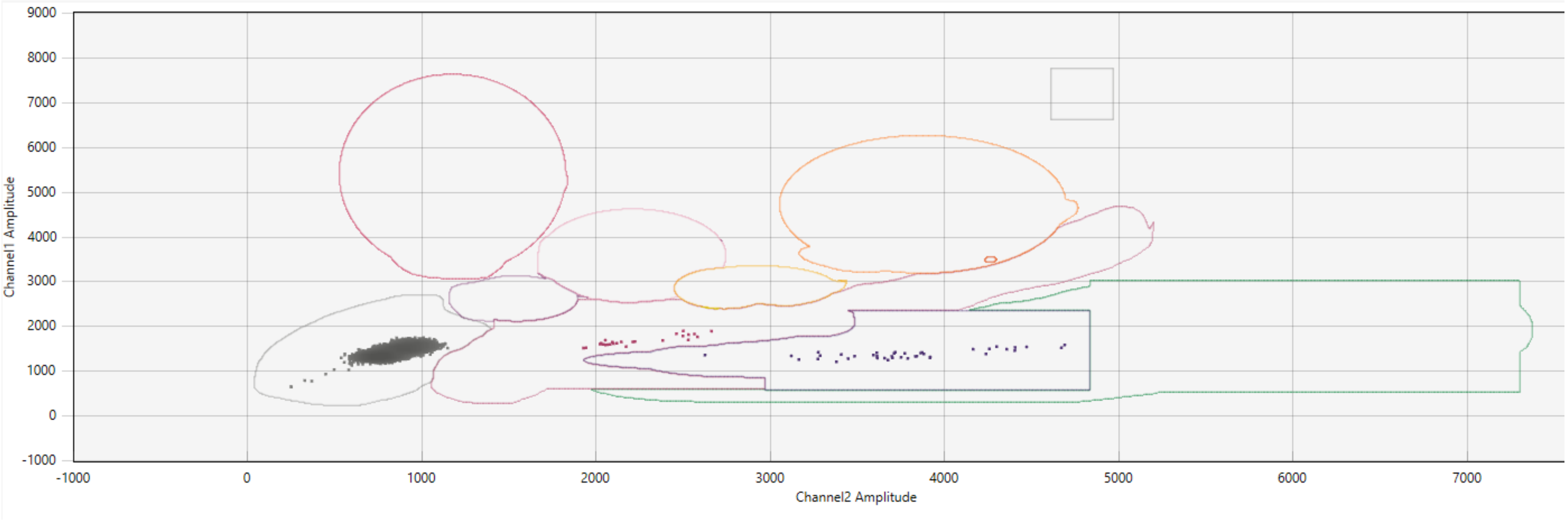

9000
8000
7000
6000
5000
4000
3000
2000
1000
0
-1000
Channel1 Amplitude
-1000
0
1000
2000
3000
4000
5000
6000
7000
Channel2 Amplitude

MB19 CSF d133

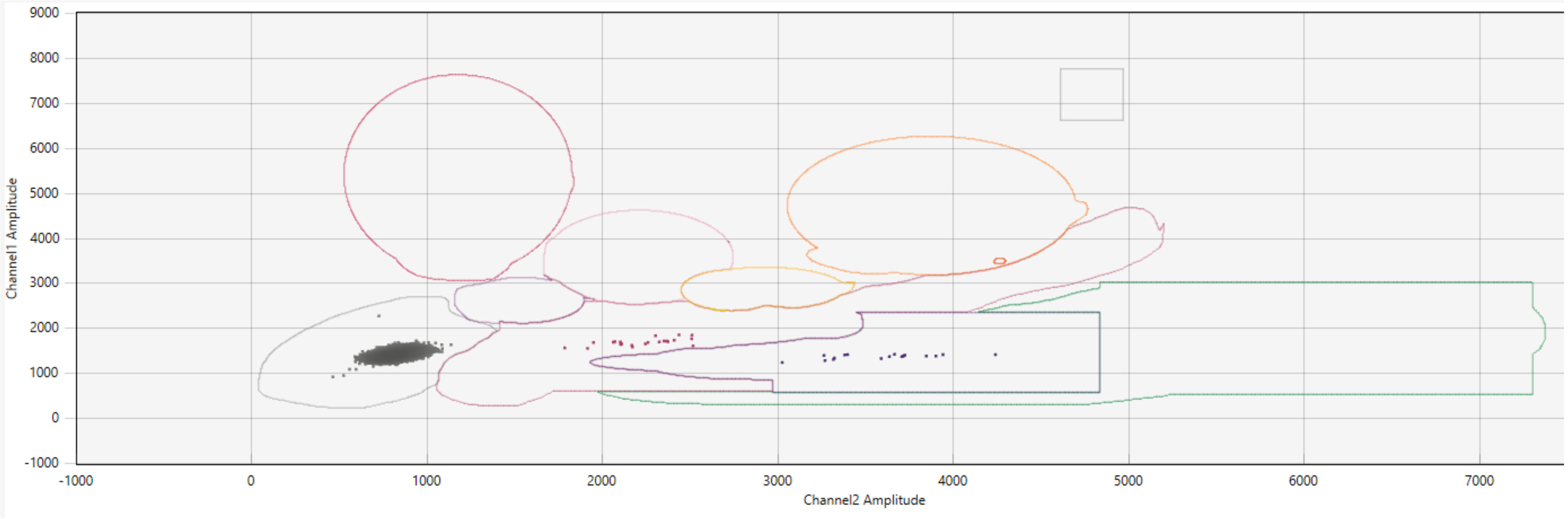

MB19 CSF d182

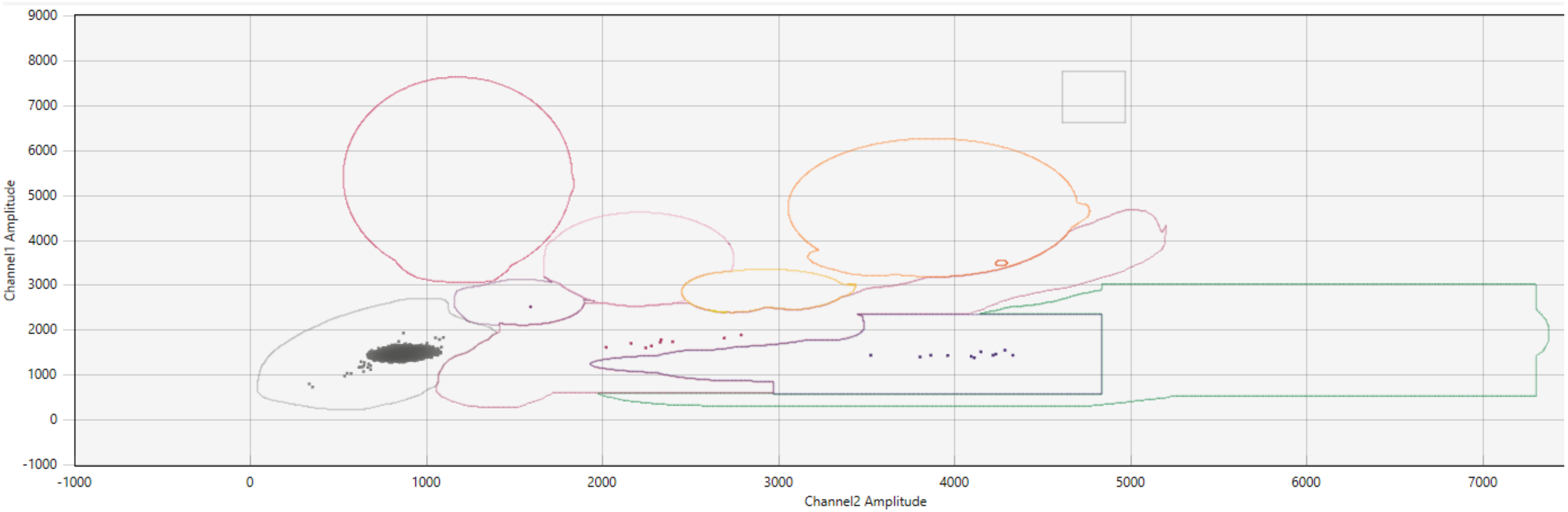

MB19 CSF d195

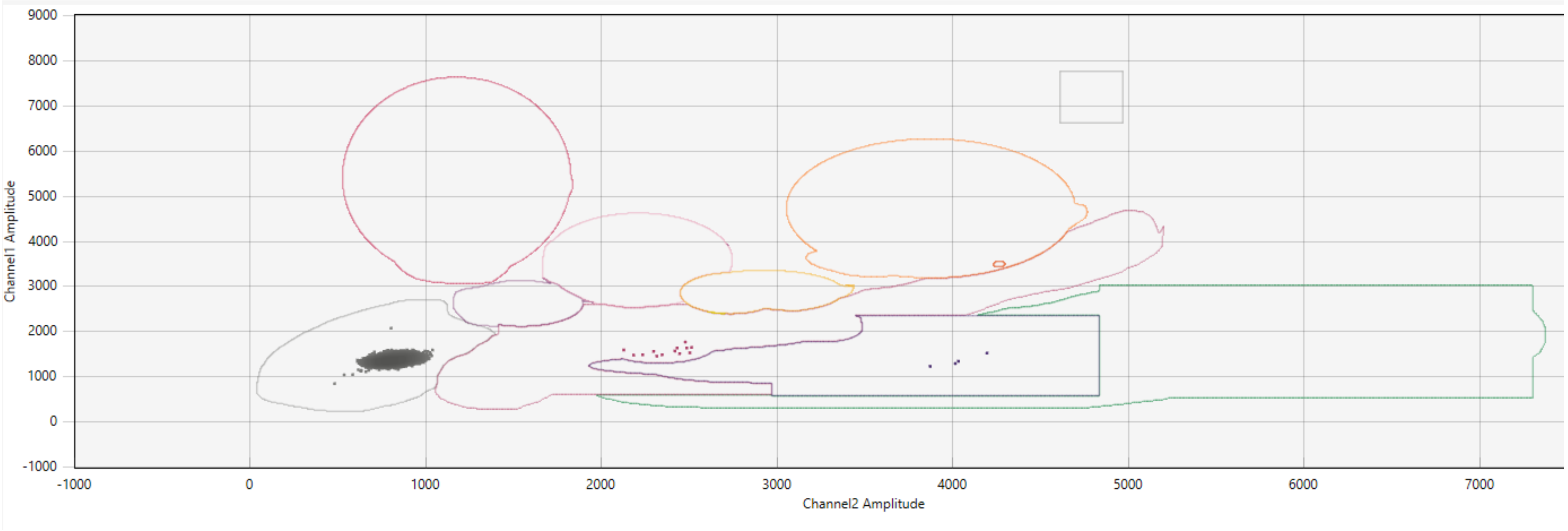

MB19 CSF d209

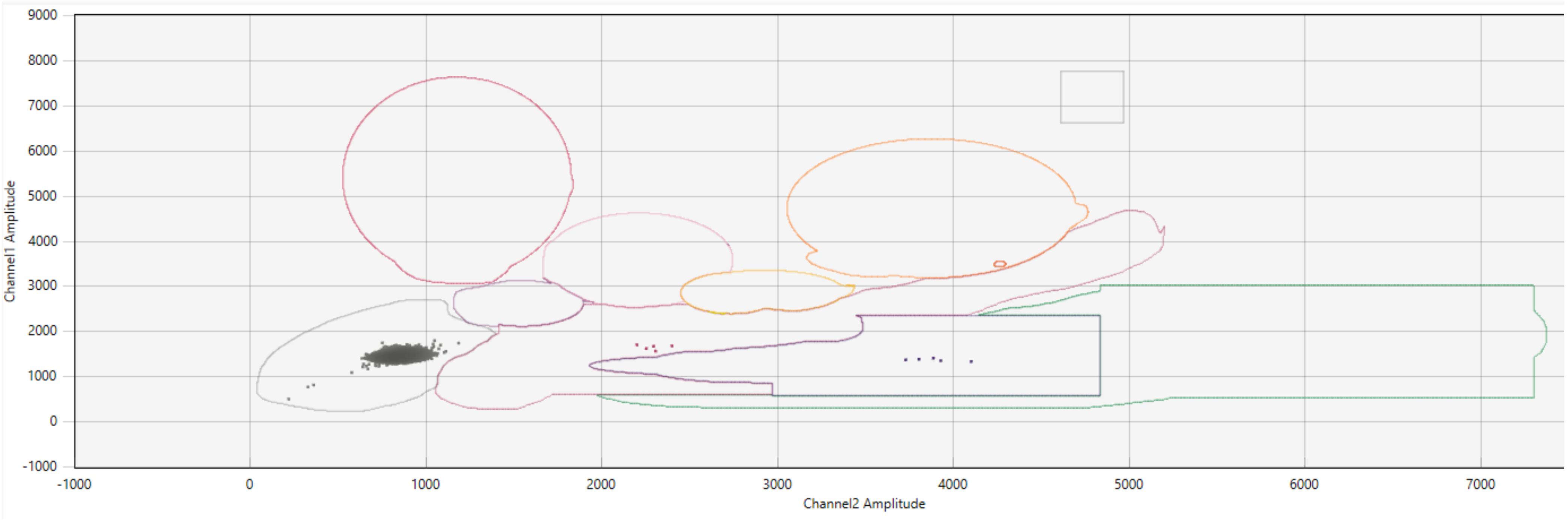

MB19 CSF d449

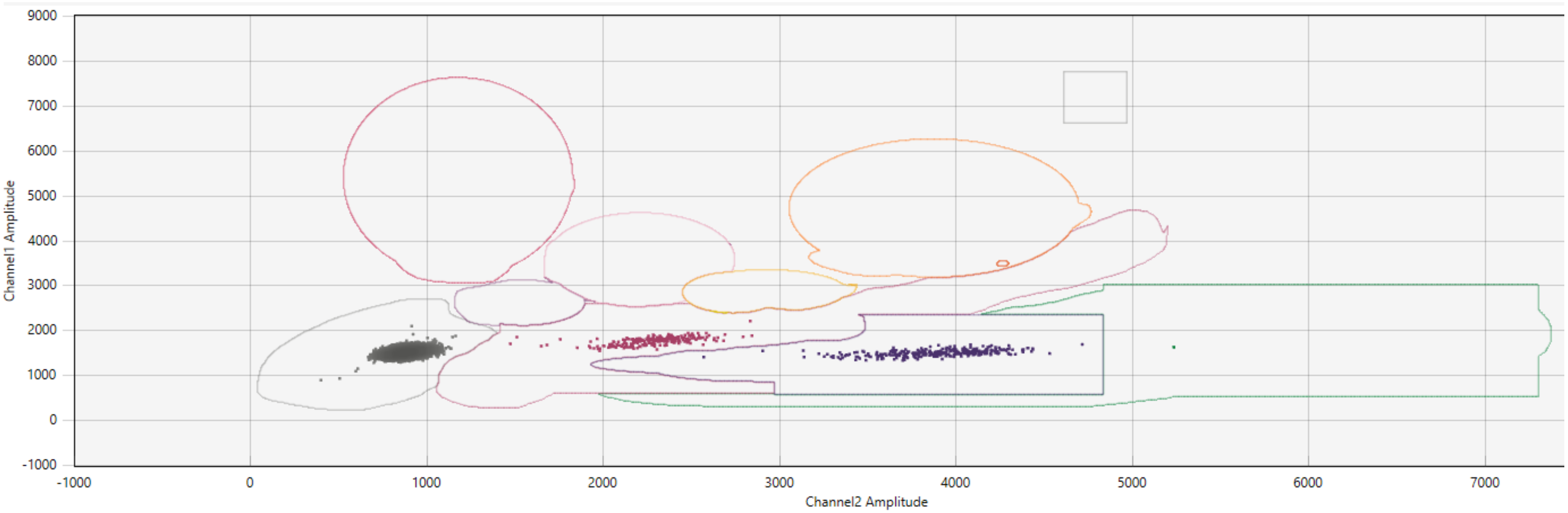

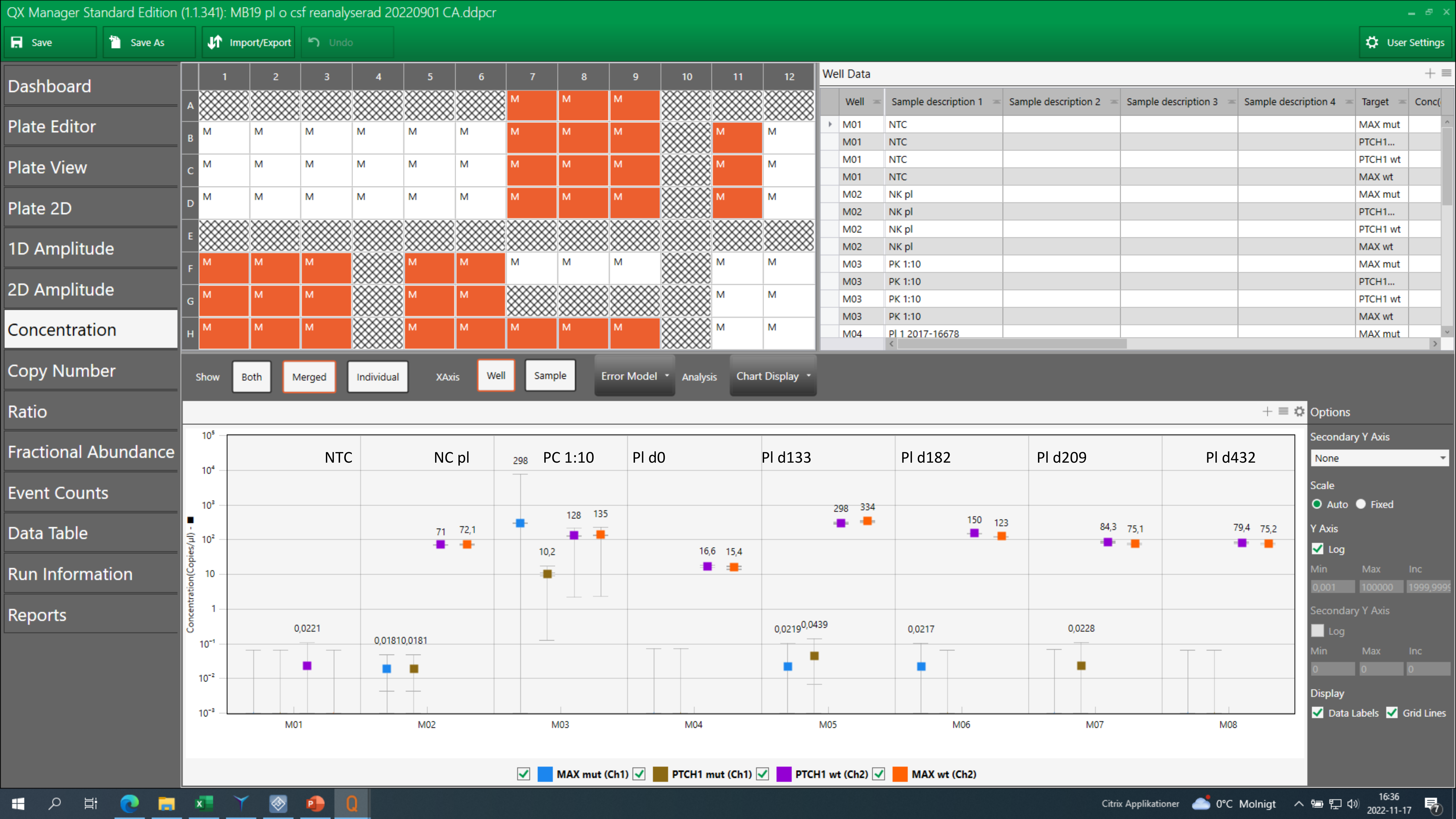
QX Manager Standard Edition (1.1.341): MB19 pl o csf reanalyserad 20220901 CA.ddpcr
Save
Save As
Import/Export
Undo
User Settings
Dashboard
Plate Editor
Plate View
Plate 2D
1D Amplitude
2D Amplitude
Concentration
Copy Number
Ratio
Fractional Abundance
Event Counts
Data Table
Run Information
Reports
Well Data
Well
Sample description 1
Sample description 2
Sample description 3
Sample description 4
Target
Conc(
M01 NTC MAX mut
M01 NTC PTCH1...
M01 NTC PTCH1 wt
M01 NTC MAX wt
M02 NK pl MAX mut
M02 NK pl PTCH1...
M02 NK pl PTCH1 wt
M02 NK pl MAX wt
M03 PK 1:10 MAX mut
M03 PK 1:10 PTCH1...
M03 PK 1:10 PTCH1 wt
M03 PK 1:10 MAX wt
M04 Pl 1 2017-16678 MAX mut
Show
Both
Merged
Individual
XAxis
Well
Sample
Error Model
Analysis
Chart Display
Options
Secondary Y Axis
None
Scale
Auto
Fixed
Y Axis
Log
Min
Max
Inc
0,001
100000
1999,999
Secondary Y Axis
Log
Min
Max
Inc
0
0
0
Display
Data Labels
Grid Lines
NTC
NC pl
PC 1:10
Pl d0
Pl d133
Pl d182
Pl d209
Pl d432
Concentration(Copies/µl)
298
71
72,1
10,2
128
135
16,6
15,4
298
334
150
123
84,3
75,1
79,4
75,2
0,0221
0,0181
0,0181
0,0219
0,0439
0,0217
0,0228
M01
M02
M03
M04
M05
M06
M07
M08
MAX mut (Ch1)
PTCH1 mut (Ch1)
PTCH1 wt (Ch2)
MAX wt (Ch2)
Citrix Applikationer
0°C Molnigt
16:36
2022-11-17

MB19 Pl d0

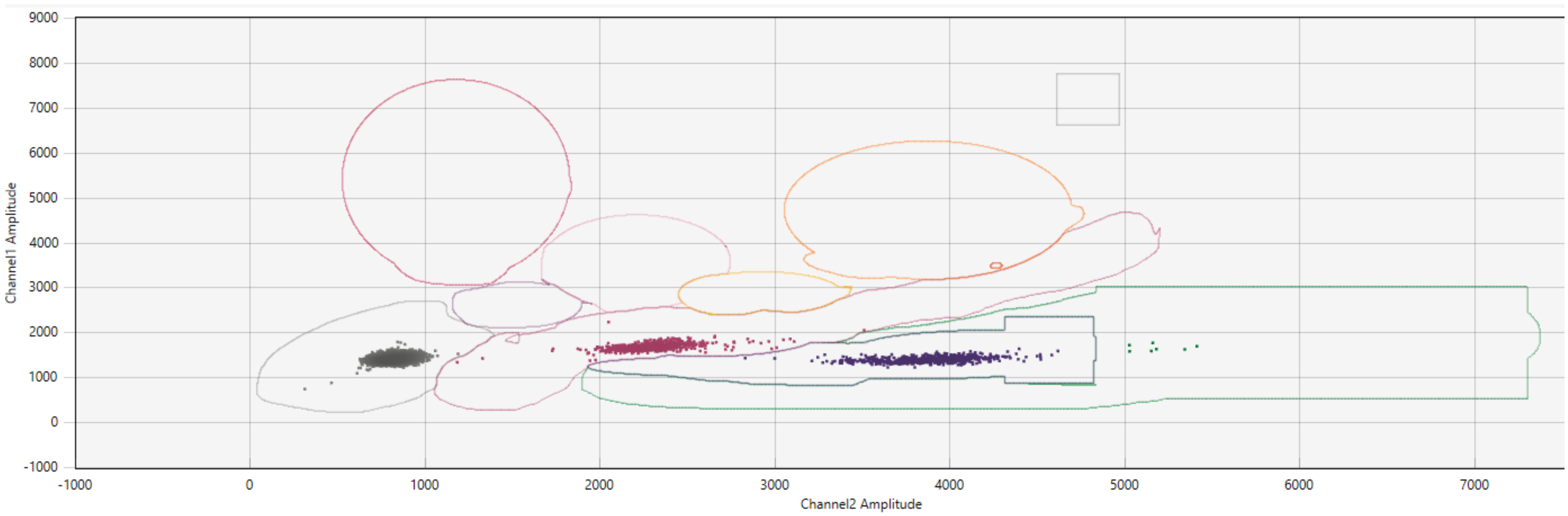
9000
8000
7000
6000
5000
4000
3000
2000
1000
0
-1000
Channel1 Amplitude
-1000
0
1000
2000
3000
4000
5000
6000
7000
Channel2 Amplitude

MB19 Pl d133

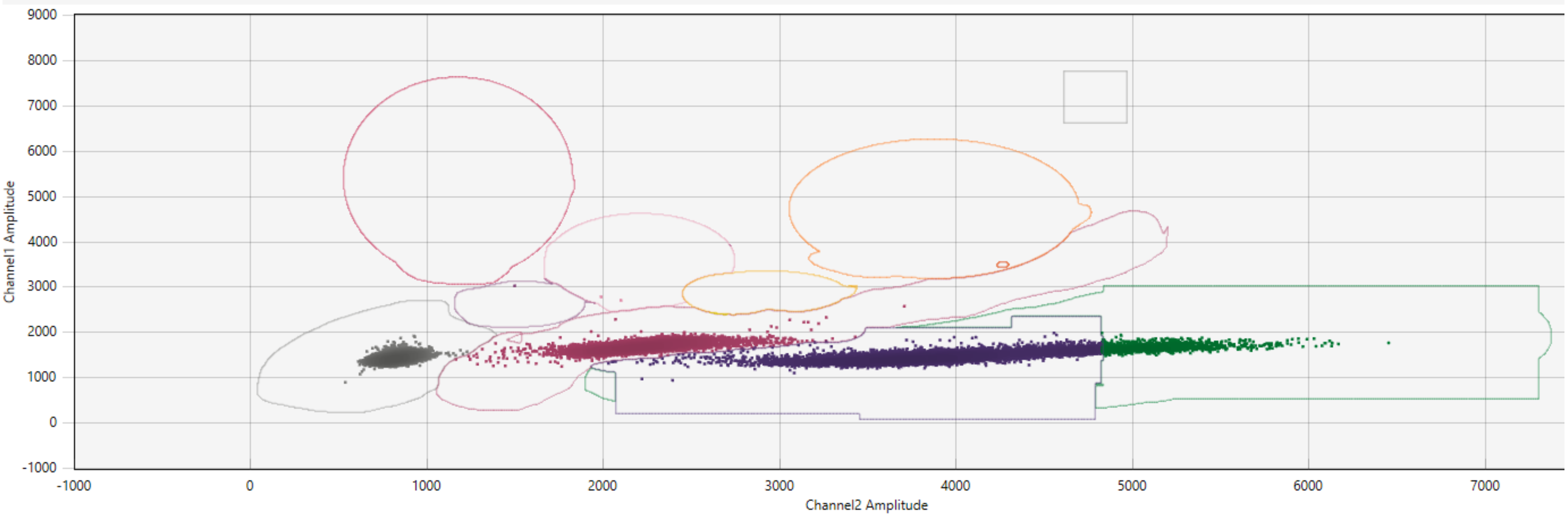

9000
8000
7000
6000
5000
4000
3000
2000
1000
0
-1000
Channel1 Amplitude
-1000
0
1000
2000
3000
4000
5000
6000
7000
Channel2 Amplitude

MB19 Pl d182

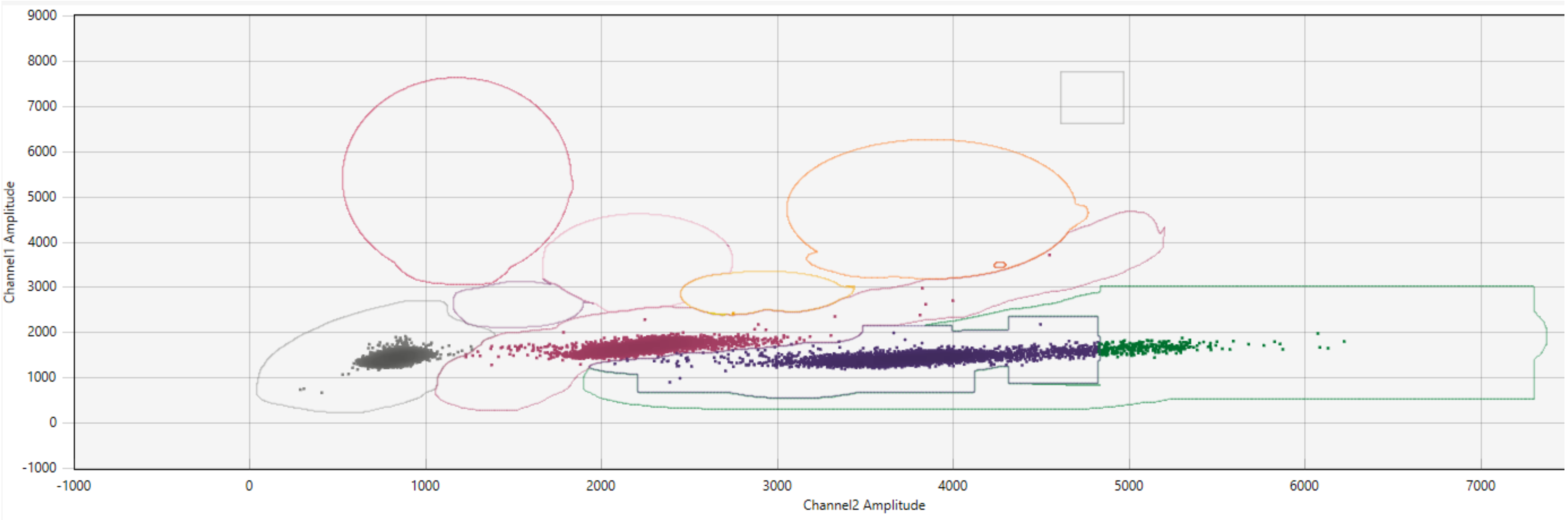

9000
8000
7000
6000
5000
4000
3000
2000
1000
0
-1000
Channel1 Amplitude
-1000
0
1000
2000
3000
4000
5000
6000
7000
Channel2 Amplitude

MB19 Pl d209

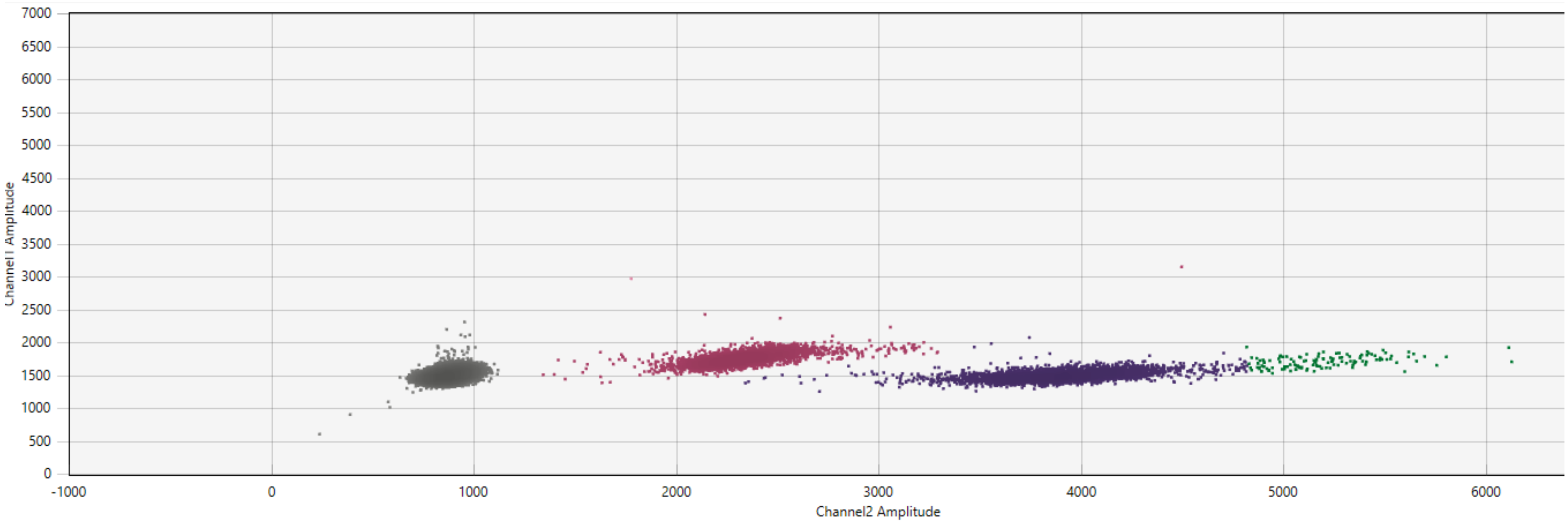

7000
6500
6000
5500
5000
4500
4000
3500
3000
2500
2000
1500
1000
500
0
Channel1 Amplitude
-1000
0
1000
2000
3000
4000
5000
6000
Channel2 Amplitude

MB19 Pl d432 (2018-24650) neg

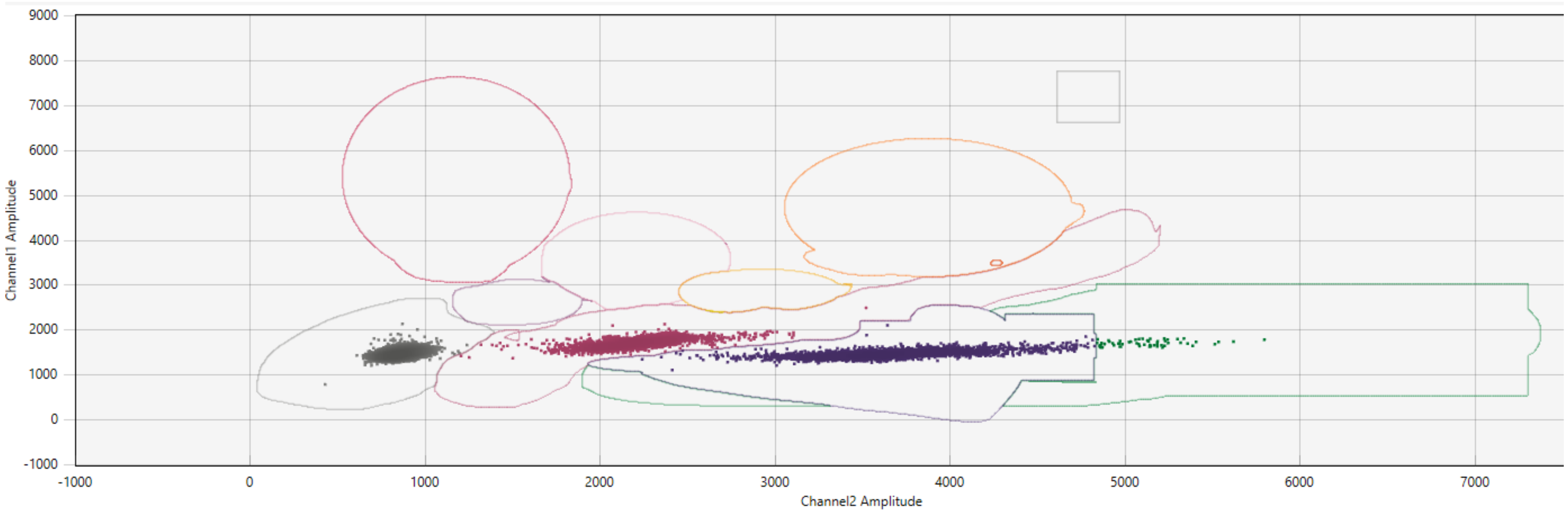

Supplement: Supplementary file 1 [file cancers-15-01972-s001.zip › File S2 QX Manager Software output data on liquid biopsies/Liquid biopsies MB19.pdf]
