# Supplementary material for: Simultaneous Ultra-Sensitive Detection of Structural and Single Nucleotide Variants Using Multiplex Droplet Digital PCR in Liquid Biopsies from Children with Medulloblastoma
Source: Cancers (Basel). 2023 Mar 25;15(7):1972. doi: 10.3390/cancers15071972 (PMC10092983; doi:10.3390/cancers15071972)
Supplement: Supplementary file 1 [file cancers-15-01972-s001.zip › File S2 QX Manager Software output data on liquid biopsies/Liquid biopsies MB21.pdf]

MB21 NTC

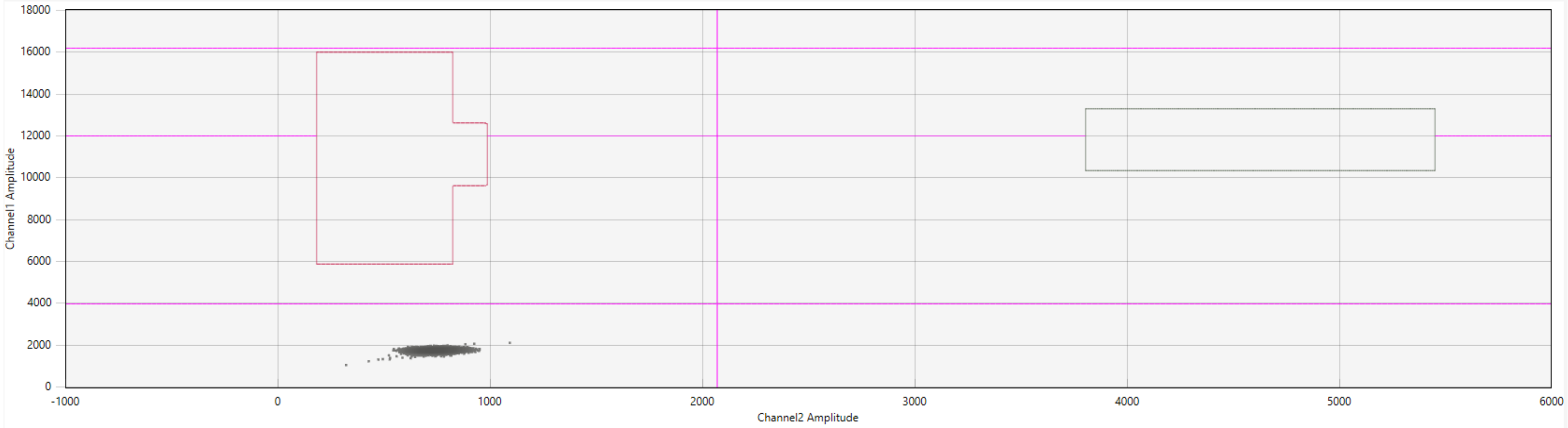

Channel1 Amplitude
18000
16000
14000
12000
10000
8000
6000
4000
2000
0
-1000
0
1000
2000
3000
4000
5000
6000
Channel2 Amplitude

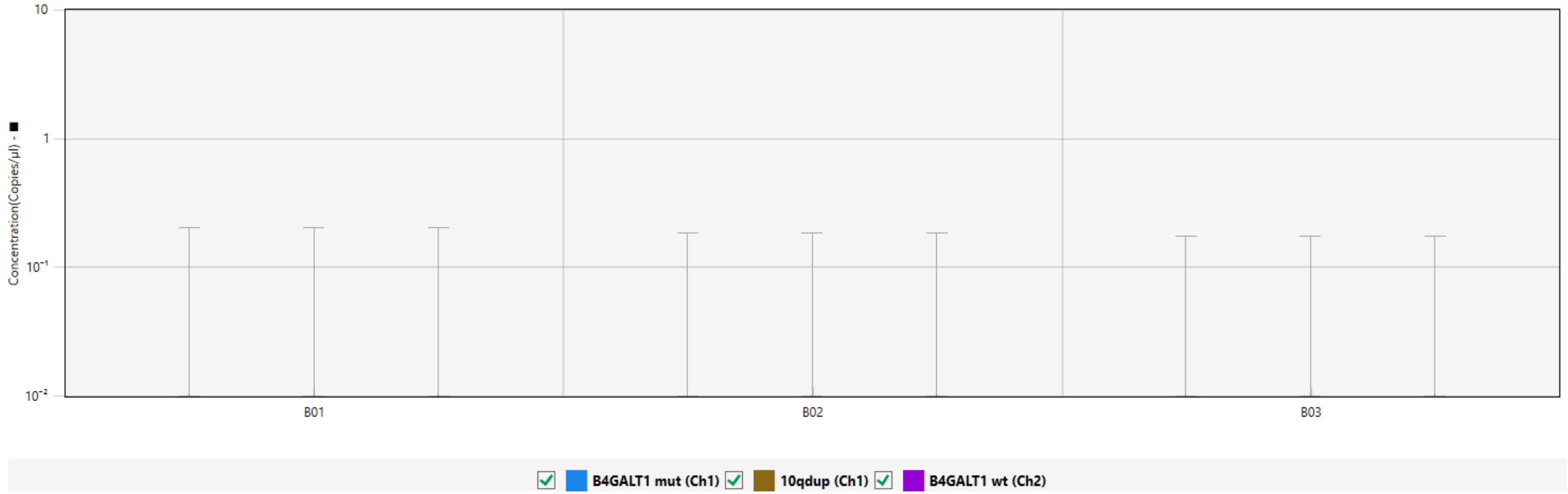

Concentration(Copies/µl) -
10
1
10^{-1}
10^{-2}
B01
B02
B03
B4GALT1 mut (Ch1)
10qdup (Ch1)
B4GALT1 wt (Ch2)

MB 21 NC pl & gNC

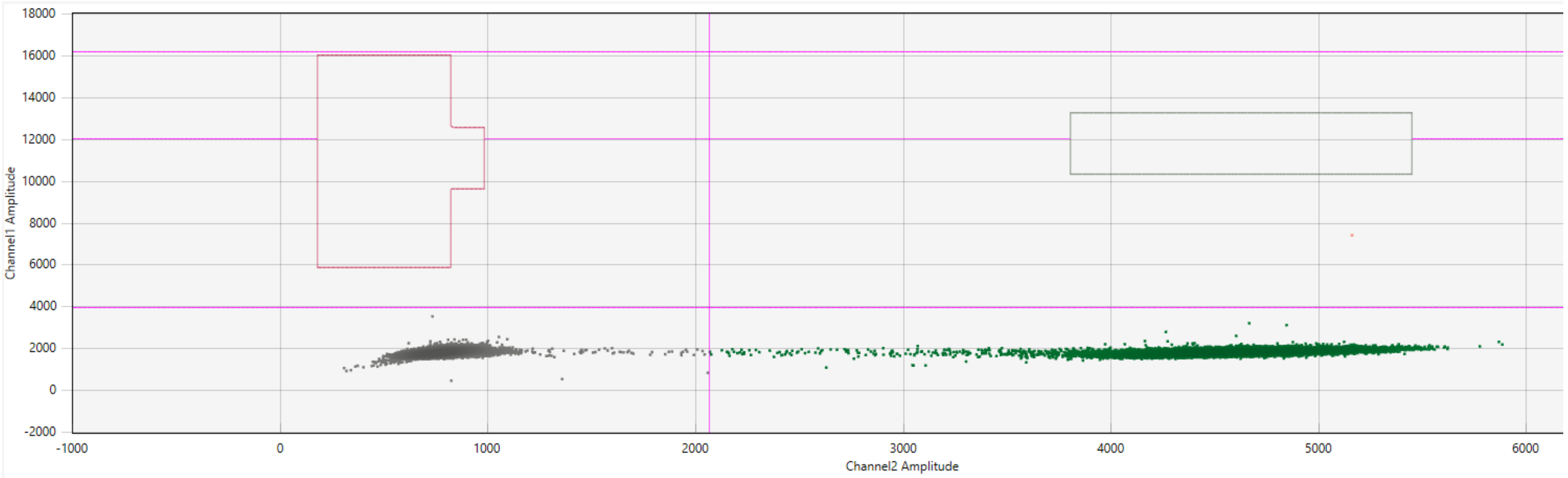

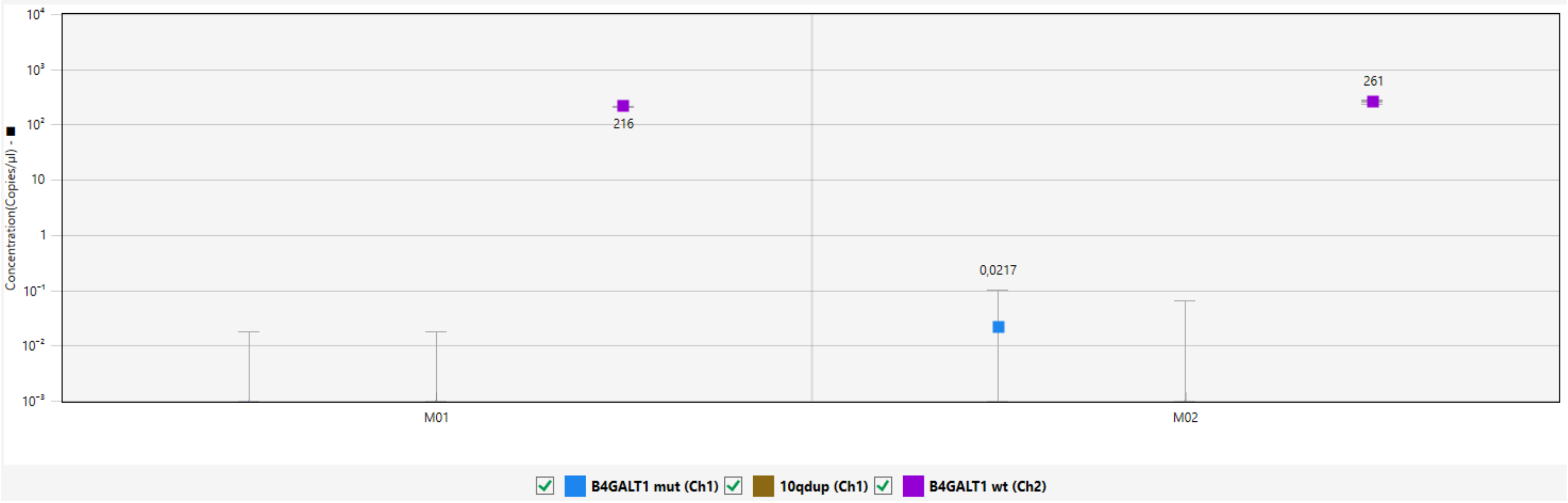

MB21 Example of clusters (PC 1:10)

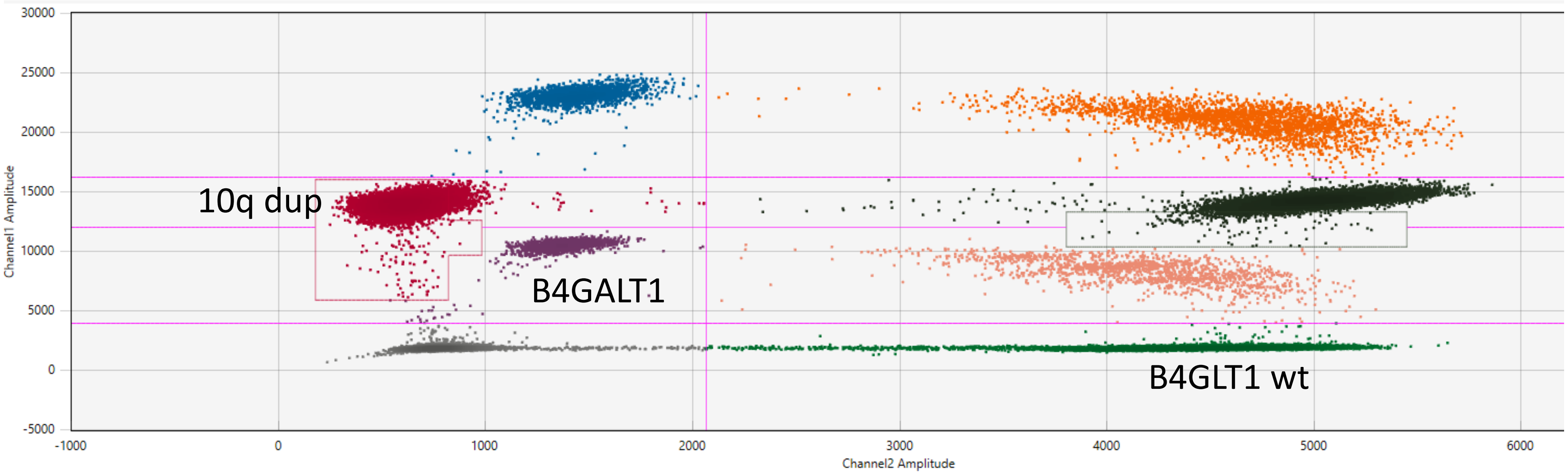

MB21 Dilution series:
LoD & LoQ B4GALT 1:1000. 10qdup 1:10000.

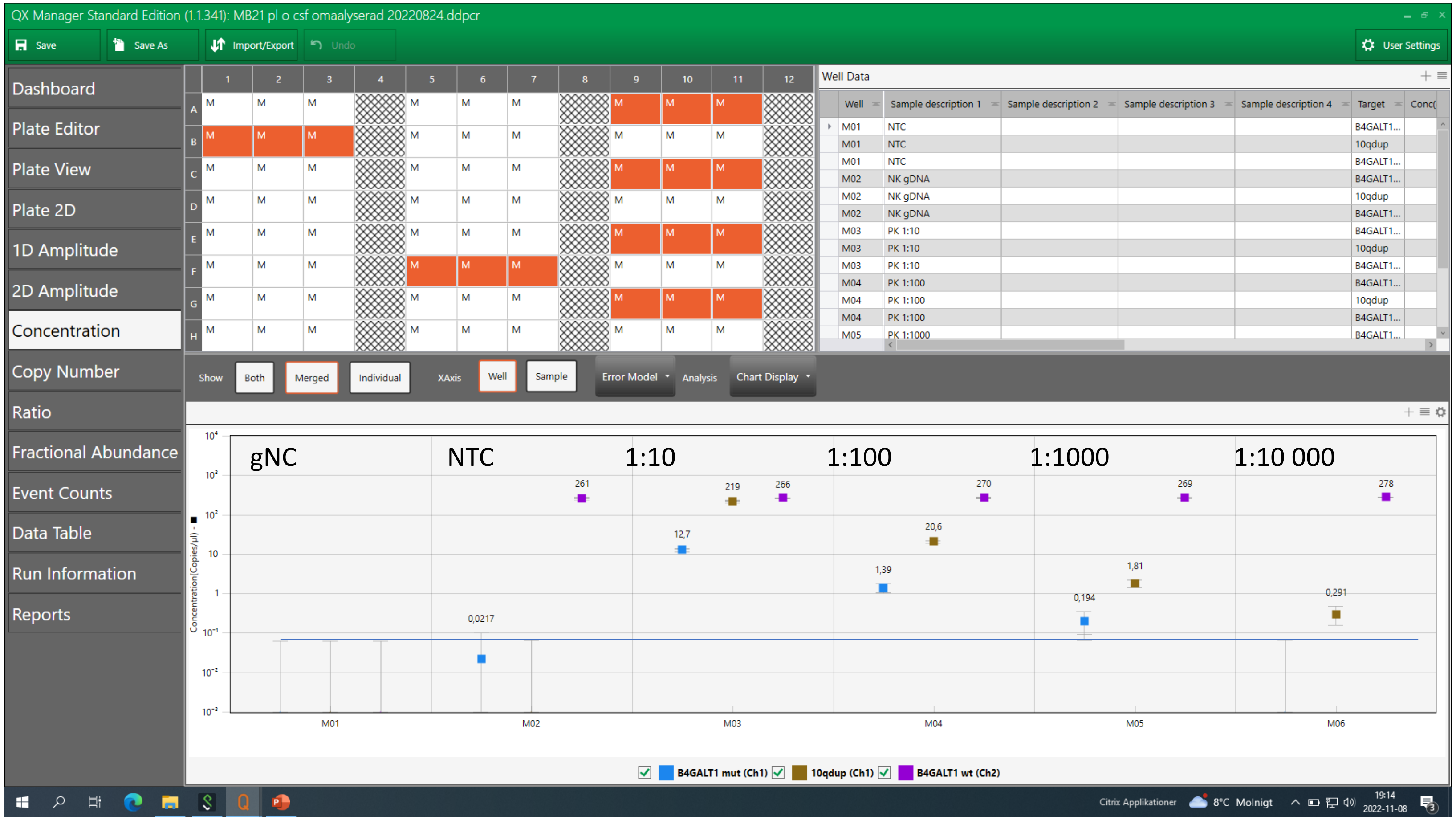

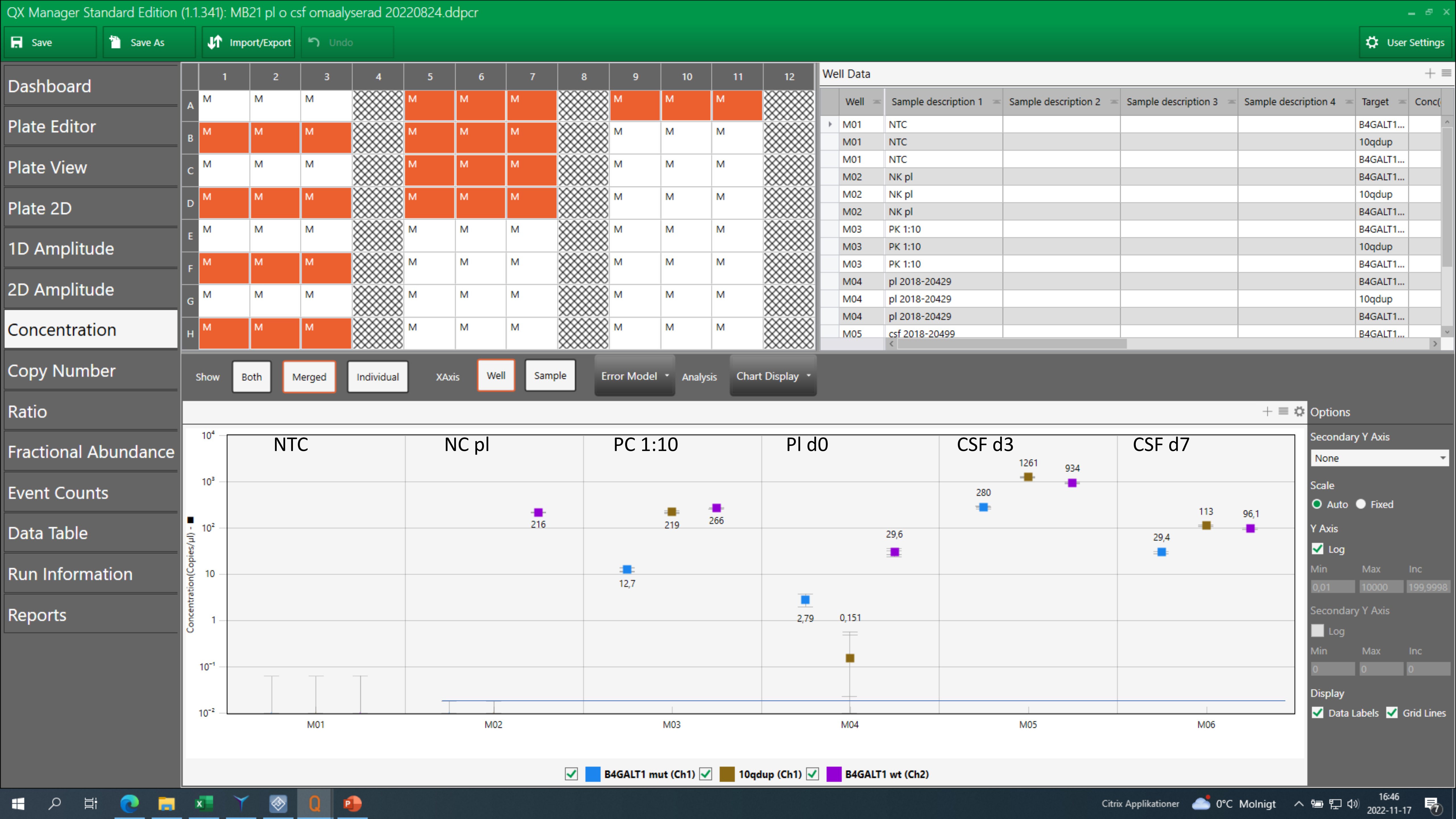
QX Manager Standard Edition (1.1.341): MB21 pl o csf omaalyserad 20220824.ddpcr
Save
Save As
Import/Export
Undo
User Settings
Dashboard
Plate Editor
Plate View
Plate 2D
1D Amplitude
2D Amplitude
Concentration
Copy Number
Ratio
Fractional Abundance
Event Counts
Data Table
Run Information
Reports
Well Data
Well
Sample description 1
Sample description 2
Sample description 3
Sample description 4
Target
Conc(
M01 NTC B4GALT1...
M01 NTC 10qdup
M01 NTC B4GALT1...
M02 NK pl B4GALT1...
M02 NK pl 10qdup
M02 NK pl B4GALT1...
M03 PK 1:10 B4GALT1...
M03 PK 1:10 10qdup
M03 PK 1:10 B4GALT1...
M04 pl 2018-20429 B4GALT1...
M04 pl 2018-20429 10qdup
M04 pl 2018-20429 B4GALT1...
M05 csf 2018-20499 B4GALT1...
Show
Both
Merged
Individual
XAxis
Well
Sample
Error Model
Analysis
Chart Display
Options
Secondary Y Axis
None
Scale
Auto
Fixed
Y Axis
Log
Min
Max
Inc
0,01
10000
199,9998
Secondary Y Axis
Log
Display
Data Labels
Grid Lines
NTC
NC pl
PC 1:10
Pl d0
CSF d3
CSF d7
Concentration(Copies/µl)
216
219
266
12,7
2,79
0,151
29,6
280
1261
934
29,4
113
96,1
M01
M02
M03
M04
M05
M06
B4GALT1 mut (Ch1)
10qdup (Ch1)
B4GALT1 wt (Ch2)
Citrix Applikationer
0°C Molnigt
16:46
2022-11-17

MB1 Pl d0

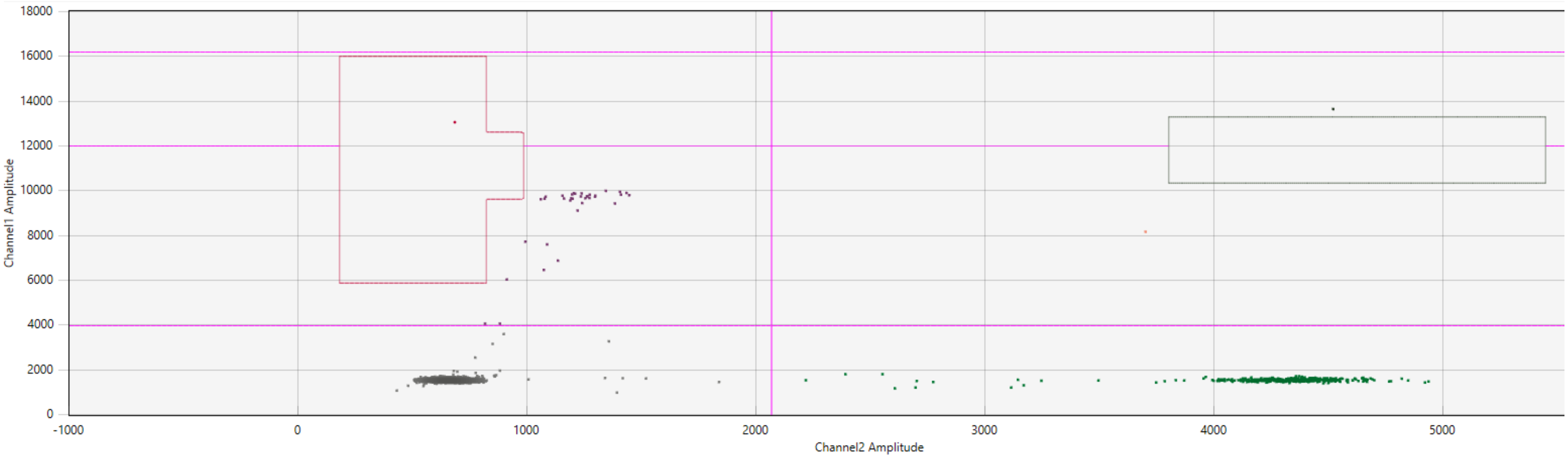

18000
16000
14000
12000
10000
8000
6000
4000
2000
0
Channel1 Amplitude
-1000
0
1000
2000
3000
4000
5000
Channel2 Amplitude

MB21 CSF d3

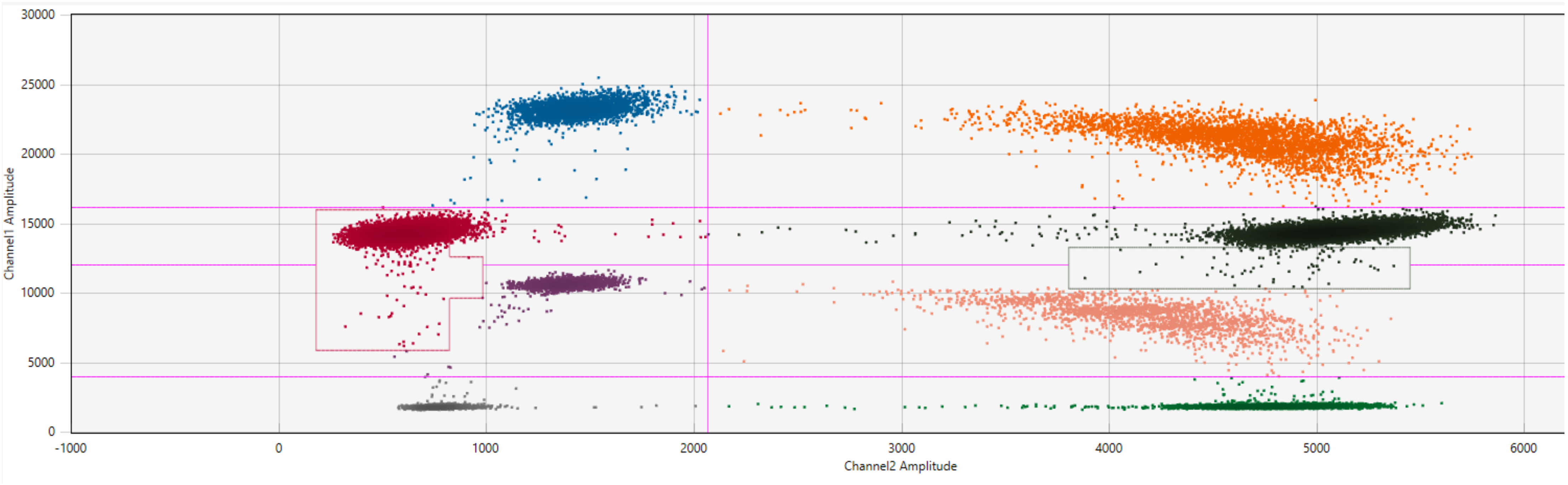

30000
25000
20000
15000
10000
5000
0
Channel1 Amplitude
-1000
0
1000
2000
3000
4000
5000
6000
Channel2 Amplitude

MB21 CSF d7

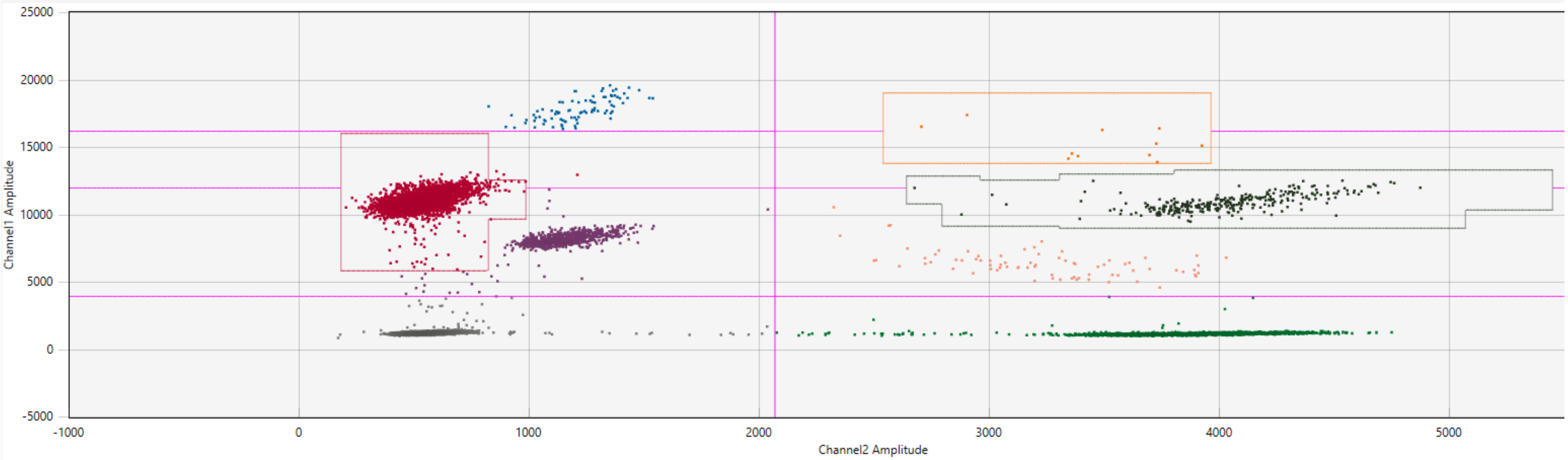
25000
20000
15000
10000
5000
0
-5000
Channel1 Amplitude
-1000
0
1000
2000
3000
4000
5000
Channel2 Amplitude
