# Supplementary material for: Simultaneous Ultra-Sensitive Detection of Structural and Single Nucleotide Variants Using Multiplex Droplet Digital PCR in Liquid Biopsies from Children with Medulloblastoma
Source: Cancers (Basel). 2023 Mar 25;15(7):1972. doi: 10.3390/cancers15071972 (PMC10092983; doi:10.3390/cancers15071972)
Supplement: Supplementary file 1 [file cancers-15-01972-s001.zip › File S2 QX Manager Software output data on liquid biopsies/Liquid biopsies MB22.pdf]

MB22 NTC

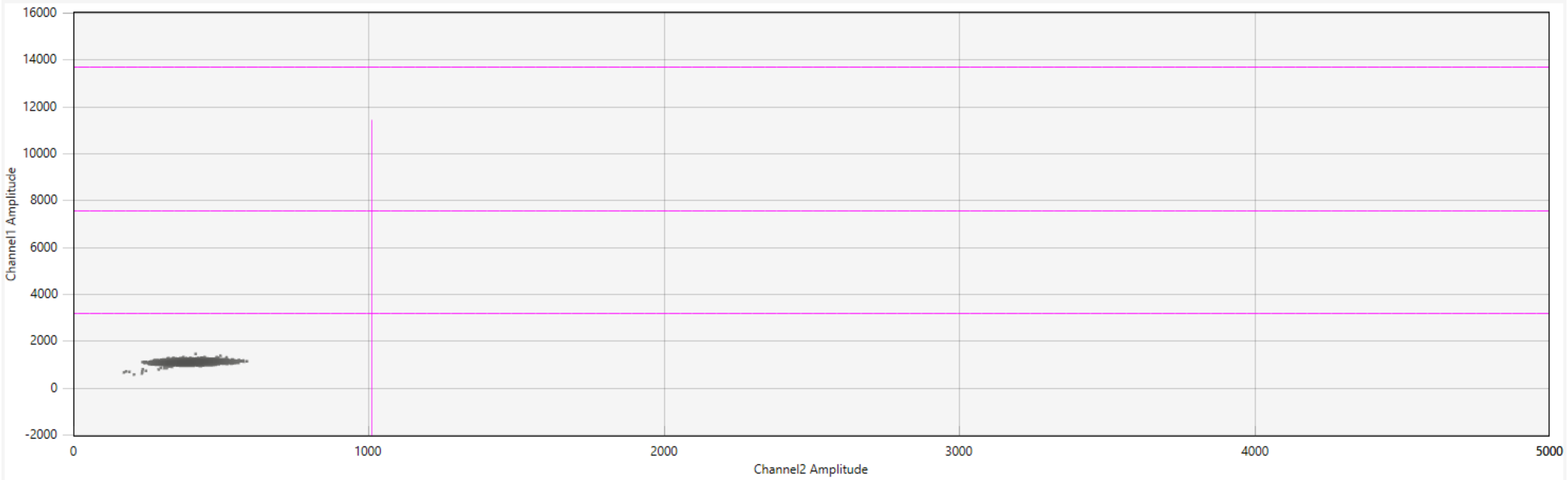

16000
14000
12000
10000
8000
6000
4000
2000
0
-2000
Channel1 Amplitude
0
1000
2000
3000
4000
5000
Channel2 Amplitude

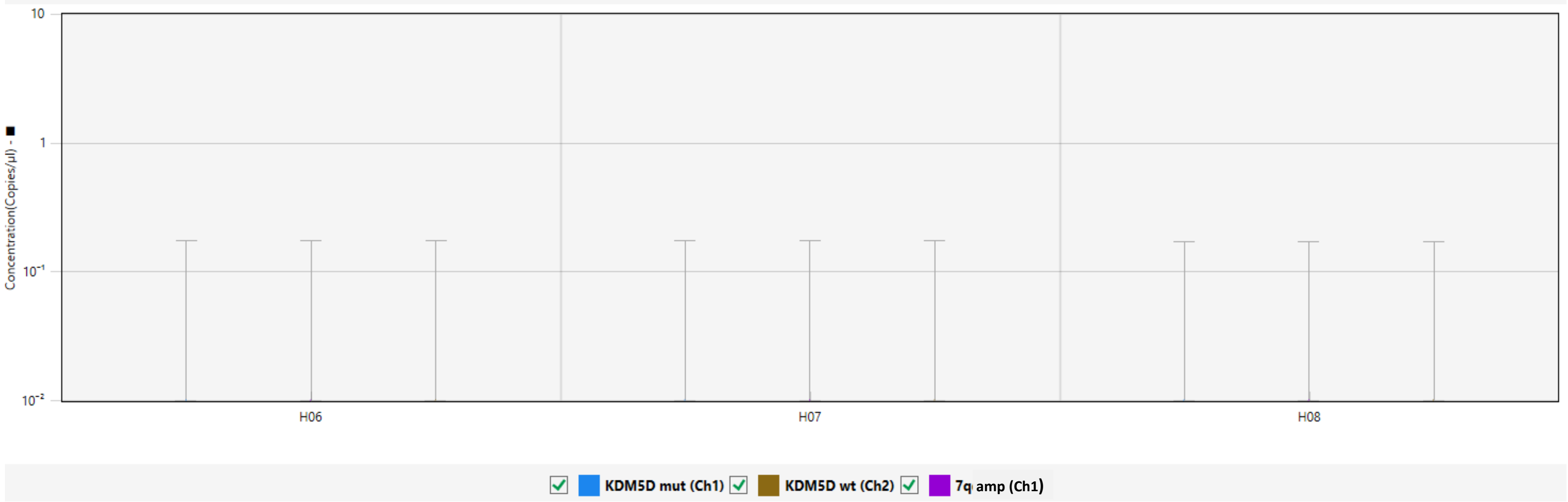

10
1
10⁻¹
10⁻²
Concentration(Copies/µl)
H06
H07
H08
KDM5D mut (Ch1)
KDM5D wt (Ch2)
7q amp (Ch1)

MB22 NC pl & gNC

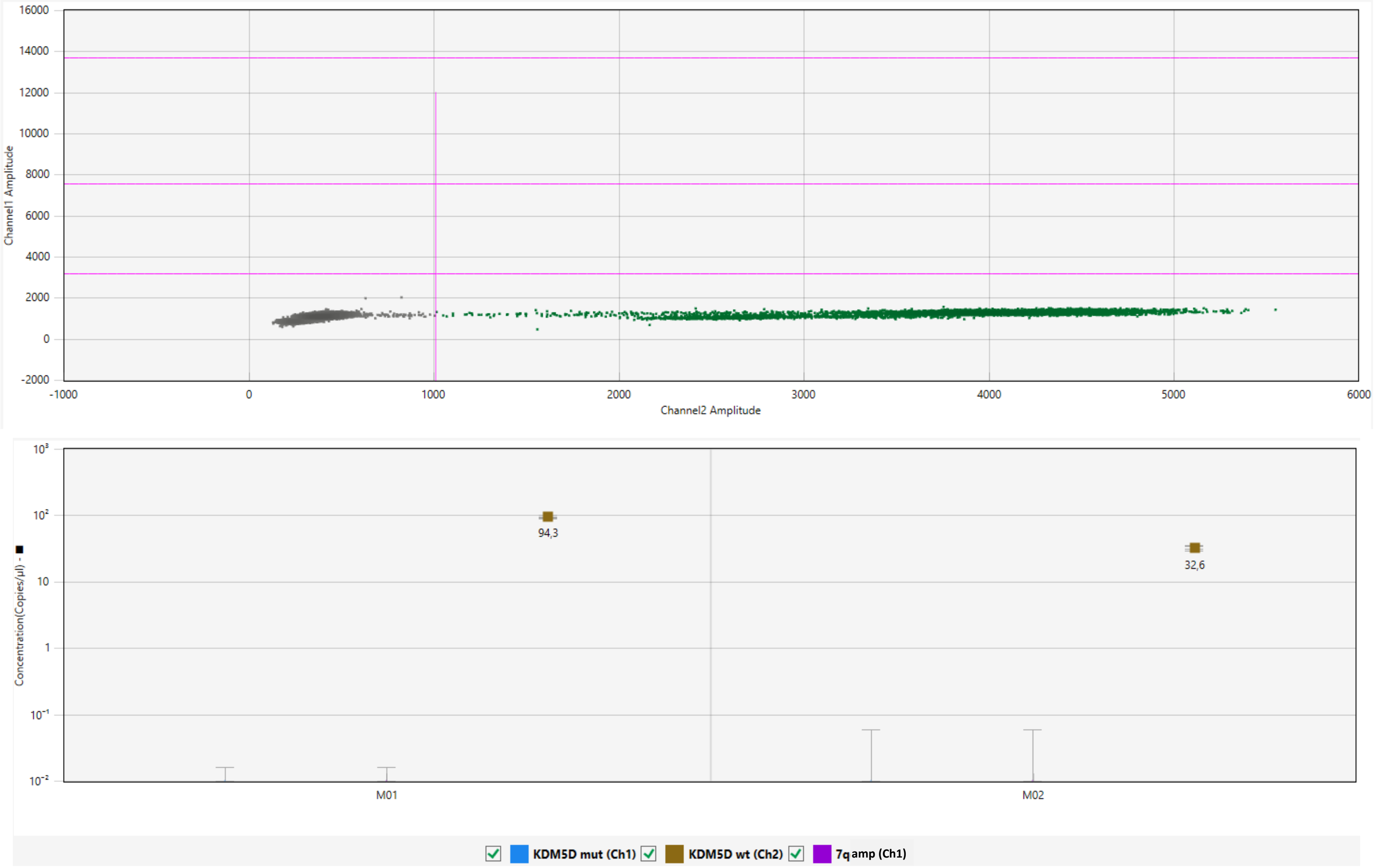

MB22 example clusters (PC 1:10)

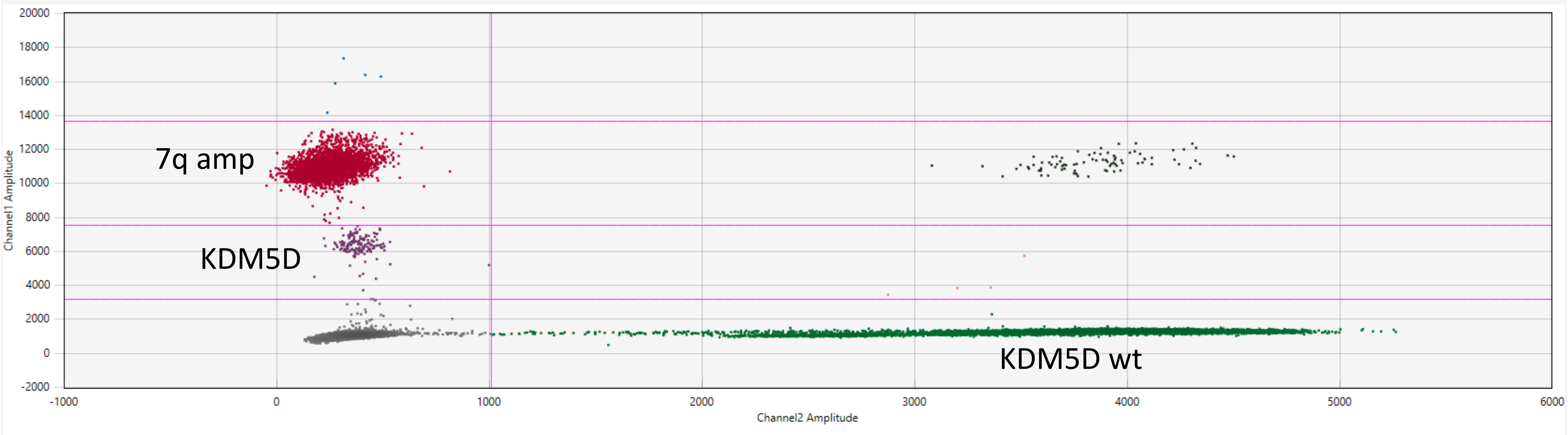

MB22 Dilution series: LoD & LoQ KDM5D 1:100. LoD & LoQ 7q amp 1:1000.

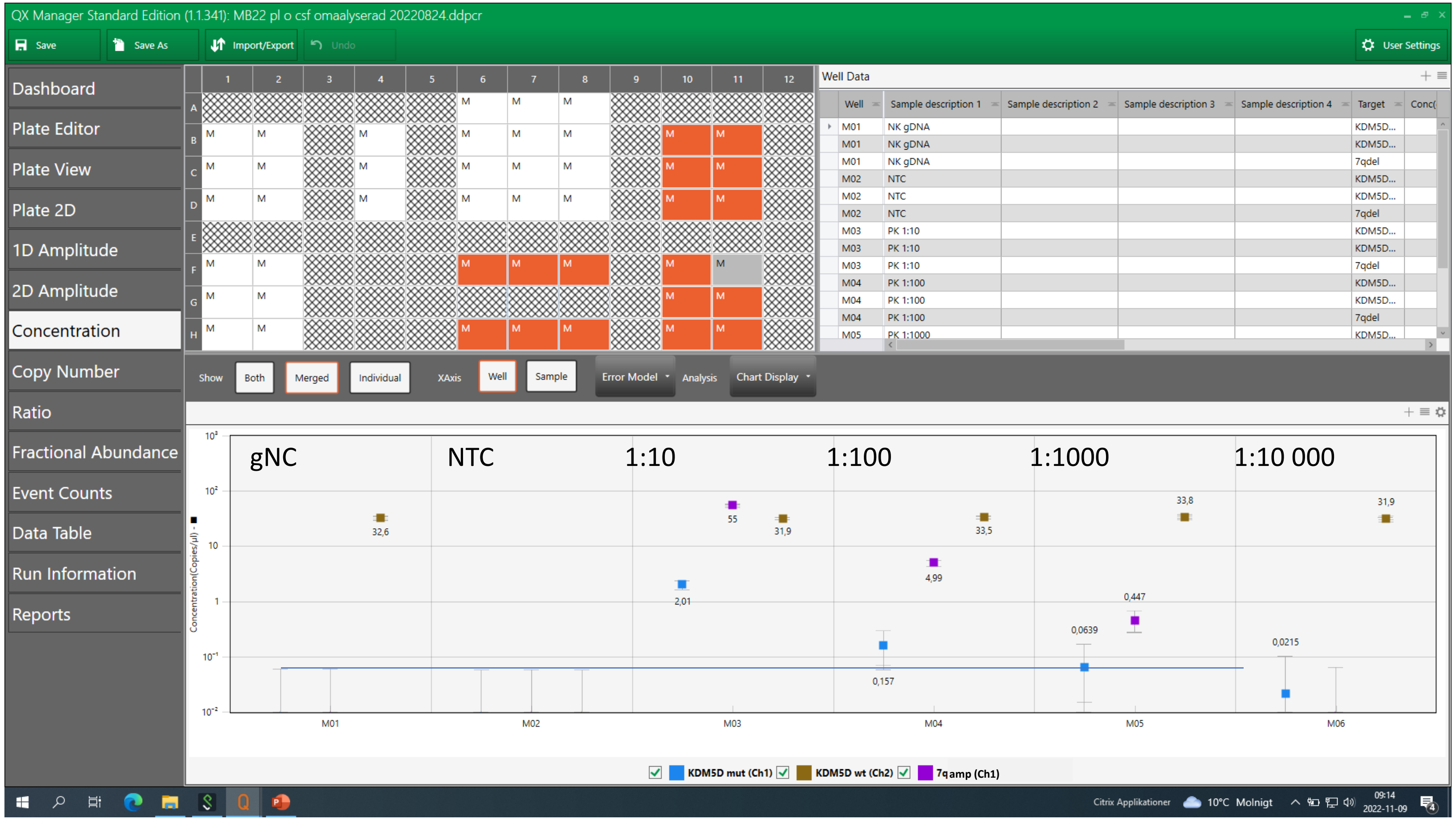

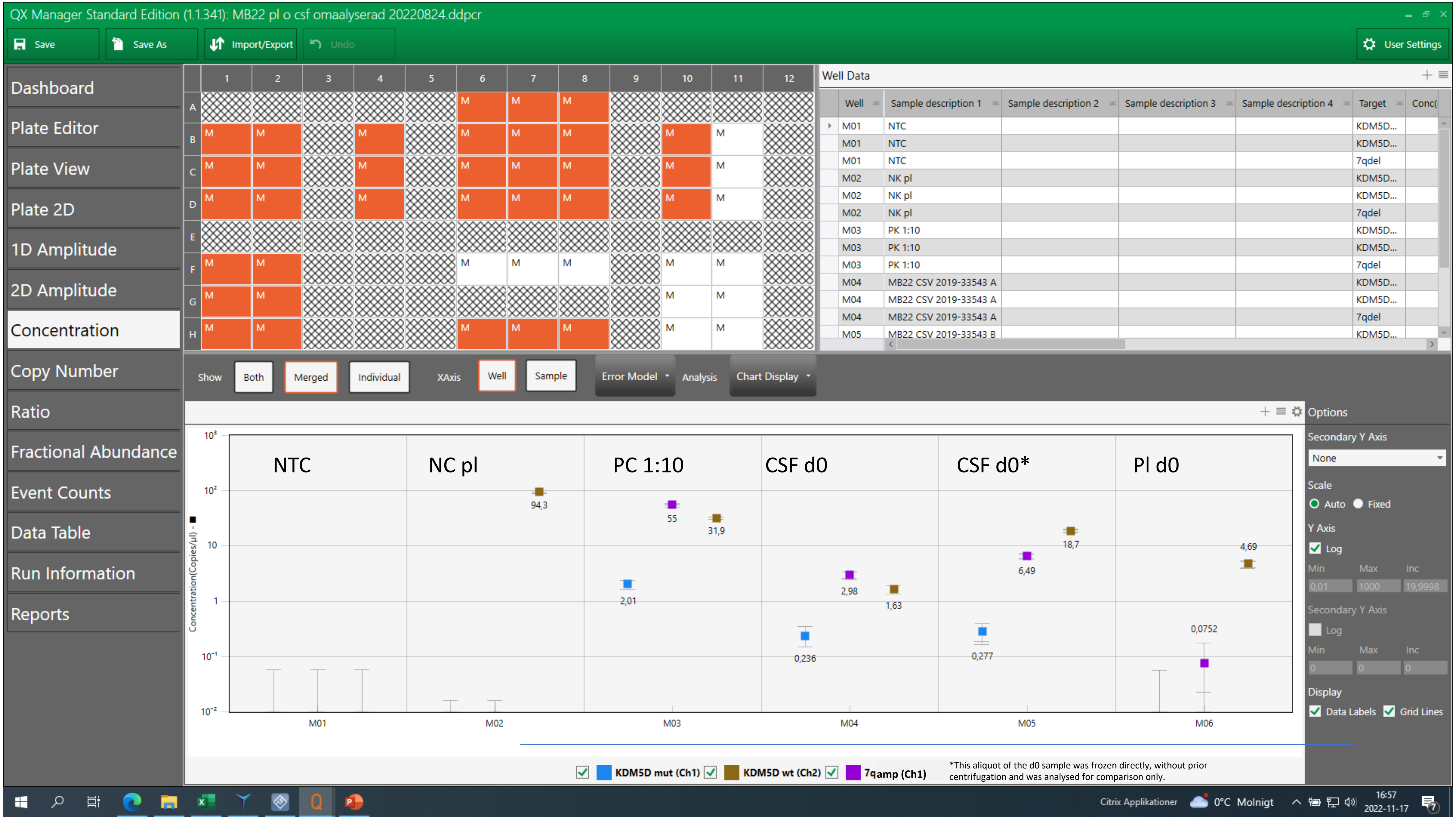

*This aliquot of the d0 sample was frozen directly, without prior centrifugation and was analysed for comparison only.

MB22 CSF d0

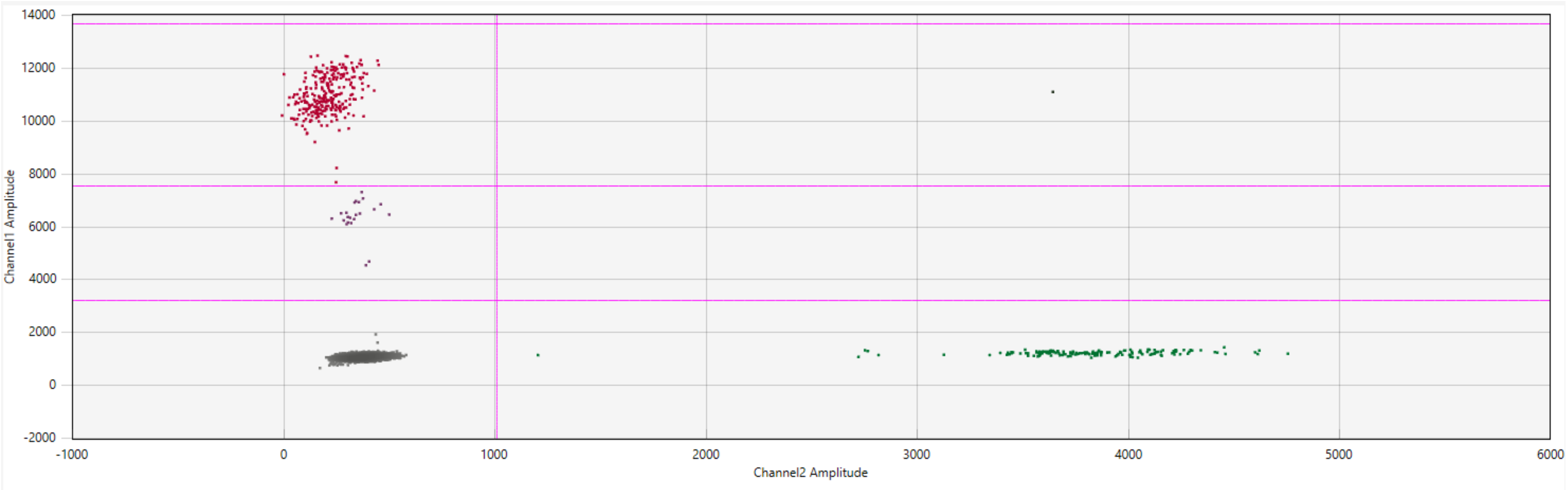

14000
12000
10000
8000
6000
4000
2000
0
-2000
Channel1 Amplitude
-1000
0
1000
2000
3000
4000
5000
6000
Channel2 Amplitude

MB22 Pl d0

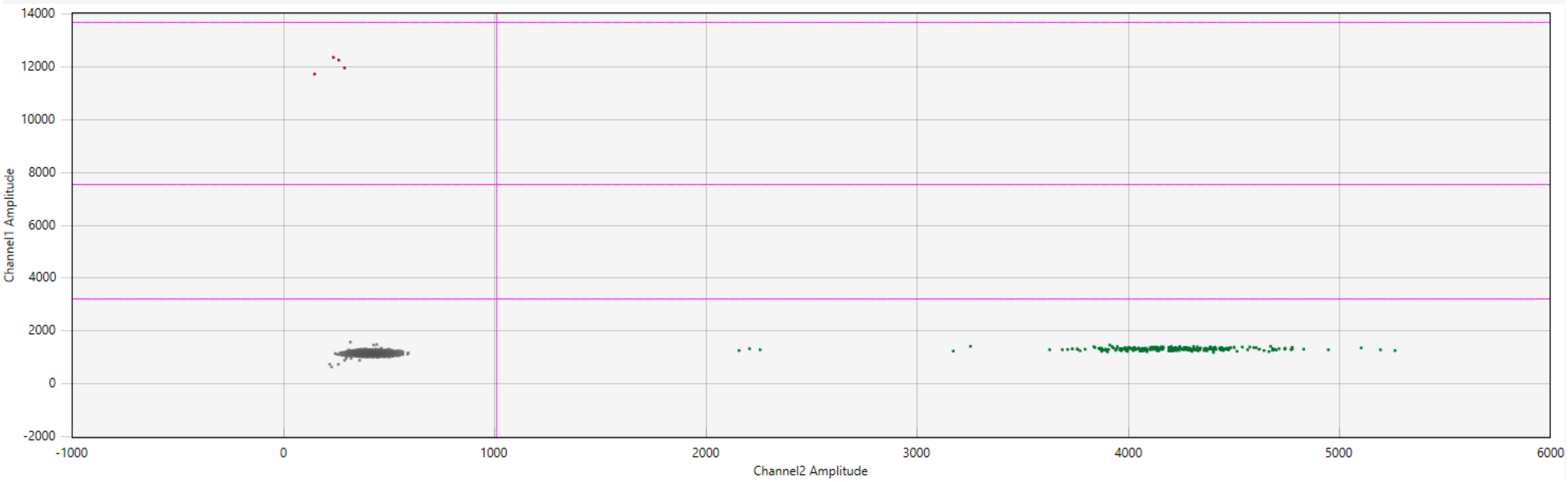

14000
12000
10000
8000
6000
4000
2000
0
-2000
Channel1 Amplitude
-1000
0
1000
2000
3000
4000
5000
6000
Channel2 Amplitude
