# Supplementary material for: Simultaneous Ultra-Sensitive Detection of Structural and Single Nucleotide Variants Using Multiplex Droplet Digital PCR in Liquid Biopsies from Children with Medulloblastoma
Source: Cancers (Basel). 2023 Mar 25;15(7):1972. doi: 10.3390/cancers15071972 (PMC10092983; doi:10.3390/cancers15071972)

# MB24 NTC

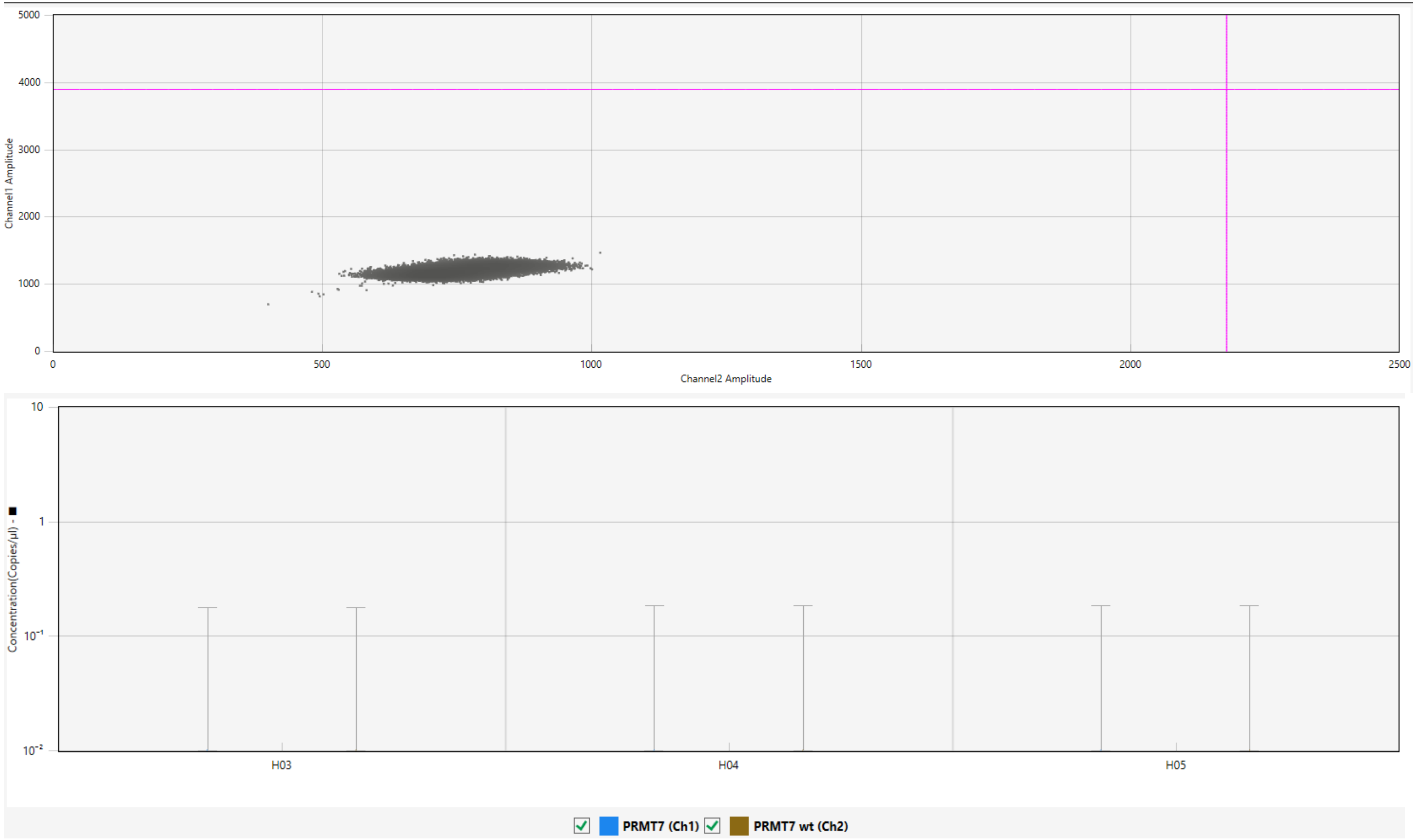

5000
4000
3000
2000
1000
0
Channel1 Amplitude
0
500
1000
1500
2000
2500
Channel2 Amplitude
10
1
$10^{-1}$
$10^{-2}$
Concentration(Copies/µl) -
H03
H04
H05
PRMT7 (Ch1)
PRMT7 wt (Ch2)

# MB24 NC pl & gNC

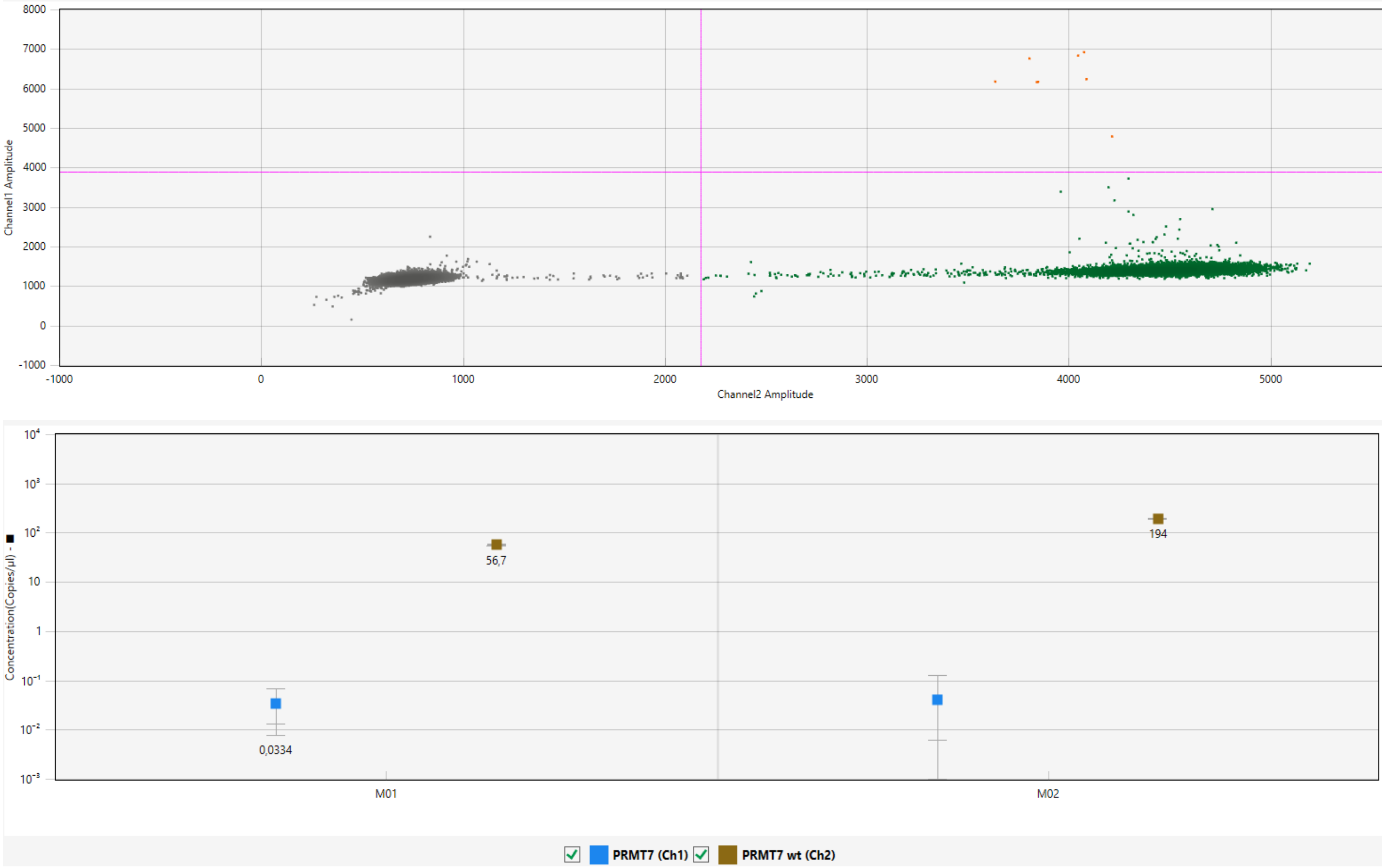

# MB24 example of clusters (PC 1:10)

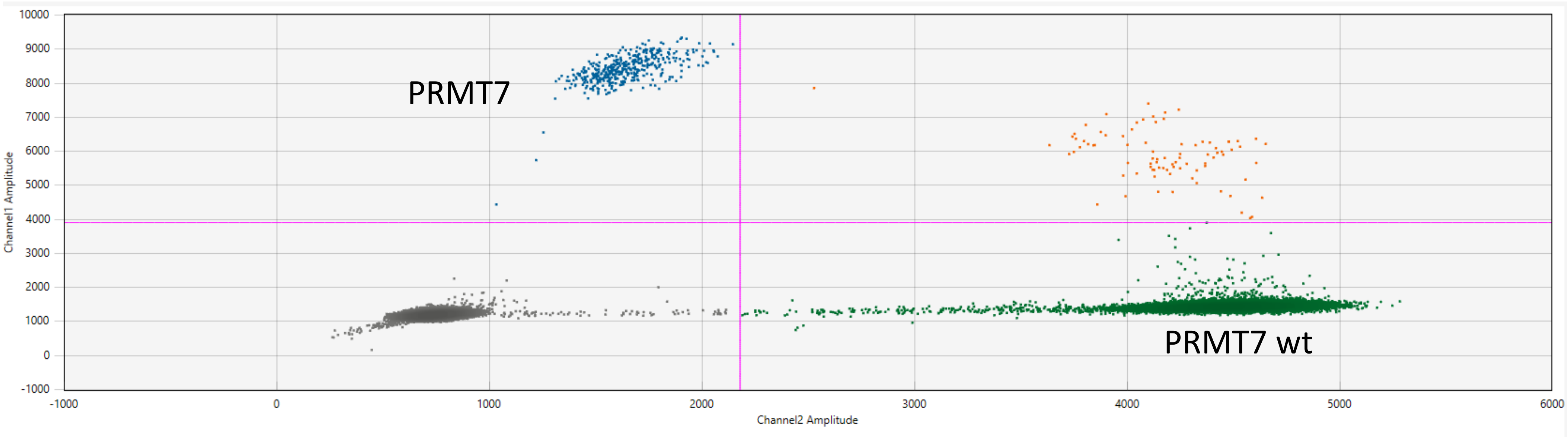

# MB24 Dilution series: LoD 1:1000, LoQ 1:100.

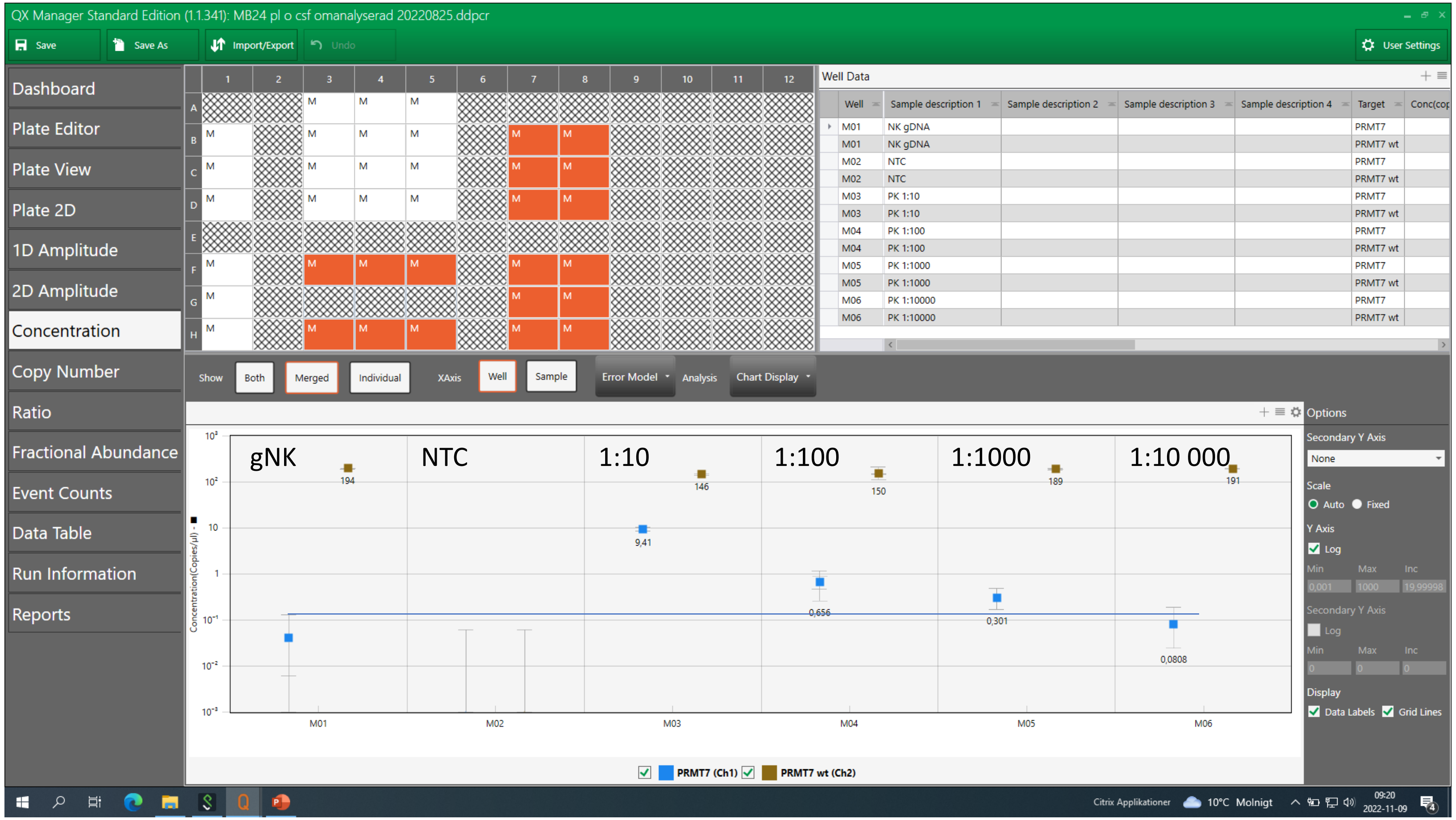

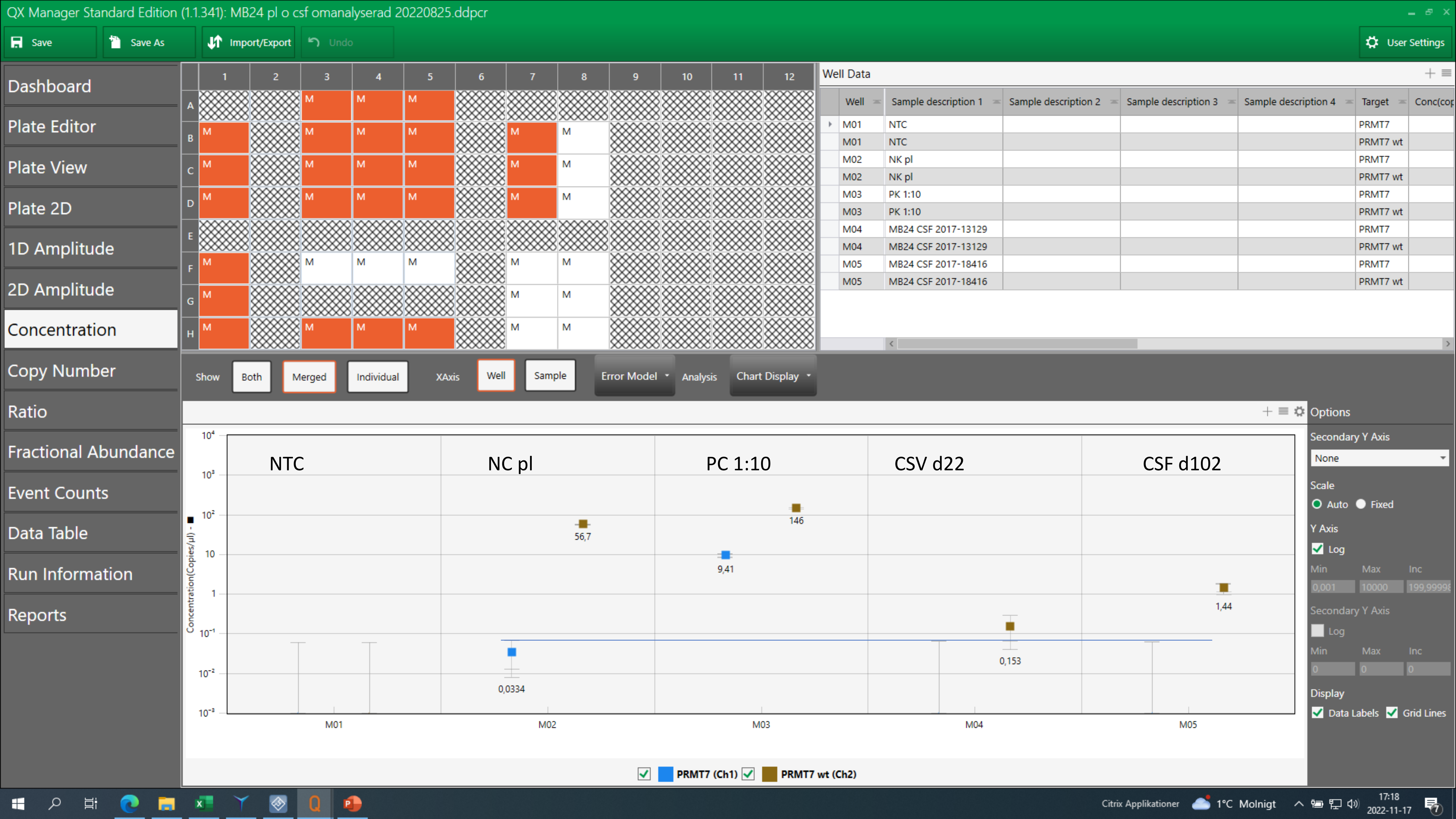
QX Manager Standard Edition (1.1.341): MB24 pl o csf omanalyserad 20220825.ddpcr
Save
Save As
Import/Export
Undo
User Settings
Dashboard
Plate Editor
Plate View
Plate 2D
1D Amplitude
2D Amplitude
Concentration
Copy Number
Ratio
Fractional Abundance
Event Counts
Data Table
Run Information
Reports
Well Data
Well
Sample description 1
Sample description 2
Sample description 3
Sample description 4
Target
M01 NTC PRMT7
M01 NTC PRMT7 wt
M02 NK pl PRMT7
M02 NK pl PRMT7 wt
M03 PK 1:10 PRMT7
M03 PK 1:10 PRMT7 wt
M04 MB24 CSF 2017-13129 PRMT7
M04 MB24 CSF 2017-13129 PRMT7 wt
M05 MB24 CSF 2017-18416 PRMT7
M05 MB24 CSF 2017-18416 PRMT7 wt
Show
Both
Merged
Individual
XAxis
Well
Sample
Error Model
Analysis
Chart Display
Options
Secondary Y Axis
None
Scale
Auto
Fixed
Y Axis
Log
Min
Max
Inc
0,001
10000
Display
Data Labels
Grid Lines
NTC
NC pl
PC 1:10
CSV d22
CSF d102
Concentration(Copies/µl)
56,7
146
9,41
1,44
0,153
0,0334
M01
M02
M03
M04
M05
PRMT7 (Ch1)
PRMT7 wt (Ch2)
Citrix Applikationer
1°C Molnigt
17:18
2022-11-17

# MB24 CSF d22

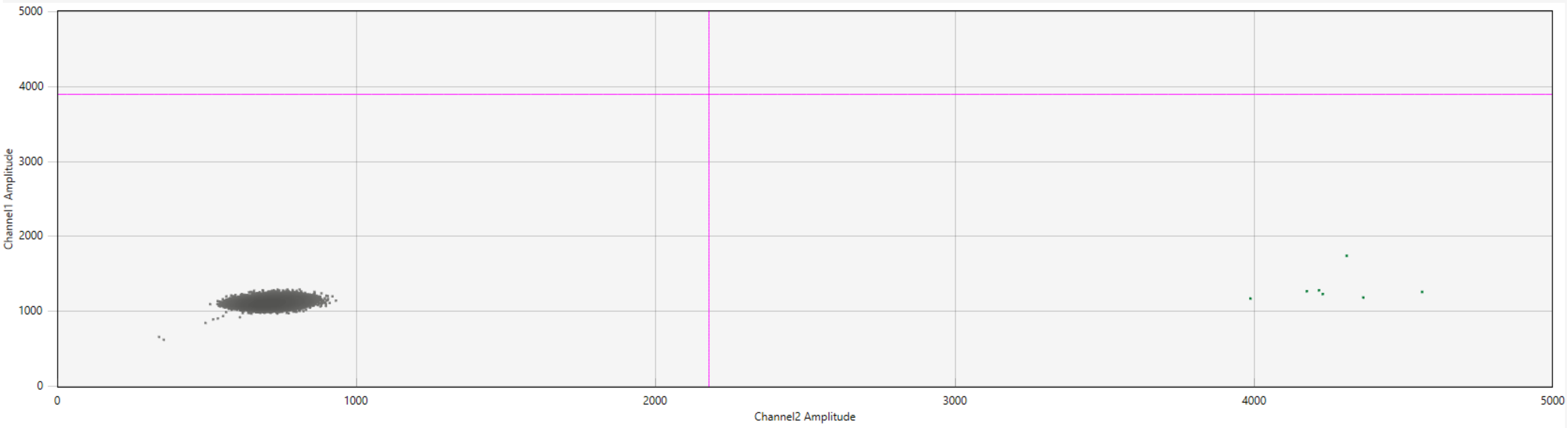

5000
4000
3000
2000
1000
0
Channel1 Amplitude
0
1000
2000
3000
4000
5000
Channel2 Amplitude

# MB24 CSF d102

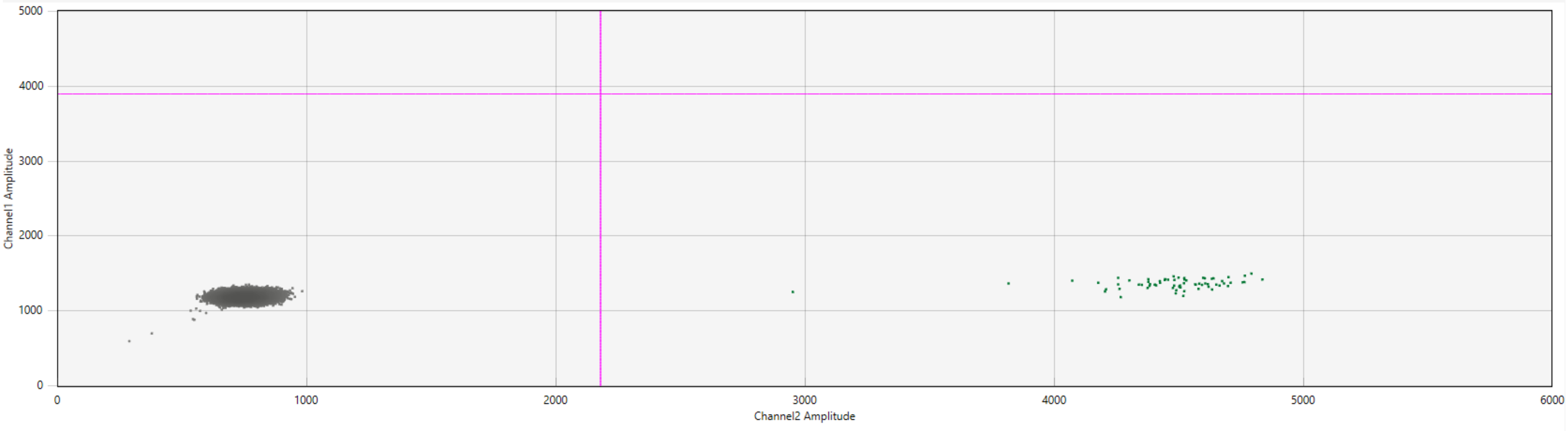

Supplement: Supplementary file 1 [file cancers-15-01972-s001.zip › File S2 QX Manager Software output data on liquid biopsies/Liquid biopsies MB24.pdf]
