# Supplementary material for: Simultaneous Ultra-Sensitive Detection of Structural and Single Nucleotide Variants Using Multiplex Droplet Digital PCR in Liquid Biopsies from Children with Medulloblastoma
Source: Cancers (Basel). 2023 Mar 25;15(7):1972. doi: 10.3390/cancers15071972 (PMC10092983; doi:10.3390/cancers15071972)
Supplement: Supplementary file 1 [file cancers-15-01972-s001.zip › File S2 QX Manager Software output data on liquid biopsies/Liquid biopsies MB27.pdf]

MB27 NTC

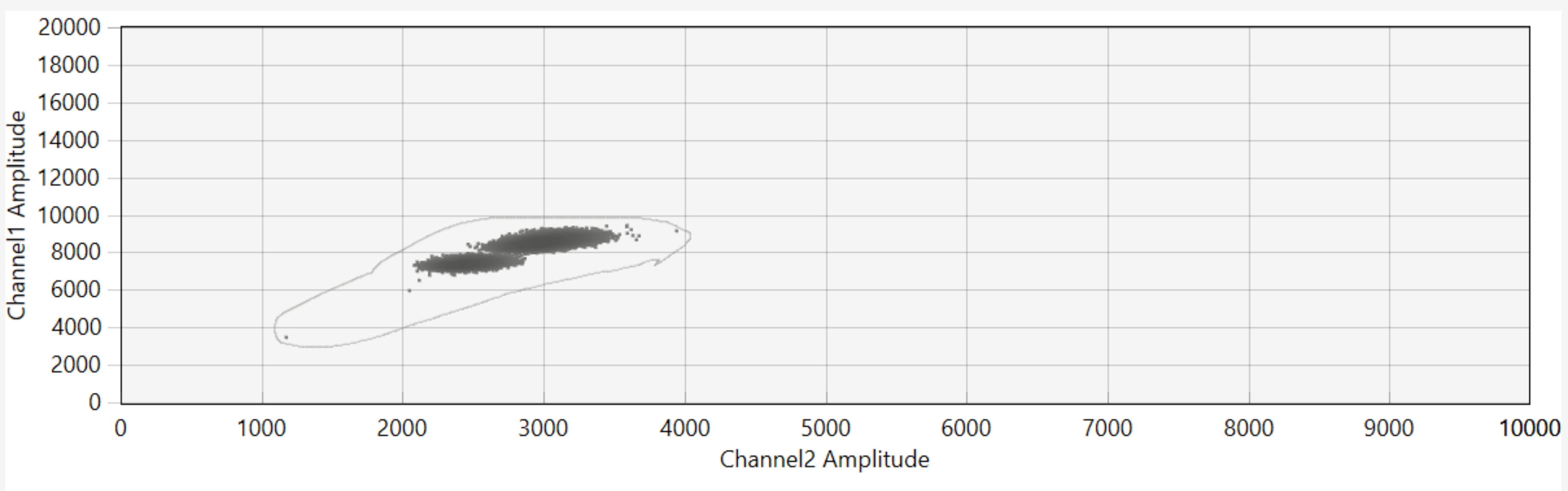

20000
18000
16000
14000
12000
10000
8000
6000
4000
2000
0
Channel1 Amplitude
0
1000
2000
3000
4000
5000
6000
7000
8000
9000
10000
Channel2 Amplitude

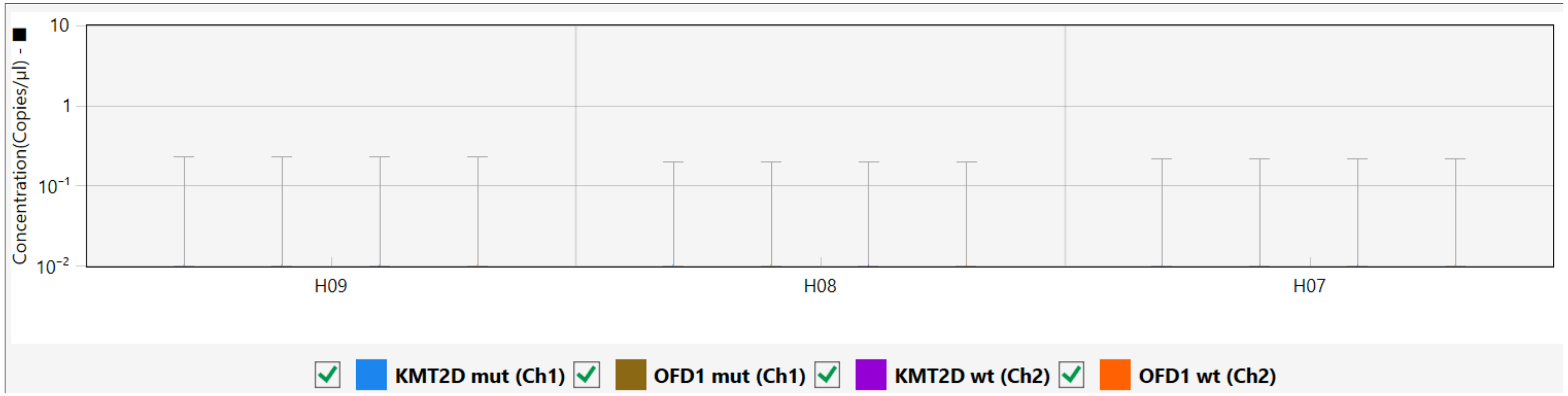

Concentration(Copies/µl)
10
1
10^{-1}
10^{-2}
H09
H08
H07
KMT2D mut (Ch1)
OFD1 mut (Ch1)
KMT2D wt (Ch2)
OFD1 wt (Ch2)

MB27 NC pl & gNC

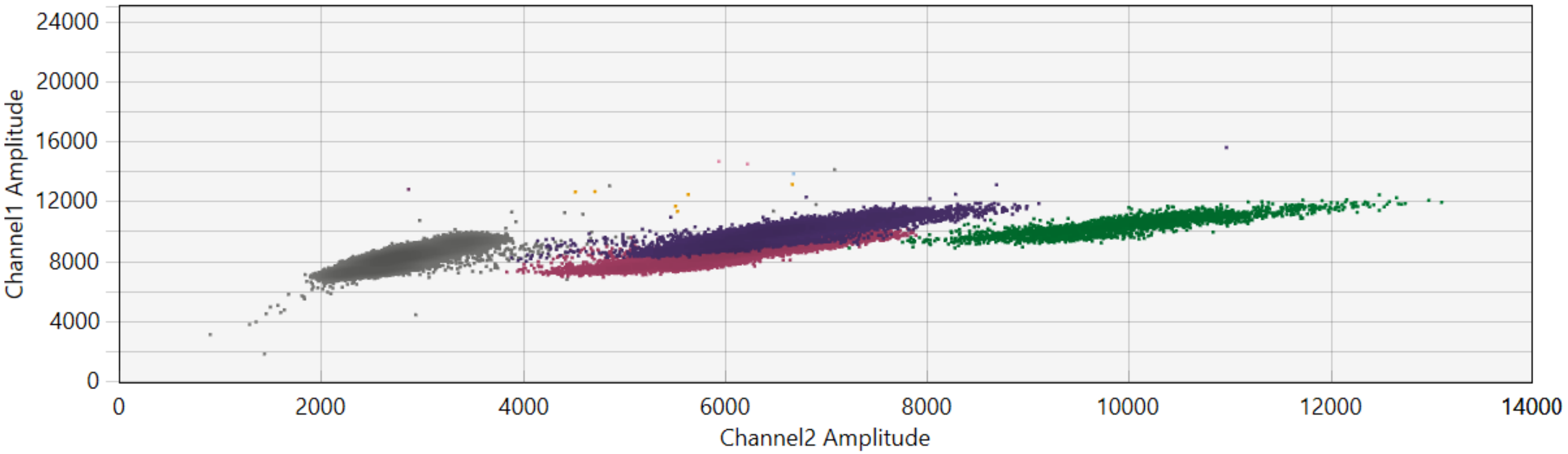

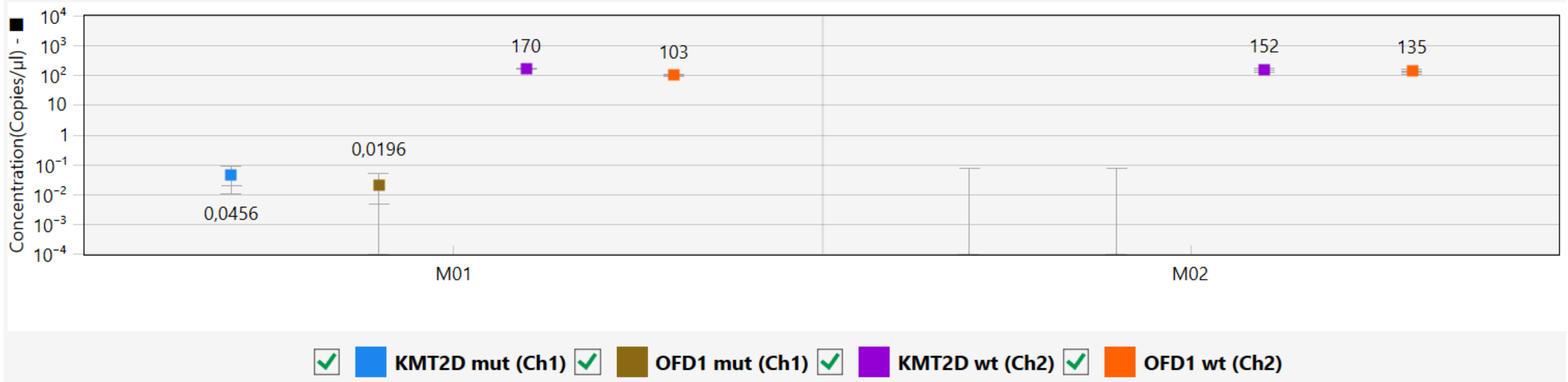

MB27 Example of clustsers (PC 1:10)

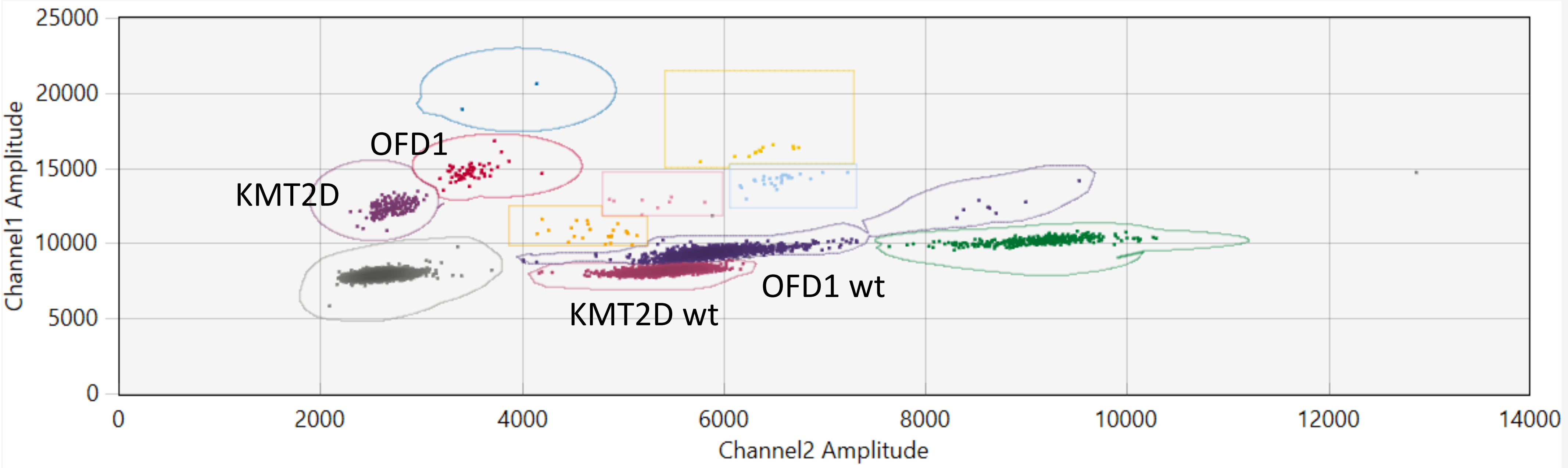

MB27 Dilution series: LoD & LoQ KMT2D 1:100. LoD OFD1 1:1000, LoQ 1:100.

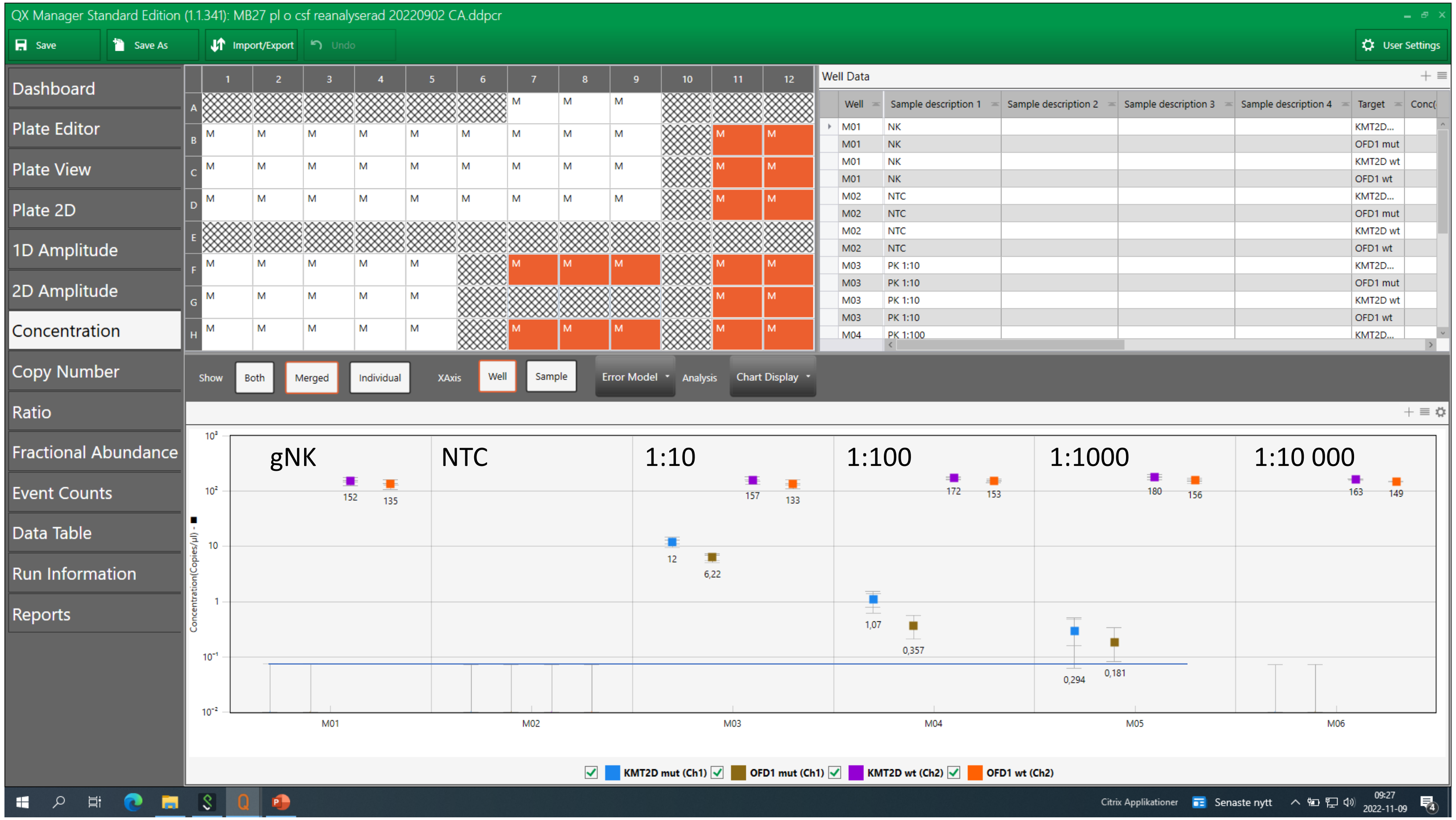

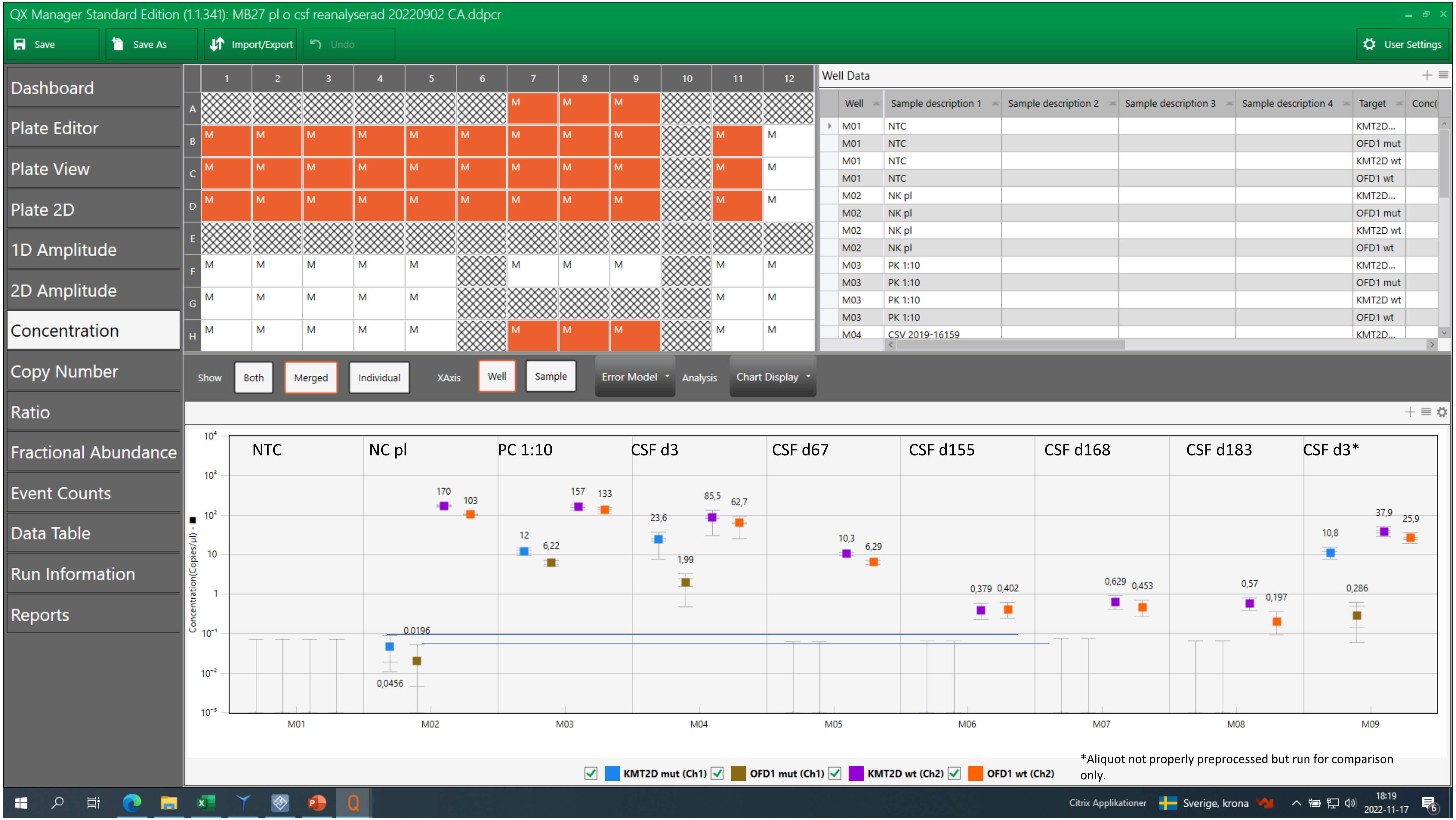

*Aliquot not properly preprocessed but run for comparison only.

MB27 CSF d3

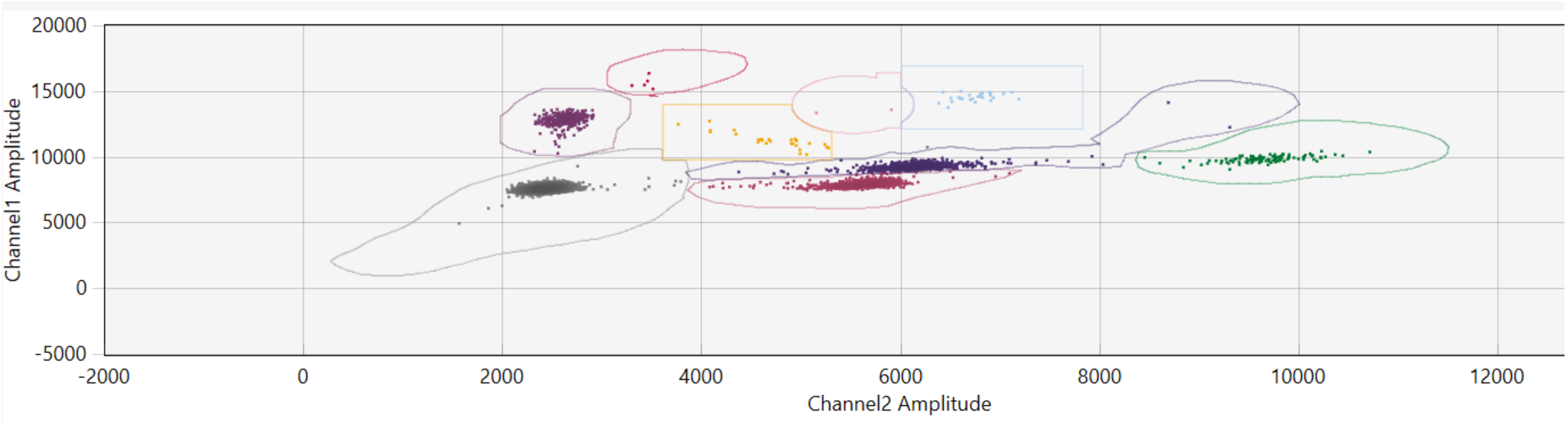
20000
15000
10000
5000
0
-5000
Channel1 Amplitude
-2000
0
2000
4000
6000
8000
10000
12000
Channel2 Amplitude

MB27 CSF d67

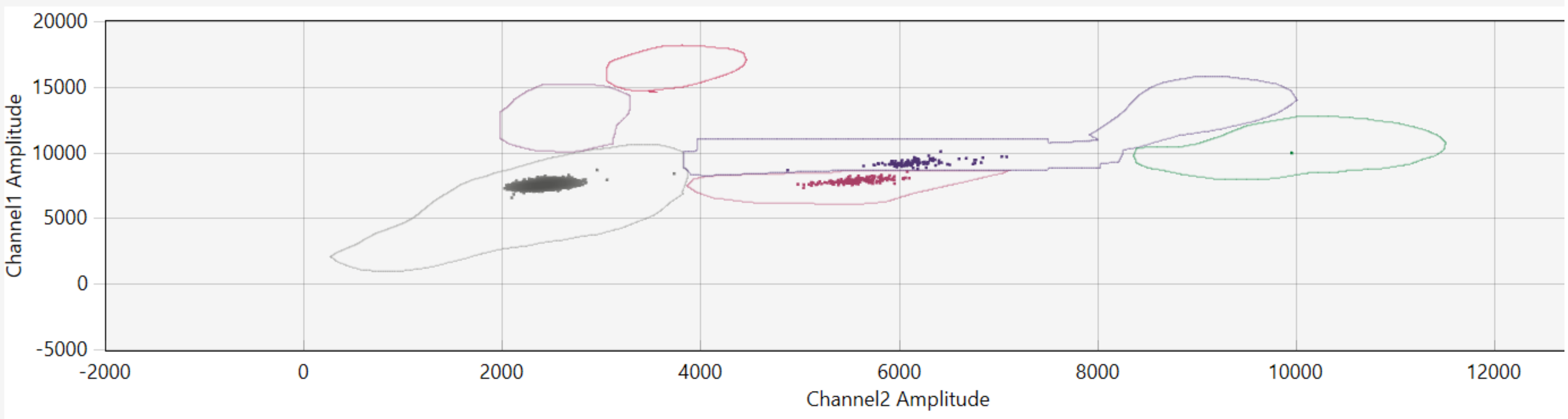
20000
15000
10000
5000
0
-5000
Channel1 Amplitude
-2000
0
2000
4000
6000
8000
10000
12000
Channel2 Amplitude

MB27 CSF d155

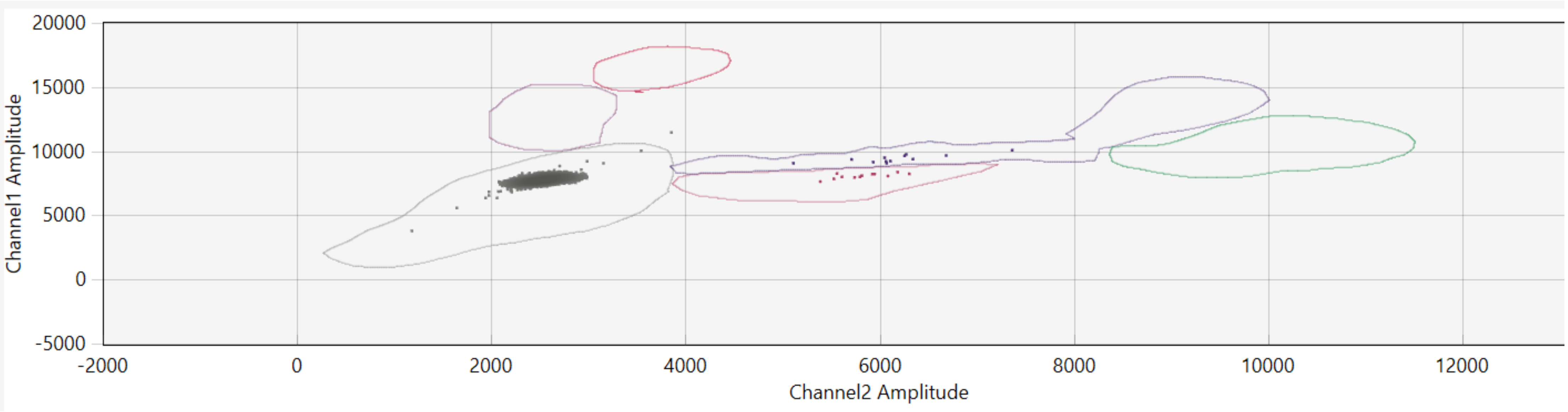

MB27 CSF d168

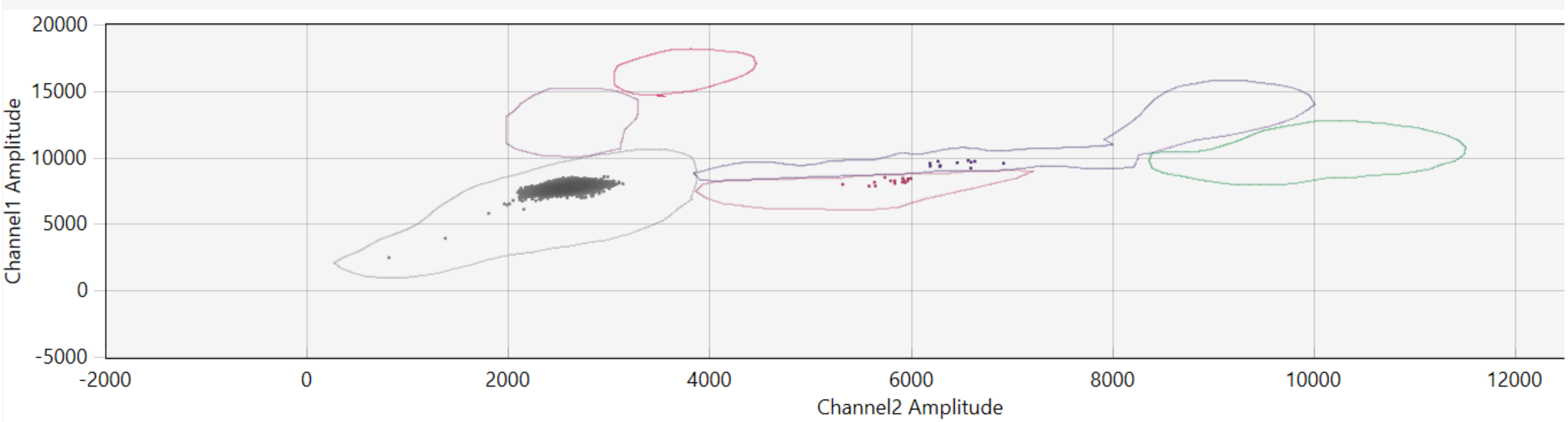

MB27 CSF d183

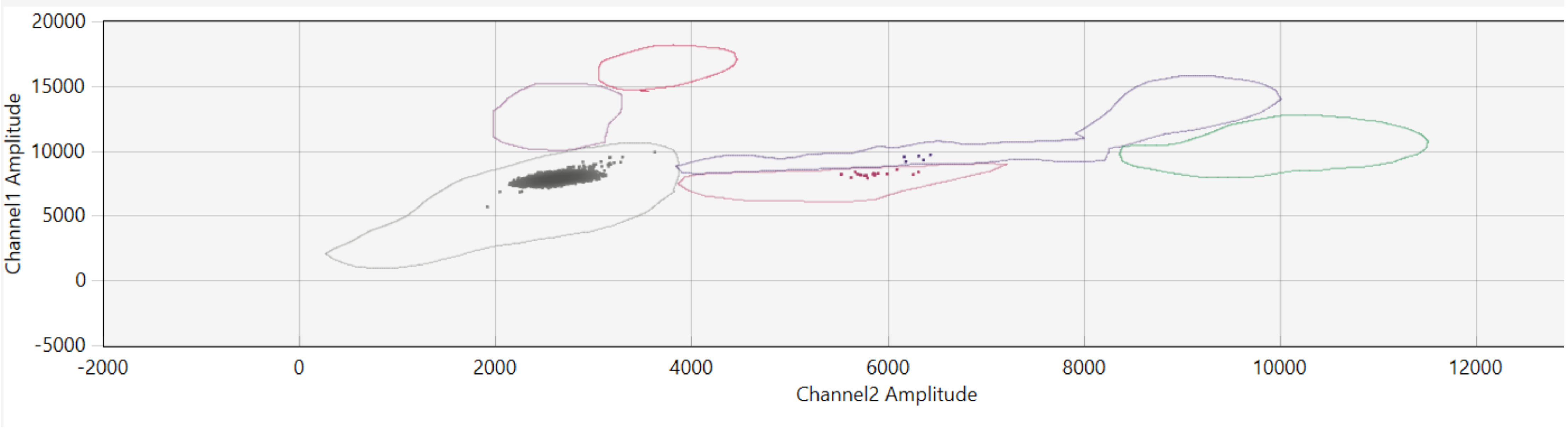

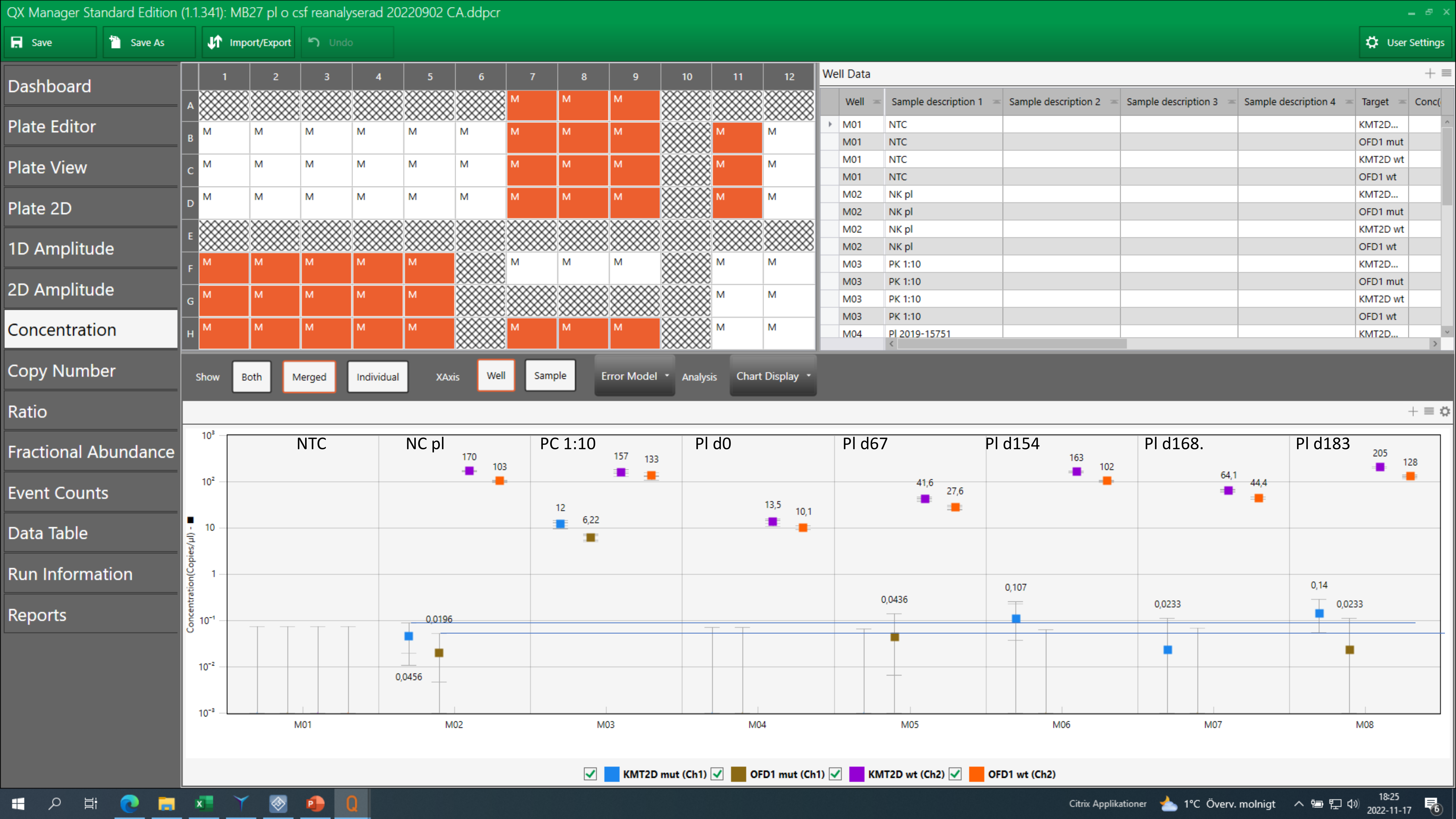

QX Manager Standard Edition (1.1.341): MB27 pl o csf reanalyserad 20220902 CA.ddpcr
Save
Save As
Import/Export
Undo
User Settings
Dashboard
Plate Editor
Plate View
Plate 2D
1D Amplitude
2D Amplitude
Concentration
Copy Number
Ratio
Fractional Abundance
Event Counts
Data Table
Run Information
Reports
Well Data
Well | Sample description 1 | Sample description 2 | Sample description 3 | Sample description 4 | Target | Conc(
M01 NTC KMT2D...
M01 NTC OFD1 mut
M01 NTC KMT2D wt
M01 NTC OFD1 wt
M02 NK pl KMT2D...
M02 NK pl OFD1 mut
M02 NK pl KMT2D wt
M02 NK pl OFD1 wt
M03 PK 1:10 KMT2D...
M03 PK 1:10 OFD1 mut
M03 PK 1:10 KMT2D wt
M03 PK 1:10 OFD1 wt
M04 Pl 2019-15751 KMT2D...
Show
Both
Merged
Individual
XAxis
Well
Sample
Error Model
Analysis
Chart Display
NTC
NC pl
PC 1:10
Pl d0
Pl d67
Pl d154
Pl d168.
Pl d183
Concentration(Copies/µl) -
M01
M02
M03
M04
M05
M06
M07
M08
KMT2D mut (Ch1)
OFD1 mut (Ch1)
KMT2D wt (Ch2)
OFD1 wt (Ch2)
Citrix Applikationer
1°C Överv. molnigt
18:25
2022-11-17

MB27 Pl d0

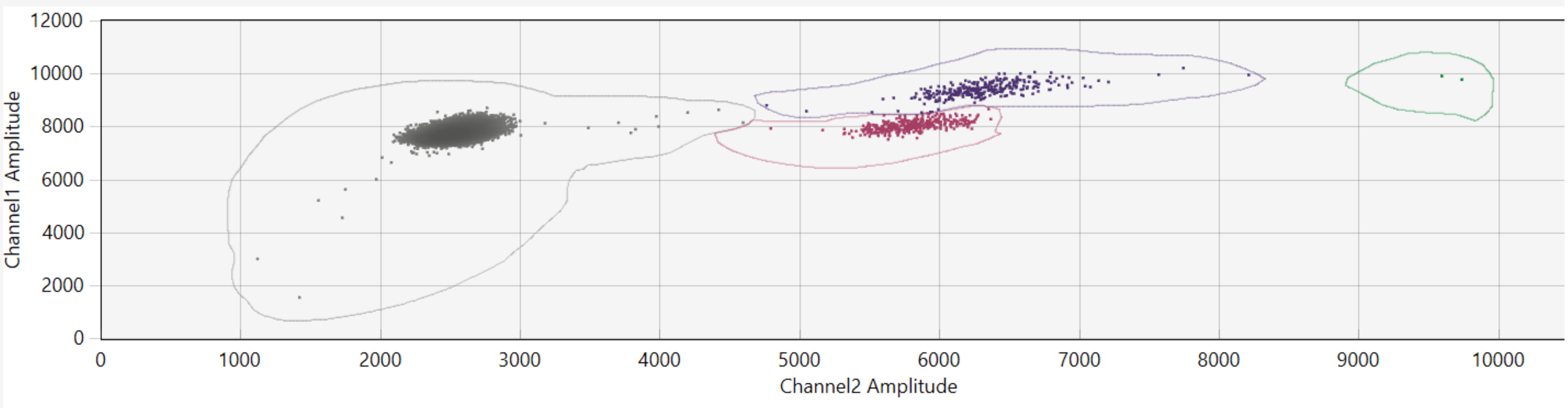

12000
10000
8000
6000
4000
2000
0
Channel1 Amplitude
0
1000
2000
3000
4000
5000
6000
7000
8000
9000
10000
Channel2 Amplitude

MB27 Pl d67

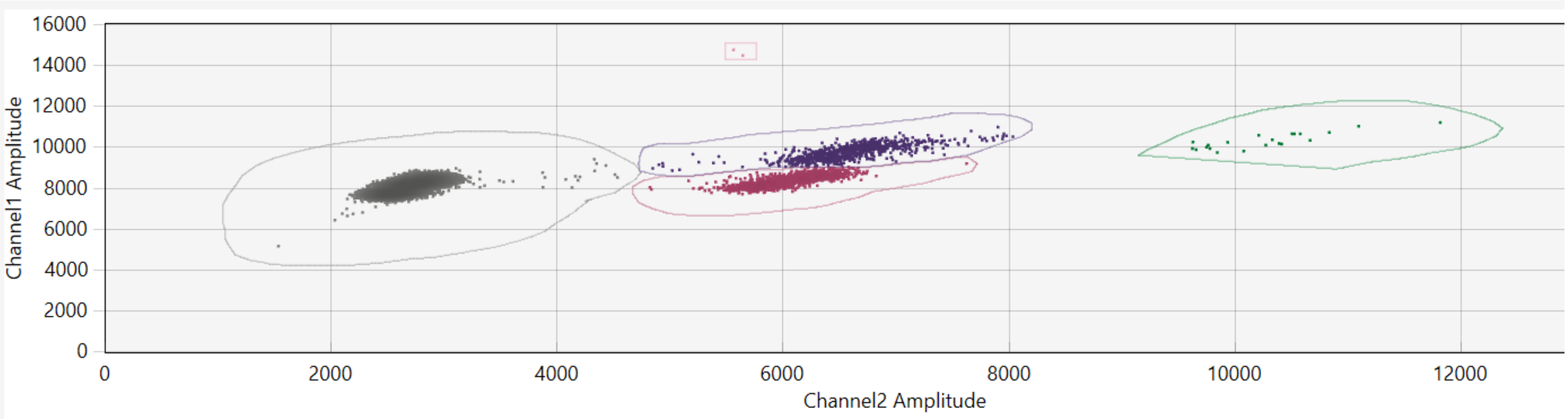

16000
14000
12000
10000
8000
6000
4000
2000
0
Channel1 Amplitude
0
2000
4000
6000
8000
10000
12000
Channel2 Amplitude

MB27 Pl d154

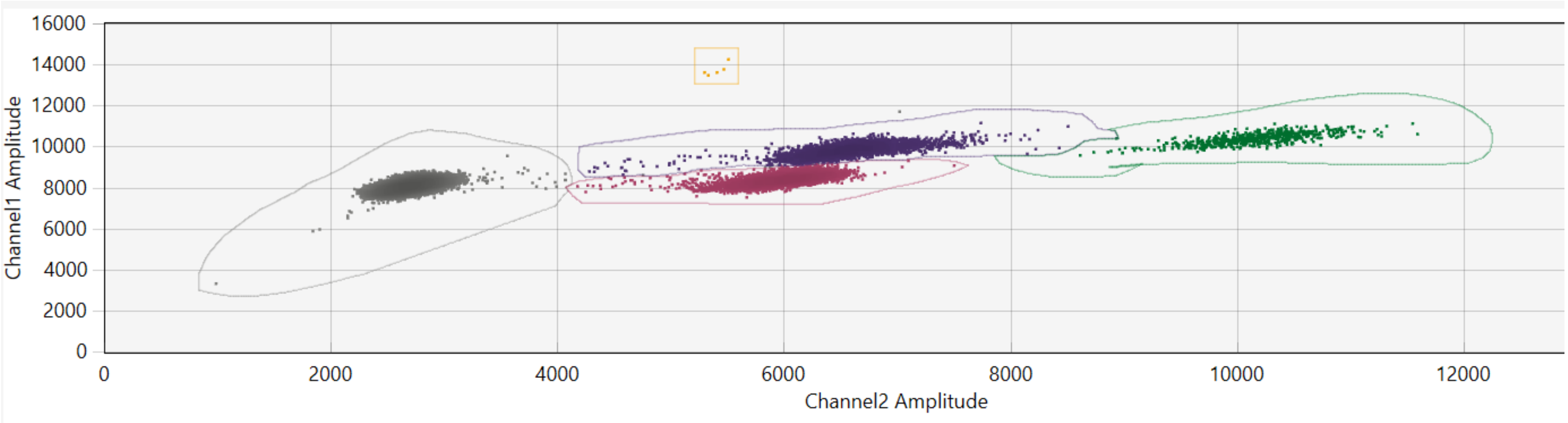

16000
14000
12000
10000
8000
6000
4000
2000
0
Channel1 Amplitude
0
2000
4000
6000
8000
10000
12000
Channel2 Amplitude

MB27 Pl d168

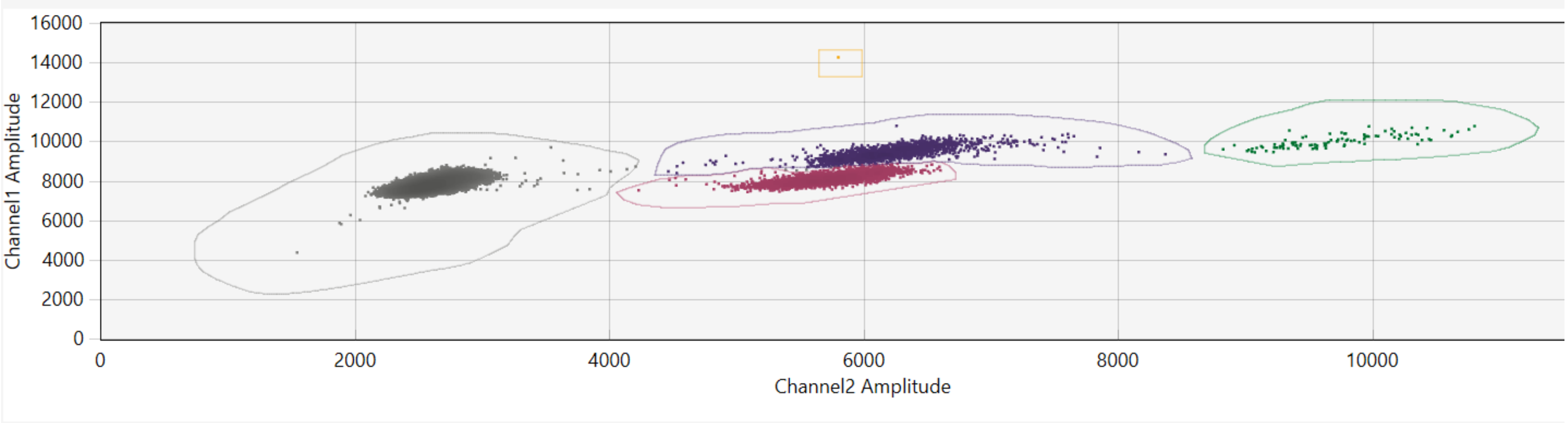

16000
14000
12000
10000
8000
6000
4000
2000
0
Channel1 Amplitude
0
2000
4000
6000
8000
10000
Channel2 Amplitude

MB27 Pl d183

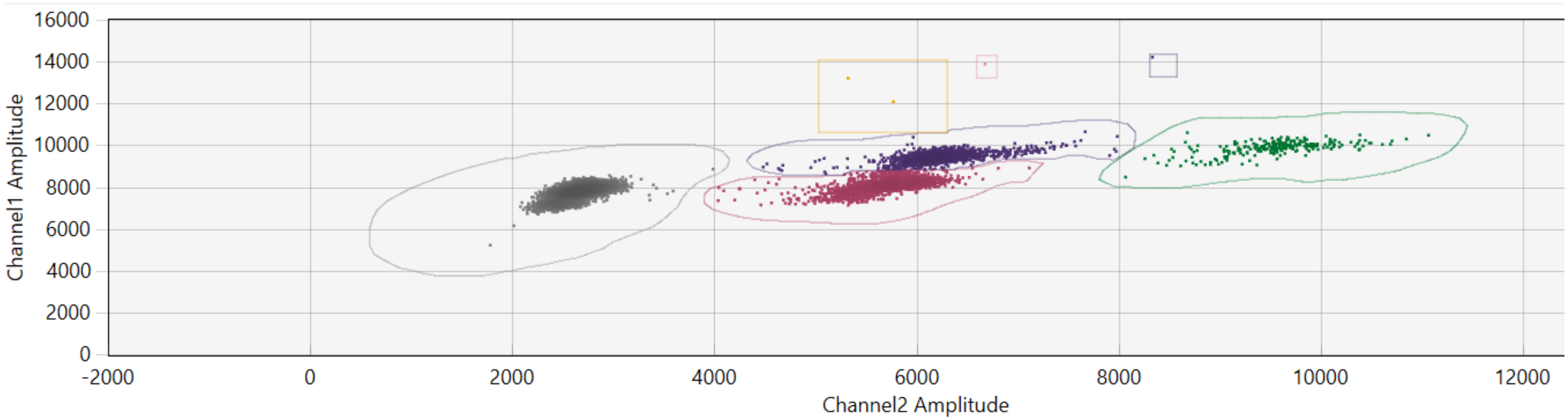

16000
14000
12000
10000
8000
6000
4000
2000
0
Channel1 Amplitude
-2000
0
2000
4000
6000
8000
10000
12000
Channel2 Amplitude
