# Supplementary material for: Simultaneous Ultra-Sensitive Detection of Structural and Single Nucleotide Variants Using Multiplex Droplet Digital PCR in Liquid Biopsies from Children with Medulloblastoma
Source: Cancers (Basel). 2023 Mar 25;15(7):1972. doi: 10.3390/cancers15071972 (PMC10092983; doi:10.3390/cancers15071972)
Supplement: Supplementary file 1 [file cancers-15-01972-s001.zip › File S2 QX Manager Software output data on liquid biopsies/Liquid biopsies MB31.pdf]

MB31 NTC

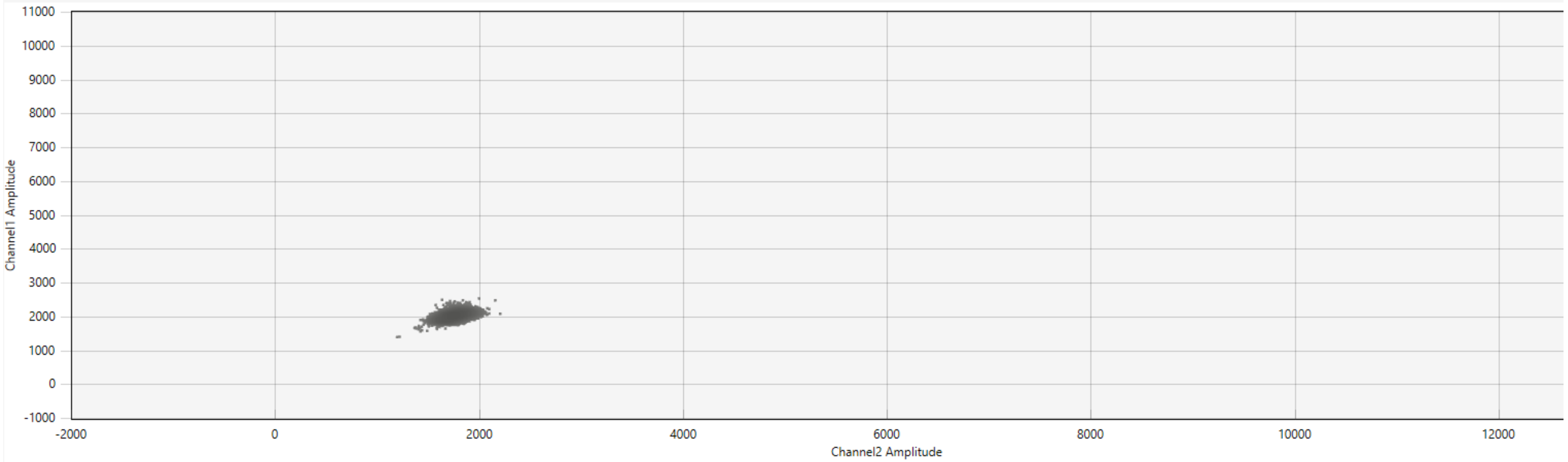

Channel1 Amplitude
11000
10000
9000
8000
7000
6000
5000
4000
3000
2000
1000
0
-1000
-2000
0
2000
4000
6000
8000
10000
12000
Channel2 Amplitude

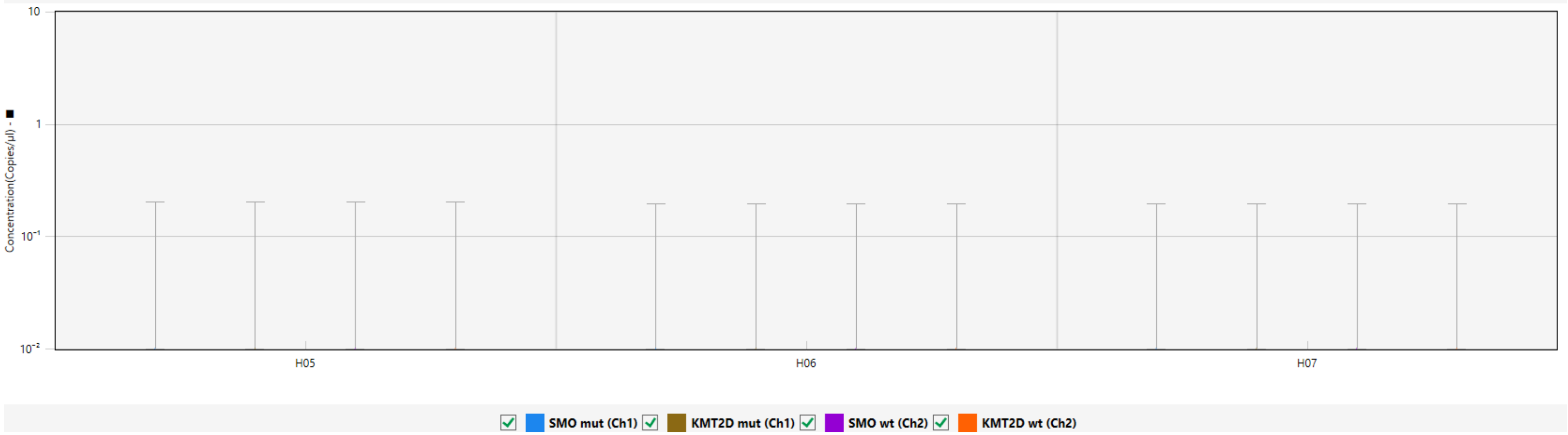

Concentration(Copies/µl)
10
1
10⁻¹
10⁻²
H05
H06
H07
SMO mut (Ch1)
KMT2D mut (Ch1)
SMO wt (Ch2)
KMT2D wt (Ch2)

MB31 NC pl and gNC

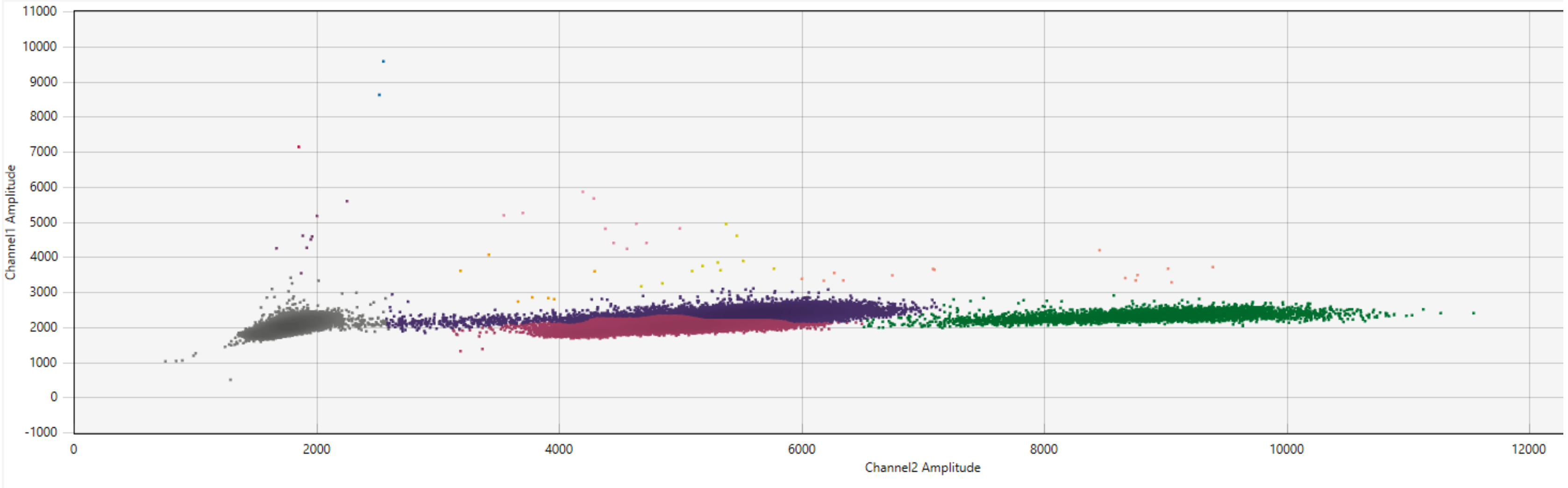

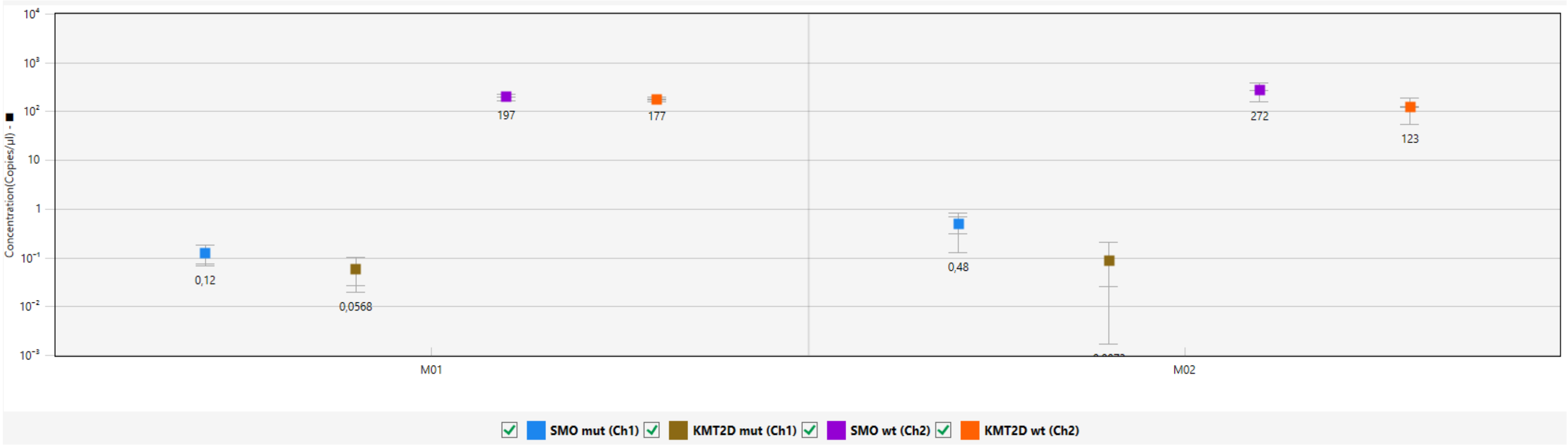

MB31 Example of clusters (PC 1:10)

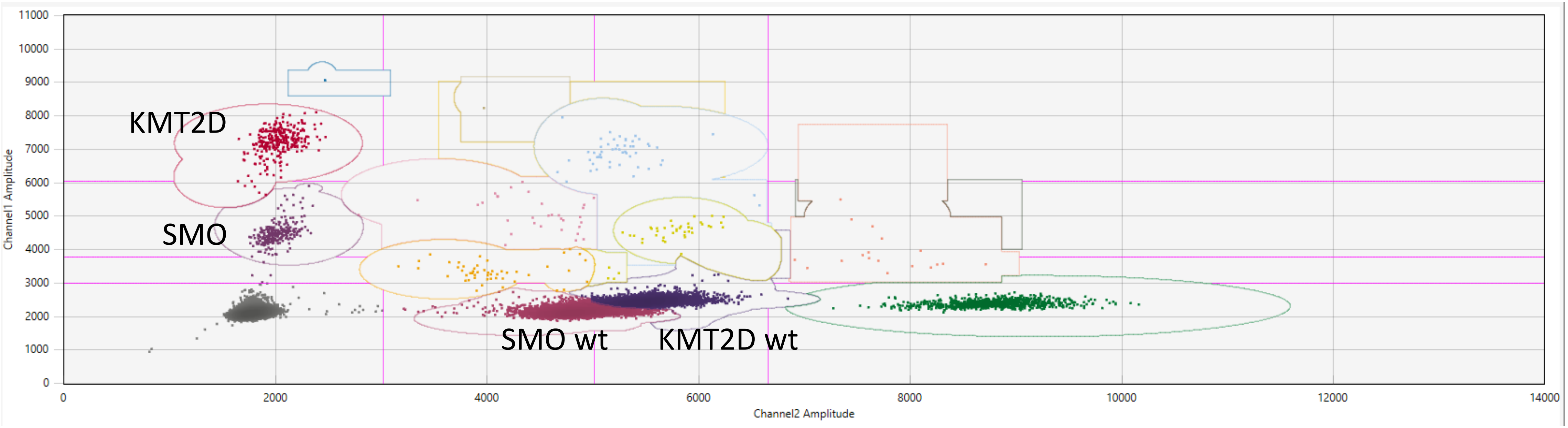

MB31 Dilution series: LoD & LoQ 1:100 both targets.

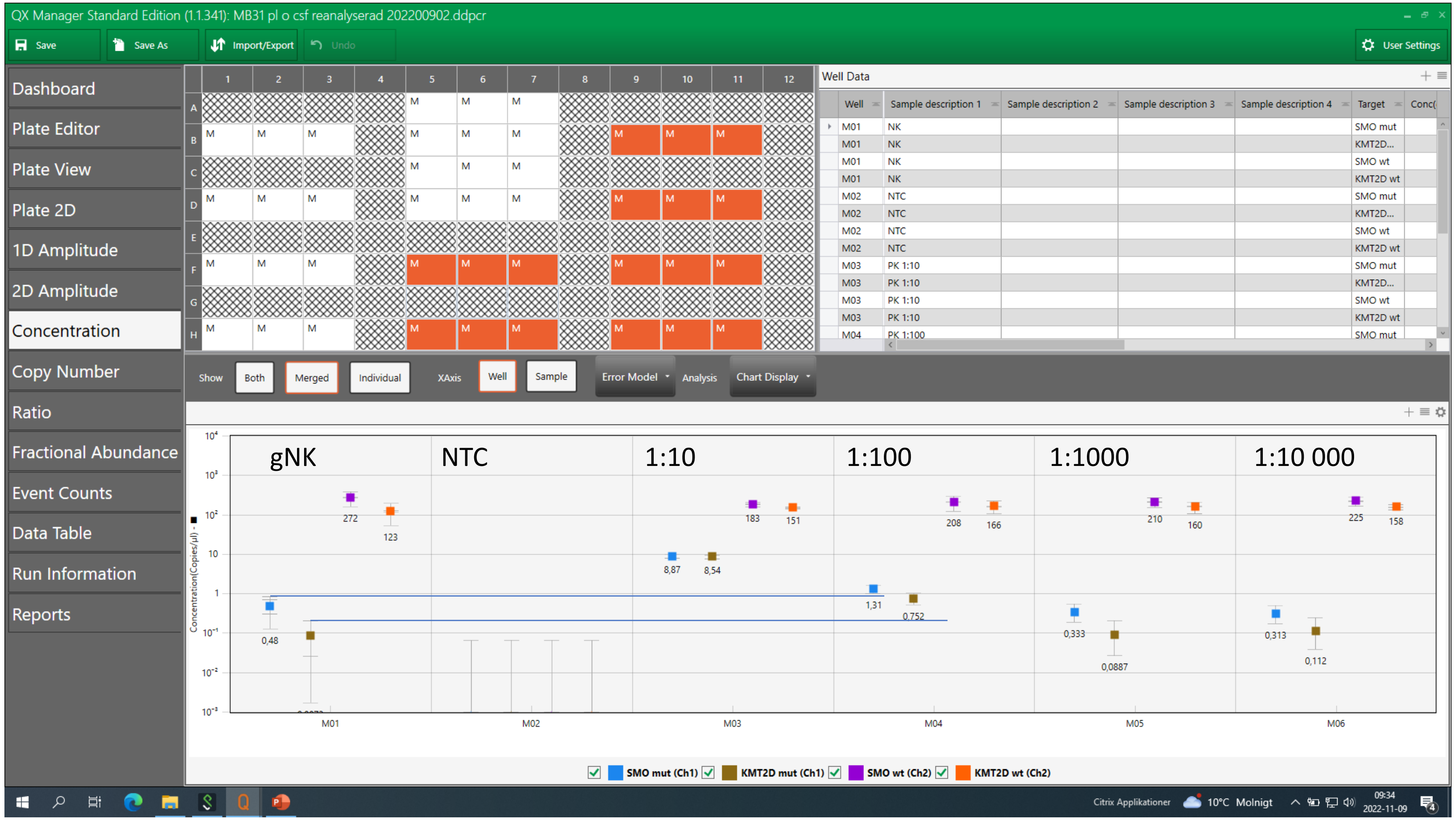

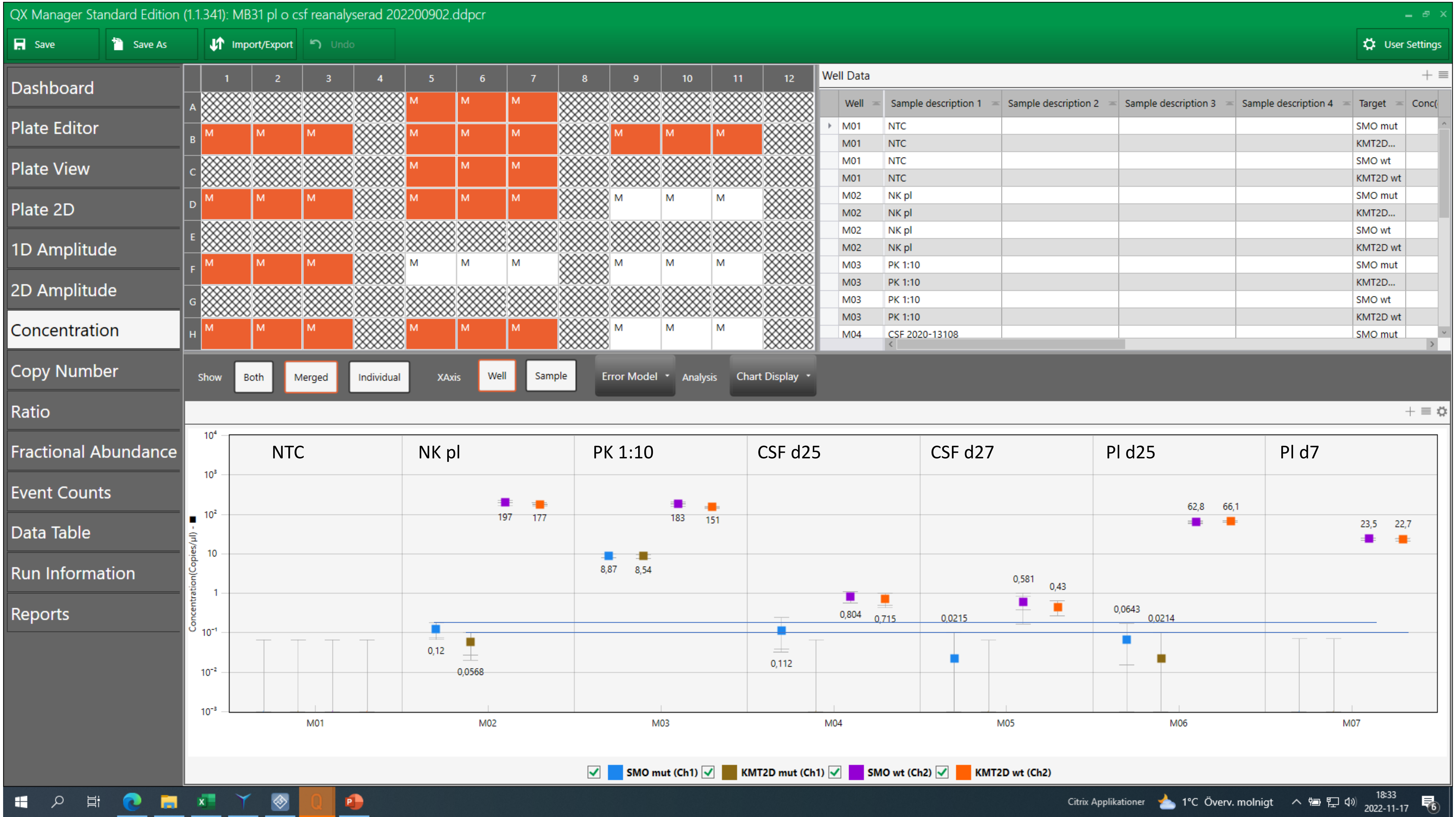
QX Manager Standard Edition (1.1.341): MB31 pl o csf reanalyserad 202200902.ddpcr
Save
Save As
Import/Export
Undo
User Settings
Dashboard
Plate Editor
Plate View
Plate 2D
1D Amplitude
2D Amplitude
Concentration
Copy Number
Ratio
Fractional Abundance
Event Counts
Data Table
Run Information
Reports
Well Data
Well | Sample description 1 | Sample description 2 | Sample description 3 | Sample description 4 | Target | Conc(
M01 | NTC | SMO mut
M01 | NTC | KMT2D...
M01 | NTC | SMO wt
M01 | NTC | KMT2D wt
M02 | NK pl | SMO mut
M02 | NK pl | KMT2D...
M02 | NK pl | SMO wt
M02 | NK pl | KMT2D wt
M03 | PK 1:10 | SMO mut
M03 | PK 1:10 | KMT2D...
M03 | PK 1:10 | SMO wt
M03 | PK 1:10 | KMT2D wt
M04 | CSF 2020-13108 | SMO mut
Show
Both
Merged
Individual
XAxis
Well
Sample
Error Model
Analysis
Chart Display
NTC
NK pl
PK 1:10
CSF d25
CSF d27
Pl d25
Pl d7
197
177
183
151
8,87
8,54
0,12
0,0568
0,804
0,715
0,112
0,581
0,43
0,0215
62,8
66,1
0,0643
0,0214
23,5
22,7
Concentration(Copies/µl)
M01
M02
M03
M04
M05
M06
M07
SMO mut (Ch1)
KMT2D mut (Ch1)
SMO wt (Ch2)
KMT2D wt (Ch2)
Citrix Applikationer
1°C Överv. molnigt
18:33
2022-11-17

MB31 CSF d25

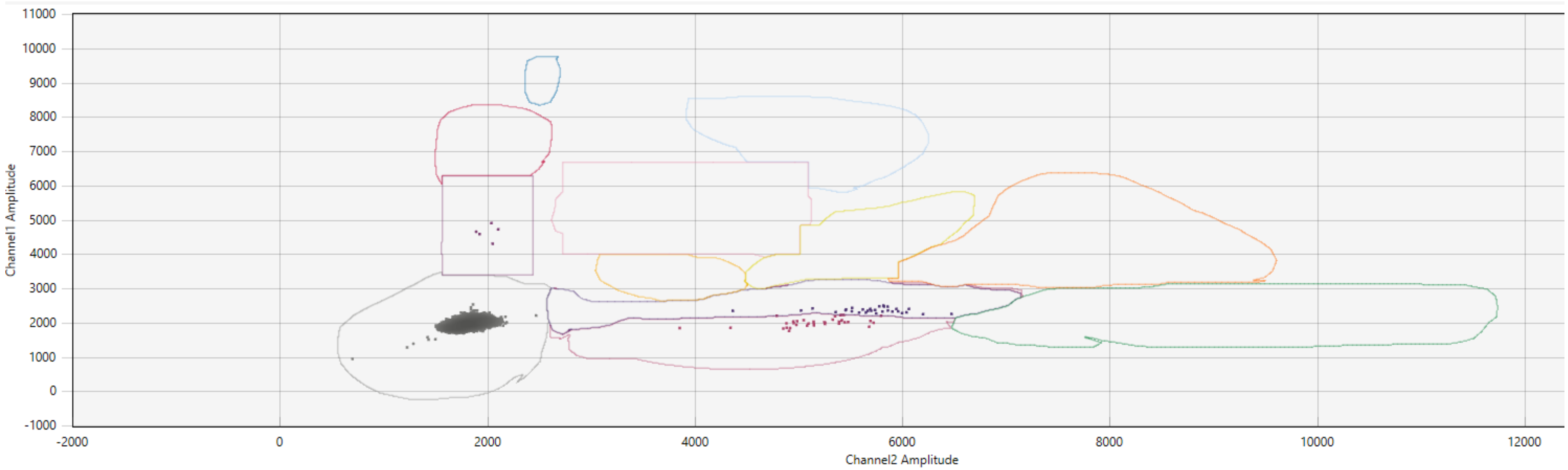

11000
10000
9000
8000
7000
6000
5000
4000
3000
2000
1000
0
-1000
Channel1 Amplitude
-2000
0
2000
4000
6000
8000
10000
12000
Channel2 Amplitude

MB31 CSF d27

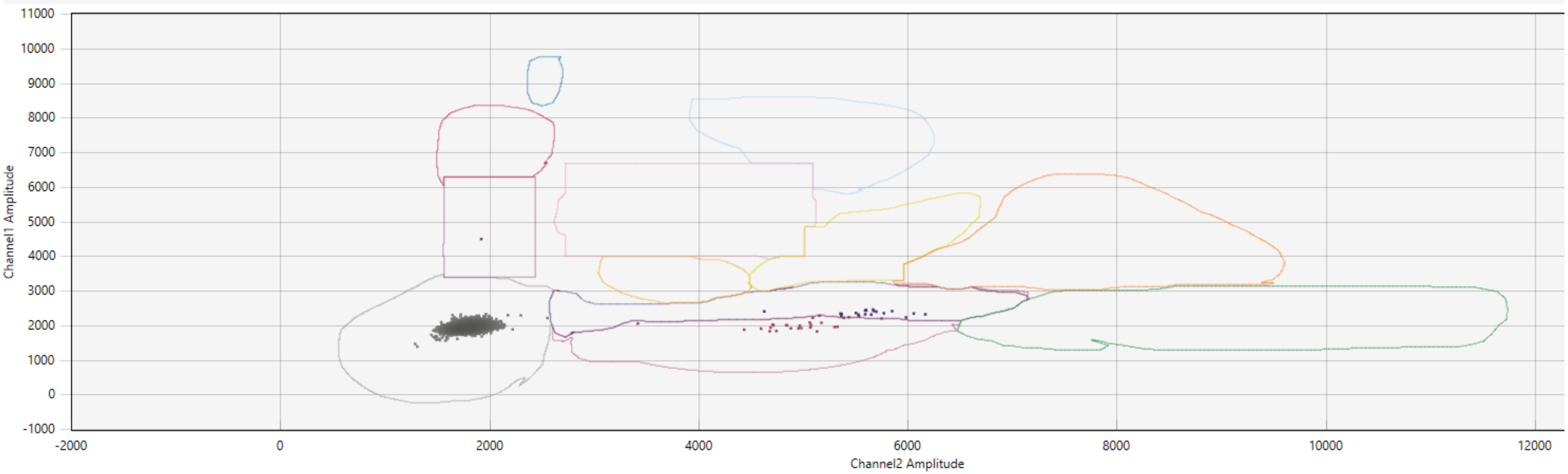

11000
10000
9000
8000
7000
6000
5000
4000
3000
2000
1000
0
-1000
Channel1 Amplitude
-2000
0
2000
4000
6000
8000
10000
12000
Channel2 Amplitude

MB31 Pl d25

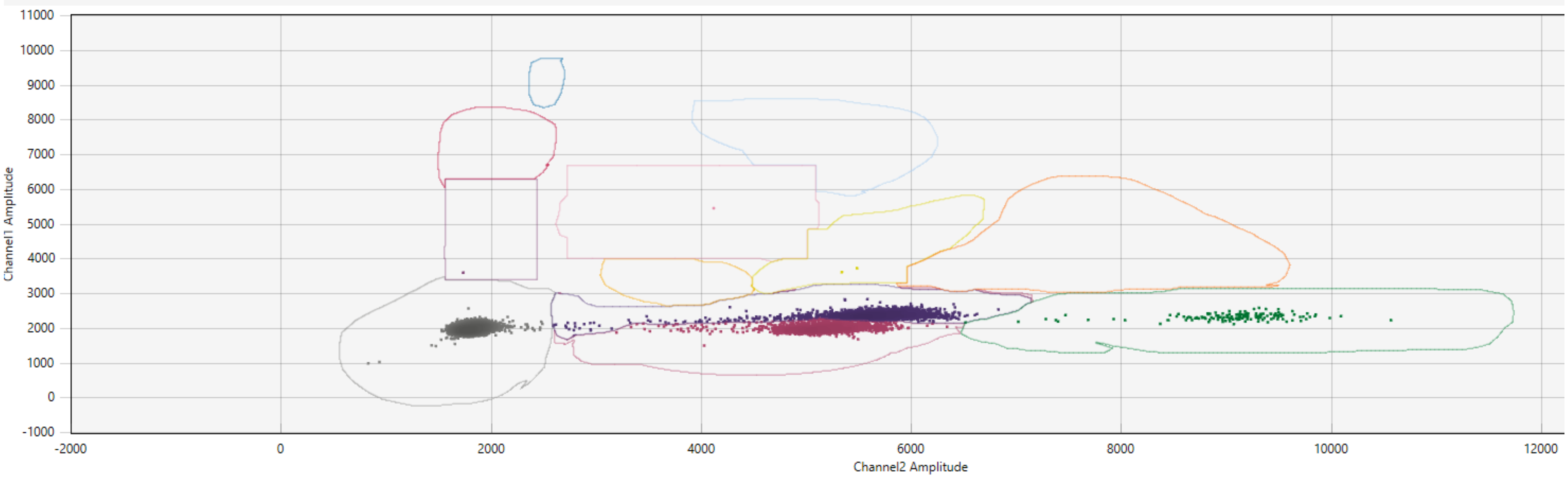

11000
10000
9000
8000
7000
6000
5000
4000
3000
2000
1000
0
-1000
Channel1 Amplitude
-2000
0
2000
4000
6000
8000
10000
12000
Channel2 Amplitude

MB31 Pl d27

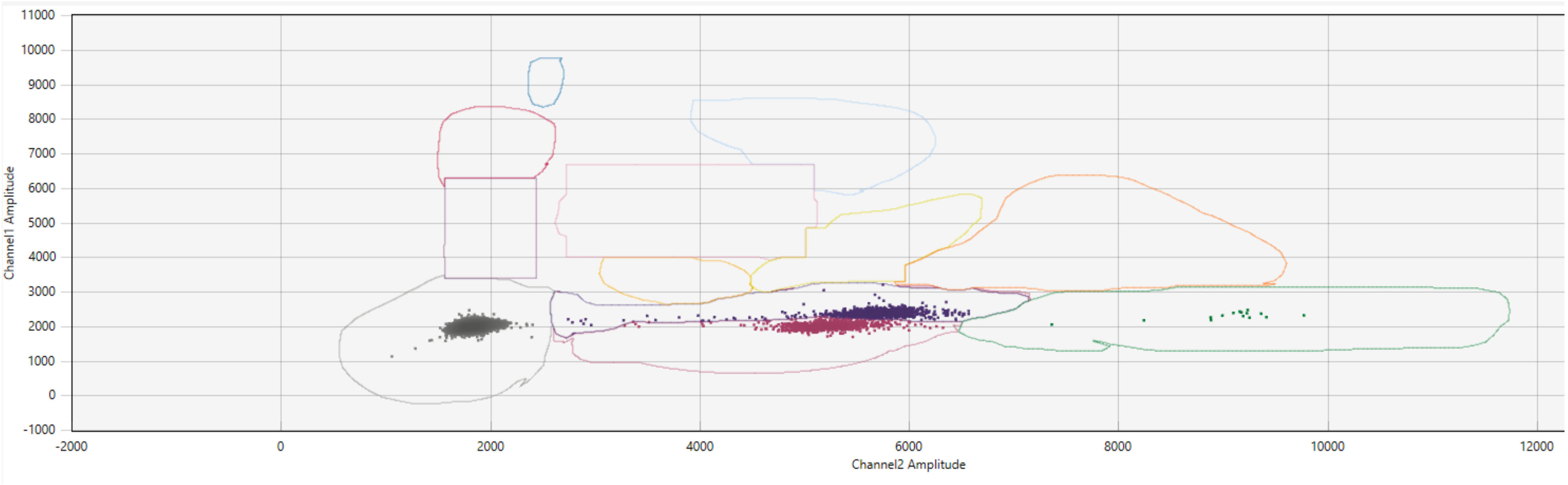

11000
10000
9000
8000
7000
6000
5000
4000
3000
2000
1000
0
-1000
Channel1 Amplitude
-2000
0
2000
4000
6000
8000
10000
12000
Channel2 Amplitude
