# Supplementary material for: Simultaneous Ultra-Sensitive Detection of Structural and Single Nucleotide Variants Using Multiplex Droplet Digital PCR in Liquid Biopsies from Children with Medulloblastoma
Source: Cancers (Basel). 2023 Mar 25;15(7):1972. doi: 10.3390/cancers15071972 (PMC10092983; doi:10.3390/cancers15071972)
Supplement: Supplementary file 1 [file cancers-15-01972-s001.zip › File S2 QX Manager Software output data on liquid biopsies/Liquid biopsies MB35.pdf]

MB35 NTC

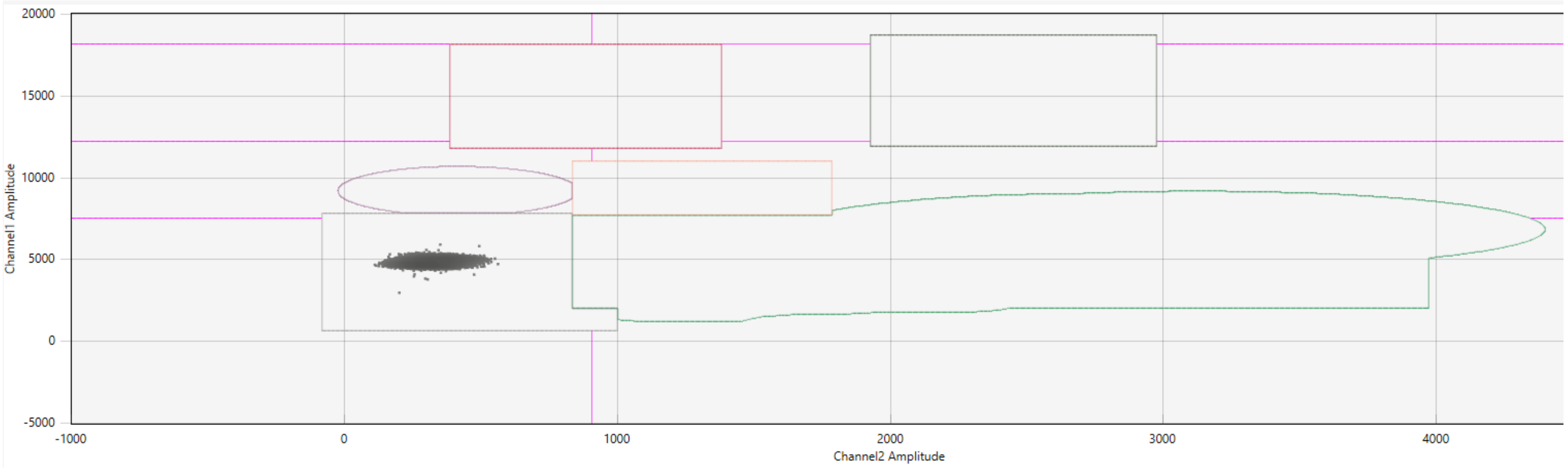

20000
15000
10000
5000
0
-5000
Channel1 Amplitude
-1000
0
1000
2000
3000
4000
Channel2 Amplitude

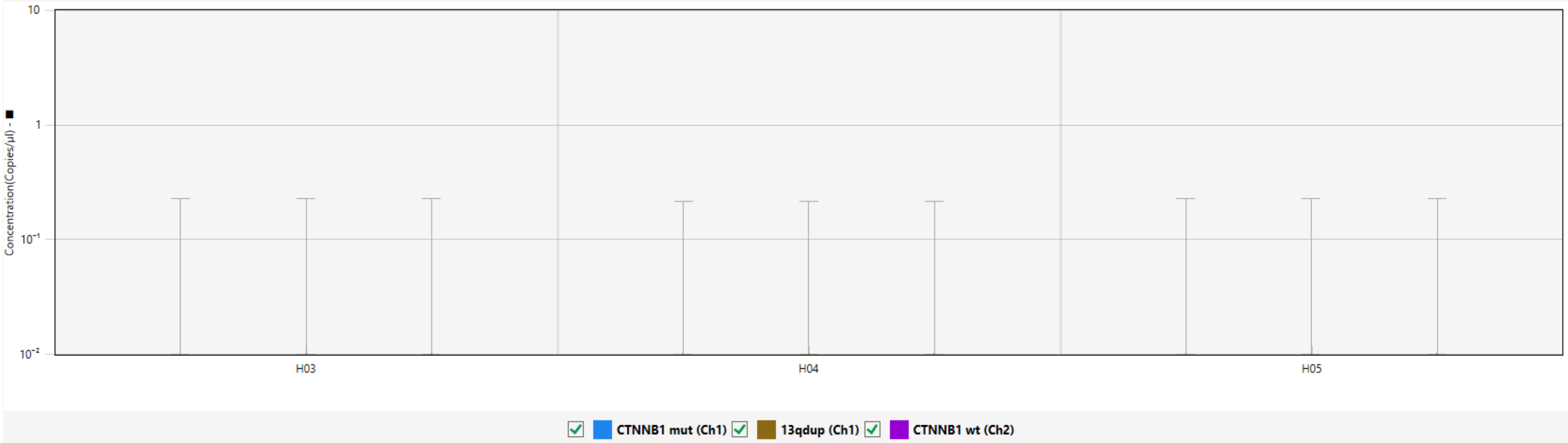

10
1
10^{-1}
10^{-2}
Concentration(Copies/µl) -
H03
H04
H05
CTNNB1 mut (Ch1)
13qdup (Ch1)
CTNNB1 wt (Ch2)

MB35 NC pl & gNC

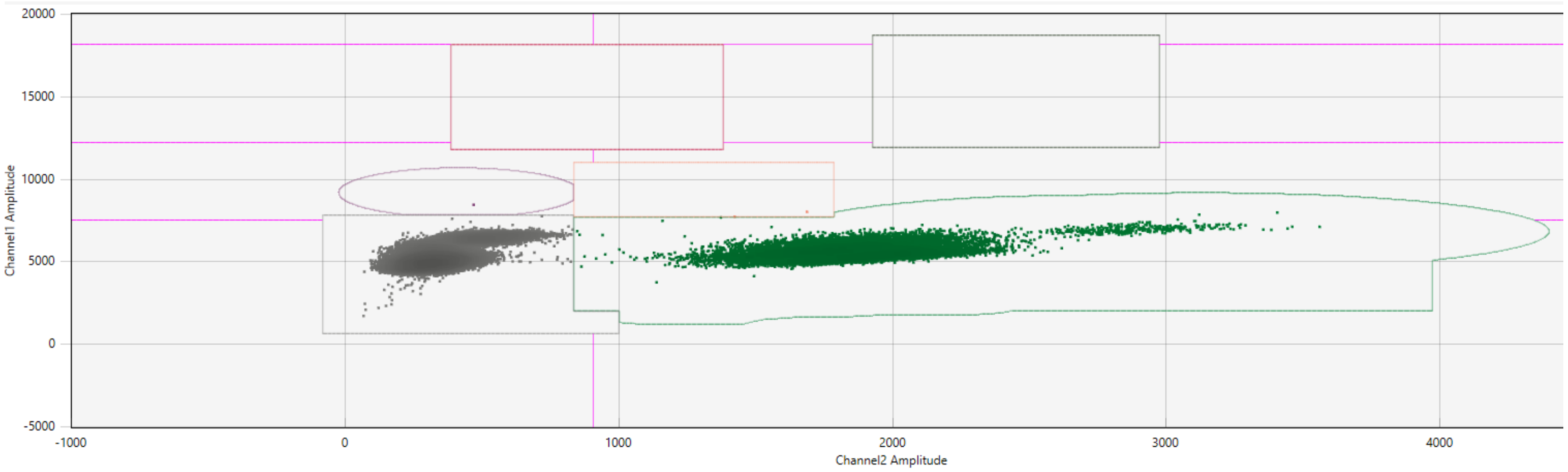

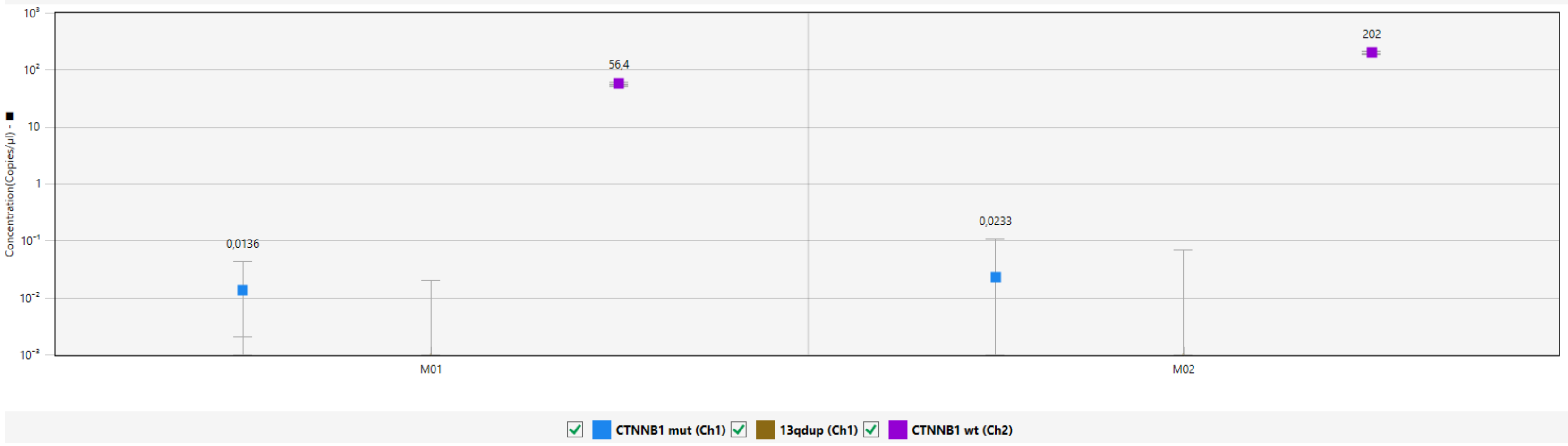

MB35 Cluster example (PC 1:10)

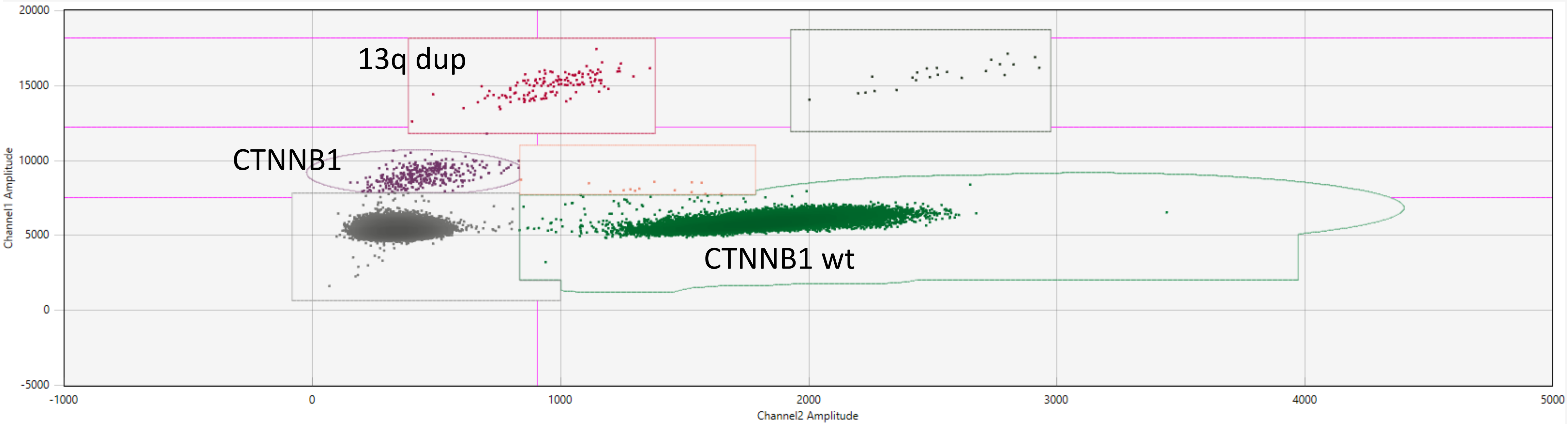

MB35 Dilution series: LoD & LoQ 1:100 both targets.

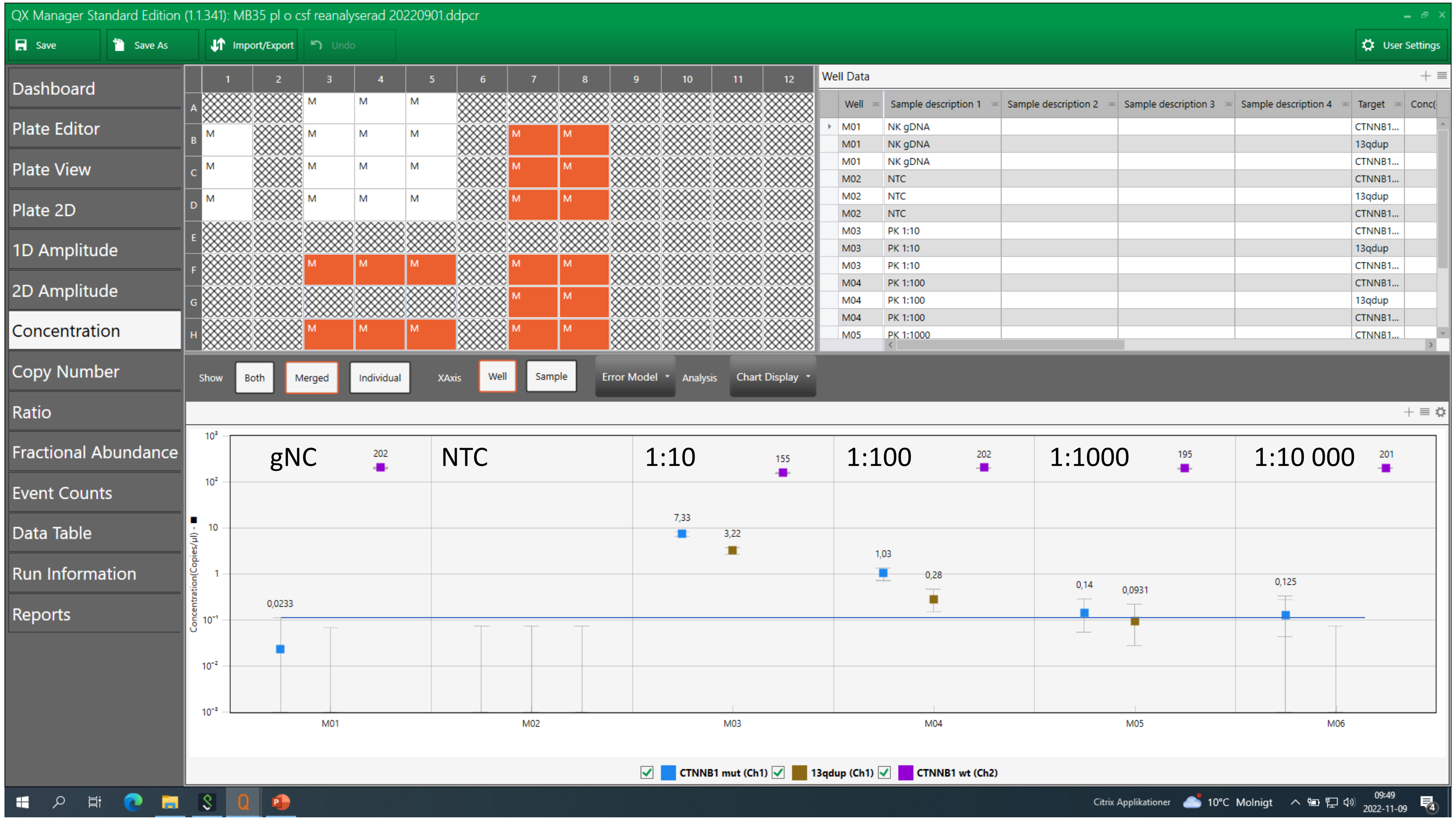

MB35 Pl d0

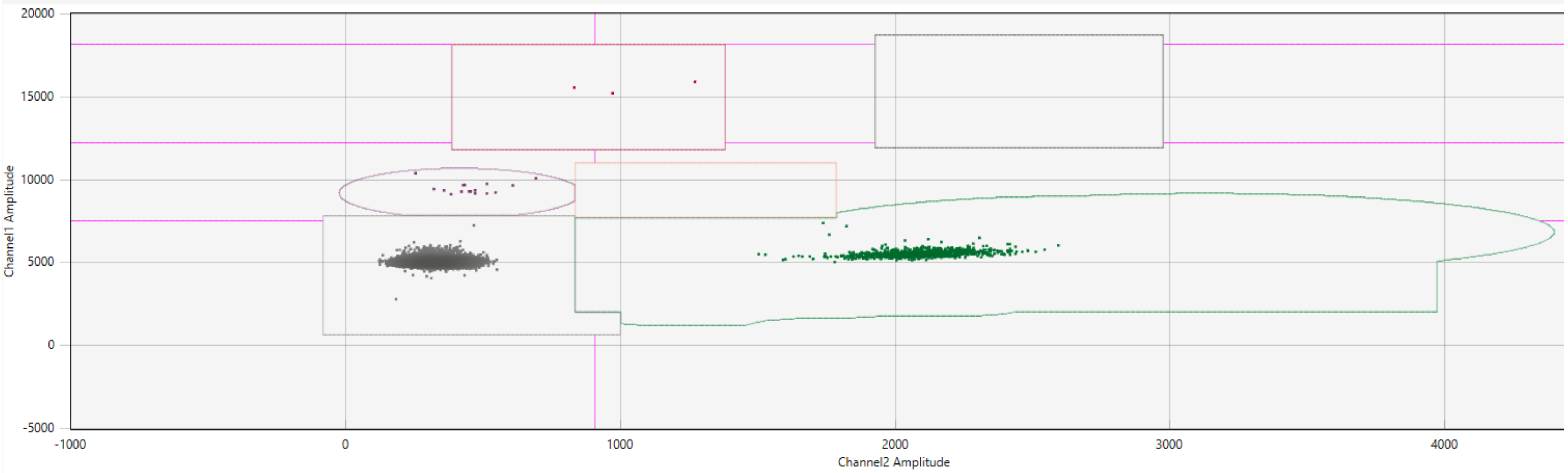

20000
15000
10000
5000
0
-5000
Channel1 Amplitude
-1000
0
1000
2000
3000
4000
Channel2 Amplitude

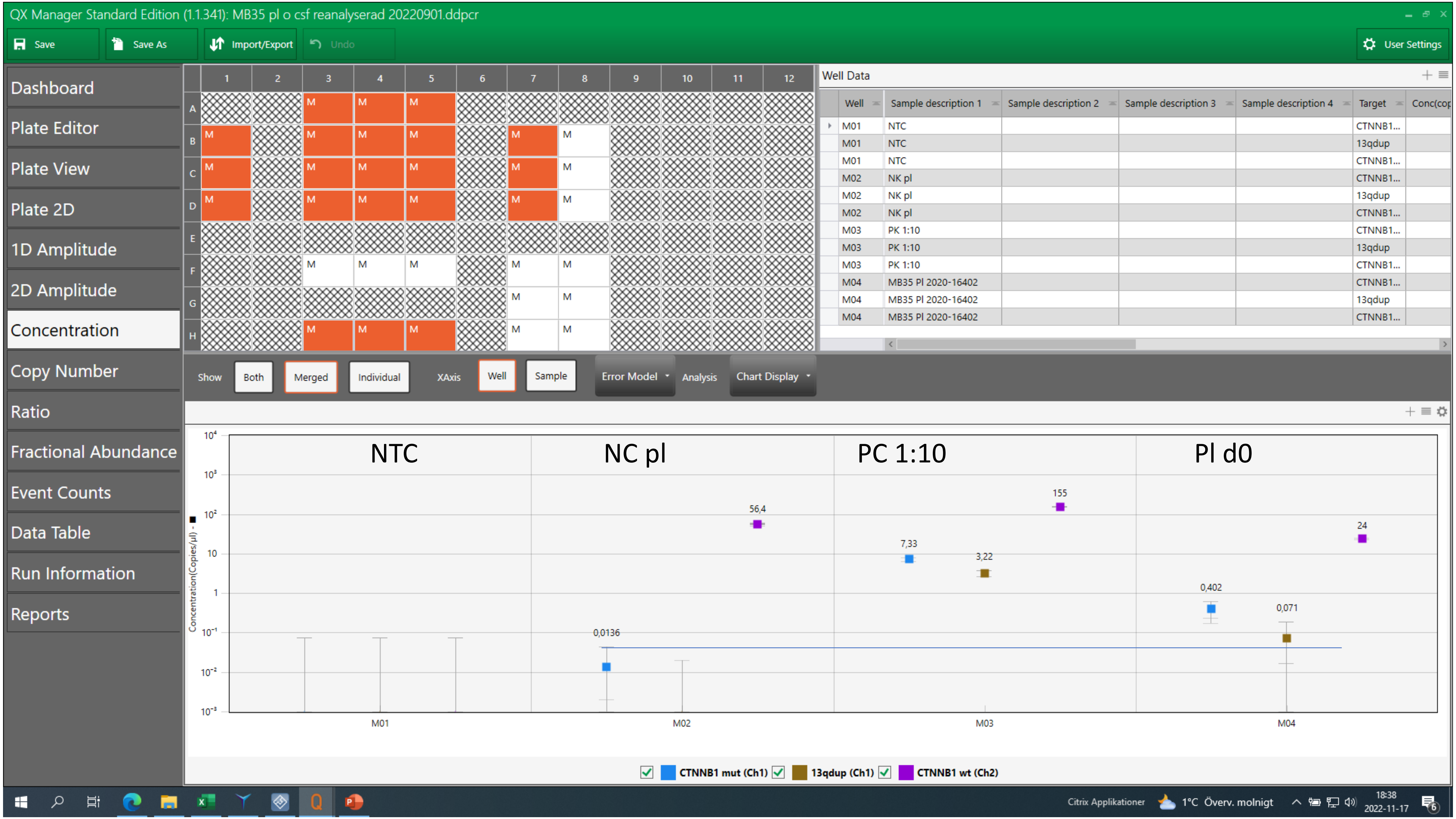
QX Manager Standard Edition (1.1.341): MB35 pl o csf reanalyserad 20220901.ddpcr
Save
Save As
Import/Export
Undo
User Settings
Dashboard
Plate Editor
Plate View
Plate 2D
1D Amplitude
2D Amplitude
Concentration
Copy Number
Ratio
Fractional Abundance
Event Counts
Data Table
Run Information
Reports
Well Data
Well
Sample description 1
Sample description 2
Sample description 3
Sample description 4
Target
M01 NTC CTNNB1...
M01 NTC 13qdup
M01 NTC CTNNB1...
M02 NK pl CTNNB1...
M02 NK pl 13qdup
M02 NK pl CTNNB1...
M03 PK 1:10 CTNNB1...
M03 PK 1:10 13qdup
M03 PK 1:10 CTNNB1...
M04 MB35 Pl 2020-16402 CTNNB1...
M04 MB35 Pl 2020-16402 13qdup
M04 MB35 Pl 2020-16402 CTNNB1...
Show
Both
Merged
Individual
XAxis
Well
Sample
Error Model
Analysis
Chart Display
NTC
NC pl
PC 1:10
Pl d0
Concentration(Copies/µl)
56,4
155
7,33
3,22
24
0,402
0,071
0,0136
M01
M02
M03
M04
CTNNB1 mut (Ch1)
13qdup (Ch1)
CTNNB1 wt (Ch2)
18:38
2022-11-17
