# Supplementary material for: Simultaneous Ultra-Sensitive Detection of Structural and Single Nucleotide Variants Using Multiplex Droplet Digital PCR in Liquid Biopsies from Children with Medulloblastoma
Source: Cancers (Basel). 2023 Mar 25;15(7):1972. doi: 10.3390/cancers15071972 (PMC10092983; doi:10.3390/cancers15071972)
Supplement: Supplementary file 1 [file cancers-15-01972-s001.zip › File S2 QX Manager Software output data on liquid biopsies/Liquid biopsies MB43.pdf]

MB43 NTC

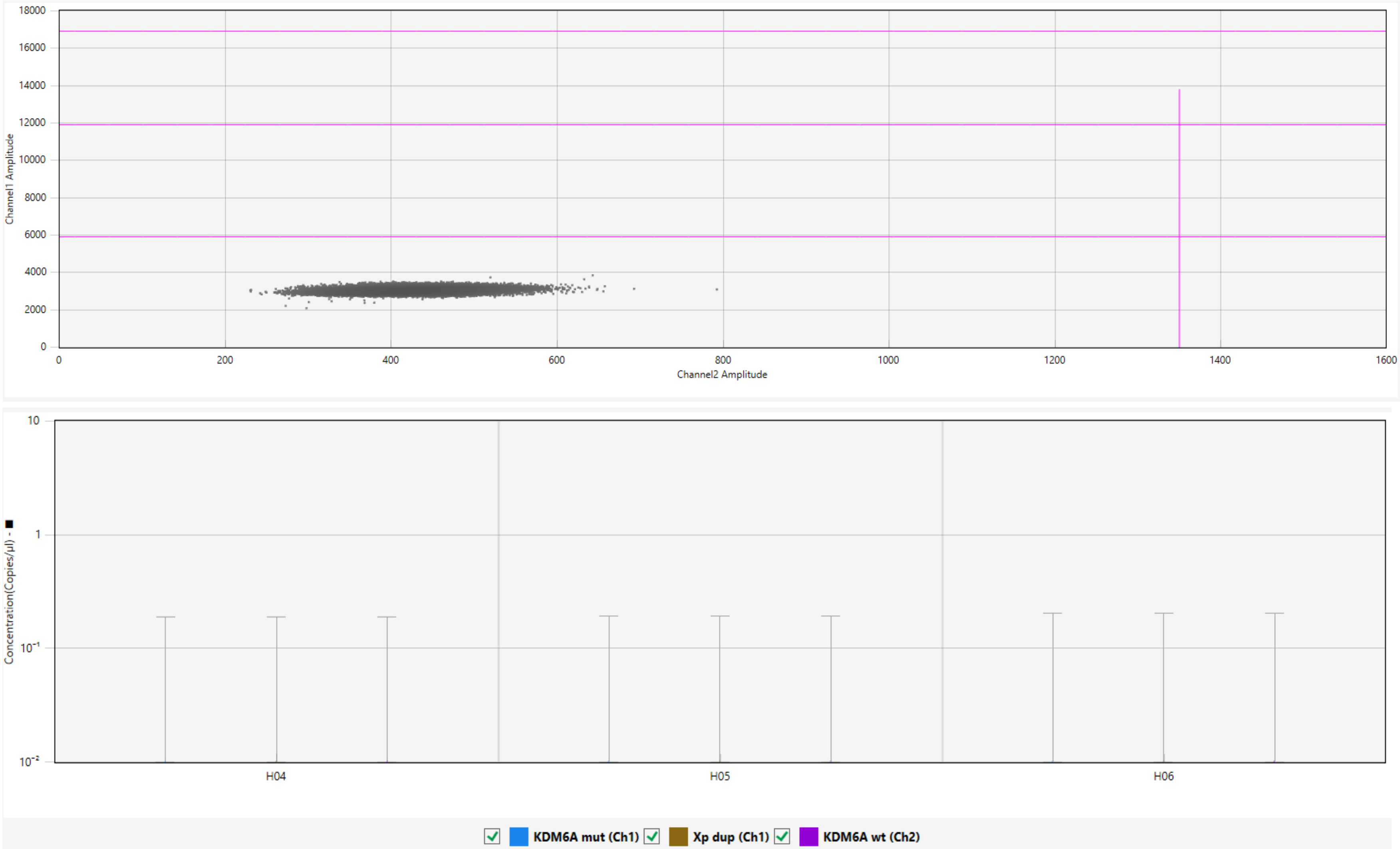

Channel1 Amplitude
Channel2 Amplitude
18000
16000
14000
12000
10000
8000
6000
4000
2000
0
200
400
600
800
1000
1200
1400
1600
Concentration(Copies/µl) -
10
1
10^{-1}
10^{-2}
H04
H05
H06
KDM6A mut (Ch1)
Xp dup (Ch1)
KDM6A wt (Ch2)

MB43 NC pl och gNC

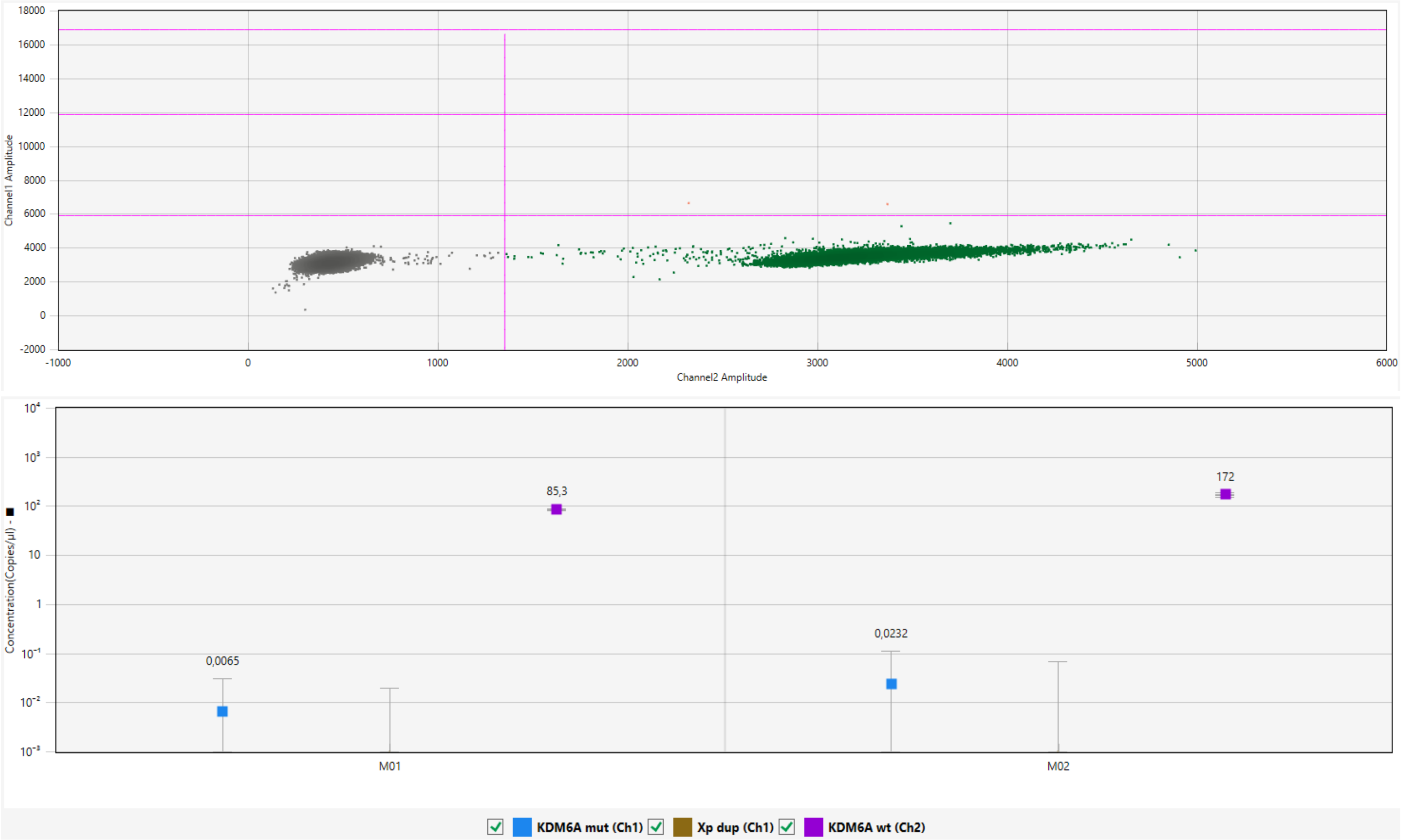

MB43 Example of clusters (PC 1:10)

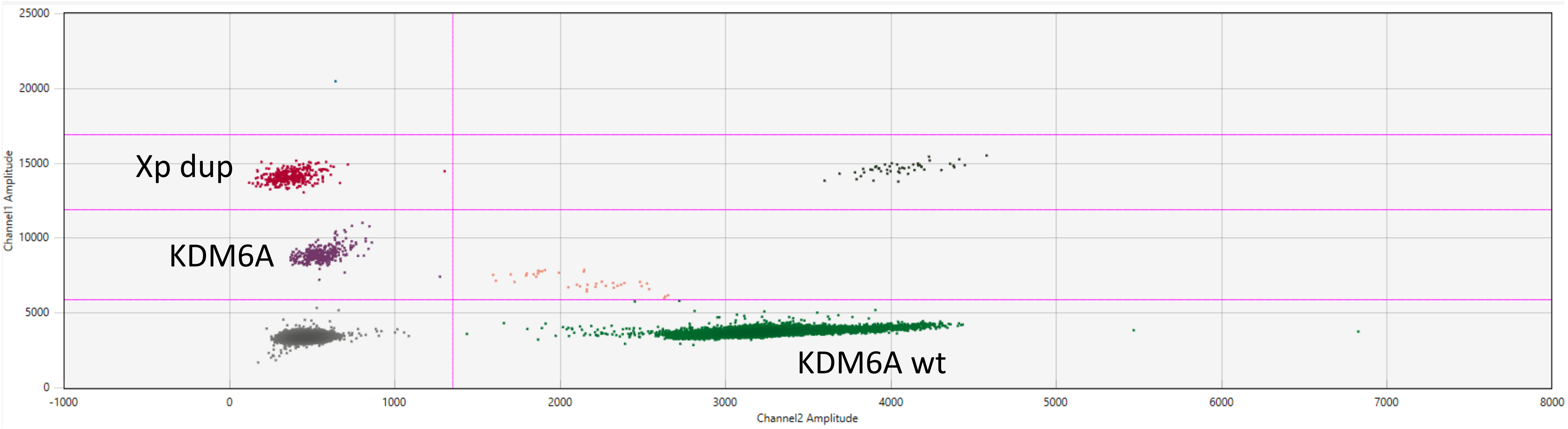

MB43 Dilution series: LoD & LoQ 1:100 both targets.

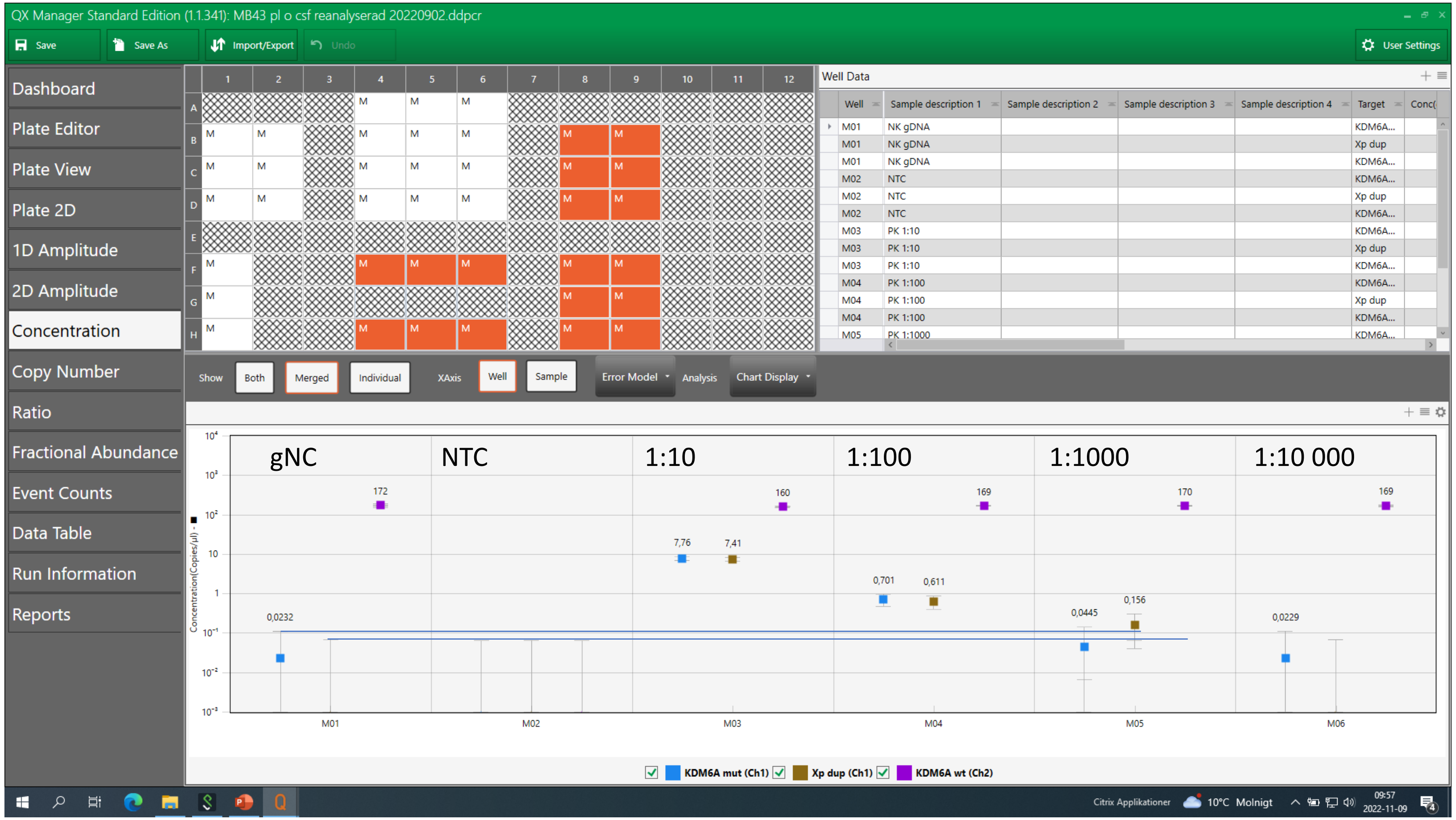

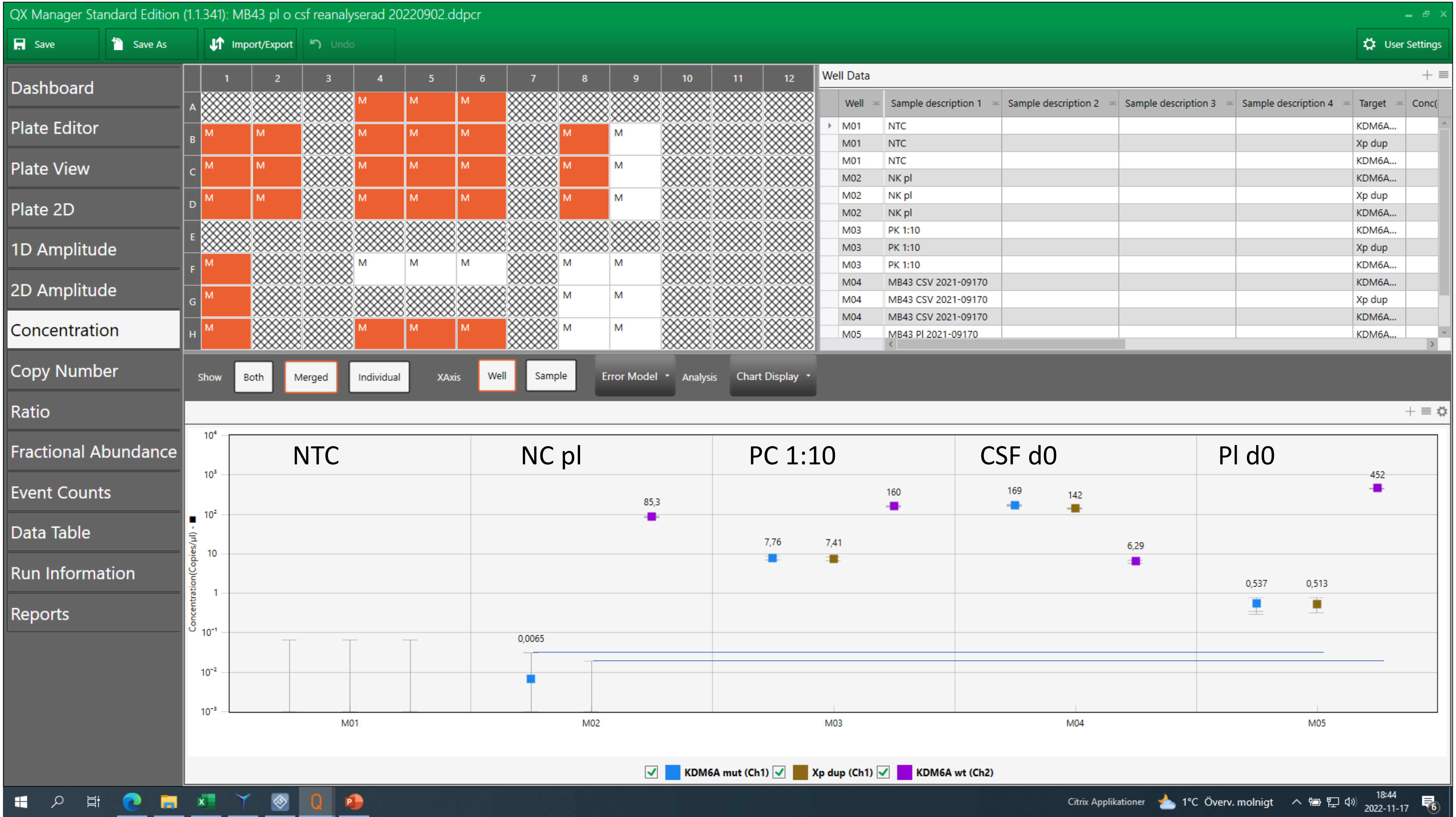

QX Manager Standard Edition (1.1.341): MB43 pl o csf reanalyserad 20220902.ddpcr
Save
Save As
Import/Export
Undo
User Settings
Dashboard
Plate Editor
Plate View
Plate 2D
1D Amplitude
2D Amplitude
Concentration
Copy Number
Ratio
Fractional Abundance
Event Counts
Data Table
Run Information
Reports
Well Data
Well | Sample description 1 | Sample description 2 | Sample description 3 | Sample description 4 | Target | Conc(
M01 NTC KDM6A...
M01 NTC Xp dup
M01 NTC KDM6A...
M02 NK pl KDM6A...
M02 NK pl Xp dup
M02 NK pl KDM6A...
M03 PK 1:10 KDM6A...
M03 PK 1:10 Xp dup
M03 PK 1:10 KDM6A...
M04 MB43 CSV 2021-09170 KDM6A...
M04 MB43 CSV 2021-09170 Xp dup
M04 MB43 CSV 2021-09170 KDM6A...
M05 MB43 Pl 2021-09170 KDM6A...
Show
Both
Merged
Individual
XAxis
Well
Sample
Error Model
Analysis
Chart Display
NTC
NC pl
PC 1:10
CSF d0
Pl d0
Concentration(Copies/µl)
85,3
0,0065
7,76
7,41
160
169
142
6,29
452
0,537
0,513
M01
M02
M03
M04
M05
KDM6A mut (Ch1)
Xp dup (Ch1)
KDM6A wt (Ch2)
Citrix Applikationer
1°C Överv. molnigt
18:44
2022-11-17

MB43 CSF d0

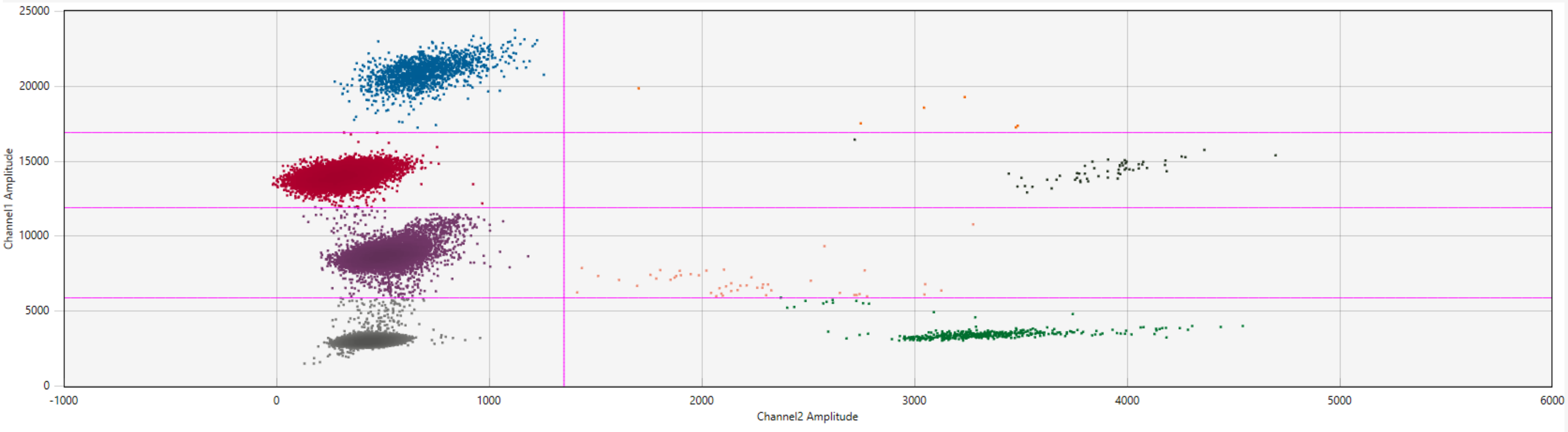

25000
20000
15000
10000
5000
0
Channel1 Amplitude
-1000
0
1000
2000
3000
4000
5000
6000
Channel2 Amplitude

MB43 Pl d0

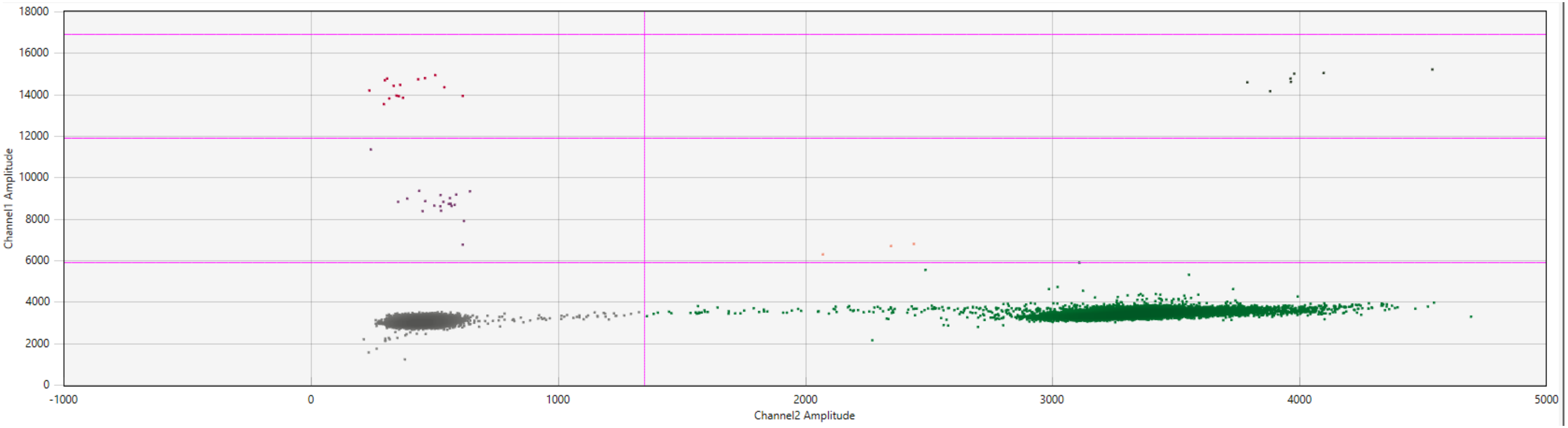

18000
16000
14000
12000
10000
8000
6000
4000
2000
0
Channel1 Amplitude
-1000
0
1000
2000
3000
4000
5000
Channel2 Amplitude
