# Supplementary figures and images for: Simultaneous Ultra-Sensitive Detection of Structural and Single Nucleotide Variants Using Multiplex Droplet Digital PCR in Liquid Biopsies from Children with Medulloblastoma
Source: Cancers (Basel). 2023 Mar 25;15(7):1972. doi: 10.3390/cancers15071972 (PMC10092983; doi:10.3390/cancers15071972)

# MB01 Concentration plot

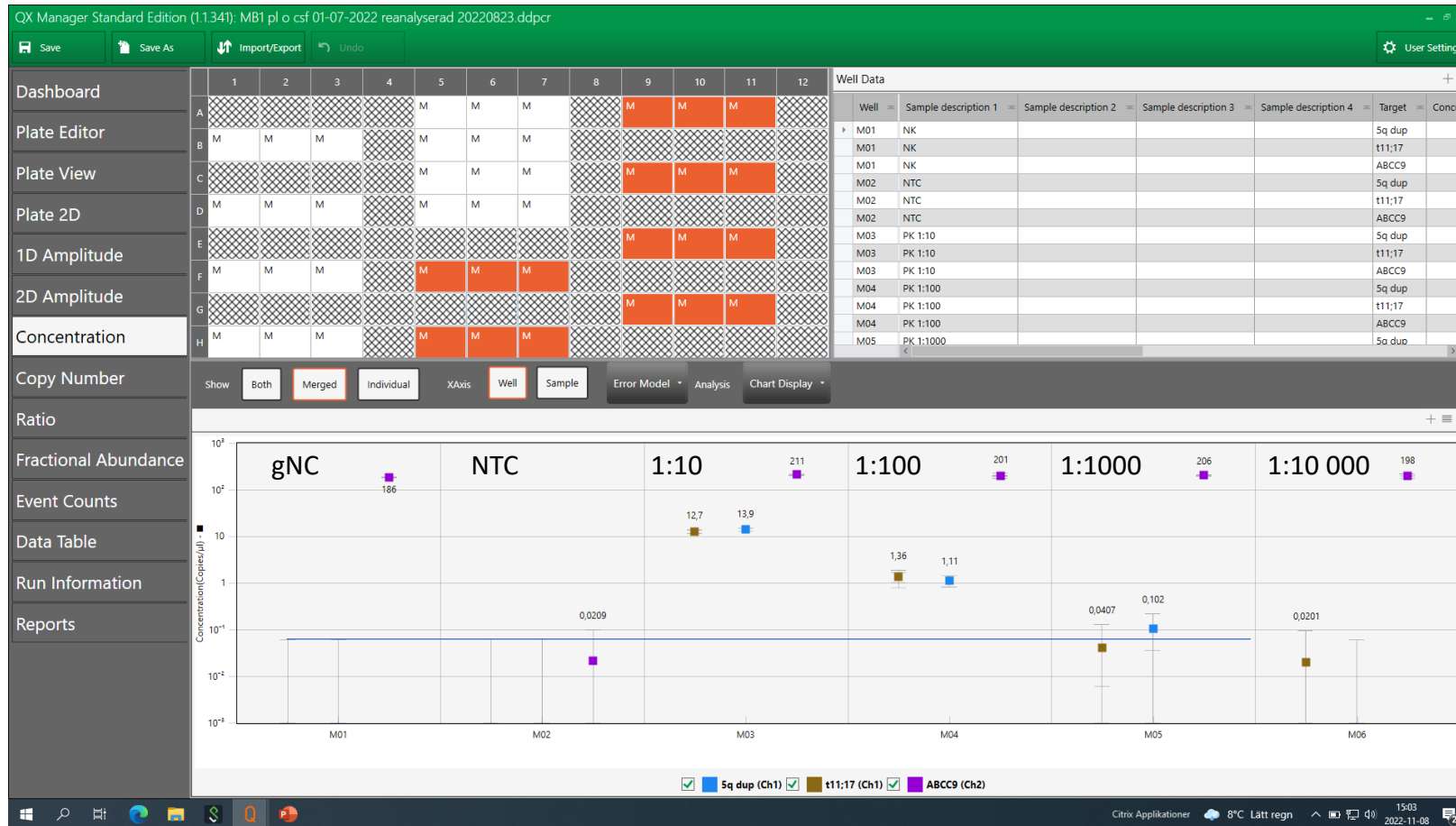

# MB01 NTC

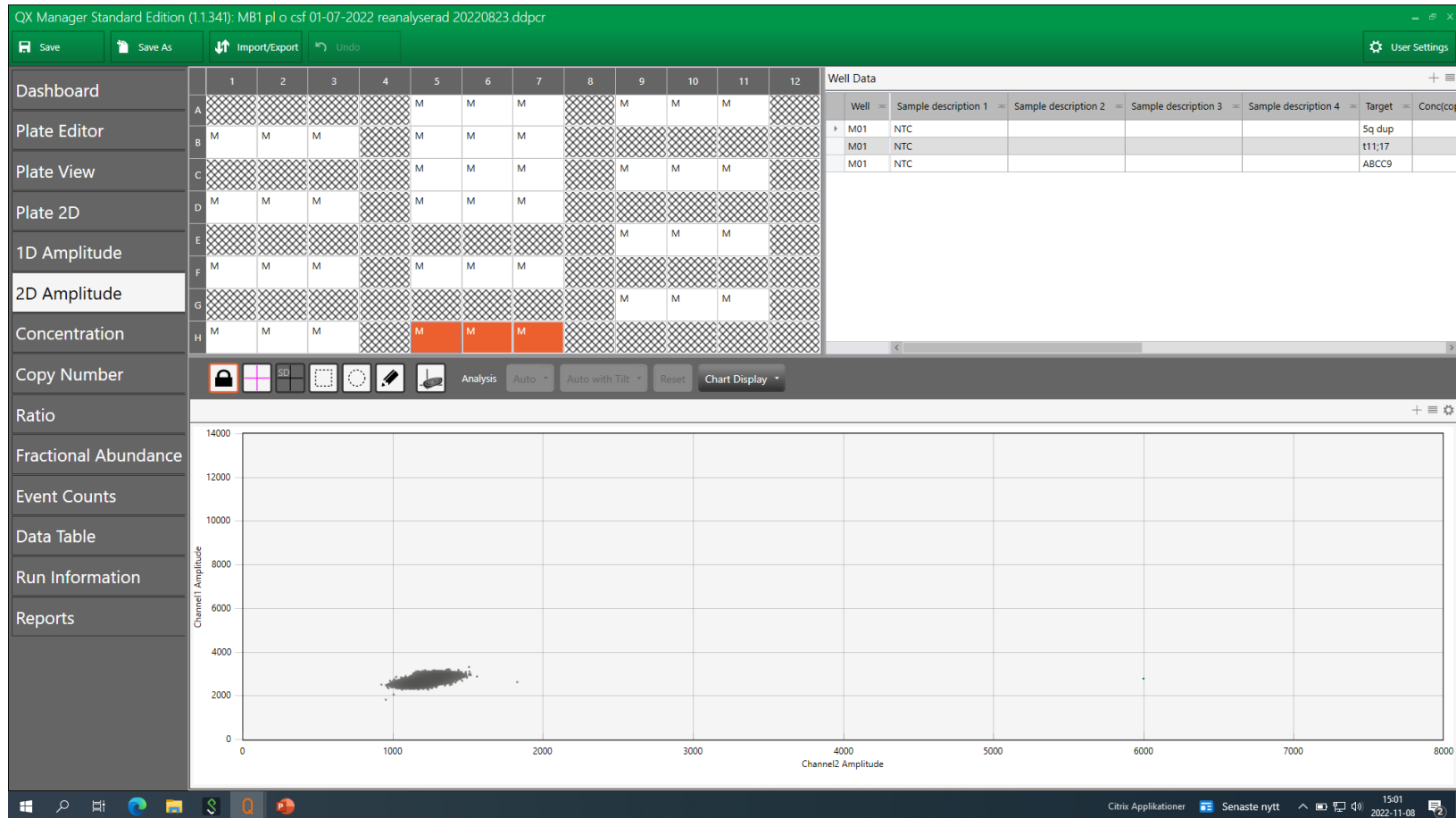

# MB01 gNC

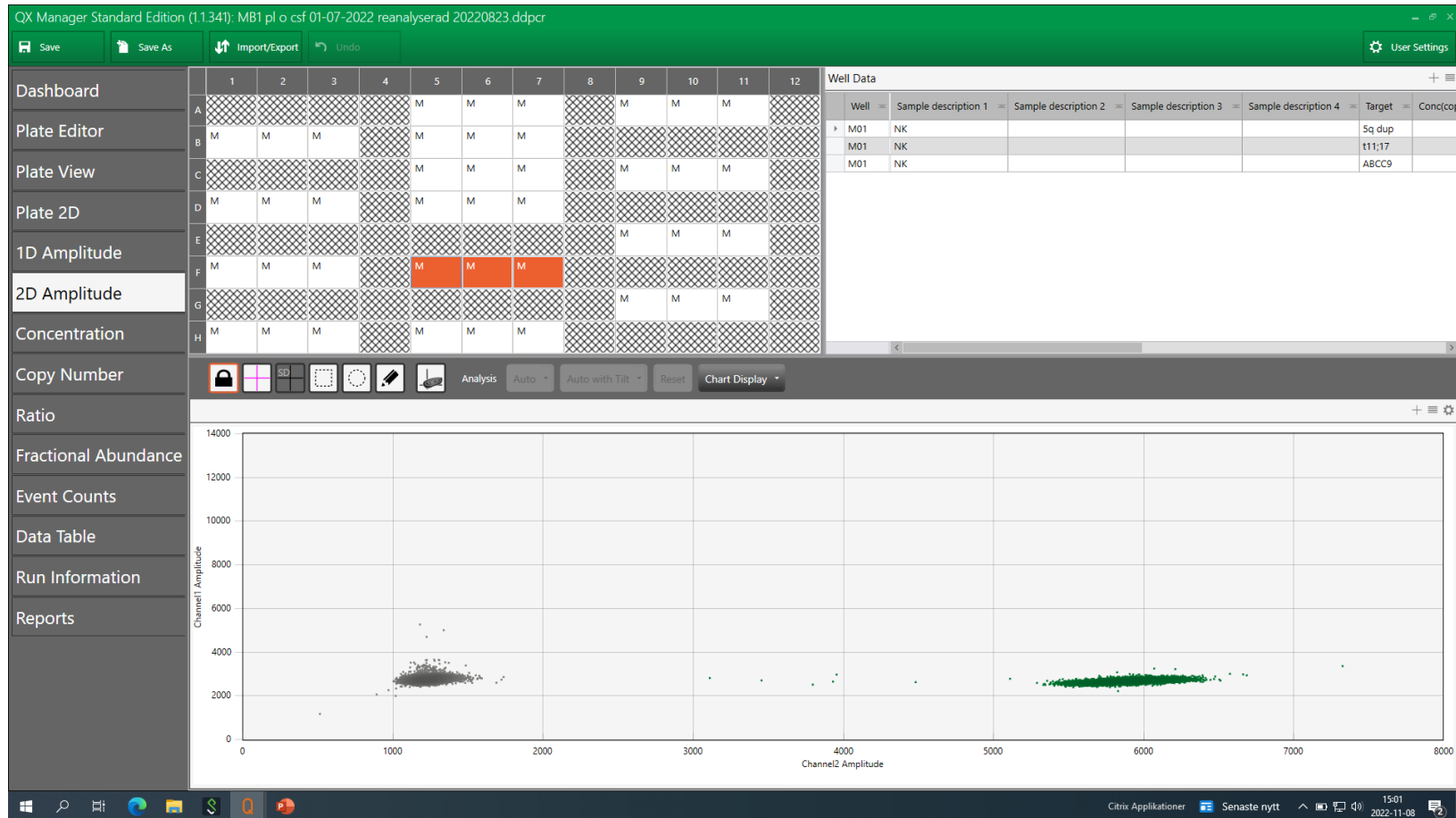

# MB01 1:10

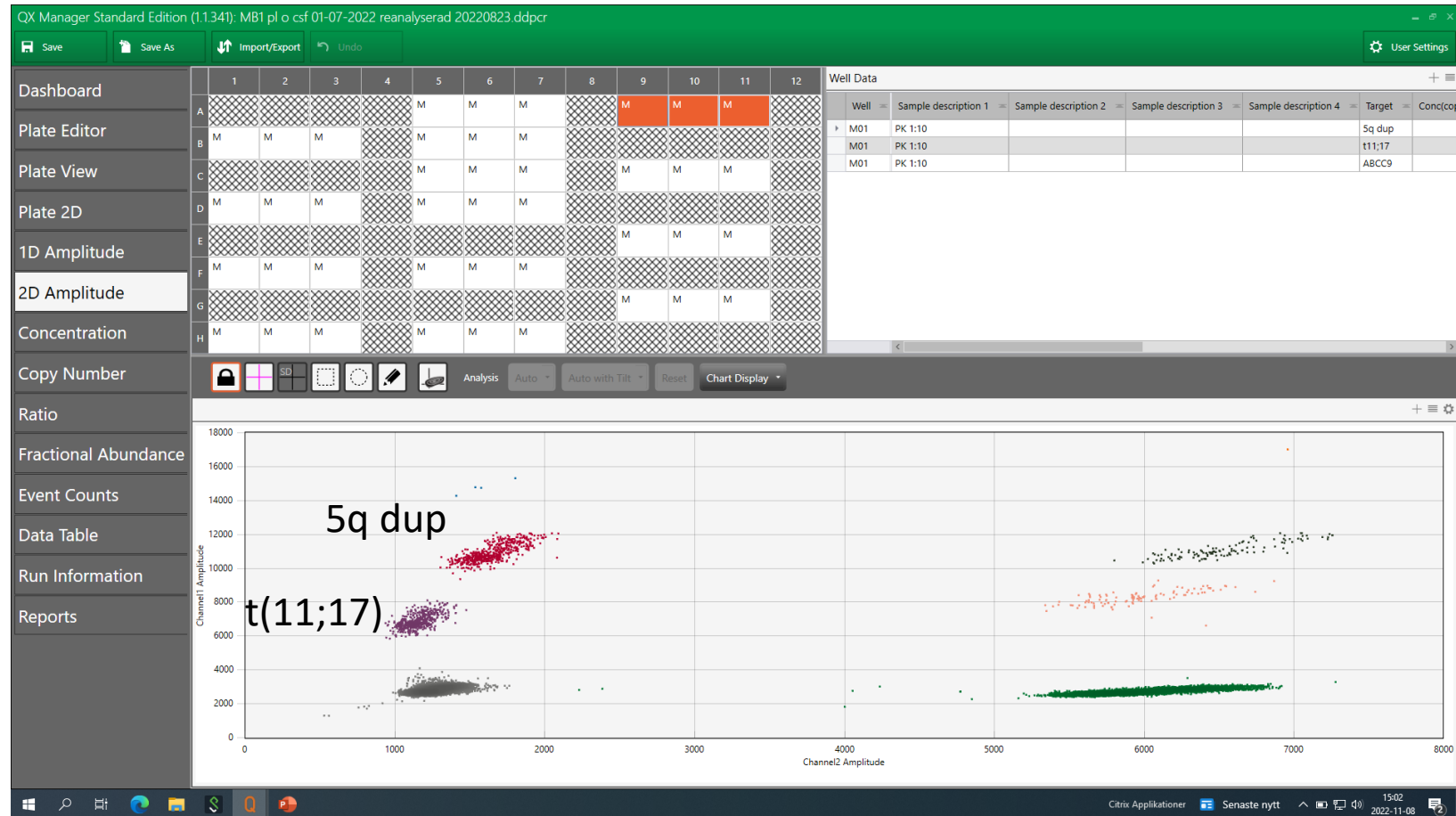

# MB01 1:100

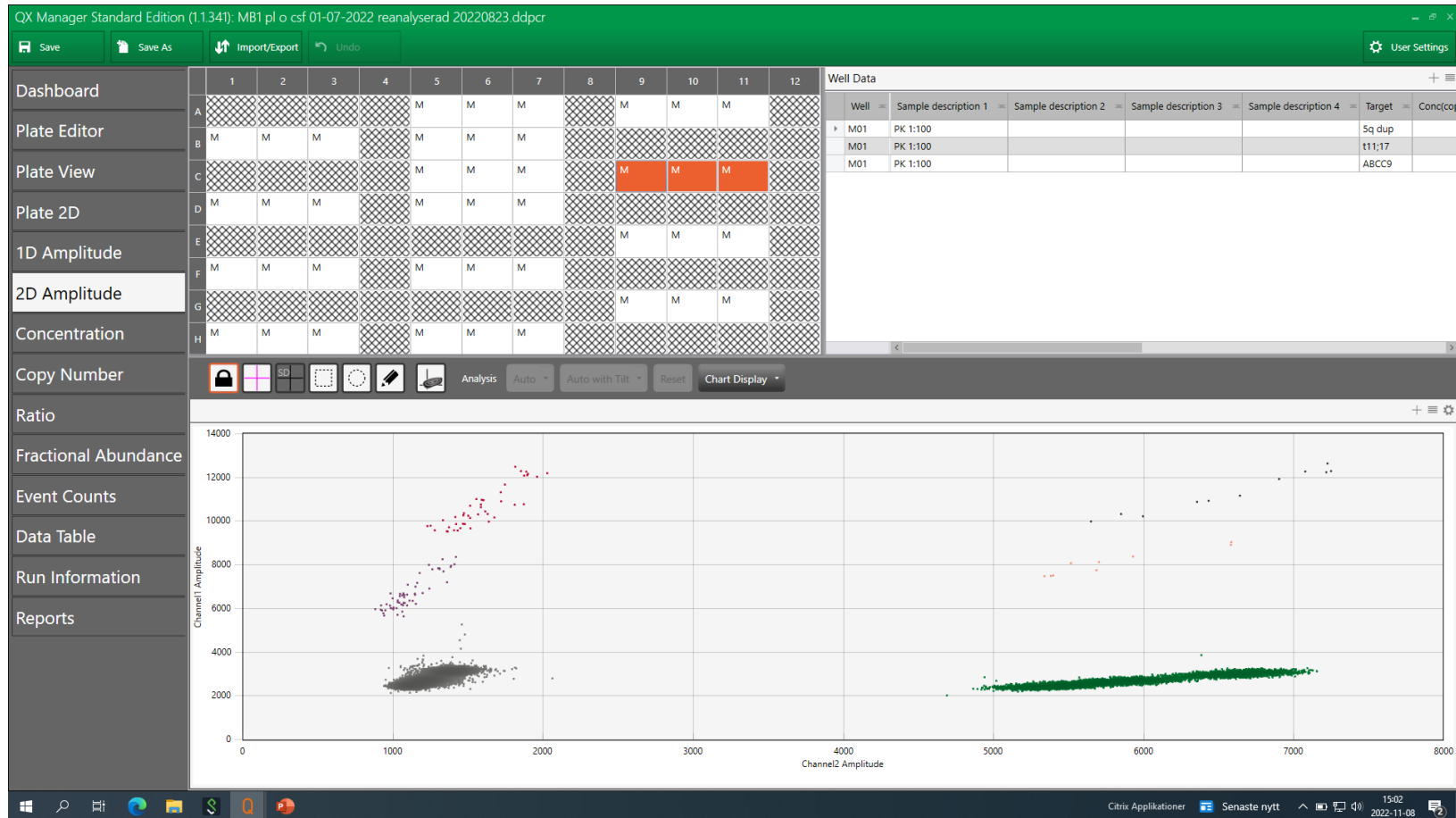

# MB01 1:1000

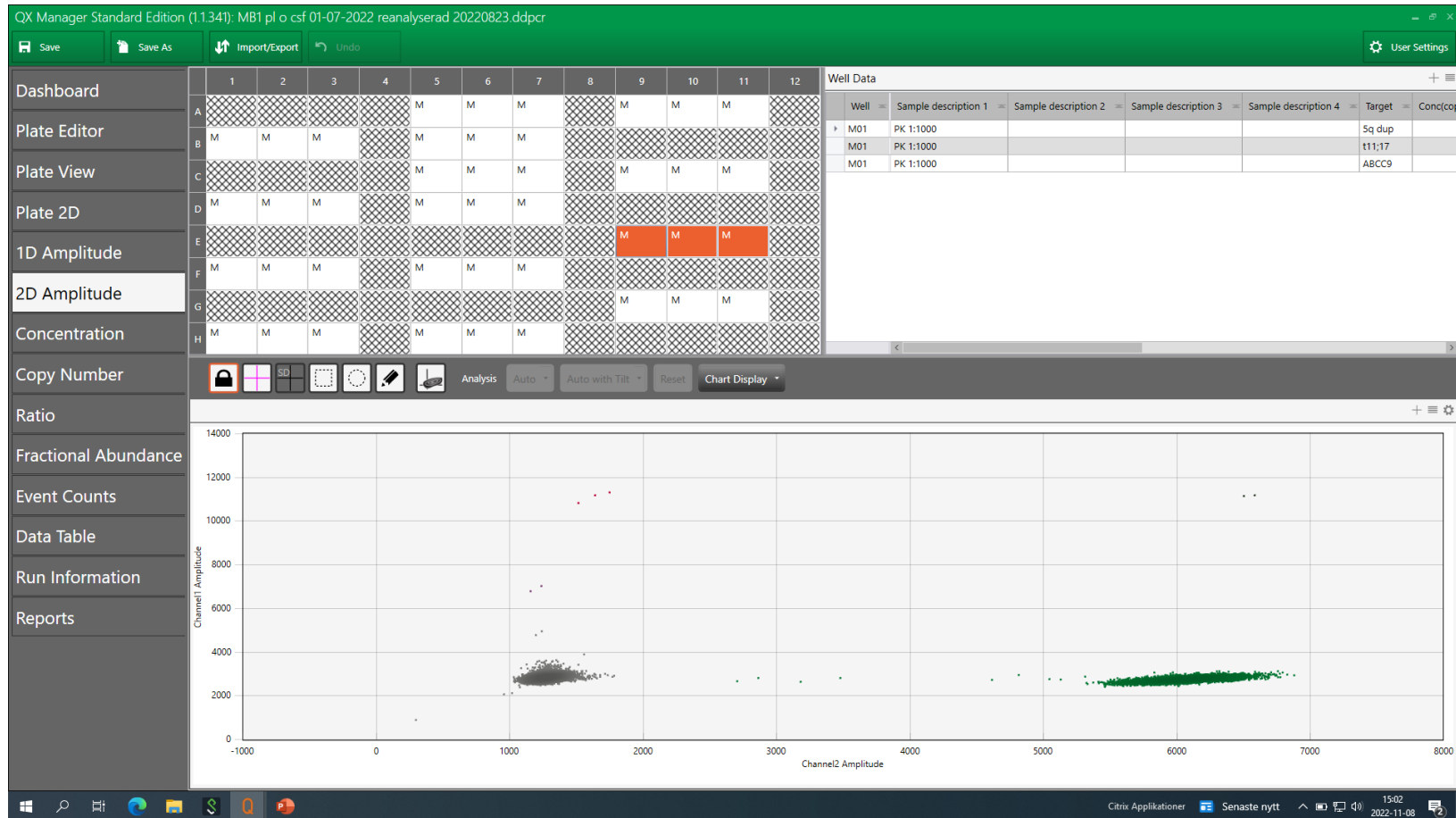

# MB01 1:10000

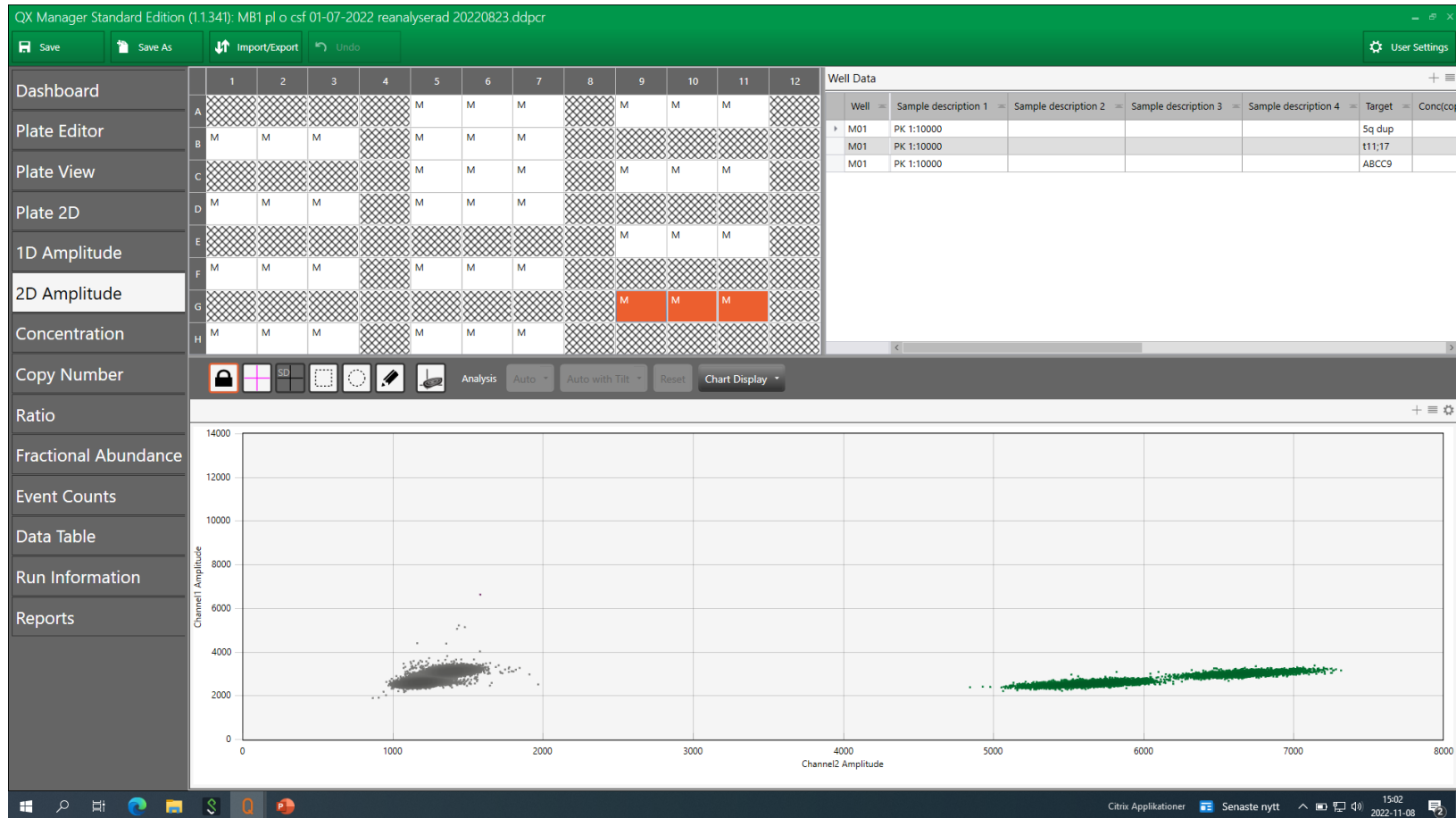

Supplement: Supplementary file 1 [file cancers-15-01972-s001.zip › File S1 QX Manager Software output data on dilution series/Dilution series MB01.pdf]

# MB09 Concentration plot

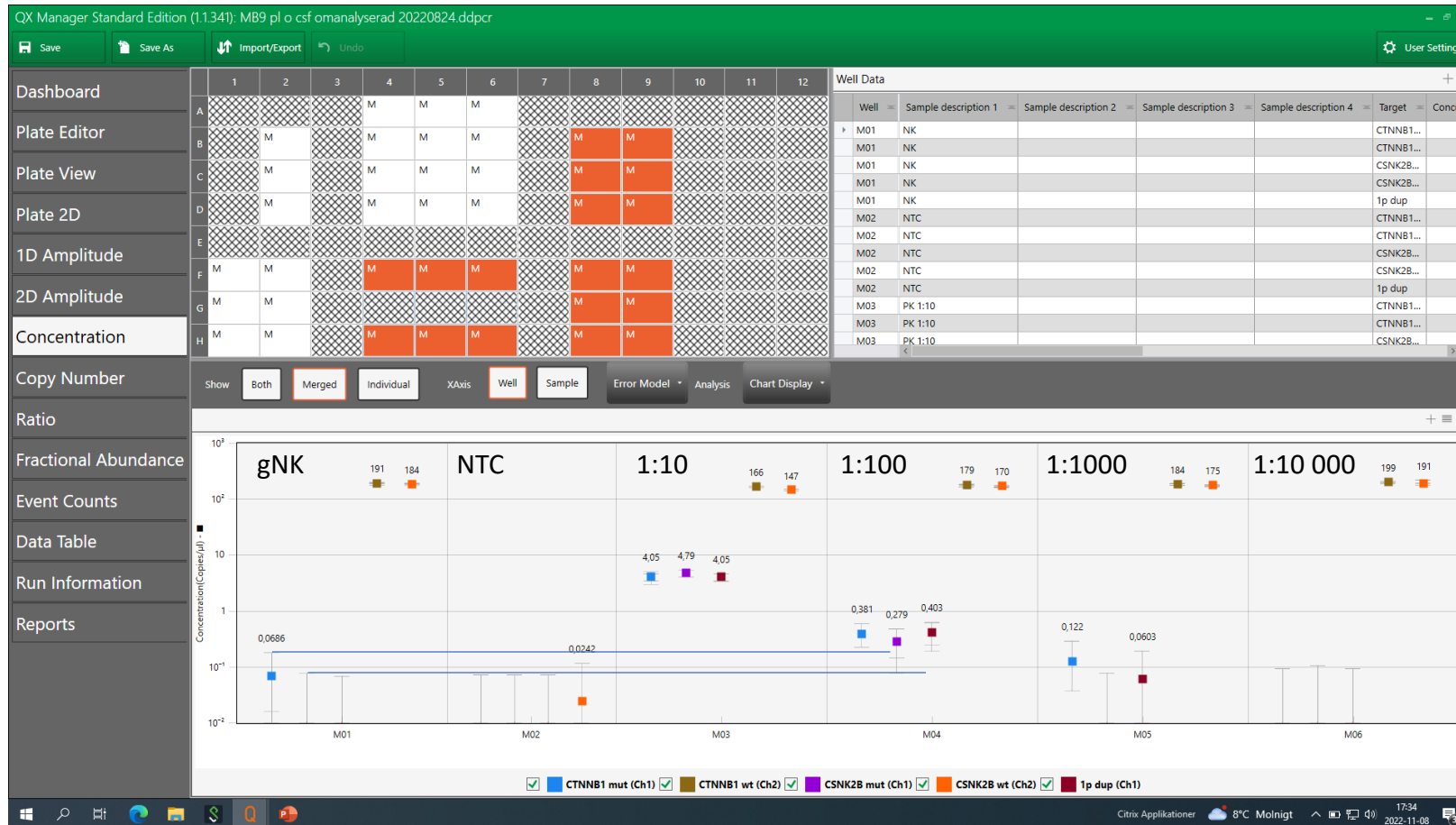

# MB09 NTC

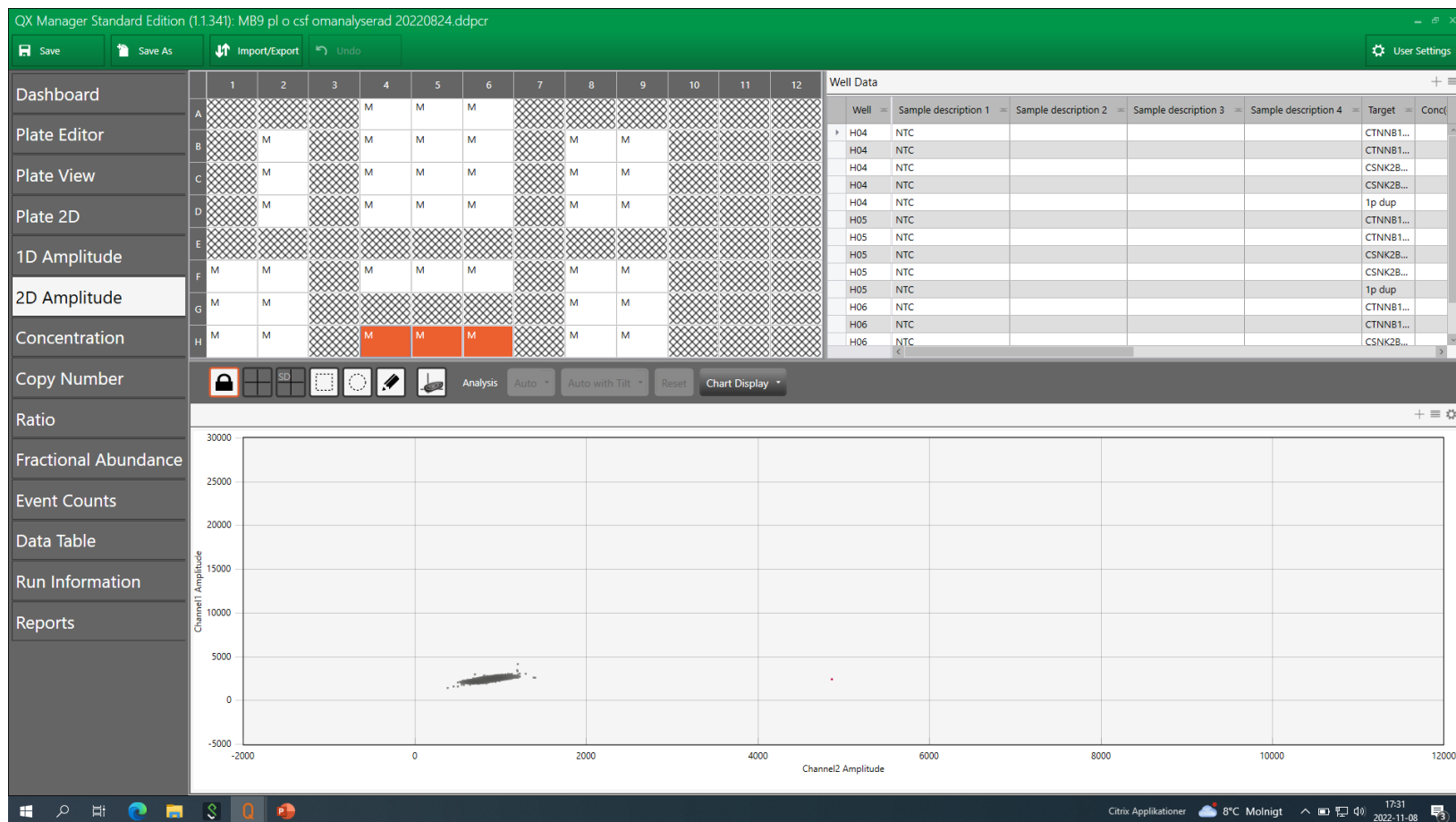

# MB09 gNC

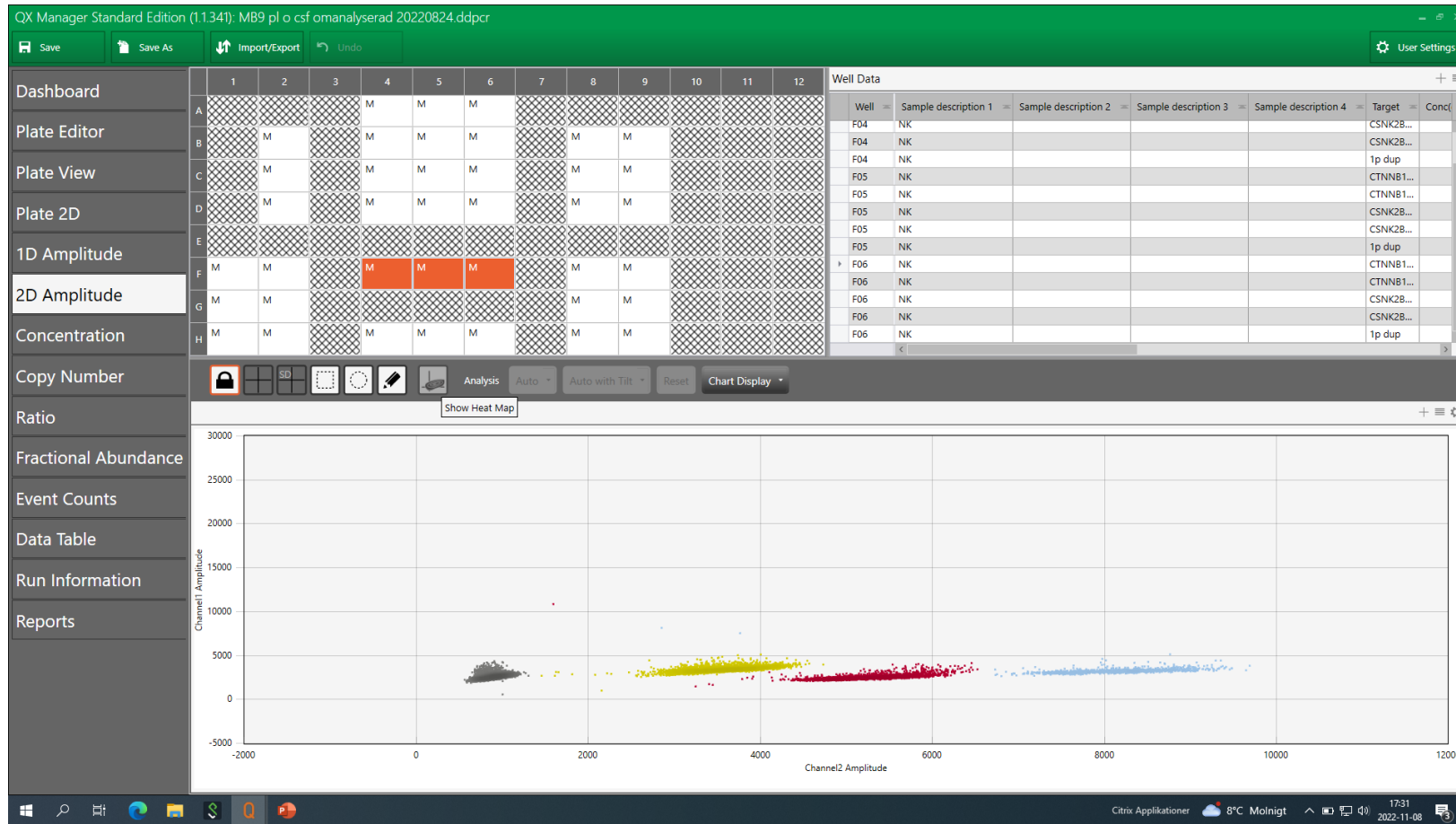

# MB09 Example of clusters

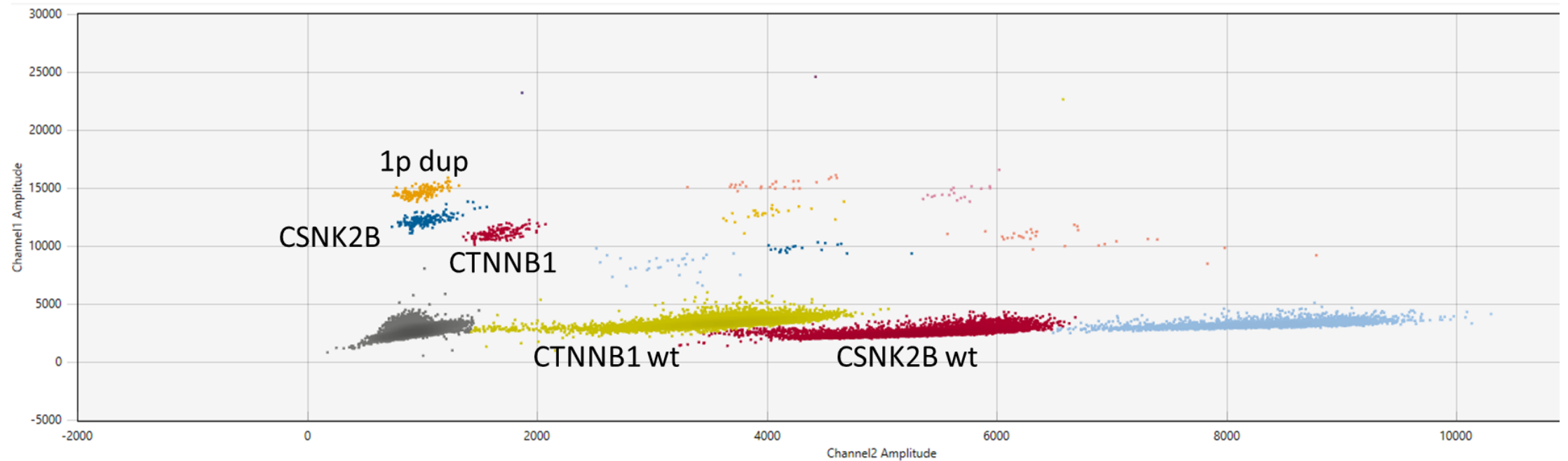

# MB09 1:10

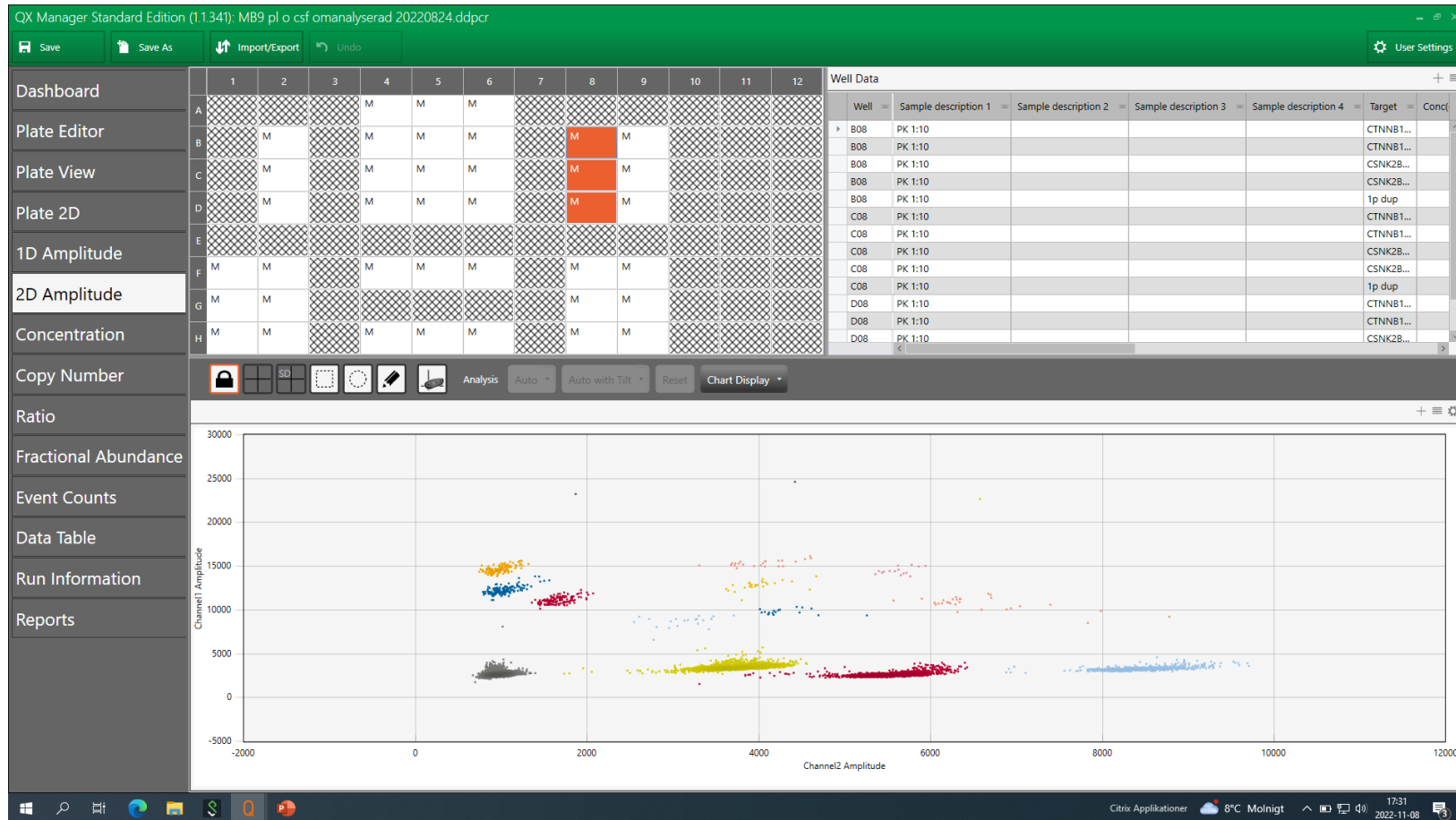

# MB09 1:100

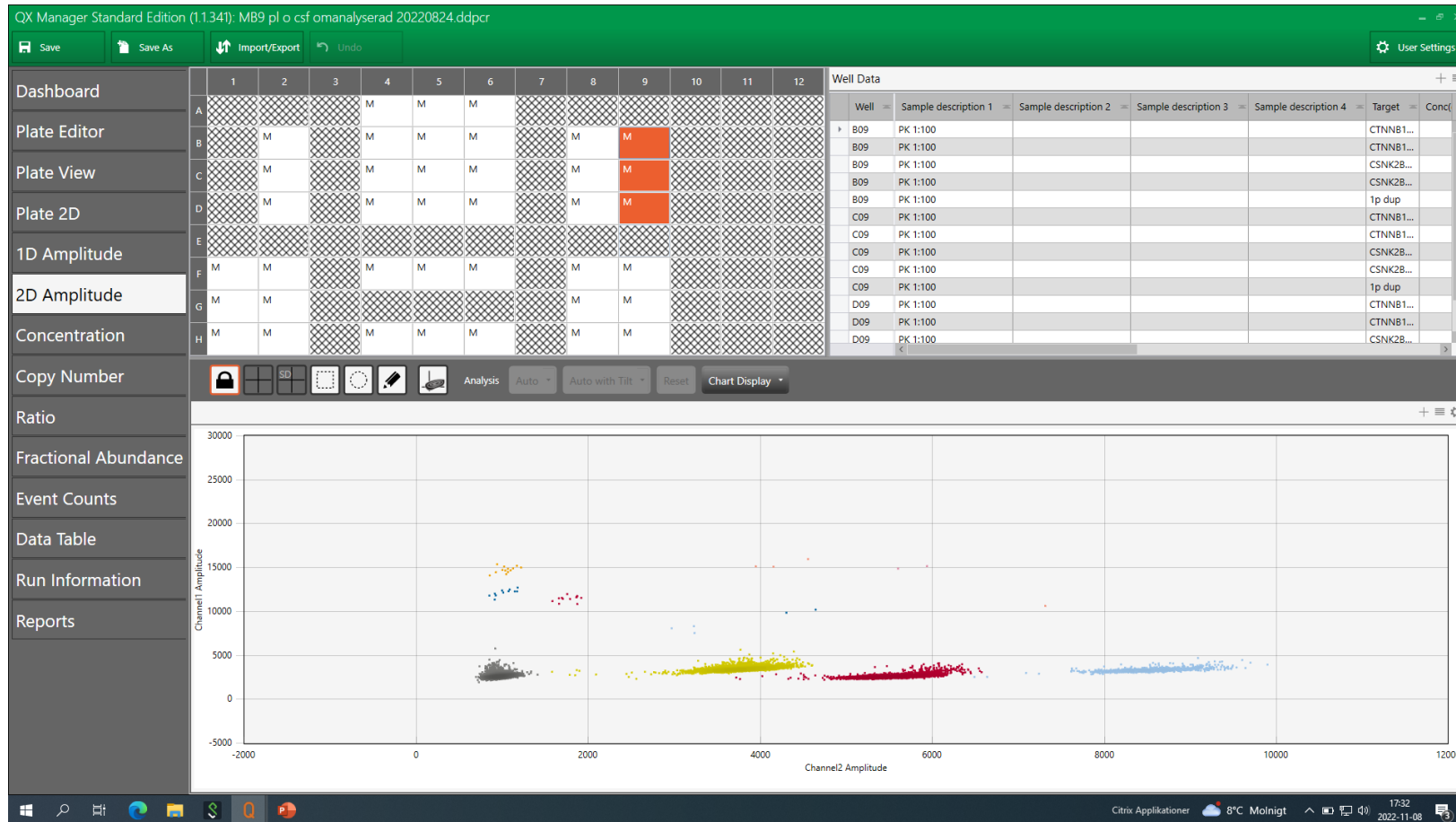

# MB09 1:1000

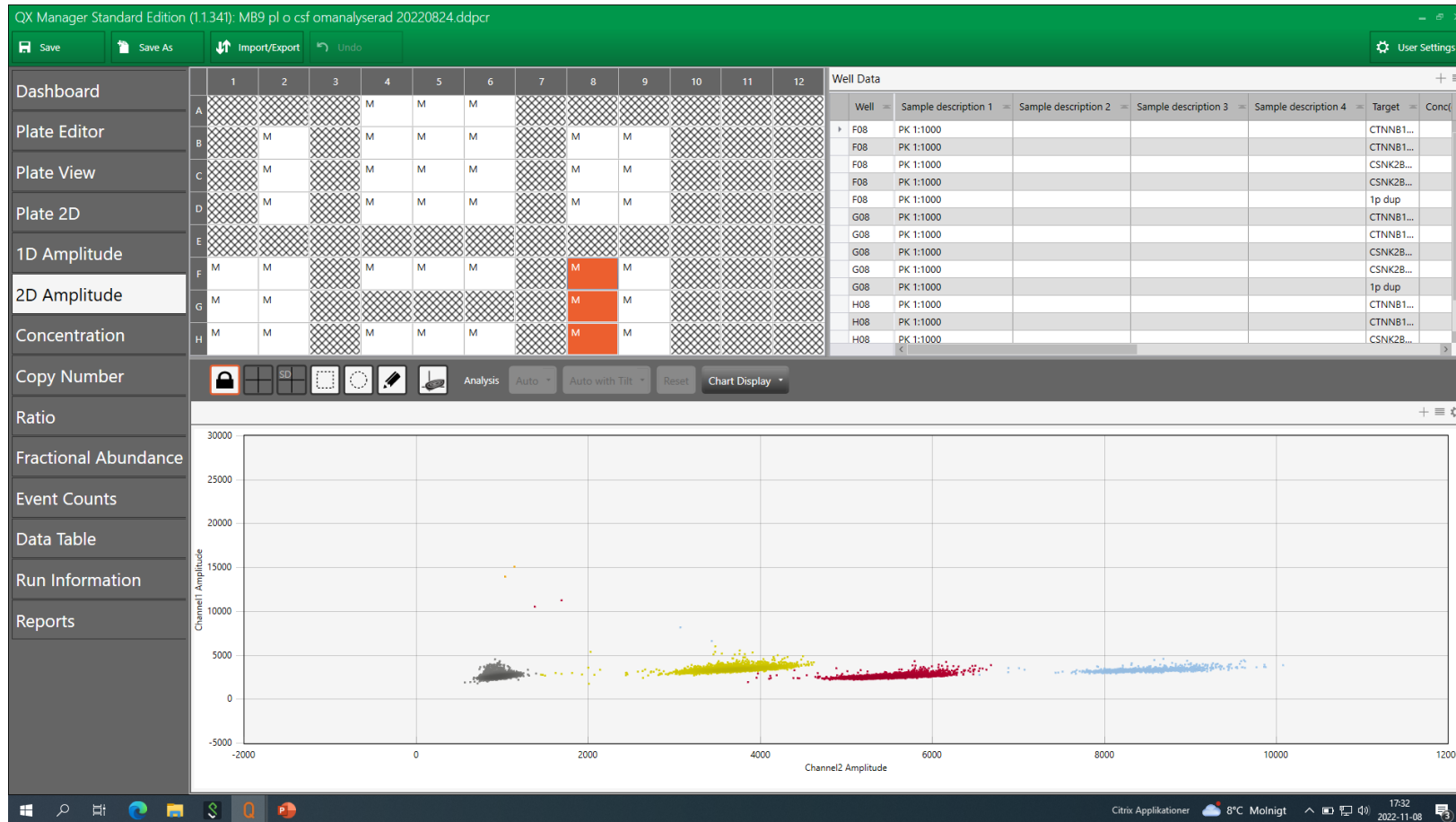

# MB09 1:10000

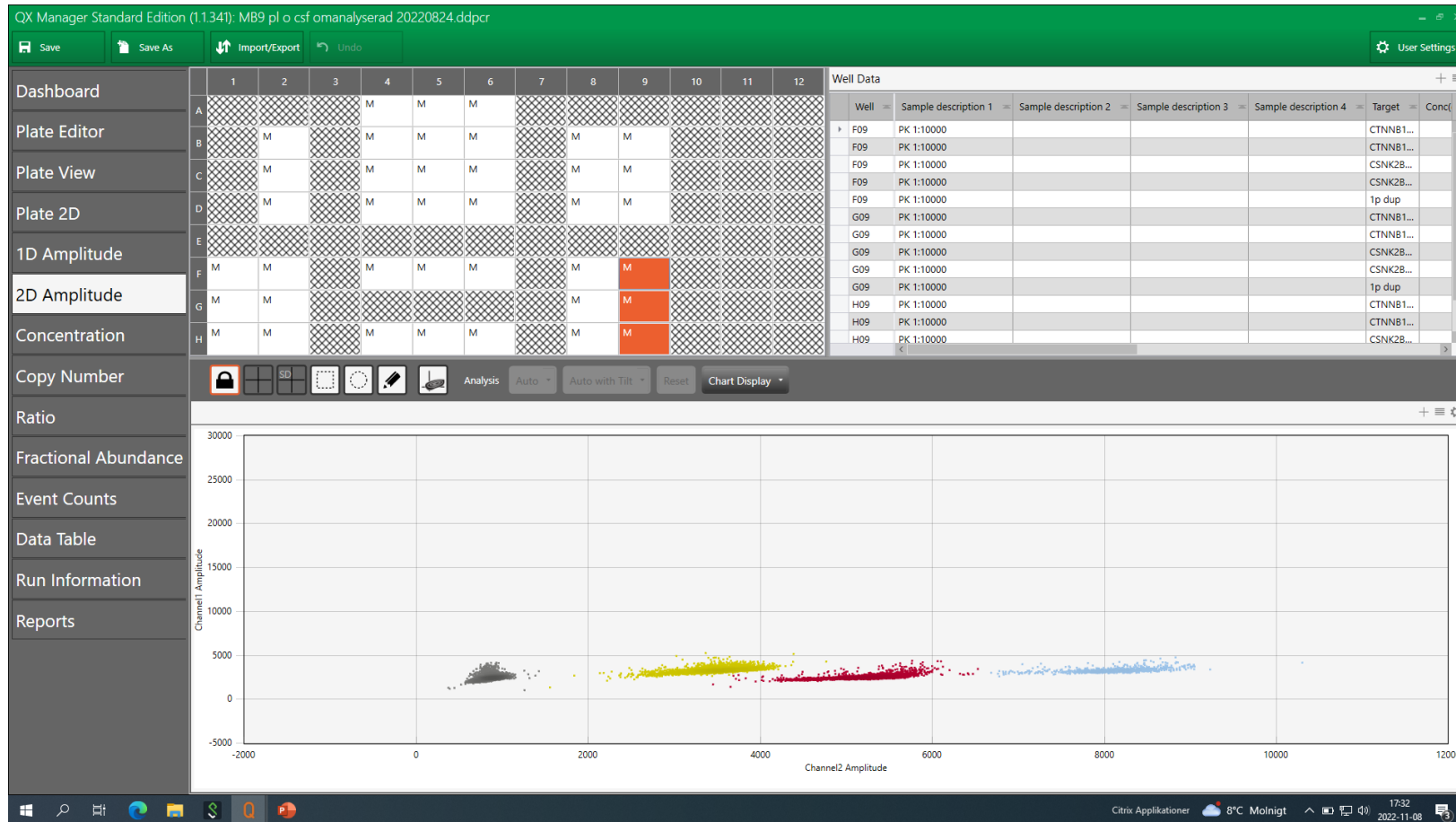

Supplement: Supplementary file 1 [file cancers-15-01972-s001.zip › File S1 QX Manager Software output data on dilution series/Dilution series MB09.pdf]

# MB13 Concentration plot

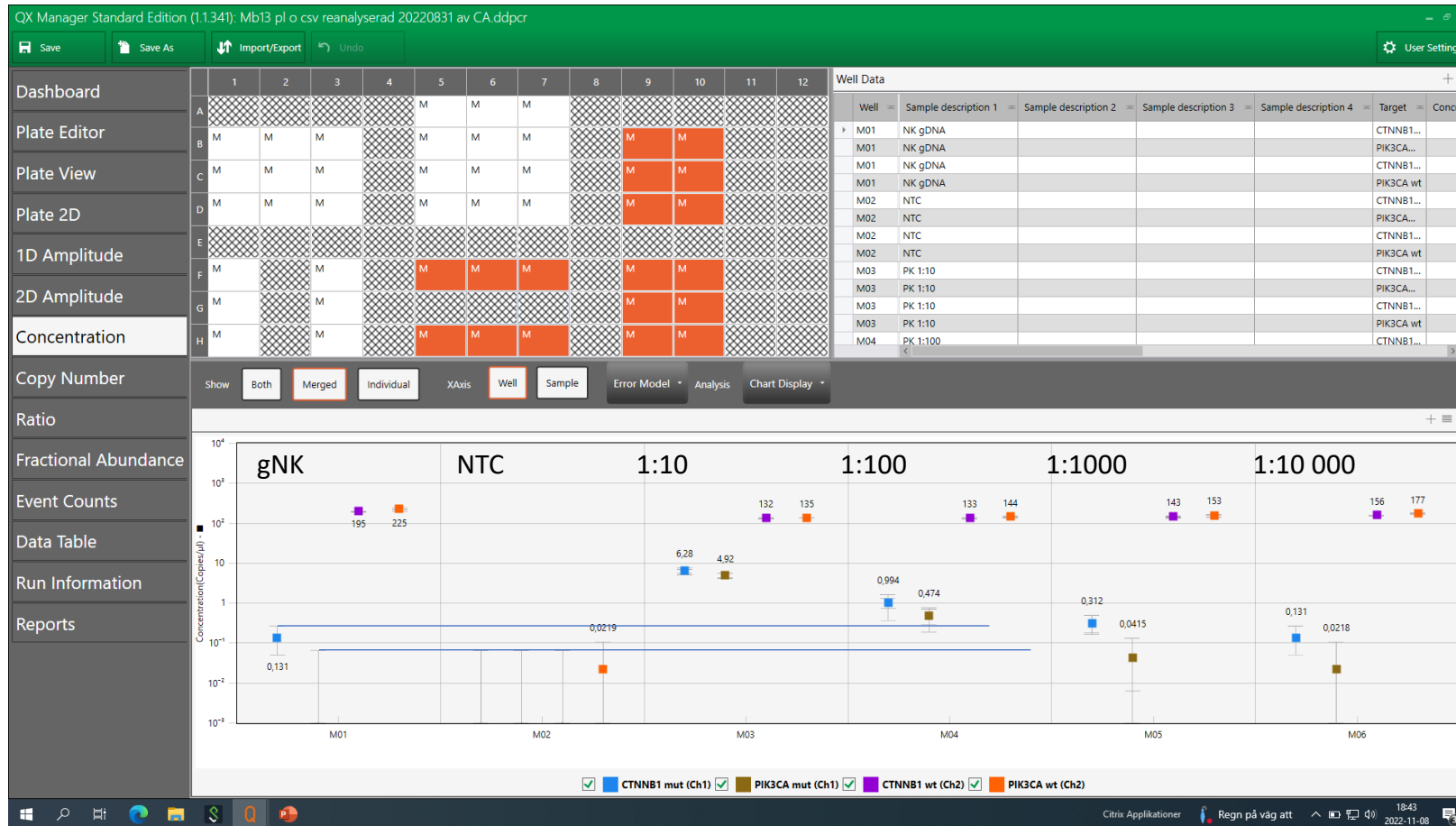

# MB13 NTC

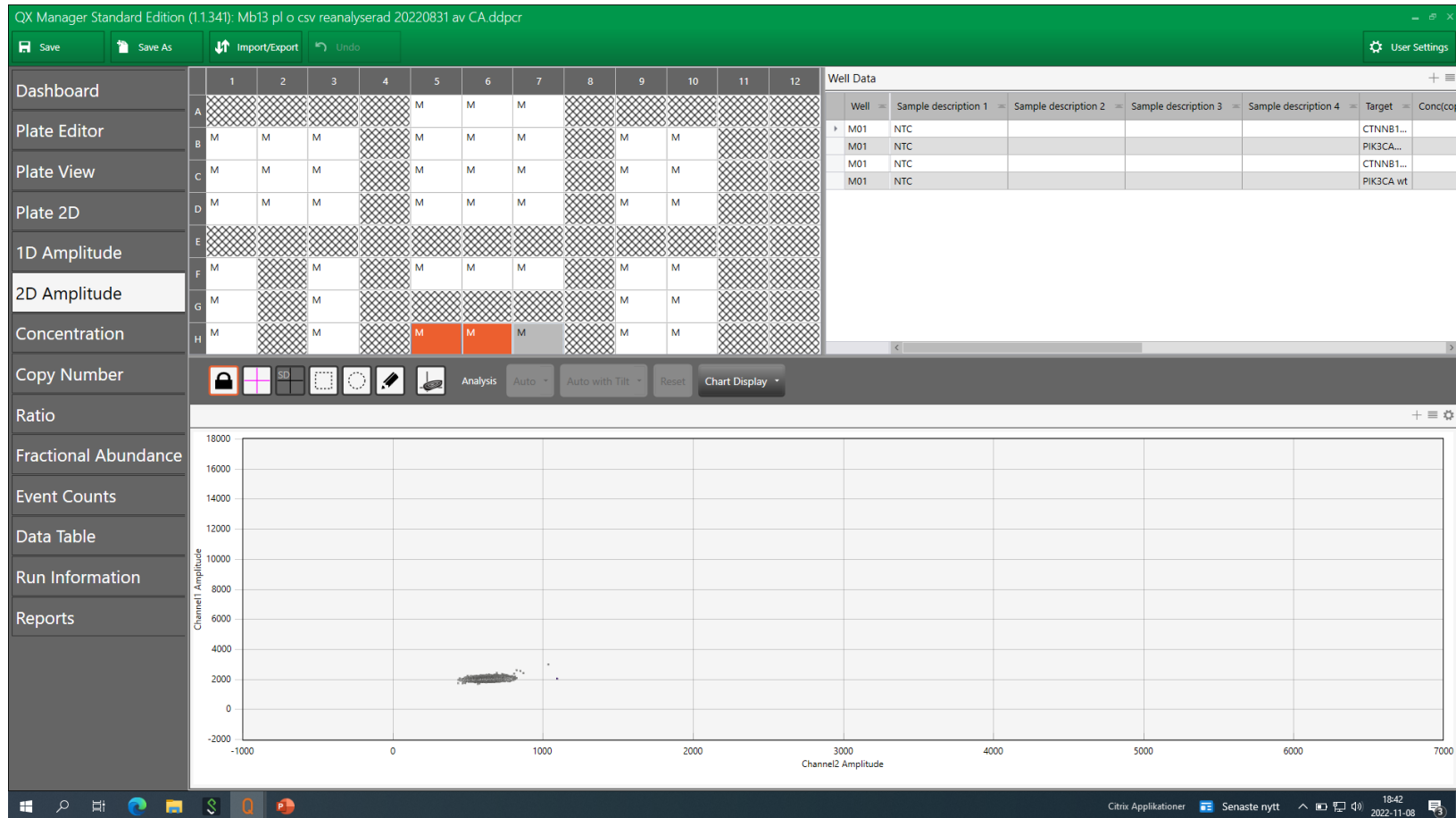

# MB13 gNC

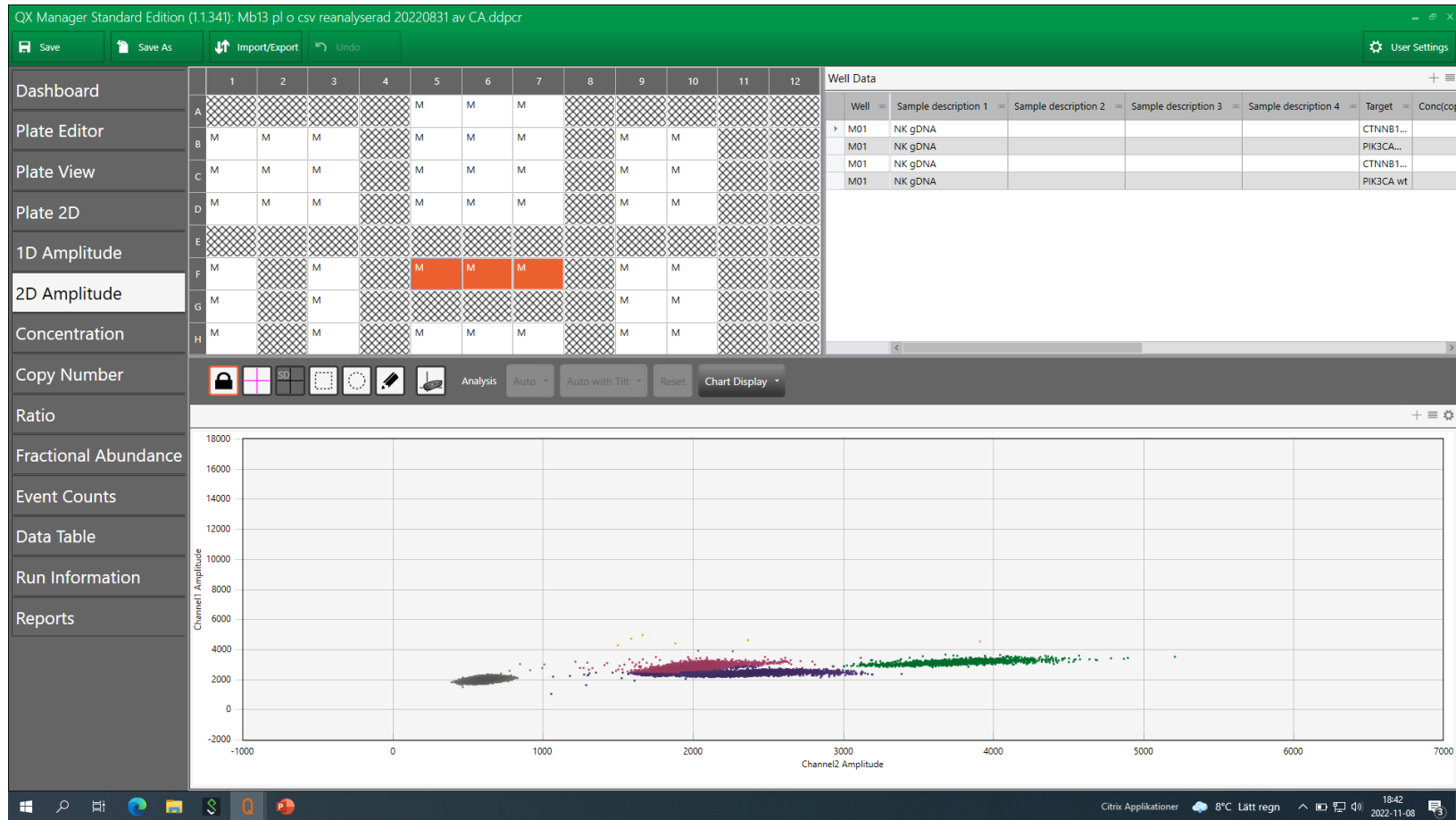

# MB13 Example of clusters

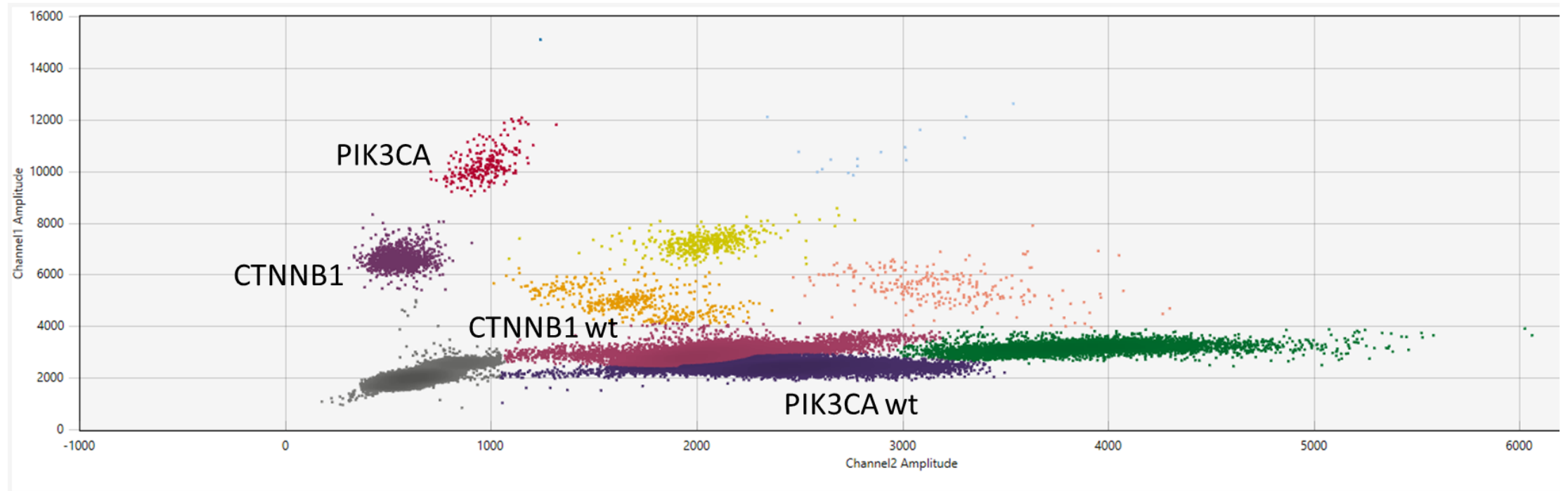

# MB13 1:10

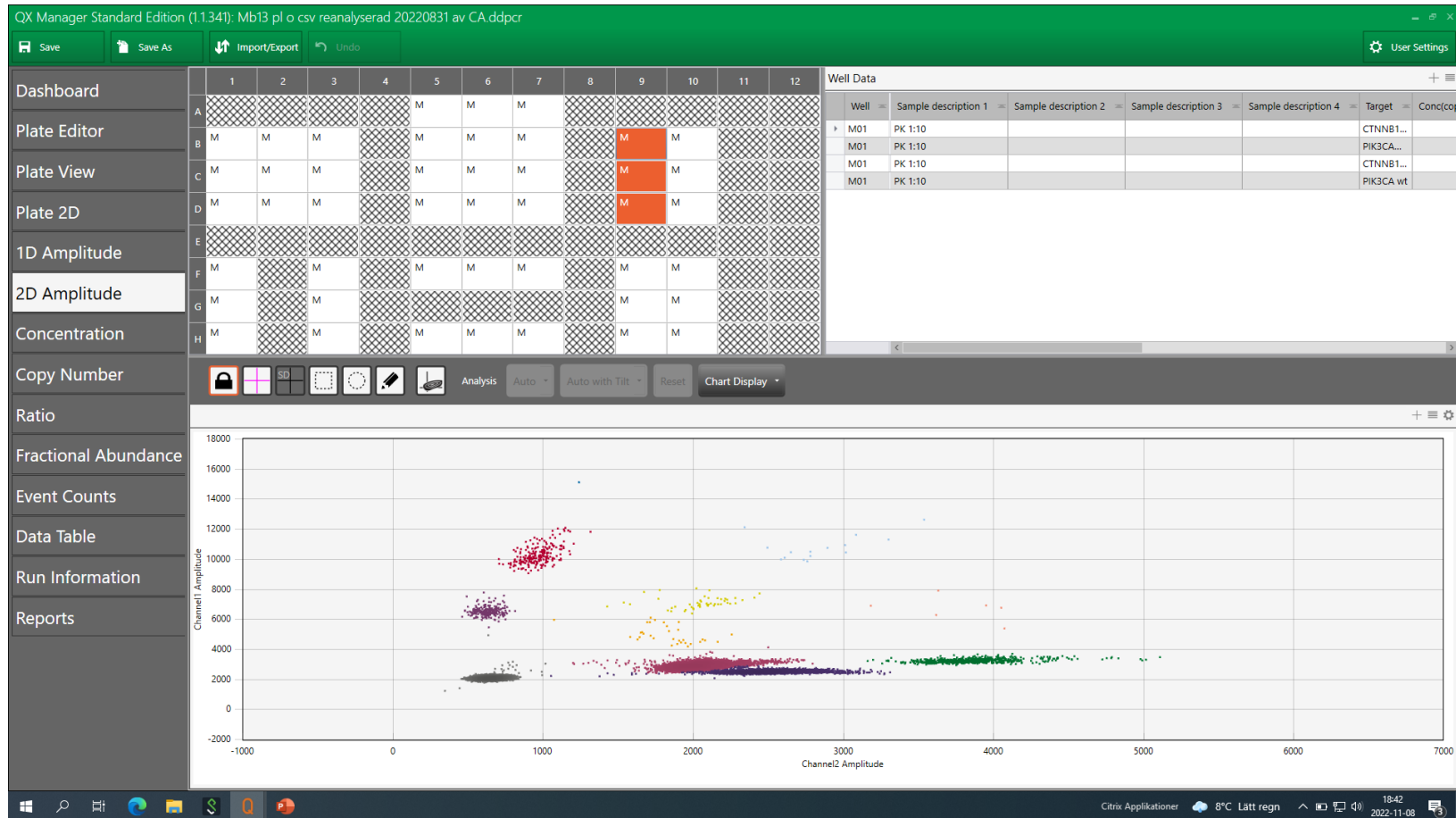

# MB13 1:100

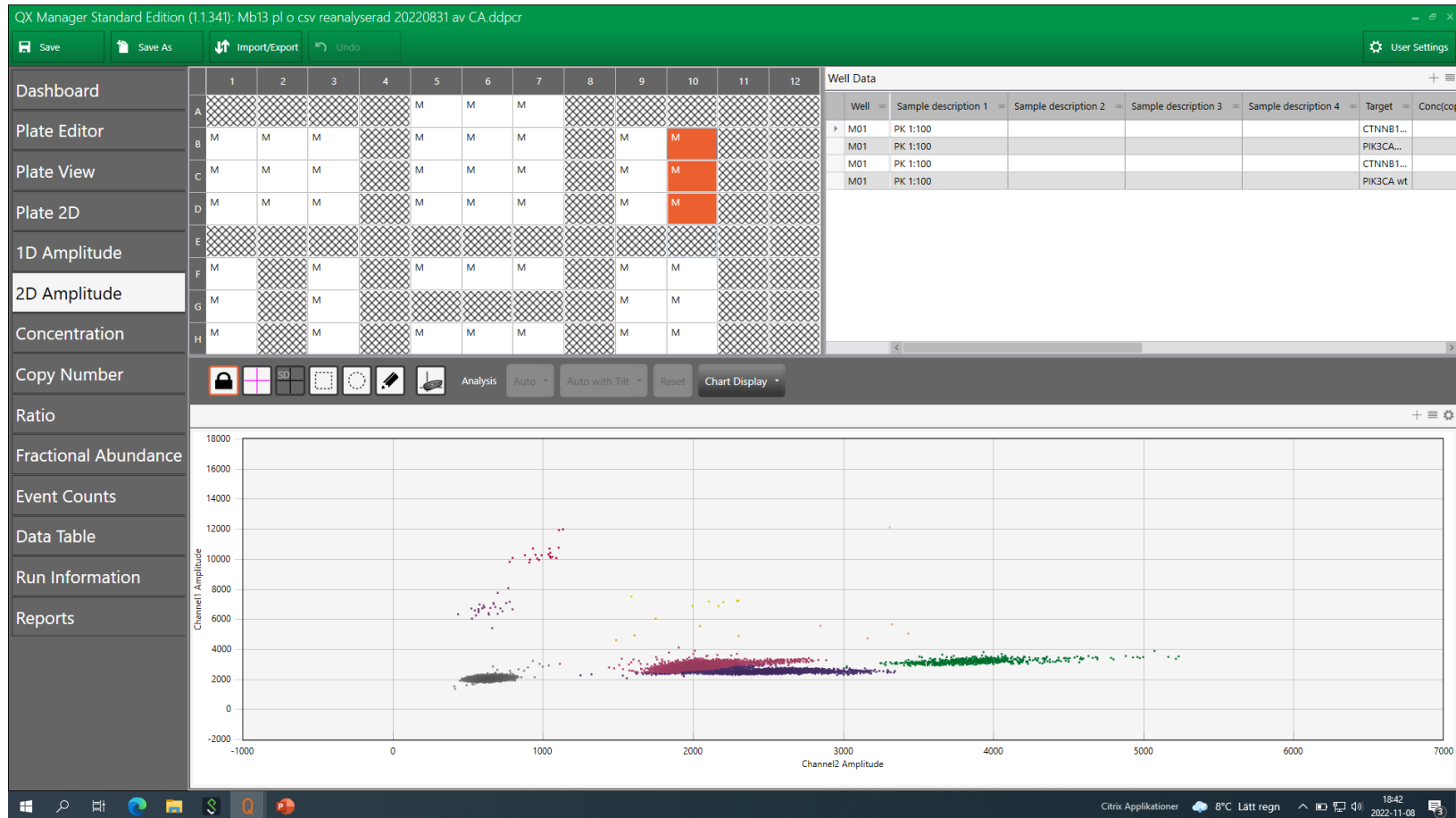

# MB13 1:1000

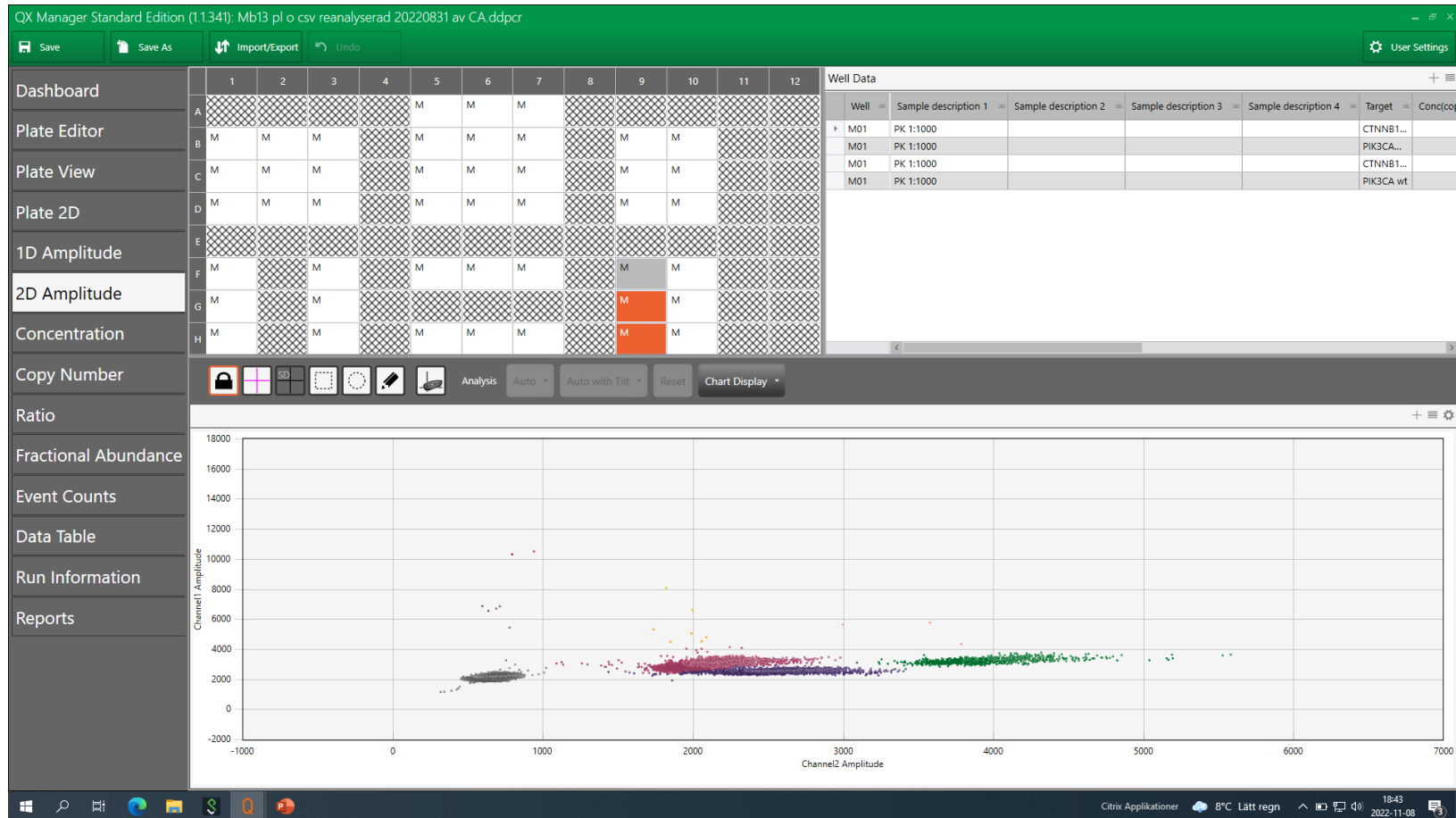

# MB13 1:10000

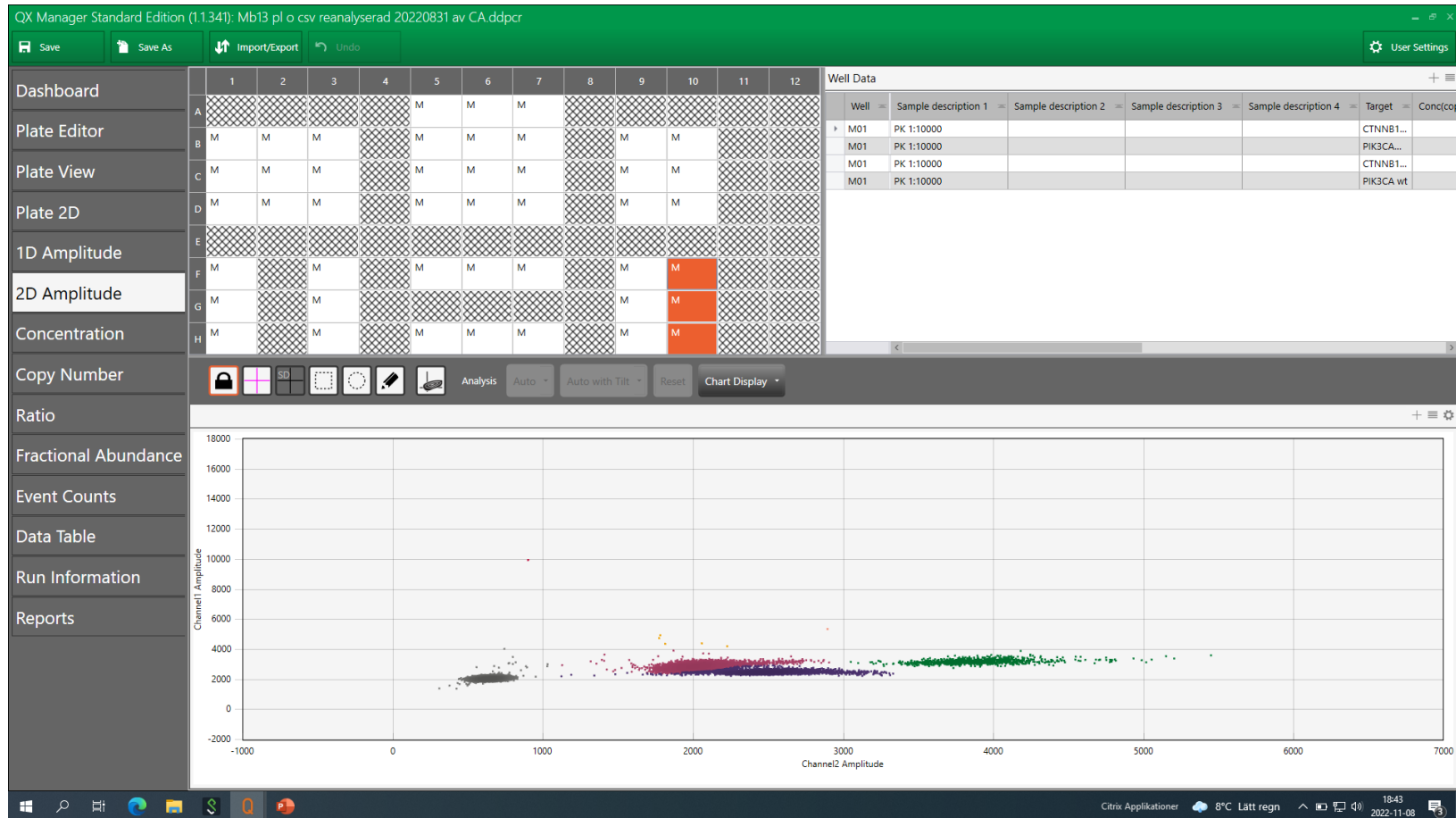

Supplement: Supplementary file 1 [file cancers-15-01972-s001.zip › File S1 QX Manager Software output data on dilution series/Dilution series MB13.pdf]

# MB15 Concentration plot

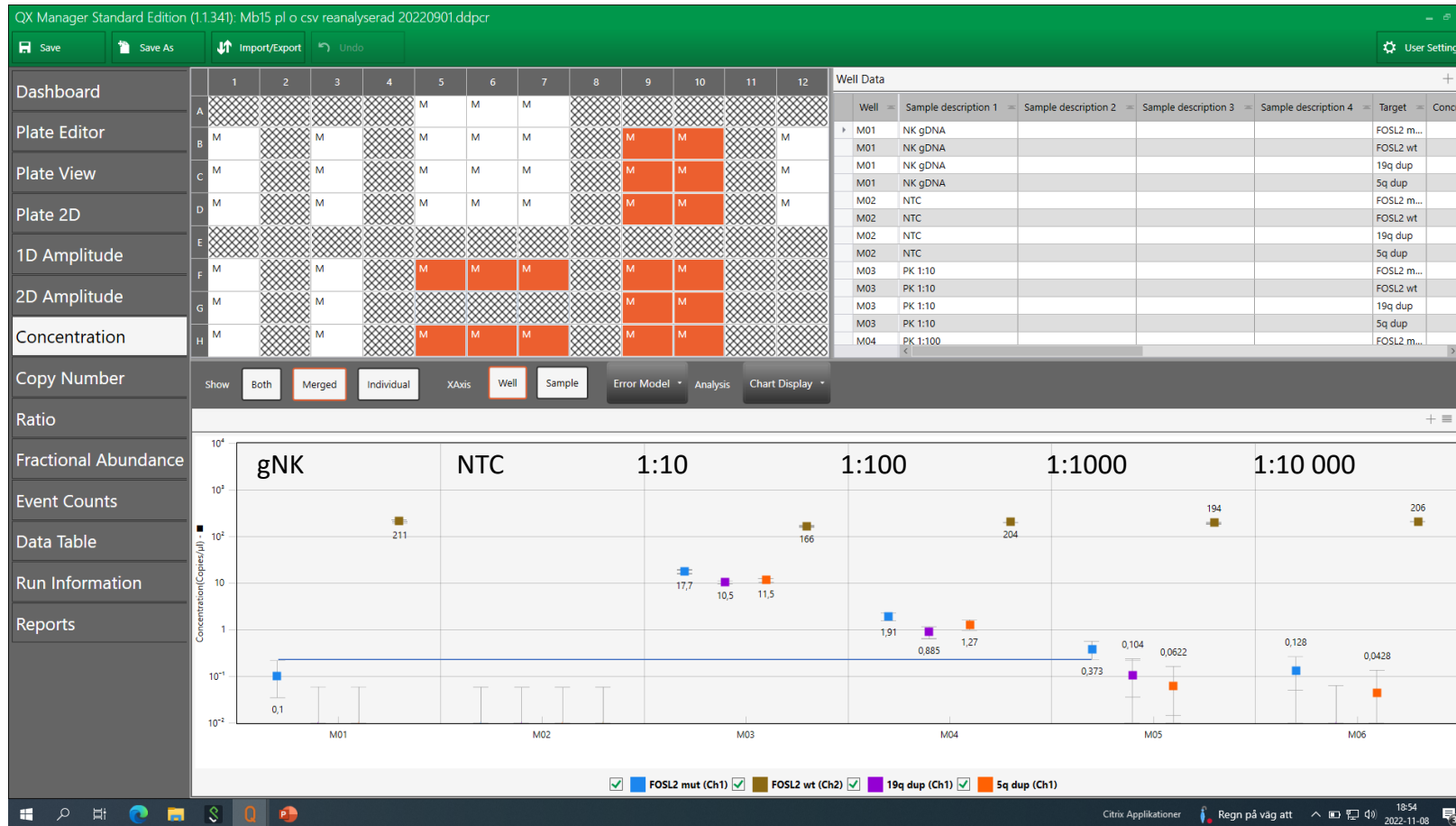

# MB15 NTC

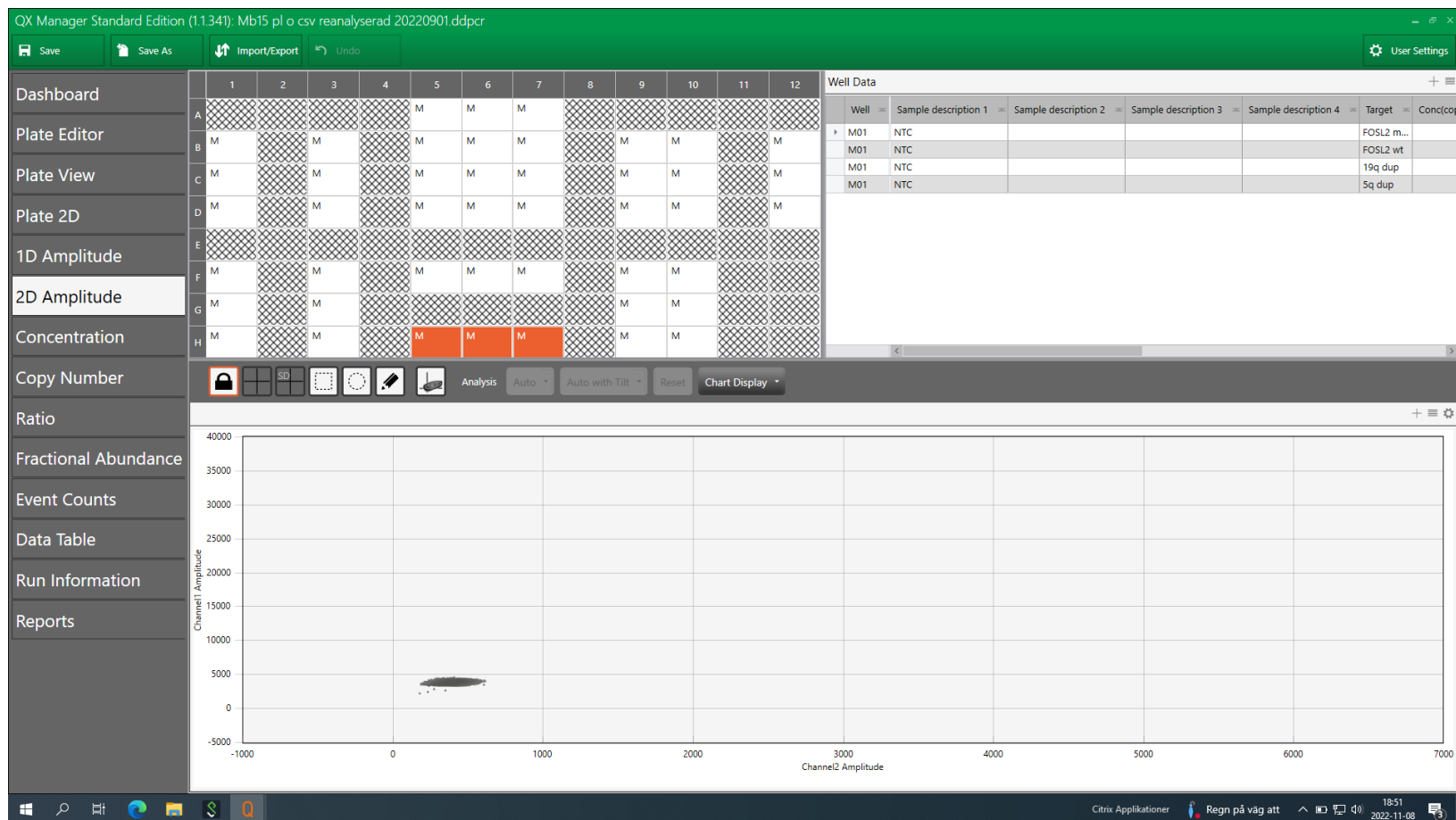

# MB15 gNC

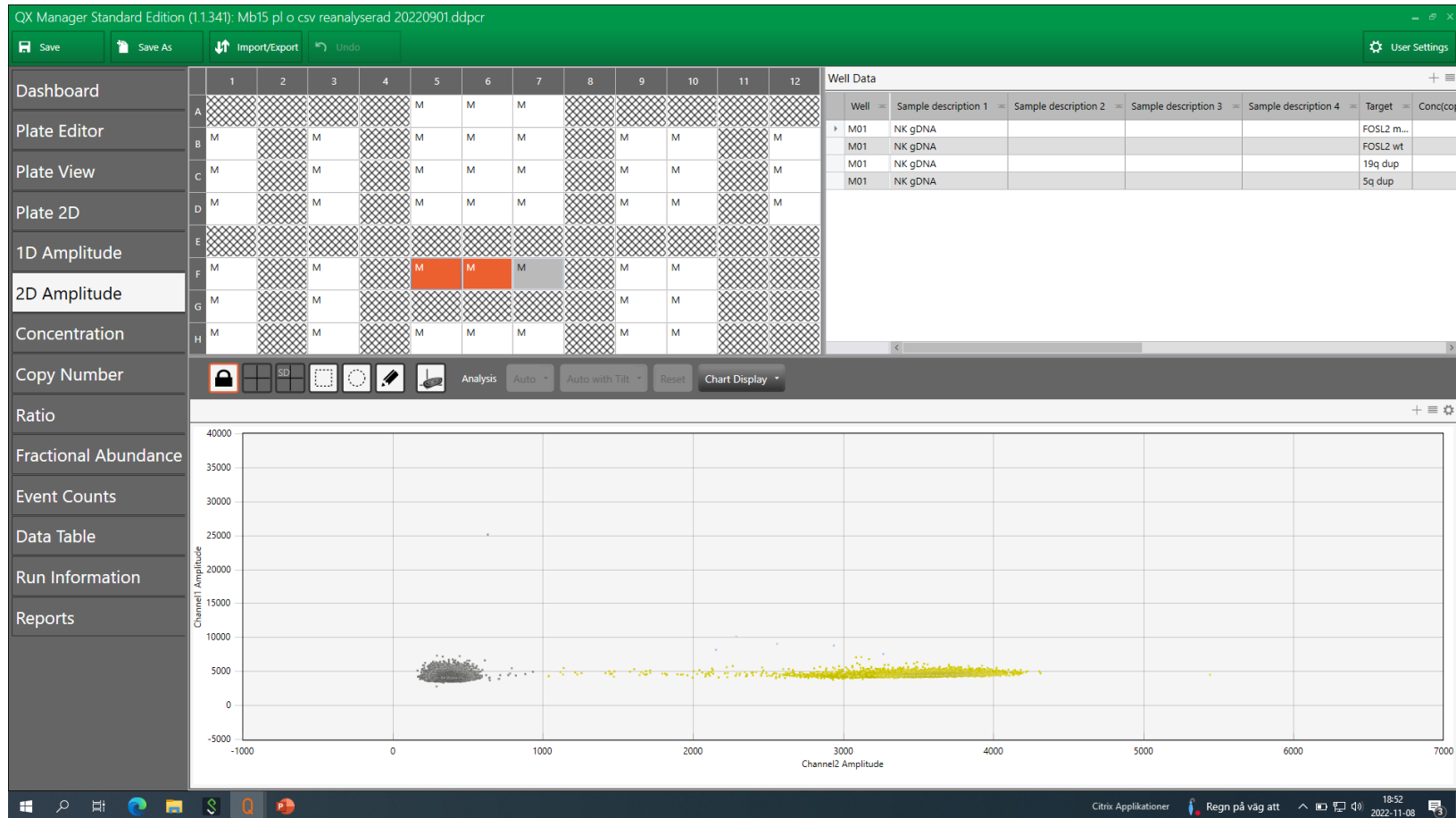

# MB15 Example of clusters

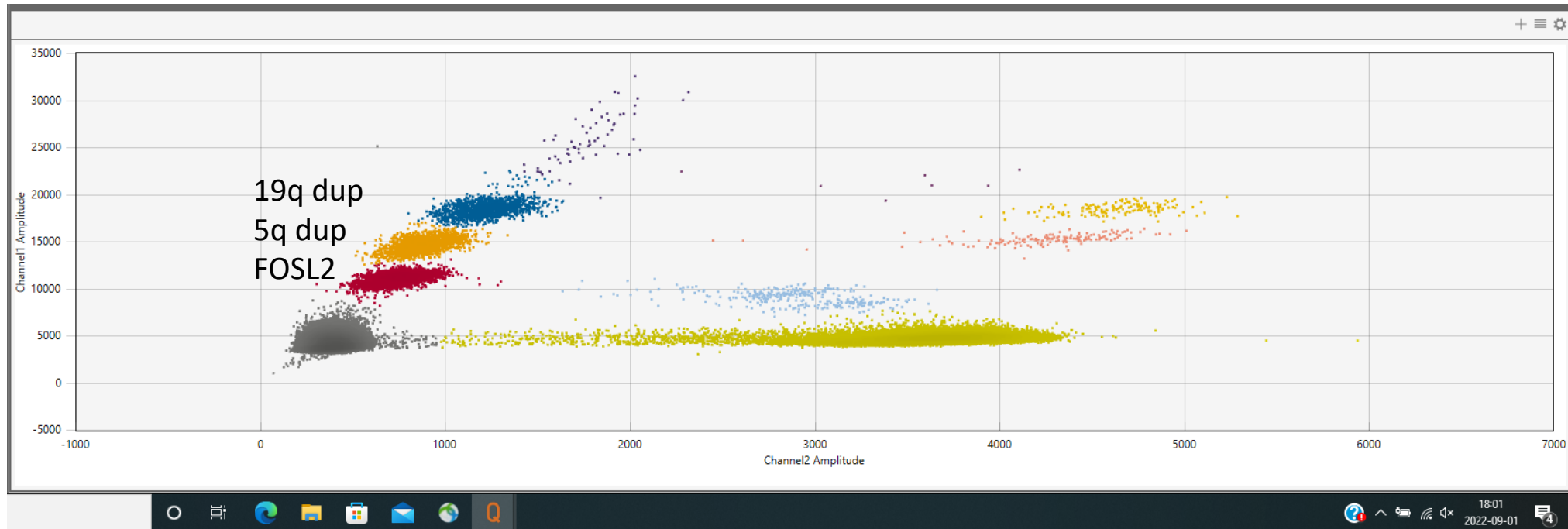

# MB15 1:10

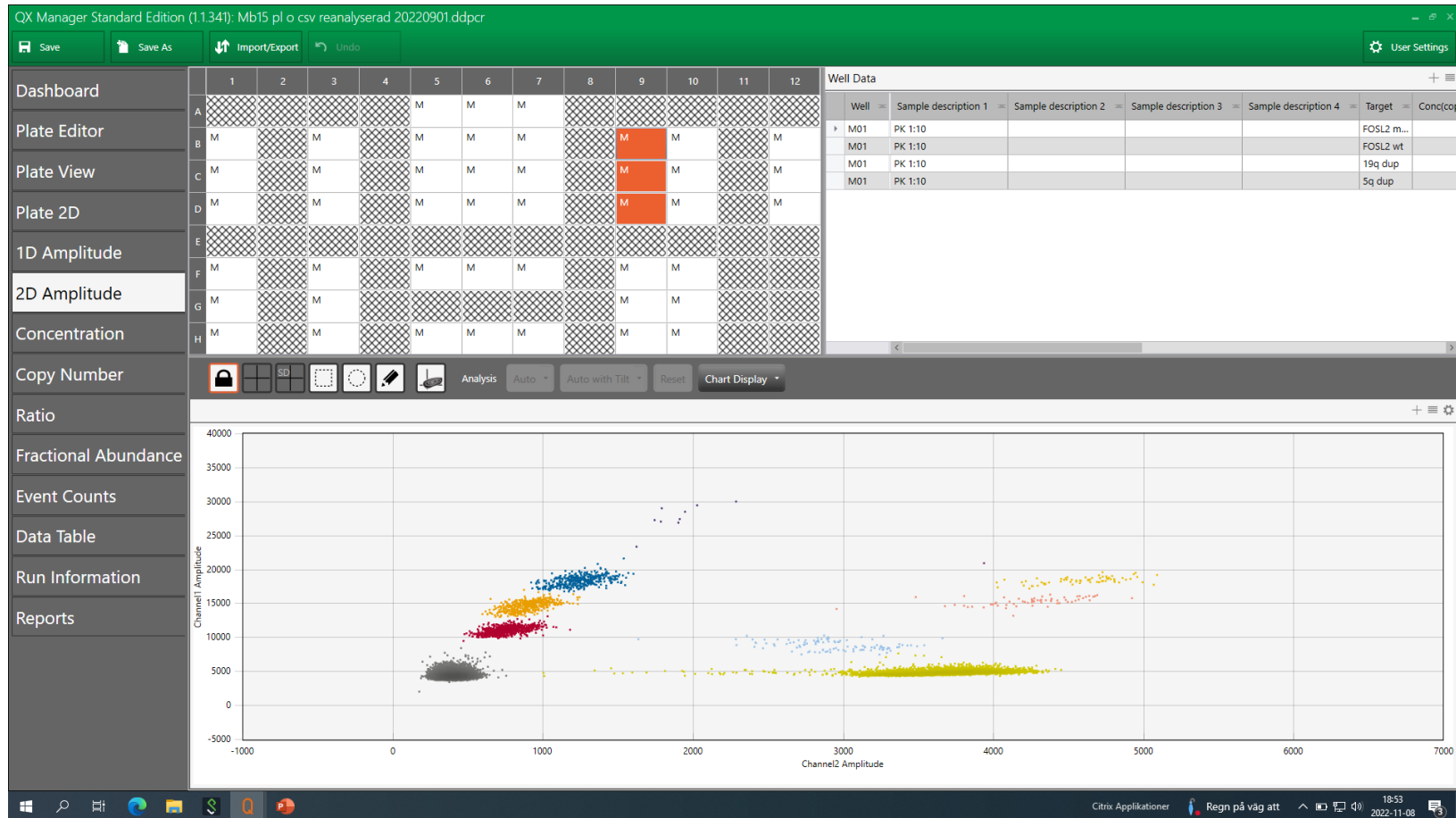

# MB15 1:100

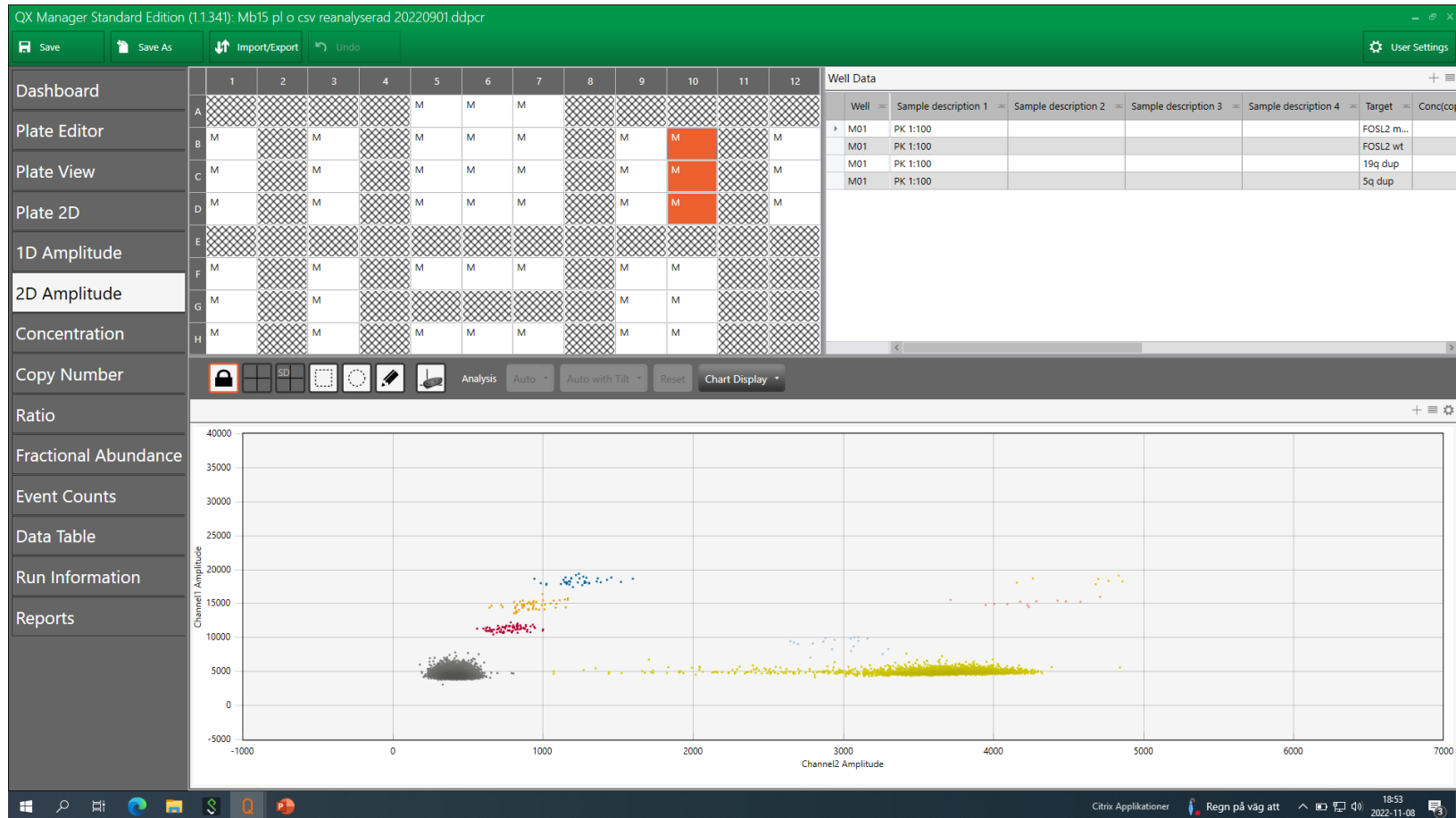

# MB15 1:1000

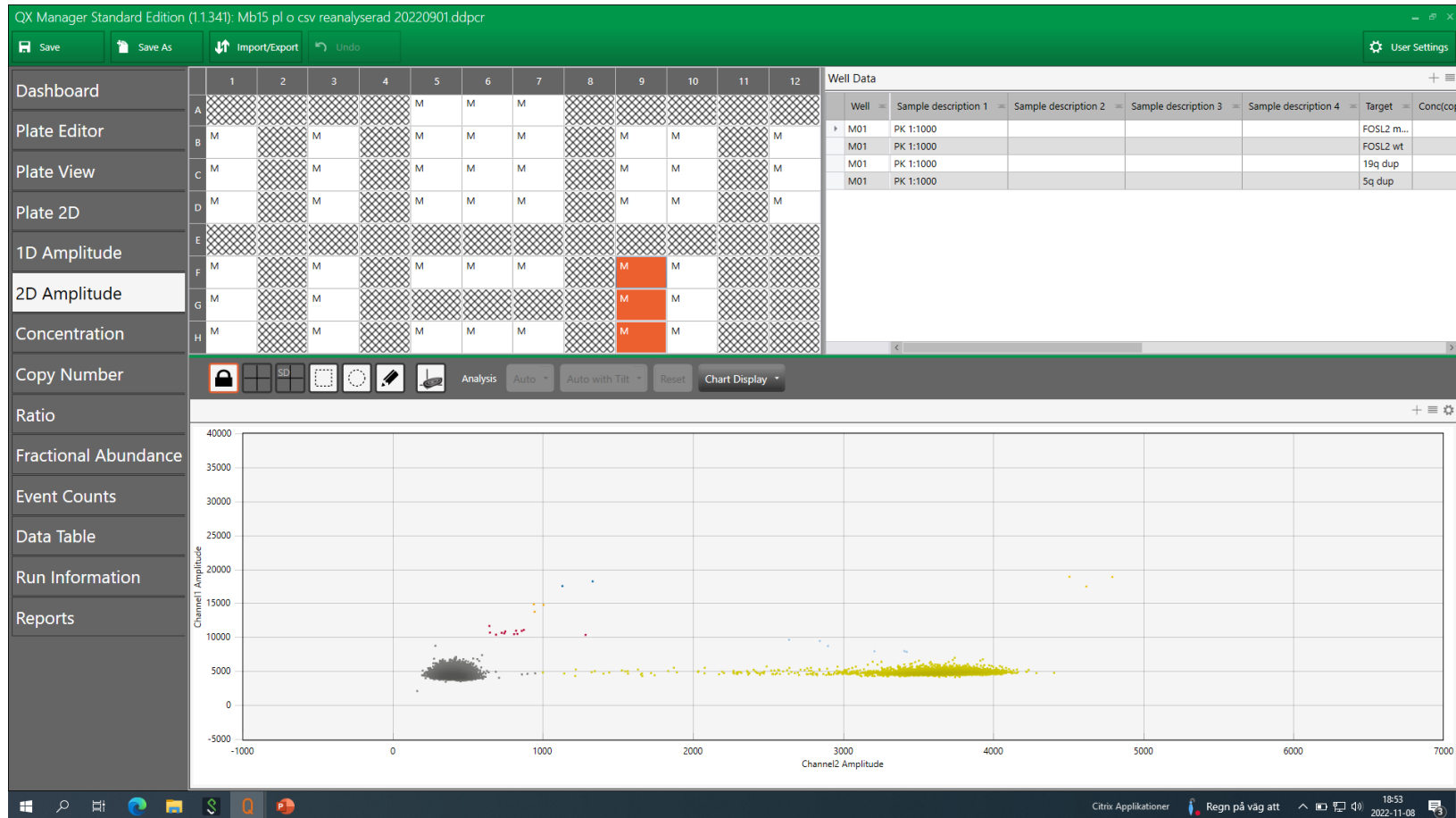

# MB15 1:10000

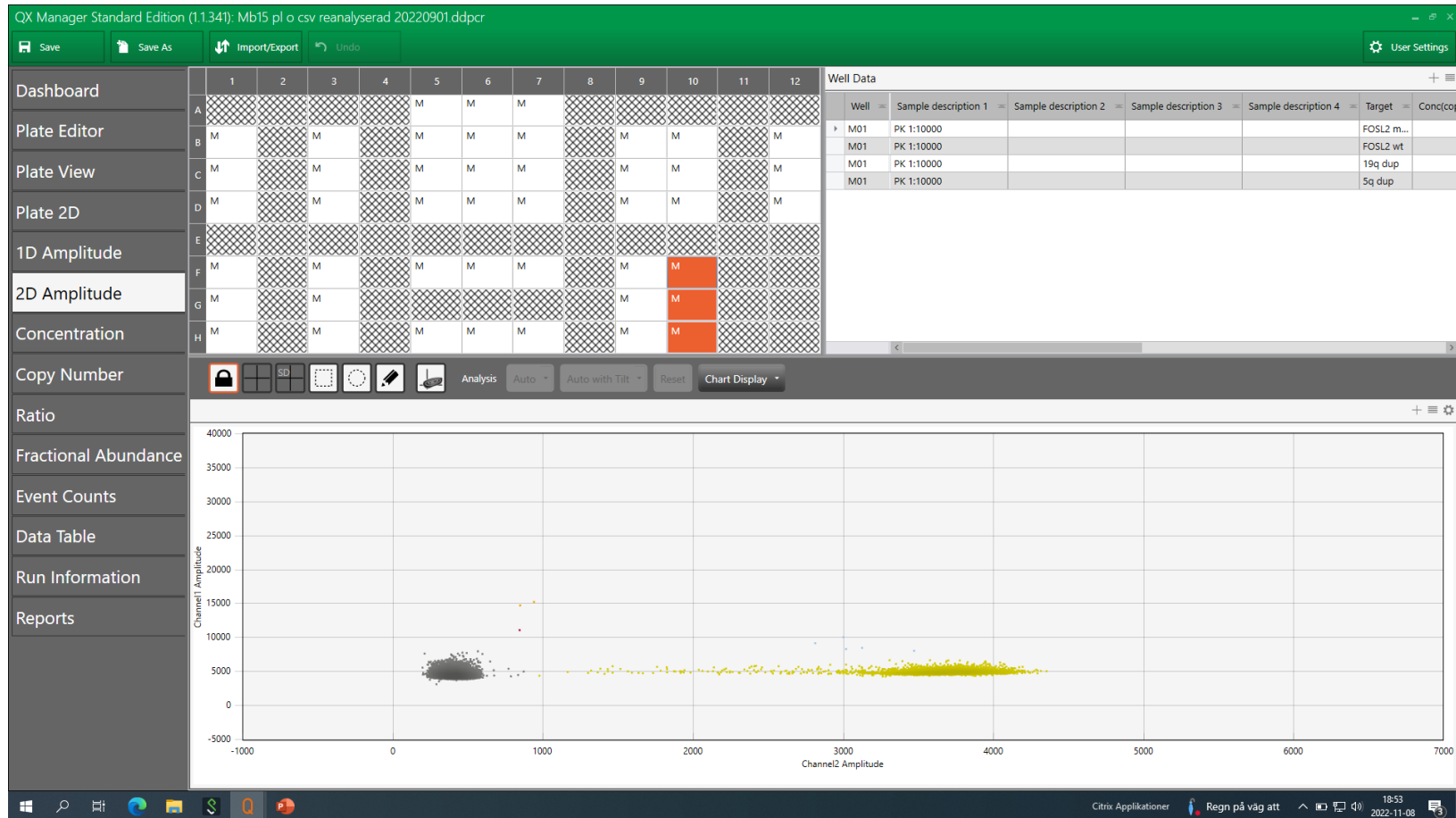

Supplement: Supplementary file 1 [file cancers-15-01972-s001.zip › File S1 QX Manager Software output data on dilution series/Dilution series MB15.pdf]

# MB19 Concentration plot

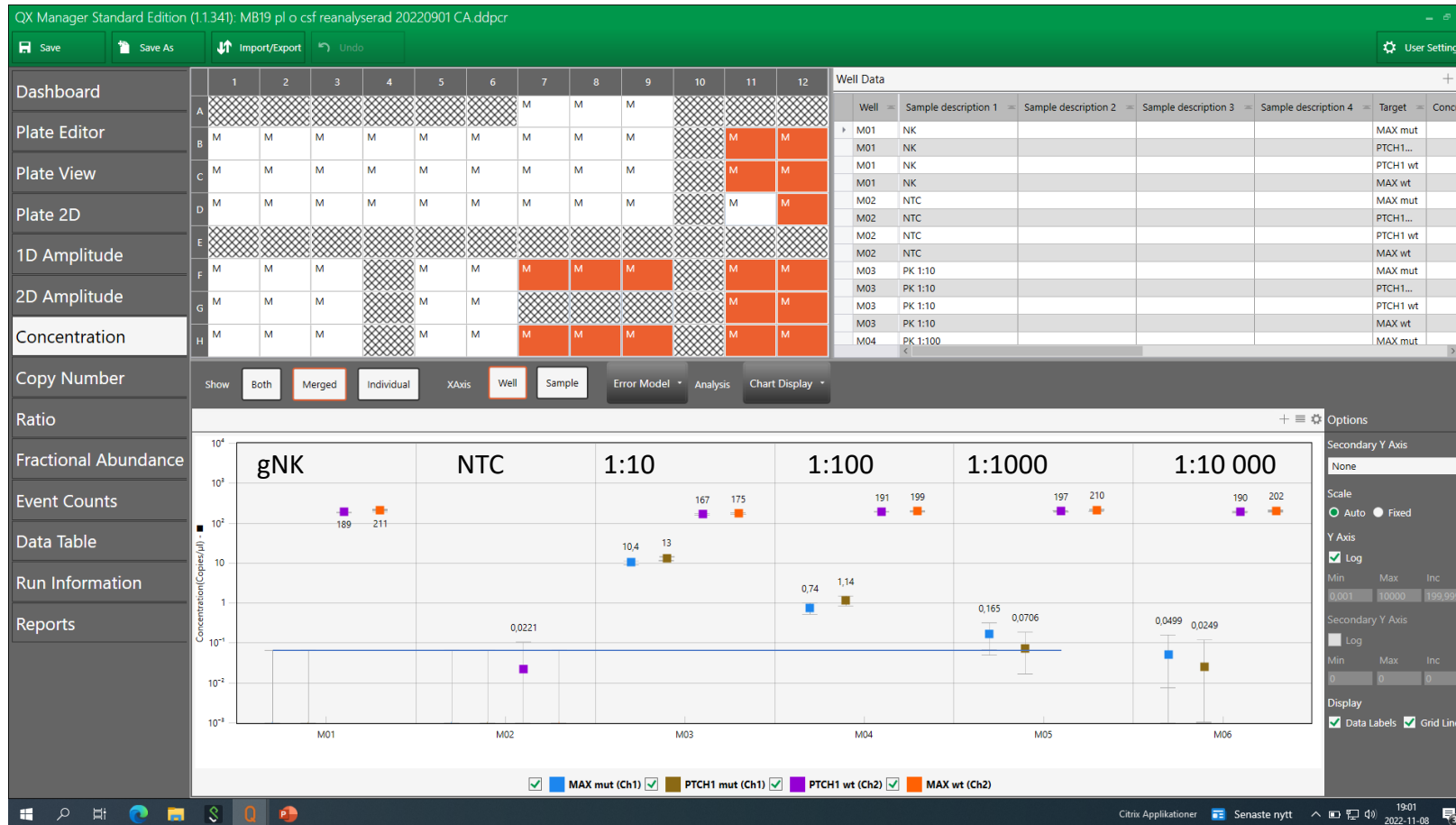

# MB19 NTC

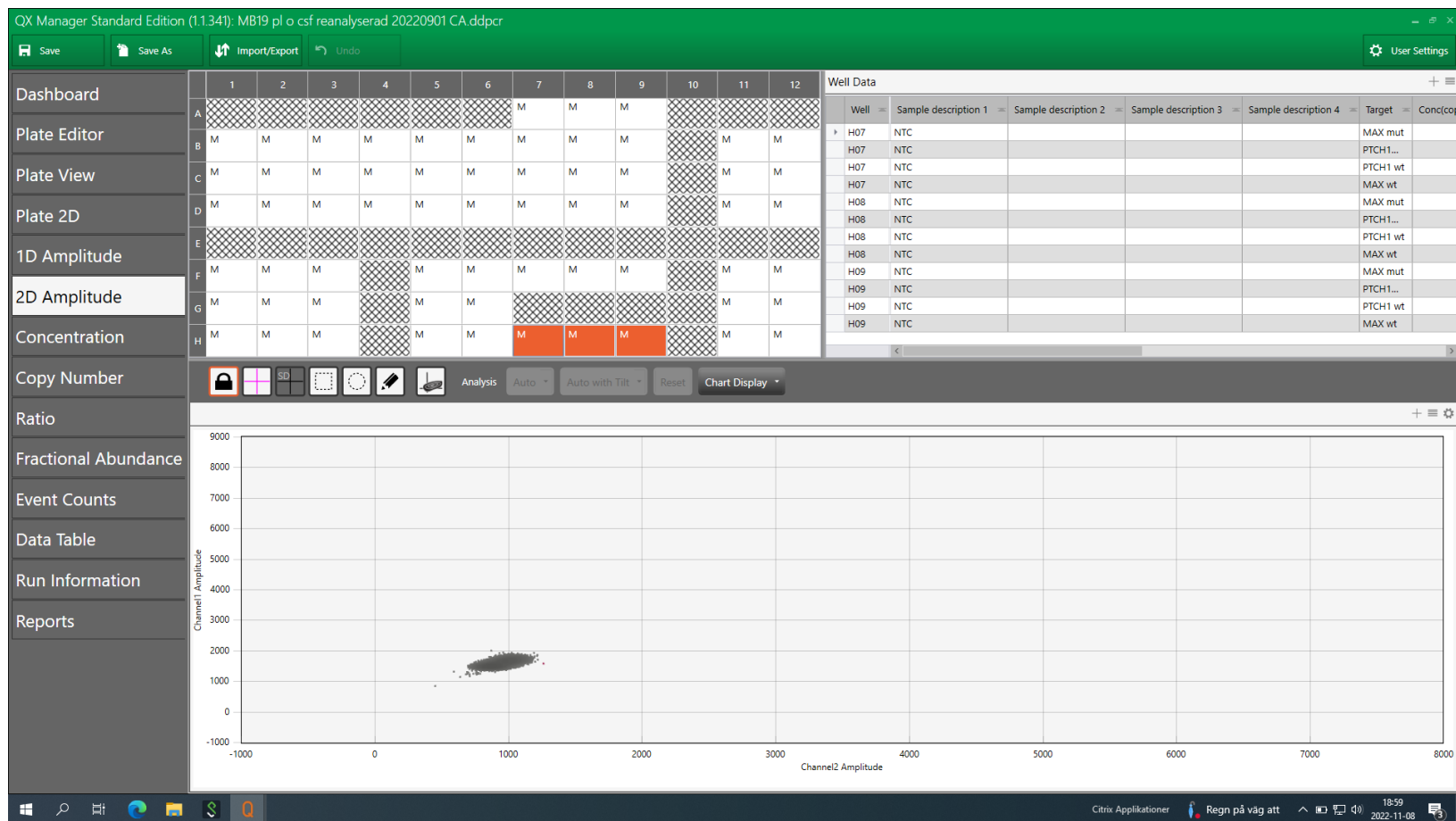

# MB19 gNC

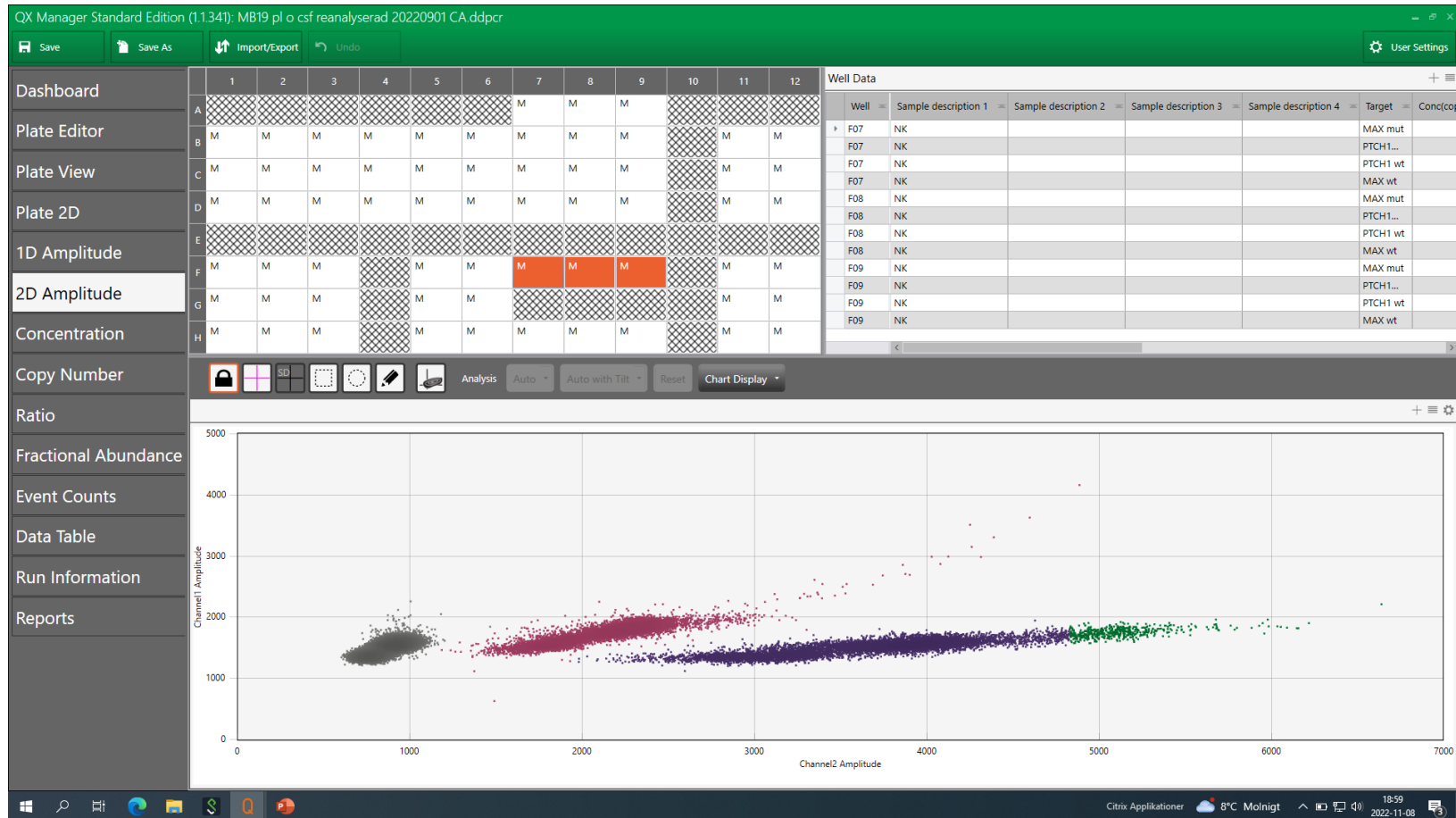

# MB19 Example of clusters

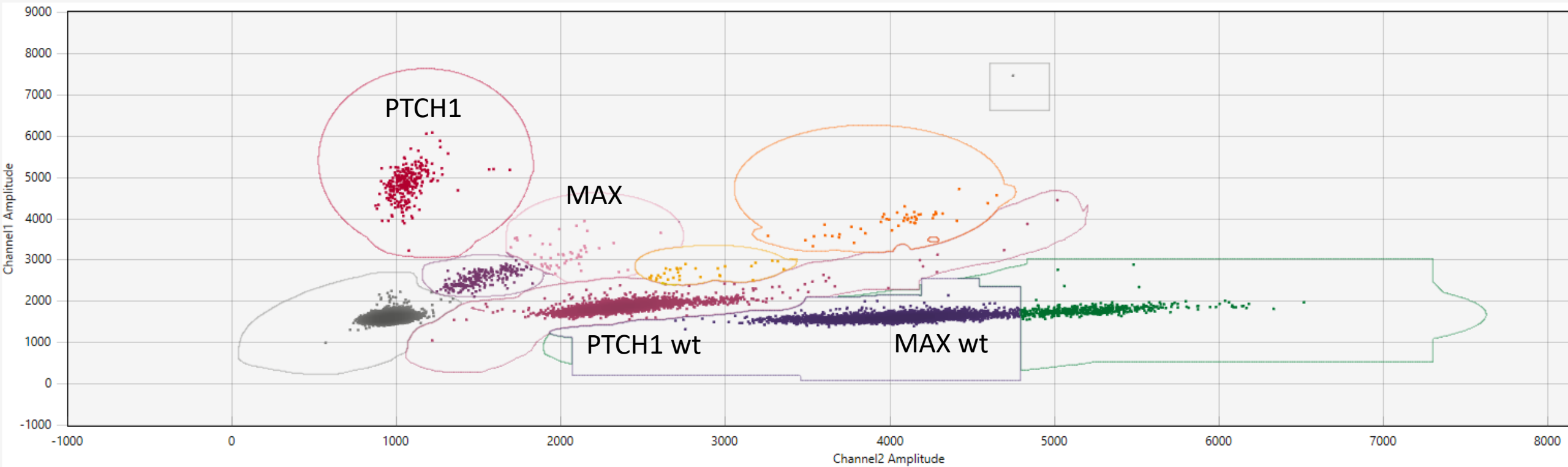

# MB19 1:10

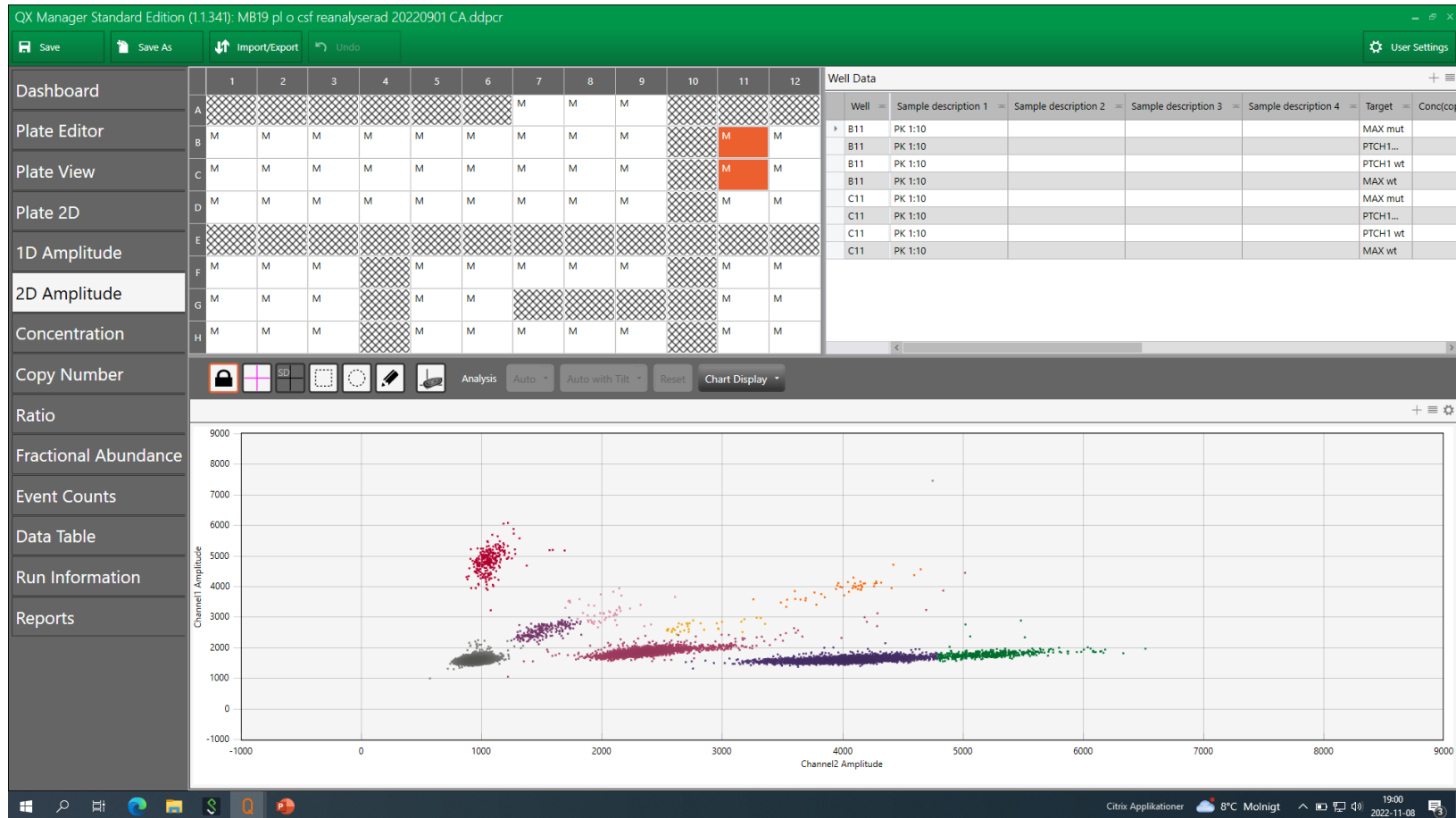

# MB19 1:100

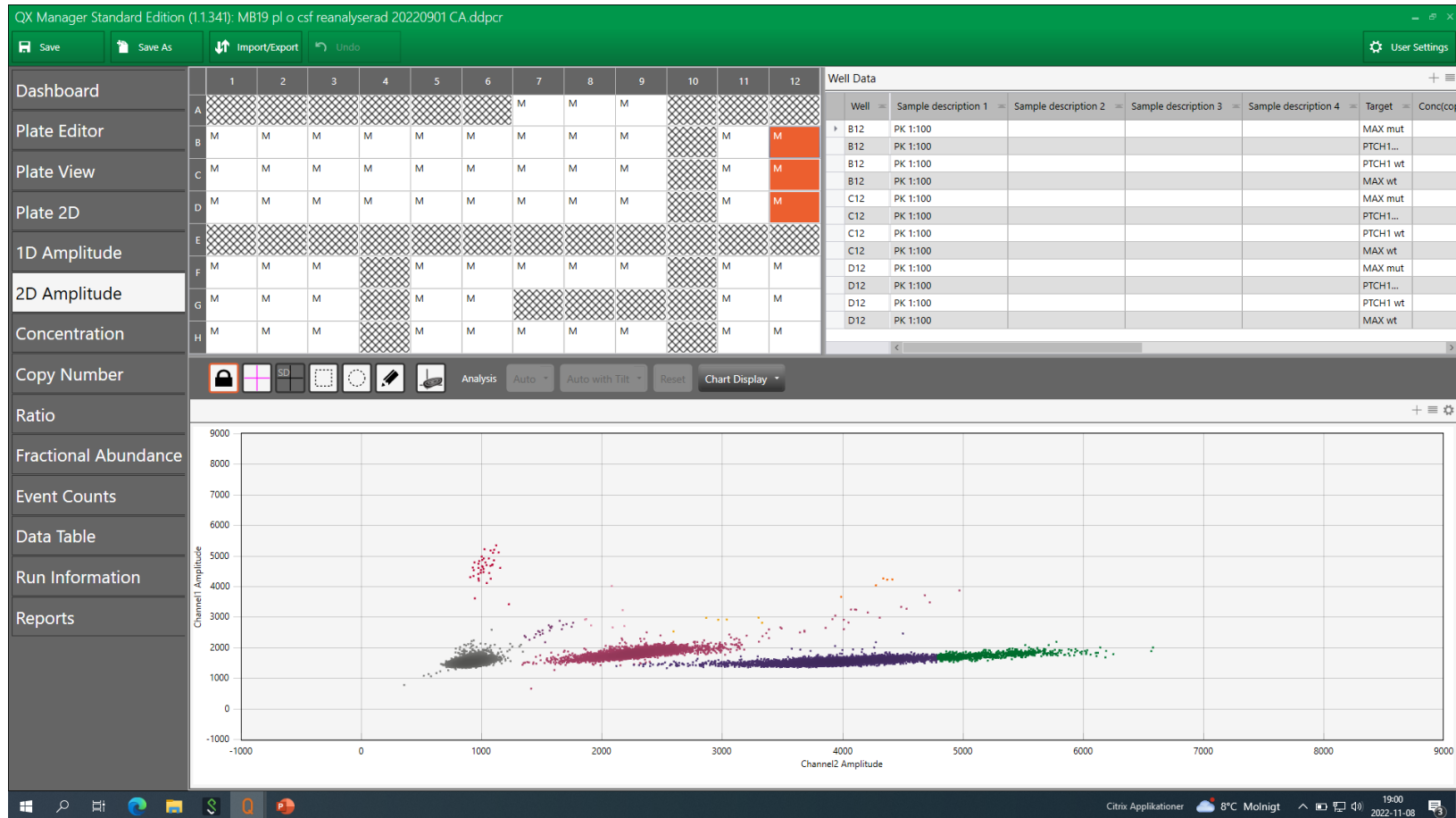

# MB19 1:1000

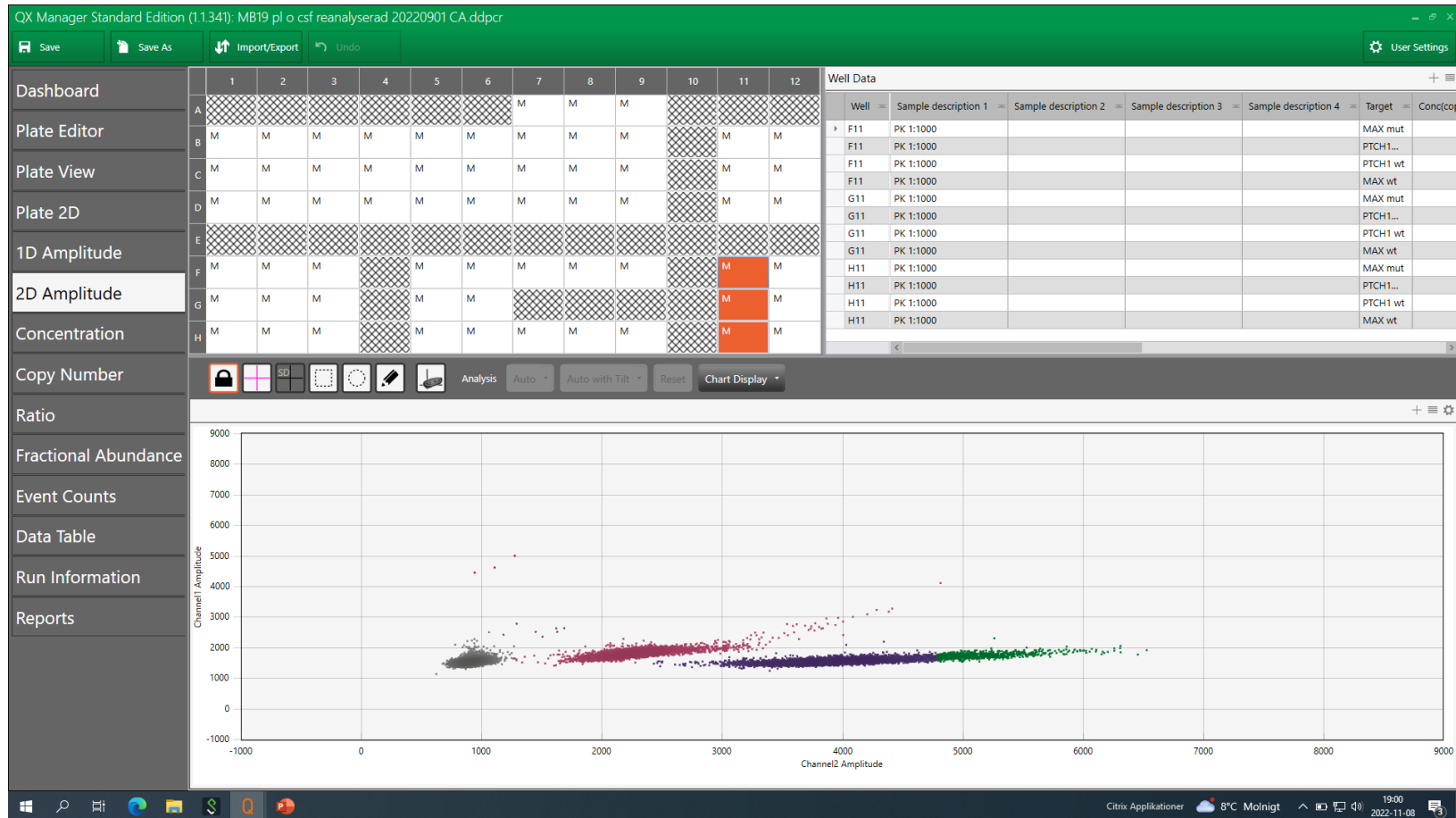

# MB19 1:10000

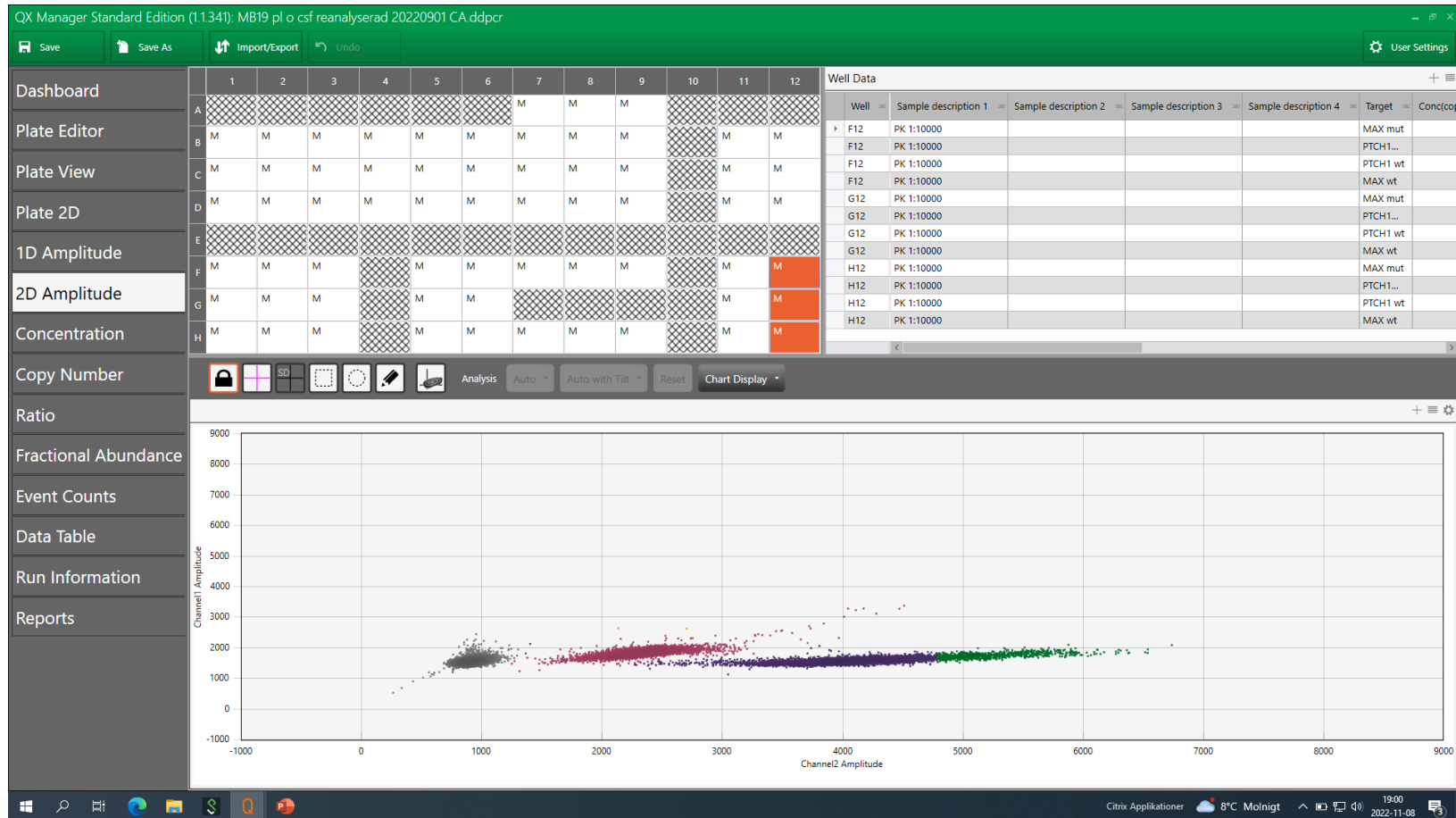

Supplement: Supplementary file 1 [file cancers-15-01972-s001.zip › File S1 QX Manager Software output data on dilution series/Dilution series MB19.pdf]

# MB21 Concentration

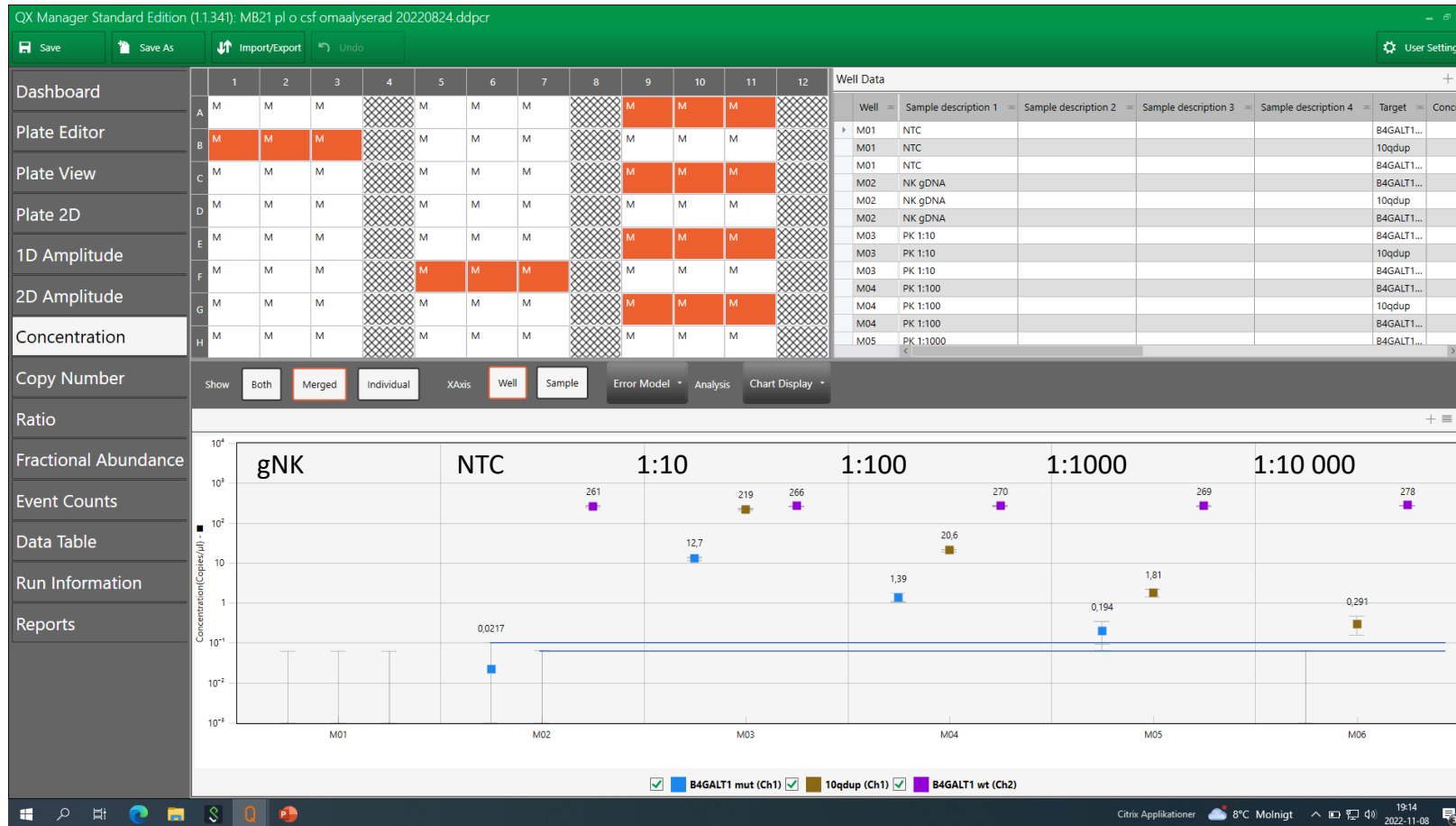

# MB21 NTC

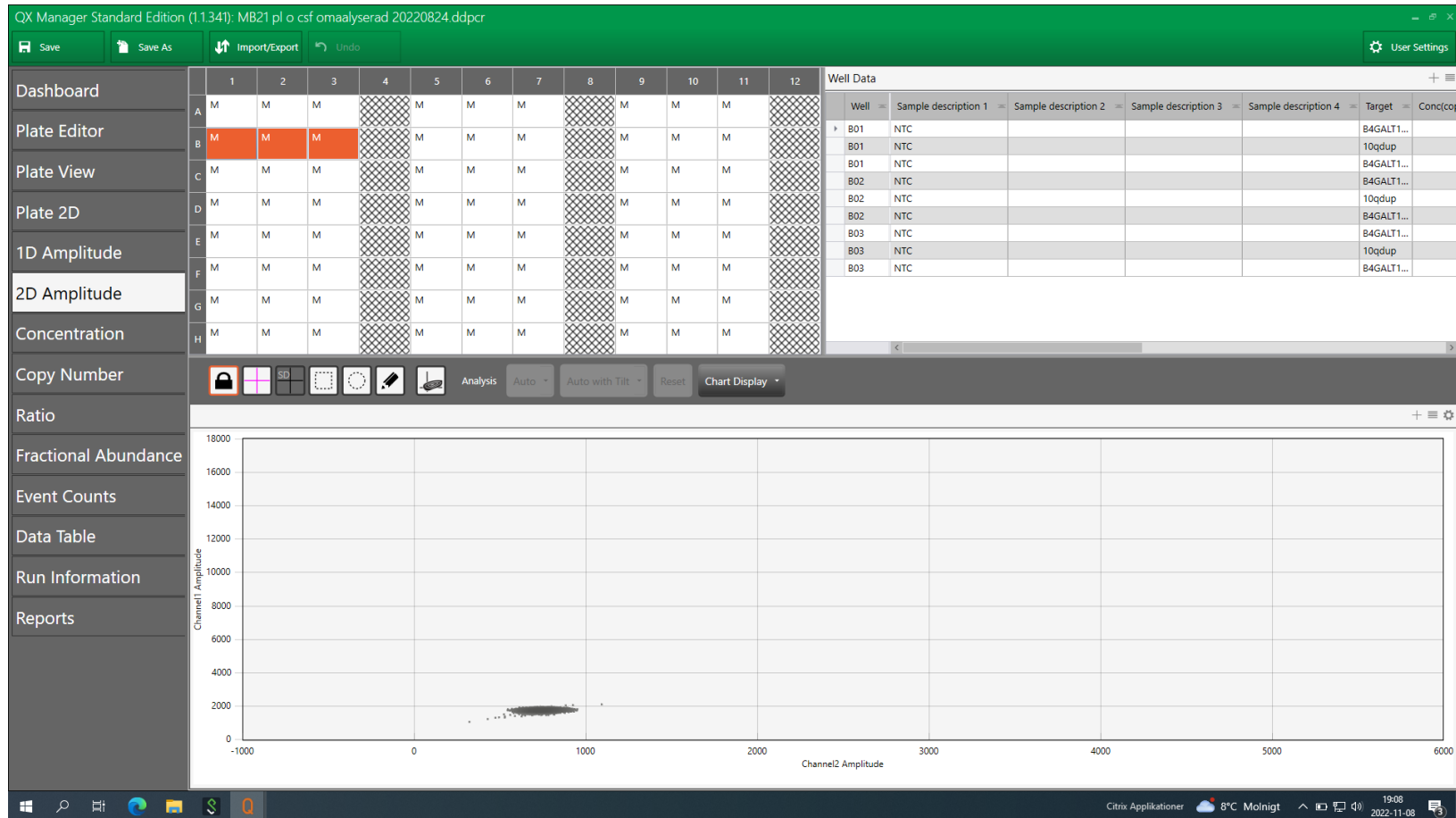

# MB21 gNC

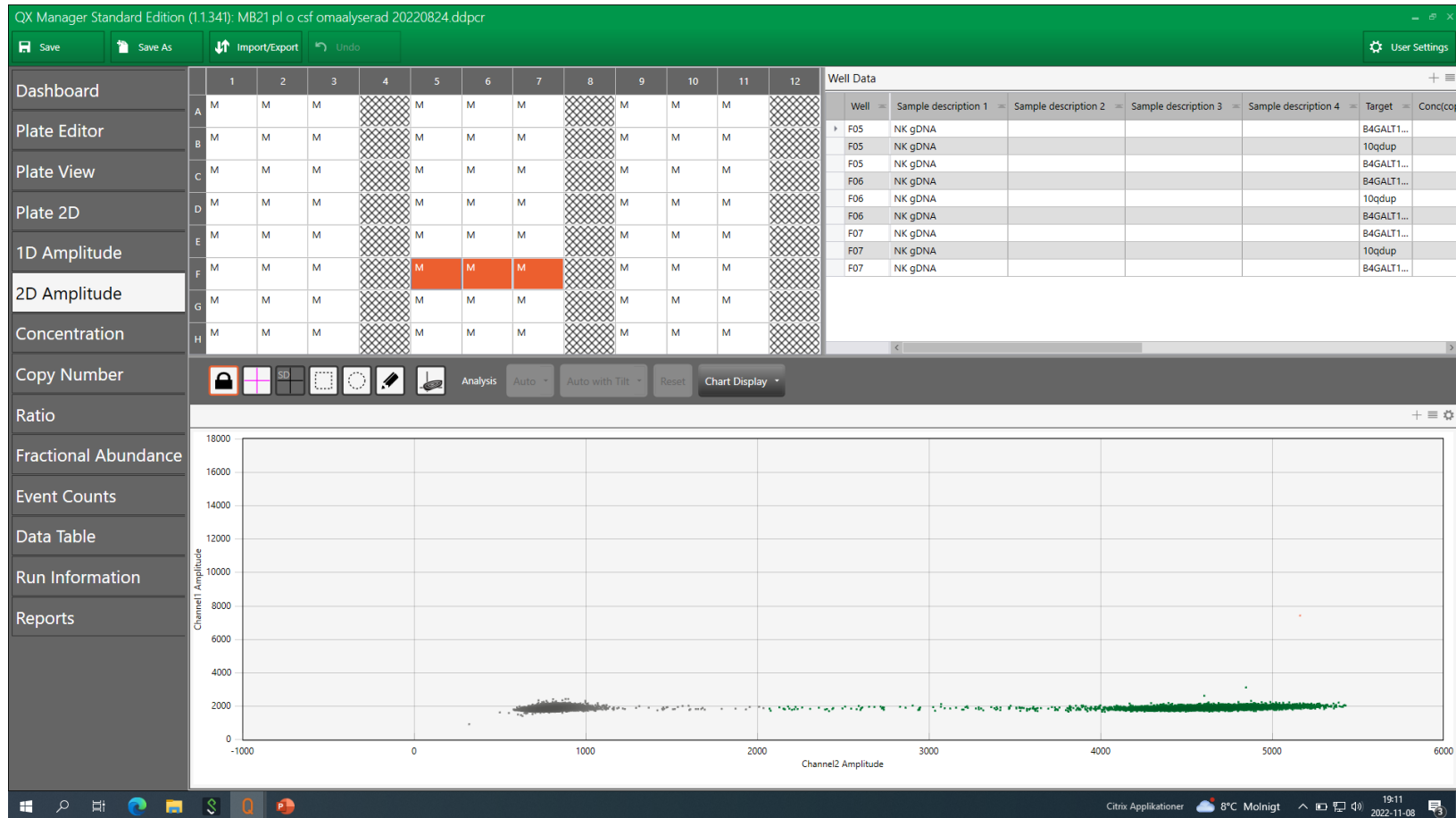

# MB21 Example of clusters

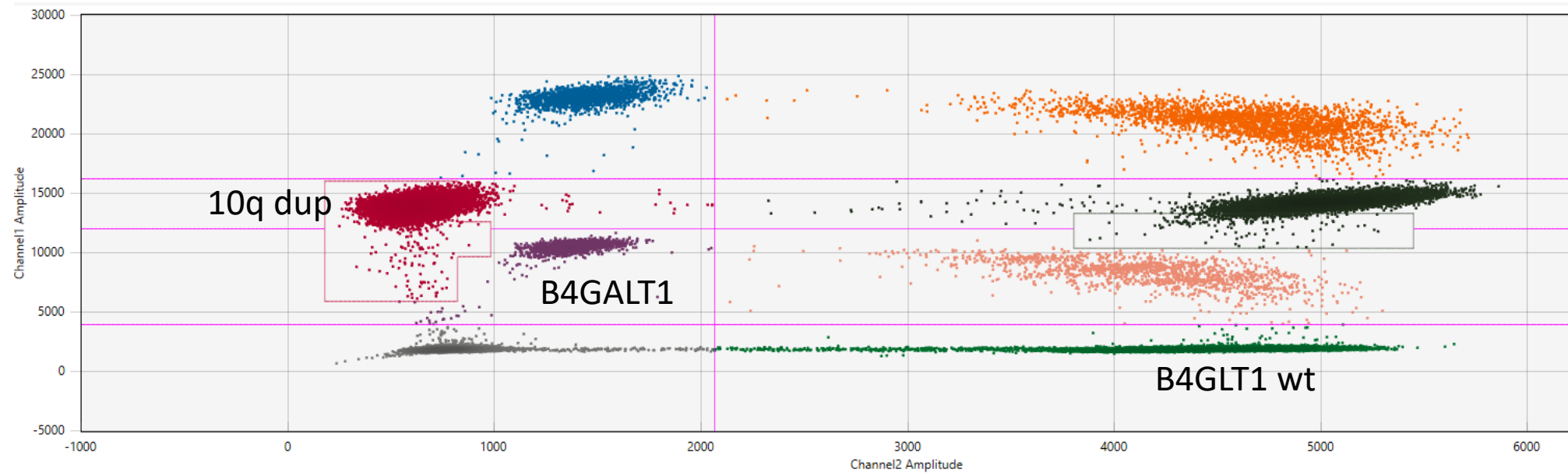

# MB21 1:10

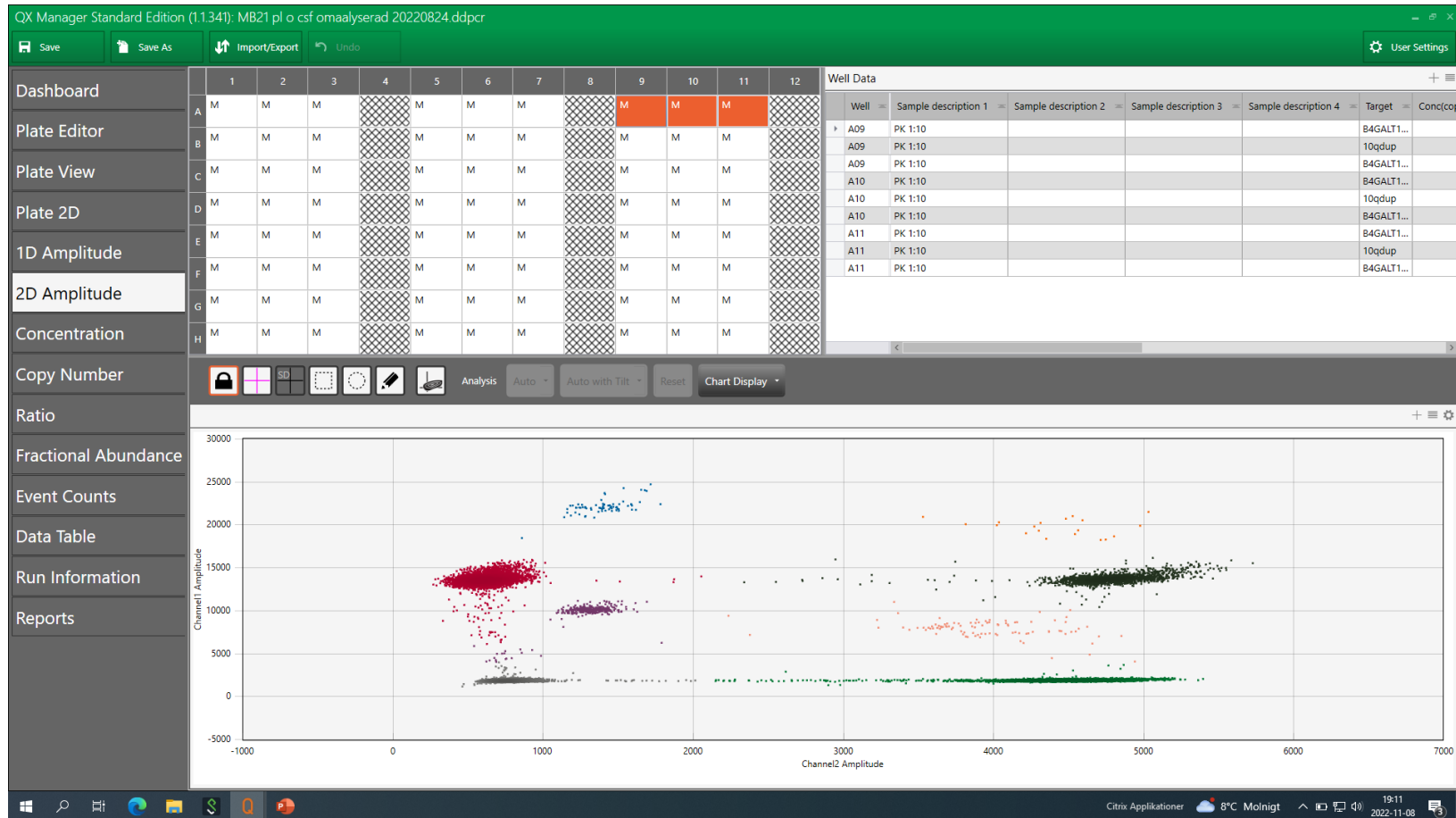

# MB21 1:100

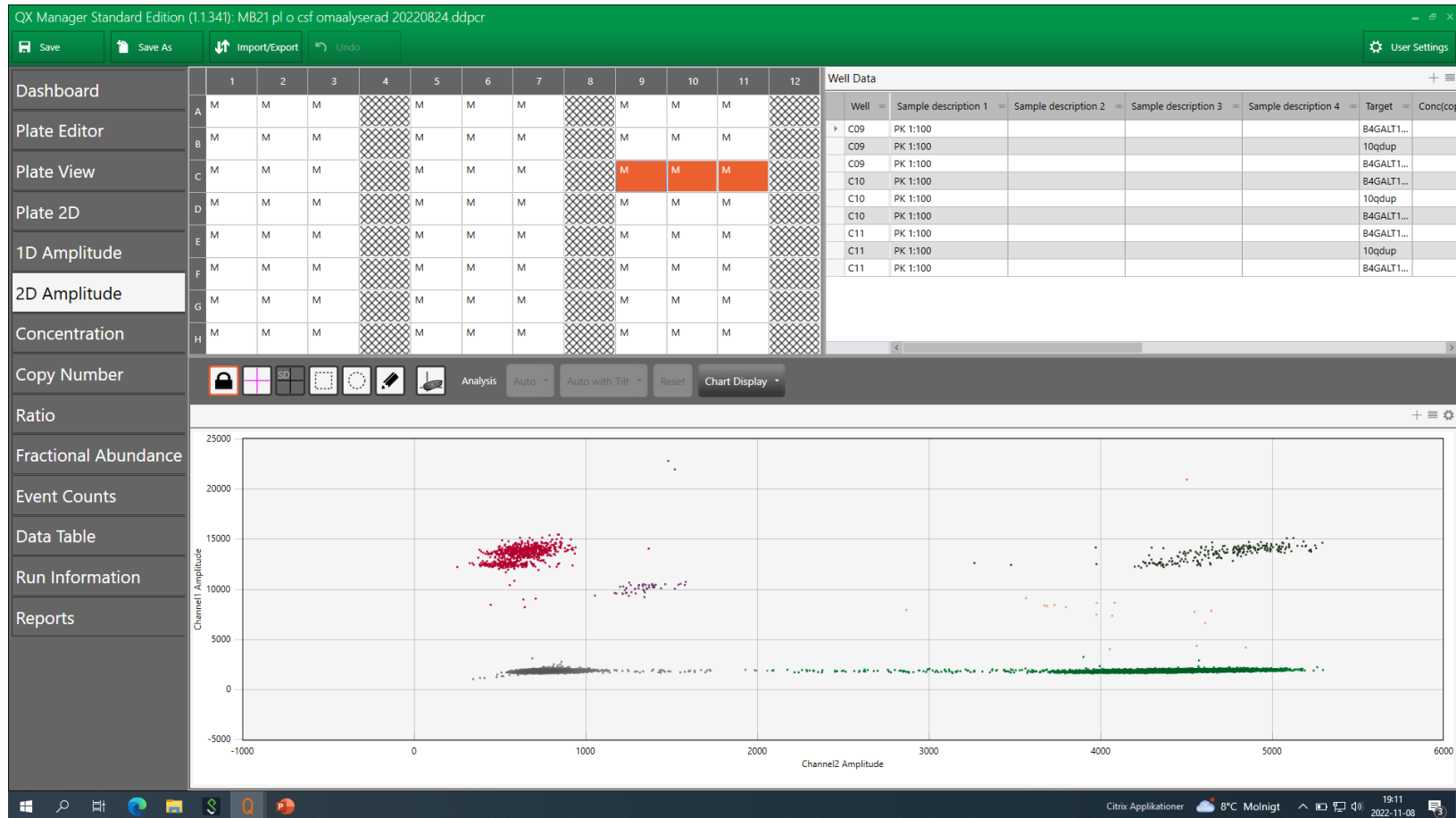

# MB21 1:1000

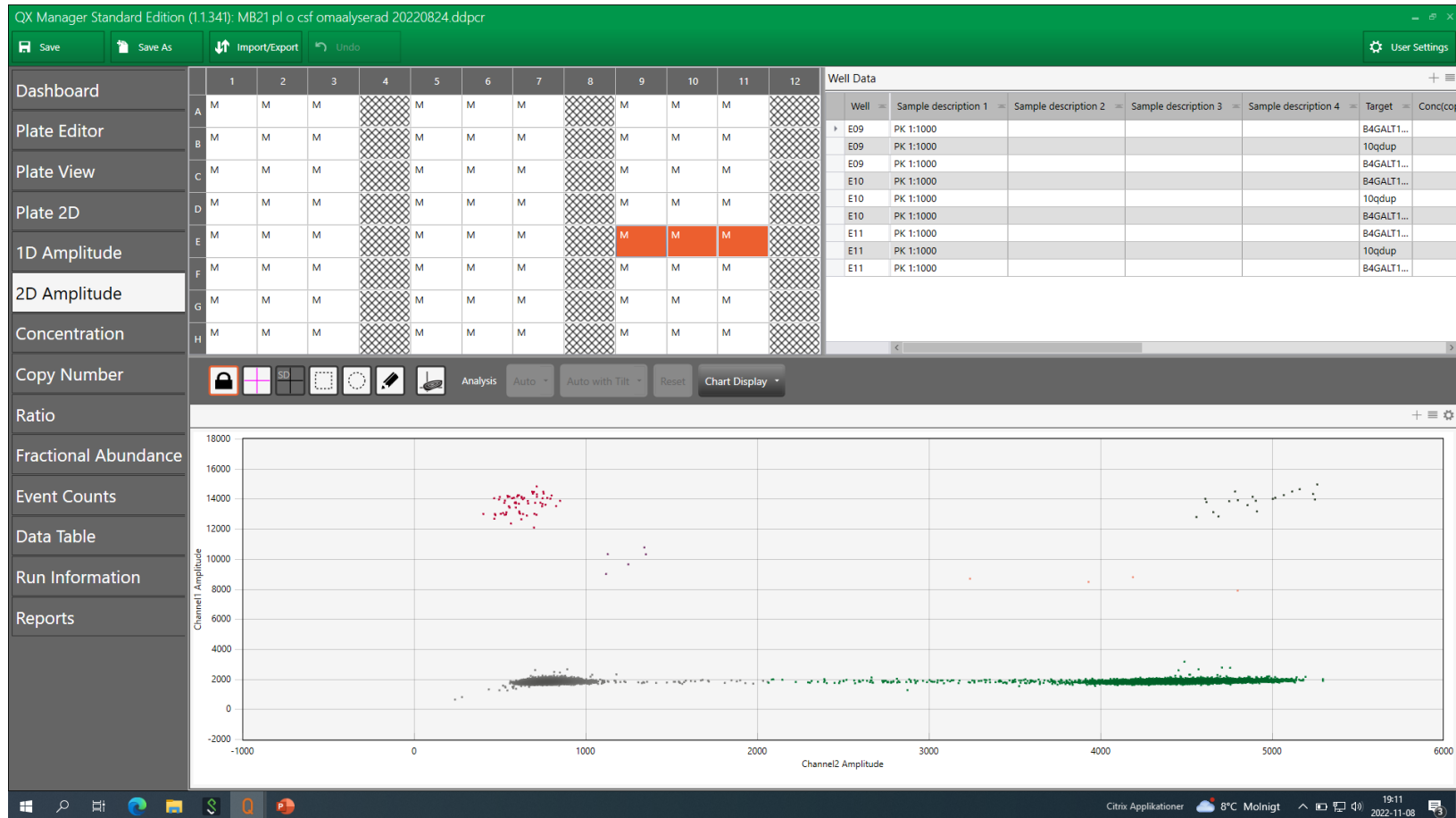

# MB21 1:10000

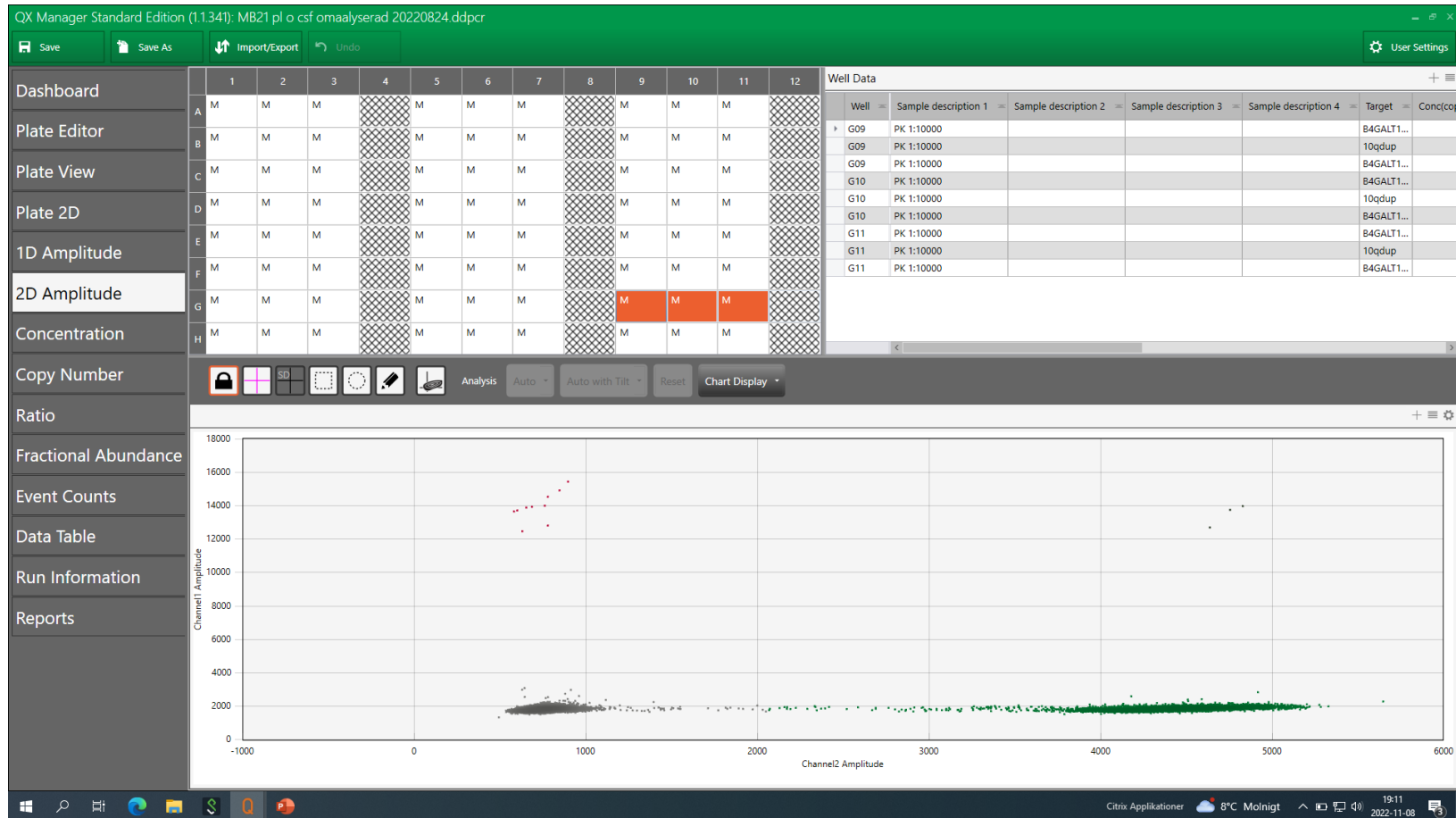

Supplement: Supplementary file 1 [file cancers-15-01972-s001.zip › File S1 QX Manager Software output data on dilution series/Dilution series MB21.pdf]

# MB22 Concentration

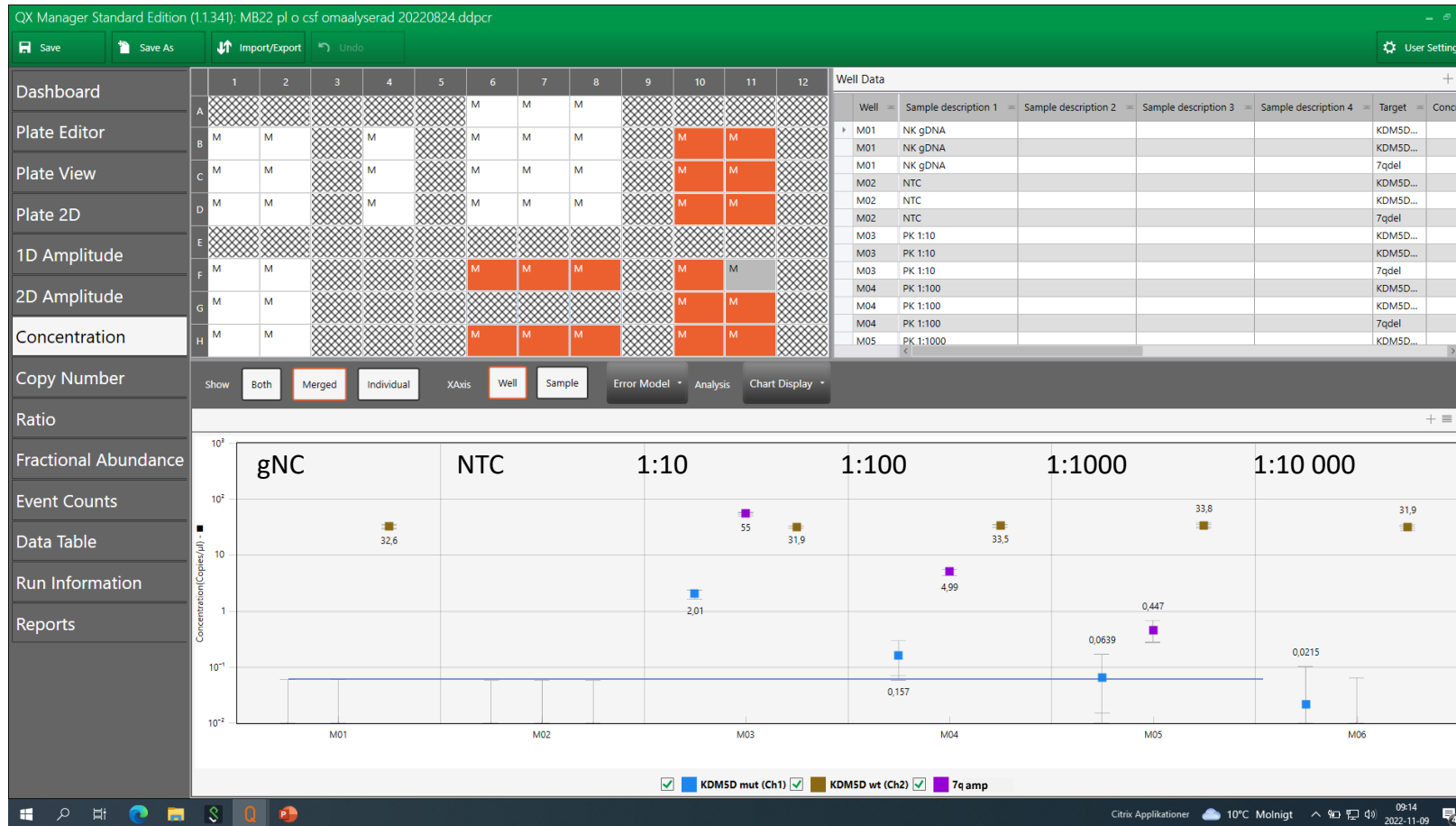

# MB22 NTC

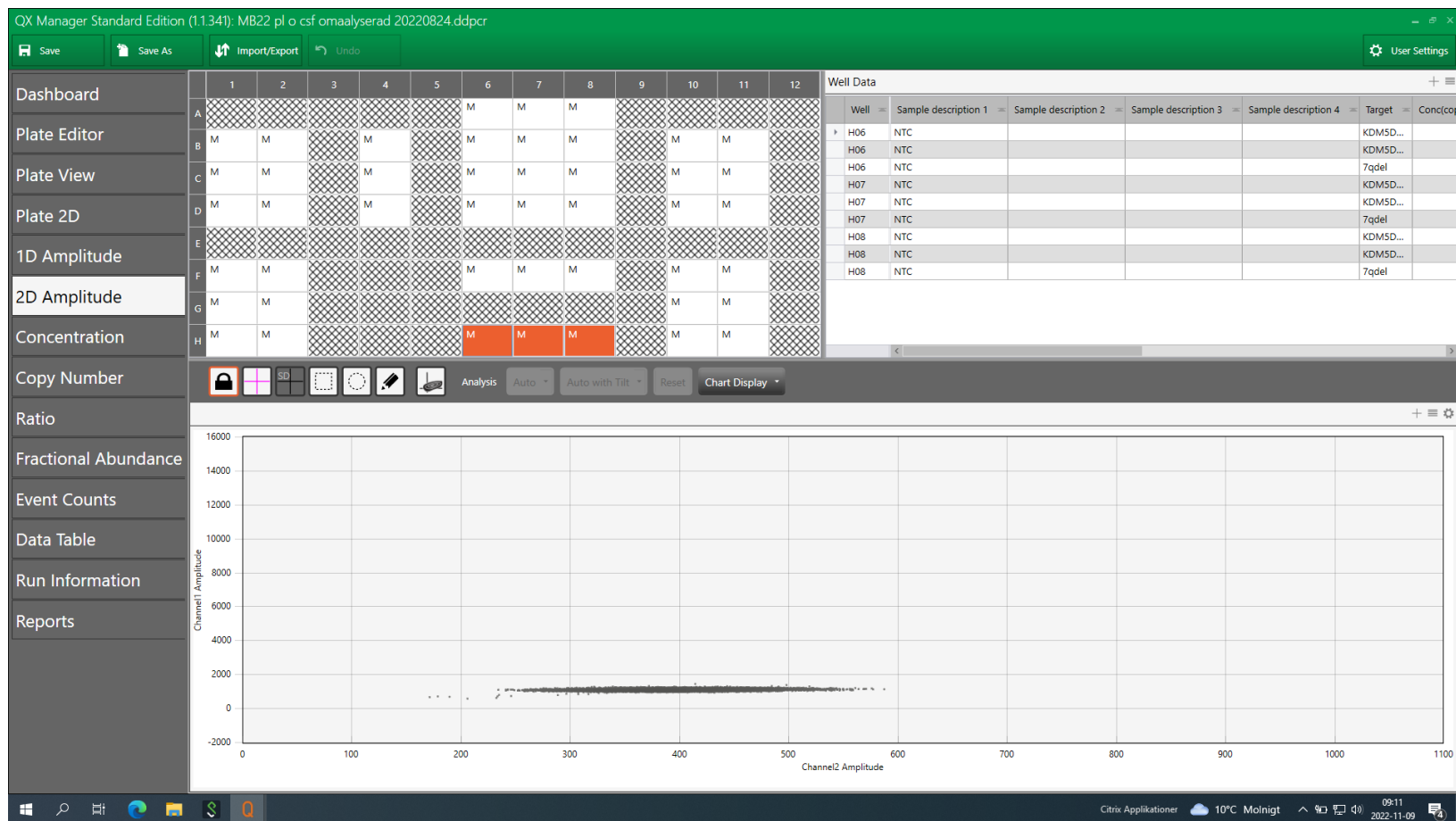

# MB22 gNC

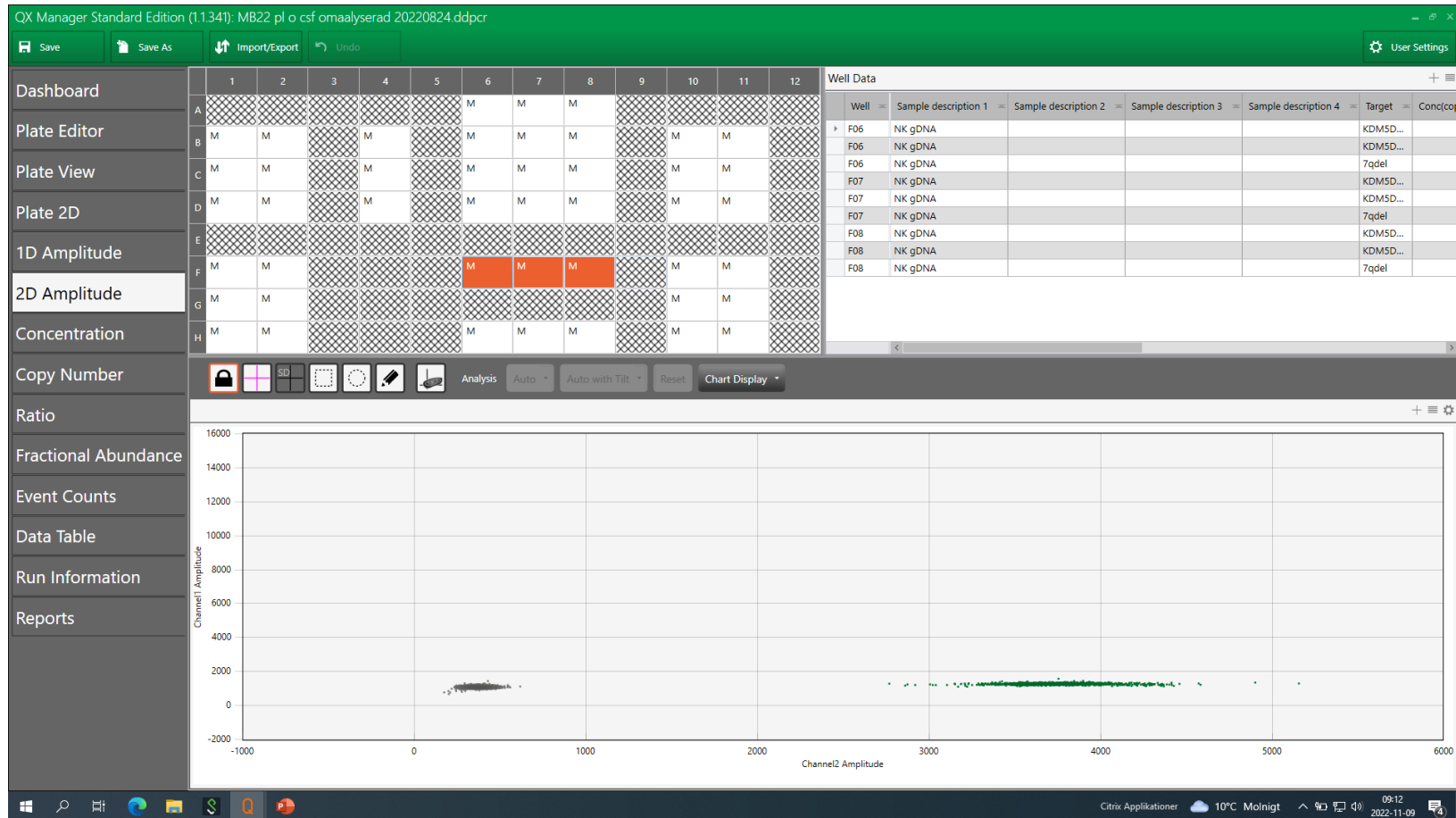

# MB22 Example of clusters

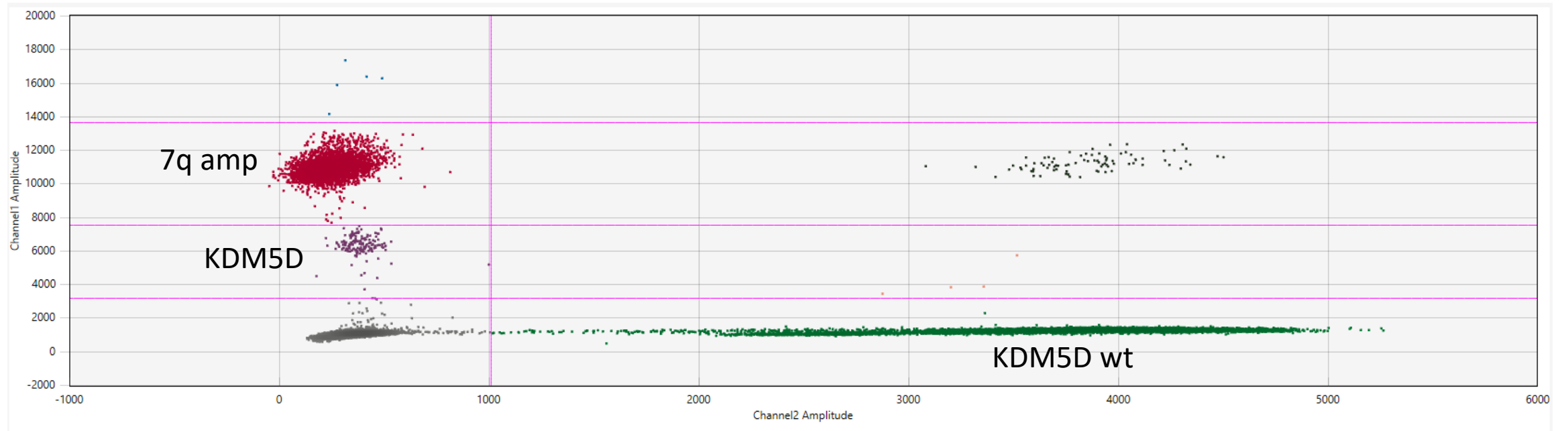

# MB22 1:10

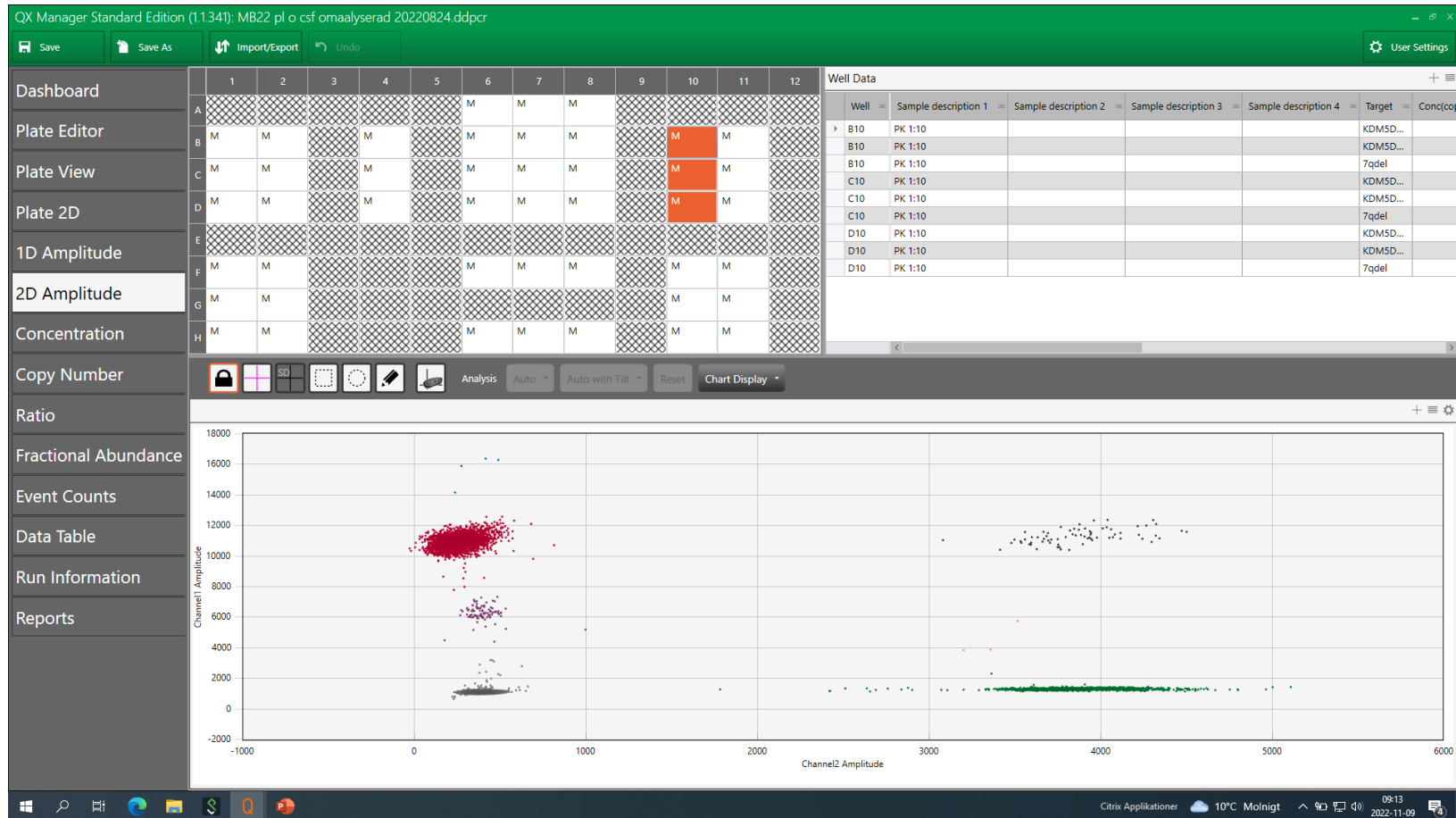

# MB22 1:100

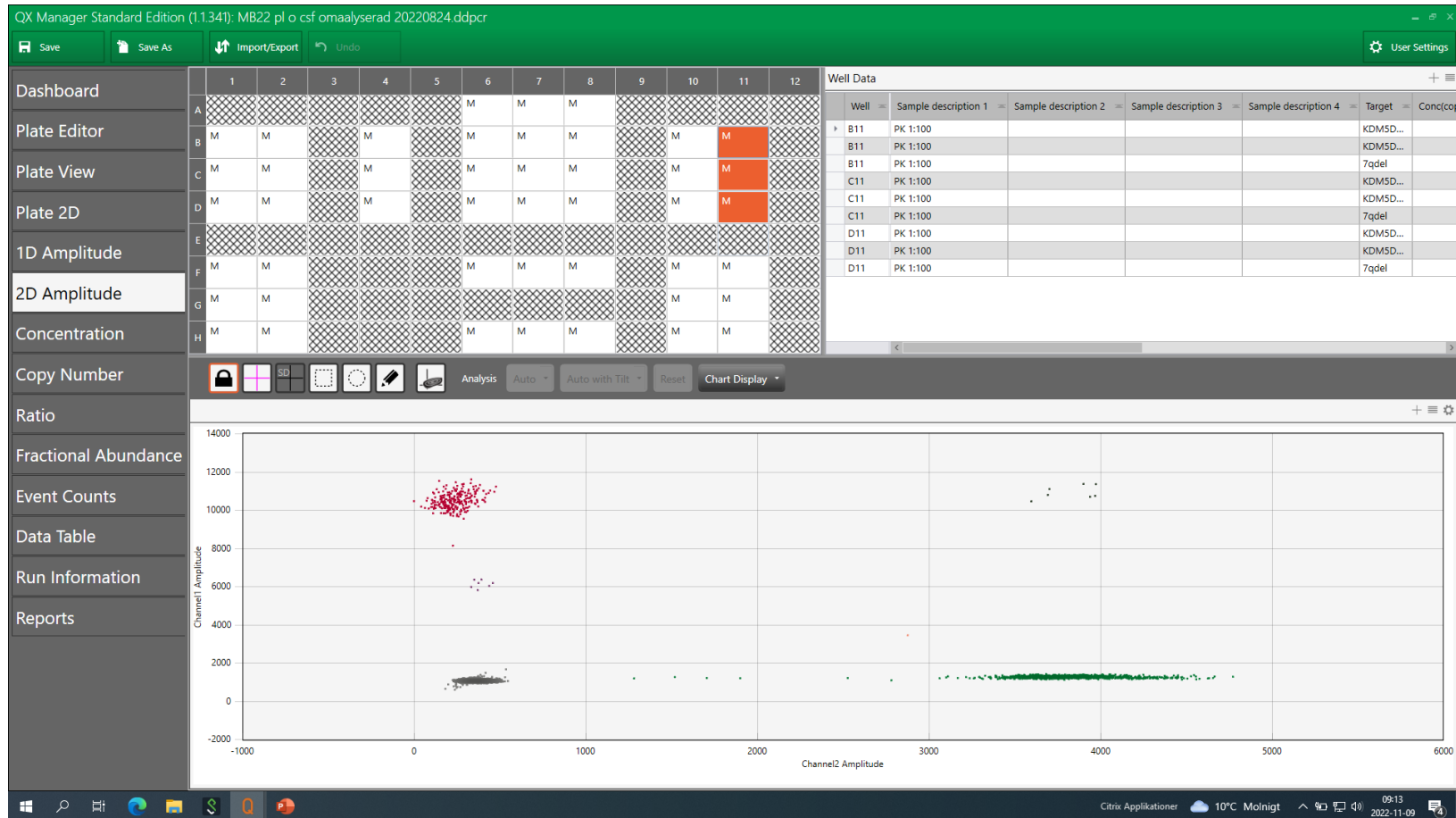

# MB22 1:1000

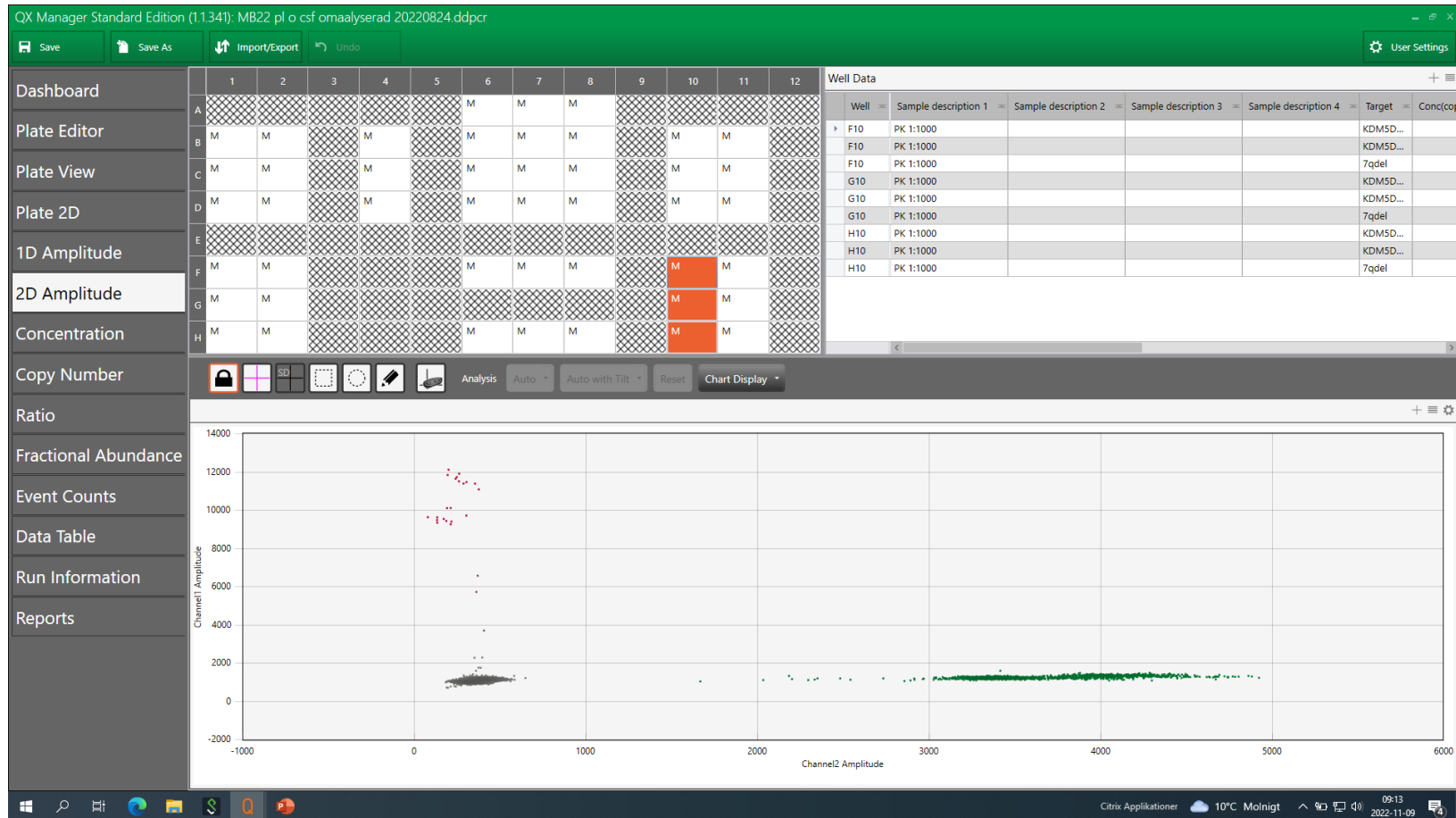

# MB22 1:10000

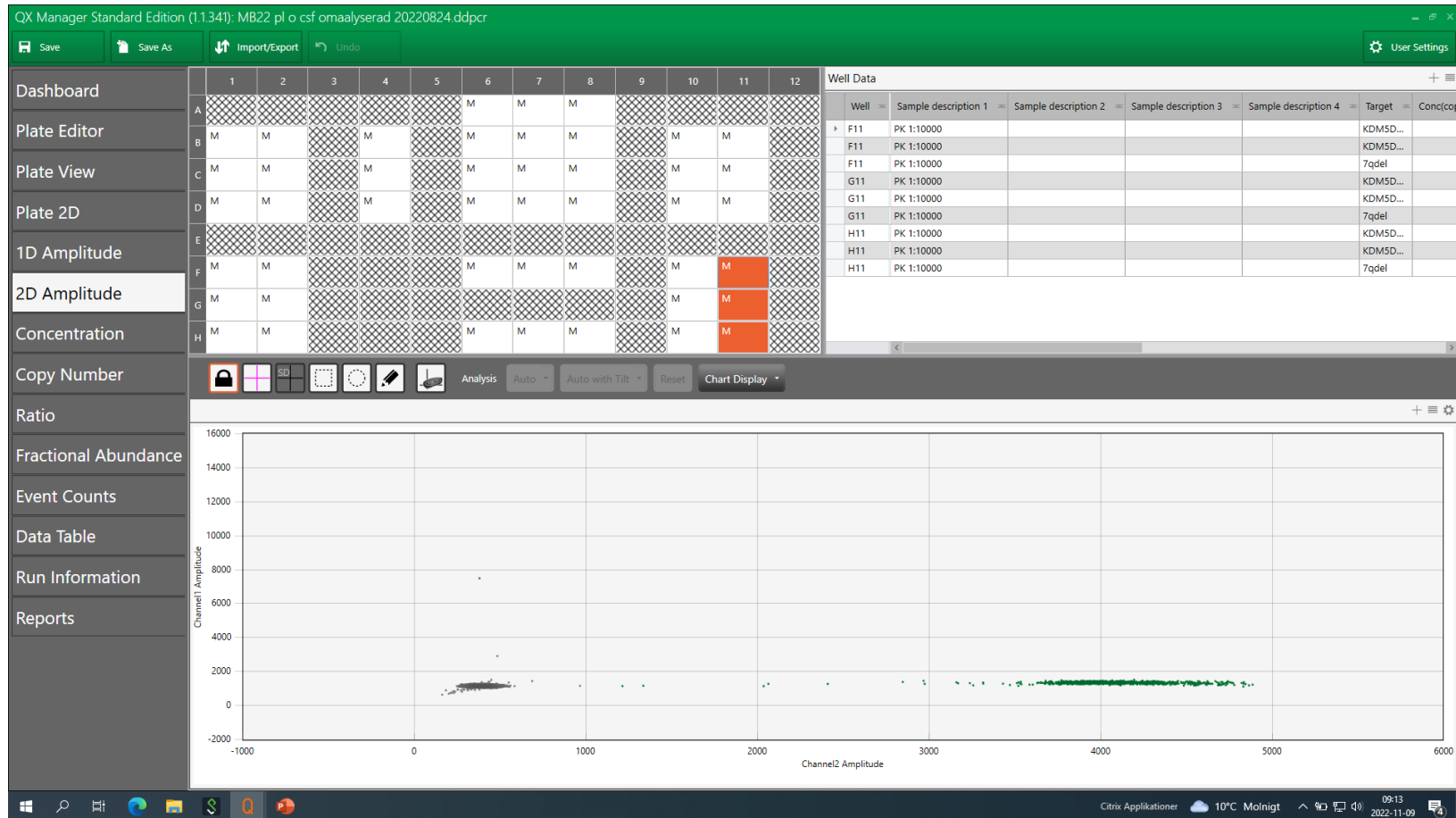

Supplement: Supplementary file 1 [file cancers-15-01972-s001.zip › File S1 QX Manager Software output data on dilution series/Dilution series MB22.pdf]

# MB24 Concentration plot

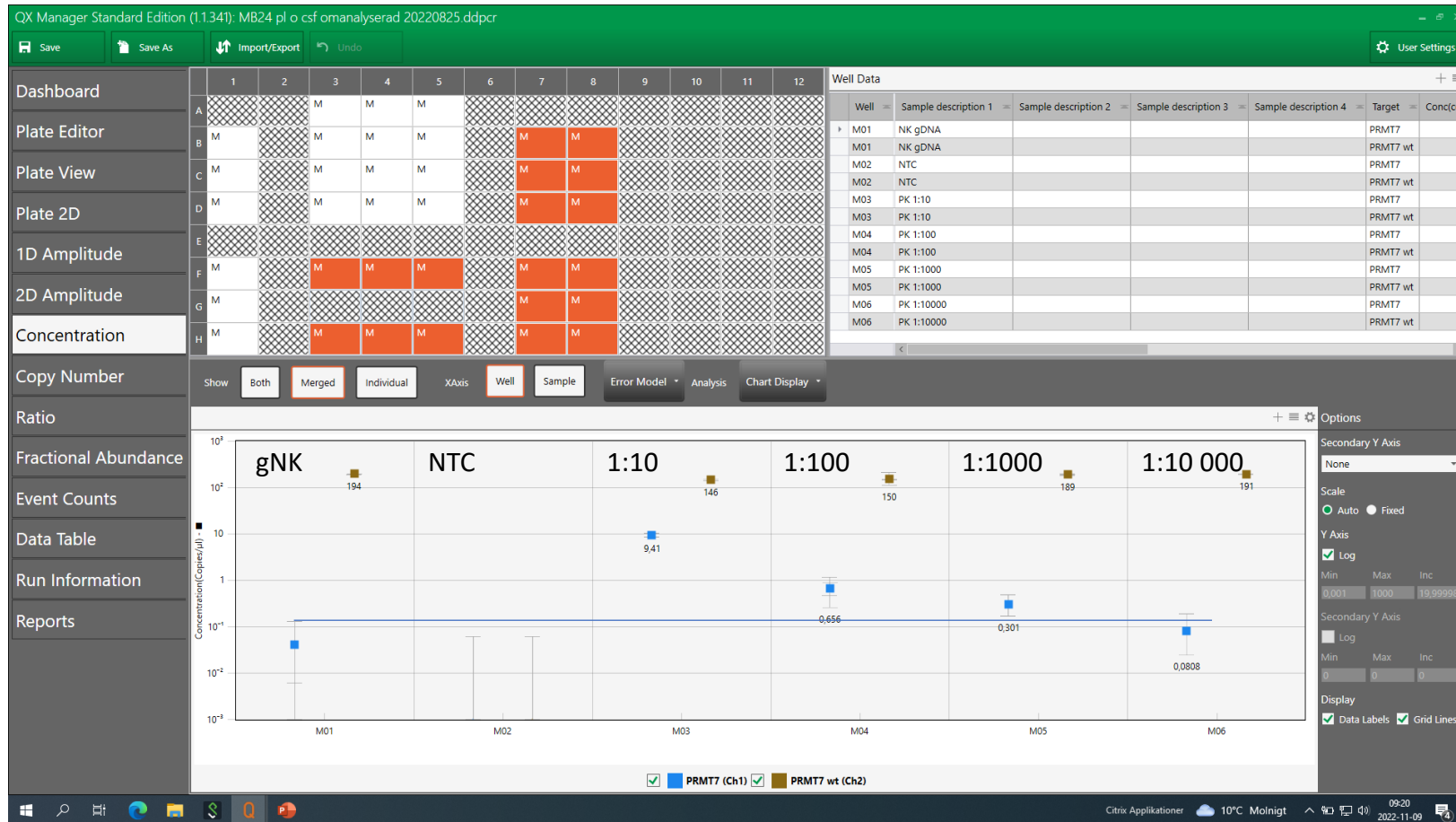

# MB24 NTC

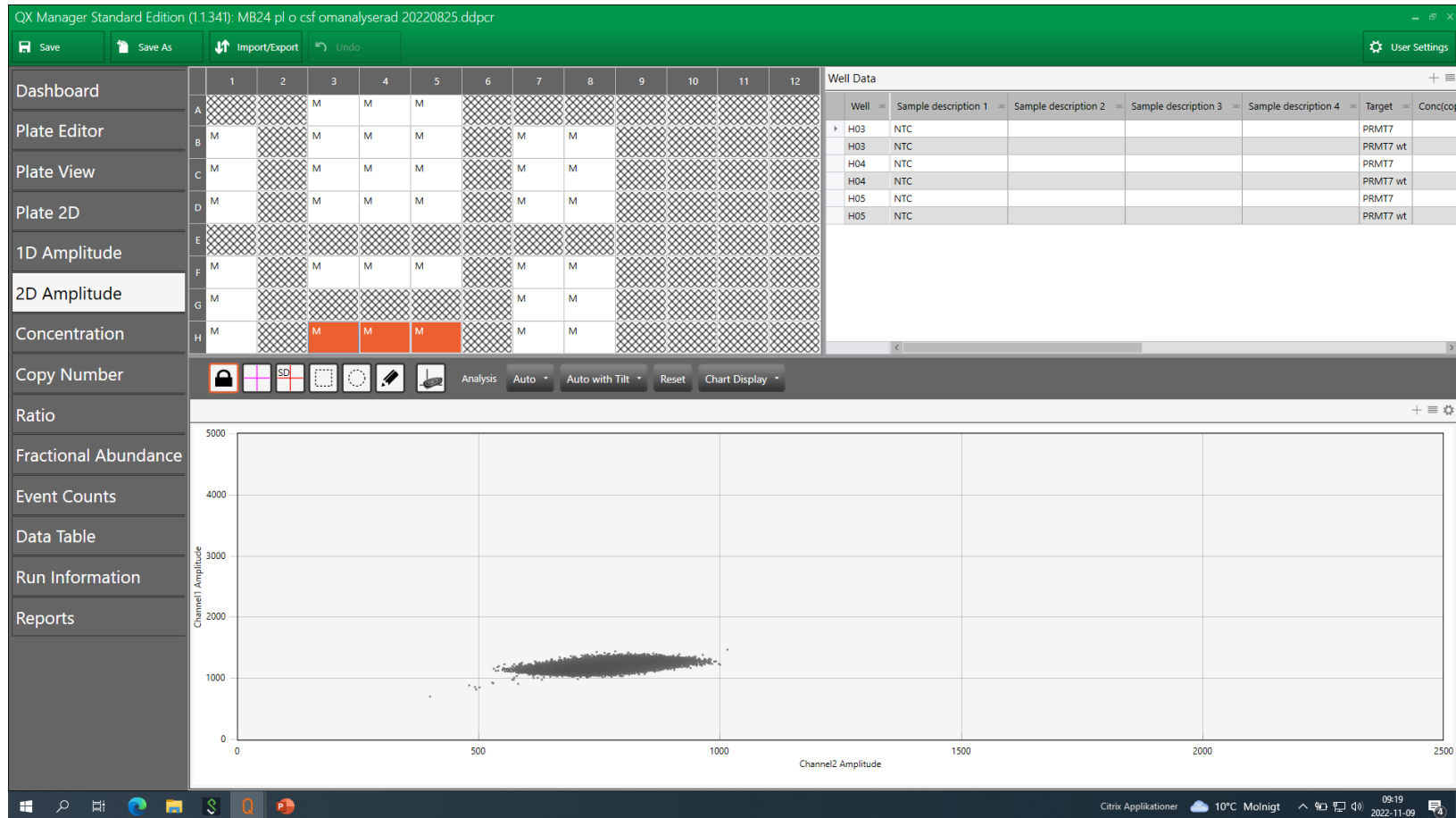

# MB24 gNC

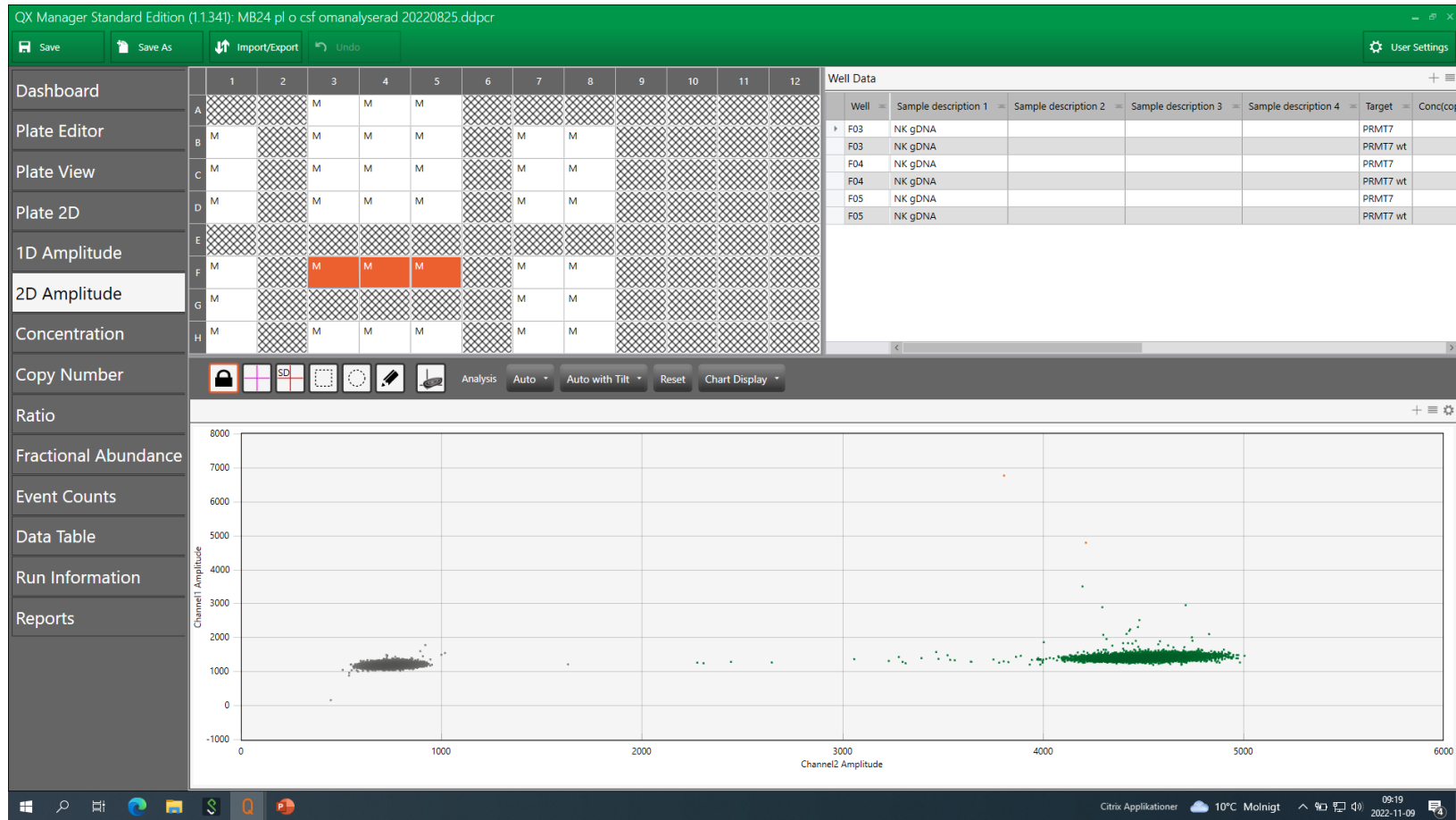

# MB24 Example of clusters

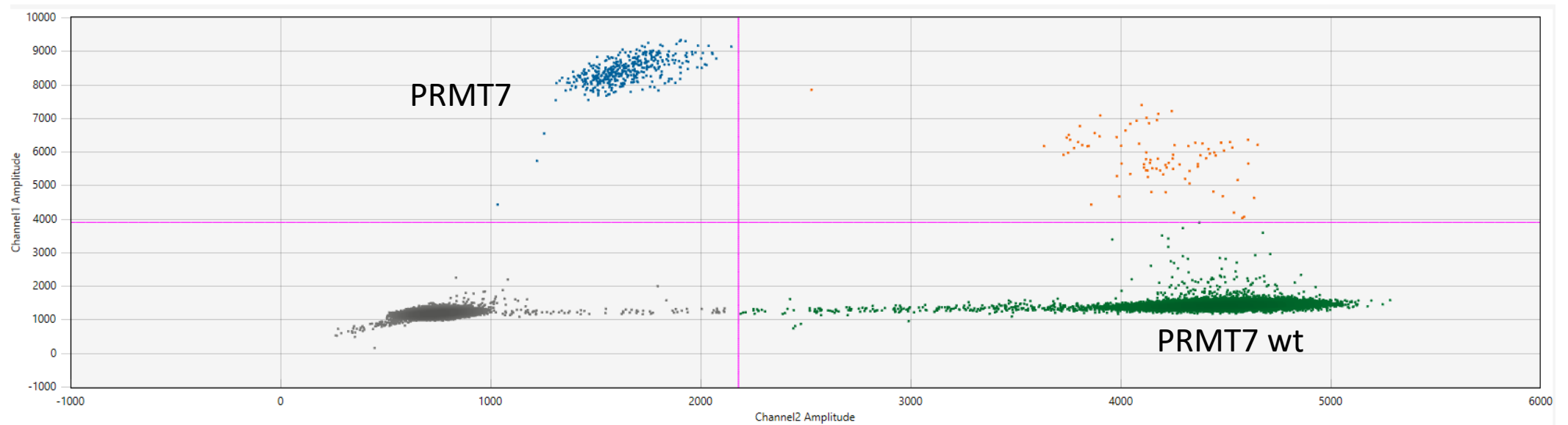

# MB24 1:10

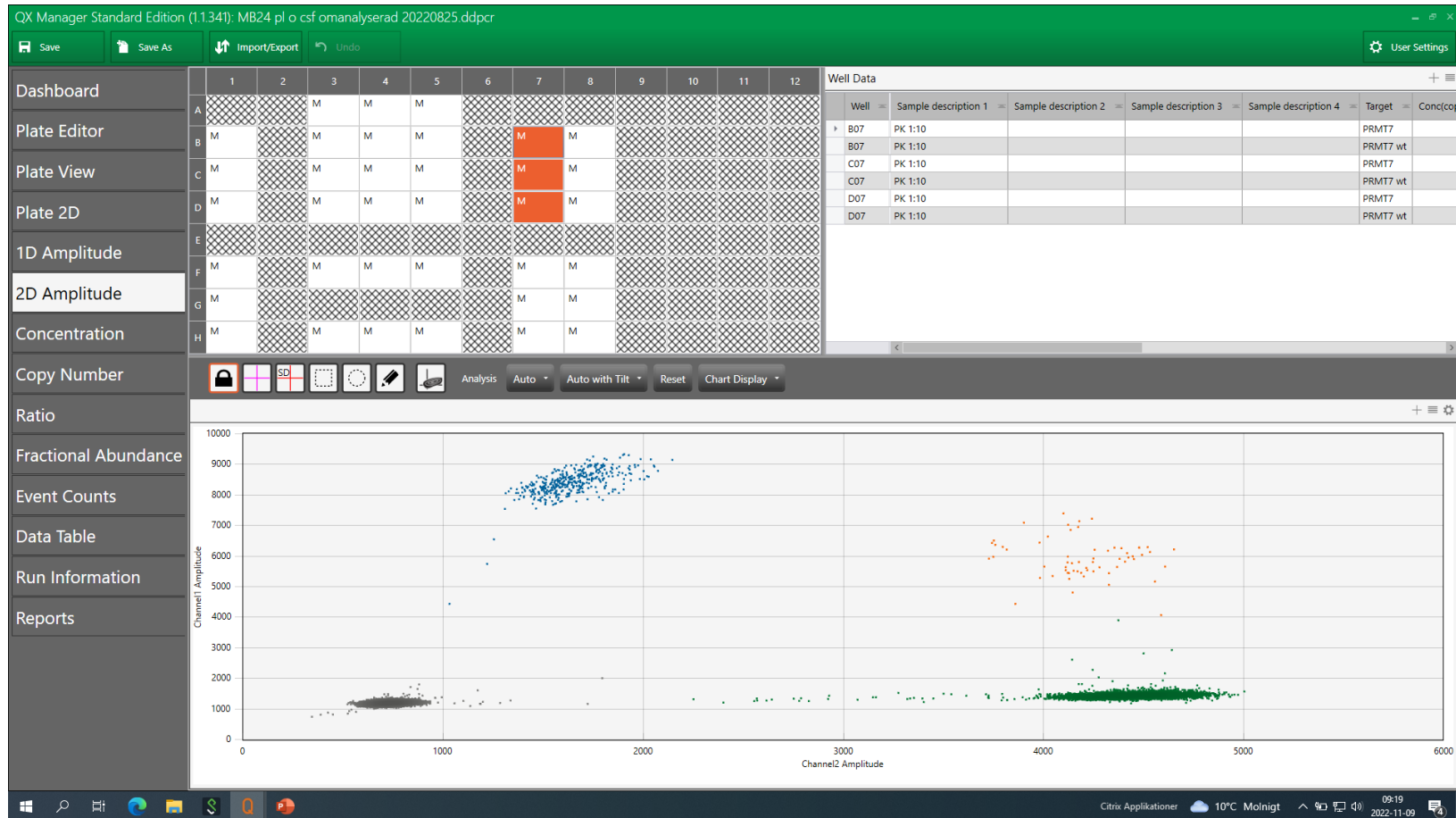

# MB24 1:100

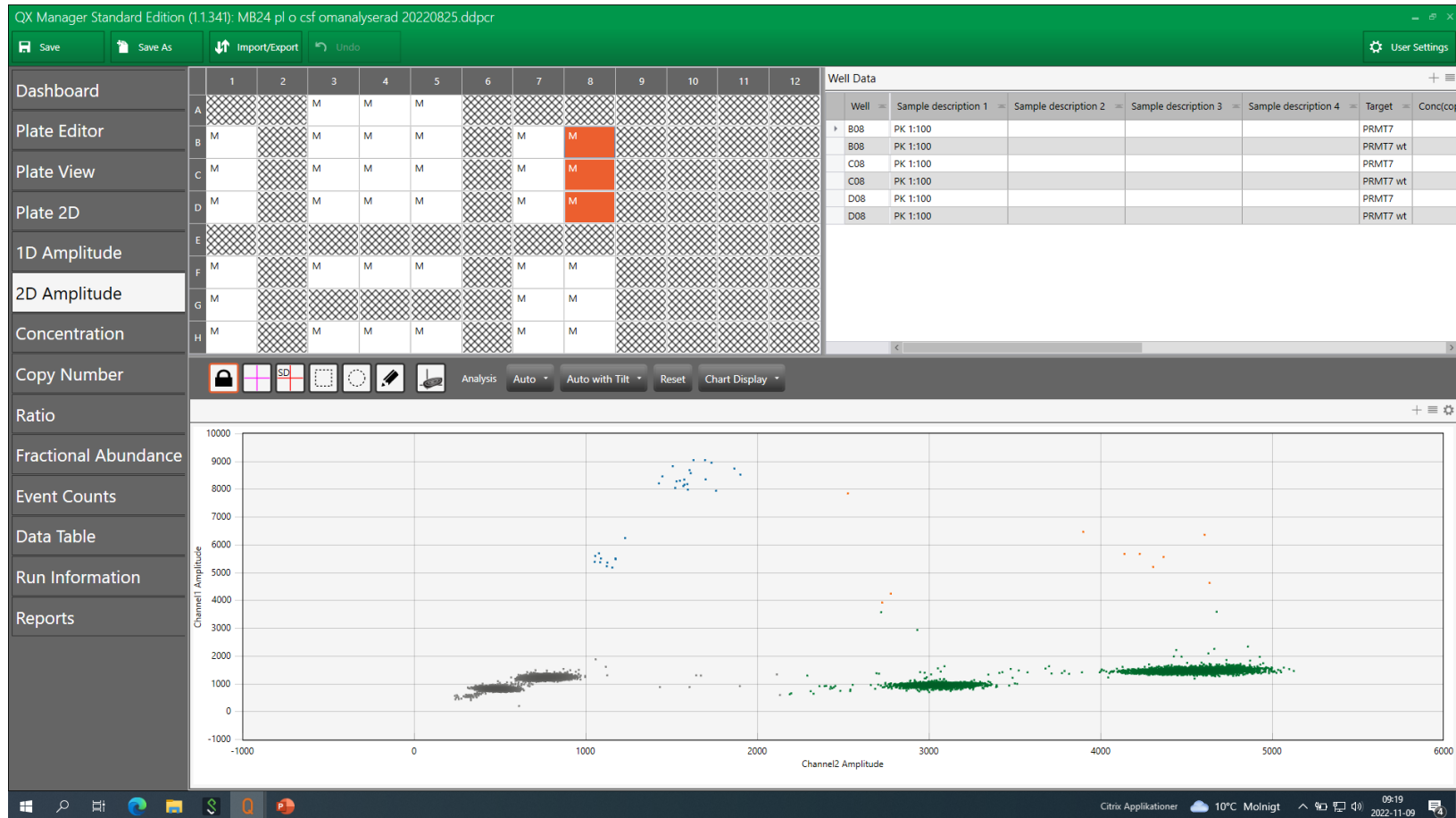

# MB24 1:1000

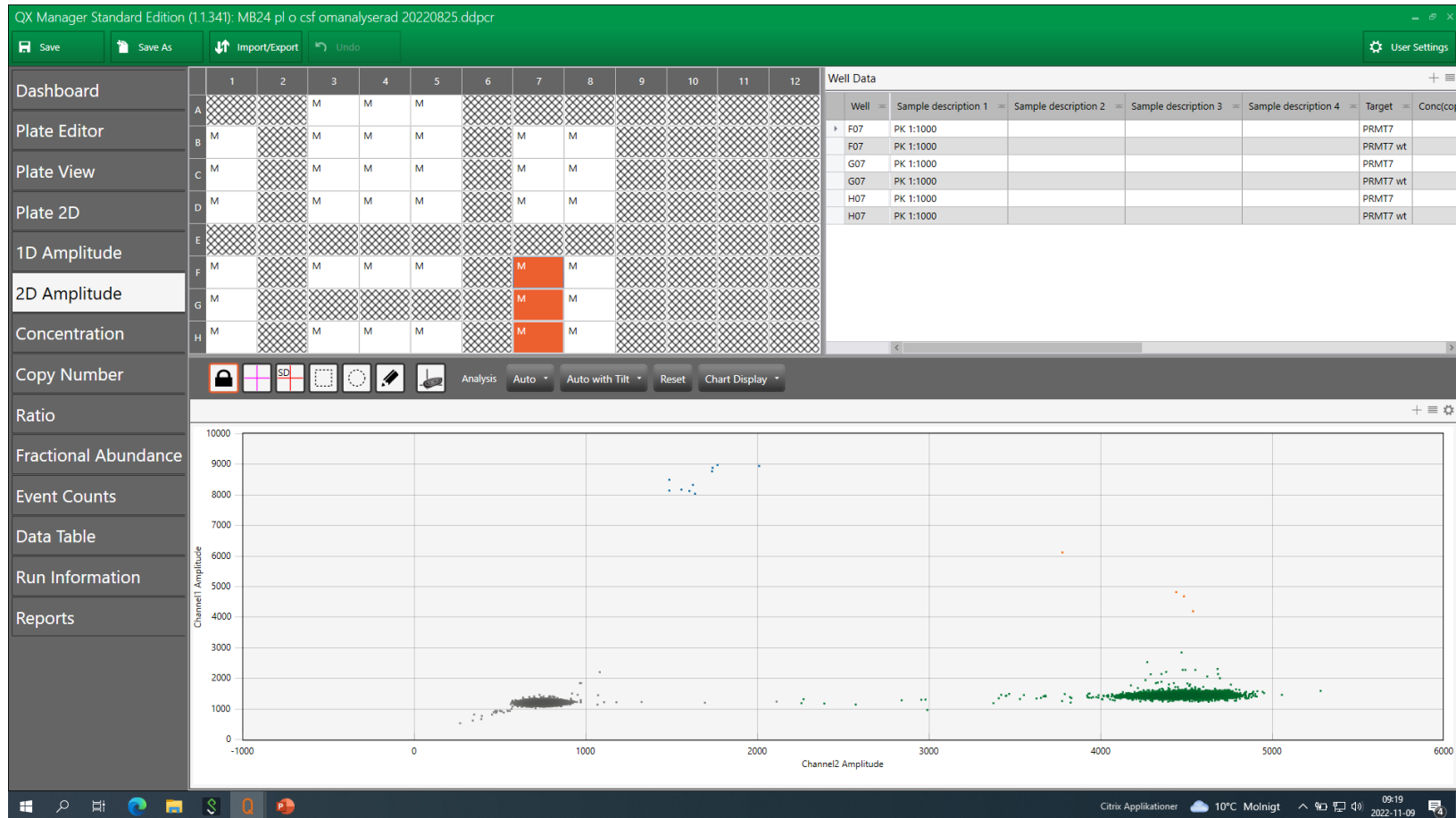

# MB24 1:10000

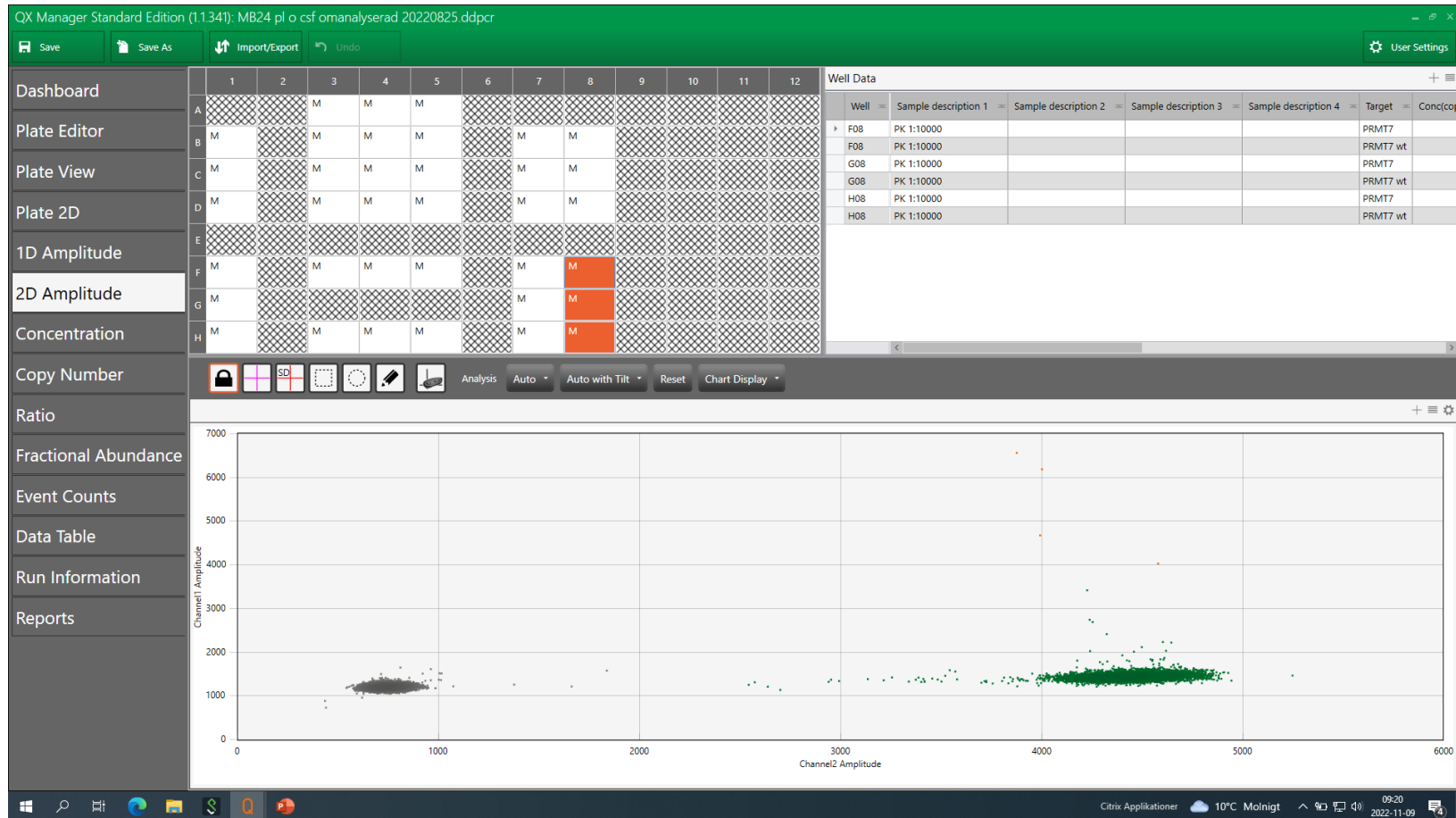

Supplement: Supplementary file 1 [file cancers-15-01972-s001.zip › File S1 QX Manager Software output data on dilution series/Dilution series MB24.pdf]

# MB27 Concentration plot

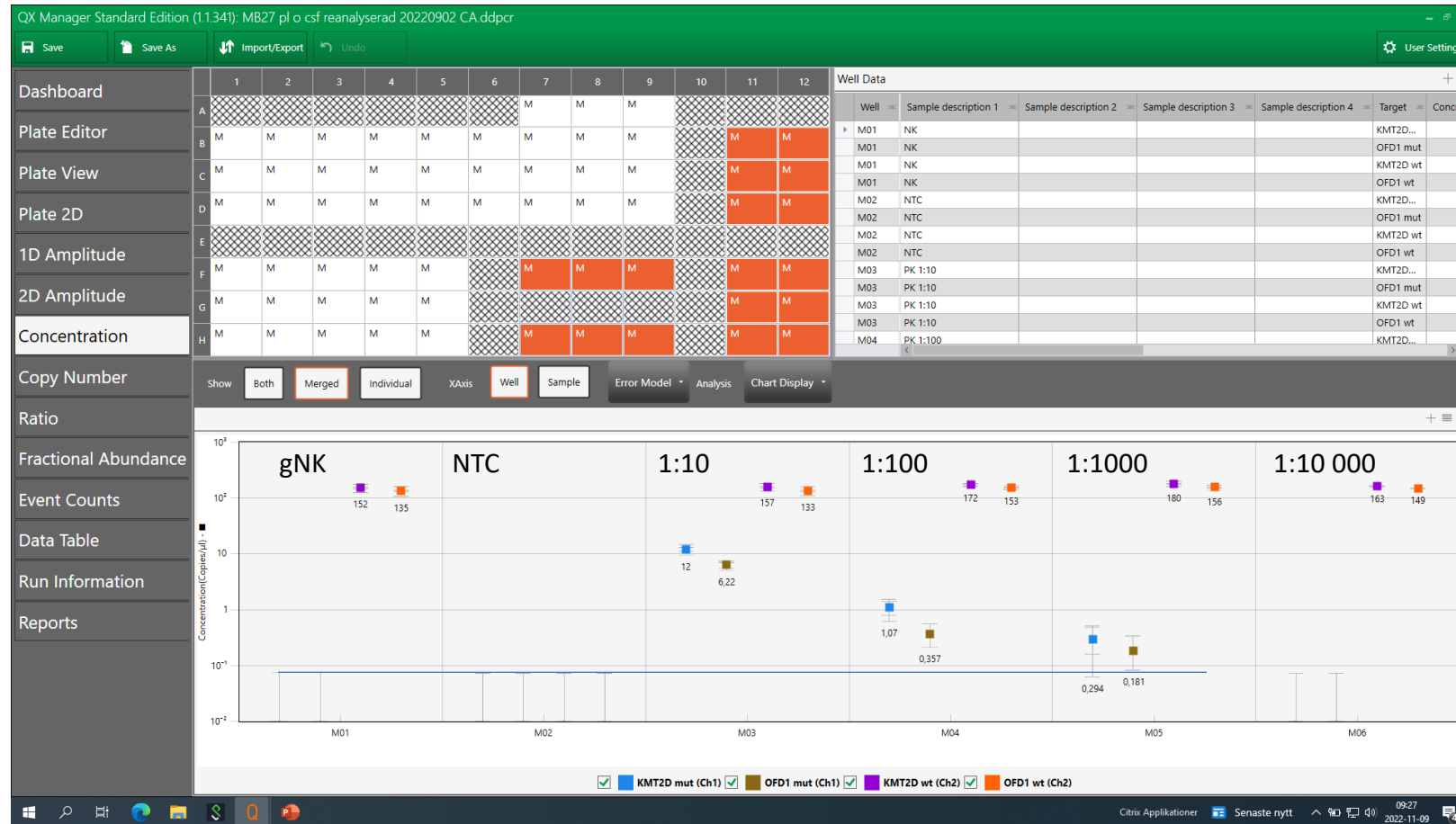

# MB27 NTC

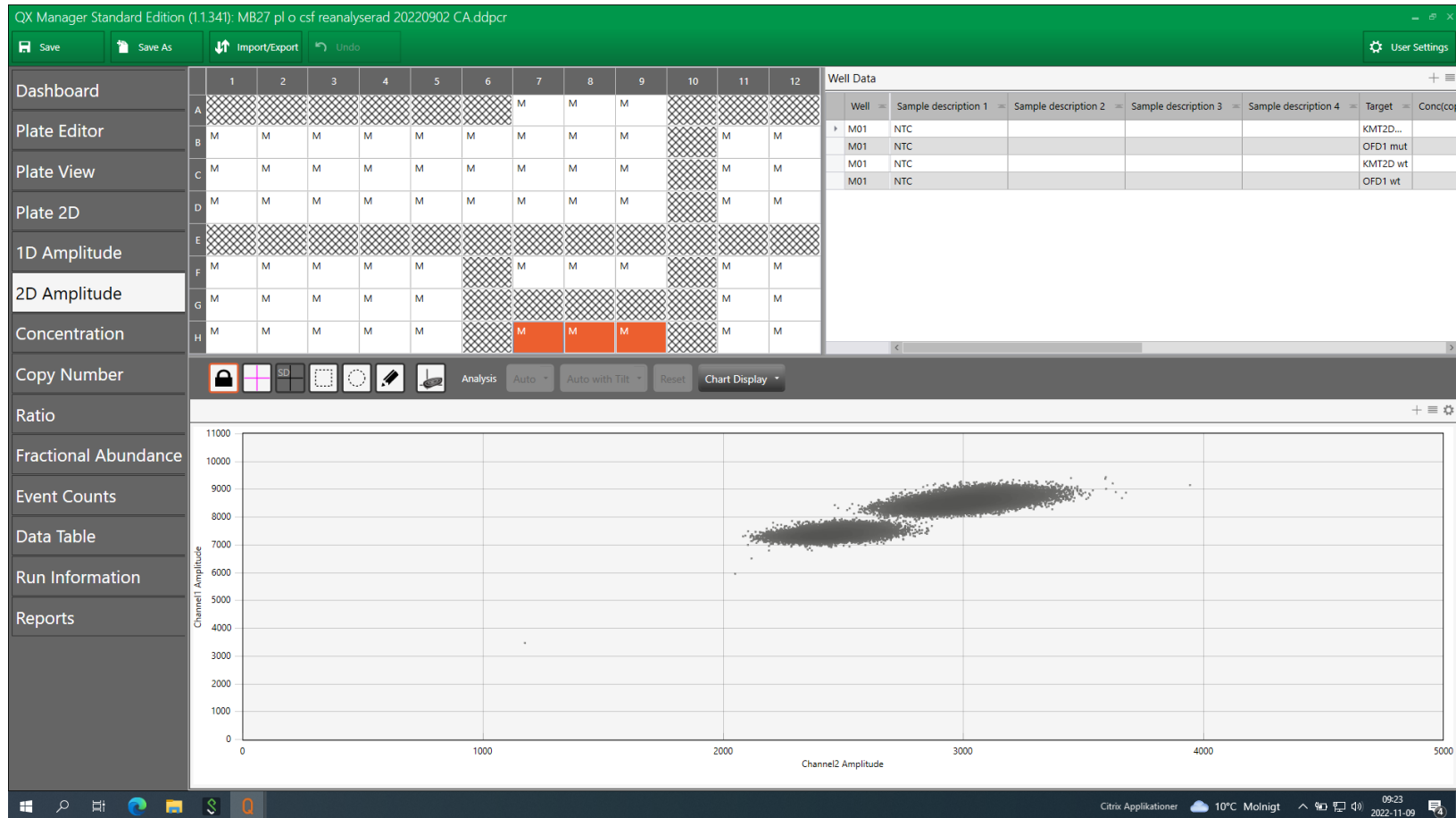

# MB27 gNC

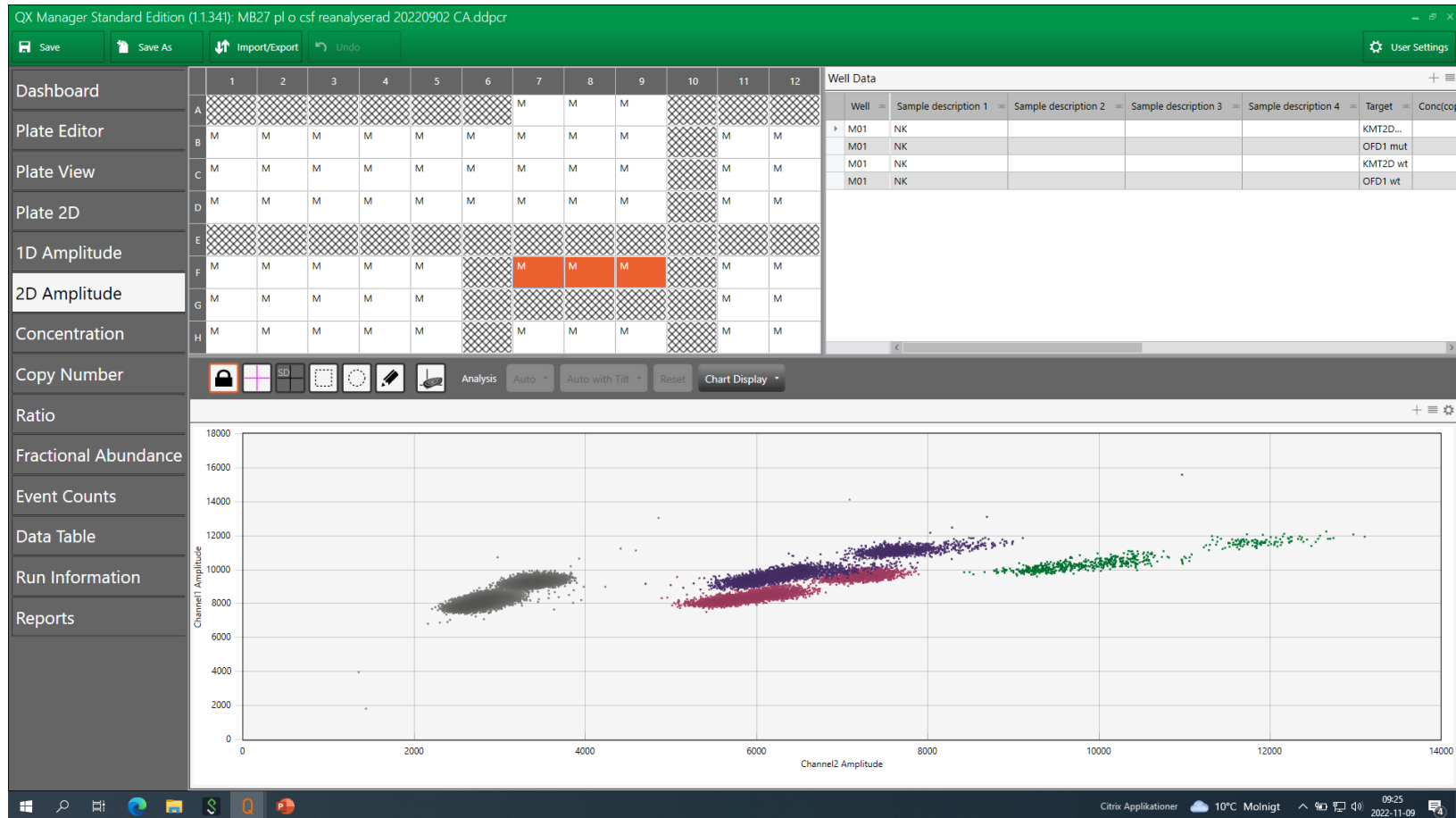

# MB27 Example of clusters

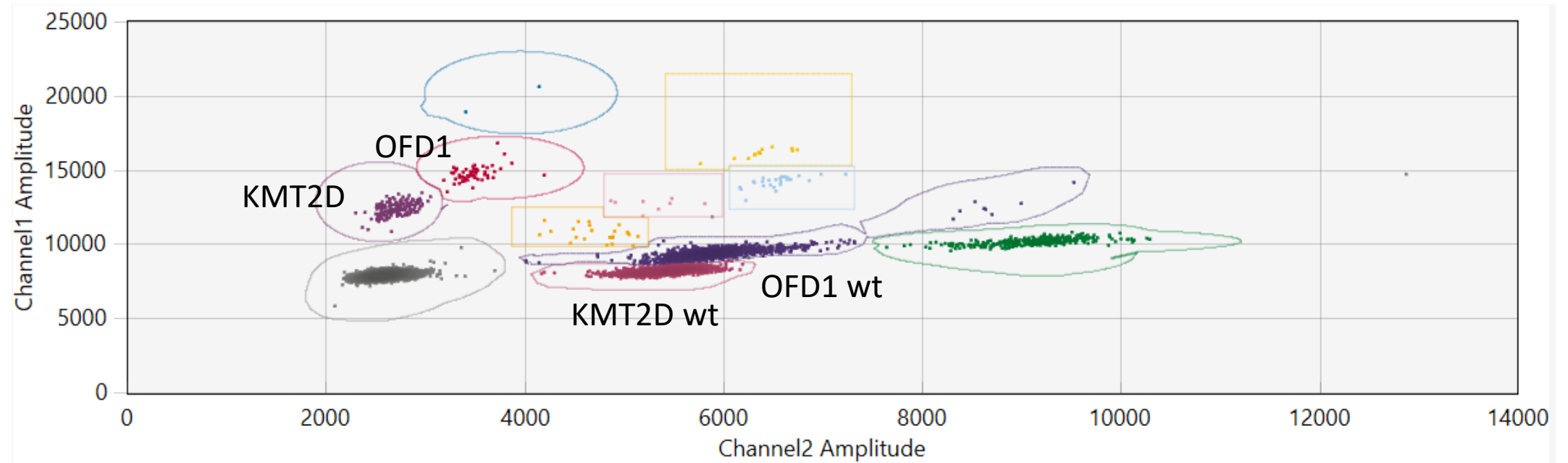

# MB27 1:10

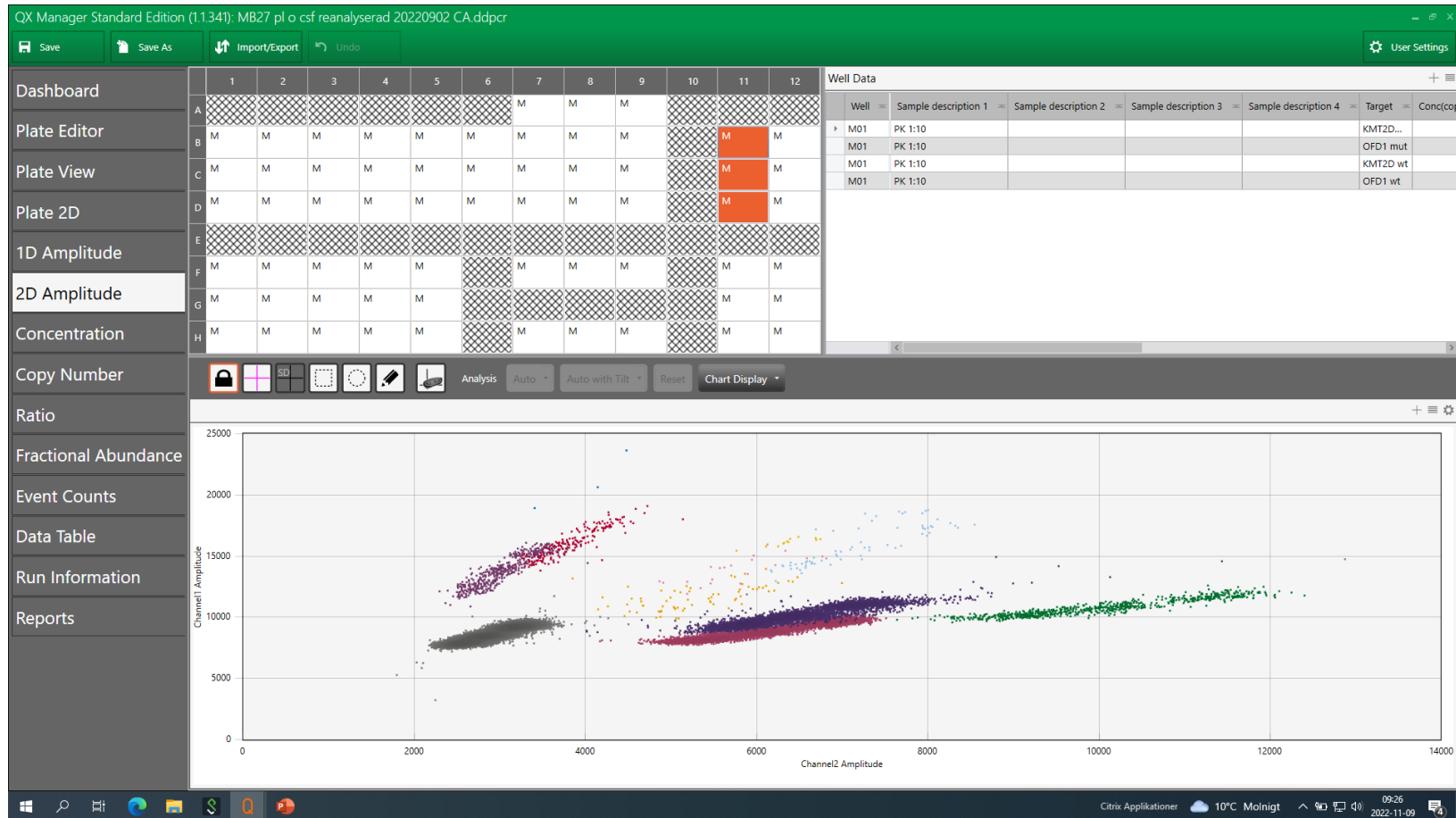

# MB27 1:100

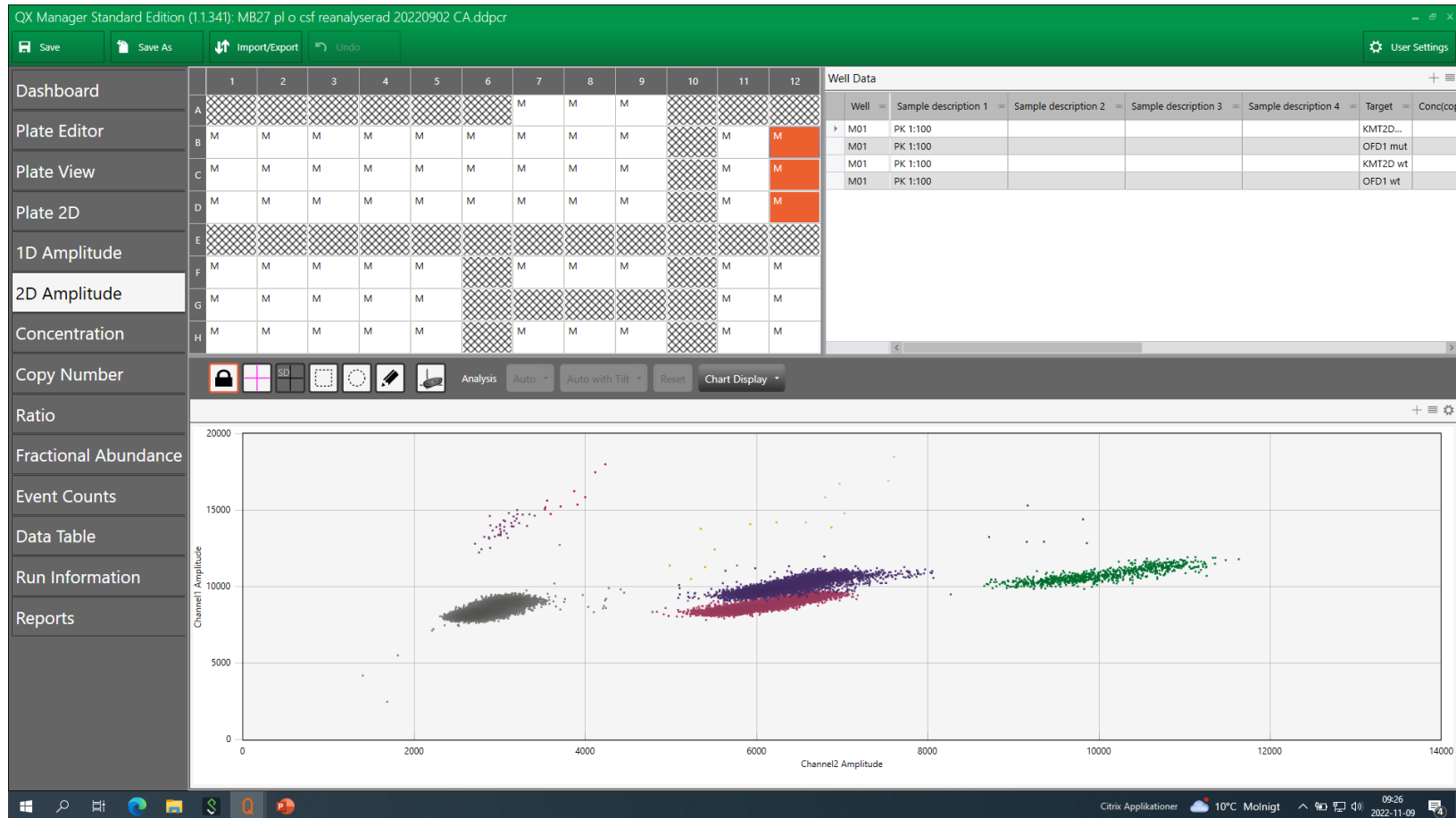

# MB27 1:1000

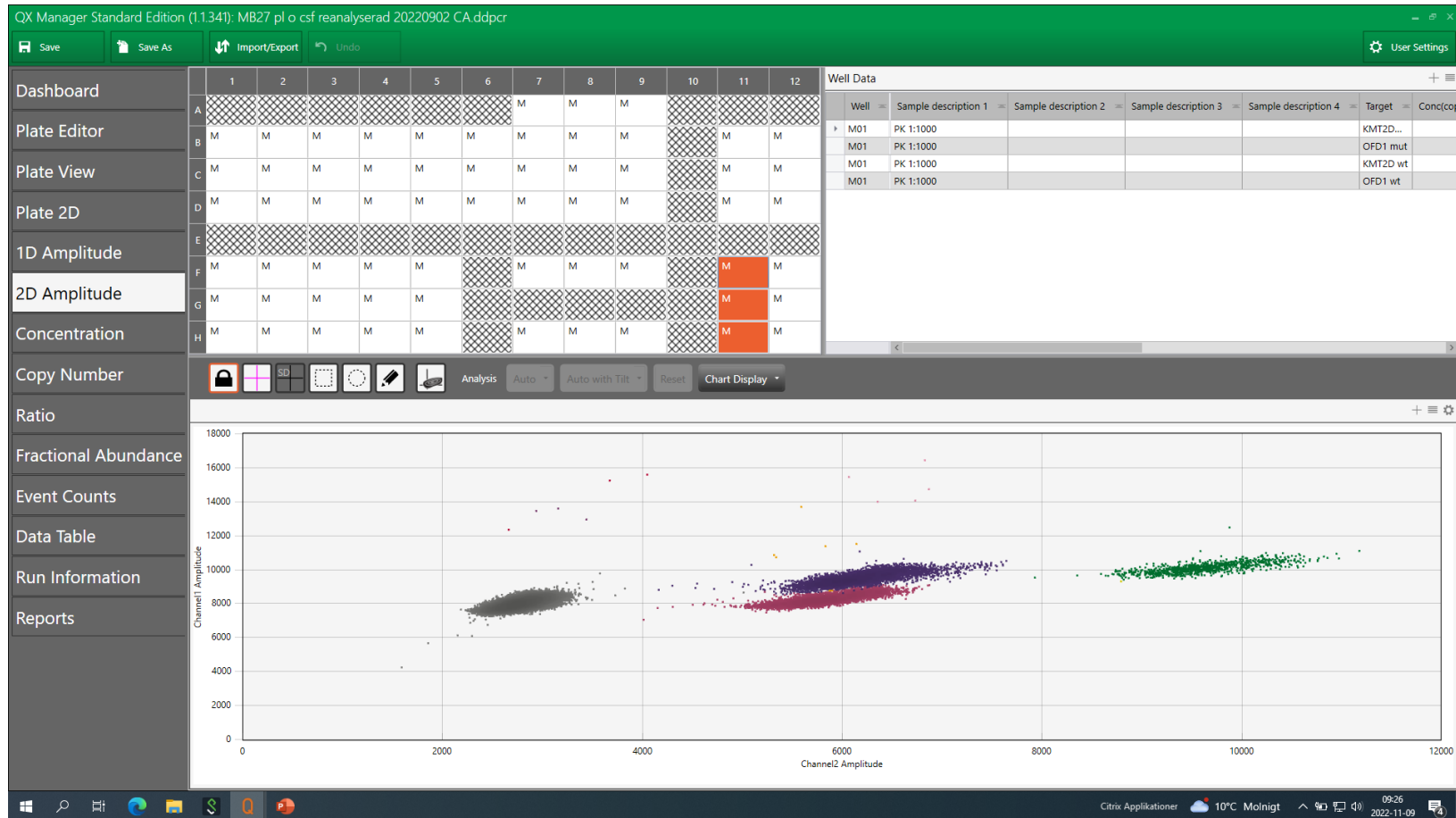

# MB27 1:10000

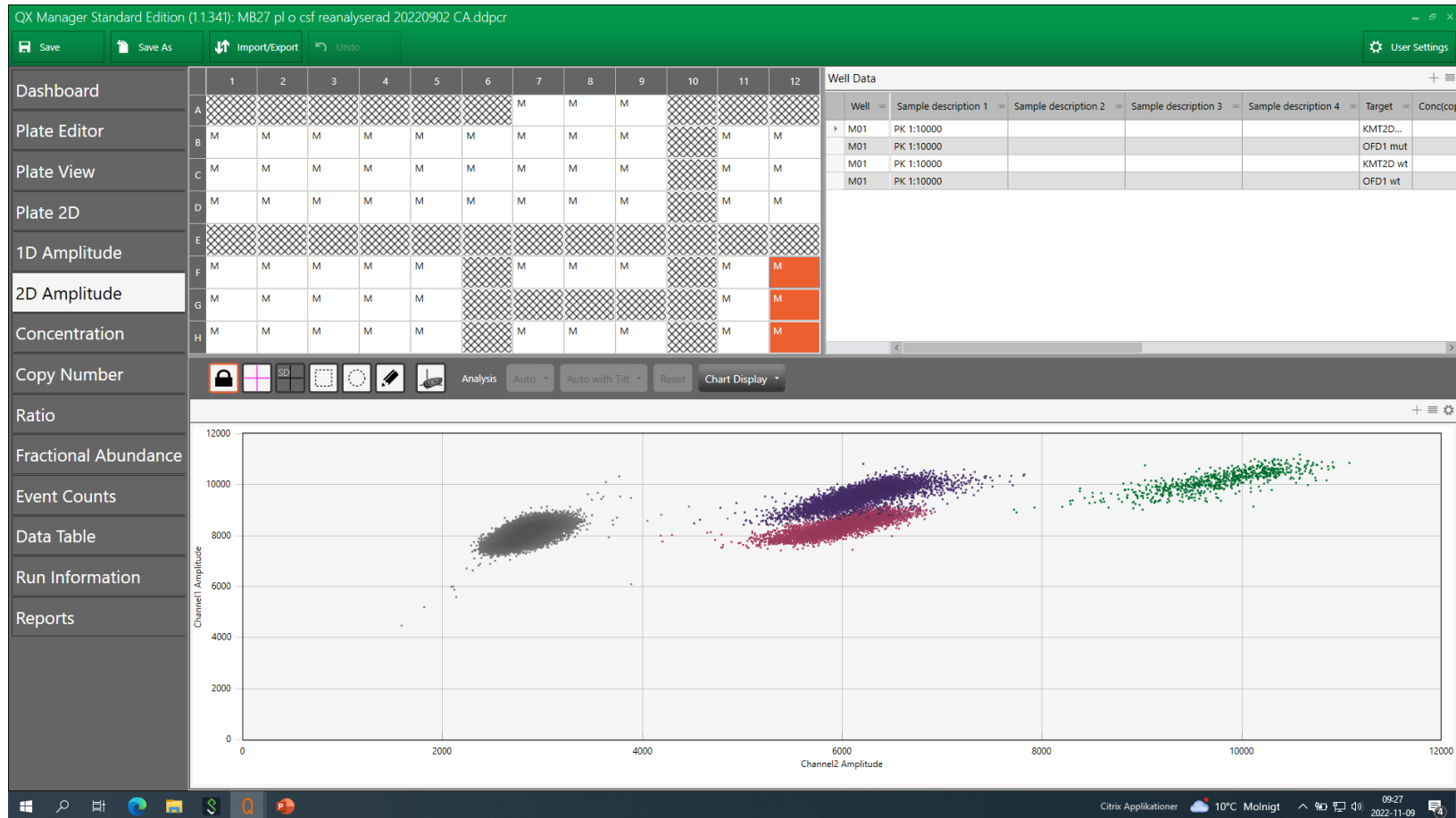

Supplement: Supplementary file 1 [file cancers-15-01972-s001.zip › File S1 QX Manager Software output data on dilution series/Dilution series MB27.pdf]

# MB31 Concentration

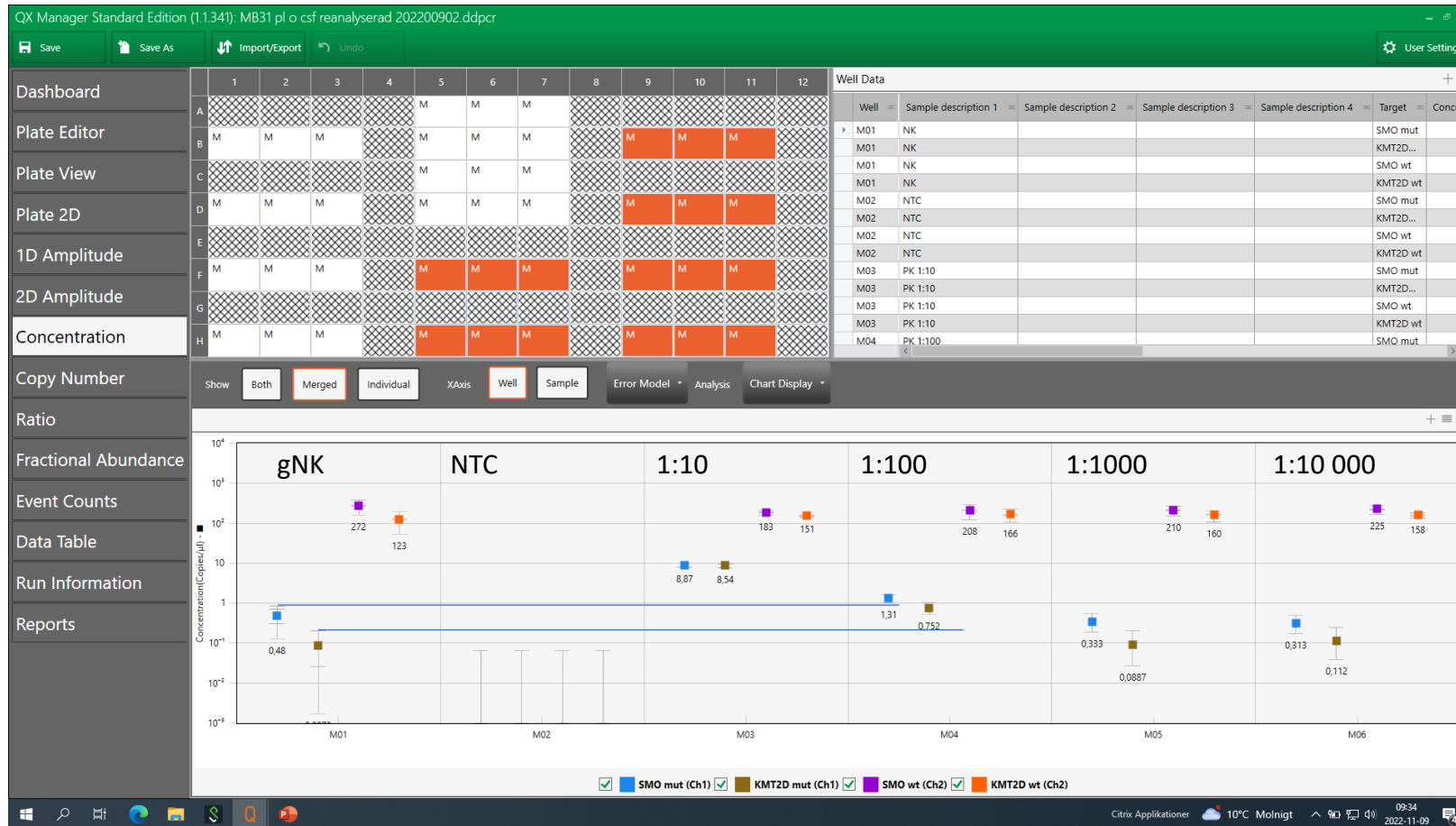

# MB31 NTC

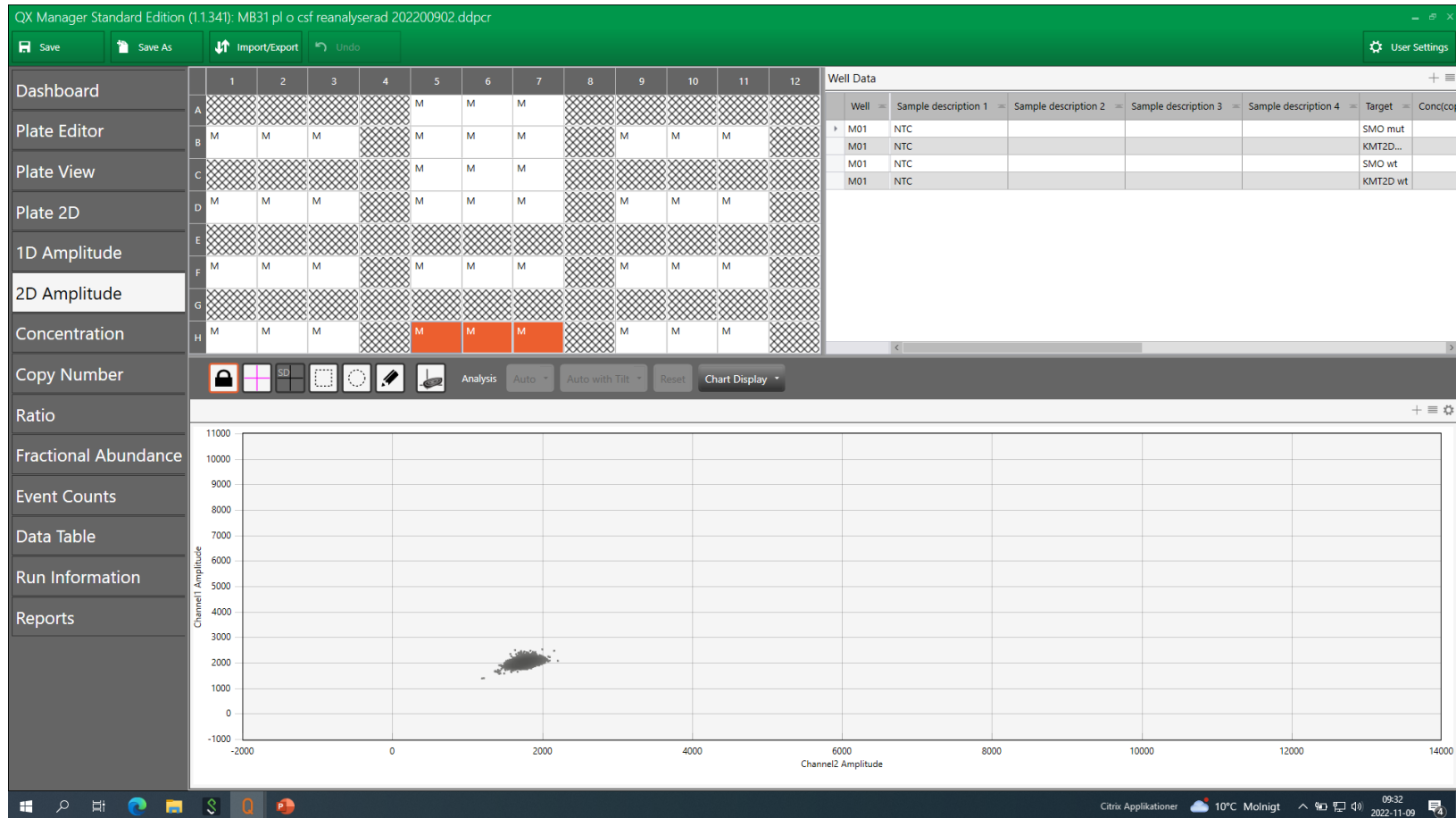

# MB31 gNC

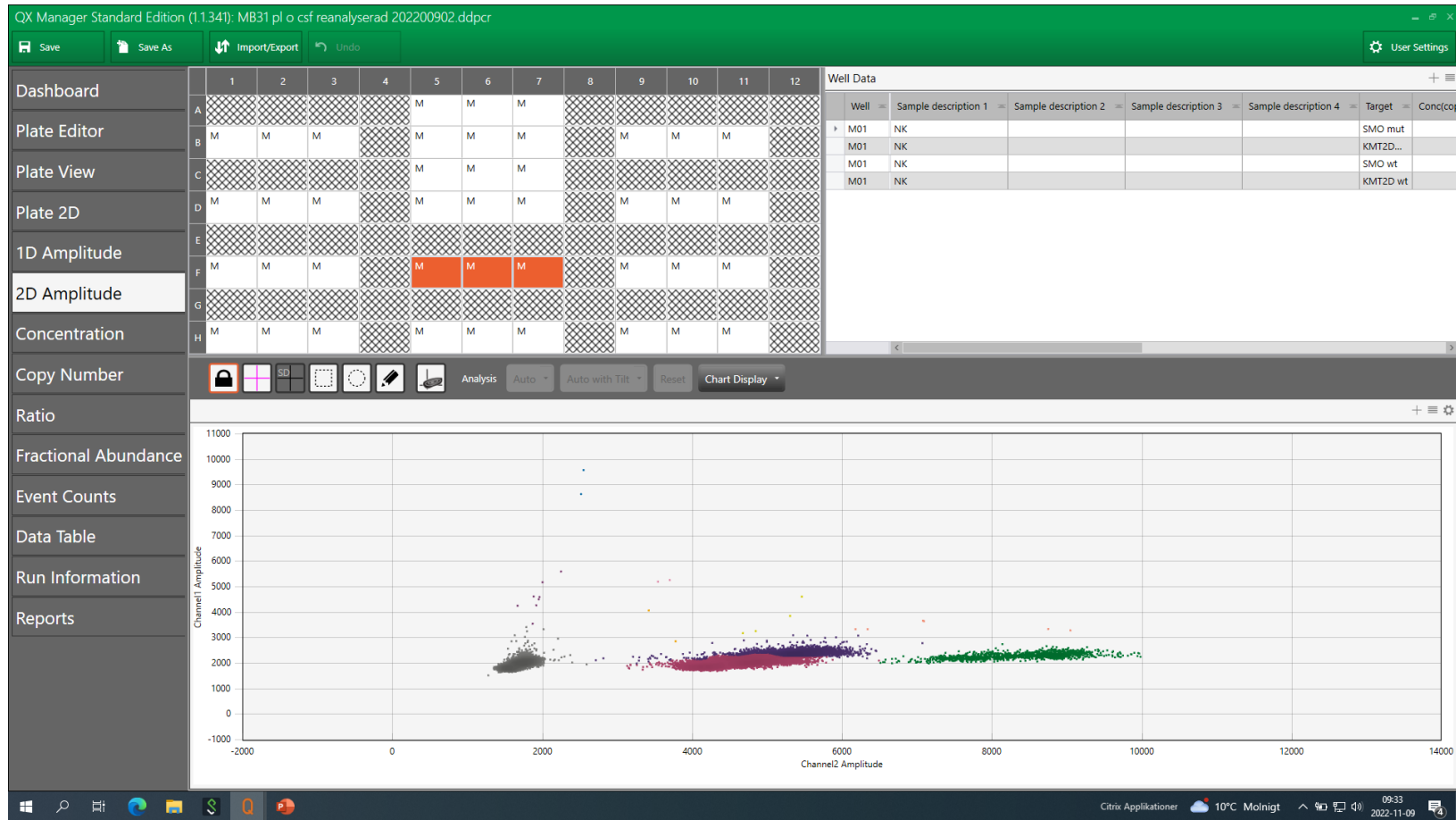

# MB31 Example of clusters

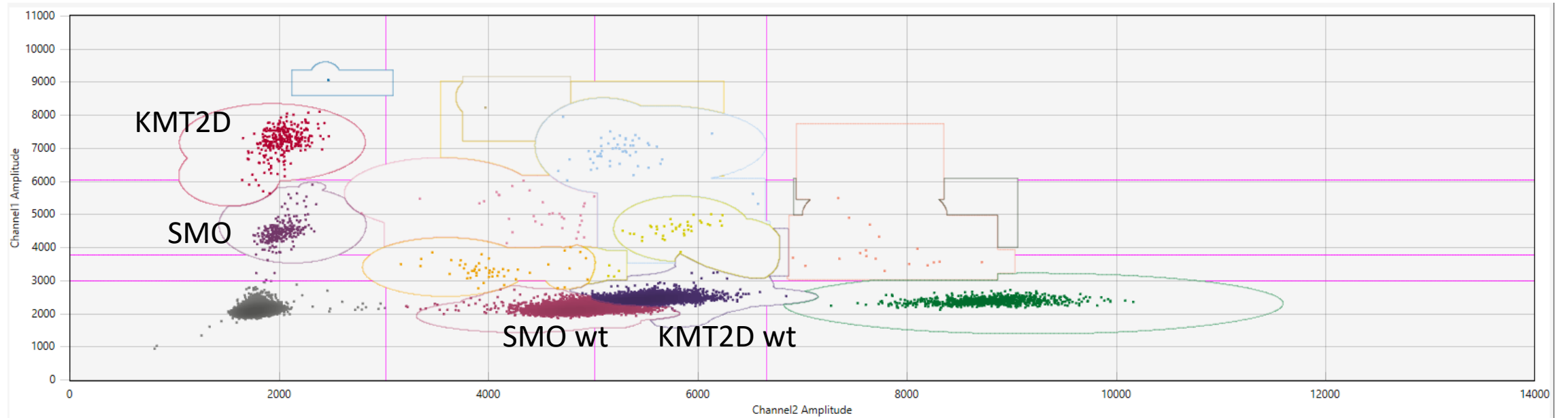

# MB31 1:10

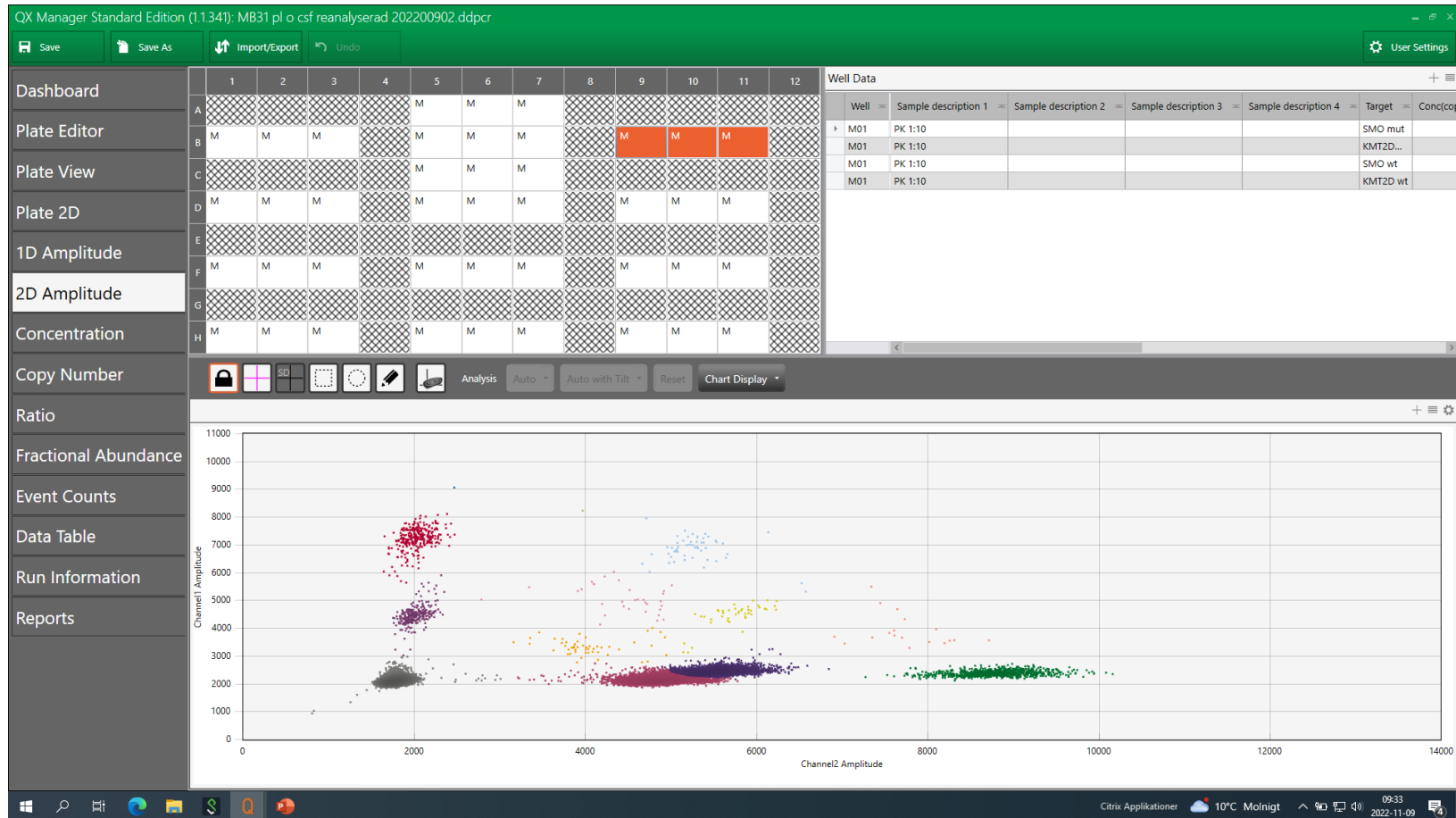

# MB31 1:100

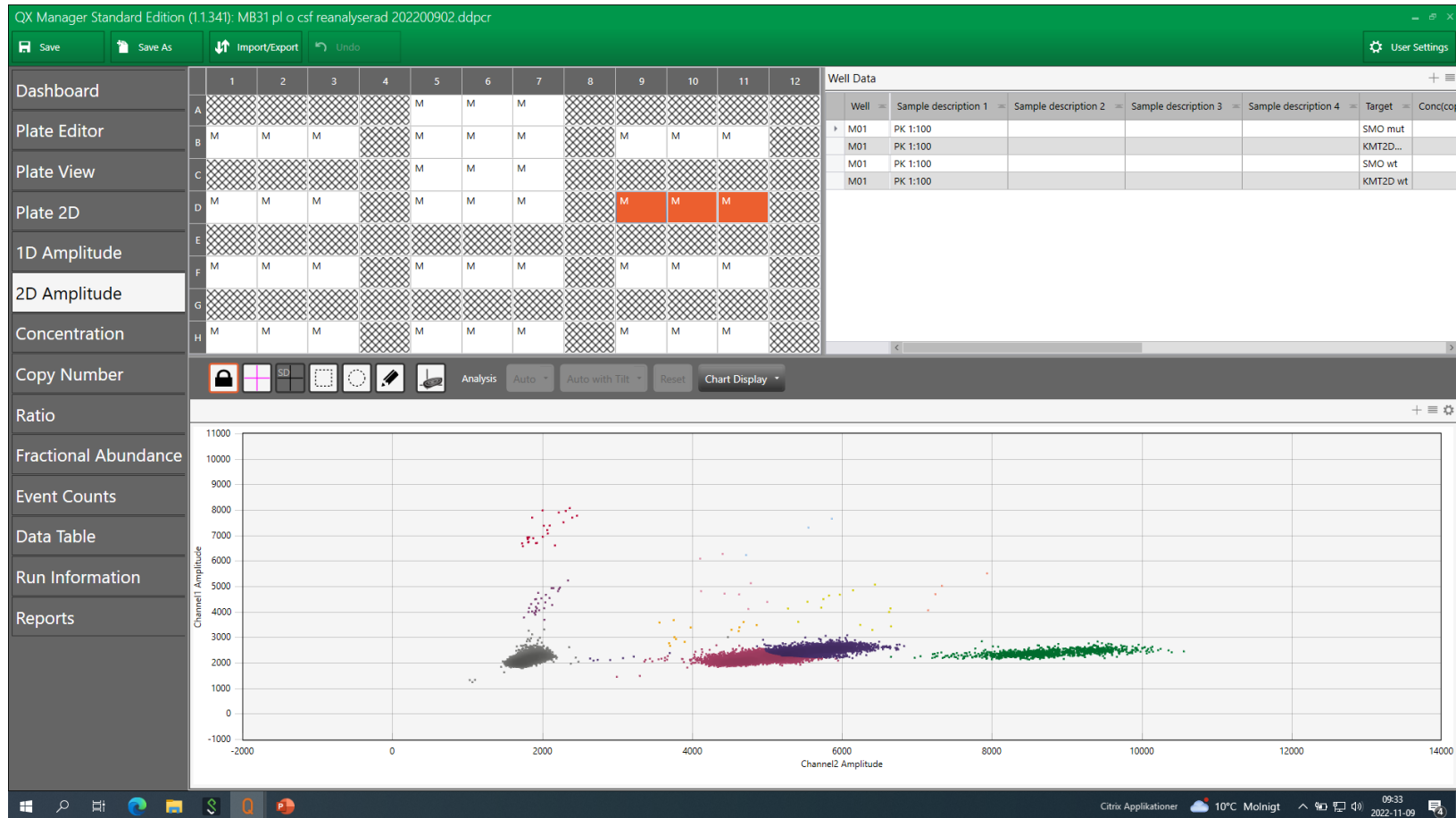

# MB31 1:1000

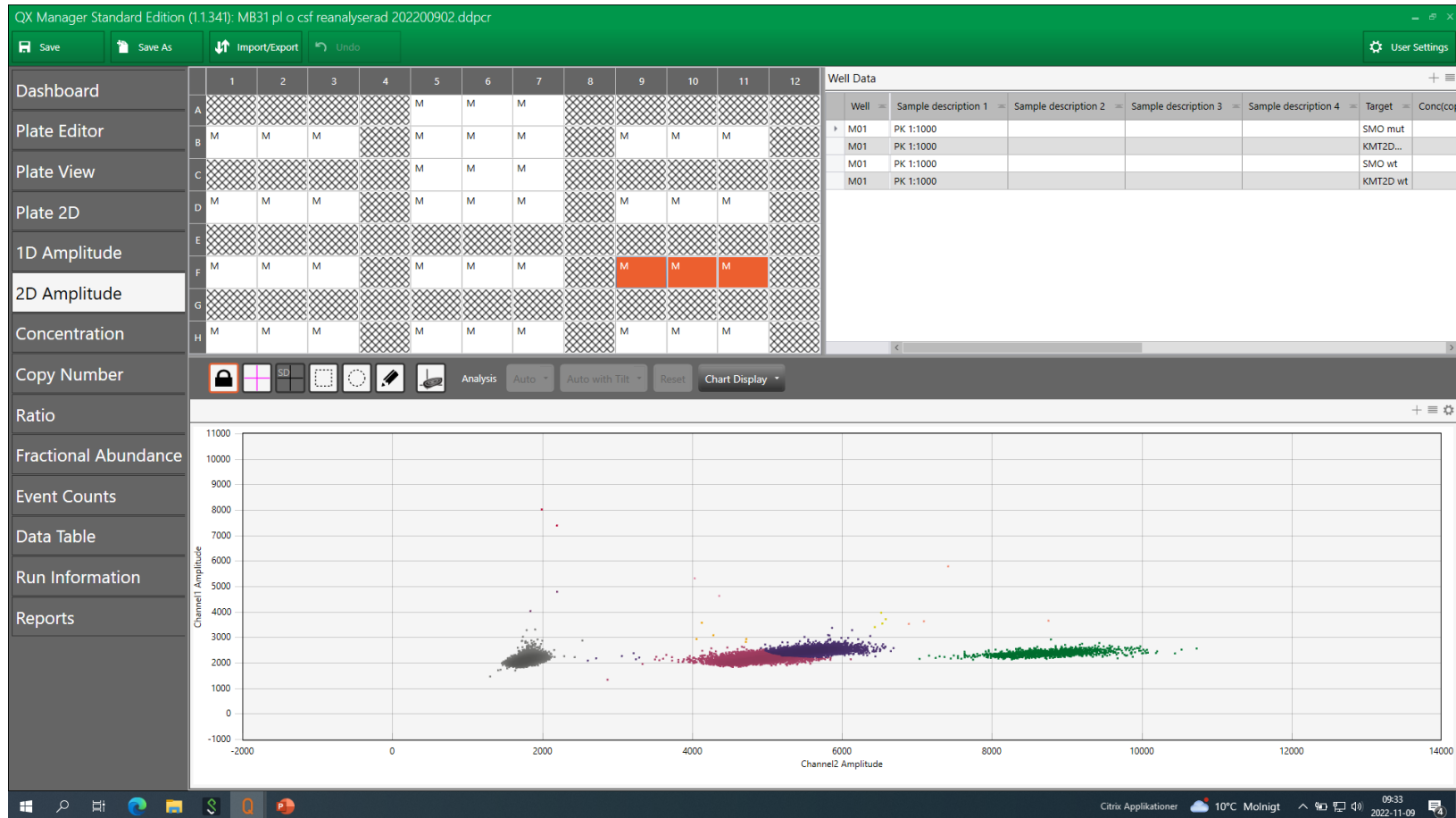

# MB31 1:10000

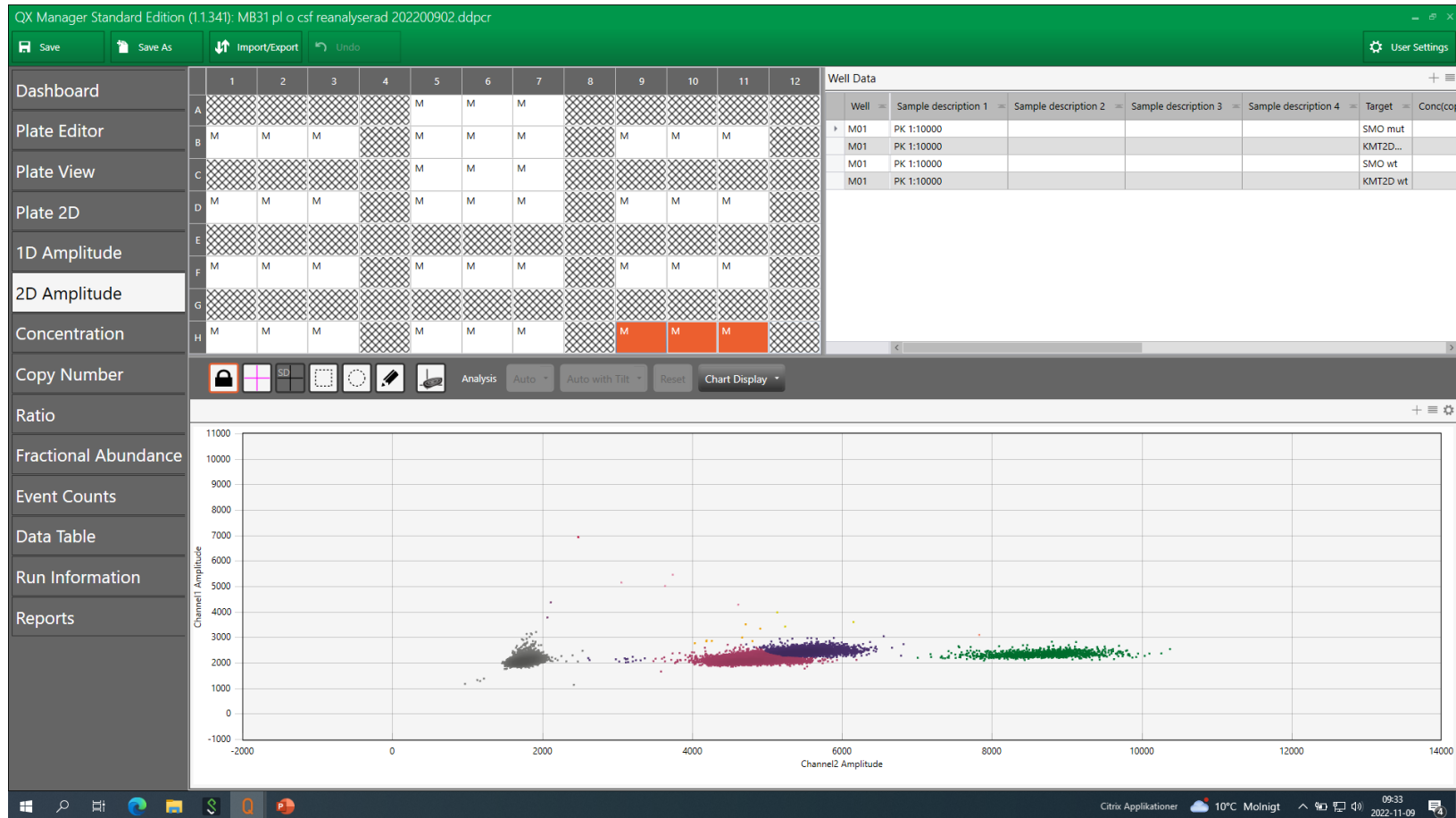

Supplement: Supplementary file 1 [file cancers-15-01972-s001.zip › File S1 QX Manager Software output data on dilution series/Dilution series MB31.pdf]

# MB35 Concentration plot

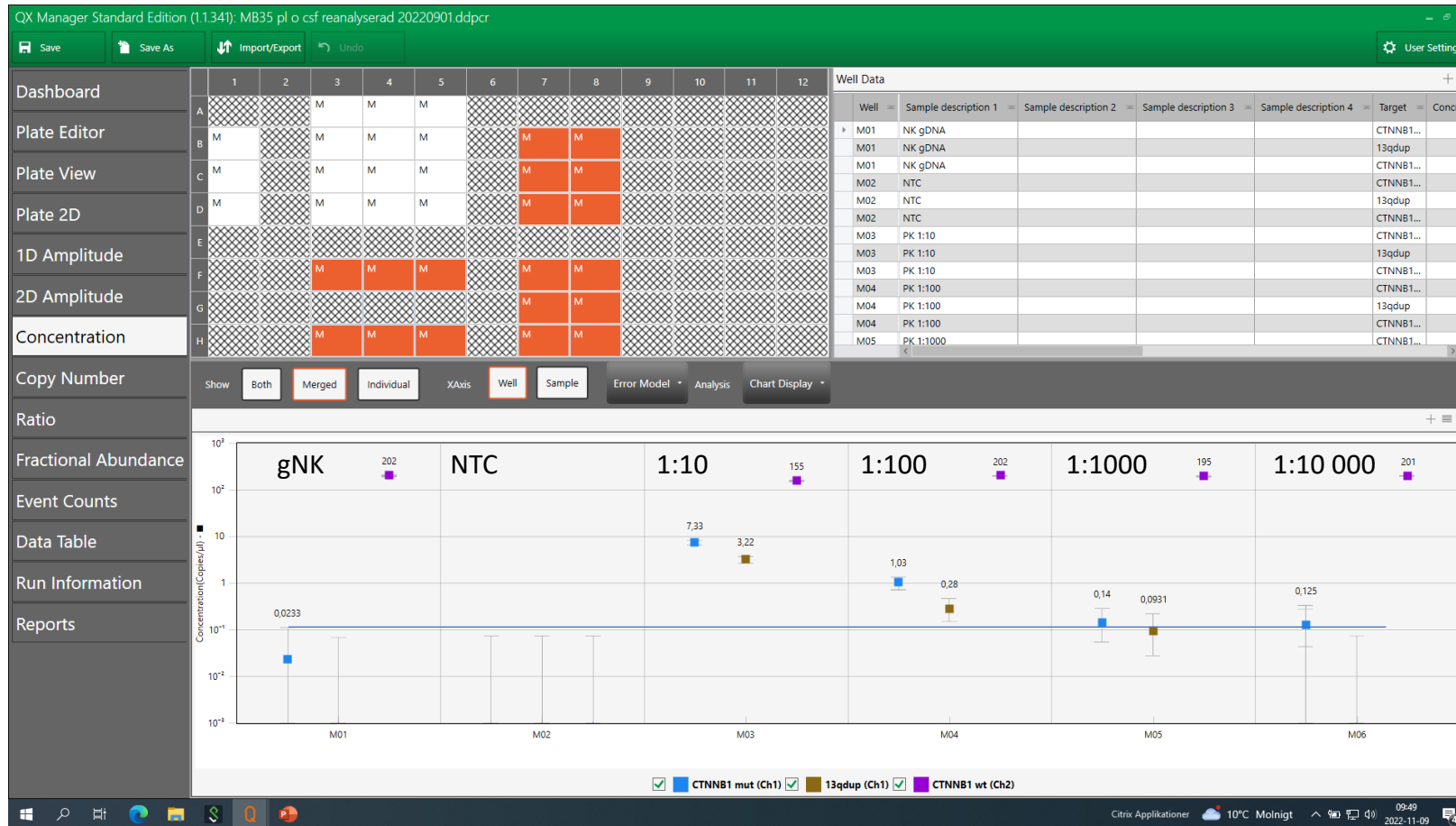

# MB35 NTC

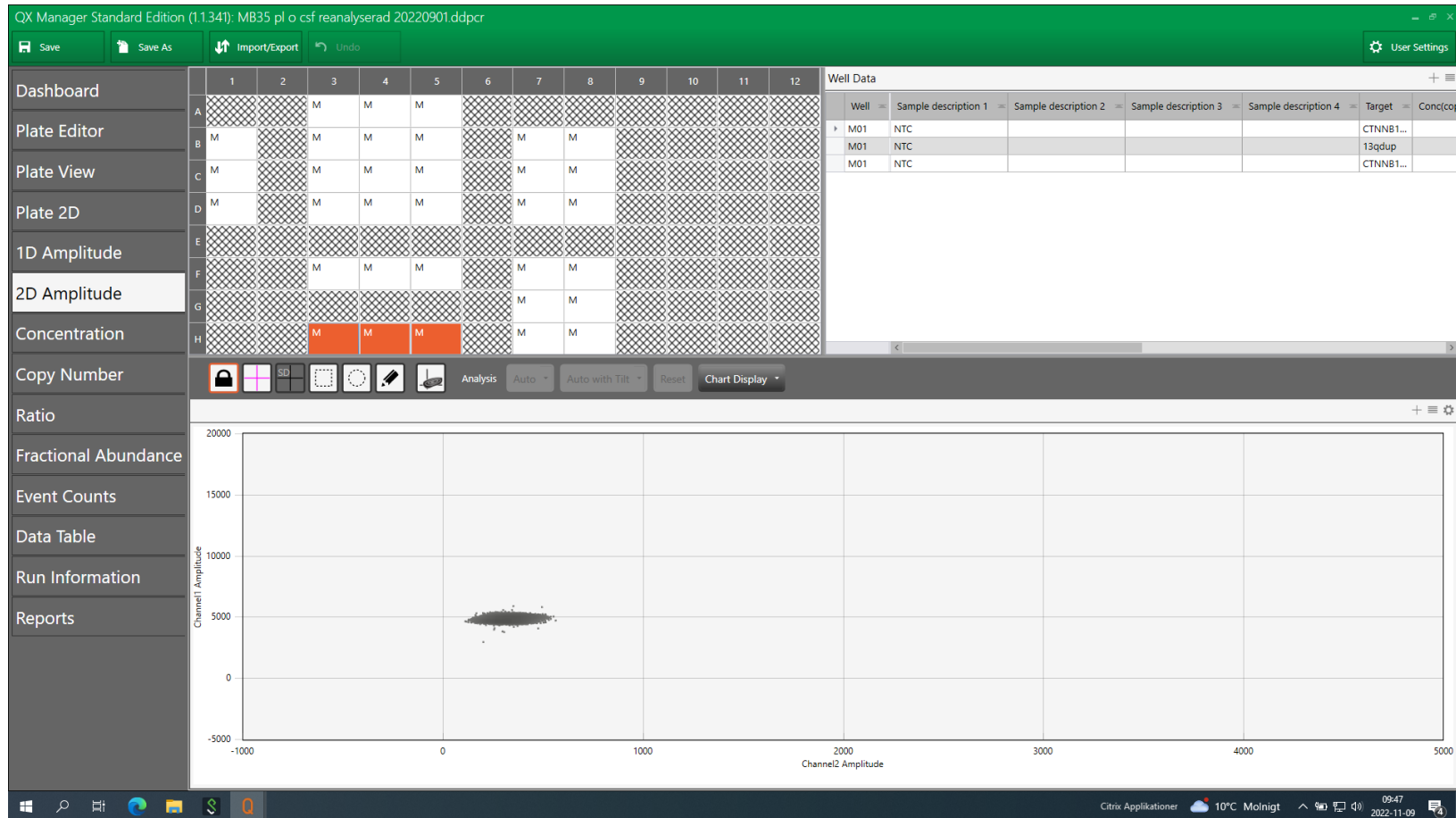

# MB35 gNC

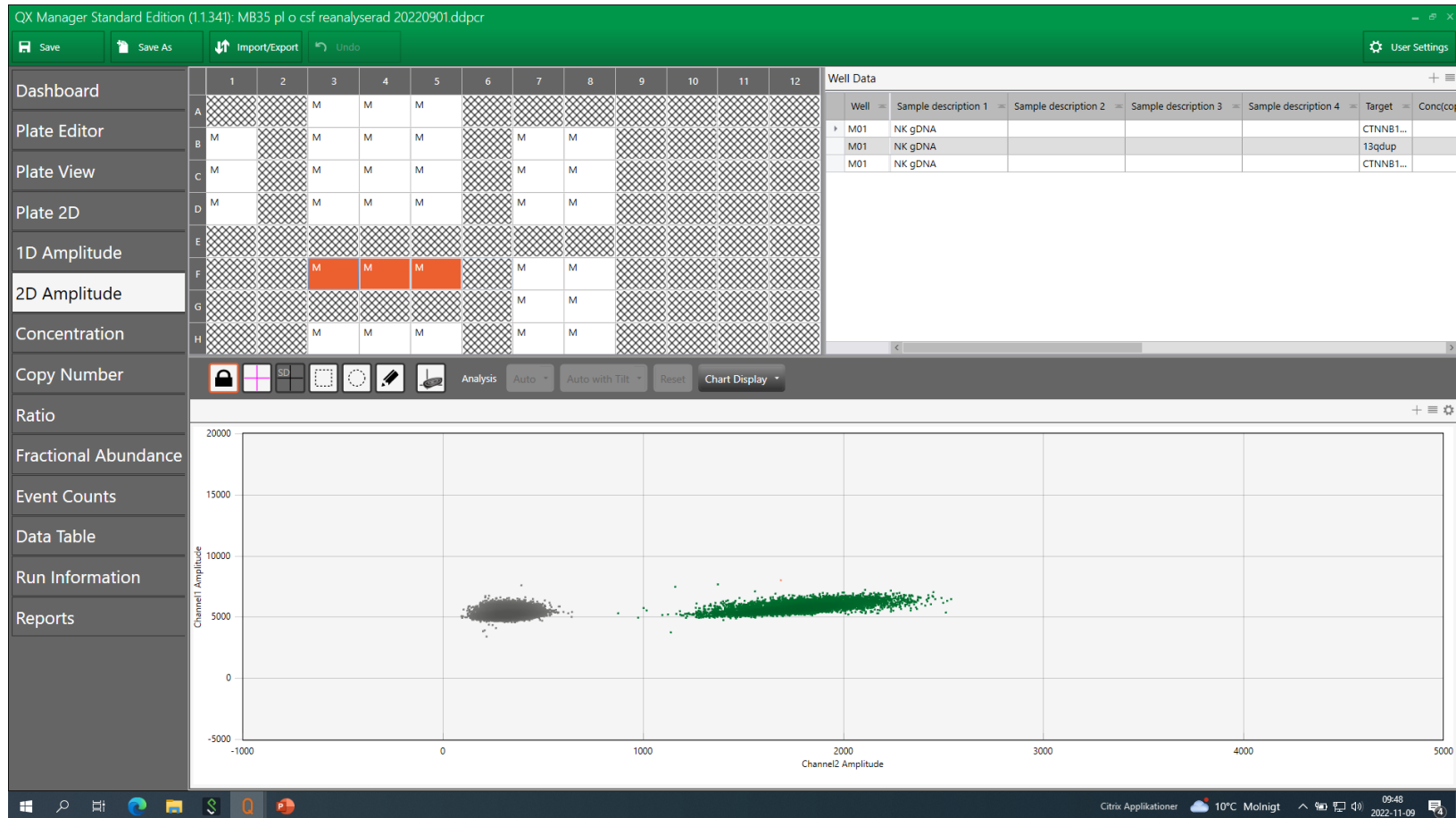

# MB35 Example of clusters

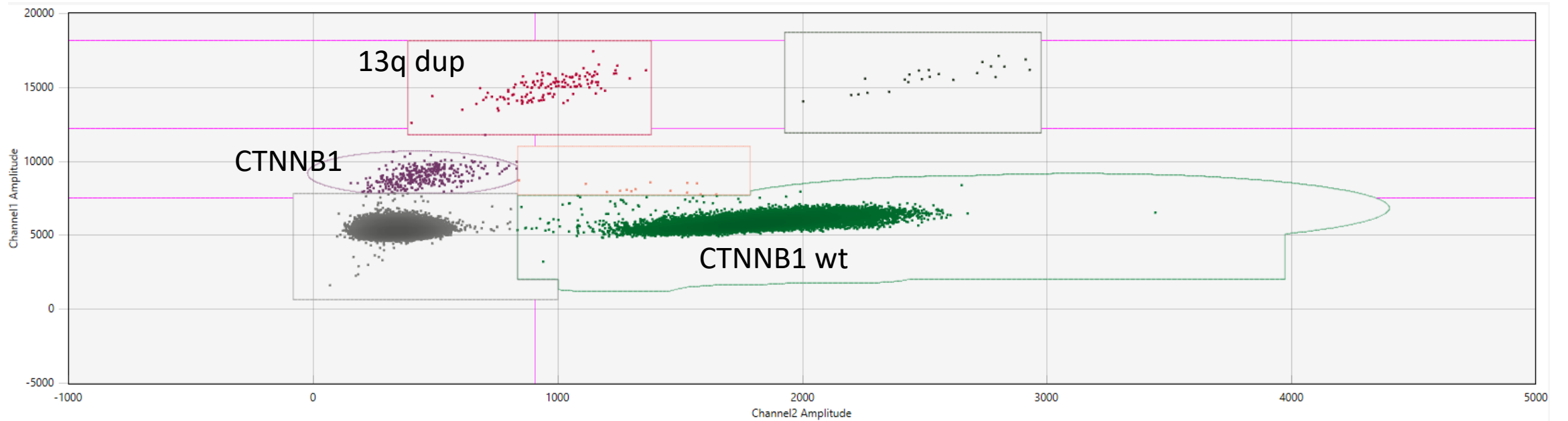

# MB35 1:10

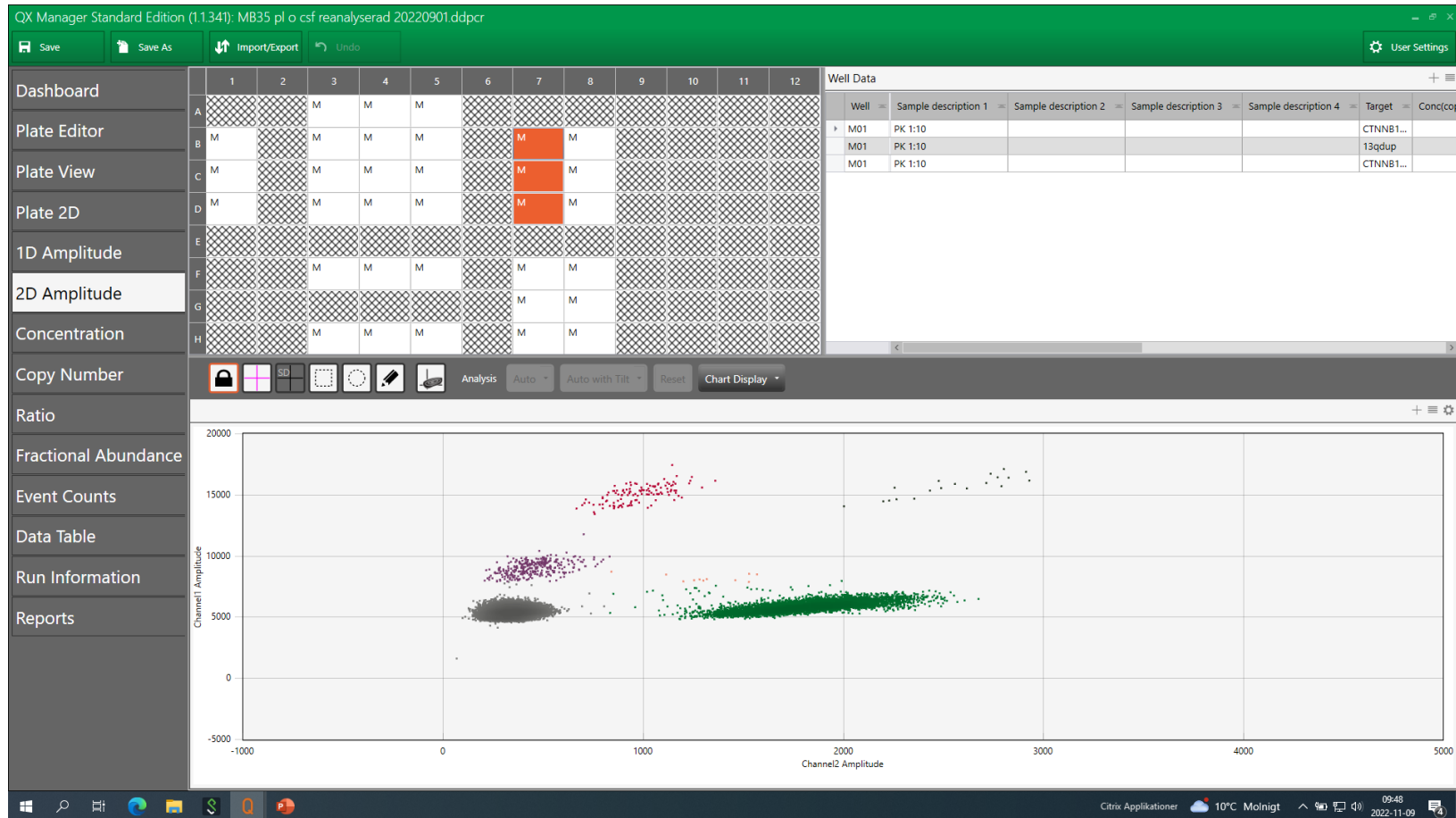

# MB35 1:100

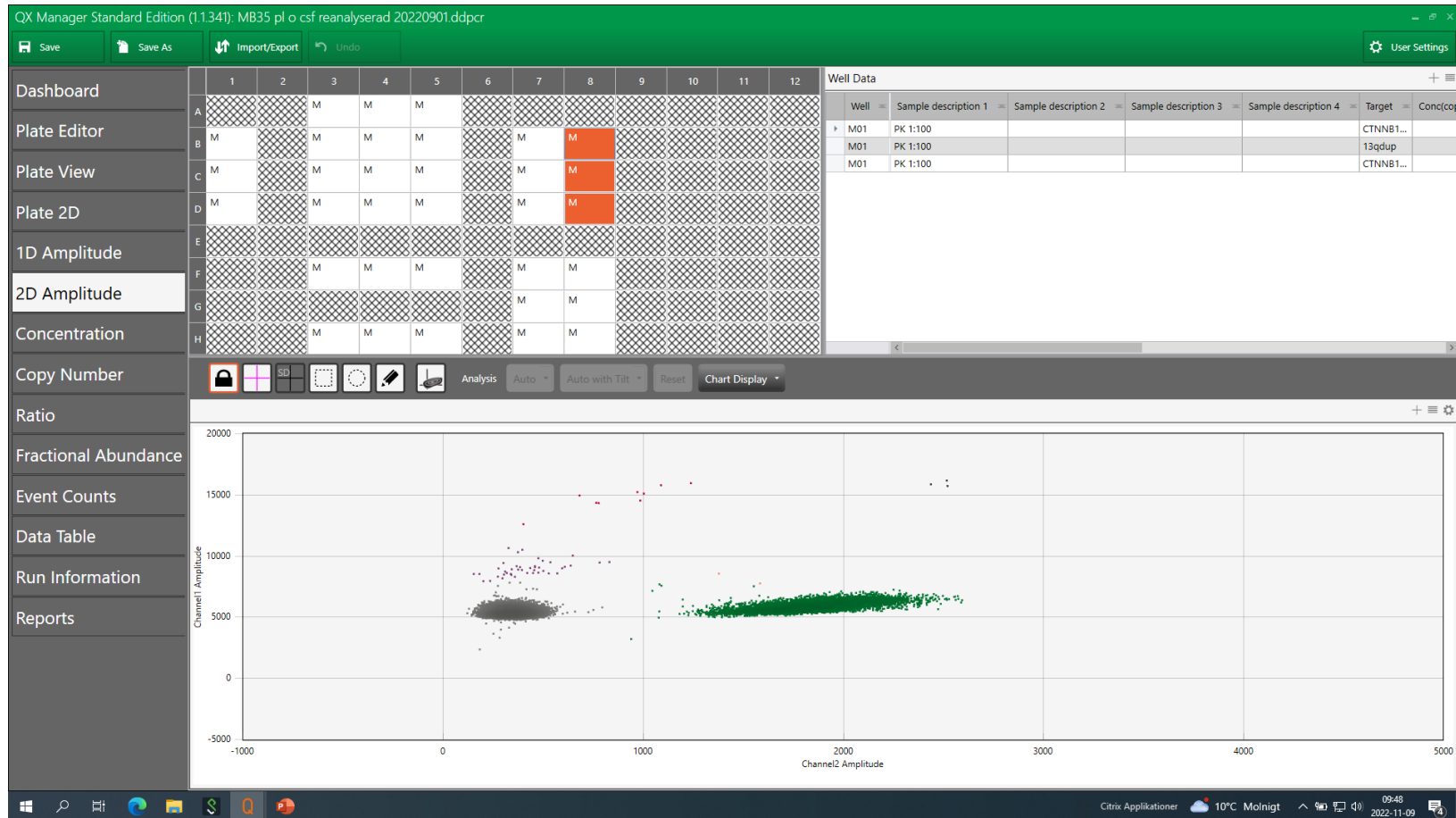

# MB35 1:1000

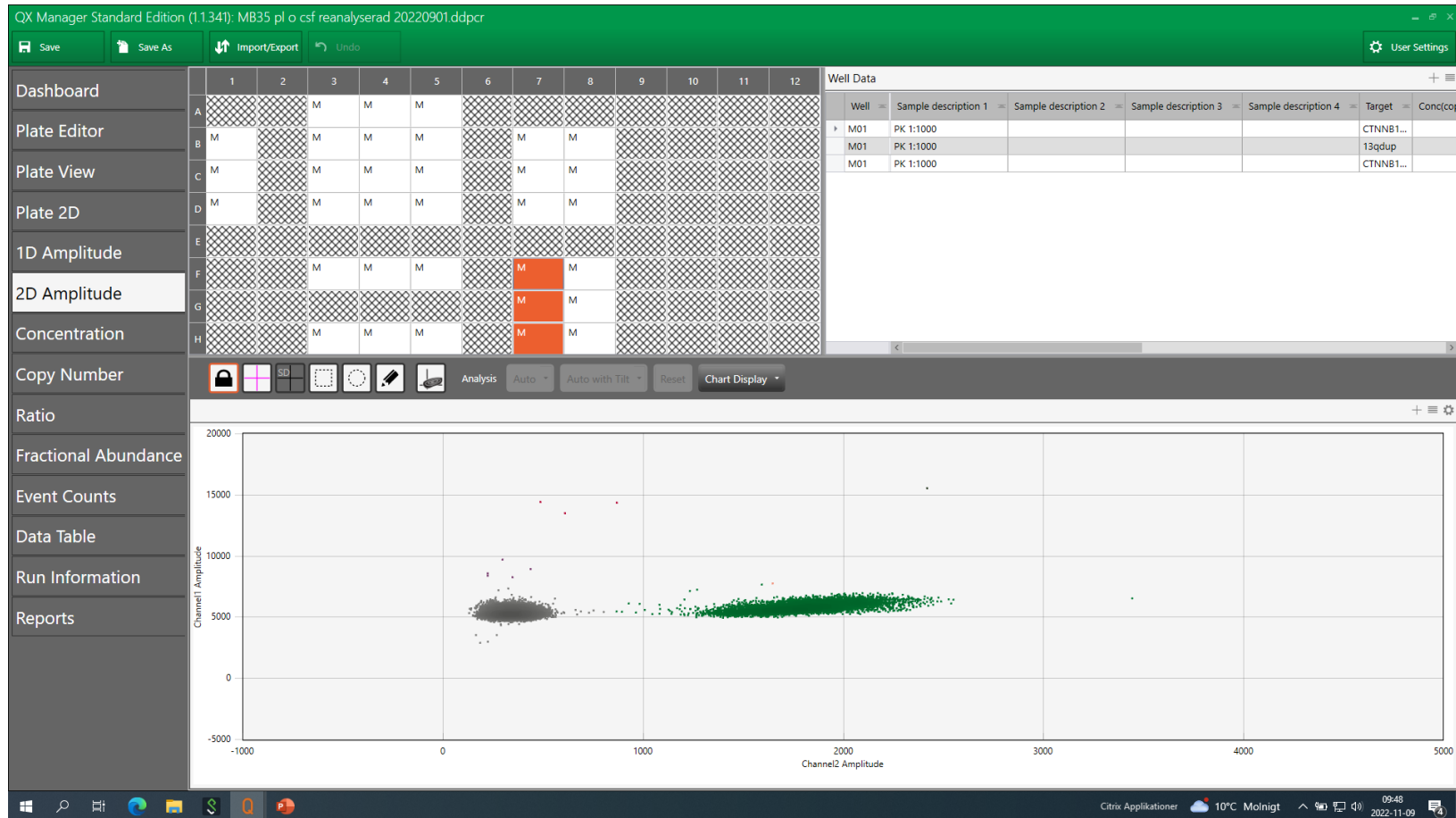

# MB35 1:10000

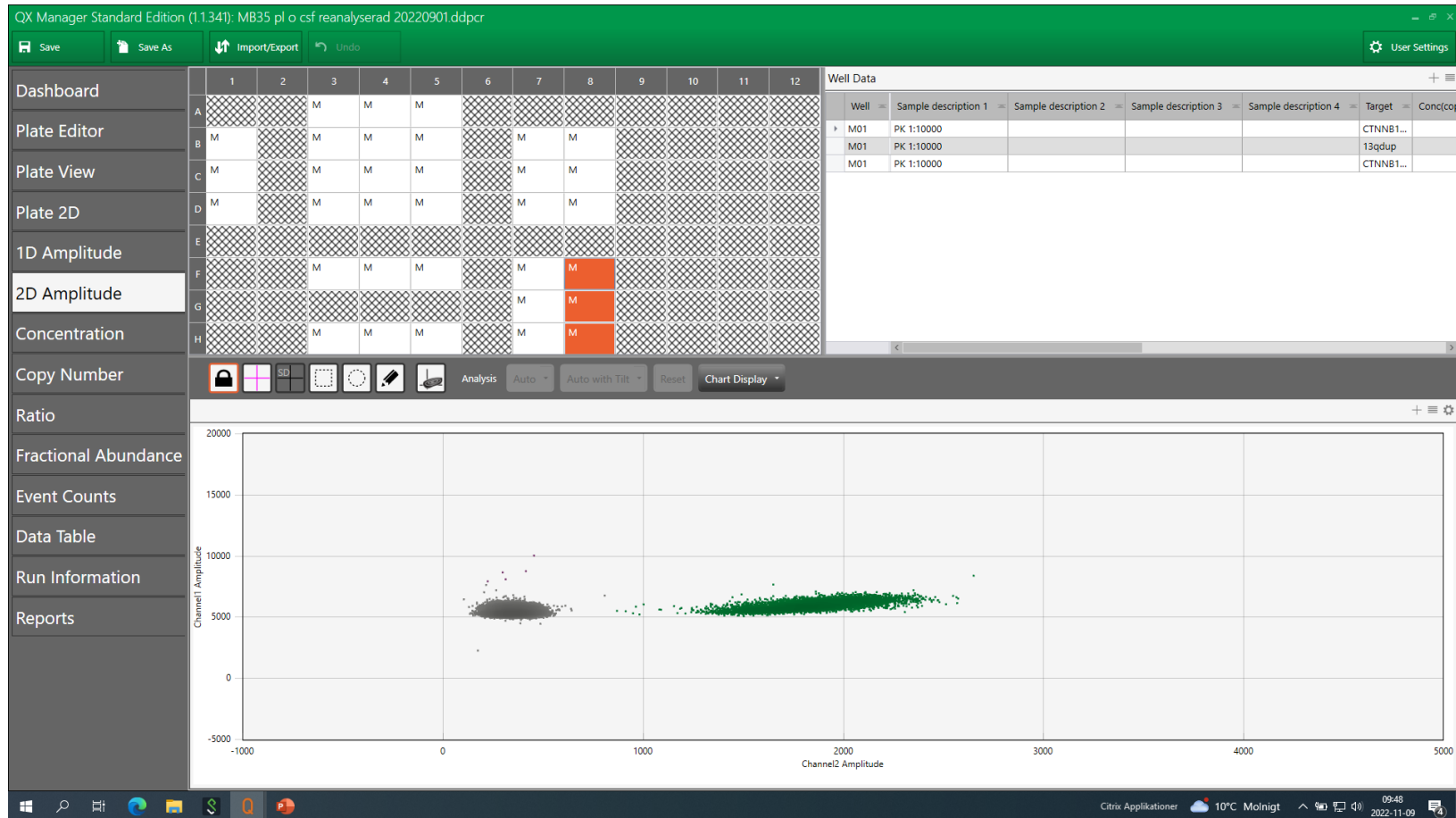

Supplement: Supplementary file 1 [file cancers-15-01972-s001.zip › File S1 QX Manager Software output data on dilution series/Dilution series MB35.pdf]

# MB43 Concentration plot

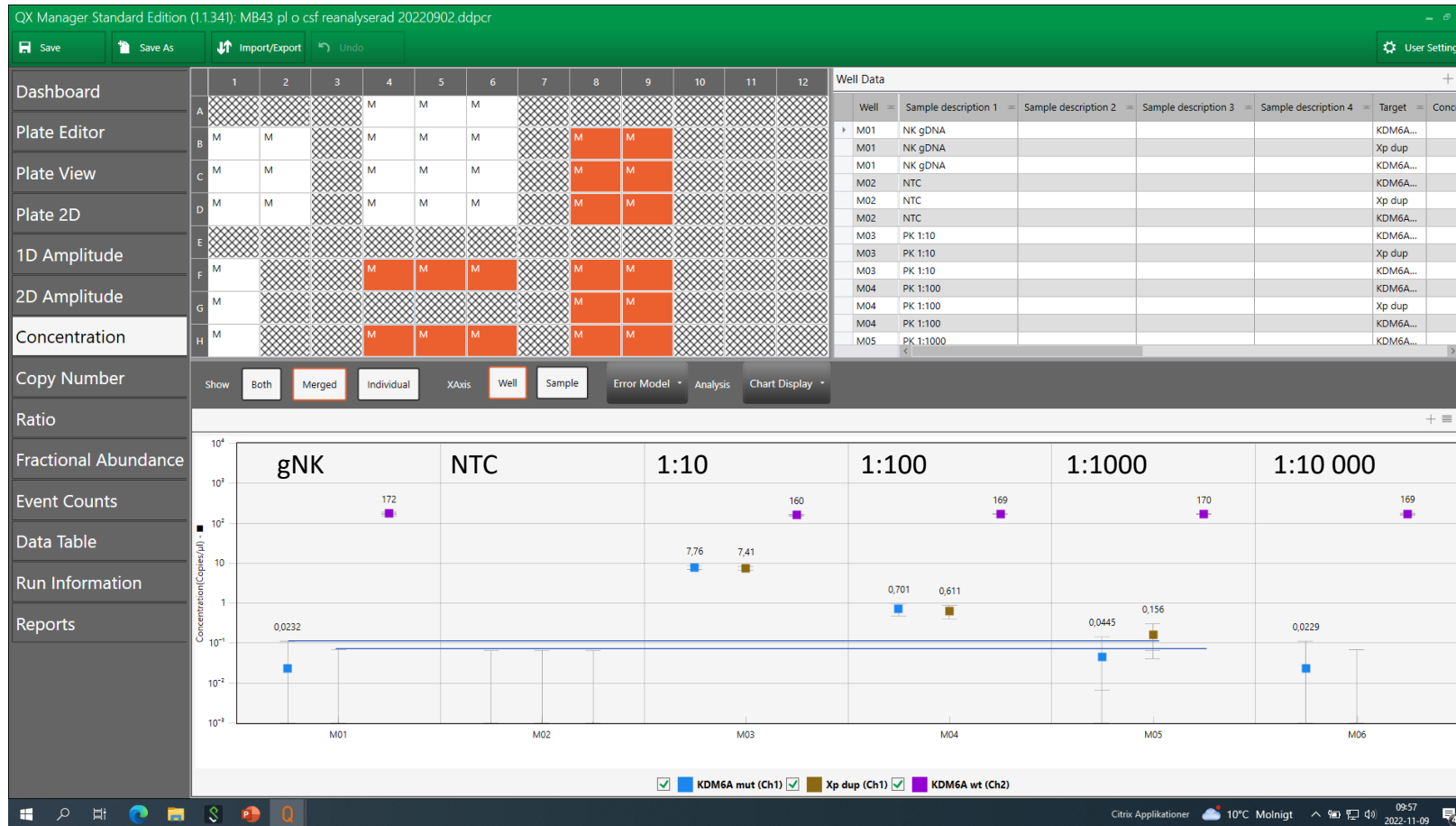

# MB43 NTC

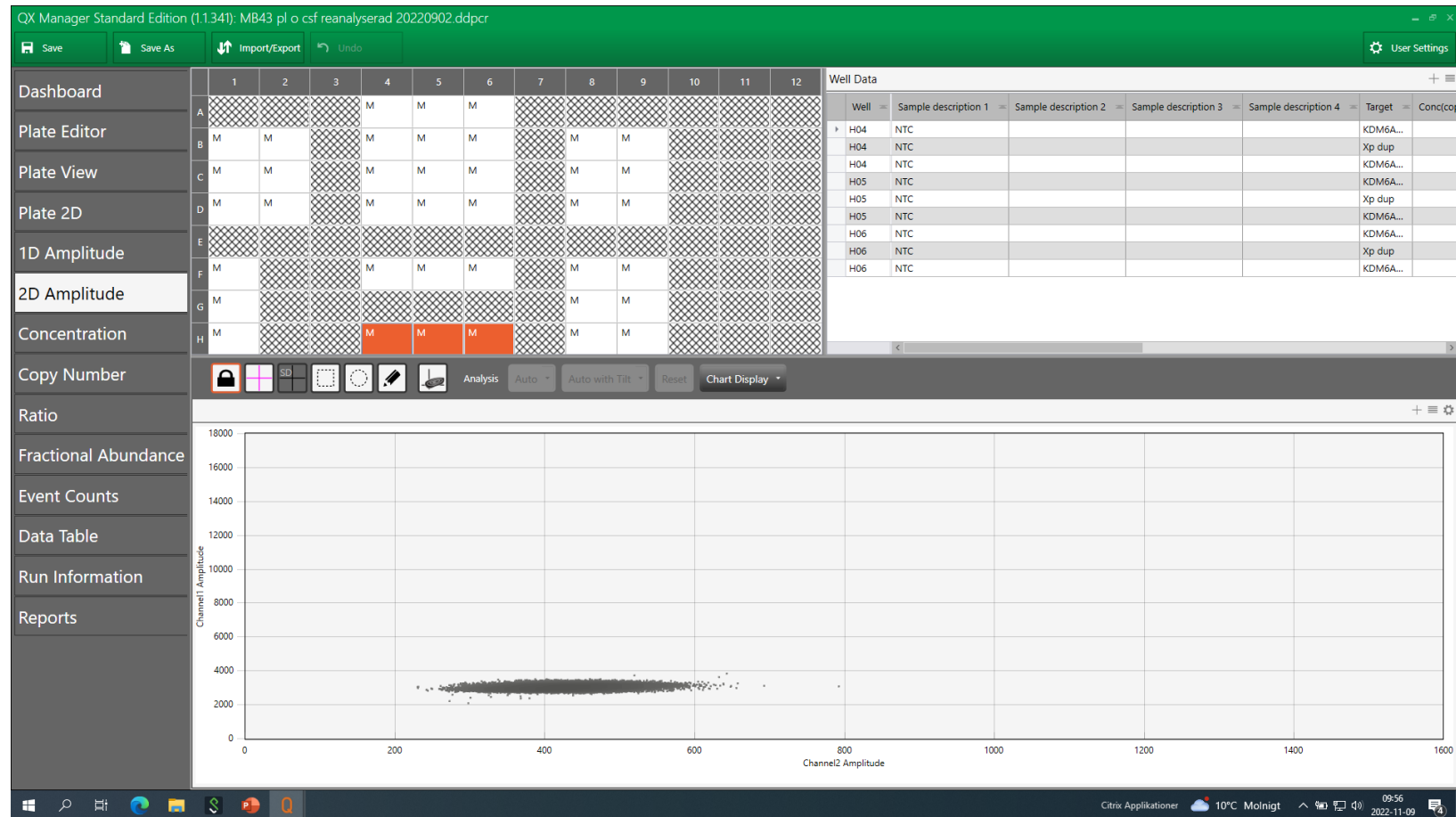

# MB43 gNC

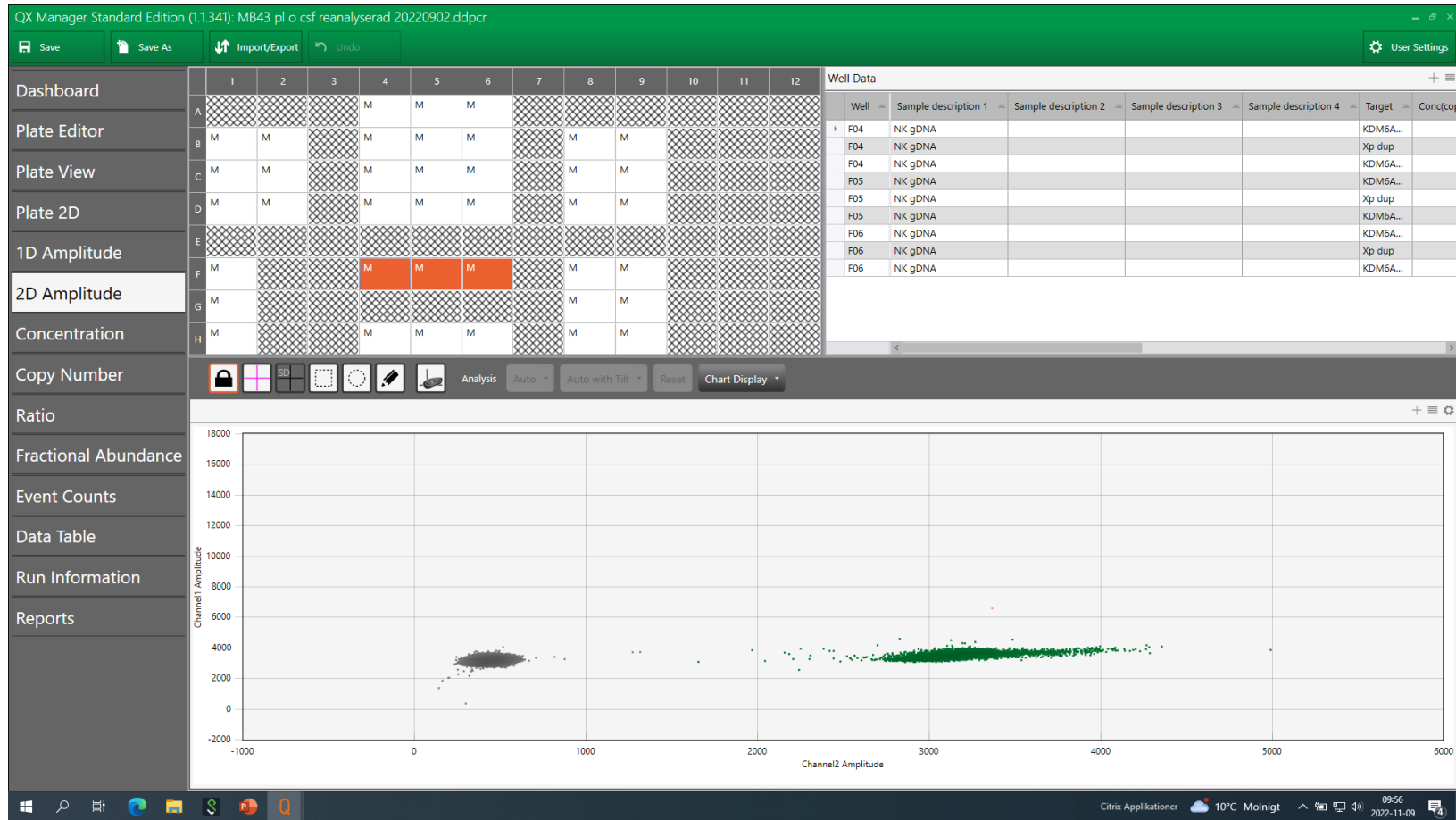

# MB43 Example of clusters

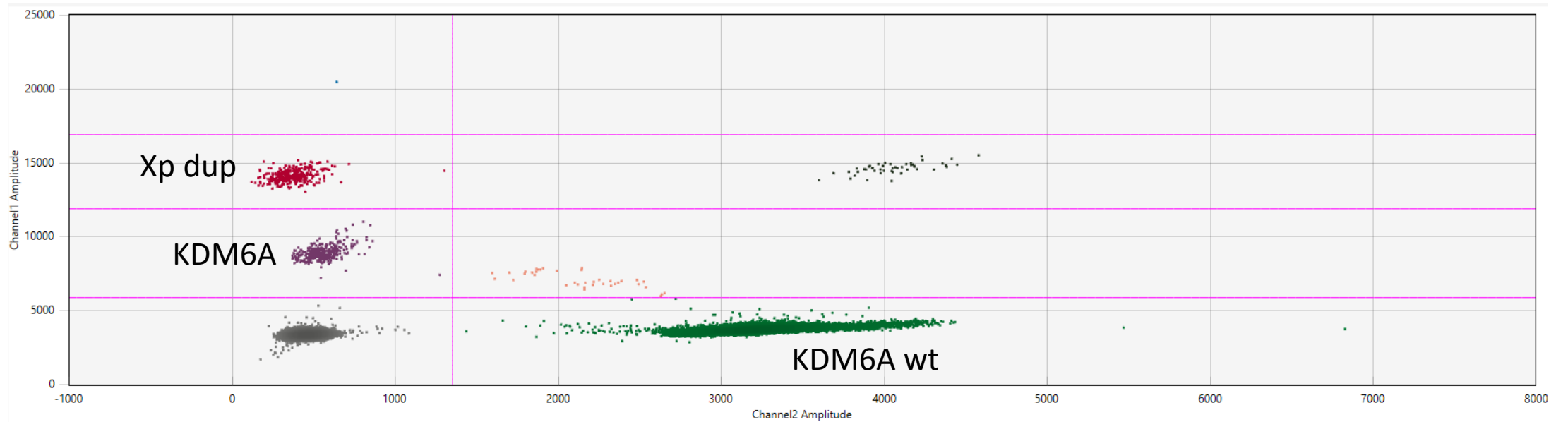

# MB43 1:10

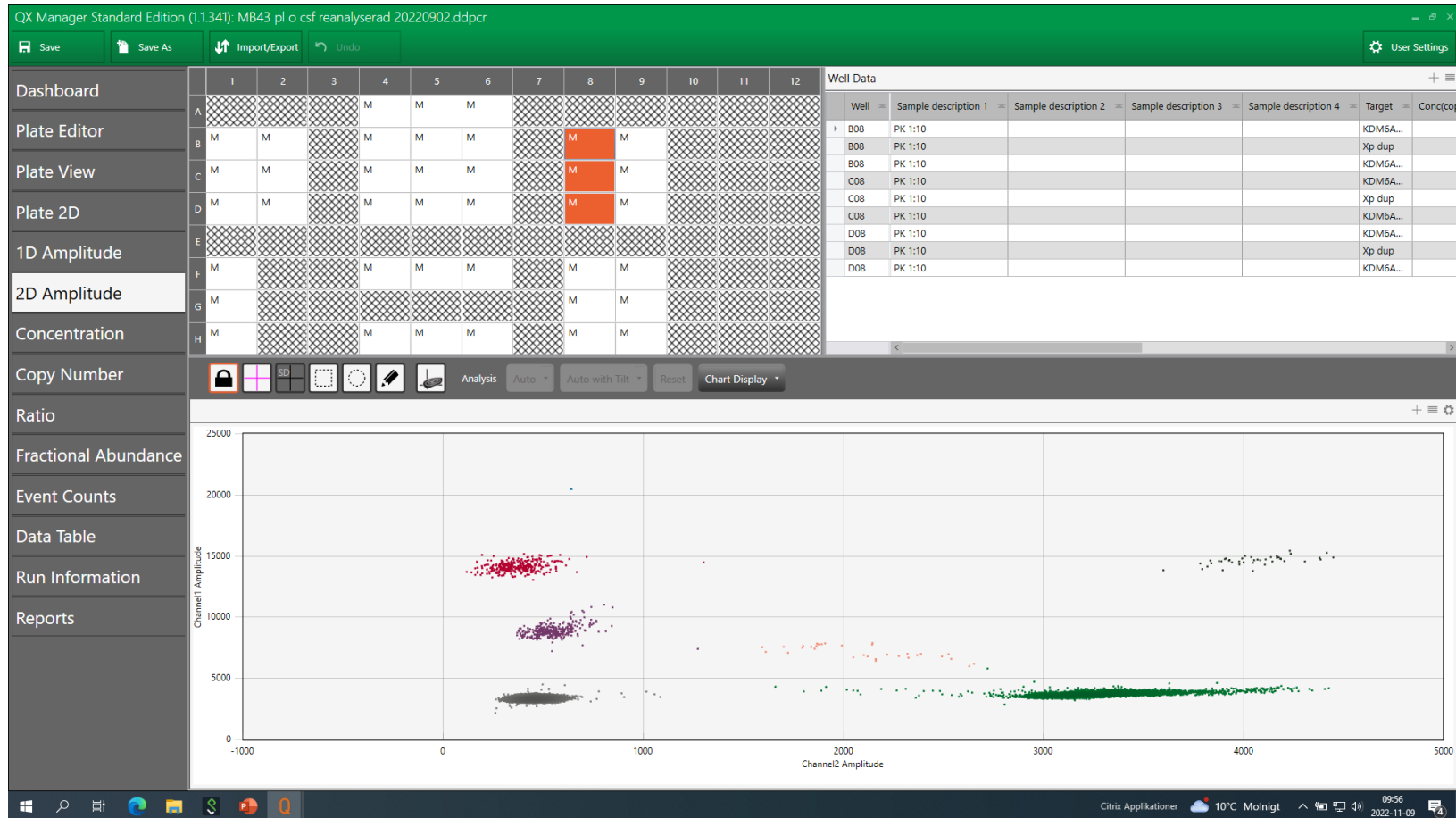

# MB43 1:100

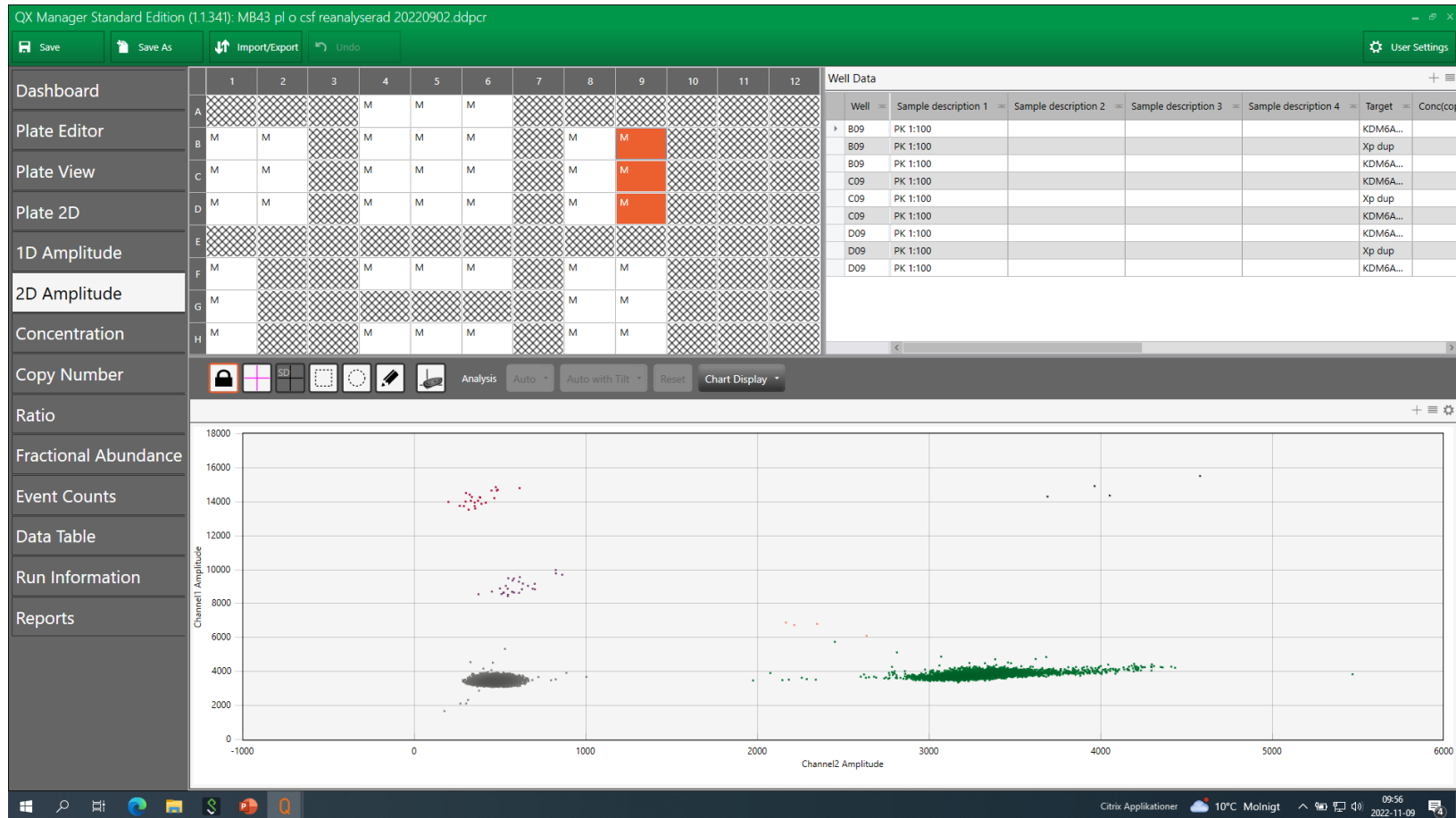

# MB43 1:1000

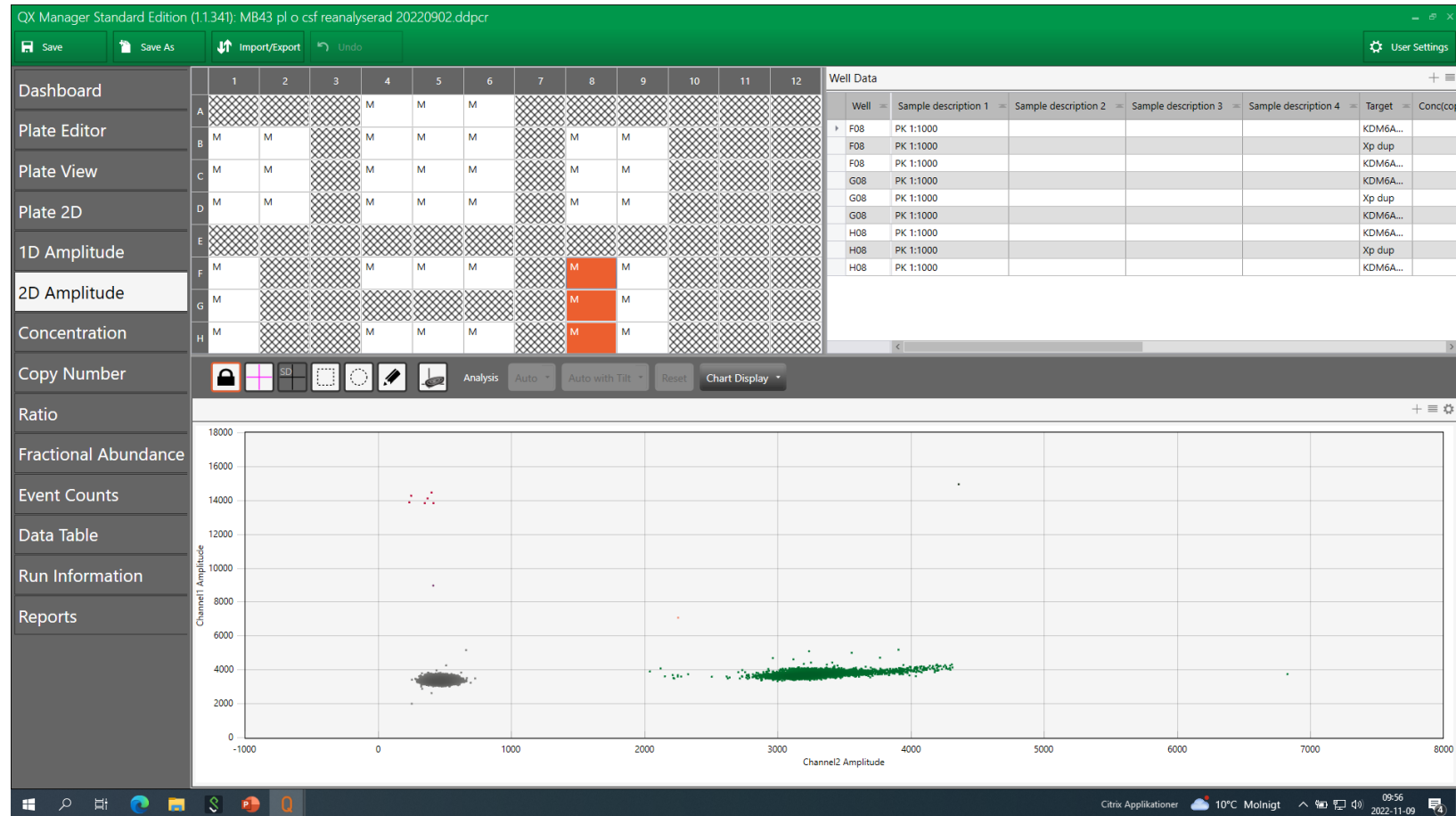

# MB43 1:10000

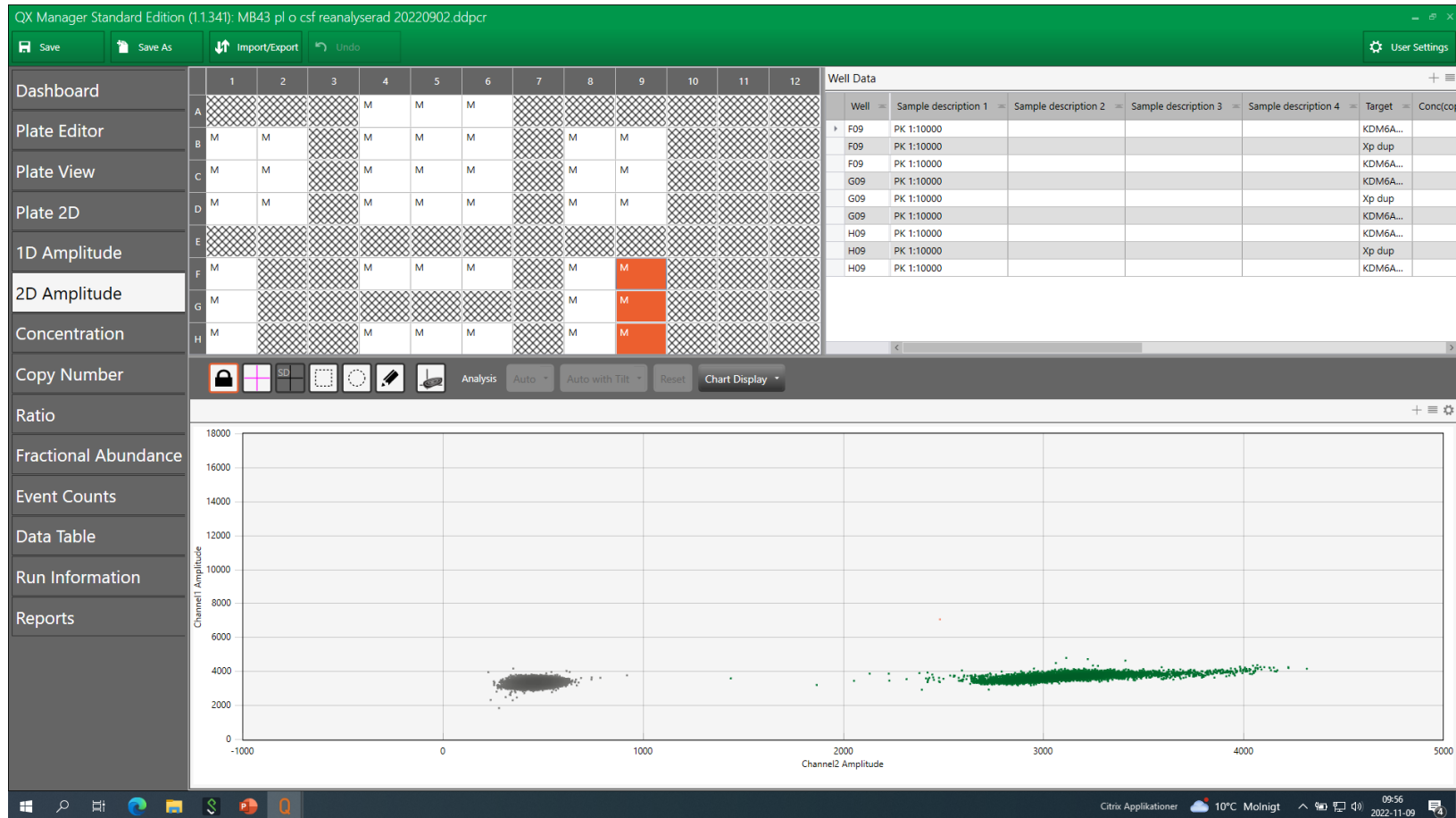

Supplement: Supplementary file 1 [file cancers-15-01972-s001.zip › File S1 QX Manager Software output data on dilution series/Dilution series MB43.pdf]
